# Leveraging genetic overlap between irritability and psychiatric disorders to identify genetic variants of major psychiatric disorders

**DOI:** 10.1038/s12276-023-01005-0

**Published:** 2023-06-01

**Authors:** Kyeongmin Jung, Joohyun Yoon, Yeeun Ahn, Soyeon Kim, Injeong Shim, Hyunwoong Ko, Sang-Hyuk Jung, Jaeyoung Kim, Hyejin Kim, Dong June Lee, Soojin Cha, Hyewon Lee, Beomsu Kim, Min Young Cho, Hyunbin Cho, Dan Say Kim, Jinho Kim, Woong-Yang Park, Tae Hwan Park, Kevin S. O`Connell, Ole A. Andreassen, Woojae Myung, Hong-Hee Won

**Affiliations:** 1 Department of Digital Health, Samsung Advanced Institute for Health Sciences and Technology (SAIHST), Sungkyunkwan University, Samsung Medical Center, Seoul, 06355 South Korea; 2 Department of Neuropsychiatry, Seoul National University Bundang Hospital, Seongnam, 13620 South Korea; 3 Interdisciplinary Program in Cognitive Science, Seoul National University, Seoul, 08826 South Korea; 4 Department of Psychiatry, SMG-SNU Boramae Medical Center, Seoul National University College of Medicine, Seoul, 03080 South Korea; 5 Dental Research Institute, Seoul National University School of Dentistry, Seoul, 03080 South Korea; 6 Department of Health Sciences and Technology, Samsung Advanced Institute for Health Sciences and Technology (SAIHST), Sungkyunkwan University, Samsung Medical Center, Seoul, 06355 South Korea; 7 Department of Health Administration and Management, College of Medical Sciences, Soonchunhyang University, Asan, 31538 South Korea; 8 Precision Medicine Center, Future Innovation Research Division, Seoul National University Bundang Hospital, Seongnam, 13620 South Korea; 9 Samsung Genome Institute, Samsung Medical Center, Sungkyunkwan University School of Medicine, Seoul, 06351 South Korea; 10 Department of Plastic and Reconstructive Surgery, Hallym University Dongtan Sacred Heart Hospital, Dongtan Sacred Heart Hospital, Hallym University College of Medicine, Hwaseong, 18450 South Korea; 11 Norwegian Center for Mental Disorders Research (NORMENT), Institute of Clinical Medicine, University of Oslo and Division of Mental Health and Addiction, Oslo University Hospital, Oslo, NO-316 Norway; 12 Department of Psychiatry, Seoul National University, College of Medicine, Seoul, 03080 South Korea

**Keywords:** Genome-wide association studies, Schizophrenia, Bipolar disorder, Depression

## Abstract

Irritability is a heritable core mental trait associated with several psychiatric illnesses. However, the genomic basis of irritability is unclear. Therefore, this study aimed to 1) identify the genetic variants associated with irritability and investigate the associated biological pathways, genes, and tissues as well as single-nucleotide polymorphism (SNP)-based heritability; 2) explore the relationships between irritability and various traits, including psychiatric disorders; and 3) identify additional and shared genetic variants for irritability and psychiatric disorders. We conducted a genome-wide association study (GWAS) using 379,506 European samples (105,975 cases and 273,531 controls) from the UK Biobank. We utilized various post-GWAS analyses, including linkage disequilibrium score regression, the bivariate causal mixture model (MiXeR), and conditional and conjunctional false discovery rate approaches. This GWAS identified 15 independent loci associated with irritability; the total SNP heritability estimate was 4.19%. Genetic correlations with psychiatric disorders were most pronounced for major depressive disorder (MDD) and bipolar II disorder (BD II). MiXeR analysis revealed polygenic overlap with schizophrenia (SCZ), bipolar I disorder (BD I), and MDD. Conditional false discovery rate analyses identified additional loci associated with SCZ (number [*n*] of additional SNPs = 105), BD I (*n* = 54), MDD (*n* = 107), and irritability (*n* = 157). Conjunctional false discovery rate analyses identified 85, 41, and 198 shared loci between irritability and SCZ, BD I, and MDD, respectively. Multiple genetic loci were associated with irritability and three main psychiatric disorders. Given that irritability is a cross-disorder trait, these findings may help to elucidate the genomics of psychiatric disorders.

## Psychiatry: Genetic associations between irritability and major psychiatric disorders

Identifying genetic variants associated with irritability, a heritable condition, has revealed some variants that are also associated with major psychiatric disorders. Researchers in Seoul, South Korea, led by Hong-Hee Won at Sungkyunkwan University and Woojae Myung at Seoul National University, performed a genome-wide association study with 379,506 individuals of European ancestry from the UK Biobank database. The study compared millions of genetic variants between 105,975 people with self-reported irritability and 273,531 controls. Multiple genetic analyses illustrated the genetic overlap between irritability and psychiatric disorders such as major depressive disorder, bipolar disorder, and schizophrenia. The study also investigated specific biological pathways associated with these variants. The findings help shed light on the genetic basis of psychiatric disorders, which may pave the way for new treatment.

## Introduction

Irritability is an emotional response characterized by disproportionate reactivity to negative stimuli^1^. Irritability can also be defined as a temperament type with a predisposition to feeling negative emotions (i.e., anger, annoyance, envy, frustration) and a lack of control over excessive emotional responses^1,2^.

Various psychopathological outcomes have been associated with irritability. Correlational and longitudinal studies have shown that irritability during adolescence predicts major depressive disorder (MDD) and generalized anxiety disorder in adulthood and is associated with current depressive symptoms, neuroticism, impulsivity, borderline personality pathology, and suicidality^3–5^. Genetic studies have suggested that irritability is genetically associated with various psychiatric illnesses. For instance, studies have indicated that a genetic predisposition toward schizophrenia (SCZ), attention-deficit/hyperactivity disorder (ADHD), and MDD can manifest as irritability^6–8^. In addition, youth at familial risk of bipolar disorder (BD) are more likely to demonstrate chronic irritability than controls; chronic irritability in turn is associated with increased rates of BD, depressive disorders, disruptive behavior disorders, and ADHD^9^. Moreover, irritability is a diagnostic criterion for the following mental illnesses: generalized anxiety disorder, depressive disorders (disruptive mood dysregulation disorder, MDD, and persistent depressive disorder), bipolar I disorder (BD I) and bipolar II disorder (BD II), acute stress disorder and posttraumatic stress disorder, oppositional defiant disorder, and personality disorders (antisocial and borderline)^10^. Essentially, irritability is a common component of various psychiatric disorders and bridges the internalizing–externalizing divide.

Irritability is heritable. Twin studies have shown that its heritability estimates range from 31% to 37%^11,12^. While prior studies have examined the neurophysiological associations of irritability with brain structures and their respective functions^13,14^, there is a paucity of literature on the genome-wide basis of irritability. The genetic relationship between irritability and psychiatric disorders is an emerging issue that merits further exploration.

In the present study, we had three main objectives: 1) to clarify the genetic architecture of irritability by identifying associated genetic variants via a genome-wide association study (GWAS) and investigating the associated biological pathways, genes, and tissues as well as single-nucleotide polymorphism (SNP)-based heritability via post-GWAS analyses; 2) to explore the relationships between irritability and various traits, as well as psychiatric disorders, using linkage disequilibrium score regression (LDSC) and the bivariate causal mixture model (MiXeR); and 3) to apply conditional false discovery rate (condFDR) and conjunctional false discovery rate (conjFDR) approaches to identify additional and shared genetic variants for irritability and psychiatric disorders via overlapping SNP-based associations.

## Materials and methods

### UK Biobank

The UK Biobank is a nationwide cohort of approximately 500,000 individuals aged between 40 and 69 years at the time of recruitment. Individuals were recruited during a four-year baseline period (from 2006 to 2010) at multiple centers located in the UK. The collected data include computer-assisted interviews, touchscreen-based self-report questionnaires, physical health measures, and biological samples (including genotype data). More details regarding the UK Biobank can be found at https://www.ukbiobank.ac.uk/about-biobank-uk. For the present study, individuals were excluded if they were non-European, if their genetically determined sex did not match their self-reported sex, if they presented with sex chromosome aneuploidy, if they did not provide sex information, or if they withdrew from the UK Biobank. Finally, 379,506 participants of European ancestry who answered the baseline irritability question (“Are you an irritable person?”) were included in this study.

### Phenotype measures

The UK Biobank included the above irritability question as part of the baseline assessment. Participants who responded “yes” to this question (“Are you an irritable person?”) were defined as irritable cases, and those who responded “no” were defined as controls. A total of 379,506 participants of European ancestry who answered the above irritability question were included in the analysis (cases, *n* = 105,975; controls, *n* = 273,531; Supplementary Table 1). All participants provided written informed consent. The National Research Ethics Committee (REC reference 11/NW/0382) approved the UK Biobank study, and this secondary analysis was conducted in accordance with the principles of the Declaration of Helsinki and its later amendments.

### Genotyping and quality control

The UK Biobank released the genetic data for 487,409 individuals in March 2018 (version 3). These samples were genotyped using either Affymetrix UK BiLEVE Axiom or Affymetrix UK Biobank Axiom arrays (Santa Clara, CA, USA), which include > 800,000 variants. The UK Biobank researchers applied extensive quality control (QC) procedures to the genotype data^15^. Imputation was performed centrally by the UK Biobank using combined data from the 1000 Genomes Project and the UK10K panel; SHAPEIT3 was used for phasing, and IMPUTE2 was used for imputation^16,17^. Additionally, we excluded variants with a call rate < 0.95, a Hardy–Weinberg equilibrium of *P* < 1 × 10^−6^, a minor allele frequency (MAF) < 0.5%, or imputation quality scores (INFO) < 0.4, as in a previous study^18^. These QC criteria resulted in the inclusion of 9,575,249 SNPs.

### Genome-wide association analyses

We performed genome-wide association analyses using the scalable and accurate implementation of the generalized mixed model (SAIGE)^19^. This is a logistic mixed-model approach that analyzes binary traits with unbalanced case‒control ratios. This method also accounts for sample relatedness and effectively controls the inflation of type I error rates using saddle point approximation. Age, sex, genotyping array type, and the first 10 principal components of genetic ancestry supplied by the UK Biobank (field 22009) were included as covariates in the multivariate analyses as reported in a previous study^20^. We used 844,770 pruned genotyped markers to estimate the genetic relationship matrix with the following parameters: a window size of 500 base pairs (bp), a step size of 50 bp, and a pairwise *r*^2^ values < 0.2.

The summary statistics were processed via linkage disequilibrium (LD) clumping using PLINK software (https://zzz.bwh.harvard.edu/plink) to define regional lead SNPs with *r*^2^ values > 0.2^21^. Variants that exceeded the genome-wide significance threshold of *P* < 5 × 10^−8^ were identified as associated with irritability. LD was calculated using UK Biobank samples that had passed the same QC criteria that were used for the current GWAS. Regional plots of these variants were generated using LocusZoom software (http://locuszoom.sph.umich.edu)^22^.

### Sensitivity analysis

As a sensitivity analysis, we conducted a two-stage GWAS to examine whether our association results were robust. We randomly divided the 379,506 European samples into 10 subsets. One subset was used as a replication set, and the remaining nine subsets were used as a discovery set, creating 10 pairs of discovery and replication sets. For the 15 lead SNPs, we conducted an association test in a discovery set and a replication set and performed a meta-analysis of the results from the discovery set and replication set. We tested if the lead SNPs were significant (*P* < 5 × 10^−8^) in the meta-analysis and repeated this test 10 times.

### Gene mapping, gene set analysis, SNP-based heritability, and cell type-specific analyses

The functional mapping and annotation (FUMA) platform was used to examine functional annotations and evidence of expression quantitative trait loci (eQTL) and to conduct pathway analyses^23^. SNP annotation was performed using the ANNOVAR package implemented in FUMA^24^. The eQTL analyses based on the Genotype-Tissue Expression (GTEx) database (https://www.gtexportal.org/home/datasets; v8) and PsychENCODE^25^ database (http://resource.psychencode.org) were performed to identify the genes that were significantly associated with lead SNPs^26^. Statistically significant eQTL associations were identified at a false discovery rate (FDR) < 0.05. The enrichment of gene sets was examined using the MAGMA package implemented in FUMA to analyze the biological pathways determined by the Gene Ontology Consortium^27^.

We used LDSC to estimate SNP-based heritability for irritability using precomputed European LD scores from the 1000 Genomes Project v3 (https://github.com/bulik/ldsc). Variants in the major histocompatibility complex region were excluded, and common autosomal variants with a MAF > 1% in the European population were included. We performed partitioned heritability analyses using LDSC to evaluate the enrichment of 53 genomic annotations within the full baseline model^28^.

We used LDSC applied to specifically expressed genes (LDSC-SEG) to identify enrichments in tissue-specific gene expression and chromatin modification using gene sets obtained from the LDSC website (https://github.com/bulik/ldsc)^29^. We used several gene sets for cell type-specific analyses, including multitissue gene expression (including both GTEx^30^ and Franke lab^31,32^ data) and multitissue chromatin (including both Roadmap Epigenomics^33^ and ENCODE^34^ data), as well as gene sets previously described by Finucane et al. (2018)^29^ and Cahoy et al. (2008)^35^.

### Genetic correlation

Cross-trait genetic correlations (*r*_g_) between irritability and other phenotypes were examined using LDSC^36^ with the default options: imputation quality scores > 0.9 and MAF values > 1%. We used European GWAS summary statistics for 90 phenotypes (these can be downloaded publicly). In addition, European GWAS summary statistics for nine psychiatric disorders were used to analyze shared genetic backgrounds with respect to irritability. Among the well-studied GWASs, we selected those that focused on psychiatric disorders from which we could obtain European samples with the least overlap with the UK Biobank^37^. These psychiatric disorders were classified into three groups: mood and psychotic disorders (SCZ, BD I, BD II, MDD), early-onset neurodevelopmental disorders (autism spectrum disorder, ADHD, Tourette’s syndrome), and disorders with compulsive behaviors (obsessive-compulsive disorder, anorexia nervosa)^37^. Tourette’s syndrome was classified as both an early-onset neurodevelopmental disorder and a disorder with obsessive behaviors in a prior study that used genomic structural equation modeling. We classified this disorder as an early-onset neurodevelopmental disorder in the present study based on the factor loading value in this previous study.

For this analysis, we applied the FDR correction for multiple comparisons. Additionally, structural magnetic resonance imaging (MRI) of the brain region of interest (ROI) as well as diffusion tensor imaging (DTI) was used to analyze the genetic correlations between irritability and neuroimaging traits (Supplementary Table 9).

The volumes of the ROIs in the brain were mapped using the ROI atlas^38^, and DTI was performed using the brain DTI atlas for brain annotation^39^. BrainNet Viewer 1.7 was used to visualize the brain regions; *r*_g_ values ranged from −1 to 1^40^. The MNI-ICBM-152 template^41^ in MATLAB R2020a (Mathworks, Inc., Natick, MA, USA) was used to normalize interpolations and color scales. The UK Biobank Brain Imaging Documentation provides a detailed description of brain volume and DTI measures (https://biobank.ctsu.ox.ac.uk/crystal/crystal/docs/brain_mri.pdf). We applied FDR correction for the ROI and for each DTI scalar (FA, MD, AD, RD, MO).

### Polygenic overlap

We used GWAS summary statistics for nine major psychiatric disorders (Supplementary Table 10) to investigate whether irritability had a shared genetic basis with these phenotypes. We applied MiXeR^42^ (http://github.com/precimed/mixer) to quantify polygenic overlap and evaluated genetic correlations. Univariate analyses using MiXeR yield the number of trait-influencing loci for each trait (i.e., polygenicity) and the average magnitude of additive genetic associations among these variants (i.e., discoverability)^43^. Bivariate analysis models were used to determine additive genetic effects as a mixture of four bivariate Gaussian components, representing variants not affecting either trait, variants affecting only one of the traits, and variants affecting both traits^42^. To evaluate polygenic overlap, MiXeR was implemented to calculate the Dice coefficient (i.e., the ratio of shared variants to the total number of variants). Model fit evaluated via the Akaike information criterion (AIC) was based on the maximum likelihood of GWAS z scores and was illustrated with conditional quantile‒quantile (Q-Q) plots.

### CondFDR and conjFDR analyses

The condFDR approach (https://github.com/precimed/pleiofdr) was applied to identify additional loci associated with psychiatric disorders and irritability^44^. This technique was used because combining GWASs for the two associated phenotypes increases statistical power, allowing for the identification of new common variants associated with both phenotypes that were not identified in the original GWASs^44^. Additionally, the conjFDR approach was employed to identify loci shared between irritability and psychiatric disorders^44^. This method assigns the maximum condFDR values of each trait as the conjFDR value between two traits and allows the identification of genomic loci related to both traits^44^. Psychiatric disorders with reasonable polygenic overlap in MiXeR were included in this analysis. We plotted all SNPs within an LD block in relation to their chromosomal location in a conditional/conjunctional Manhattan plot and identified the strongest signal in each LD block by ranking all SNPs based on the conditional/conjunctional FDR value. Independent genomic loci were defined using the FUMA protocol. To calculate how many loci were additionally identified in this study compared to the original GWAS results, we determined if the variants were located within ± 1 Mbp of the variants reported in previous studies. Finally, eQTL mapping was applied to link-defined loci with related genes, and tissue specificity analyses were performed with mapped genes using the GTEx database within the FUMA platform. Overall, MiXeR and cond/conjFDR provided a more comprehensive overview of the genetic relationships between traits, as these methods are not affected by the effect direction.

### Sex-stratified analyses

We conducted sex-stratified GWAS and post-GWAS analyses to examine the genetic architecture of irritability in each sex (females: 52,529 cases and 152,268 controls; males: 53,446 cases and 121,263 controls). These analyses included genome-wide association tests, SNP-based heritability and partitioned heritability analyses, multiple tissue analyses, genetic correlation analysis, and MiXeR analysis.

## Results

### Genetic architecture of irritability

**Fig. 1:**
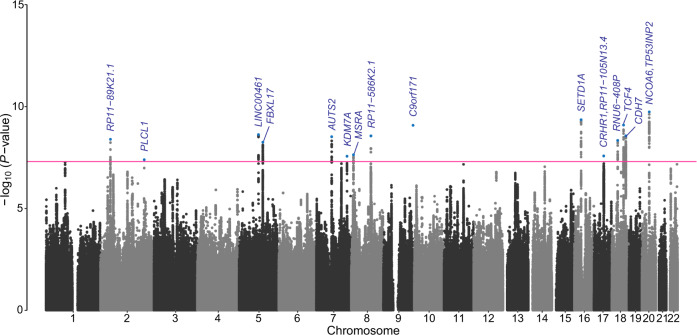
Manhattan plot displaying GWAS results for irritability. The *x*-axis shows the genomic positions, and the *y*-axis shows statistical significance as -log_10_ (*P*) values. The threshold for significance, which was adjusted for multiple comparisons, is shown by the red horizontal line (*P* = 5 × 10^−8^). The blue dots show the nearest genes to the lead SNPs.

A total of 15 lead SNPs with genome-wide statistical significance (*P* < 5 × 10^−8^) were identified (Table 1 and Fig. 1) using 379,506 samples from the UK Biobank (regional plots presented in Supplementary Fig. 1). The Q-Q plot of the GWAS results indicated genomic inflation (λ = 1.121, Supplementary Fig. 2), which was attributable to the polygenicity of irritability (LDSC intercept = 0.9195; standard error = 0.008). In the additional two-stage GWAS analyses that were conducted as a sensitivity analysis, all the lead SNPs were significant (*P* < 5 × 10^−8^) in meta-analyses of 10 randomly assigned discovery and replication sets (Supplementary Table 22).

**Table 1:** Summary of the lead SNPs in the 15 loci associated with irritability.

SNP ID	CHR	BP	A1/A2	EAF	OR	95% CI	Beta	*SE*	*P*	Nearest genes	eQTL genes
rs4953150	2	45157336	T/C	0.341	1.034	(1.023–1.046)	0.034	0.006	4.05 × 10^-9^	*RP11-89K21.1*	
rs7573001	2	198929896	C/G	0.379	0.970	(0.959–0.980)	−0.031	0.006	4.07 × 10^-8^	*PLCL1*	
rs13166120	5	87948883	A/G	0.156	1.045	(1.030–1.061)	0.044	0.007	2.39 × 10^-9^	*LINC00461*	
rs11948261	5	107753691	A/T	0.214	1.039	(1.026–1.053)	0.038	0.007	5.64 × 10^-9^	*FBXL17*	
rs2158507	7	69862423	A/C	0.389	0.968	(0.957–0.978)	−0.033	0.006	3.03 × 10^-9^	*AUTS2*	
rs62491417	7	139835245	A/C	0.065	0.941	(0.920–0.961)	−0.061	0.011	2.75 × 10^-8^	*KDM7A*	*KDM7A*
rs2952176	8	10143553	G/A	0.264	1.035	(1.022–1.047)	0.034	0.006	2.32 × 10^-8^	*MSRA*	
rs16884419	8	89579649	A/G	0.236	1.039	(1.026–1.052)	0.038	0.006	2.77 × 10^-9^	*RP11-586K2.1*	
rs999483	9	135301389	G/T	0.250	1.039	(1.026–1.052)	0.038	0.006	8.33 × 10^-10^	*C9orf171*	
rs2054213	16	30971810	A/G	0.374	0.966	(0.955–0.976)	−0.035	0.006	4.44 × 10^-10^	*SETD1A*	*C16orf93, HSD3B7, KAT8, PRSS36* *SETD1A, STX1B, STX4, VKORC1*, *TRIM72, INO80E, RNF40, ZNF668*
rs78454137	17	43785096	A/G	0.221	1.037	(1.024–1.050)	0.036	0.007	2.66 × 10^-8^	*CRHR1*, *RP11-105N13.4*	*ARHGAP27, ARL17A, CRHR1, FMNL1, KANSL1, LRRC37A, LRRC37A2, MAPT, NMT1, NSF, PLEKHM1, SPPL2C*
rs1941879	18	26604317	A/G	0.066	1.066	(1.043–1.089)	0.064	0.011	4.63 × 10^-9^	*RNU6-408P*	
rs10503002	18	53109202	T/C	0.197	1.043	(1.029–1.057)	0.042	0.007	8.12 × 10^-10^	*TCF4*	*TCF4*
rs2628207	18	63546386	C/T	0.294	1.036	(1.024–1.048)	0.035	0.006	2.75 × 10^-9^	*CDH7*	
rs13037664	20	33296988	T/C	0.191	1.045	(1.031–1.059)	0.044	0.007	1.83 × 10^-10^	*NCOA6, TP53INP2*	*NCOA6, EDEM2, GSS*

*SNP* Single nucleotide polymorphism, *CHR* Chromosome, *BP* Genomic position in human genome assembly GRCh37 (hg19), *A1* Effect allele, *A2* Noneffect allele, *EAF* Effect allele frequency, *OR* Odds ratio, *CI* Confidence interval, *Beta* Regression coefficient, *SE* Standard error.

The eQTL analysis identified 29 cis-eQTL genes mapped to the lead SNPs (Supplementary Table 2); five lead SNPs (rs62491417, rs2054213, rs78454137, rs10503002 and rs13037664) were detected (Table 1). The eQTL genes and genes located in the 15 GWAS loci were significantly enriched in three gene sets (neurogenesis, stress-induced premature senescence, and neuron differentiation) in the FUMA gene set enrichment test (Supplementary Table 3).

**Fig. 2:**
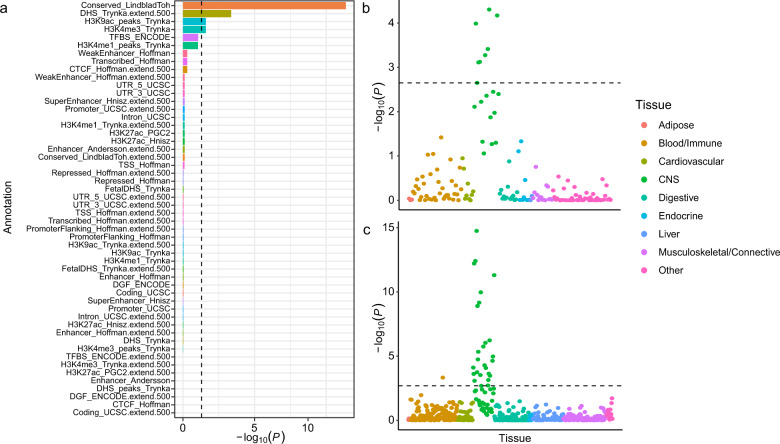
Partitioned heritability analyses using linkage disequilibrium score regression. **a** Enrichment estimates for 53 functional annotations. Annotations are ordered by their *P* values. The dashed line indicates significance at a false discovery rate (FDR) < 5%. **b** The results of multiple-tissue analysis using gene expression data. Each circle represents a tissue or cell type from either the Genotype-Tissue Expression (GTEx) dataset or Franke lab dataset. The dashed line indicates the FDR cutoff, which was < 5% at -log_10_ (*P*) = 2.65. **c** The results of multiple-tissue analysis using chromatin data. Each circle represents a peak for DNase I hypersensitivity site (DHS) or histone marks in a tissue or cell type. The dashed line indicates the FDR cutoff, which was < 5% at -log_10_ (*P*) = 2.69.

The total SNP heritability of irritability was estimated to be 4.19% (standard error = 0.2%). In the partitioned heritability analysis, four of the 53 annotations passed the FDR threshold of 0.05 (Fig. 2a and Supplementary Table 4): the conserved genomic region defined by Lindblad-Toh et al. ^45^, the DNase I hypersensitivity site (DHS), H3K4me3 (indicative of active promoters), and H3K9ac (indicative of active promoters or enhancers).

In the LDSC-SEG analysis, the central nervous system (CNS) showed strong enrichment at an FDR < 5% (Supplementary Table 5). Among the CNS cells, neurons were enriched (Supplementary Table 6). Regarding multitissue gene expression, the cerebral cortex, frontal lobe, and limbic system showed the highest enrichment (Fig. 2b and Supplementary Table 5). In terms of multitissue chromatin results, the highest enrichment was observed in the CNS, including the fetal brain, dorsolateral prefrontal cortex, and germinal matrix regions (Fig. 2c and Supplementary Table 7). Additionally, the LDSC-SEG analysis restricted to only the conserved genomic region defined by Lindblad-Toh et al. showed enrichment in the retinal tissue of the CNS in the gene expression data and enrichment in multiple CNS tissues in the chromatin data (Supplementary Fig. 9).

### Genetic relationships between irritability and other traits

**Fig. 3:**
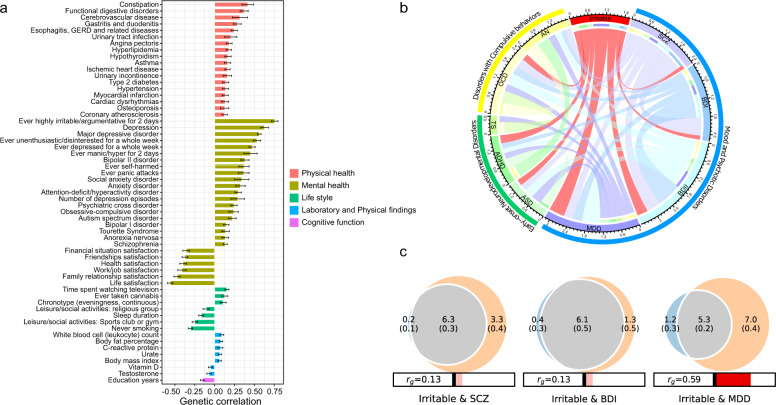
Genetic relationships (genetic correlation estimates and polygenic overlap) between irritability and other traits. **a** This figure displays only significant genetic correlations (false discovery rate (FDR)-corrected *P*-values < 0.05). See Supplementary Table 8 for all results. GERD Gastroesophageal reflux disease. **b** Genetic correlation estimates between irritability and psychiatric disorders. This figure includes only significant genetic correlations (Bonferroni-corrected *P*-values < 0.05). See Supplementary Table 11 for all results. The thickness of the links between two traits indicates the strength of their estimated genetic correlation. The circumference of each trait represents the sum of genetic correlations with other traits. ADHD Attention deficit/hyperactivity disorder, AN Anorexia nervosa, ASD Autism spectrum disorder, BD I Bipolar I disorder, BD II Bipolar II disorder, MDD Major depressive disorder, OCD Obsessive-compulsive disorder, SCZ Schizophrenia, TS Tourette’s syndrome. **c** Venn diagrams depicting the estimated number of shared (gray) trait-influencing variants between irritability (left circle) and psychiatric disorders (right circle; schizophrenia, bipolar I disorder, and major depressive disorder) and the numbers of unique variants (blue and orange) (see Supplementary Fig. 4 for all results). The number of trait-influencing variants (in thousands) is shown, with the standard error (in thousands) given in parentheses. The estimated genetic correlation for each pair is also shown below the corresponding Venn diagram, with an accompanying directional scale (blue shades for negative values and red shades for positive values).

We performed LDSC on the summary statistics for health-associated traits to compute genetic correlations (*r*_g_) (Fig. 3a and Supplementary Table 8). A total of 59 mental and physical health-associated traits showed significant genetic correlations with irritability. Statistically significant positive correlations were observed between irritability and constipation (*r*_g_ = 0.42), functional digestive disorders (*r*_g_ = 0.37), MDD (*r*_g_ = 0.56), and positive answers to “ever depressed for a whole week” (*r*_g_ = 0.47) and “ever unenthusiastic/disinterested for a whole week” (*r*_g_ = 0.53). Statistically significant negative correlations were observed with satisfaction-related traits (*r*_g_ range = −0.56 to −0.36), never smoking (*r*_g_ = −0.30), and leisure/social activities (*r*_g_ = −0.25 for sports club or gym, *r*_g_ = −0.11 for religious group). These correlations suggest common genetic associations between irritability and multiple health and lifestyle factors.

LDSC analysis was likewise conducted to test the genetic correlations of irritability with brain region volumes and connectivity via structural MRI and DTI, respectively (Supplementary Fig. 3 and Supplementary Table 9). The volumes of several brain regions were correlated with irritability, but no statistically significant correlations were observed after FDR correction. With respect to DTI traits, the fractional anisotropy of the fornix and stria terminalis showed a statistically significant genetic correlation (*r*_g_ = −0.17) at an FDR < 5%.

Genetic correlations between irritability and nine major psychiatric disorders are presented in Supplementary Tables 10 and 11. Statistically significant estimates (after Bonferroni correction) are shown in Fig. 3b. Irritability was strongly correlated with MDD (*r*_g_ = 0.56) and BD II (*r*_g_ = 0.38) in the category of mood and psychotic disorders.

We obtained MiXeR estimates for polygenic overlap between irritability and psychiatric disorders (Supplementary Fig. 4, Supplementary Tables 12, 13). In addition to demonstrating the highest *r*_g_ estimate of the LDSC analyses, MDD showed considerable polygenic overlap (Dice coefficient = 0.56), sharing approximately 5300 variants with irritability (Fig. 3c). Furthermore, MiXeR analysis showed that SCZ (Dice coefficient = 0.78) and BD I (Dice coefficient = 0.88) had the largest amount of genetic overlap with adequate model fit. Despite the weak genetic correlations observed in LDSC analysis, SCZ and BD I shared approximately 6300 and 6100 variants with irritability, respectively, indicating that nearly all SNPs affecting irritability also influenced SCZ and BD I (Fig. 3c). Moreover, although irritability showed a moderate genetic correlation with BD II, MiXeR was unable to quantify the polygenic overlap between these two traits, as the negative value of the Akaike information criterion indicated poor model fit.

### Additional and shared loci of psychiatric disorders and functional annotation

**Fig. 4:**
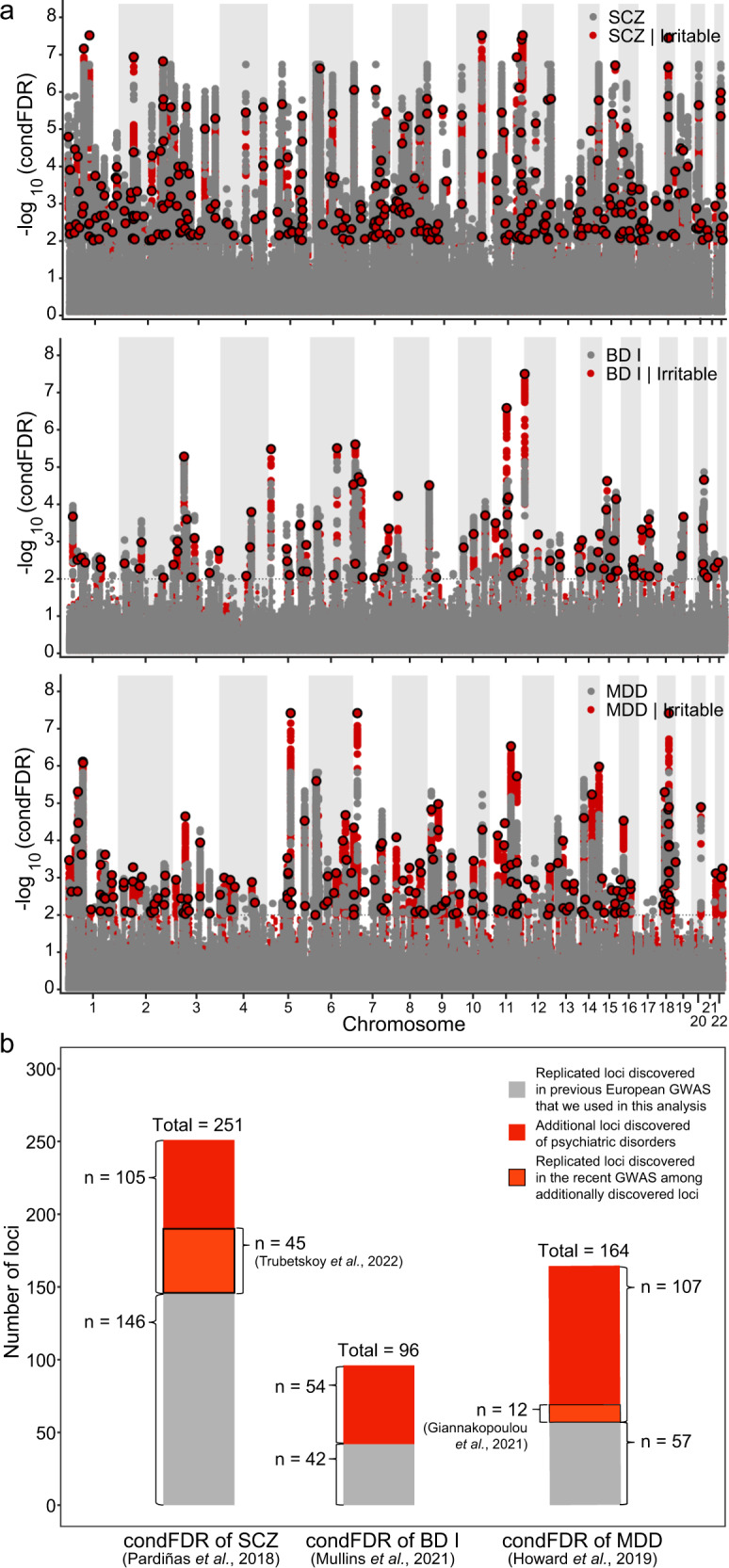
Conditional false discovery rate analyses of irritability and psychiatric disorders (schizophrenia, bipolar I disorder and major depressive disorder). **a** Conditional Manhattan plot (with a -log_10_ scale) of conditional false discovery rate (condFDR) values for psychiatric disorders alone (gray) and psychiatric disorders given irritability (red). SNPs with -log_10_ (condFDR) > 2 (i.e., FDR < 0.01) are shown with large dots. A black line around the dots indicates the most significant SNP in each linkage disequilibrium block. **b** Comparison of newly-identified significant condFDR variants compared to previously reported variants. A total of 105 variants for SCZ, 54 variants for BD I, and 107 variants for MDD were additionally identified (see Supplementary Tables 14–16). Among the 105 SCZ variants, 45 were replicated in the latest GWAS by Trubetskoy et al. (2022)^49^. Among the 107 MDD variants, 12 were replicated in the GWAS by Giannakopoulou et al. (2021)^50^.

For condFDR analyses of SCZ, BD I, and MDD (using irritability as an associated phenotype), we employed a conditional Manhattan plot to visualize the localization of genetic markers (Fig. 4a). We identified 251 SCZ-associated genomic loci based on their associations with irritability. Of these loci, 105 were additionally significant when compared with a previous GWAS included in this analysis^46^. In the condFDR analysis for BD I and MDD, we identified 96 BD I-associated genomic loci and 164 MDD-associated genomic loci, of which 54 and 107, respectively, were new compared to those reported in prior studies^47,48^. (Fig. 4b; Supplementary Tables 14−16). Moreover, we compared our results with the SCZ GWAS findings obtained by Trubetskoy et al. in 2022^49^ to determine how many of the 105 additional loci associated with SCZ were replicated in reference to the most recent and largest cross-ancestry SCZ GWAS to date. Among the 105 additional loci, 45 were statistically significant at the genome-wide level, and 60 were unique to this study. Of the 107 additional loci associated with MDD, 12 were replicated in a recent cross-ancestry GWAS by Giannakopoulou et al. (2021)^50^, while 95 loci remained unique.

We mapped the additional loci (105 for SCZ, 54 for BD I, and 107 for MDD) to genes via eQTL analysis and identified 117, 45, and 90 genes, respectively (Supplementary Table 17). The protein-coding gene mitochondrial ribosomal protein S33 (*MRPS33)* was detected in all three disorders. Furthermore, 117 genes mapped with additional loci for SCZ were enriched in five tissue types, 45 genes mapped with BD I loci were enriched in seven tissue types, and 90 genes mapped with MDD loci were enriched in eight tissue types, with enrichment in the brain demonstrated in all three disorders (Supplementary Fig. 5).

For condFDR analyses of irritability (using SCZ, BD I, and MDD as associated phenotypes) and conjFDR analyses between irritability and psychiatric disorders, we also generated a conditional/conjunctional Manhattan plot to visualize the localization of the genetic markers (Supplementary Fig. 6). Using SCZ, BD I, and MDD as associated phenotypes for condFDR analyses, we identified 69, 54, and 116 irritability-associated genomic loci (Supplementary Table 18), which were mapped to 136, 48, and 151 genes, respectively, via eQTL analysis (Supplementary Table 19). As 157 loci were additionally significant in comparison to our GWAS results, leveraging the phenotype of psychiatric disorders greatly improved the identification of SNPs associated with irritability. The conjFDR analyses indicated 85, 41, and 198 shared genetic loci between irritability and psychiatric disorders (SCZ, BD I, and MDD, respectively) (Supplementary Table 20), which were mapped to 142, 45, and 153 genes (Supplementary Table 21). In all cases, except for condFDR analysis of irritability associated with BD I, we detected significantly enriched brain expression (Supplementary Figs. 5 and 7).

### Sex-stratified analysis

We performed sex-stratified analyses to enhance our understanding of the genetic architecture of irritability in males and females. Due to the reduced statistical power, the sex-specific GWAS identified fewer significant loci than the overall GWAS (Supplementary Fig. 8a–d). Nevertheless, one additional locus (rs12675694) was identified in the female-specific GWAS that was not found in the overall GWAS. In addition, we also conducted post-GWAS analyses in each subgroup. The SNP-based heritability estimates in female and male samples were 4.94% and 5.56%, respectively, and both were slightly higher than that in the overall sample (4.19%). The genetic correlation between female and male samples was 1.04 (standard error = 0.05). Similarly, most functional analysis results were consistent between sex-stratified and overall analyses and between male and female subgroup analyses. The SNP heritability in both sexes was strongly enriched in the conserved or regulatory genomic regions and the CNS (Supplementary Fig. 8e–j). The genetic correlation analysis also revealed similar patterns in males and females (Supplementary Fig. 8k–n). Consistent with the overall analysis, SCZ and BD I showed the largest shared polygenicity with irritability (Supplementary Fig. 8o–t).

## Discussion

In the current study, we performed comprehensive genomic analyses to determine the genomic basis of irritability and its relationship with psychiatric disorders. Our GWAS identified 15 lead variants associated with irritability in the UK Biobank participants of European ancestry. Sensitivity analysis via a two-stage GWAS supported the robustness of our association results. The estimated SNP-based heritability of irritability was 4.19% (Fig. 1 and Table 1). Using eQTL and LDSC-SEG analyses, we determined genes associated with the identified SNPs in brain tissues and found that partitioned heritability was enriched in the CNS and neurons (Fig. 2b, c, Supplementary Tables 5, 6). LDSC analysis revealed statistically significant genetic correlations between irritability and multiple health-associated traits, with positive correlations with physical and psychiatric disorders/symptoms and negative correlations with beneficial lifestyle factors (Fig. 3a). MiXeR analyses revealed statistically significant polygenic overlaps between irritability and SCZ, BD I, and MDD (Fig. 3c). Using condFDR, we identified additional SNPs associated with these psychiatric disorders by leveraging the reduced FDR obtained via the associated trait of irritability (Fig. 4, Supplementary Tables 14, 15, and 16). These analyses also helped to identify 157 additional SNPs associated with irritability (Supplementary Table 18). The conjFDR analyses revealed a significant number of loci shared between irritability and the following three psychiatric disorders: MDD, BD I, and SCZ (Supplementary Table 20). Moreover, the sex-stratified GWAS identified an additional locus associated with irritability in females despite its reduced statistical power. Most of the sex-stratified functional analyses yielded results consistent with the overall GWAS and specific sex subgroups, suggesting a shared genetic architecture of irritability between males and females.

Our identified loci associated with irritability are in line with those of previous studies, including twin studies, which have indicated that irritability is heritable^11,12^. Using eQTL analysis, we identified 29 cis-eQTL genes that were mapped to the following five lead SNPs: rs62491417, rs2054213, rs78454137, rs10503002 and rs13037664 (Table 1). The rs62491417 locus is in an intron of *KDM7A*, which is involved in brain development^51^. *SETD1A*, which is located near the rs2054213 locus, is associated with feelings of worry, tension, and irritability as well as with SCZ, BD I, and neurodevelopmental disorders^52–54^. *CRHR1*, at the rs78454137 locus, is associated with interpersonal sensitivity, frequent mood swings, and depression^52^. Moreover, *TCF4*, at the rs10501002 locus, is associated with measurements of worry^55^, and *NCOA6*, at the rs13037664 locus, is associated with feeling nervous^52^. Thus, these five lead SNPs may be involved with brain development, emotion regulation, and mood and psychotic disorders, which is consistent with our findings.

Partitioned heritability analysis revealed four statistically significant genomic annotations: the conserved genomic regions defined by Lindblad-Toh et al. ^45^, the DHS (i.e., transcriptionally active genomic regions), H3K4me3 (i.e., active promoters), and H3K9ac (i.e., active promoters or enhancers). Approximately 2.6% of the total SNP heritability was attributed to the conserved regions, representing the largest proportion of enrichment in any category. The importance and significance of the conserved regions have been reported in prior studies on intelligence, physical illnesses and traits, SCZ, and BD^28,56^. H3K4me3 and H3K9ac annotations were similarly enriched in SCZ, BD, and MDD, while the DHS was associated with physical and emotional functioning^28,57,58^. The LDSC-SEG analysis suggested that the CNS was strongly enriched, indicating its significant involvement in the biological basis of irritability (Supplementary Table 5). In terms of cell types, neurons were found to be enriched to a greater extent than oligodendrocytes and astrocytes (Supplementary Table 6). Multitissue gene expression analyses demonstrated that the cerebral cortex, frontal lobe, and limbic system had the highest enrichment (Fig. 2b and Supplementary Table 5). These findings, which align with prior genetic findings^59,60^, suggest that irritability is strongly associated with the biological functions of the brain.

The current findings provide new insights into the genetic relationship between irritability and psychiatric disorders. The LDSC analyses revealed genetic correlations with nine major psychiatric disorders (Supplementary Table 11). This suggests that shared genetic factors may underpin the presence of irritability across several psychiatric disorders. In particular, the LDSC analyses indicated that irritability was most strongly correlated with MDD (*r*_g_ = 0.56) and BD II (*r*_g_ = 0.38) in the category of mood and psychotic disorders (Fig. 3b)^3,4^. Moreover, although the genetic correlations between irritability and SCZ and between irritability and BD I were not strongly positive, the MiXeR analysis revealed significant polygenic overlap (Fig. 3c). MDD also showed considerable polygenic overlap with irritability (Fig. 3c). Positive associations between irritability and SCZ, BD I, and MDD have been demonstrated in epidemiological^3,4,61^, longitudinal genetic^11^, genetic liability^6^, and imaging genetic^62^ studies. However, our findings of polygenic associations of irritability with SCZ and BD I imply that some of the genetic variants shared with irritability may have opposing effects, as low genetic correlation but high polygenic overlap indicates that these two traits share a large number of genetic variants with a mixture of opposing and parallel effect directions.

Based on the polygenic associations between irritability and SCZ, BD I, and MDD, we identified additional and shared SNPs associated with the above four phenotypes via the condFDR and conjFDR approaches (Fig. 4 and Supplementary Fig. 6). Compared to the most recent cross-ancestry GWAS^49^, the present study identified 60 unique loci for SCZ, 54 for BD I^47^, and 95 for MDD^50^. In terms of irritability, 157 additional loci were found along with the 15 loci identified in our GWAS by using the three psychiatric disorders as associated phenotypes. As leveraging irritability or the associated psychiatric disorders revealed additional loci, the genetic architecture of irritability, SCZ, BD I, and MDD may have a common genetic basis. Furthermore, using conjFDR analyses, we found 85, 41, and 198 shared genetic loci between irritability and the three psychiatric disorders (Supplementary Table 20), once again suggesting that there are common genetic underpinnings of these phenotypes. Most of these loci demonstrated significant enrichment in corresponding brain regions. The discovery of shared loci, despite low genetic correlations between irritability and SCZ (*r*_g_ = 0.13) and BD I (*r*_g_ = 0.15), indicates that most of these shared variants have different directions of effects on each trait. These polygenic relationships between irritability and psychiatric disorders provide important evidence for subsequent studies to examine and clarify the genetic basis of irritability and the overlap of biological mechanisms with those of psychiatric disorders. As irritability is a symptom of many psychiatric disorders, including MDD (in children and adolescents), BD I/II, generalized anxiety disorder, and oppositional defiant disorder^10^, we expect that investigating the genomics of irritability more comprehensively will help to elucidate the genomics of other associated psychiatric disorders and vice versa.

This study has some limitations. First, we used a single yes-or-no question (“Are you an irritable person?”) to define irritability. The results should be interpreted carefully because exploring irritability with a simple question could only reveal certain aspects of the complex characteristics of human behavior. Numerous previous studies investigating personality traits using large-scale biobank data share this limitation (of relying on simple questionnaires) but have sufficient statistical power for genetic analyses due to the hundreds of thousands of participants; such studies have nonetheless yielded novel genetic discoveries and insights into human behavior^63,64^. For example, the GWASs of risk-taking behavior and mood instability, which were based on a single question, identified and replicated loci associated with the respective traits and revealed a shared genetic basis with other psychiatric traits^63,65,66^. In the future, studies incorporating more comprehensive questionnaires, such as the Temperament Evaluation of Memphis, Pisa, Paris, and San Diego-Auto Questionnaire^2^, may be used to support our findings. Second, although irritability is predicted to be heritable, environmental factors also influence this trait. Thus, our results should not be utilized to predict an individual’s temperament but rather as evidence of the genetic basis of irritability for a comprehensive evaluation of the associated neurobiological and genetic manifestations of this trait. Third, although our analyses identified strong enrichment in multiple brain tissues, we were unable to determine specific brain regions. This may be partly due to the use of limited functional datasets for brain tissues and cell types^25^. Additionally, we observed significant enrichment in neurons but not in oligodendrocytes and astrocytes (Supplementary Table 6). This may explain the observed widespread enrichment in the brain and suggests that single-cell data across brain regions may be needed to identify specific brain regions that are functionally important for irritability in future studies. Fourth, as our study was limited to individuals of European ancestry, future studies should attempt to replicate our findings across diverse populations. Since the clinical presentations of irritability may differ by culture, subsequent GWASs conducted with cohorts of various ancestries, as well as meta-analyses of cross-ancestry data, may identify additional genetic factors and enhance our knowledge of the genetic architecture of irritability, leading to greater understanding of mental disorders associated with this trait.

## Supplementary information

Supplementary Information

## Data Availability

The UK Biobank data are publicly available upon reasonable application (https://www.ukbiobank.ac.uk). The GWAS summary statistics for nine major psychiatric disorders are available from the Psychiatric Genomics Consortium website (https://www.med.unc.edu/pgc/download-results/).
