# Supplementary material for: Leveraging genetic overlap between irritability and psychiatric disorders to identify genetic variants of major psychiatric disorders
Source: Exp Mol Med. 2023 Jun 1;55(6):1193–202. doi: 10.1038/s12276-023-01005-0 (PMC10317967; doi:10.1038/s12276-023-01005-0)
Supplement: Supplementary file 1 — Supplementary Information [file 12276_2023_1005_MOESM1_ESM.pdf]

Supplementary Figures

Supplementary Figure 1. Regional association plots of a GWAS of irritability. Each plot was generated using the LocusZoom standalone software. Plots are listed by chromosome and position.

Supplementary Figure 1a. chr2:45157336

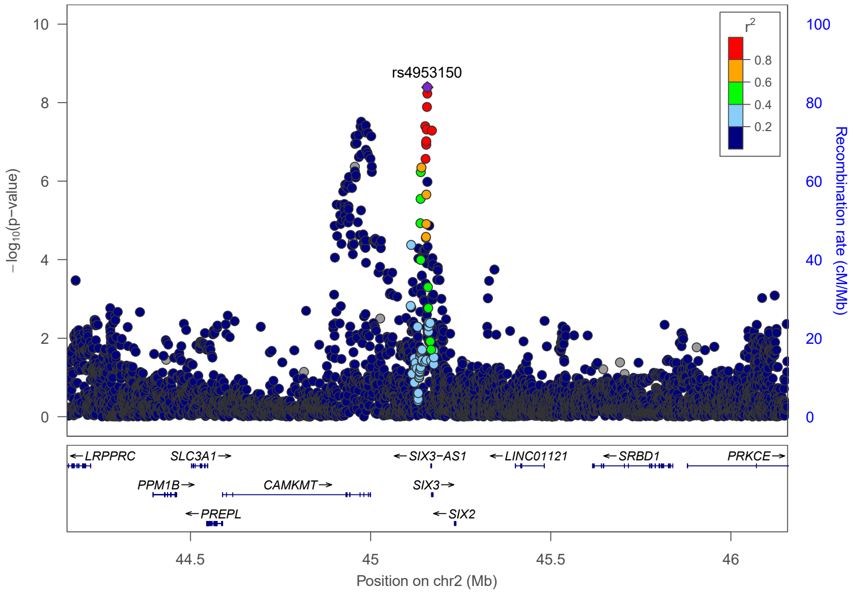

Supplementary Figure 1b. chr2:198929896

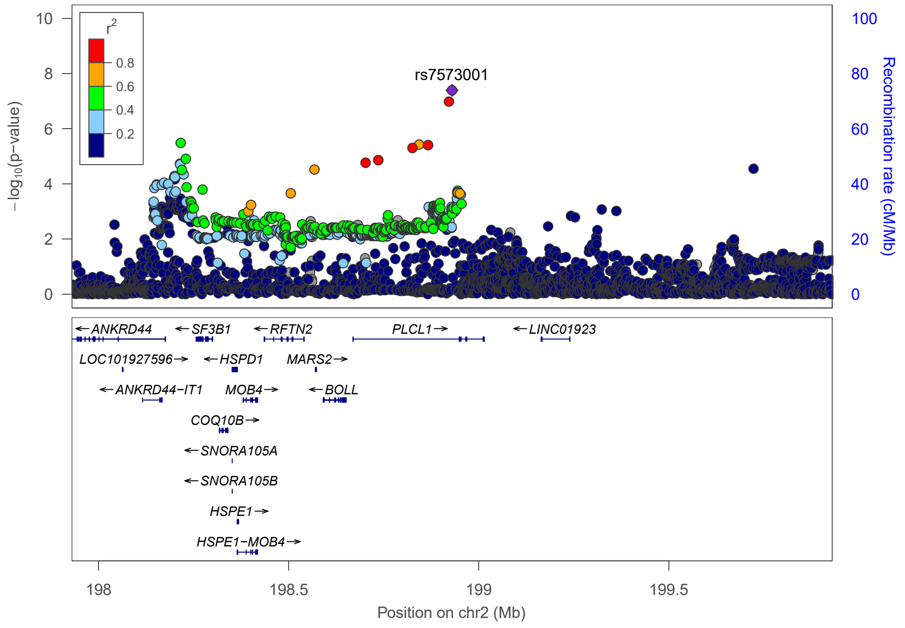

Supplementary Figure 1c. chr5:87948883

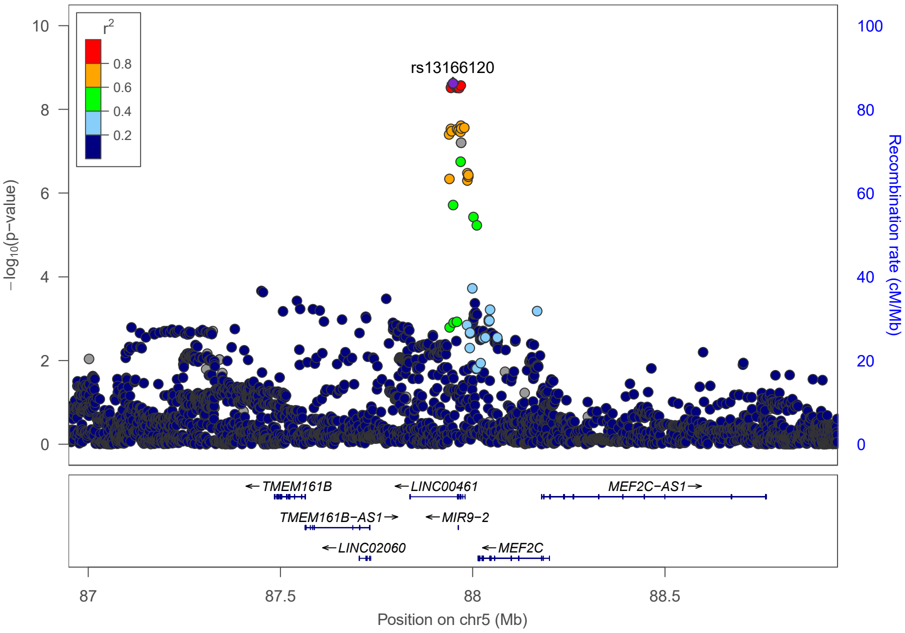

Supplementary Figure 1d. chr5:107753691

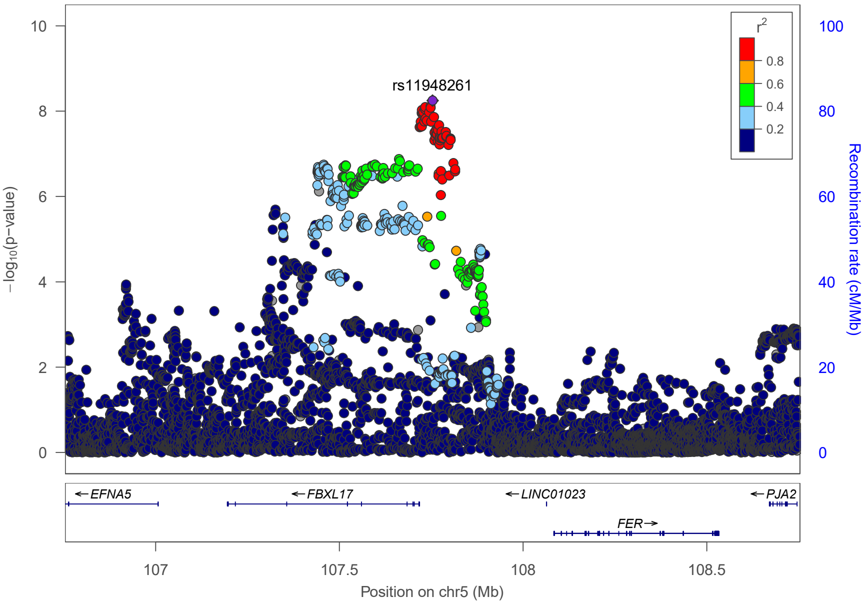

Supplementary Figure 1e. chr7:69862423

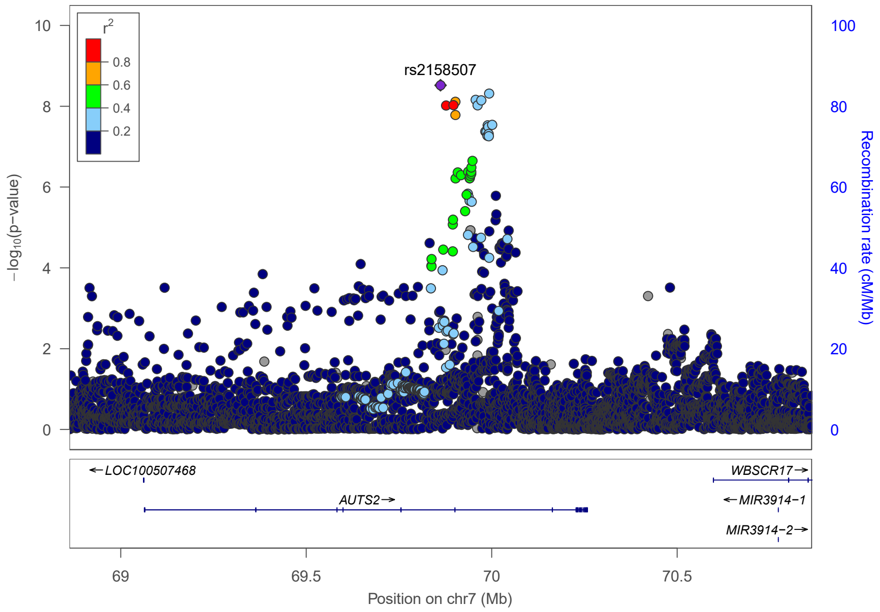

Supplementary Figure 1f. chr7:139835245

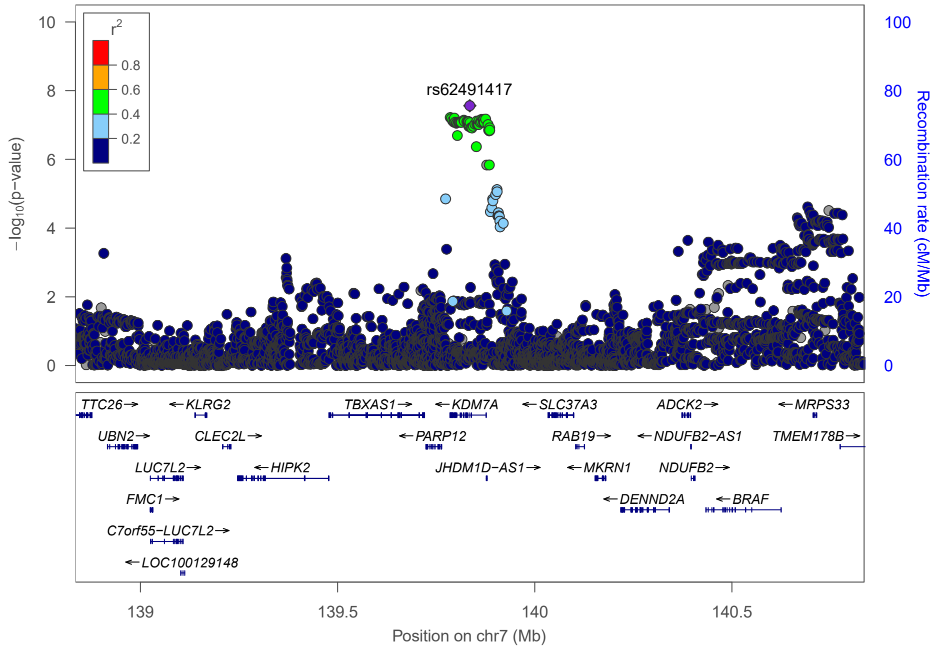

Supplementary Figure 1g. chr8:10143553

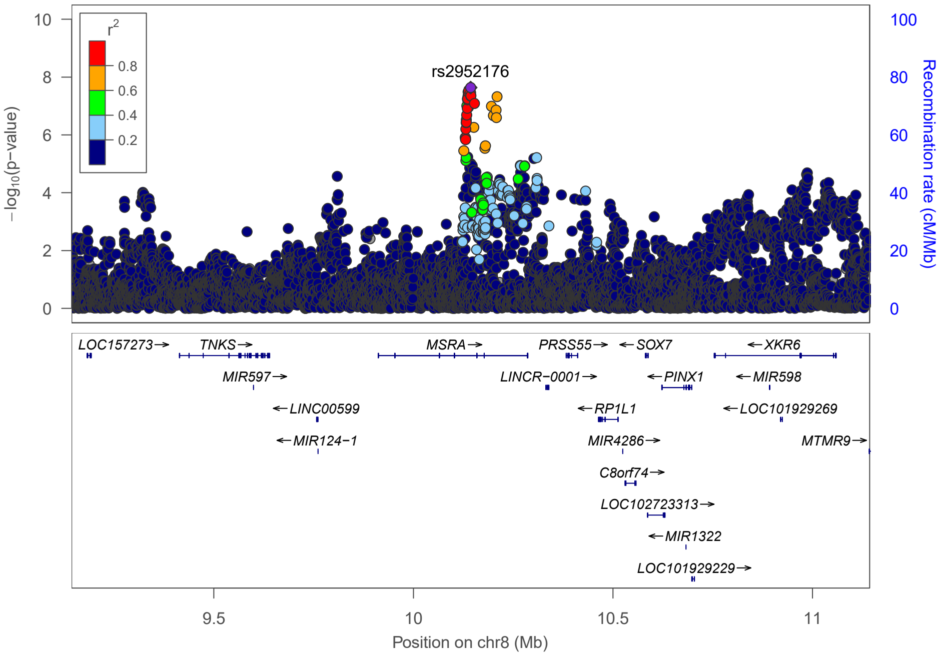

Supplementary Figure 1h. chr8:89579649

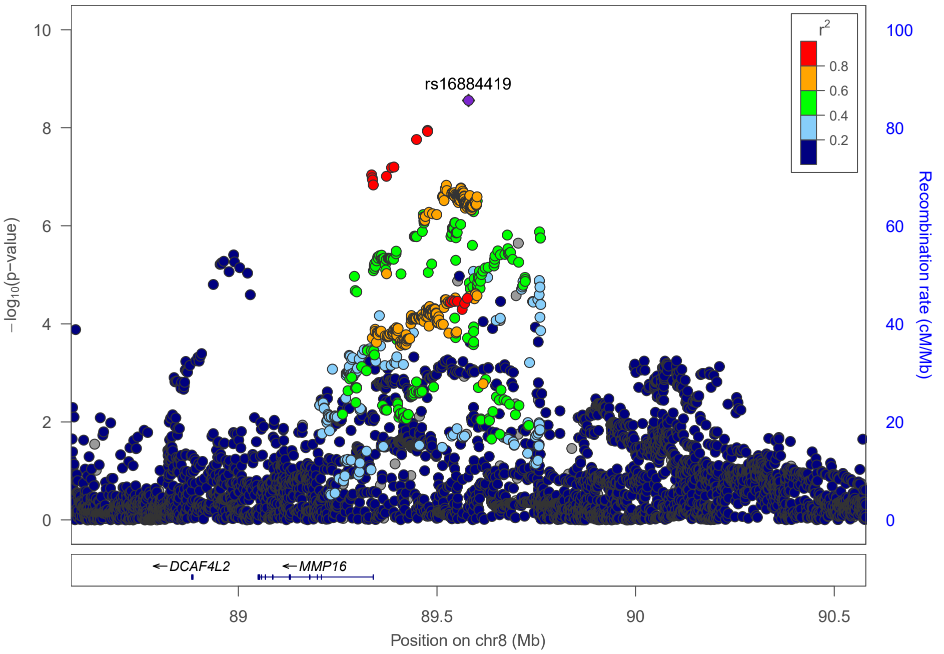

Supplementary Figure 1i. chr9:135301389

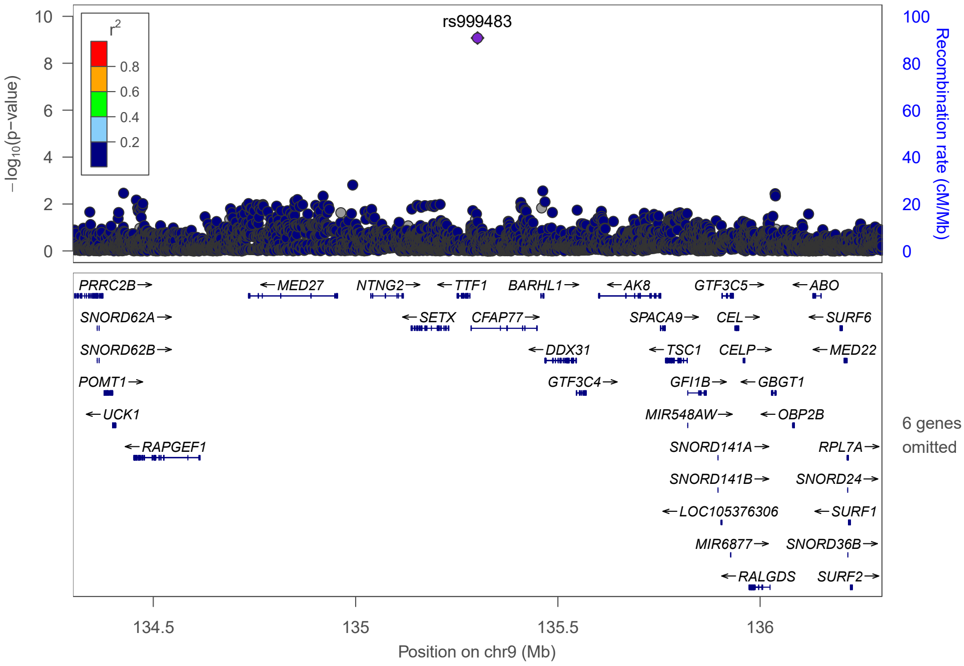

Supplementary Figure 1j. chr16:30971810

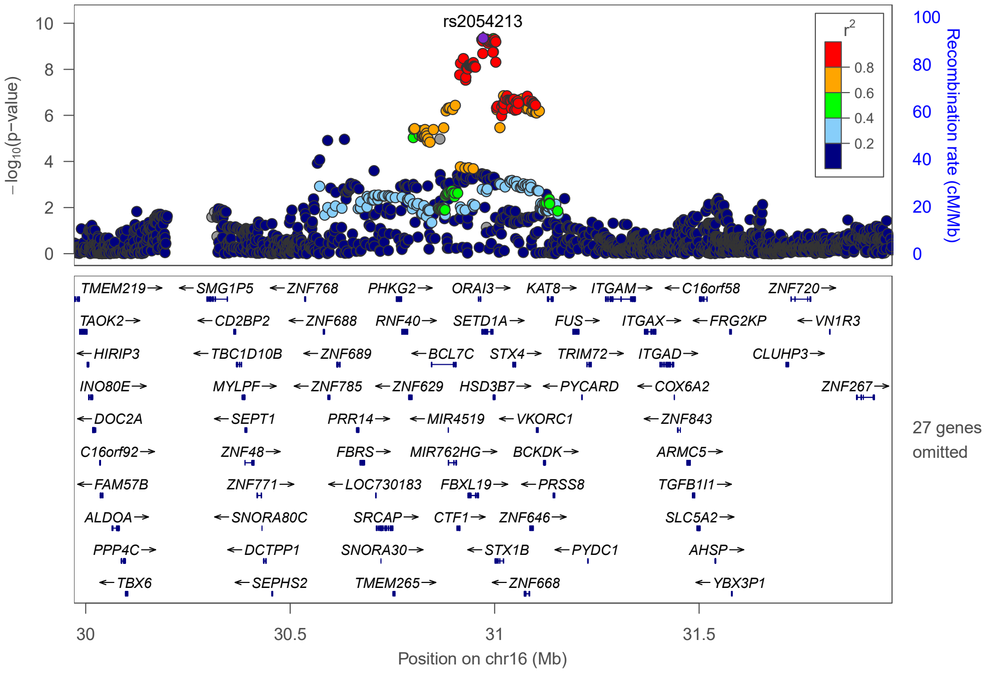

Supplementary Figure 1k. chr17:43785096

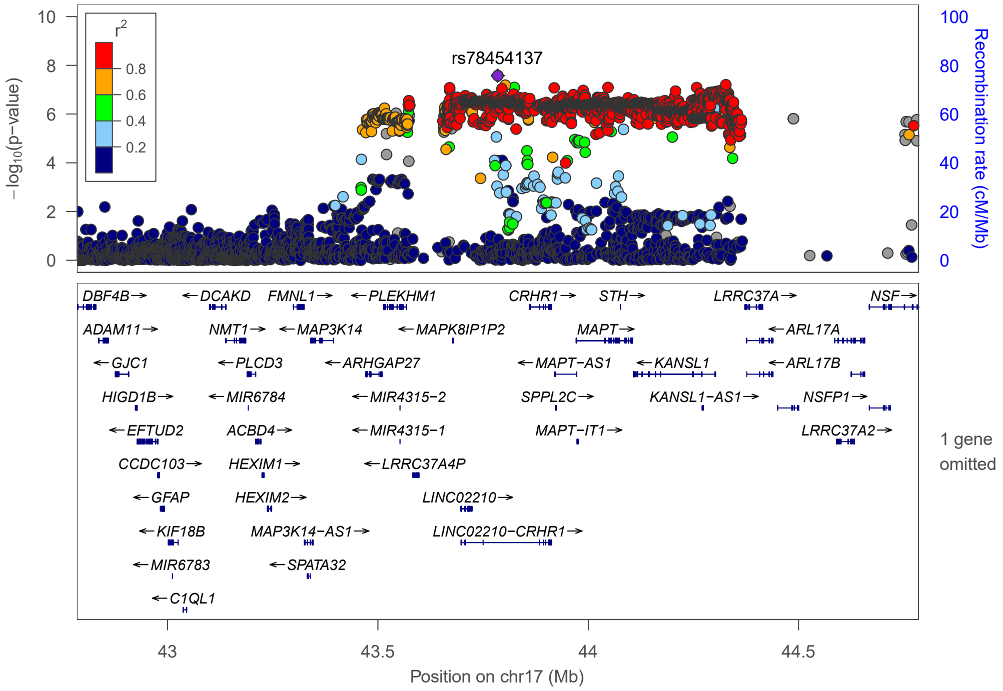

Supplementary Figure 1l. chr18:26604317

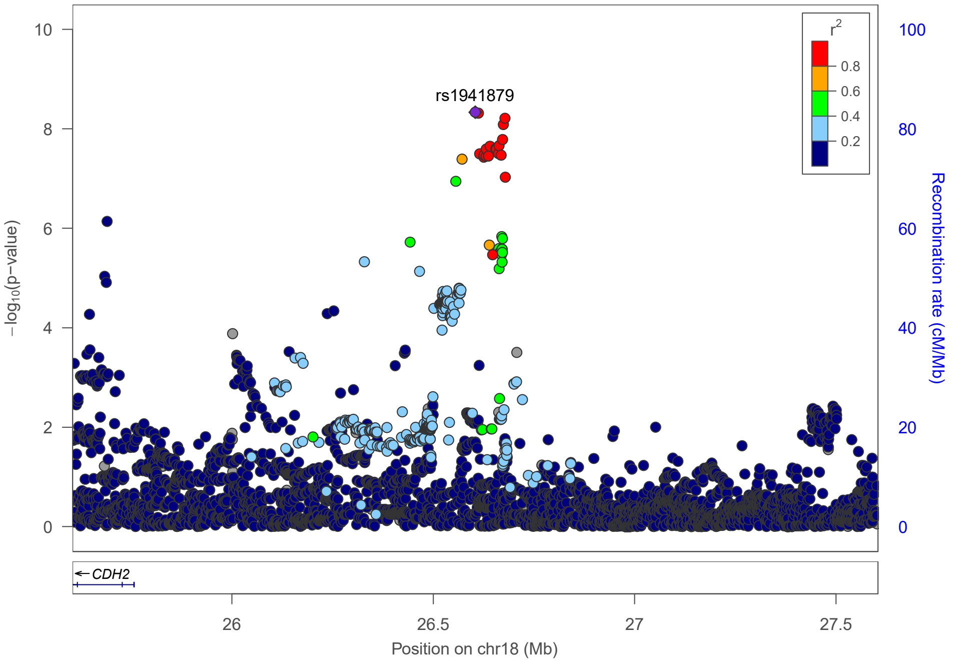

Supplementary Figure 1m. chr18:53109202

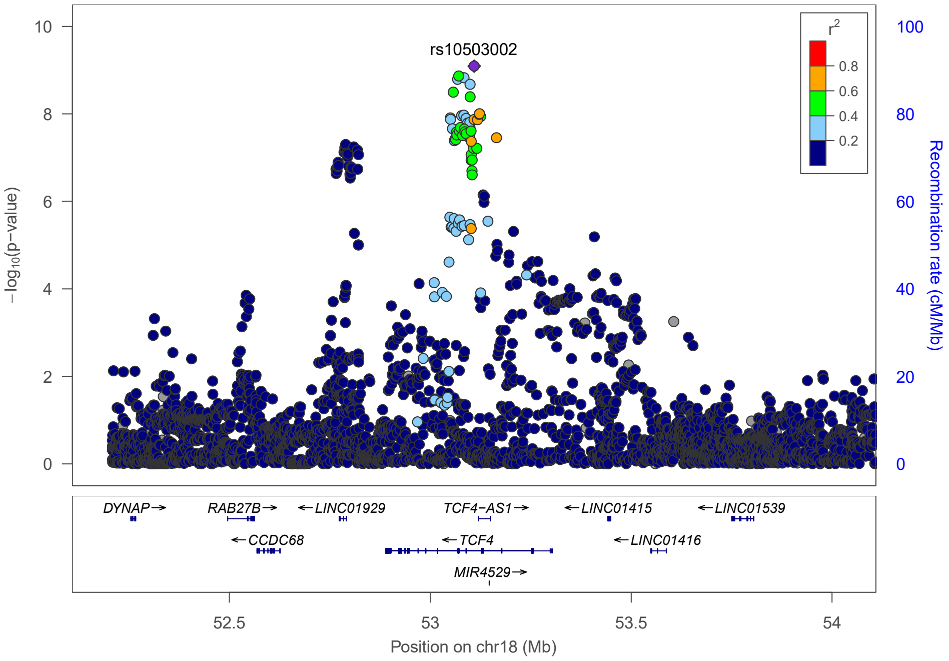

Supplementary Figure 1n. chr18:63546386

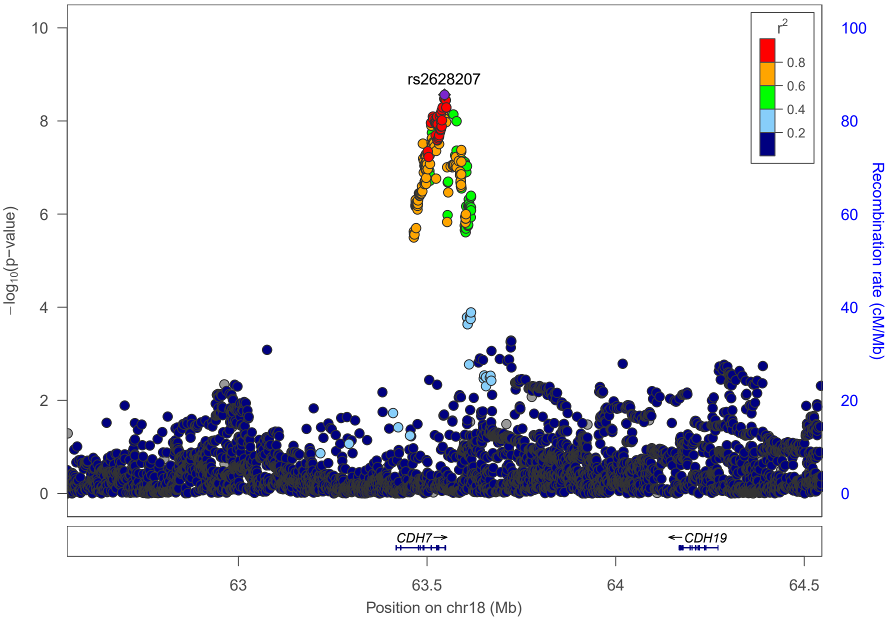

Supplementary Figure 1o. chr20:33296988

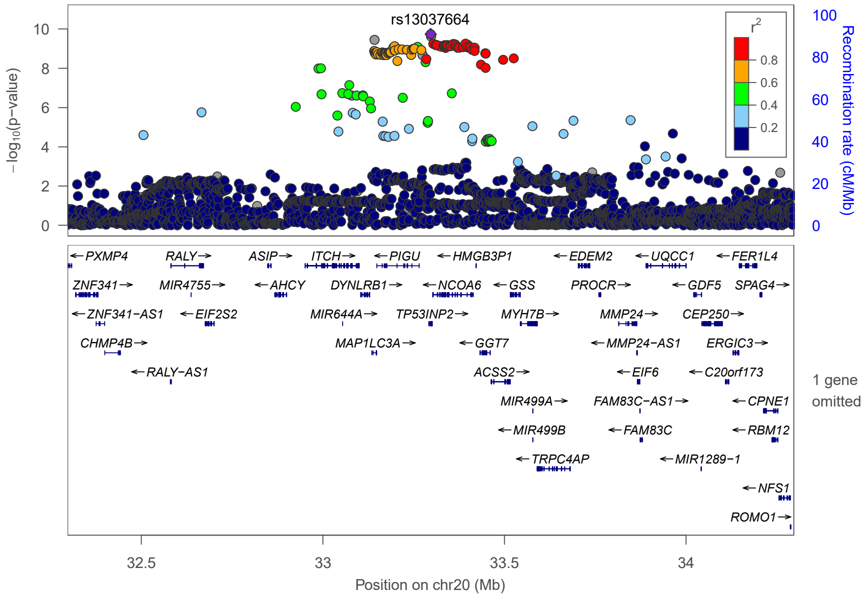

Supplementary Figure 2. Quantile-quantile plot for a GWAS of irritability (n = 379,506). The y-axis shows the observed GWAS P value on a $-\log_{10}$ scale.

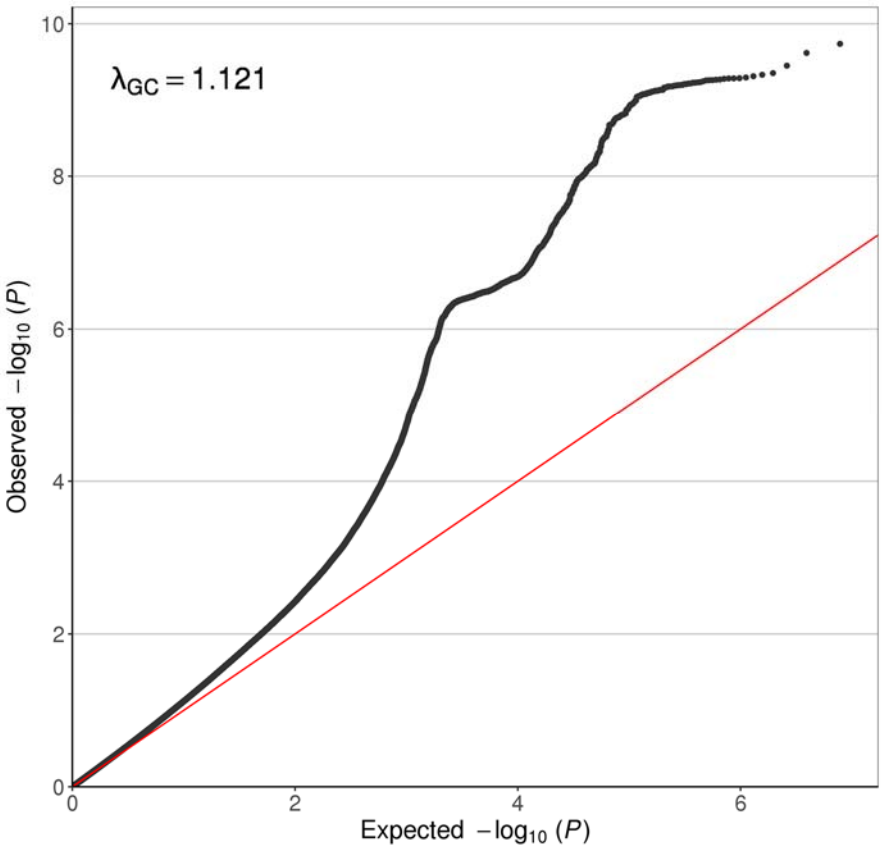

Supplementary Figure 3. Brain regions representing genetic correlations with irritability in LDSC analysis. The blue and red colors indicate negative and positive correlations, respectively. The darker the color, the stronger the correlation.

Supplementary Figure 3a. Brain image showing genetic correlations between irritability and volumes of region of interest (ROI) of the brain at P values < 0.05.

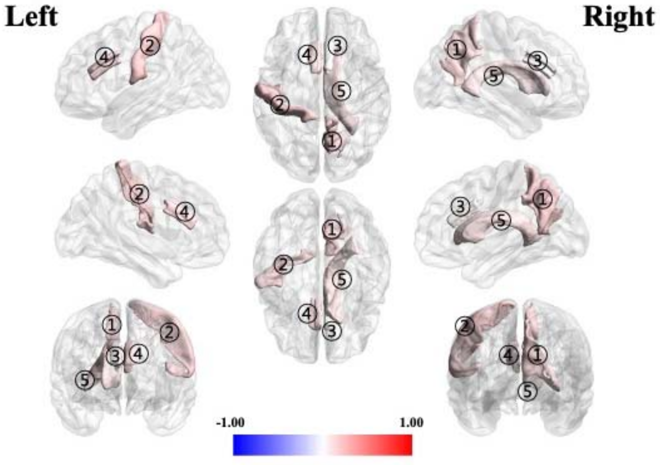

Description	r_g	s.e.	*P*
① right precuneus	0.1622	0.0541	0.0027
② left postcentral	0.1855	0.0628	0.0031
③ left caudal anterior cingulate	0.1281	0.0533	0.0162
④ right caudal anterior cingulate	0.2139	0.0905	0.0181
⑤ right lateral ventricle	0.1043	0.0476	0.0285
Total brain volume	-0.0877	0.0441	0.0467

Supplementary Figure 3b. Brain image showing genetic correlations between irritability and performed diffusion tensor imaging (DTI) of the brain at FDR value < 5%.

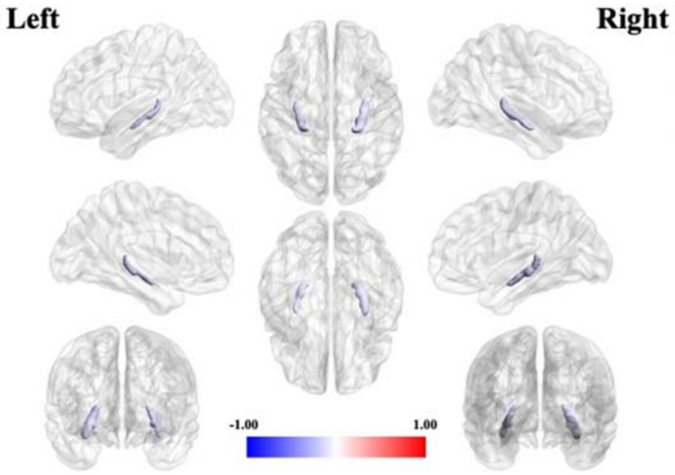

Description	r_g	s.e.	*P*
① FXST.FA	-0.17	0.0536	0.0016

Supplementary Figure 3c. Brain image showing genetic correlations between irritability and performed diffusion tensor imaging (DTI) of the brain at P value < 0.05.

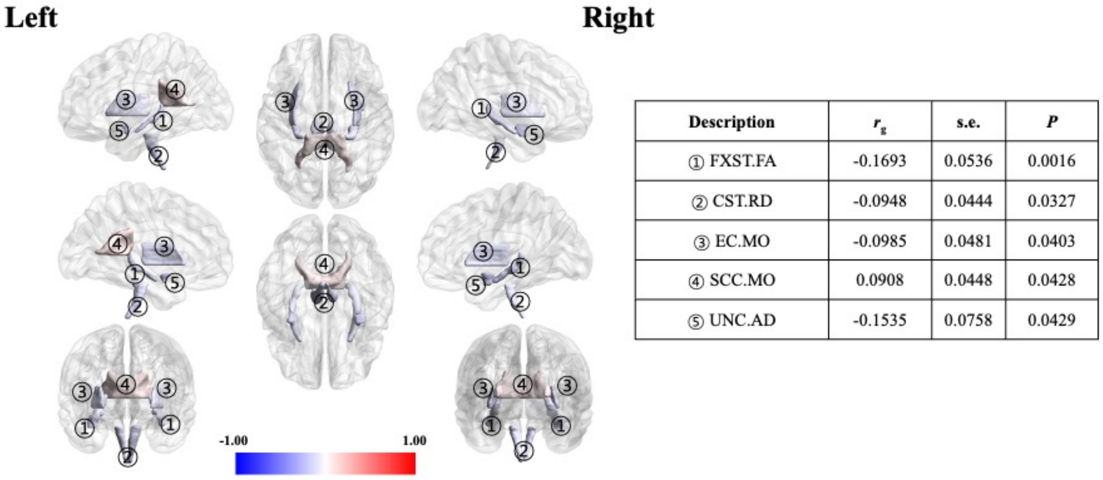

Description	r_g	s.e.	*P*
① FXST.FA	-0.1693	0.0536	0.0016
② CST.RD	-0.0948	0.0444	0.0327
③ EC.MO	-0.0985	0.0481	0.0403
④ SCC.MO	0.0908	0.0448	0.0428
⑤ UNC.AD	-0.1535	0.0758	0.0429

Supplementary Figure 4. Shared polygenicity underlying irritability and psychiatric disorders.

Supplementary Figure 4a. Schizophrenia

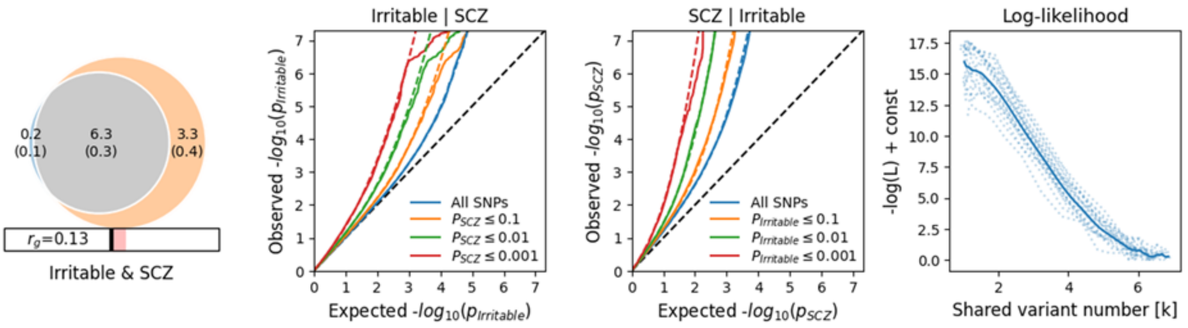

Supplementary Figure 4b. Bipolar I disorder

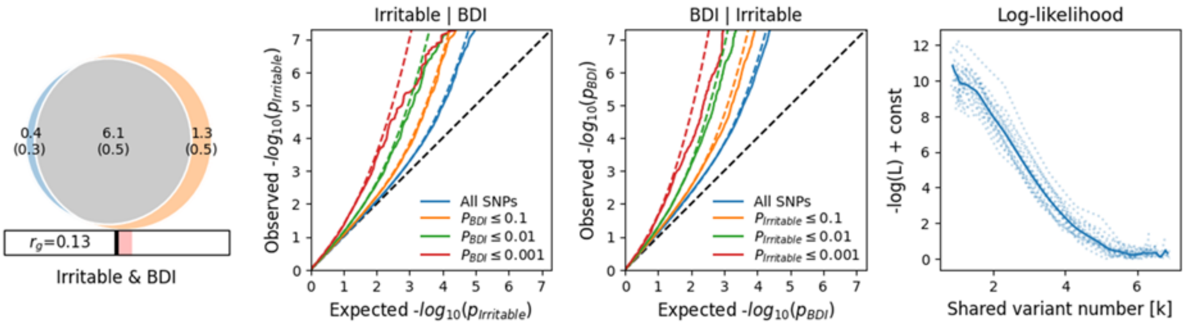

Supplementary Figure 4c. Bipolar II disorder

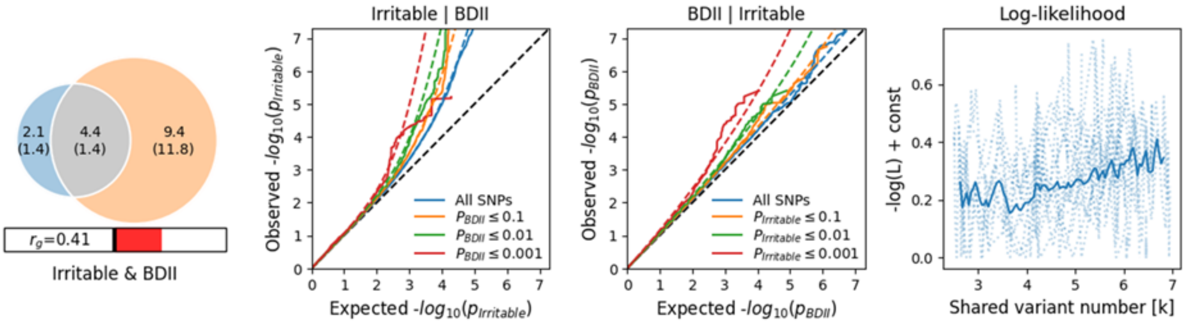

Supplementary Figure 4d. Major depressive disorder

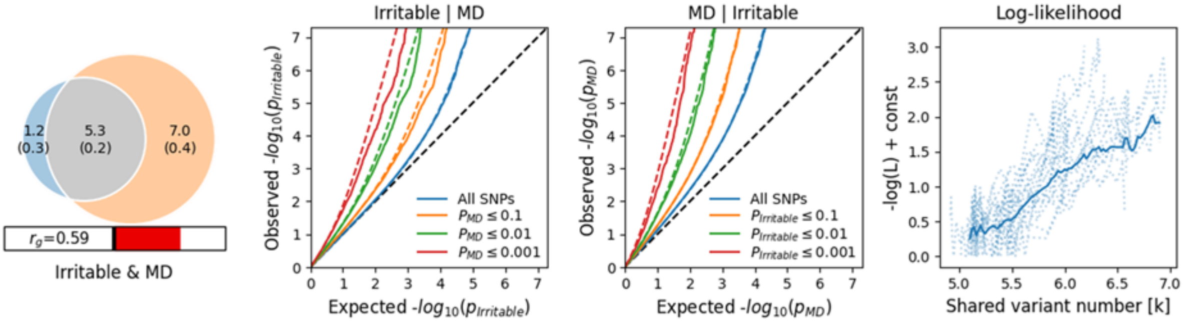

Supplementary Figure 4e. Autism spectrum disorder

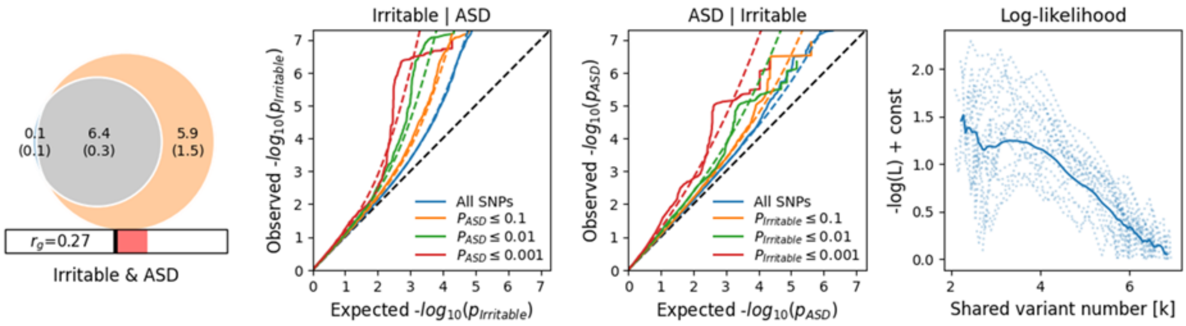

Supplementary Figure 4f. Attention deficit/hyperactivity disorder

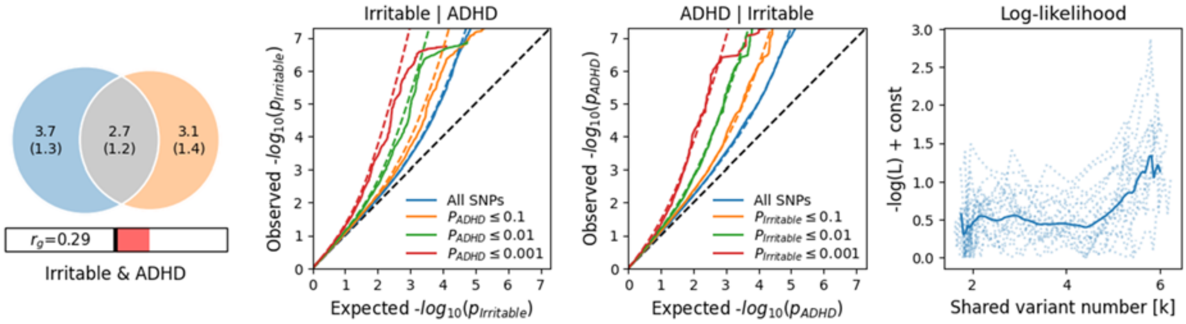

Supplementary Figure 4g. Tourette's syndrome

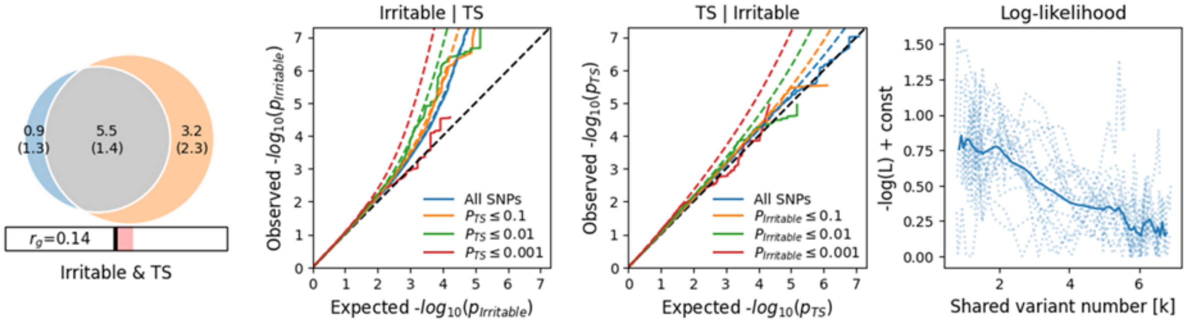

Supplementary Figure 4h. Obsessive-compulsive disorder

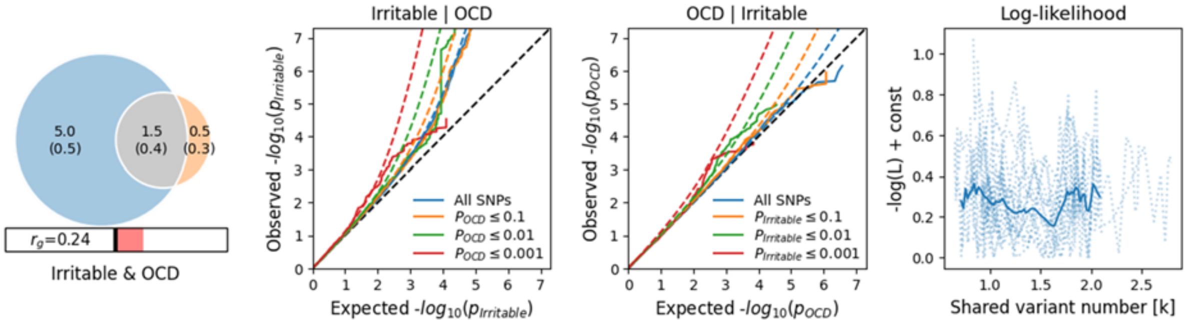

Supplementary Figure 4i. Anorexia nervosa

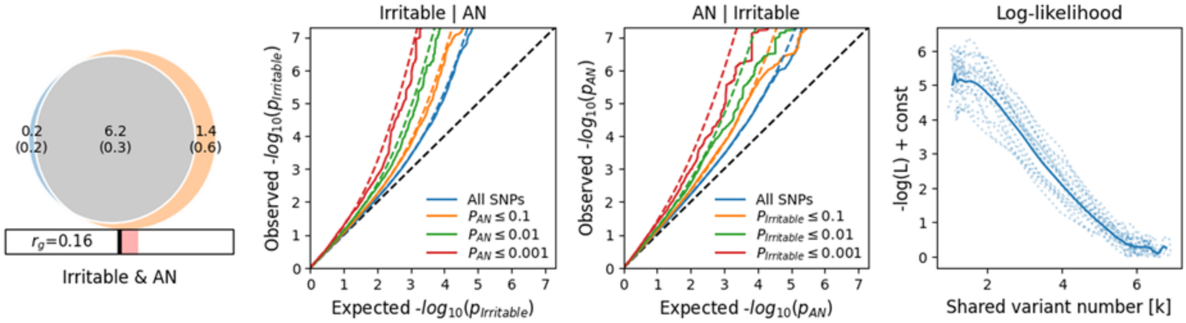

Supplementary Figure 5. Tissue specificity significantly associated with mapped genes for loci from the conditional FDR results. 54 tissue types from GTEx v8 were selected for gene expression data sets. Significantly enriched DEG sets with $P_{bon} < 0.05$ are shown in red. For conditional FDR results of psychiatric disorders given irritability, mapped genes from additionally identified loci were used.

Supplementary Figure 5a. condFDR results of schizophrenia given irritability

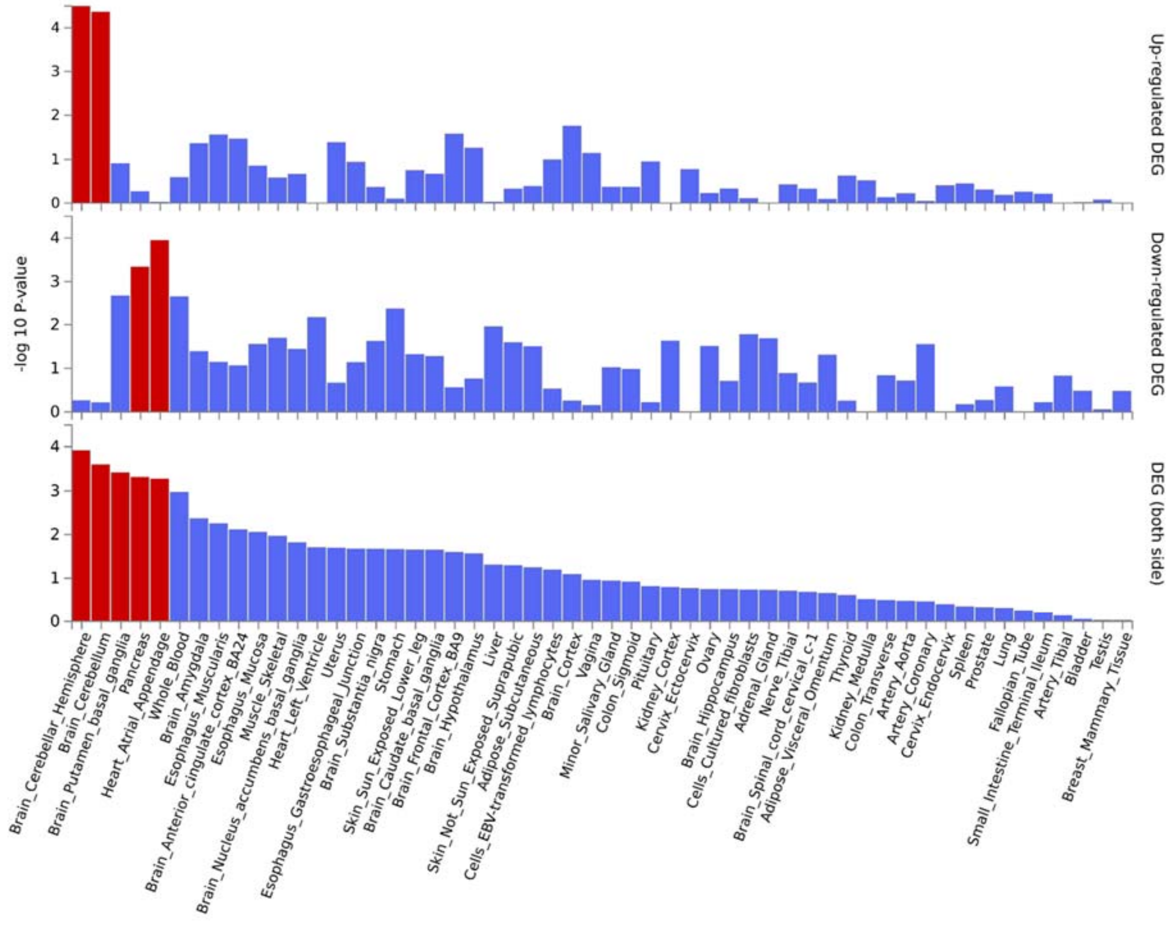

Supplementary Figure 5b. condFDR results for bipolar I disorder given irritability

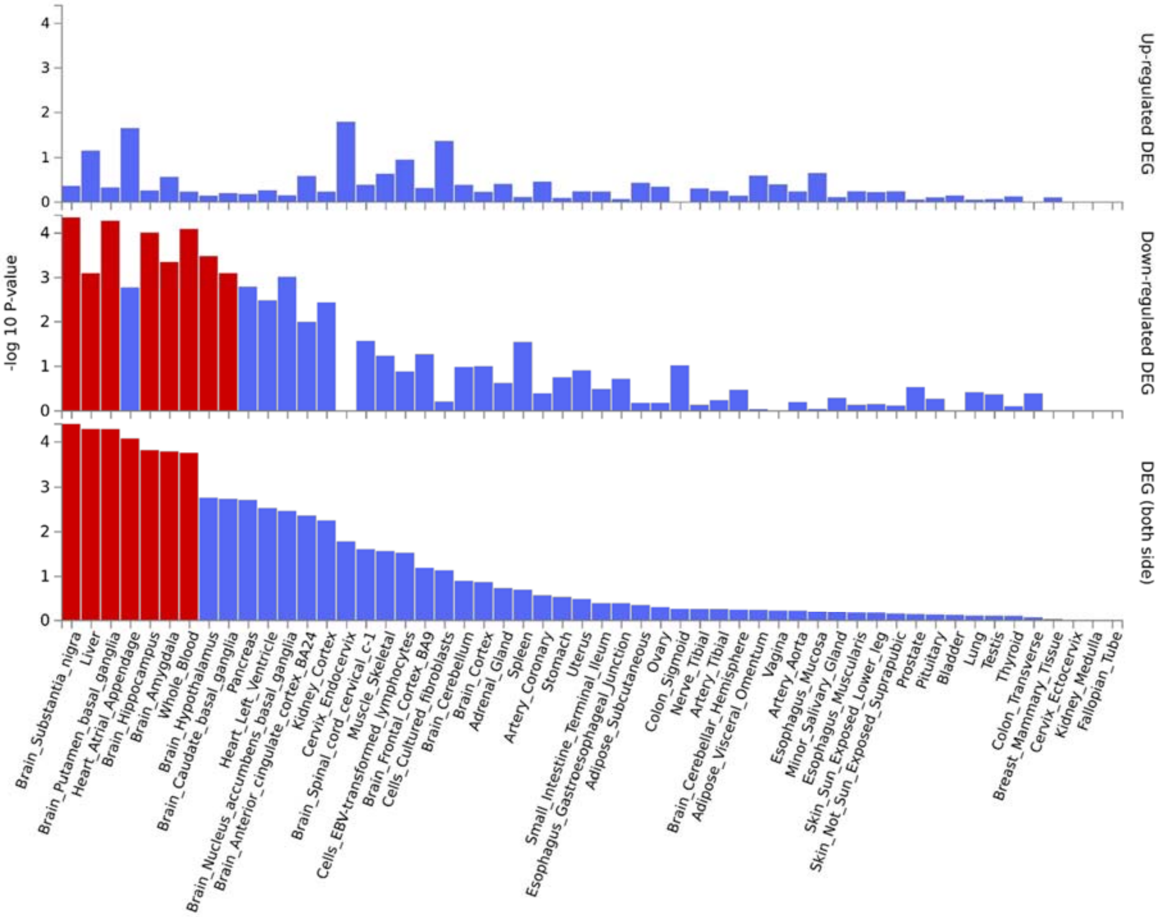

Supplementary Figure 5c. condFDR results of major depressive disorder given irritability

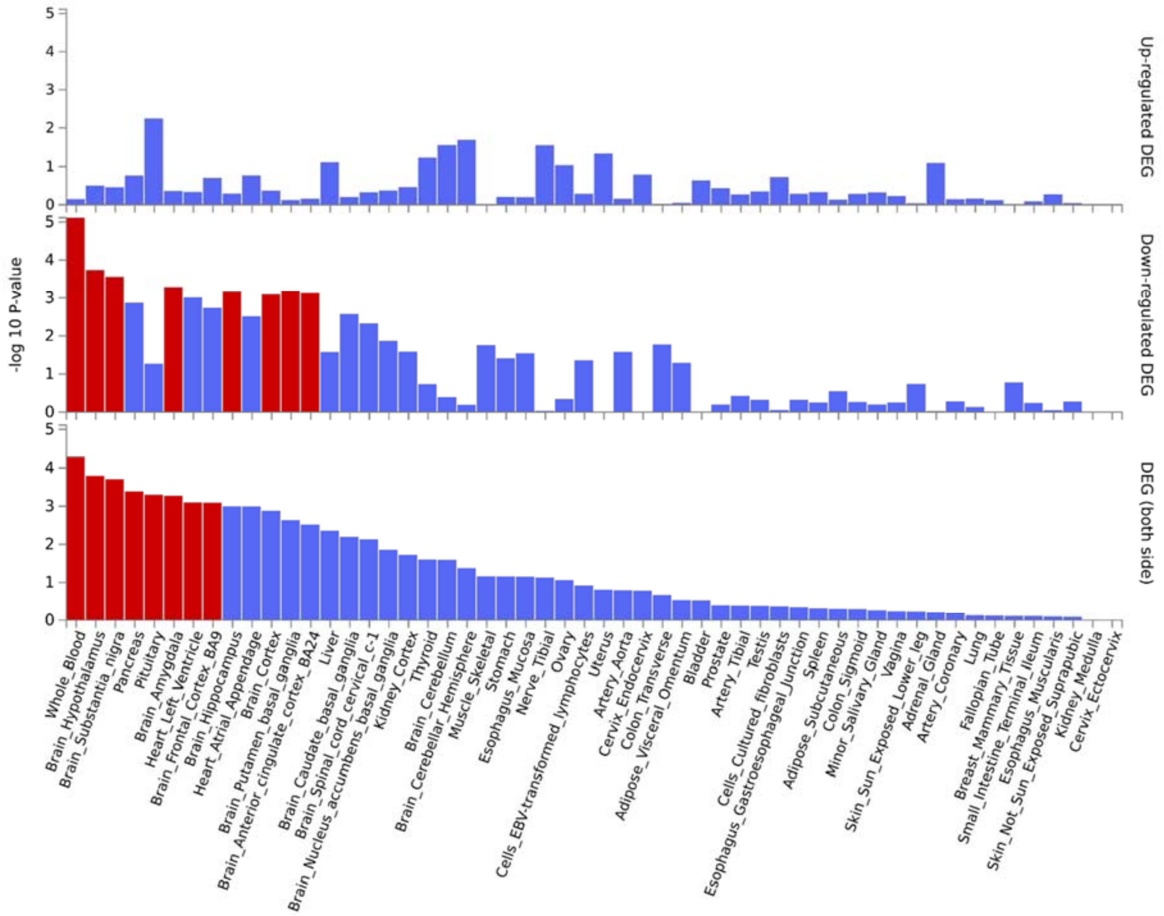

Supplementary Figure 6. Manhattan plot in $-\log_{10}$ scale of conditional/conjunctional FDR values for irritability and psychiatric disorders. SNPs with $-\log_{10}$(condFDR) > 2 (*i.e.* FDR < 0.01) or $-\log_{10}$(conjFDR) > 1.3 (*i.e.* FDR < 0.05) are shown with large circles. A black outline around the circles indicates the most significant SNP in each LD block.

Supplementary Figure 6a. condFDR results of irritability given schizophrenia

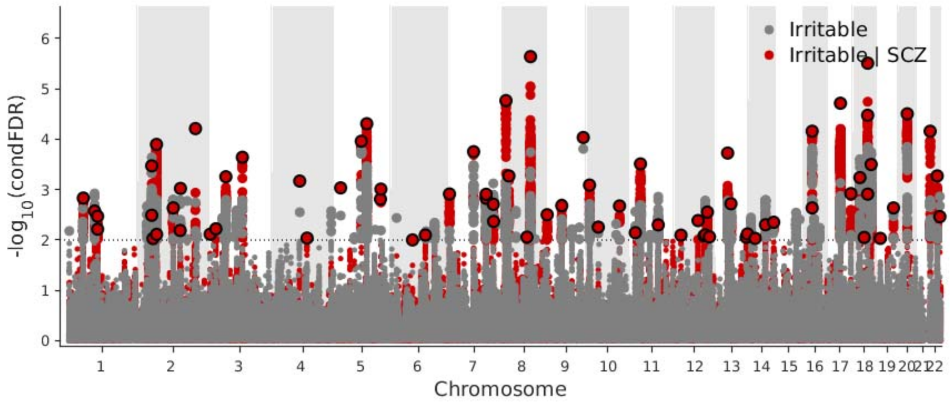

Supplementary Figure 6b. condFDR results of irritability given bipolar I disorder

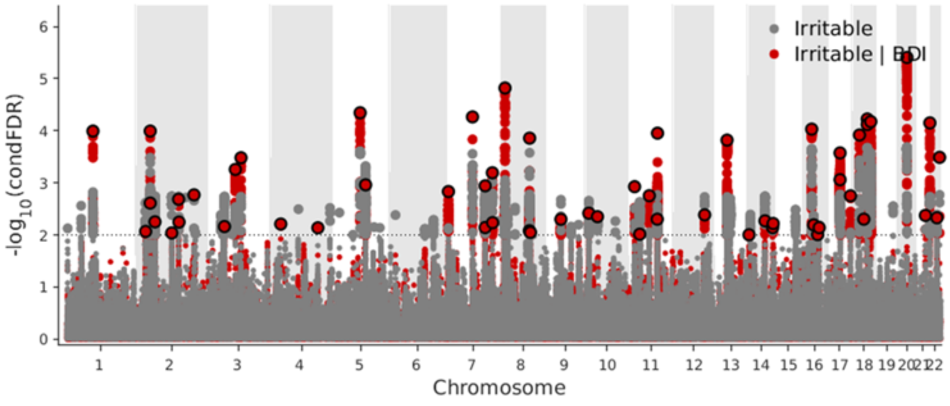

Supplementary Figure 6c. condFDR results of irritability given major depressive disorder

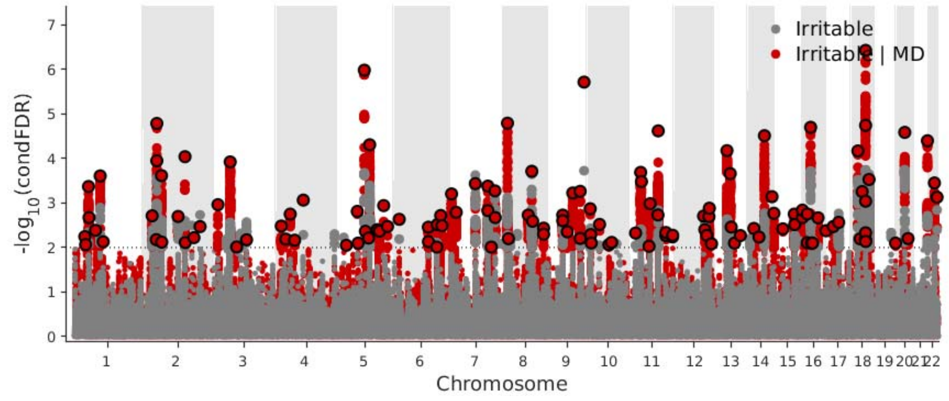

Supplementary Figure 6d. conjFDR results of irritability and schizophrenia

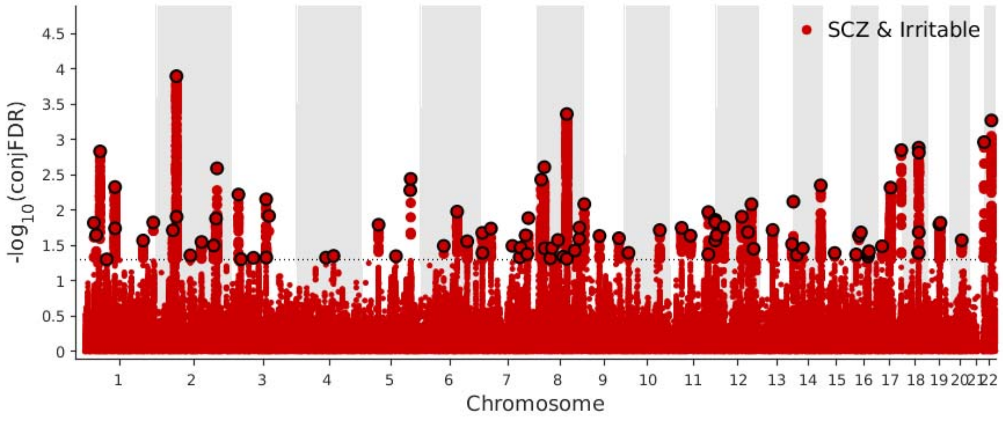

Supplementary Figure 6e. conjFDR results of irritability and bipolar I disorder

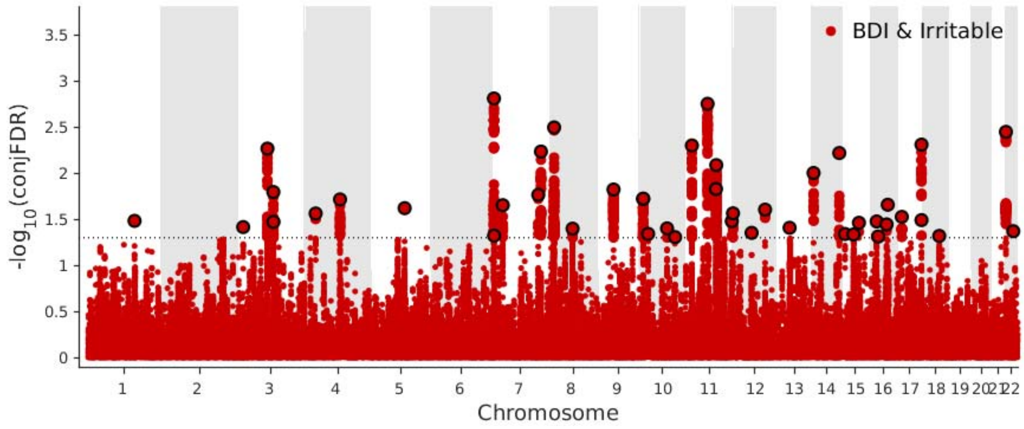

Supplementary Figure 6f. conjFDR results of irritability and major depressive disorder

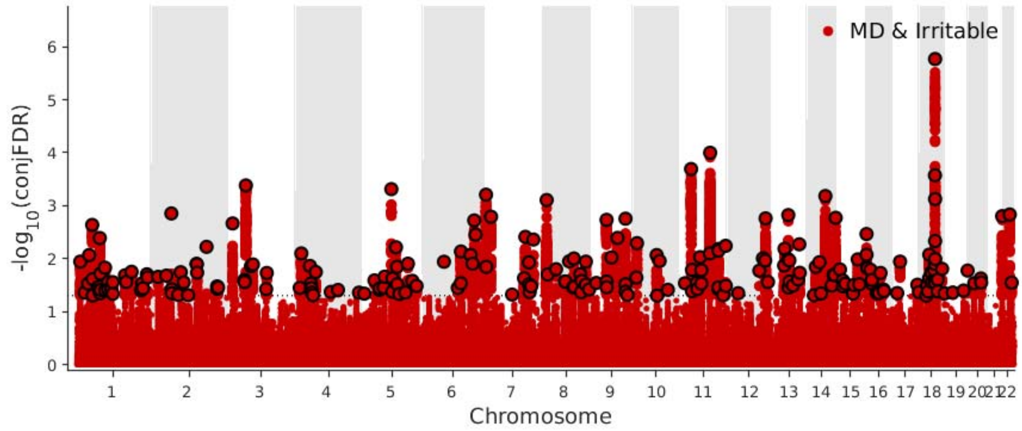

Supplementary Figure 7. Tissue specificity significantly associated with mapped genes for loci from the conditional/conjunctional FDR results. 54 tissue types from GTEx v8 were selected for gene expression data sets. Significantly enriched DEG sets with $P_{bon} < 0.05$ are shown in red.

Supplementary Figure 7a. condFDR results of irritability given schizophrenia

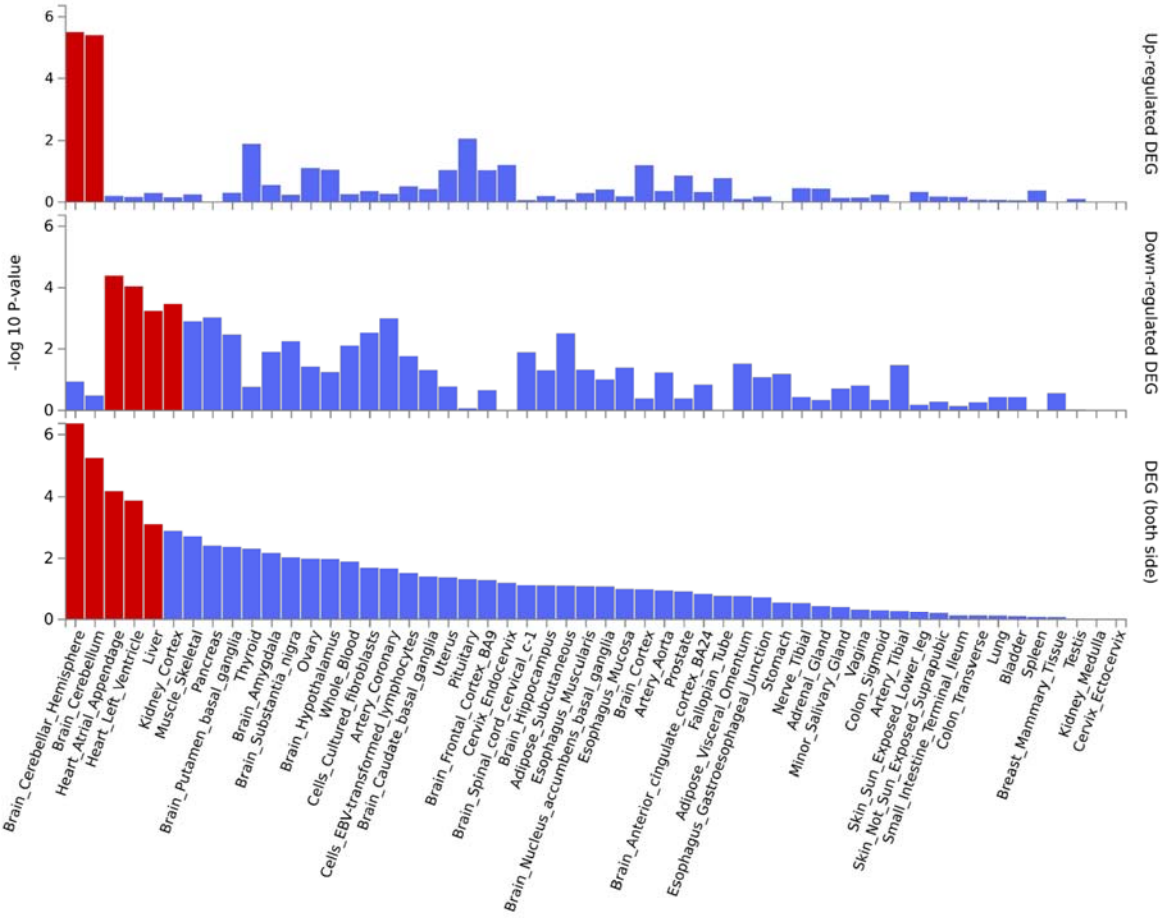

Supplementary Figure 7b. condFDR results of irritability given bipolar I disorder

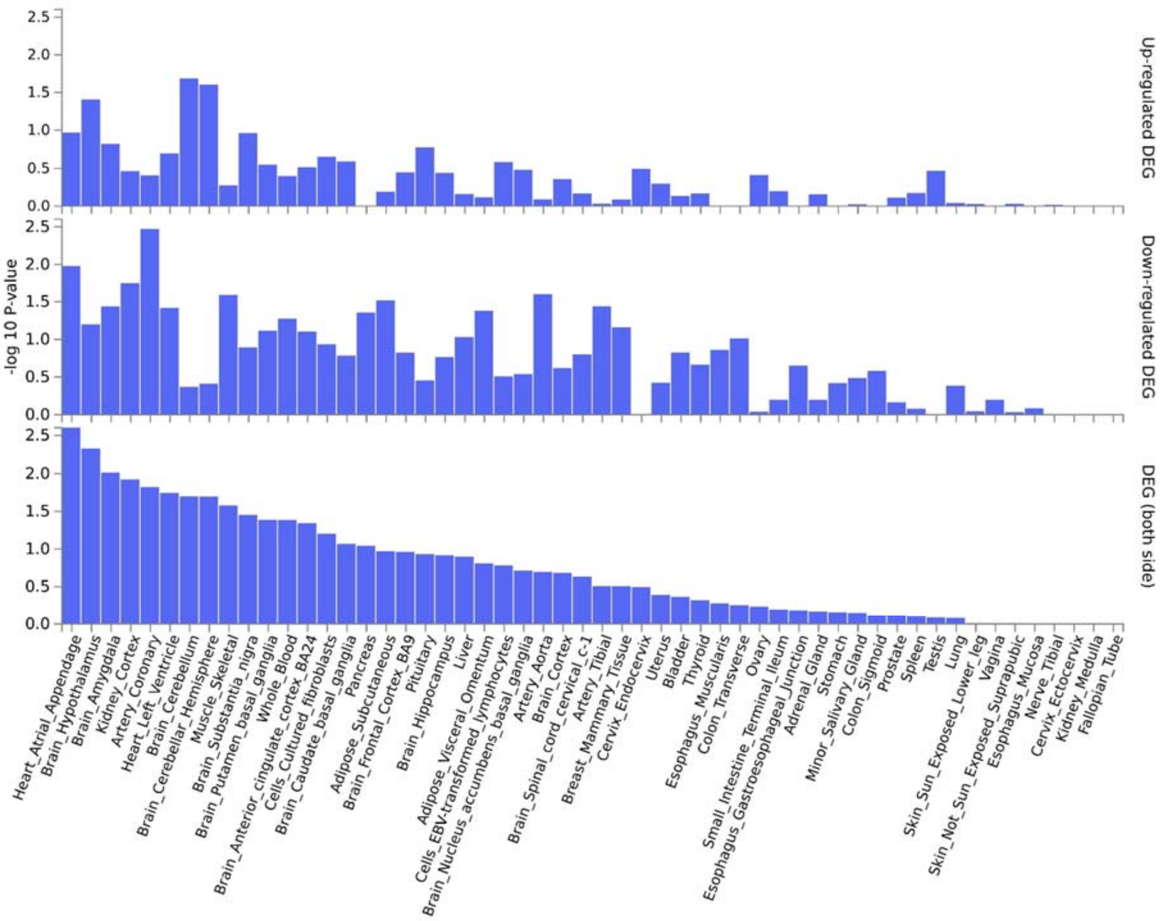

Supplementary Figure 7c. condFDR results of irritability given major depressive disorder

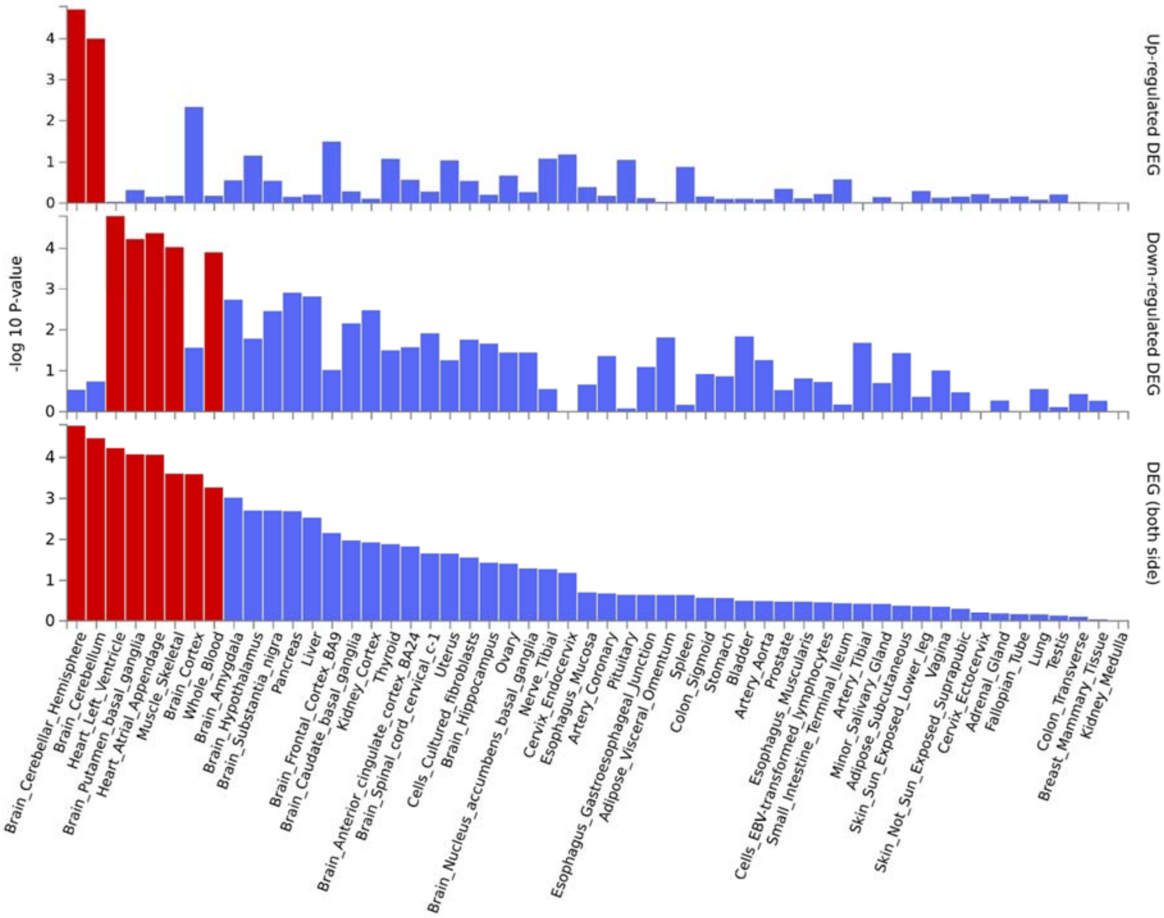

Supplementary Figure 7d. conjFDR results of irritability and schizophrenia

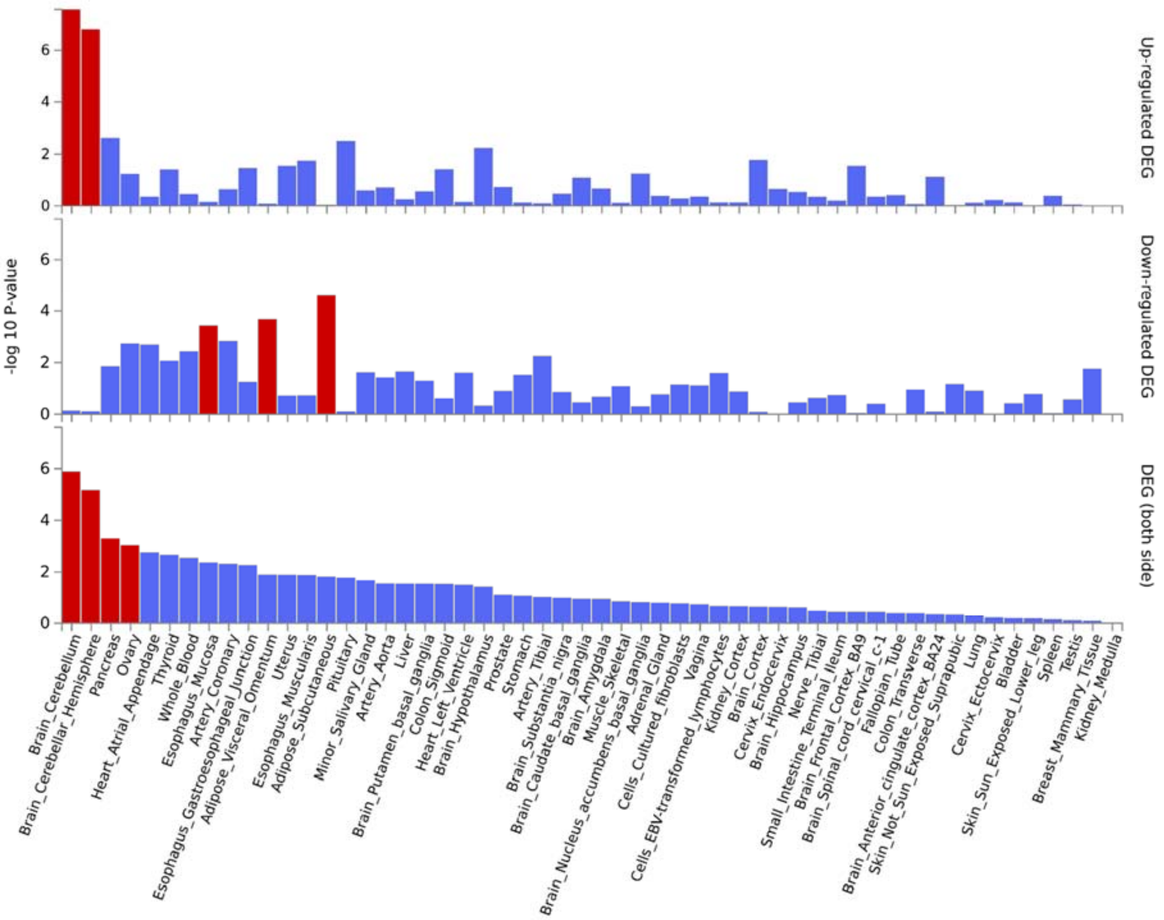

Supplementary Figure 7e. conjFDR results of irritability and bipolar I disorder

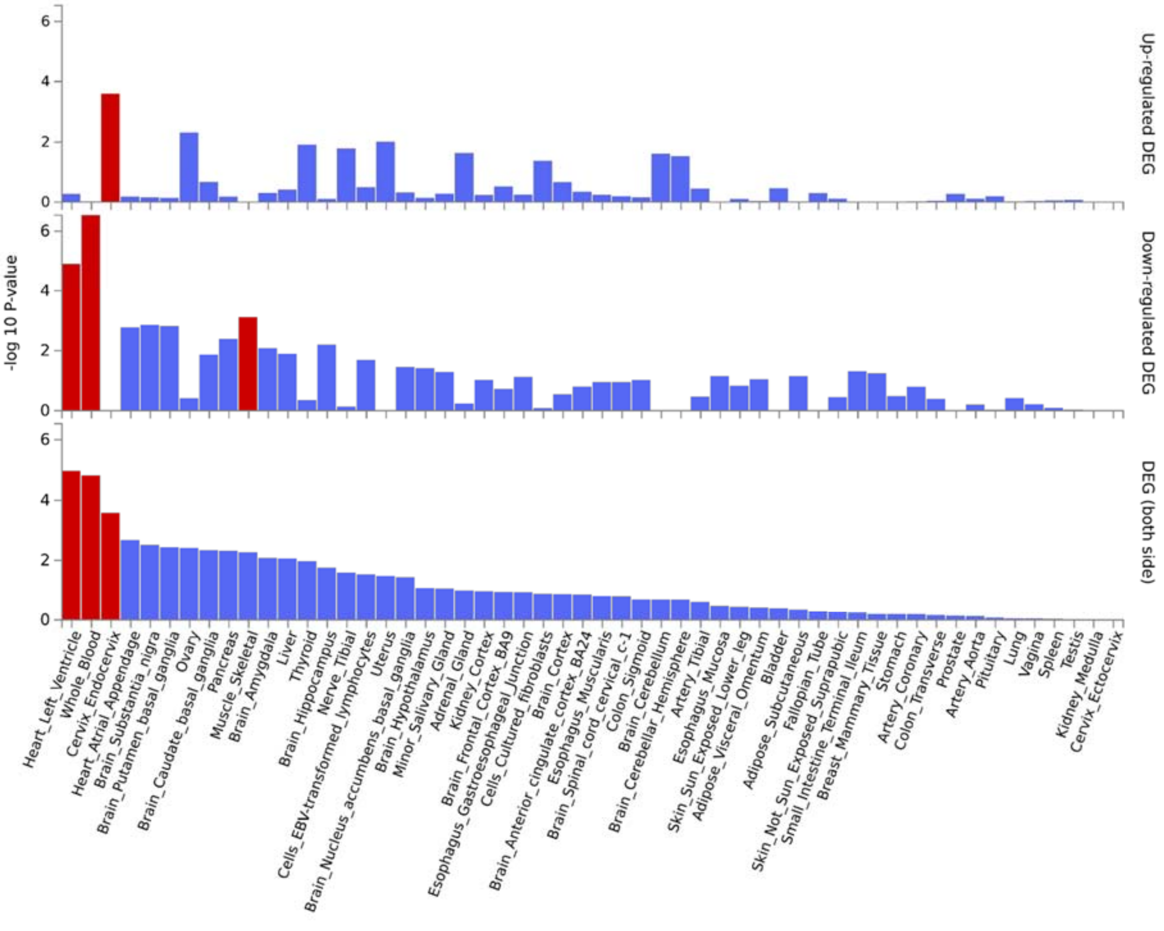

Supplementary Figure 7f. conjFDR results of irritability and major depressive disorder

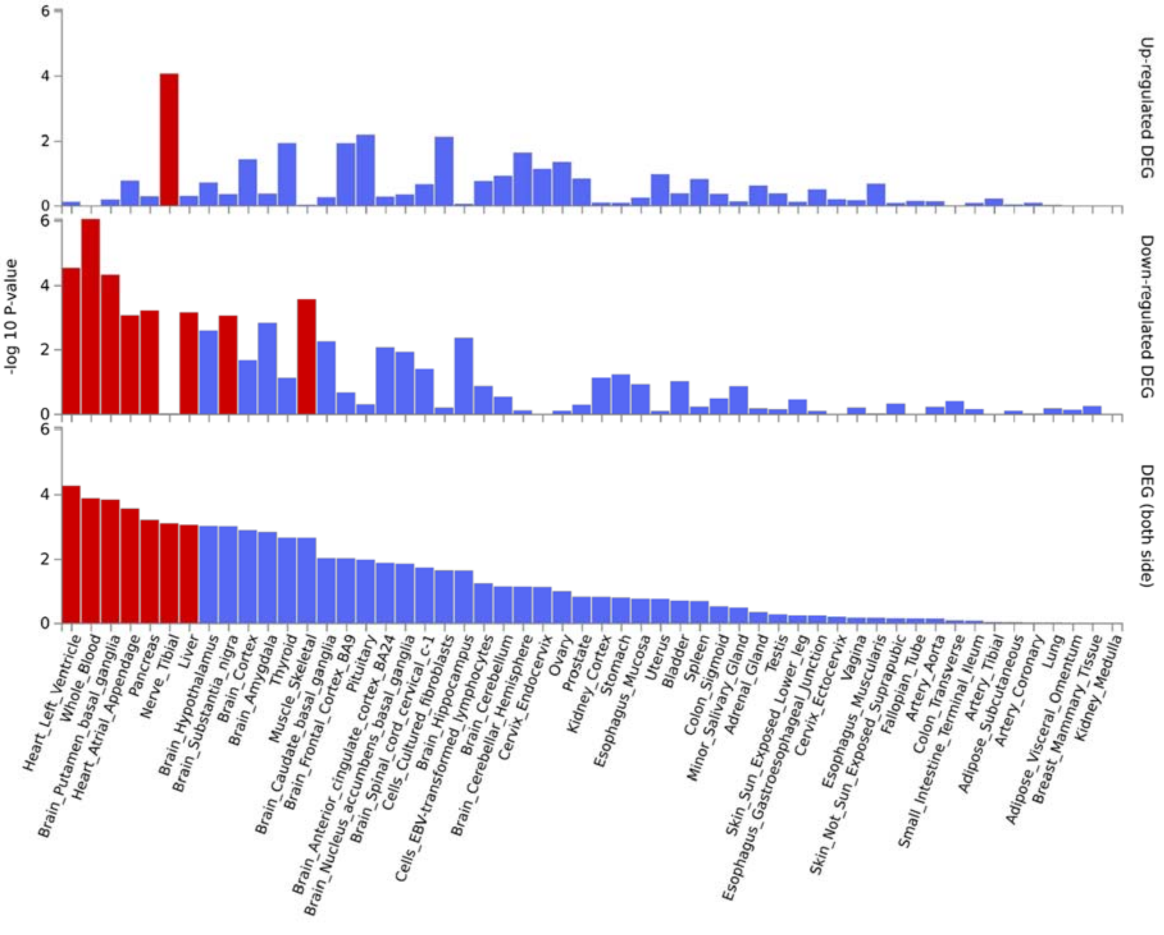

Supplementary Figure 8. Sex-stratified analyses of irritability (female: 52,529 cases and 152,268 controls; male: 53,446 cases and 121,263 controls).

Supplementary Figure 8a. Manhattan plot for GWAS of irritability (male)

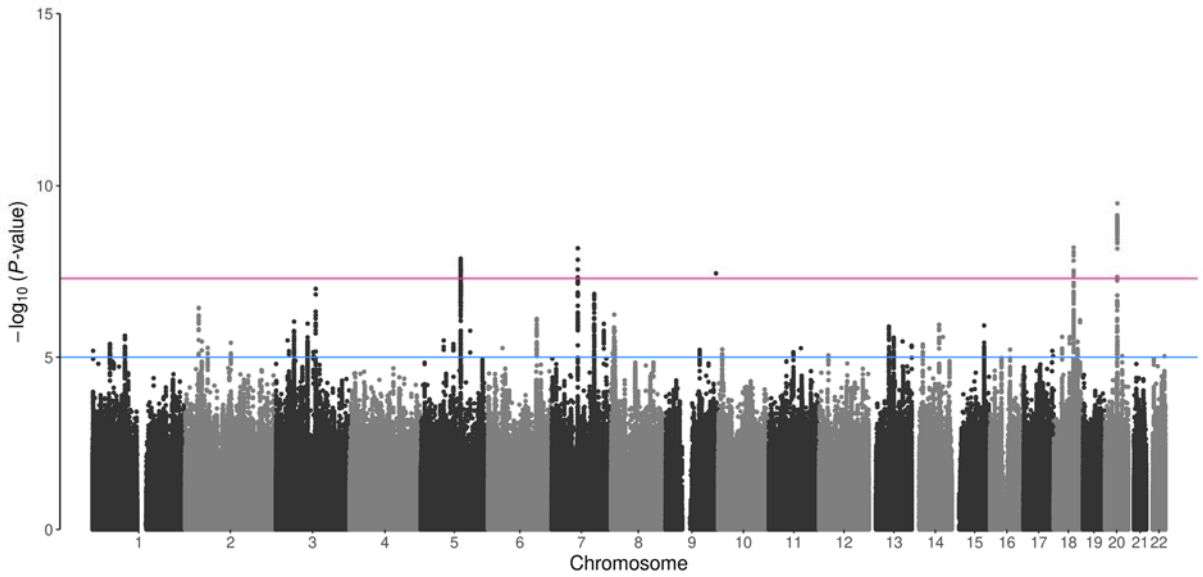

Supplementary Figure 8b. Manhattan plot for GWAS of irritability (female)

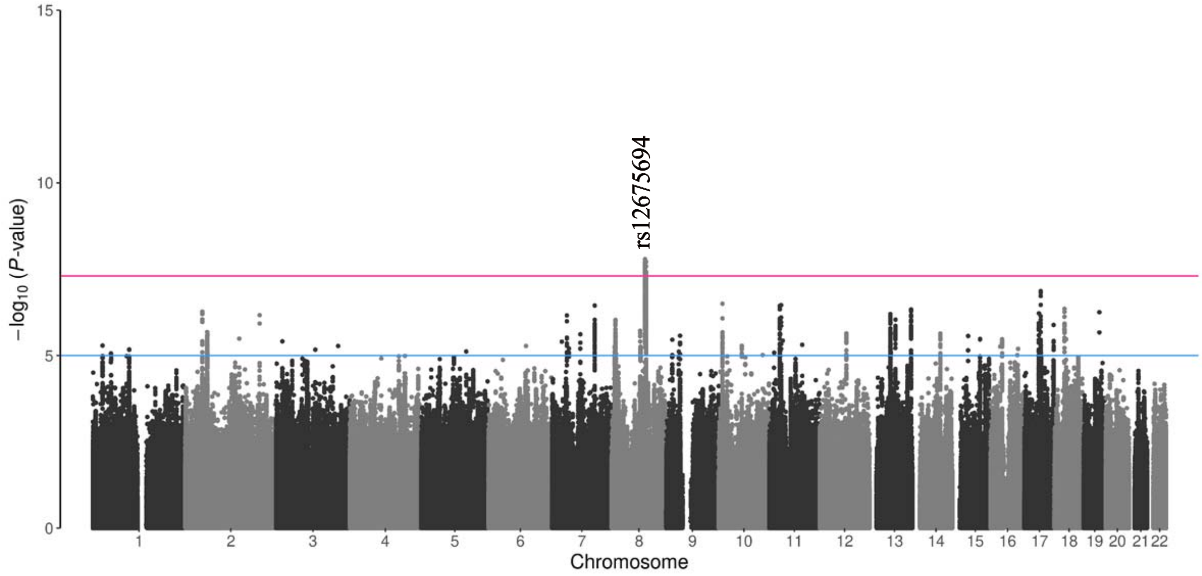

Supplementary Figure 8c. Quantile-quantile plot for GWAS of irritability (male)

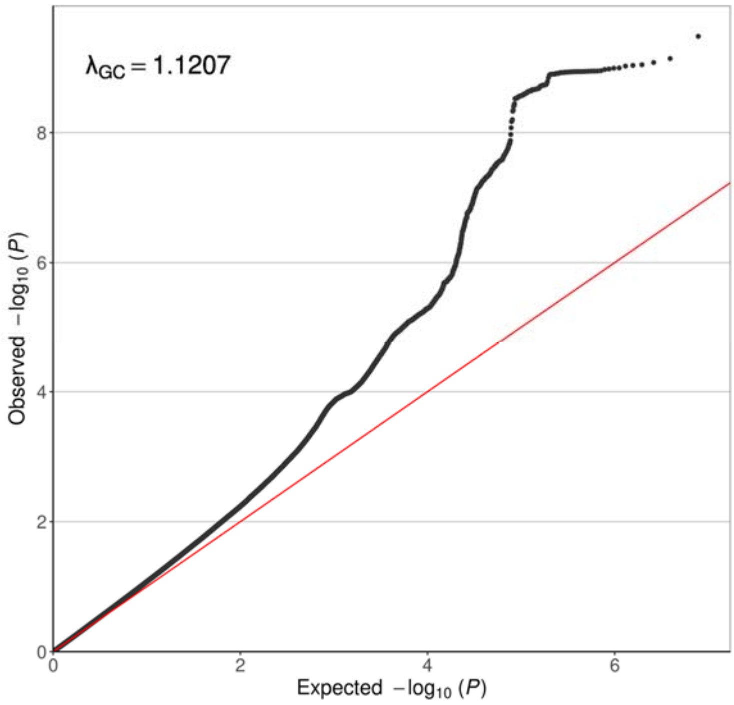

Supplementary Figure 8d. Quantile-quantile plot for GWAS of irritability (female)

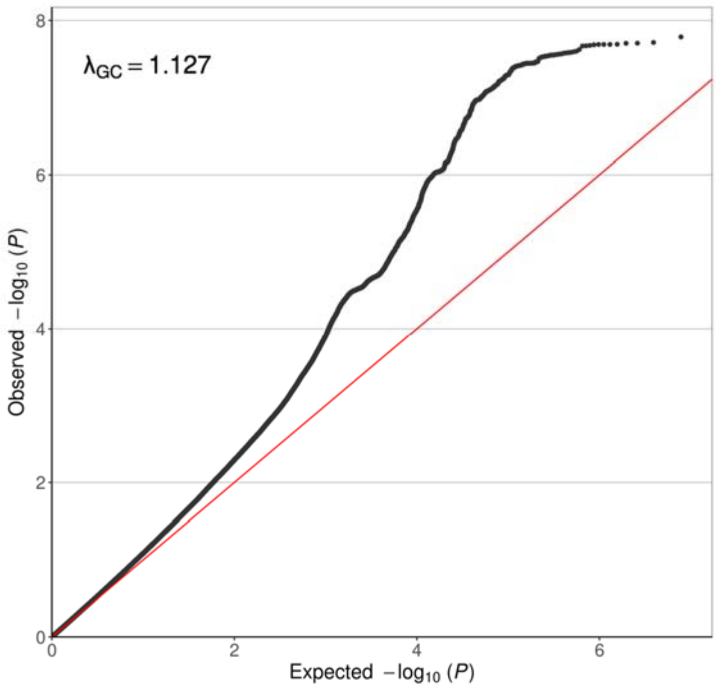

Supplementary Figure 8e. Partitioned heritability analysis for GWAS of irritability (male)

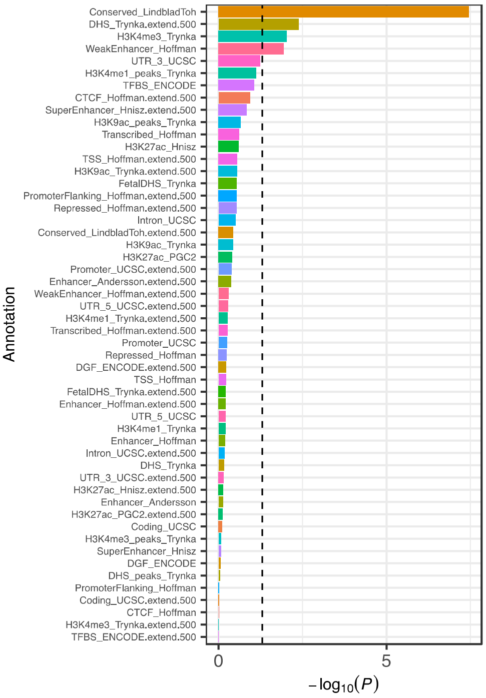

Supplementary Figure 8f. Partitioned heritability analysis for GWAS of irritability (female)

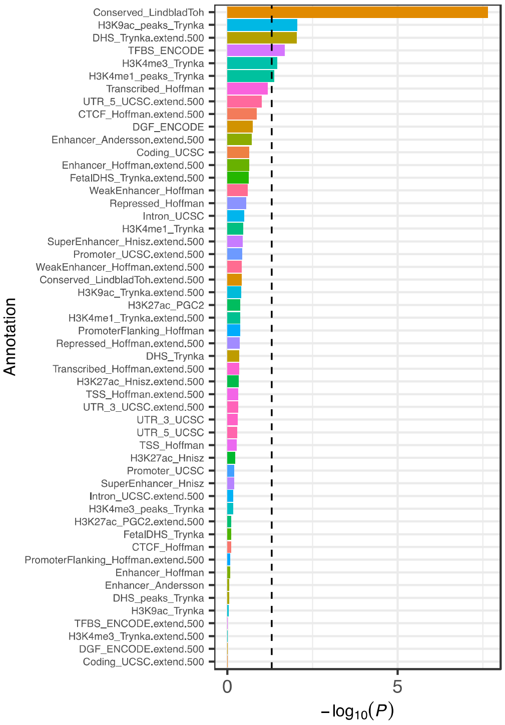

Supplementary Figure 8g. Results of multiple-tissue analysis using gene expression data for GWAS of irritability (male)

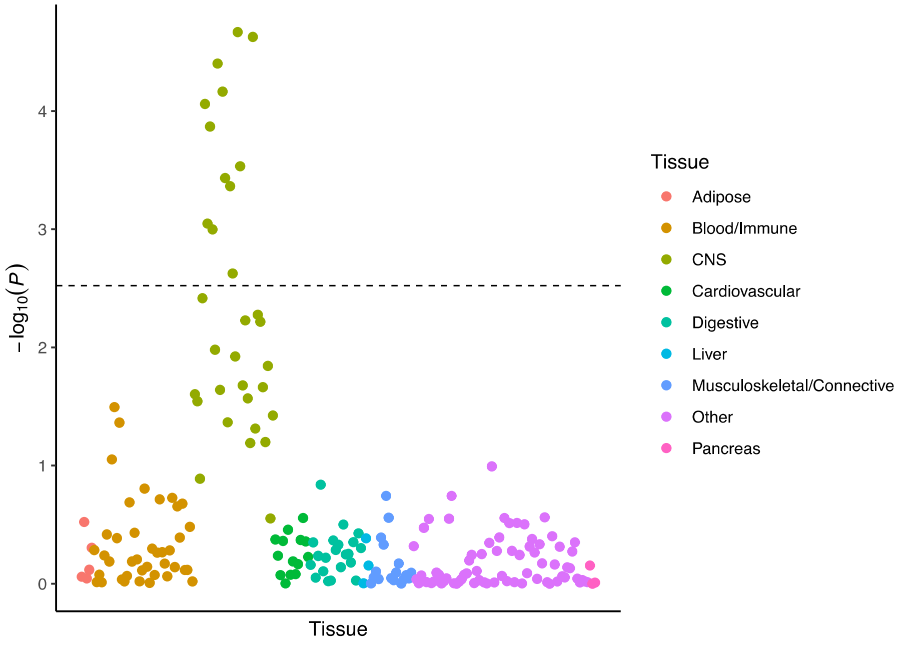

Supplementary Figure 8h. Results of multiple-tissue analysis using chromatin data for GWAS of irritability (male)

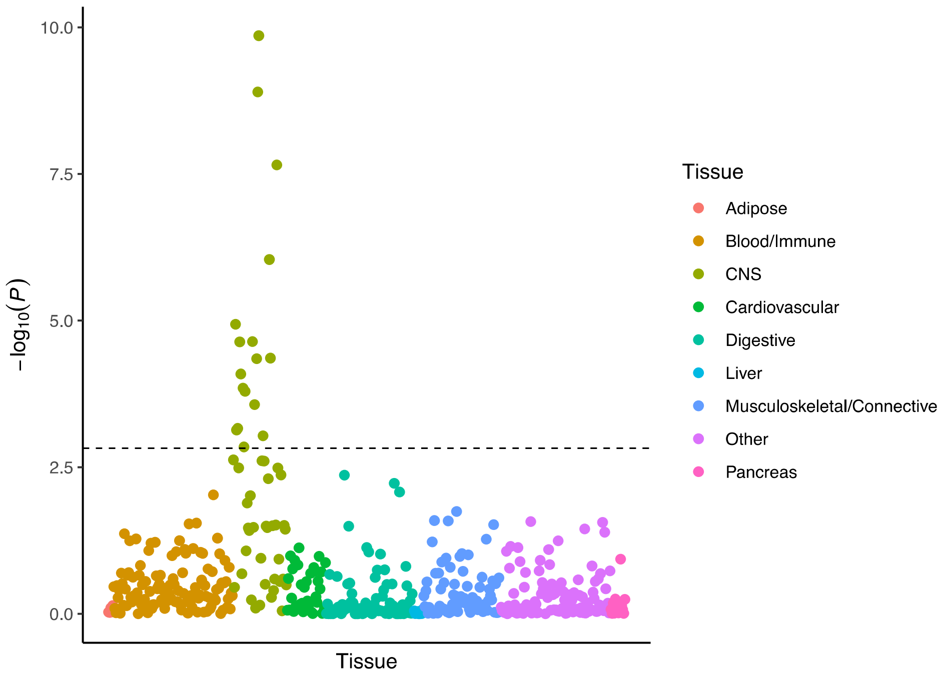

Supplementary Figure 8i. Results of multiple-tissue analysis using gene expression data for GWAS of irritability (female)

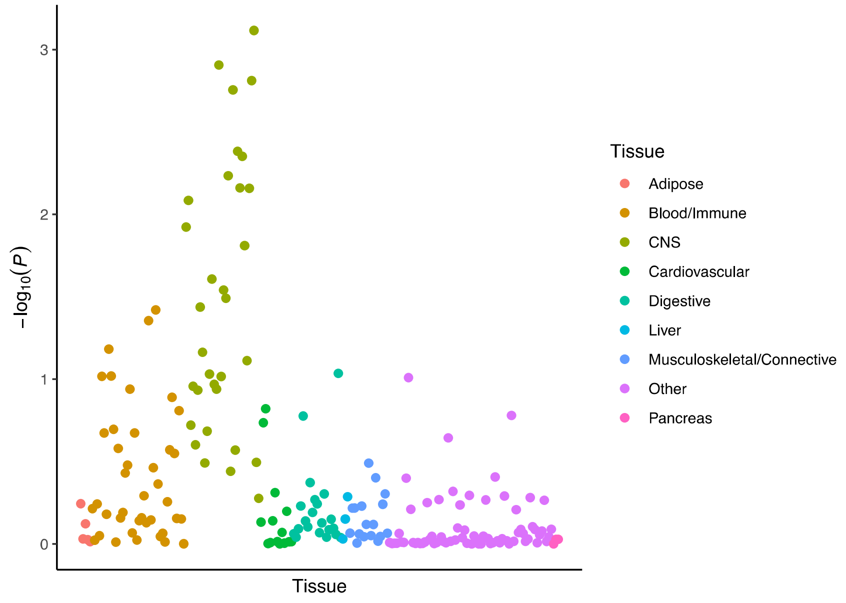

Supplementary Figure 8j. Results of multiple-tissue analysis using chromatin data for GWAS of irritability (female)

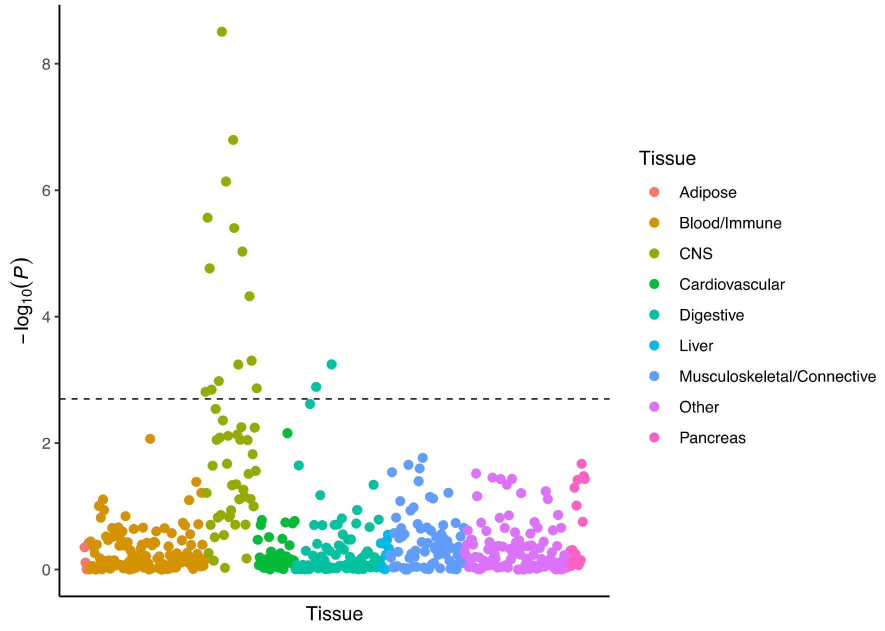

Supplementary Figure 8k. Genetic correlation estimates between irritability (male) and other traits

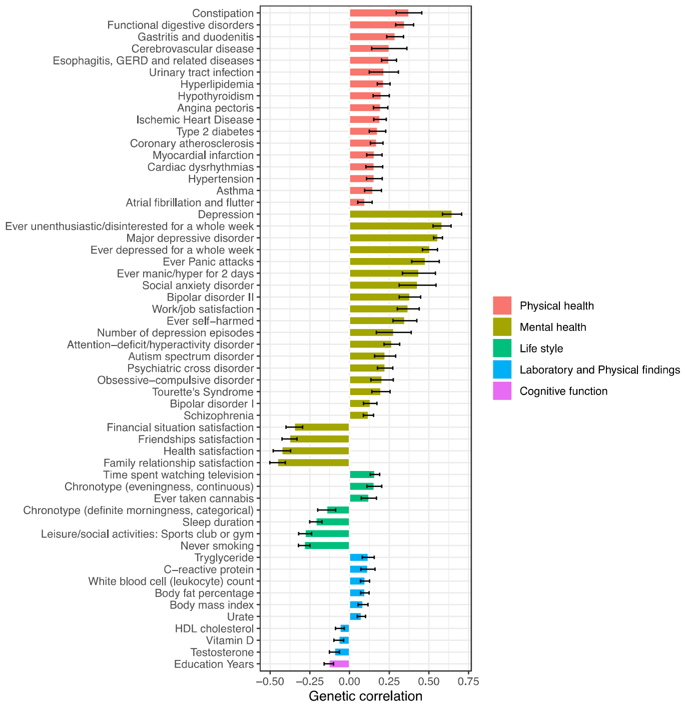

Supplementary Figure 8l. Genetic correlation estimates between irritability (female) and other traits

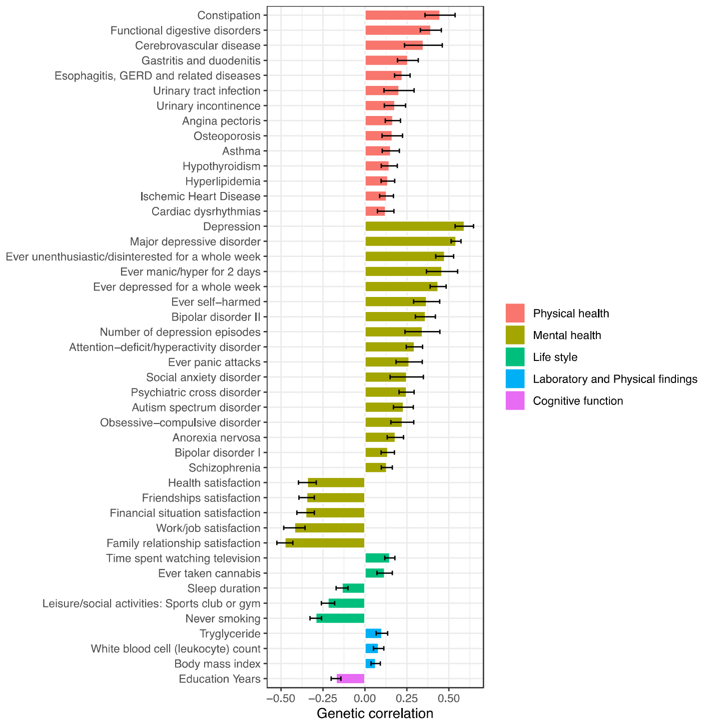

Supplementary Figure 8m. Genetic correlation estimates between irritability (male) and psychiatric disorders

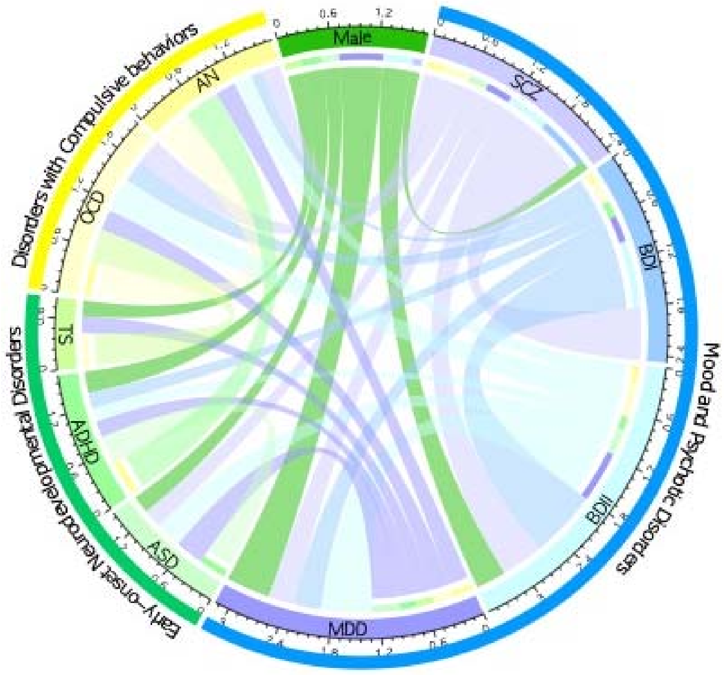

Supplementary Figure 8n. Genetic correlation estimates between irritability (female) and psychiatric disorders

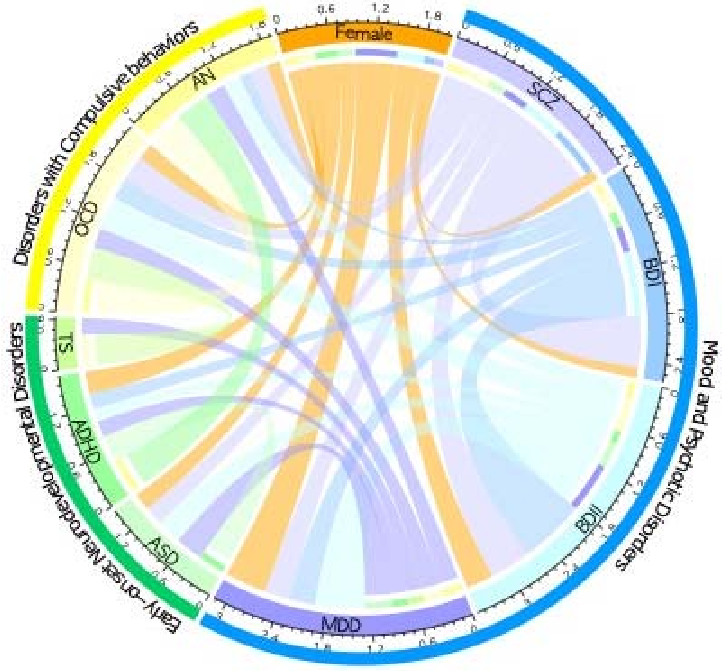

Supplementary Figure 8o. Shared polygenicity underlying irritability (male) and schizophrenia

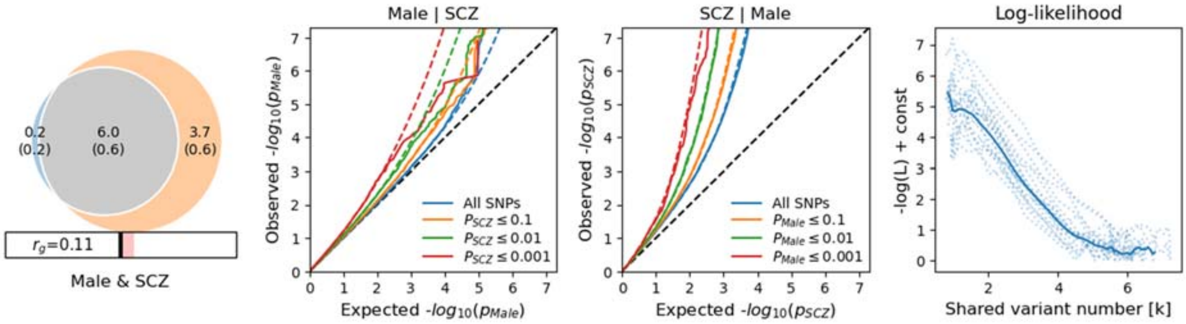

Supplementary Figure 8p. Shared polygenicity underlying irritability (male) and bipolar I disorder

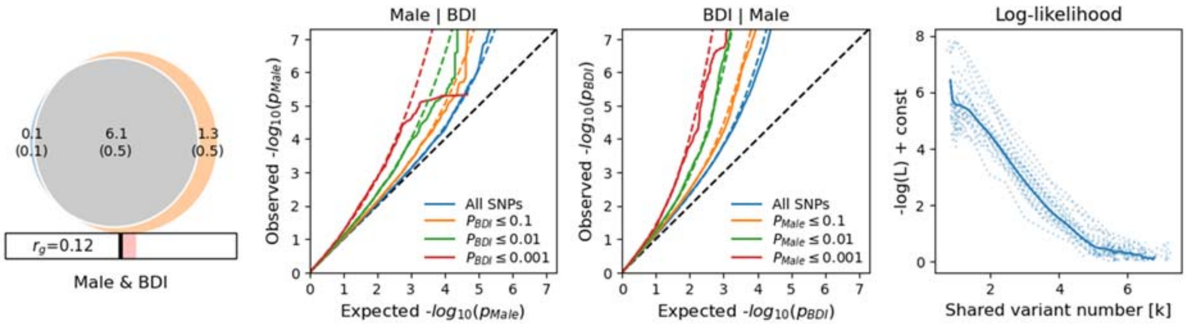

Supplementary Figure 8q. Shared polygenicity underlying irritability (male) and major depressive disorder

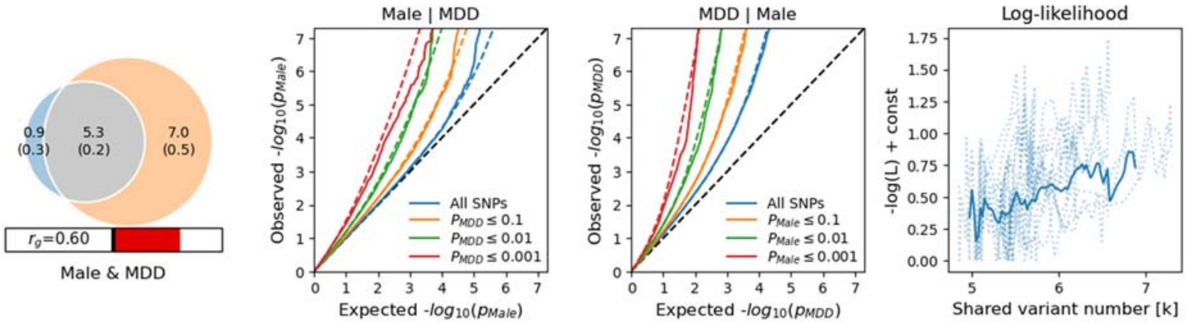

Supplementary Figure 8r. Shared polygenicity underlying irritability (female) and schizophrenia

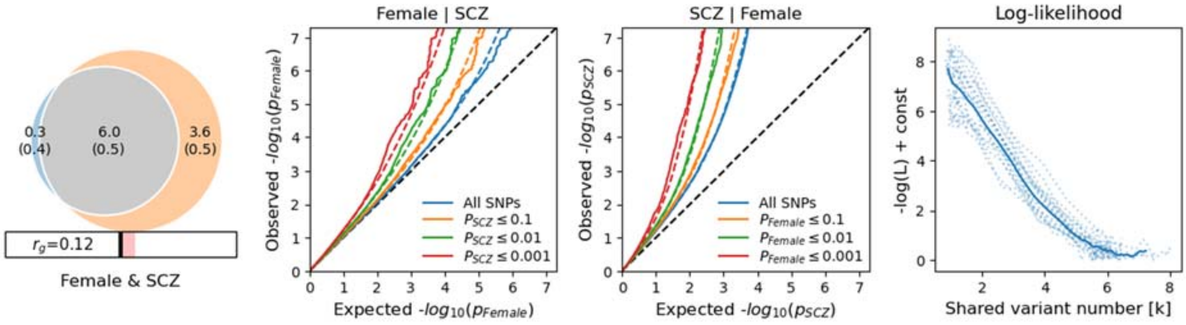

Supplementary Figure 8s. Shared polygenicity underlying irritability (female) and bipolar I disorder)

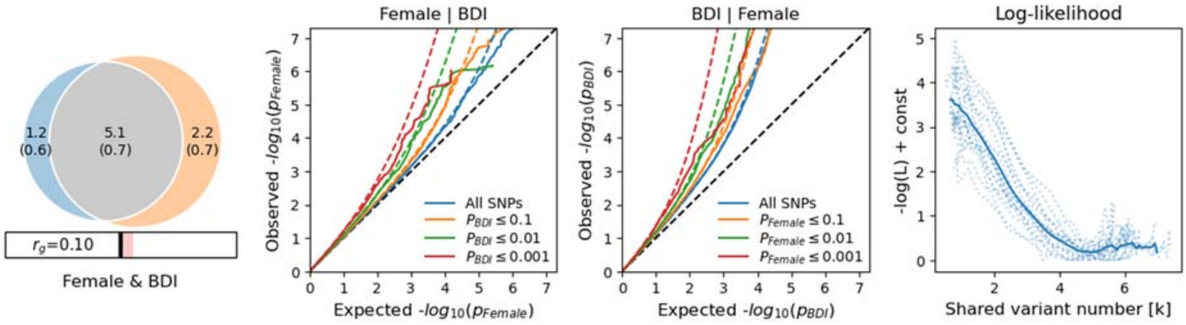

Supplementary Figure 8t. Shared polygenicity underlying irritability (female) and major depressive disorder

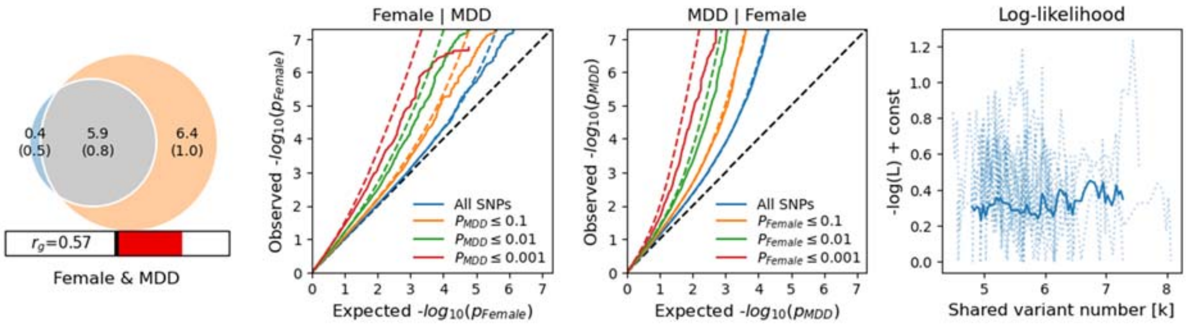

Supplementary Figure 9. Multiple-tissue analysis restricted to the conserved genomic loci defined by Lindblad-Toh et al.

Supplementary Figure 9a. Results of multiple-tissue analysis using gene expression data

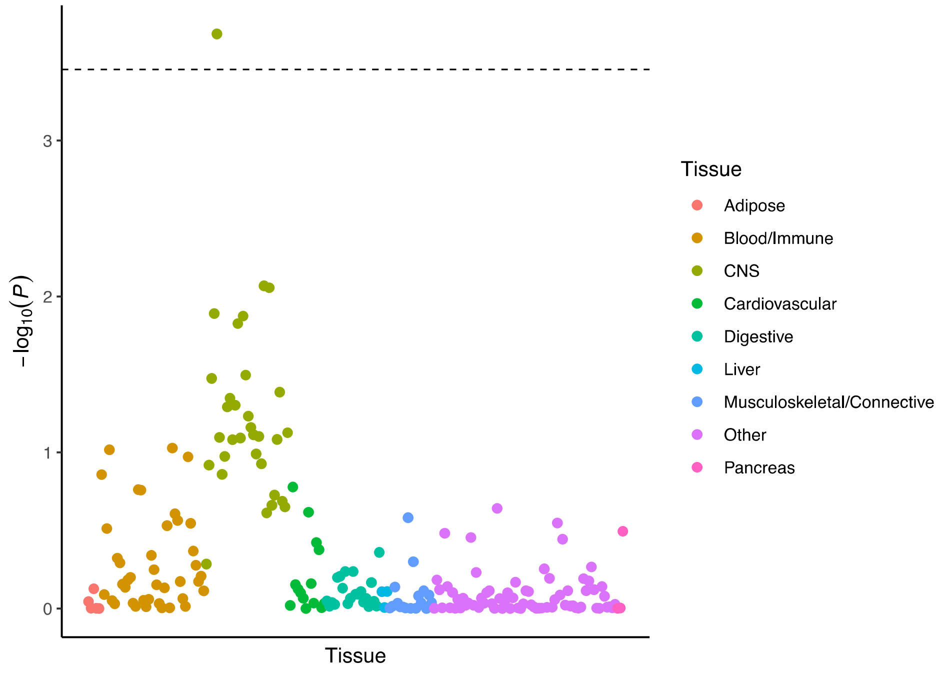

Supplementary Figure 9b. Results of multiple-tissue analysis using chromatin data

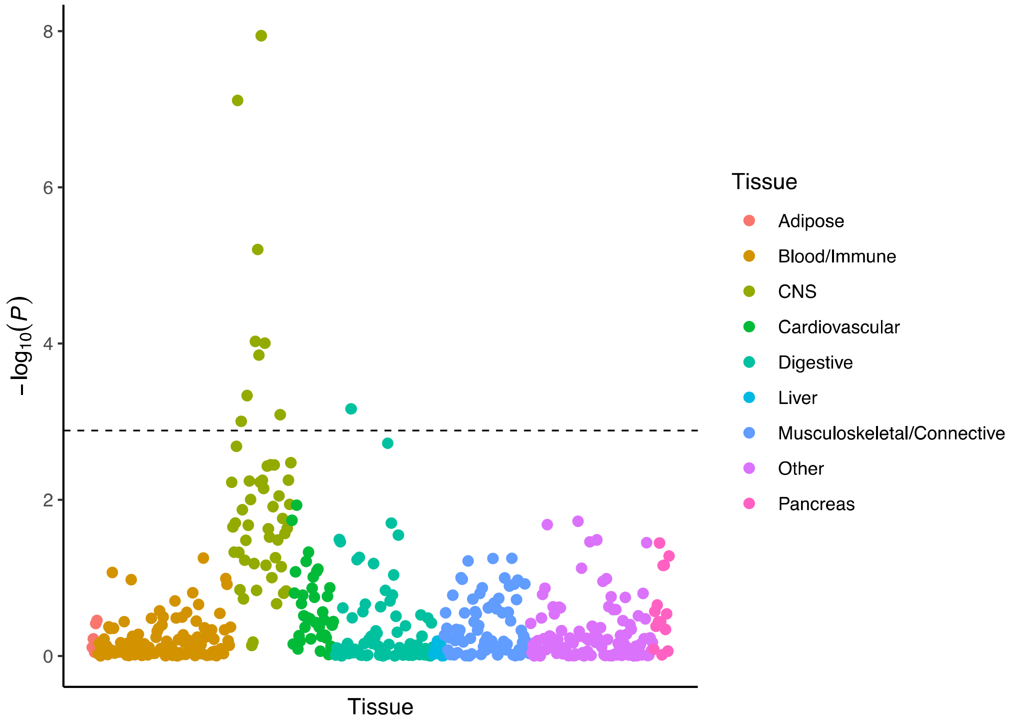

Contents and Legends

Supplementary Table 1. Demographics of the participants in terms of irritability
Supplementary Table 2. eQTL results for irritability loci
Supplementary Table 3. Geneset analysis
Supplementary Table 4. Enrichment for heritability partitioned based on 52 functional genomic annotation
Supplementary Table 5. Results from multiple-tissue analysis of gene expression using LDSC-SEG
Supplementary Table 6. Results from the central nervous system (Cahoy) gene expression using LDSC-SEG
Supplementary Table 7. Results from multiple-tissue analysis of chromatin data (validation) using LDSC-SEG
Supplementary Table 8. Genetic correlation between irritability and other traits (non-neuroimaging traits)
Supplementary Table 9. Genetic correlation between irritability and neuroimaging traits
Supplementary Table 10. Summary of psychiatric disorder datasets
Supplementary Table 11. Genetic correlation between irritability and psychiatric disorders (LDSC)
Supplementary Table 12. Univariate analysis (MiXeR)
Supplementary Table 13. Bivariate analysis between irritability and psychiatric disorders (MiXeR)
Supplementary Table 14. Distinct genomic loci associated with schizophrenia at condFDR<0.01 given association with irritability
Supplementary Table 15. Distinct genomic loci associated with bipolar I disorder at condFDR<0.01 given association with irritability
Supplementary Table 16. Distinct genomic loci associated with major depressive disorder at condFDR<0.01 given association with irritability
Supplementary Table 17. eQTL mapping for additional genomic loci from the condFDR results for psychiatric disorders conditional on irritability
Supplementary Table 18. Distinct genomic loci associated with irritability at condFDR<0.01 given association with each psychiatric disorder
Supplementary Table 19. eQTL mapping for distinct genomic loci from the condFDR results for irritability conditional on each psychiatric disorder
Supplementary Table 20. Distinct genomic loci shared between irritability and each psychiatric disorder at conjFDR<0.05
Supplementary Table 21. eQTL mapping for distinct genomic loci from the conjFDR results between irritability and each psychiatric disorder
Supplementary Table 22. Two-stage GWAS results for 10 randomly divided data sets

Supplementary Table 1. Demographics of the participants in terms of irritability

		Item (UKB field code) : Are you an irritable person? (1940)		
		**Yes (%)**	**No (%)**	**N**
Age	≤49	29,250 (35.3)	53,605 (64.7)	82,855
	50-59	35,418 (28.2)	89,961 (71.8)	125,379
	≥60	41,307 (24.1)	129,965 (75.9)	171,272
Sex	Female	52,529 (25.6)	152,268 (74.4)	204,797
	Male	53,446 (30.6)	121,263 (69.4)	174,709
	**Total**	105,975 (27.9)	273,531 (72.1)	**379,506**

Supplementary Table 2. eQTL results for irritability loci

a) GTEx v8 Brain

SNP	uniqID	db	tissue	gene	testedAllele	p	signed_stats	FDR	RiskIncAllele	alignedDirection	chr	pos	symbol
rs62491417	7:139835245:A:C	GTEx/v8	Brain_Caudate_basal_ganglia	ENSG00000006459	A	1.56E-08	0.709312	1.11E-06	A	+	7	139835245	KDM7A
rs62491417	7:139835245:A:C	GTEx/v8	Brain_Cerebellar_Hemisphere	ENSG00000006459	A	4.40E-07	0.477236	2.68E-06	A	+	7	139835245	KDM7A
rs62491417	7:139835245:A:C	GTEx/v8	Brain_Cerebellum	ENSG00000006459	A	1.15E-09	0.650612	4.77E-08	A	+	7	139835245	KDM7A
rs62491417	7:139835245:A:C	GTEx/v8	Brain_Cortex	ENSG00000006459	A	1.64E-14	0.883369	3.67E-14	A	+	7	139835245	KDM7A
rs62491417	7:139835245:A:C	GTEx/v8	Brain_Frontal_Cortex_BA9	ENSG00000006459	A	9.57E-11	0.76729	2.02E-08	A	+	7	139835245	KDM7A
rs62491417	7:139835245:A:C	GTEx/v8	Brain_Nucleus_accumbens_basal_ganglia	ENSG00000006459	A	9.71E-09	0.707577	7.15E-06	A	+	7	139835245	KDM7A
rs62491417	7:139835245:A:C	GTEx/v8	Brain_Putamen_basal_ganglia	ENSG00000006459	A	2.81E-10	0.878373	1.86E-07	A	+	7	139835245	KDM7A
rs62491417	7:139835245:A:C	GTEx/v8	Brain_Spinal_cord_cervical_c-1	ENSG00000006459	A	4.86E-06	0.823834	0.000656515	A	+	7	139835245	KDM7A
rs2054213	16:30971810:A:G	GTEx/v8	Brain_Anterior_cingulate_cortex_BA24	ENSG00000103496	G	2.01E-06	0.275113	0.000230056	A	-	16	30971810	STX4
rs2054213	16:30971810:A:G	GTEx/v8	Brain_Caudate_basal_ganglia	ENSG00000103510	G	3.93E-07	-0.262983	9.10E-06	A	+	16	30971810	KAT8
rs2054213	16:30971810:A:G	GTEx/v8	Brain_Caudate_basal_ganglia	ENSG00000178226	G	2.12E-05	-0.276357	5.38E-19	A	+	16	30971810	PRSS36
rs2054213	16:30971810:A:G	GTEx/v8	Brain_Cerebellar_Hemisphere	ENSG00000099365	G	2.68E-06	-0.182646	0.00160488	A	+	16	30971810	STX1B
rs2054213	16:30971810:A:G	GTEx/v8	Brain_Cerebellar_Hemisphere	ENSG00000103510	G	3.76E-09	-0.257859	4.50E-17	A	+	16	30971810	KAT8
rs2054213	16:30971810:A:G	GTEx/v8	Brain_Cerebellum	ENSG00000099365	G	2.60E-06	-0.152015	0.000791194	A	+	16	30971810	STX1B
rs2054213	16:30971810:A:G	GTEx/v8	Brain_Cerebellum	ENSG00000099381	G	6.95E-06	-0.134648	0.00390586	A	+	16	30971810	SETD1A
rs2054213	16:30971810:A:G	GTEx/v8	Brain_Cerebellum	ENSG00000103496	G	3.02E-07	-0.247032	2.06E-05	A	+	16	30971810	STX4
rs2054213	16:30971810:A:G	GTEx/v8	Brain_Cerebellum	ENSG00000103510	G	2.97E-07	-0.280564	3.51E-17	A	+	16	30971810	KAT8
rs2054213	16:30971810:A:G	GTEx/v8	Brain_Cerebellum	ENSG00000178226	G	1.86E-05	-0.378141	1.01E-22	A	+	16	30971810	PRSS36
rs2054213	16:30971810:A:G	GTEx/v8	Brain_Cortex	ENSG00000099377	G	1.57E-05	-0.220361	8.50E-05	A	+	16	30971810	HSD3B7
rs2054213	16:30971810:A:G	GTEx/v8	Brain_Cortex	ENSG00000103510	G	7.47E-08	-0.305962	2.06E-14	A	+	16	30971810	KAT8
rs2054213	16:30971810:A:G	GTEx/v8	Brain_Cortex	ENSG00000167397	G	3.13E-06	0.242905	0.000396364	A	-	16	30971810	VKORC1
rs2054213	16:30971810:A:G	GTEx/v8	Brain_Cortex	ENSG00000196118	G	6.05E-05	0.231019	1.47E-09	A	-	16	30971810	C16orf93
rs2054213	16:30971810:A:G	GTEx/v8	Brain_Frontal_Cortex_BA9	ENSG00000103496	G	1.30E-08	0.268402	9.49E-06	A	-	16	30971810	STX4
rs2054213	16:30971810:A:G	GTEx/v8	Brain_Frontal_Cortex_BA9	ENSG00000103510	G	1.45E-05	-0.241072	5.27E-07	A	+	16	30971810	KAT8

rs2054213	16:30971810:A:G	GTEx/v8	Brain_Frontal_Cortex_BA9	ENSG00000178226	G	3.95E-05	-0.35752	1.22E-08	A	+	16	30971810	PRSS36
rs2054213	16:30971810:A:G	GTEx/v8	Brain_Hypothalamus	ENSG00000099377	G	4.16E-06	-0.314841	0.0094501	A	+	16	30971810	HSD3B7
rs2054213	16:30971810:A:G	GTEx/v8	Brain_Hypothalamus	ENSG00000103496	G	7.38E-07	0.208595	5.32E-06	A	-	16	30971810	STX4
rs2054213	16:30971810:A:G	GTEx/v8	Brain_Hypothalamus	ENSG00000103510	G	1.18E-06	-0.271052	3.84E-06	A	+	16	30971810	KAT8
rs2054213	16:30971810:A:G	GTEx/v8	Brain_Hypothalamus	ENSG00000178226	G	7.11E-05	-0.294215	1.54E-07	A	+	16	30971810	PRSS36
rs2054213	16:30971810:A:G	GTEx/v8	Brain_Nucleus_accumbens_basal_ganglia	ENSG00000103496	G	6.43E-10	0.284854	1.78E-08	A	-	16	30971810	STX4
rs2054213	16:30971810:A:G	GTEx/v8	Brain_Nucleus_accumbens_basal_ganglia	ENSG00000178226	G	6.50E-05	-0.289417	2.67E-19	A	+	16	30971810	PRSS36
rs2054213	16:30971810:A:G	GTEx/v8	Brain_Nucleus_accumbens_basal_ganglia	ENSG00000196118	G	4.40E-05	0.250884	2.99E-15	A	-	16	30971810	C16orf93
rs2054213	16:30971810:A:G	GTEx/v8	Brain_Putamen_basal_ganglia	ENSG00000178226	G	2.14E-06	-0.325655	3.91E-12	A	+	16	30971810	PRSS36
rs78454137	17:43785096:A:G	GTEx/v8	Brain_Amygdala	ENSG00000176681	A	6.33E-07	0.679567	2.49E-20	G	-	17	43785096	LRRC37A
rs78454137	17:43785096:A:G	GTEx/v8	Brain_Amygdala	ENSG00000185829	A	6.19E-10	0.816611	1.19E-06	G	-	17	43785096	ARL17A
rs78454137	17:43785096:A:G	GTEx/v8	Brain_Amygdala	ENSG00000225190	A	1.74E-05	0.337383	0.00225518	G	-	17	43785096	PLEKHM1
rs78454137	17:43785096:A:G	GTEx/v8	Brain_Amygdala	ENSG00000238083	A	9.01E-26	1.10433	7.12E-22	G	-	17	43785096	LRRC37A2
rs78454137	17:43785096:A:G	GTEx/v8	Brain_Anterior_cingulate_cortex_BA24	ENSG00000176681	A	1.22E-07	0.67051	3.11E-25	G	-	17	43785096	LRRC37A
rs78454137	17:43785096:A:G	GTEx/v8	Brain_Anterior_cingulate_cortex_BA24	ENSG00000185829	A	4.60E-13	0.892391	1.04E-09	G	-	17	43785096	ARL17A
rs78454137	17:43785096:A:G	GTEx/v8	Brain_Anterior_cingulate_cortex_BA24	ENSG00000225190	A	6.17E-08	0.386642	9.35E-05	G	-	17	43785096	PLEKHM1
rs78454137	17:43785096:A:G	GTEx/v8	Brain_Anterior_cingulate_cortex_BA24	ENSG00000238083	A	1.16E-29	1.22552	2.21E-23	G	-	17	43785096	LRRC37A2
rs78454137	17:43785096:A:G	GTEx/v8	Brain_Caudate_basal_ganglia	ENSG00000120088	A	6.57E-05	-0.263963	0.0041038	G	+	17	43785096	CRHR1
rs78454137	17:43785096:A:G	GTEx/v8	Brain_Caudate_basal_ganglia	ENSG00000159314	A	4.77E-09	0.240715	4.56E-06	G	-	17	43785096	ARHGAP27
rs78454137	17:43785096:A:G	GTEx/v8	Brain_Caudate_basal_ganglia	ENSG00000176681	A	4.77E-10	0.715212	3.08E-32	G	-	17	43785096	LRRC37A
rs78454137	17:43785096:A:G	GTEx/v8	Brain_Caudate_basal_ganglia	ENSG00000185829	A	2.20E-17	0.856008	8.68E-14	G	-	17	43785096	ARL17A
rs78454137	17:43785096:A:G	GTEx/v8	Brain_Caudate_basal_ganglia	ENSG00000225190	A	4.66E-08	0.313105	3.83E-07	G	-	17	43785096	PLEKHM1
rs78454137	17:43785096:A:G	GTEx/v8	Brain_Caudate_basal_ganglia	ENSG00000238083	A	7.47E-41	1.22199	7.07E-35	G	-	17	43785096	LRRC37A2
rs78454137	17:43785096:A:G	GTEx/v8	Brain_Cerebellar_Hemisphere	ENSG00000120071	A	1.27E-05	0.353543	1.09E-09	G	-	17	43785096	KANSL1
rs78454137	17:43785096:A:G	GTEx/v8	Brain_Cerebellar_Hemisphere	ENSG00000136448	A	7.92E-05	-0.227737	0.00258696	G	+	17	43785096	NMT1
rs78454137	17:43785096:A:G	GTEx/v8	Brain_Cerebellar_Hemisphere	ENSG00000176681	A	2.81E-25	1.12453	1.16E-23	G	-	17	43785096	LRRC37A
rs78454137	17:43785096:A:G	GTEx/v8	Brain_Cerebellar_Hemisphere	ENSG00000184922	A	5.12E-19	-0.640966	1.76E-15	G	+	17	43785096	FMNL1
rs78454137	17:43785096:A:G	GTEx/v8	Brain_Cerebellar_Hemisphere	ENSG00000185294	A	8.27E-20	0.67929	1.39E-15	G	-	17	43785096	SPPL2C
rs78454137	17:43785096:A:G	GTEx/v8	Brain_Cerebellar_Hemisphere	ENSG00000185829	A	8.25E-33	1.10786	8.41E-27	G	-	17	43785096	ARL17A

rs78454137	17:43785096:A:G	GTEx/v8	Brain_Cerebellar_Hemisphere	ENSG00000225190	A	1.33E-27	-0.839202	4.69E-23	G	+	17	43785096	PLEKHM1
rs78454137	17:43785096:A:G	GTEx/v8	Brain_Cerebellar_Hemisphere	ENSG00000238083	A	2.08E-42	1.30629	1.05E-34	G	-	17	43785096	LRRC37A2
rs78454137	17:43785096:A:G	GTEx/v8	Brain_Cerebellum	ENSG00000073969	A	0.000171866	-0.14865	2.14E-10	G	+	17	43785096	NSF
rs78454137	17:43785096:A:G	GTEx/v8	Brain_Cerebellum	ENSG00000120071	A	1.05E-05	0.331017	1.35E-08	G	-	17	43785096	KANSL1
rs78454137	17:43785096:A:G	GTEx/v8	Brain_Cerebellum	ENSG00000176681	A	6.27E-27	1.04178	1.33E-33	G	-	17	43785096	LRRC37A
rs78454137	17:43785096:A:G	GTEx/v8	Brain_Cerebellum	ENSG00000184922	A	1.55E-22	-0.703595	7.89E-19	G	+	17	43785096	FMNL1
rs78454137	17:43785096:A:G	GTEx/v8	Brain_Cerebellum	ENSG00000185294	A	5.47E-24	0.760892	5.44E-19	G	-	17	43785096	SPPL2C
rs78454137	17:43785096:A:G	GTEx/v8	Brain_Cerebellum	ENSG00000185829	A	1.76E-36	1.11953	7.42E-31	G	-	17	43785096	ARL17A
rs78454137	17:43785096:A:G	GTEx/v8	Brain_Cerebellum	ENSG00000186868	A	7.94E-07	-0.285805	7.42E-05	G	+	17	43785096	MAPT
rs78454137	17:43785096:A:G	GTEx/v8	Brain_Cerebellum	ENSG00000225190	A	5.49E-40	-1.0217	8.80E-35	G	+	17	43785096	PLEKHM1
rs78454137	17:43785096:A:G	GTEx/v8	Brain_Cerebellum	ENSG00000238083	A	1.41E-51	1.18601	7.91E-44	G	-	17	43785096	LRRC37A2
rs78454137	17:43785096:A:G	GTEx/v8	Brain_Cortex	ENSG00000159314	A	2.82E-06	0.317929	0.000186356	G	-	17	43785096	ARHGAP27
rs78454137	17:43785096:A:G	GTEx/v8	Brain_Cortex	ENSG00000176681	A	1.09E-13	0.782074	4.87E-42	G	-	17	43785096	LRRC37A
rs78454137	17:43785096:A:G	GTEx/v8	Brain_Cortex	ENSG00000185829	A	8.94E-28	1.06058	8.50E-24	G	-	17	43785096	ARL17A
rs78454137	17:43785096:A:G	GTEx/v8	Brain_Cortex	ENSG00000225190	A	9.21E-14	0.440667	5.25E-12	G	-	17	43785096	PLEKHM1
rs78454137	17:43785096:A:G	GTEx/v8	Brain_Cortex	ENSG00000238083	A	6.76E-42	1.20536	4.81E-35	G	-	17	43785096	LRRC37A2
rs78454137	17:43785096:A:G	GTEx/v8	Brain_Frontal_Cortex_BA9	ENSG00000176681	A	5.06E-11	0.766885	3.82E-31	G	-	17	43785096	LRRC37A
rs78454137	17:43785096:A:G	GTEx/v8	Brain_Frontal_Cortex_BA9	ENSG00000185294	A	9.05E-06	0.3708	0.00224603	G	-	17	43785096	SPPL2C
rs78454137	17:43785096:A:G	GTEx/v8	Brain_Frontal_Cortex_BA9	ENSG00000185829	A	3.18E-26	1.12952	2.13E-20	G	-	17	43785096	ARL17A
rs78454137	17:43785096:A:G	GTEx/v8	Brain_Frontal_Cortex_BA9	ENSG00000225190	A	8.01E-07	0.324298	3.26E-05	G	-	17	43785096	PLEKHM1
rs78454137	17:43785096:A:G	GTEx/v8	Brain_Frontal_Cortex_BA9	ENSG00000238083	A	4.31E-35	1.23549	1.95E-30	G	-	17	43785096	LRRC37A2
rs78454137	17:43785096:A:G	GTEx/v8	Brain_Hippocampus	ENSG00000120088	A	7.45E-05	-0.229731	0.0137363	G	+	17	43785096	CRHR1
rs78454137	17:43785096:A:G	GTEx/v8	Brain_Hippocampus	ENSG00000176681	A	3.17E-09	0.715883	5.43E-23	G	-	17	43785096	LRRC37A
rs78454137	17:43785096:A:G	GTEx/v8	Brain_Hippocampus	ENSG00000185829	A	3.75E-10	0.683399	5.45E-07	G	-	17	43785096	ARL17A
rs78454137	17:43785096:A:G	GTEx/v8	Brain_Hippocampus	ENSG00000238083	A	9.35E-34	1.19171	1.30E-27	G	-	17	43785096	LRRC37A2
rs78454137	17:43785096:A:G	GTEx/v8	Brain_Hypothalamus	ENSG00000176681	A	7.35E-13	0.893178	3.01E-29	G	-	17	43785096	LRRC37A
rs78454137	17:43785096:A:G	GTEx/v8	Brain_Hypothalamus	ENSG00000185829	A	7.94E-21	1.0354	2.17E-16	G	-	17	43785096	ARL17A
rs78454137	17:43785096:A:G	GTEx/v8	Brain_Hypothalamus	ENSG00000238083	A	6.64E-41	1.30353	2.49E-35	G	-	17	43785096	LRRC37A2
rs78454137	17:43785096:A:G	GTEx/v8	Brain_Nucleus_accumbens_basal_ganglia	ENSG00000120088	A	9.48E-05	-0.256662	0.00117547	G	+	17	43785096	CRHR1

rs78454137	17:43785096:A:G	GTEx/v8	Brain_Nucleus_accumbens_basal_ganglia	ENSG00000159314	A	4.06E-18	0.427502	7.23E-14	G	-	17	43785096	ARHGAP27
rs78454137	17:43785096:A:G	GTEx/v8	Brain_Nucleus_accumbens_basal_ganglia	ENSG00000176681	A	7.60E-09	0.651052	2.08E-40	G	-	17	43785096	LRRC37A
rs78454137	17:43785096:A:G	GTEx/v8	Brain_Nucleus_accumbens_basal_ganglia	ENSG00000185829	A	2.55E-23	0.989819	2.05E-18	G	-	17	43785096	ARL17A
rs78454137	17:43785096:A:G	GTEx/v8	Brain_Nucleus_accumbens_basal_ganglia	ENSG00000225190	A	1.55E-13	0.409056	3.43E-13	G	-	17	43785096	PLEKHM1
rs78454137	17:43785096:A:G	GTEx/v8	Brain_Nucleus_accumbens_basal_ganglia	ENSG00000238083	A	1.99E-44	1.20528	4.15E-37	G	-	17	43785096	LRRC37A2
rs78454137	17:43785096:A:G	GTEx/v8	Brain_Putamen_basal_ganglia	ENSG00000176681	A	8.58E-08	0.681975	1.62E-31	G	-	17	43785096	LRRC37A
rs78454137	17:43785096:A:G	GTEx/v8	Brain_Putamen_basal_ganglia	ENSG00000185829	A	2.16E-12	0.776594	8.99E-09	G	-	17	43785096	ARL17A
rs78454137	17:43785096:A:G	GTEx/v8	Brain_Putamen_basal_ganglia	ENSG00000225190	A	2.30E-07	0.338848	7.92E-07	G	-	17	43785096	PLEKHM1
rs78454137	17:43785096:A:G	GTEx/v8	Brain_Putamen_basal_ganglia	ENSG00000238083	A	1.54E-35	1.20334	1.79E-29	G	-	17	43785096	LRRC37A2
rs78454137	17:43785096:A:G	GTEx/v8	Brain_Spinal_cord_cervical_c-1	ENSG00000176681	A	7.19E-10	0.908005	1.10E-10	G	-	17	43785096	LRRC37A
rs78454137	17:43785096:A:G	GTEx/v8	Brain_Spinal_cord_cervical_c-1	ENSG00000238083	A	1.07E-21	1.11361	2.48E-16	G	-	17	43785096	LRRC37A2
rs78454137	17:43785096:A:G	GTEx/v8	Brain_Substantia_nigra	ENSG00000176681	A	2.00E-08	0.914186	1.21E-10	G	-	17	43785096	LRRC37A
rs78454137	17:43785096:A:G	GTEx/v8	Brain_Substantia_nigra	ENSG00000238083	A	2.52E-20	1.24082	3.09E-15	G	-	17	43785096	LRRC37A2

b) PsychENCODE

SNP	uniqID	db	tissue	gene	testedAllele	p	signed_stats	FDR	RiskIncAllele	alignedDirection	chr	pos	symbol
rs2054213	16:30916430:C:G	PsychENCODE	PsychENCODE_eQTLs	ENSG00000099377	G	8.61E-16	-0.145664	3.43E-13	C	+	16	30916430	HSD3B7
rs2054213	16:30916430:C:G	PsychENCODE	PsychENCODE_eQTLs	ENSG00000103510	G	1.30E-10	-0.108188	3.09E-08	C	+	16	30916430	KAT8
rs2054213	16:30916430:C:G	PsychENCODE	PsychENCODE_eQTLs	ENSG00000196118	G	2.13E-06	0.0868658	0.000268432	C	-	16	30916430	C16orf93
rs2054213	16:30916430:C:G	PsychENCODE	PsychENCODE_eQTLs	ENSG00000103496	G	9.12E-06	0.051017	0.001004276	C	-	16	30916430	STX4
rs2054213	16:30916430:C:G	PsychENCODE	PsychENCODE_eQTLs	ENSG00000178226	G	8.34E-05	-0.0894573	0.007190033	C	+	16	30916430	PRSS36
rs2054213	16:30916430:C:G	PsychENCODE	PsychENCODE_eQTLs	ENSG00000177238	G	0.000155278	0.0766764	0.012329485	C	-	16	30916430	TRIM72
rs2054213	16:30916430:C:G	PsychENCODE	PsychENCODE_eQTLs	ENSG00000169592	G	0.00052643	-0.044546	0.034470274	C	+	16	30916430	INO80E
rs2054213	16:30916430:C:G	PsychENCODE	PsychENCODE_eQTLs	ENSG00000103549	G	0.000585528	-0.0540064	0.037603176	C	+	16	30916430	RNF40
rs2054213	16:30923602:A:T	PsychENCODE	PsychENCODE_eQTLs	ENSG00000099377	T	1.49E-15	-0.14392	5.81E-13	A	+	16	30923602	HSD3B7
rs2054213	16:30923602:A:T	PsychENCODE	PsychENCODE_eQTLs	ENSG00000103510	T	1.87E-10	-0.106863	4.36E-08	A	+	16	30923602	KAT8
rs2054213	16:30923602:A:T	PsychENCODE	PsychENCODE_eQTLs	ENSG00000196118	T	1.74E-06	0.0872836	0.000222603	A	-	16	30923602	C16orf93
rs2054213	16:30923602:A:T	PsychENCODE	PsychENCODE_eQTLs	ENSG00000103496	T	1.39E-05	0.0497768	0.001470543	A	-	16	30923602	STX4

rs2054213	16:30923602:A:T	PsychENCODE	PsychENCODE_eQTLs	ENSG00000177238	T	2.75E-05	0.0846298	0.002700547	A	-	16	30923602	TRIM72
rs2054213	16:30923602:A:T	PsychENCODE	PsychENCODE_eQTLs	ENSG00000178226	T	5.97E-05	-0.0909102	0.005361483	A	+	16	30923602	PRSS36
rs2054213	16:30923602:A:T	PsychENCODE	PsychENCODE_eQTLs	ENSG00000103549	T	0.000561321	-0.0539792	0.036336563	A	+	16	30923602	RNF40
rs2054213	16:30923602:A:T	PsychENCODE	PsychENCODE_eQTLs	ENSG00000169592	T	0.000574059	-0.0440793	0.037006817	A	+	16	30923602	INO80E
rs2054213	16:30929444:C:T	PsychENCODE	PsychENCODE_eQTLs	ENSG00000103510	T	3.08E-09	-0.111965	6.14E-07	C	+	16	30929444	KAT8
rs2054213	16:30929444:C:T	PsychENCODE	PsychENCODE_eQTLs	ENSG00000099377	T	2.01E-07	-0.106209	3.05E-05	C	+	16	30929444	HSD3B7
rs2054213	16:30929444:C:T	PsychENCODE	PsychENCODE_eQTLs	ENSG00000177238	T	2.29E-05	0.0961498	0.002298056	C	-	16	30929444	TRIM72
rs2054213	16:30929444:C:T	PsychENCODE	PsychENCODE_eQTLs	ENSG00000169592	T	0.000228755	-0.0530572	0.017176934	C	+	16	30929444	INO80E
rs2054213	16:30929444:C:T	PsychENCODE	PsychENCODE_eQTLs	ENSG00000178226	T	0.000603086	-0.0874841	0.038516765	C	+	16	30929444	PRSS36
rs2054213	16:30931968:C:T	PsychENCODE	PsychENCODE_eQTLs	ENSG00000099377	T	7.82E-18	-0.156369	3.67E-15	C	+	16	30931968	HSD3B7
rs2054213	16:30931968:C:T	PsychENCODE	PsychENCODE_eQTLs	ENSG00000103510	T	3.88E-10	-0.105995	8.68E-08	C	+	16	30931968	KAT8
rs2054213	16:30931968:C:T	PsychENCODE	PsychENCODE_eQTLs	ENSG00000196118	T	2.44E-05	0.0778391	0.002427595	C	-	16	30931968	C16orf93
rs2054213	16:30931968:C:T	PsychENCODE	PsychENCODE_eQTLs	ENSG00000103496	T	3.58E-05	0.0478102	0.003415319	C	-	16	30931968	STX4
rs2054213	16:30931968:C:T	PsychENCODE	PsychENCODE_eQTLs	ENSG00000177238	T	4.68E-05	0.0829633	0.004322085	C	-	16	30931968	TRIM72
rs2054213	16:30931968:C:T	PsychENCODE	PsychENCODE_eQTLs	ENSG00000178226	T	0.000106582	-0.0886158	0.008903563	C	+	16	30931968	PRSS36
rs2054213	16:30931968:C:T	PsychENCODE	PsychENCODE_eQTLs	ENSG00000169592	T	0.000547702	-0.0446607	0.035611163	C	+	16	30931968	INO80E
rs2054213	16:30943096:A:G	PsychENCODE	PsychENCODE_eQTLs	ENSG00000099377	G	3.80E-18	-0.157854	1.83E-15	A	+	16	30943096	HSD3B7
rs2054213	16:30943096:A:G	PsychENCODE	PsychENCODE_eQTLs	ENSG00000103510	G	3.45E-10	-0.106312	7.78E-08	A	+	16	30943096	KAT8
rs2054213	16:30943096:A:G	PsychENCODE	PsychENCODE_eQTLs	ENSG00000196118	G	2.21E-05	0.0782606	0.002221973	A	-	16	30943096	C16orf93
rs2054213	16:30943096:A:G	PsychENCODE	PsychENCODE_eQTLs	ENSG00000177238	G	3.41E-05	0.0844484	0.00327252	A	-	16	30943096	TRIM72
rs2054213	16:30943096:A:G	PsychENCODE	PsychENCODE_eQTLs	ENSG00000103496	G	3.59E-05	0.0478106	0.003423594	A	-	16	30943096	STX4
rs2054213	16:30943096:A:G	PsychENCODE	PsychENCODE_eQTLs	ENSG00000178226	G	9.13E-05	-0.089484	0.007778727	A	+	16	30943096	PRSS36
rs2054213	16:30943096:A:G	PsychENCODE	PsychENCODE_eQTLs	ENSG00000169592	G	0.000636152	-0.0441459	0.040221325	A	+	16	30943096	INO80E
rs2054213	16:30944248:A:G	PsychENCODE	PsychENCODE_eQTLs	ENSG00000099377	G	4.20E-18	-0.157973	2.01E-15	A	+	16	30944248	HSD3B7
rs2054213	16:30944248:A:G	PsychENCODE	PsychENCODE_eQTLs	ENSG00000103510	G	2.72E-10	-0.107149	6.21E-08	A	+	16	30944248	KAT8
rs2054213	16:30944248:A:G	PsychENCODE	PsychENCODE_eQTLs	ENSG00000196118	G	2.25E-05	0.0783422	0.002260217	A	-	16	30944248	C16orf93
rs2054213	16:30944248:A:G	PsychENCODE	PsychENCODE_eQTLs	ENSG00000103496	G	3.66E-05	0.0478617	0.003477334	A	-	16	30944248	STX4
rs2054213	16:30944248:A:G	PsychENCODE	PsychENCODE_eQTLs	ENSG00000177238	G	6.17E-05	0.0818294	0.00551378	A	-	16	30944248	TRIM72
rs2054213	16:30944248:A:G	PsychENCODE	PsychENCODE_eQTLs	ENSG00000178226	G	0.000110491	-0.0886112	0.009185486	A	+	16	30944248	PRSS36

rs2054213	16:30944248:A:G	PsychENCODE	PsychENCODE_eQTLs	ENSG00000169592	G	0.000575167	-0.0445889	0.03706487	A	+	16	30944248	INO80E
rs2054213	16:30945861:C:T	PsychENCODE	PsychENCODE_eQTLs	ENSG00000099377	C	4.20E-18	-0.157977	2.01E-15	T	+	16	30945861	HSD3B7
rs2054213	16:30945861:C:T	PsychENCODE	PsychENCODE_eQTLs	ENSG00000103510	C	2.73E-10	-0.107137	6.24E-08	T	+	16	30945861	KAT8
rs2054213	16:30945861:C:T	PsychENCODE	PsychENCODE_eQTLs	ENSG00000196118	C	2.25E-05	0.0783439	0.002259942	T	-	16	30945861	C16orf93
rs2054213	16:30945861:C:T	PsychENCODE	PsychENCODE_eQTLs	ENSG00000103496	C	3.66E-05	0.0478596	0.003480607	T	-	16	30945861	STX4
rs2054213	16:30945861:C:T	PsychENCODE	PsychENCODE_eQTLs	ENSG00000177238	C	6.18E-05	0.0818191	0.005525428	T	-	16	30945861	TRIM72
rs2054213	16:30945861:C:T	PsychENCODE	PsychENCODE_eQTLs	ENSG00000178226	C	0.000110412	-0.0886165	0.009179834	T	+	16	30945861	PRSS36
rs2054213	16:30945861:C:T	PsychENCODE	PsychENCODE_eQTLs	ENSG00000169592	C	0.000574639	-0.0445928	0.037037697	T	+	16	30945861	INO80E
rs2054213	16:30949047:A:G	PsychENCODE	PsychENCODE_eQTLs	ENSG00000099377	G	3.78E-18	-0.157868	1.82E-15	A	+	16	30949047	HSD3B7
rs2054213	16:30949047:A:G	PsychENCODE	PsychENCODE_eQTLs	ENSG00000103510	G	3.50E-10	-0.106282	7.88E-08	A	+	16	30949047	KAT8
rs2054213	16:30949047:A:G	PsychENCODE	PsychENCODE_eQTLs	ENSG00000196118	G	2.22E-05	0.0782374	0.002234263	A	-	16	30949047	C16orf93
rs2054213	16:30949047:A:G	PsychENCODE	PsychENCODE_eQTLs	ENSG00000177238	G	3.42E-05	0.0844486	0.003273725	A	-	16	30949047	TRIM72
rs2054213	16:30949047:A:G	PsychENCODE	PsychENCODE_eQTLs	ENSG00000103496	G	3.56E-05	0.0478349	0.003397233	A	-	16	30949047	STX4
rs2054213	16:30949047:A:G	PsychENCODE	PsychENCODE_eQTLs	ENSG00000178226	G	9.06E-05	-0.08953	0.007724801	A	+	16	30949047	PRSS36
rs2054213	16:30949047:A:G	PsychENCODE	PsychENCODE_eQTLs	ENSG00000169592	G	0.00063549	-0.0441507	0.040187047	A	+	16	30949047	INO80E
rs2054213	16:30969557:C:G	PsychENCODE	PsychENCODE_eQTLs	ENSG00000099377	C	5.48E-18	-0.156849	2.60E-15	G	+	16	30969557	HSD3B7
rs2054213	16:30969557:C:G	PsychENCODE	PsychENCODE_eQTLs	ENSG00000103510	C	1.82E-10	-0.107773	4.26E-08	G	+	16	30969557	KAT8
rs2054213	16:30969557:C:G	PsychENCODE	PsychENCODE_eQTLs	ENSG00000103496	C	1.74E-05	0.0496013	0.001798977	G	-	16	30969557	STX4
rs2054213	16:30969557:C:G	PsychENCODE	PsychENCODE_eQTLs	ENSG00000177238	C	4.24E-05	0.0832995	0.003960392	G	-	16	30969557	TRIM72
rs2054213	16:30969557:C:G	PsychENCODE	PsychENCODE_eQTLs	ENSG00000196118	C	7.75E-05	0.0728088	0.006737245	G	-	16	30969557	C16orf93
rs2054213	16:30969557:C:G	PsychENCODE	PsychENCODE_eQTLs	ENSG00000178226	C	0.000120251	-0.0878095	0.009884317	G	+	16	30969557	PRSS36
rs2054213	16:30969557:C:G	PsychENCODE	PsychENCODE_eQTLs	ENSG00000169592	C	0.000618402	-0.0441696	0.039309118	G	+	16	30969557	INO80E
rs2054213	16:30980054:A:G	PsychENCODE	PsychENCODE_eQTLs	ENSG00000099377	G	3.74E-18	-0.157882	1.80E-15	A	+	16	30980054	HSD3B7
rs2054213	16:30980054:A:G	PsychENCODE	PsychENCODE_eQTLs	ENSG00000103510	G	1.09E-10	-0.109265	2.61E-08	A	+	16	30980054	KAT8
rs2054213	16:30980054:A:G	PsychENCODE	PsychENCODE_eQTLs	ENSG00000103496	G	4.32E-05	0.0473231	0.004026999	A	-	16	30980054	STX4
rs2054213	16:30980054:A:G	PsychENCODE	PsychENCODE_eQTLs	ENSG00000177238	G	5.90E-05	0.0818736	0.005303008	A	-	16	30980054	TRIM72
rs2054213	16:30980054:A:G	PsychENCODE	PsychENCODE_eQTLs	ENSG00000196118	G	9.22E-05	0.0721636	0.00784612	A	-	16	30980054	C16orf93
rs2054213	16:30980054:A:G	PsychENCODE	PsychENCODE_eQTLs	ENSG00000178226	G	0.000112093	-0.0883492	0.009301317	A	+	16	30980054	PRSS36
rs2054213	16:30980054:A:G	PsychENCODE	PsychENCODE_eQTLs	ENSG00000167394	G	0.000404252	0.0596082	0.027730214	A	-	16	30980054	ZNF668

rs2054213	16:30980054:A:G	PsychENCODE	PsychENCODE_eQTLs	ENSG00000167397	G	0.000580439	0.0371026	0.037340372	A	-	16	30980054	VKORC1
rs2054213	16:30980054:A:G	PsychENCODE	PsychENCODE_eQTLs	ENSG00000169592	G	0.000773406	-0.0434558	0.047096911	A	+	16	30980054	INO80E
rs2054213	16:30981544:C:G	PsychENCODE	PsychENCODE_eQTLs	ENSG00000099377	G	3.20E-18	-0.158109	1.55E-15	C	+	16	30981544	HSD3B7
rs2054213	16:30981544:C:G	PsychENCODE	PsychENCODE_eQTLs	ENSG00000103510	G	1.12E-10	-0.109136	2.68E-08	C	+	16	30981544	KAT8
rs2054213	16:30981544:C:G	PsychENCODE	PsychENCODE_eQTLs	ENSG00000103496	G	4.34E-05	0.0472796	0.004049093	C	-	16	30981544	STX4
rs2054213	16:30981544:C:G	PsychENCODE	PsychENCODE_eQTLs	ENSG00000177238	G	6.19E-05	0.0815974	0.005531263	C	-	16	30981544	TRIM72
rs2054213	16:30981544:C:G	PsychENCODE	PsychENCODE_eQTLs	ENSG00000196118	G	0.000109913	0.0713397	0.009143642	C	-	16	30981544	C16orf93
rs2054213	16:30981544:C:G	PsychENCODE	PsychENCODE_eQTLs	ENSG00000178226	G	0.000111429	-0.0883319	0.009253205	C	+	16	30981544	PRSS36
rs2054213	16:30981544:C:G	PsychENCODE	PsychENCODE_eQTLs	ENSG00000167394	G	0.000372763	0.0599324	0.025913479	C	-	16	30981544	ZNF668
rs2054213	16:30981544:C:G	PsychENCODE	PsychENCODE_eQTLs	ENSG00000167397	G	0.000569805	0.0371351	0.036781769	C	-	16	30981544	VKORC1
rs2054213	16:30981544:C:G	PsychENCODE	PsychENCODE_eQTLs	ENSG00000169592	G	0.000827695	-0.0431895	0.049730692	C	+	16	30981544	INO80E
rs2054213	16:30983260:A:C	PsychENCODE	PsychENCODE_eQTLs	ENSG00000099377	C	4.28E-18	-0.157903	2.05E-15	A	+	16	30983260	HSD3B7
rs2054213	16:30983260:A:C	PsychENCODE	PsychENCODE_eQTLs	ENSG00000103510	C	7.72E-11	-0.110333	1.89E-08	A	+	16	30983260	KAT8
rs2054213	16:30983260:A:C	PsychENCODE	PsychENCODE_eQTLs	ENSG00000103496	C	4.06E-05	0.0475766	0.003810311	A	-	16	30983260	STX4
rs2054213	16:30983260:A:C	PsychENCODE	PsychENCODE_eQTLs	ENSG00000196118	C	9.28E-05	0.0722686	0.007888742	A	-	16	30983260	C16orf93
rs2054213	16:30983260:A:C	PsychENCODE	PsychENCODE_eQTLs	ENSG00000177238	C	0.000118016	0.0786393	0.009724742	A	-	16	30983260	TRIM72
rs2054213	16:30983260:A:C	PsychENCODE	PsychENCODE_eQTLs	ENSG00000178226	C	0.000130834	-0.0876473	0.010633582	A	+	16	30983260	PRSS36
rs2054213	16:30983260:A:C	PsychENCODE	PsychENCODE_eQTLs	ENSG00000167394	C	0.000347461	0.0603863	0.024435564	A	-	16	30983260	ZNF668
rs2054213	16:30983260:A:C	PsychENCODE	PsychENCODE_eQTLs	ENSG00000167397	C	0.000424297	0.0380731	0.028867369	A	-	16	30983260	VKORC1
rs2054213	16:30983260:A:C	PsychENCODE	PsychENCODE_eQTLs	ENSG00000169592	C	0.000706387	-0.0438574	0.043779286	A	+	16	30983260	INO80E
rs2054213	16:30996872:C:G	PsychENCODE	PsychENCODE_eQTLs	ENSG00000103510	C	3.69E-10	-0.116108	8.28E-08	G	+	16	30996872	KAT8
rs2054213	16:30996872:C:G	PsychENCODE	PsychENCODE_eQTLs	ENSG00000099377	C	7.66E-09	-0.115708	1.45E-06	G	+	16	30996872	HSD3B7
rs2054213	16:30996872:C:G	PsychENCODE	PsychENCODE_eQTLs	ENSG00000178226	C	0.000135942	-0.0954677	0.010993309	G	+	16	30996872	PRSS36
rs2054213	16:30996872:C:G	PsychENCODE	PsychENCODE_eQTLs	ENSG00000177238	C	0.000178771	0.0835829	0.013913802	G	-	16	30996872	TRIM72
rs2054213	16:30996872:C:G	PsychENCODE	PsychENCODE_eQTLs	ENSG00000169592	C	0.000343708	-0.0506022	0.024214165	G	+	16	30996872	INO80E
rs2054213	16:30996872:C:G	PsychENCODE	PsychENCODE_eQTLs	ENSG00000103496	C	0.000718765	0.0428526	0.044397195	G	-	16	30996872	STX4
rs2054213	16:31000809:A:G	PsychENCODE	PsychENCODE_eQTLs	ENSG00000099377	A	2.57E-18	-0.158998	1.25E-15	G	+	16	31000809	HSD3B7
rs2054213	16:31000809:A:G	PsychENCODE	PsychENCODE_eQTLs	ENSG00000103510	A	7.17E-11	-0.110566	1.76E-08	G	+	16	31000809	KAT8
rs2054213	16:31000809:A:G	PsychENCODE	PsychENCODE_eQTLs	ENSG00000103496	A	1.11E-05	0.0509438	0.001197585	G	-	16	31000809	STX4

rs2054213	16:31000809:A:G	PsychENCODE	PsychENCODE_eQTLs	ENSG00000196118	A	9.83E-05	0.0720428	0.008295956	G	-	16	31000809	C16orf93
rs2054213	16:31000809:A:G	PsychENCODE	PsychENCODE_eQTLs	ENSG00000177238	A	0.000107115	0.0791544	0.008941905	G	-	16	31000809	TRIM72
rs2054213	16:31000809:A:G	PsychENCODE	PsychENCODE_eQTLs	ENSG00000178226	A	0.000159156	-0.0865778	0.012593204	G	+	16	31000809	PRSS36
rs2054213	16:31000809:A:G	PsychENCODE	PsychENCODE_eQTLs	ENSG00000169592	A	0.000458805	-0.0453806	0.030790692	G	+	16	31000809	INO80E
rs10503002	18:53121576:C:G	PsychENCODE	PsychENCODE_eQTLs	ENSG00000196628	C	0.000811406	-0.0324159	0.048945667	G	+	18	53121576	TCF4
rs13037664	20:32987687:C:T	PsychENCODE	PsychENCODE_eQTLs	ENSG00000198646	C	1.26E-13	0.165862	4.12E-11	T	-	20	32987687	NCOA6
rs13037664	20:32987687:C:T	PsychENCODE	PsychENCODE_eQTLs	ENSG00000088298	C	0.000707919	0.105751	0.043855992	T	-	20	32987687	EDEM2
rs13037664	20:32994629:C:T	PsychENCODE	PsychENCODE_eQTLs	ENSG00000198646	T	1.77E-13	0.164519	5.71E-11	C	-	20	32994629	NCOA6
rs13037664	20:32994629:C:T	PsychENCODE	PsychENCODE_eQTLs	ENSG00000088298	T	0.000532692	0.107922	0.034805515	C	-	20	32994629	EDEM2
rs13037664	20:33140999:A:G	PsychENCODE	PsychENCODE_eQTLs	ENSG00000198646	A	1.20E-20	-0.157136	6.92E-18	A	-	20	33140999	NCOA6
rs13037664	20:33145404:C:G	PsychENCODE	PsychENCODE_eQTLs	ENSG00000198646	G	2.11E-19	-0.146457	1.12E-16	G	-	20	33145404	NCOA6
rs13037664	20:33145470:C:T	PsychENCODE	PsychENCODE_eQTLs	ENSG00000198646	T	1.16E-19	-0.147427	6.23E-17	T	-	20	33145470	NCOA6
rs13037664	20:33147737:C:G	PsychENCODE	PsychENCODE_eQTLs	ENSG00000198646	G	2.45E-20	-0.155899	1.38E-17	G	-	20	33147737	NCOA6
rs13037664	20:33158318:A:G	PsychENCODE	PsychENCODE_eQTLs	ENSG00000198646	A	1.91E-20	-0.161679	1.09E-17	A	-	20	33158318	NCOA6
rs13037664	20:33162298:C:G	PsychENCODE	PsychENCODE_eQTLs	ENSG00000198646	C	2.27E-19	-0.146415	1.20E-16	C	-	20	33162298	NCOA6
rs13037664	20:33170689:A:G	PsychENCODE	PsychENCODE_eQTLs	ENSG00000198646	A	1.91E-19	-0.152358	1.01E-16	A	-	20	33170689	NCOA6
rs13037664	20:33171368:C:G	PsychENCODE	PsychENCODE_eQTLs	ENSG00000198646	C	1.54E-20	-0.161923	8.83E-18	C	-	20	33171368	NCOA6
rs13037664	20:33175338:A:G	PsychENCODE	PsychENCODE_eQTLs	ENSG00000198646	G	1.54E-20	-0.161877	8.81E-18	G	-	20	33175338	NCOA6
rs13037664	20:33179788:A:G	PsychENCODE	PsychENCODE_eQTLs	ENSG00000198646	G	4.78E-19	-0.149415	2.47E-16	G	-	20	33179788	NCOA6
rs13037664	20:33182556:C:T	PsychENCODE	PsychENCODE_eQTLs	ENSG00000198646	C	4.88E-19	-0.149368	2.52E-16	C	-	20	33182556	NCOA6
rs13037664	20:33182577:A:G	PsychENCODE	PsychENCODE_eQTLs	ENSG00000198646	A	1.49E-19	-0.147462	7.99E-17	A	-	20	33182577	NCOA6
rs13037664	20:33186200:C:G	PsychENCODE	PsychENCODE_eQTLs	ENSG00000198646	C	3.06E-19	-0.151303	1.60E-16	C	-	20	33186200	NCOA6
rs13037664	20:33197306:C:T	PsychENCODE	PsychENCODE_eQTLs	ENSG00000198646	T	1.39E-20	-0.161708	7.97E-18	T	-	20	33197306	NCOA6
rs13037664	20:33198390:C:T	PsychENCODE	PsychENCODE_eQTLs	ENSG00000198646	C	1.39E-20	-0.161687	7.98E-18	C	-	20	33198390	NCOA6
rs13037664	20:33205011:C:T	PsychENCODE	PsychENCODE_eQTLs	ENSG00000198646	T	7.90E-20	-0.161492	4.30E-17	T	-	20	33205011	NCOA6
rs13037664	20:33208547:A:G	PsychENCODE	PsychENCODE_eQTLs	ENSG00000198646	G	1.41E-20	-0.161647	8.09E-18	G	-	20	33208547	NCOA6
rs13037664	20:33249134:C:T	PsychENCODE	PsychENCODE_eQTLs	ENSG00000198646	T	9.34E-21	-0.1626	5.44E-18	T	-	20	33249134	NCOA6
rs13037664	20:33250360:C:G	PsychENCODE	PsychENCODE_eQTLs	ENSG00000198646	G	9.14E-21	-0.162636	5.32E-18	G	-	20	33250360	NCOA6
rs13037664	20:33252298:C:T	PsychENCODE	PsychENCODE_eQTLs	ENSG00000198646	T	1.02E-20	-0.162339	5.94E-18	T	-	20	33252298	NCOA6

rs13037664	20:33284624:A:G	PsychENCODE	PsychENCODE_eQTLs	ENSG00000198646	G	3.02E-20	-0.159806	1.69E-17	G	-	20	33284624	NCOA6
rs13037664	20:33284624:A:G	PsychENCODE	PsychENCODE_eQTLs	ENSG00000100983	G	0.000613119	-0.0487682	0.039036484	G	-	20	33284624	GSS
rs13037664	20:33303974:G:T	PsychENCODE	PsychENCODE_eQTLs	ENSG00000198646	G	1.68E-21	-0.162292	1.04E-18	G	-	20	33303974	NCOA6
rs13037664	20:33303974:G:T	PsychENCODE	PsychENCODE_eQTLs	ENSG00000100983	G	8.44E-05	-0.0550427	0.007260271	G	-	20	33303974	GSS
rs13037664	20:33303974:G:T	PsychENCODE	PsychENCODE_eQTLs	ENSG00000088298	G	0.000715749	-0.0809646	0.044246476	G	-	20	33303974	EDEM2
rs13037664	20:33310471:A:G	PsychENCODE	PsychENCODE_eQTLs	ENSG00000198646	G	1.86E-21	-0.161827	1.14E-18	G	-	20	33310471	NCOA6
rs13037664	20:33310471:A:G	PsychENCODE	PsychENCODE_eQTLs	ENSG00000100983	G	6.87E-05	-0.0556211	0.006067431	G	-	20	33310471	GSS
rs13037664	20:33310471:A:G	PsychENCODE	PsychENCODE_eQTLs	ENSG00000088298	G	0.000763965	-0.0803895	0.046634287	G	-	20	33310471	EDEM2
rs13037664	20:33312646:A:G	PsychENCODE	PsychENCODE_eQTLs	ENSG00000198646	G	2.74E-21	-0.161085	1.66E-18	G	-	20	33312646	NCOA6
rs13037664	20:33312646:A:G	PsychENCODE	PsychENCODE_eQTLs	ENSG00000100983	G	6.42E-05	-0.0558195	0.005716151	G	-	20	33312646	GSS
rs13037664	20:33312646:A:G	PsychENCODE	PsychENCODE_eQTLs	ENSG00000088298	G	0.000832376	-0.0797881	0.049954245	G	-	20	33312646	EDEM2
rs13037664	20:33322006:C:G	PsychENCODE	PsychENCODE_eQTLs	ENSG00000198646	C	1.15E-21	-0.164146	7.15E-19	C	-	20	33322006	NCOA6
rs13037664	20:33322006:C:G	PsychENCODE	PsychENCODE_eQTLs	ENSG00000100983	C	3.43E-05	-0.0584101	0.00328418	C	-	20	33322006	GSS
rs13037664	20:33322006:C:G	PsychENCODE	PsychENCODE_eQTLs	ENSG00000088298	C	0.000717729	-0.0815424	0.044345339	C	-	20	33322006	EDEM2
rs13037664	20:33322119:C:T	PsychENCODE	PsychENCODE_eQTLs	ENSG00000198646	C	1.15E-21	-0.164148	7.13E-19	C	-	20	33322119	NCOA6
rs13037664	20:33322119:C:T	PsychENCODE	PsychENCODE_eQTLs	ENSG00000100983	C	3.41E-05	-0.0584235	0.003270904	C	-	20	33322119	GSS
rs13037664	20:33322119:C:T	PsychENCODE	PsychENCODE_eQTLs	ENSG00000088298	C	0.000718324	-0.0815352	0.044375075	C	-	20	33322119	EDEM2
rs13037664	20:33340993:C:T	PsychENCODE	PsychENCODE_eQTLs	ENSG00000198646	T	1.52E-21	-0.161756	9.37E-19	T	-	20	33340993	NCOA6
rs13037664	20:33340993:C:T	PsychENCODE	PsychENCODE_eQTLs	ENSG00000100983	T	5.40E-05	-0.0562668	0.004909232	T	-	20	33340993	GSS
rs13037664	20:33340993:C:T	PsychENCODE	PsychENCODE_eQTLs	ENSG00000088298	T	0.000734165	-0.0804437	0.045159956	T	-	20	33340993	EDEM2
rs13037664	20:33358397:G:T	PsychENCODE	PsychENCODE_eQTLs	ENSG00000198646	G	1.26E-21	-0.162098	7.80E-19	G	-	20	33358397	NCOA6
rs13037664	20:33358397:G:T	PsychENCODE	PsychENCODE_eQTLs	ENSG00000100983	G	5.61E-05	-0.0561541	0.005070417	G	-	20	33358397	GSS
rs13037664	20:33358397:G:T	PsychENCODE	PsychENCODE_eQTLs	ENSG00000088298	G	0.000742729	-0.0803773	0.045585279	G	-	20	33358397	EDEM2
rs13037664	20:33359054:C:G	PsychENCODE	PsychENCODE_eQTLs	ENSG00000198646	C	4.00E-21	-0.16189	2.40E-18	C	-	20	33359054	NCOA6
rs13037664	20:33359054:C:G	PsychENCODE	PsychENCODE_eQTLs	ENSG00000100983	C	3.82E-05	-0.0580296	0.003610949	C	-	20	33359054	GSS
rs13037664	20:33359054:C:G	PsychENCODE	PsychENCODE_eQTLs	ENSG00000088298	C	0.000430901	-0.0847963	0.029239464	C	-	20	33359054	EDEM2
rs13037664	20:33361721:A:G	PsychENCODE	PsychENCODE_eQTLs	ENSG00000198646	G	4.52E-21	-0.16172	2.70E-18	G	-	20	33361721	NCOA6
rs13037664	20:33361721:A:G	PsychENCODE	PsychENCODE_eQTLs	ENSG00000100983	G	3.73E-05	-0.0581185	0.003538999	G	-	20	33361721	GSS
rs13037664	20:33361721:A:G	PsychENCODE	PsychENCODE_eQTLs	ENSG00000088298	G	0.000423016	-0.0849359	0.028795907	G	-	20	33361721	EDEM2

rs13037664	20:33364852:A:G	PsychENCODE	PsychENCODE_eQTLs	ENSG00000198646	G	1.47E-21	-0.161848	9.10E-19	G	-	20	33364852	NCOA6
rs13037664	20:33364852:A:G	PsychENCODE	PsychENCODE_eQTLs	ENSG00000100983	G	5.41E-05	-0.056274	0.004918306	G	-	20	33364852	GSS
rs13037664	20:33364852:A:G	PsychENCODE	PsychENCODE_eQTLs	ENSG00000088298	G	0.000732477	-0.0804786	0.045077805	G	-	20	33364852	EDEM2
rs13037664	20:33366905:A:G	PsychENCODE	PsychENCODE_eQTLs	ENSG00000198646	G	1.54E-21	-0.161769	9.52E-19	G	-	20	33366905	NCOA6
rs13037664	20:33366905:A:G	PsychENCODE	PsychENCODE_eQTLs	ENSG00000100983	G	5.39E-05	-0.0562898	0.004897094	G	-	20	33366905	GSS
rs13037664	20:33366905:A:G	PsychENCODE	PsychENCODE_eQTLs	ENSG00000088298	G	0.000731916	-0.0804835	0.045050167	G	-	20	33366905	EDEM2
rs13037664	20:33370945:A:G	PsychENCODE	PsychENCODE_eQTLs	ENSG00000198646	G	1.90E-21	-0.161441	1.16E-18	G	-	20	33370945	NCOA6
rs13037664	20:33370945:A:G	PsychENCODE	PsychENCODE_eQTLs	ENSG00000100983	G	5.10E-05	-0.0564746	0.004668458	G	-	20	33370945	GSS
rs13037664	20:33370945:A:G	PsychENCODE	PsychENCODE_eQTLs	ENSG00000088298	G	0.000751679	-0.080322	0.046028475	G	-	20	33370945	EDEM2
rs13037664	20:33374897:A:G	PsychENCODE	PsychENCODE_eQTLs	ENSG00000198646	A	1.39E-21	-0.161947	8.60E-19	A	-	20	33374897	NCOA6
rs13037664	20:33374897:A:G	PsychENCODE	PsychENCODE_eQTLs	ENSG00000100983	A	5.37E-05	-0.0562994	0.004884545	A	-	20	33374897	GSS
rs13037664	20:33374897:A:G	PsychENCODE	PsychENCODE_eQTLs	ENSG00000088298	A	0.000717991	-0.0806089	0.044358671	A	-	20	33374897	EDEM2
rs13037664	20:33385552:A:G	PsychENCODE	PsychENCODE_eQTLs	ENSG00000198646	A	1.52E-21	0.161786	9.35E-19	G	-	20	33385552	NCOA6
rs13037664	20:33385552:A:G	PsychENCODE	PsychENCODE_eQTLs	ENSG00000100983	A	5.63E-05	0.0561444	0.005086585	G	-	20	33385552	GSS
rs13037664	20:33385552:A:G	PsychENCODE	PsychENCODE_eQTLs	ENSG00000088298	A	0.000741111	0.0803946	0.045504791	G	-	20	33385552	EDEM2
rs13037664	20:33386156:C:T	PsychENCODE	PsychENCODE_eQTLs	ENSG00000198646	T	1.52E-21	0.16178	9.39E-19	C	-	20	33386156	NCOA6
rs13037664	20:33386156:C:T	PsychENCODE	PsychENCODE_eQTLs	ENSG00000100983	T	5.63E-05	0.0561401	0.005093239	C	-	20	33386156	GSS
rs13037664	20:33386156:C:T	PsychENCODE	PsychENCODE_eQTLs	ENSG00000088298	T	0.000741697	0.0803902	0.045534157	C	-	20	33386156	EDEM2
rs13037664	20:33392739:C:T	PsychENCODE	PsychENCODE_eQTLs	ENSG00000198646	T	1.51E-21	0.161814	9.34E-19	C	-	20	33392739	NCOA6
rs13037664	20:33392739:C:T	PsychENCODE	PsychENCODE_eQTLs	ENSG00000100983	T	5.67E-05	0.05613	0.005119264	C	-	20	33392739	GSS
rs13037664	20:33392739:C:T	PsychENCODE	PsychENCODE_eQTLs	ENSG00000088298	T	0.000751539	0.0803165	0.046021491	C	-	20	33392739	EDEM2
rs13037664	20:33398508:A:G	PsychENCODE	PsychENCODE_eQTLs	ENSG00000198646	A	6.40E-18	0.1605	3.02E-15	G	-	20	33398508	NCOA6
rs13037664	20:33398508:A:G	PsychENCODE	PsychENCODE_eQTLs	ENSG00000088298	A	0.000114718	0.100425	0.009488988	G	-	20	33398508	EDEM2
rs13037664	20:33400355:C:G	PsychENCODE	PsychENCODE_eQTLs	ENSG00000198646	G	3.78E-21	0.162038	2.27E-18	C	-	20	33400355	NCOA6
rs13037664	20:33400355:C:G	PsychENCODE	PsychENCODE_eQTLs	ENSG00000100983	G	3.83E-05	0.0580325	0.003624847	C	-	20	33400355	GSS
rs13037664	20:33400355:C:G	PsychENCODE	PsychENCODE_eQTLs	ENSG00000088298	G	0.000493158	0.0839584	0.032674297	C	-	20	33400355	EDEM2
rs13037664	20:33400379:A:T	PsychENCODE	PsychENCODE_eQTLs	ENSG00000198646	T	3.78E-21	0.162039	2.27E-18	A	-	20	33400379	NCOA6
rs13037664	20:33400379:A:T	PsychENCODE	PsychENCODE_eQTLs	ENSG00000100983	T	3.83E-05	0.0580335	0.003624342	A	-	20	33400379	GSS
rs13037664	20:33400379:A:T	PsychENCODE	PsychENCODE_eQTLs	ENSG00000088298	T	0.000493201	0.0839586	0.032676521	A	-	20	33400379	EDEM2

rs13037664	20:33405956:G:T	PsychENCODE	PsychENCODE_eQTLs	ENSG00000198646	T	1.41E-21	0.161985	8.73E-19	G	-	20	33405956	NCOA6
rs13037664	20:33405956:G:T	PsychENCODE	PsychENCODE_eQTLs	ENSG00000100983	T	6.22E-05	0.0558447	0.005554891	G	-	20	33405956	GSS
rs13037664	20:33405956:G:T	PsychENCODE	PsychENCODE_eQTLs	ENSG00000088298	T	0.000782823	0.0800753	0.047558221	G	-	20	33405956	EDEM2
rs13037664	20:33411048:A:C	PsychENCODE	PsychENCODE_eQTLs	ENSG00000198646	A	1.46E-21	0.161407	9.04E-19	C	-	20	33411048	NCOA6
rs13037664	20:33411048:A:C	PsychENCODE	PsychENCODE_eQTLs	ENSG00000100983	A	7.64E-05	0.0549879	0.006658867	C	-	20	33411048	GSS
rs13037664	20:33417814:A:C	PsychENCODE	PsychENCODE_eQTLs	ENSG00000198646	A	1.79E-21	0.162178	1.10E-18	C	-	20	33417814	NCOA6
rs13037664	20:33417814:A:C	PsychENCODE	PsychENCODE_eQTLs	ENSG00000100983	A	2.43E-05	0.0590745	0.002415018	C	-	20	33417814	GSS
rs13037664	20:33447162:A:G	PsychENCODE	PsychENCODE_eQTLs	ENSG00000198646	A	5.36E-22	0.164101	3.41E-19	G	-	20	33447162	NCOA6
rs13037664	20:33447162:A:G	PsychENCODE	PsychENCODE_eQTLs	ENSG00000100983	A	8.50E-05	0.0549721	0.007306104	G	-	20	33447162	GSS
rs13037664	20:33496471:C:T	PsychENCODE	PsychENCODE_eQTLs	ENSG00000198646	T	1.27E-21	0.162655	7.88E-19	C	-	20	33496471	NCOA6
rs13037664	20:33496471:C:T	PsychENCODE	PsychENCODE_eQTLs	ENSG00000100983	T	8.39E-05	0.0550206	0.007225435	C	-	20	33496471	GSS

Abbreviation. SNP, single nucleotide polymorphism; uniqID, unique ID of SNPs consisting of chr:position:allele1:allele2 where alleles are alphabetically ordered; db, data source of eQTLs; tissue, tissue type; gene, ENSG ID; testedAllele, tested allele obtained from the input GWAS summary statistics; p, P-value of eQTLs; signed_stats, signed statistics, the actual value depends on the data source; FDR, FDR of eQTLs; RiskIncAllele, risk increasing allele obtained from the input GWAS summary statistics; alignedDirection, the direction of effect to gene expression after aligning risk increasing allele of GWAS and tested allele of eQTLs; chr, chromosome; pos, position on hg19; symbol, gene symbol; eqtlMapFilt; gene mapping filter of eQTL.

Supplementary Table 3. Geneset analysis

Results of top 100 variables

TEST_DIRECTION = one-sided, positive (set), two-sided (covar)

CONDITIONED_INTERNAL = gene size, gene density, inverse mac, log(gene size), log(gene density), log(inverse mac)

VARIABLE	TYPE	NGENES	BETA	BETA_STD	SE	P	P after Bonferroni's correction	P_FDR	FULL_NAME
GO_bp:go_neurogenesis	SET	1515	0.11572	0.031451	0.024066	7.66E-07	0.007653	0.007650808	GO_bp:go_neurogenesis
GO_bp:go_stress_induced_prem...	SET	8	1.3617	0.028036	0.29539	2.03E-06	0.020267	0.01013782	GO_bp:go_stress_induced_premature_senescence
GO_bp:go_neuron_differentiat...	SET	1277	0.11609	0.029166	0.026083	4.30E-06	0.042967	0.014316133	GO_bp:go_neuron_differentiation
GO_bp:go_positive_regulation...739	SET	6	1.453	0.025909	0.34688	1.41E-05	0.140841	0.0352077	GO_bp:go_positive_regulation_of_skeletal_muscle_cell_differentiation
GO_bp:go_neuron_projection_g...	SET	263	0.23241	0.02725	0.057414	2.59E-05	0.259149	0.039095886	GO_bp:go_neuron_projection_guidance
GO_bp:go_neuron_development	SET	1039	0.11561	0.026375	0.028629	2.70E-05	0.270076	0.039095886	GO_bp:go_neuron_development
GO_bp:go_commitment_of_neuro...	SET	7	1.5742	0.030318	0.39012	2.74E-05	0.273861	0.039095886	GO_bp:go_commitment_of_neuronal_cell_to_specific_neuron_type_in_forebrain
GO_bp:go_cell_morphogenesis_...2	SET	546	0.15629	0.026202	0.039341	3.57E-05	0.356282	0.04457145	GO_bp:go_cell_morphogenesis_involved_in_neuron_differentiation
GO_bp:go_glial_cell_differen...	SET	201	0.25222	0.025896	0.064341	4.44E-05	0.443807	0.0482148	GO_bp:go_glial_cell_differentiation
GO_mf:go_double_stranded_dna...	SET	908	0.11782	0.025219	0.03023	4.88E-05	0.487524	0.0482148	GO_mf:go_double_stranded_dna_binding
GO_bp:go_glial_cell_fate_com...	SET	14	1.1329	0.030851	0.2922	5.31E-05	0.529893	0.0482148	GO_bp:go_glial_cell_fate_commitment
GO_bp:go_regulation_of_skele...3	SET	19	0.68838	0.021836	0.17967	6.39E-05	0.638703	0.052552246	GO_bp:go_regulation_of_skeletal_muscle_cell_differentiation
GO_bp:go_cell_part_morphogen...	SET	640	0.13954	0.025263	0.036578	6.84E-05	0.682770	0.052552246	GO_bp:go_cell_part_morphogenesis
GO_mf:go_sequence_specific_d...2	SET	822	0.12059	0.024619	0.032251	9.26E-05	0.925029	0.066063486	GO_mf:go_sequence_specific_double_stranded_dna_binding
GO_mf:go_sequence_specific_d...1	SET	1052	0.10366	0.023788	0.028738	0.0001552	1.550138	0.103342507	GO_mf:go_sequence_specific_dna_binding
GO_bp:go_positive_regulation...405	SET	12	0.82997	0.020927	0.23298	0.00018424	1.840189	0.11501182	GO_bp:go_positive_regulation_of_interleukin_6_biosynthetic_process
GO_bp:go_regulation_of_gluco...3	SET	7	1.1932	0.02298	0.33674	0.00019807	1.978323	0.116371951	GO_bp:go_regulation_of_glucocorticoid_receptor_signaling_pathway
GO_bp:go_radial_glial_cell_d...	SET	12	1.0591	0.026703	0.30356	0.00024316	2.428682	0.128855714	GO_bp:go_radial_glial_cell_differentiation
GO_bp:go_negative_regulation...459	SET	287	0.18417	0.022543	0.05282	0.00024512	2.448259	0.128855714	GO_bp:go_negative_regulation_of_nervous_system_development
GO_mf:go_regulatory_region_n...	SET	890	0.10677	0.022638	0.030908	0.00027642	2.760883	0.137682202	GO_mf:go_regulatory_region_nucleic_acid_binding

GO_bp:go_cellular_component_...5	SET	1065	0.097507	0.022505	0.028329	0.00028948	2.891326	0.137682202	GO_bp:go_cellular_component_morphogenesis
GO_cc:go_perinuclear_region_...	SET	661	0.11573	0.021281	0.03401	0.00033413	3.337290	0.150692864	GO_cc:go_perinuclear_region_of_cytoplasm
GO_bp:go_negative_regulation...106	SET	310	0.17387	0.022105	0.051251	0.00034701	3.465936	0.150692864	GO_bp:go_negative_regulation_of_cell_development
GO_bp:go_astrocyte_different...	SET	73	0.36354	0.022571	0.1078	0.00037327	3.728221	0.155342532	GO_bp:go_astrocyte_differentiation
GO_bp:go_regulation_of_chlor...	SET	9	0.94761	0.020693	0.28556	0.0004535	4.529558	0.169169167	GO_bp:go_regulation_of_chloride_transport
GO_bp:go_regulation_of_nervo...	SET	856	0.10476	0.021804	0.031603	0.00045924	4.586889	0.169169167	GO_bp:go_regulation_of_nervous_system_development
GO_bp:go_cell_morphogenesis_...1	SET	690	0.11637	0.021845	0.035117	0.00046121	4.606565	0.169169167	GO_bp:go_cell_morphogenesis_involved_in_differentiation
GO_bp:go_striatum_development	SET	18	0.73818	0.022791	0.22383	0.00048809	4.875043	0.169169167	GO_bp:go_striatum_development
GO_bp:go_positive_regulation...355	SET	7	1.1603	0.022348	0.35204	0.00049118	4.905906	0.169169167	GO_bp:go_positive_regulation_of_histone_h4_acetylation
GO_bp:go_regulation_of_cell_...20	SET	876	0.10115	0.021286	0.031213	0.00059723	5.965133	0.198837775	GO_bp:go_regulation_of_cell_development
GO_cc:go_pml_body	SET	94	0.27642	0.019464	0.085792	0.00063765	6.368848	0.201560961	GO_cc:go_pml_body
GO_bp:go_erythrocyte_maturat...	SET	14	0.81639	0.022232	0.25366	0.00064577	6.449951	0.201560961	GO_bp:go_erythrocyte_maturation
GO_bp:go_gliogenesis	SET	271	0.17608	0.020952	0.055259	0.0007216	7.207341	0.218100822	GO_bp:go_gliogenesis
GO_bp:go_axon_development	SET	479	0.13296	0.020917	0.041933	0.00076138	7.604663	0.218100822	GO_bp:go_axon_development
GO_bp:go_negative_regulation...665	SET	11	0.88681	0.021408	0.27977	0.00076427	7.633529	0.218100822	GO_bp:go_negative_regulation_of_telomerase_activity
GO_bp:go_positive_regulation...56	SET	76	0.3254	0.020612	0.10398	0.0008775	8.764470	0.2434575	GO_bp:go_positive_regulation_of_axonogenesis
GO_bp:go_fat_cell_differenti...	SET	206	0.19534	0.020302	0.06266	0.00091328	9.121841	0.246170907	GO_bp:go_fat_cell_differentiation
GO_cc:go_axon	SET	575	0.1183	0.020336	0.038089	0.00095032	9.491796	0.246170907	GO_cc:go_axon
GO_bp:go_embryonic_camera_ty...3	SET	26	0.51171	0.018984	0.16528	0.00098231	9.811312	0.246170907	GO_bp:go_embryonic_camera_type_eye_morphogenesis
GO_bp:go_ganglion_development	SET	14	0.755	0.02056	0.24522	0.0010404	10.391515	0.246170907	GO_bp:go_ganglion_development
GO_bp:go_neuromuscular_junct...	SET	46	0.39183	0.019325	0.12759	0.0010681	10.668183	0.246170907	GO_bp:go_neuromuscular_junction_development
GO_bp:go_histone_h3_k27_deme...	SET	2	2.8915	0.029772	0.94239	0.001078	10.767064	0.246170907	GO_bp:go_histone_h3_k27_demethylation
GO_mf:go_histone_demethylase...2	SET	2	2.8915	0.029772	0.94239	0.001078	10.767064	0.246170907	GO_mf:go_histone_demethylase_activity_h3_k27_specific_
GO_bp:go_regulation_of_trans...1	SET	419	0.13308	0.019613	0.043425	0.0010913	10.899904	0.246170907	GO_bp:go_regulation_of_trans_synaptic_signaling
GO_bp:go_taxis	SET	604	0.11743	0.020673	0.038377	0.0011091	11.077691	0.246170907	GO_bp:go_taxis
GO_bp:go_neuron_migration	SET	143	0.23827	0.020667	0.078179	0.0011545	11.531146	0.250677087	GO_bp:go_neuron_migration
GO_bp:go_positive_regulation...490	SET	15	0.65762	0.018537	0.21706	0.0012258	12.243290	0.255776033	GO_bp:go_positive_regulation_of_membrane_protein_ectodomain_proteolysis
GO_mf:go_nuclear_hormone_rec...	SET	147	0.21485	0.018892	0.070935	0.0012292	12.277250	0.255776033	GO_mf:go_nuclear_hormone_receptor_binding
GO_bp:go_regulation_of_anion...1	SET	9	0.92183	0.02013	0.30547	0.0012753	12.737696	0.25609232	GO_bp:go_regulation_of_anion_channel_activity

GO_bp:go_branching_morphogen...1	SET	10	0.97648	0.022477	0.32375	0.001282	12.804616	0.25609232	GO_bp:go_branching_morphogenesis_of_a_nerve
GO_bp:go_regulation_of_skele...8	SET	48	0.37483	0.018884	0.12537	0.0013981	13.964223	0.269469352	GO_bp:go_regulation_of_skeletal_muscle_tissue_development
GO_bp:go_embryonic_camera_ty...1	SET	37	0.41806	0.018497	0.14007	0.0014217	14.199940	0.269469352	GO_bp:go_embryonic_camera_type_eye_development
GO_bp:go_embryonic_eye_morph...	SET	34	0.43588	0.018488	0.1464	0.0014556	14.538533	0.269469352	GO_bp:go_embryonic_eye_morphogenesis
GO_bp:go_vocalization_behavi...	SET	14	0.83931	0.022856	0.28297	0.0015102	15.083878	0.269469352	GO_bp:go_vocalization_behavior
GO_mf:go_steroid_hormone_rec...2	SET	89	0.27351	0.018742	0.092351	0.0015322	15.303614	0.269469352	GO_mf:go_steroid_hormone_receptor_binding
GO_bp:go_positive_regulation...646	SET	38	0.41675	0.018686	0.14092	0.0015537	15.518356	0.269469352	GO_bp:go_positive_regulation_of_protein_acetylation
GO_cc:go_neuron_to_neuron_sy...	SET	328	0.14597	0.01908	0.049369	0.0015568	15.549318	0.269469352	GO_cc:go_neuron_to_neuron_synapse
GO_bp:go_forebrain_neuron_fa...	SET	10	0.99614	0.022929	0.33709	0.0015648	15.629222	0.269469352	GO_bp:go_forebrain_neuron_fate_commitment
GO_bp:go_regulation_of_cell_...21	SET	1710	0.066928	0.019216	0.02279	0.0016608	16.588070	0.281153736	GO_bp:go_regulation_of_cell_differentiation
GO_mf:go_1_phosphatidylinosi...5	SET	15	0.76593	0.02159	0.2626	0.001771	17.688748	0.294812467	GO_mf:go_1_phosphatidylinositol_binding
GO_mf:go_nuclear_receptor_bi...	SET	108	0.24179	0.018243	0.083373	0.0018675	18.652590	0.305780164	GO_mf:go_nuclear_receptor_binding
GO_bp:go_regulation_of_neuro...5	SET	613	0.10694	0.018963	0.037178	0.0020128	20.103846	0.310536	GO_bp:go_regulation_of_neuron_differentiation
GO_bp:go_regulation_of_inorg...	SET	7	0.96201	0.018528	0.33467	0.002026	20.235688	0.310536	GO_bp:go_regulation_of_inorganic_anion_transmembrane_transport
GO_bp:go_synapse_organization	SET	372	0.13612	0.018926	0.047376	0.0020343	20.318588	0.310536	GO_bp:go_synapse_organization
GO_bp:go_telencephalon_devel...	SET	240	0.17748	0.019891	0.061808	0.0020457	20.432452	0.310536	GO_bp:go_telencephalon_development
GO_mf:go_mrna_binding	SET	219	0.16034	0.017175	0.055859	0.002052	20.495376	0.310536	GO_mf:go_mrna_binding
GO_bp:go_locomotory_behavior	SET	191	0.19171	0.019192	0.067191	0.0021669	21.642997	0.319660065	GO_bp:go_locomotory_behavior
GO_bp:go_central_nervous_sys...1	SET	919	0.086372	0.018594	0.030287	0.0021763	21.736884	0.319660065	GO_bp:go_central_nervous_system_development
GO_mf:go_dna_binding_transcr...2	SET	1608	0.069572	0.019428	0.024543	0.0022957	22.929452	0.332310893	GO_mf:go_dna_binding_transcription_factor_activity
GO_bp:go_glomerular_basement...	SET	10	0.76485	0.017605	0.27244	0.0024997	24.967004	0.353420456	GO_bp:go_glomerular_basement_membrane_development
GO_mf:go_core_promoter_bindi...	SET	52	0.35352	0.018535	0.126	0.0025123	25.092852	0.353420456	GO_mf:go_core_promoter_binding
GO_bp:go_negative_regulation...355	SET	12	0.66795	0.016841	0.23982	0.0026776	26.743869	0.362853526	GO_bp:go_negative_regulation_of_keratinocyte_proliferation
GO_bp:go_double_strand_break...4	SET	7	0.78126	0.015047	0.28053	0.0026796	26.763845	0.362853526	GO_bp:go_double_strand_break_repair_via_synthesis_dependent_strand_annealing
GO_mf:go_core_promoter_seque...	SET	44	0.37855	0.018261	0.13601	0.0026937	26.904676	0.362853526	GO_mf:go_core_promoter_sequence_specific_dna_binding
GO_bp:go_epithelial_cell_pro...1	SET	354	0.13284	0.018026	0.047802	0.0027301	27.268239	0.362853526	GO_bp:go_epithelial_cell_proliferation
GO_bp:go_keratinocyte_prolif...	SET	38	0.4077	0.01828	0.14691	0.002761	27.576868	0.362853526	GO_bp:go_keratinocyte_proliferation
GO_bp:go_cellular_response_t...14	SET	33	0.45115	0.018853	0.16313	0.0028435	28.400878	0.368842571	GO_bp:go_cellular_response_to_cadmium_ion
GO_bp:go_positive_regulation...615	SET	30	0.42736	0.017029	0.15543	0.0029869	29.833157	0.37241676	GO_bp:go_positive_regulation_of_peptidyl_lysine_acetylation

GO_bp:go_corticosteroid_rece...	SET	15	0.59221	0.016693	0.21554	0.0030051	30.014939	0.37241676	GO_bp:go_corticosteroid_receptor_signaling_pathway
GO_bp:go_skeletal_muscle_org...	SET	162	0.19021	0.017551	0.069269	0.0030202	30.165758	0.37241676	GO_bp:go_skeletal_muscle_organ_development
GO_bp:go_positive_regulation...692	SET	9	0.80229	0.01752	0.29218	0.0030202	30.165758	0.37241676	GO_bp:go_positive_regulation_of_receptor_binding
GO_bp:go_pinocytosis	SET	20	0.47478	0.015451	0.17392	0.0031713	31.674944	0.37846575	GO_bp:go_pinocytosis
GO_bp:go_behavior	SET	566	0.10708	0.018268	0.0393	0.0032221	32.182335	0.37846575	GO_bp:go_behavior
GO_mf:go_proximal_promoter_s...	SET	512	0.10912	0.017732	0.040151	0.0032895	32.855526	0.37846575	GO_mf:go_proximal_promoter_sequence_specific_dna_binding
GO_bp:go_ectopic_germ_cell_p...	SET	7	1.1233	0.021635	0.41363	0.0033091	33.051291	0.37846575	GO_bp:go_ectopic_germ_cell_programmed_cell_death
GO_bp:go_regulation_of_neutr...2	SET	7	0.78155	0.015052	0.28779	0.0033106	33.066273	0.37846575	GO_bp:go_regulation_of_neutrophil_degranulation
GO_bp:go_cellular_response_t...31	SET	33	0.42316	0.017683	0.15585	0.0033146	33.106225	0.37846575	GO_bp:go_cellular_response_to_estradiol_stimulus
GO_bp:go_skeletal_muscle_cel...1	SET	66	0.28828	0.017022	0.10625	0.0033345	33.304986	0.37846575	GO_bp:go_skeletal_muscle_cell_differentiation
GO_bp:go_spinal_cord_develop...	SET	99	0.25671	0.018548	0.09477	0.0033798	33.757442	0.379297106	GO_bp:go_spinal_cord_development
GO_cc:go_synapse	SET	1115	0.074287	0.017519	0.027487	0.0034432	34.390682	0.382118684	GO_cc:go_synapse
GO_bp:go_forebrain_neuron_di...	SET	50	0.37995	0.019535	0.14139	0.0036061	36.017727	0.390573302	GO_bp:go_forebrain_neuron_differentiation
GO_bp:go_peptidyl_lysine_met...	SET	120	0.21268	0.016909	0.079179	0.0036189	36.145573	0.390573302	GO_bp:go_peptidyl_lysine_methylation
GO_bp:go_lipid_metabolic_pro...	SET	1174	0.070212	0.016962	0.026161	0.0036425	36.381290	0.390573302	GO_bp:go_lipid_metabolic_process
GO_bp:go_regulation_of_lipas...	SET	93	0.25612	0.017939	0.09554	0.0036758	36.713890	0.390573302	GO_bp:go_regulation_of_lipase_activity
GO_bp:go_positive_regulation...219	SET	1270	0.070454	0.017655	0.026367	0.0037733	37.687720	0.395769404	GO_bp:go_positive_regulation_of_developmental_process
GO_cc:go_neuron_projection	SET	1231	0.069862	0.017254	0.026202	0.0038389	38.342933	0.395769404	GO_cc:go_neuron_projection
GO_bp:go_negative_regulation...107	SET	659	0.095788	0.017588	0.035951	0.0038597	38.550684	0.395769404	GO_bp:go_negative_regulation_of_cell_differentiation
GO_bp:go_regulation_of_axono...	SET	169	0.18507	0.017438	0.069512	0.0038832	38.785402	0.395769404	GO_bp:go_regulation_of_axonogenesis
GO_bp:go_regulation_of_synap...3	SET	7	0.90698	0.017468	0.34175	0.0039812	39.764226	0.401658844	GO_bp:go_regulation_of_synapse_structural_plasticity
GO_mf:go_metalloexopeptidase...	SET	59	0.32561	0.018182	0.12299	0.0040571	40.522315	0.404287857	GO_mf:go_metalloexopeptidase_activity

Supplementary Table 4. Enrichment for heritability partitioned based on 52 functional genomic annotation

Category	Prop._SNPs	Prop._h2	Prop._h2_std_error	Enrichment	Enrichment_std_error	Enrichment_p	Coefficient	Coefficient_std_error	Coefficient_z.score	Coefficient_P_value	Coefficient_Pval_FDR
Conserved_LindbladToh	0.026062507	0.562013897	0.067978619	21.56407683	2.60829166	1.94E-13	1.30E-07	1.76E-08	7.379472752	7.95E-14	4.21E-12
DHS_Trynka.extend.500	0.498778726	1.011070457	0.091328076	2.027092185	0.18310339	1.84E-07	1.99E-08	4.78E-09	4.154932491	1.63E-05	0.00043195
H3K4me3_Trynka	0.133307338	0.413329391	0.063511658	3.10057495	0.476430325	2.49E-05	2.16E-08	7.46E-09	2.901122395	0.001859143	0.025168468
H3K9ac_peaks_Trynka	0.038770134	0.27629673	0.056563019	7.126535294	1.458932757	3.67E-05	3.46E-08	1.19E-08	2.89438546	0.001899507	0.025168468
TFBS_ENCODE	0.132450691	0.383531609	0.105971164	2.895655782	0.800080116	0.019242407	2.11E-08	8.78E-09	2.402364921	0.008144725	0.086334085
H3K4me1_peaks_Trynka	0.171318183	0.614238844	0.1057583	3.58536866	0.617320928	6.34E-05	1.53E-08	6.61E-09	2.321495472	0.010130059	0.089482188
Transcribed_Hoffman	0.3454184	0.472636467	0.089153441	1.368301361	0.258102757	0.150741677	4.91E-09	3.40E-09	1.443083474	0.074498479	0.503455639
CTCF_Hoffman.extend.500	0.071062184	0.086906025	0.053411545	1.222957406	0.751616994	0.767400351	1.17E-08	8.21E-09	1.420157554	0.077780909	0.503455639
WeakEnhancer_Hoffman	0.021092539	0.143463466	0.048543178	6.801621414	2.301438314	0.01201759	2.57E-08	1.87E-08	1.369045883	0.085492467	0.503455639
H3K27ac_PGC2	0.269476688	0.395421224	0.070801514	1.467367093	0.262737065	0.08007717	7.68E-09	8.32E-09	0.923184393	0.17795556	0.765557061
Enhancer_Andersson.extend.500	0.019069119	0.041737883	0.024727139	2.188768359	1.296711141	0.363209848	1.07E-08	1.23E-08	0.869472133	0.192294472	0.765557061
Intron_UCSC	0.387452633	0.460716442	0.035207941	1.189091008	0.090870309	0.035873336	1.75E-08	2.15E-08	0.815137011	0.207496944	0.765557061
Promoter_UCSC.extend.500	0.03862705	0.068362974	0.024694627	1.769821222	0.639309143	0.22849319	1.62E-08	2.21E-08	0.730891384	0.23242275	0.765557061
UTR_5_UCSC	0.005424529	0.028078892	0.019974439	5.176282526	3.682244329	0.257632303	1.86E-08	2.62E-08	0.710134787	0.238810278	0.765557061
WeakEnhancer_Hoffman.extend.500	0.088958455	0.231120432	0.048218697	2.598071565	0.542036137	0.004924905	5.65E-09	8.29E-09	0.681227587	0.247863748	0.765557061
SuperEnhancer_Hnisz.extend.500	0.171606713	0.234124122	0.024355298	1.364306317	0.141925089	0.011050946	2.31E-08	3.66E-08	0.630442329	0.264202612	0.765557061
H3K4me1_Trynka.extend.500	0.609156889	0.836477415	0.053402164	1.37317238	0.087665697	5.38E-05	2.12E-09	3.61E-09	0.58786089	0.278312835	0.765557061
UTR_3_UCSC	0.011054234	0.024490988	0.020782367	2.215530129	1.880036888	0.518349644	1.14E-08	1.94E-08	0.586941483	0.278621504	0.765557061
H3K27ac_Hnisz	0.391168762	0.468563758	0.038592553	1.197855769	0.098659599	0.049789482	5.42E-09	9.42E-09	0.575191834	0.282580782	0.765557061
Conserved_LindbladToh.extend.500	0.332514033	0.750299303	0.067353274	2.25644403	0.202557688	1.53E-09	1.45E-09	2.60E-09	0.556631957	0.288889457	0.765557061
TSS_Hoffman	0.018218884	0.077576363	0.035137191	4.258019513	1.928613823	0.09295134	1.09E-08	2.19E-08	0.49714526	0.30954331	0.781228354
Repressed_Hoffman.extend.500	0.719053325	0.623997569	0.034343367	0.867804302	0.04776192	0.005699406	9.63E-10	3.33E-09	0.289290772	0.386179437	0.91082459
FetalDHS_Trynka	0.08475655	0.153670679	0.101819981	1.81308322	1.201322856	0.498839091	3.25E-09	1.30E-08	0.249971455	0.401304712	0.91082459
Repressed_Hoffman	0.461222273	0.342083548	0.097917301	0.741689135	0.212299593	0.223798068	7.63E-10	3.45E-09	0.221250047	0.412448871	0.91082459

Enhancer_Hoffman	0.063320306	0.22569861	0.067268444	3.564395434	1.062351839	0.017000346	1.31E-09	1.14E-08	0.11547479	0.454034401	0.911458717
DGF_ENCODE	0.137593608	0.156931908	0.123374827	1.140546501	0.896661039	0.875460971	6.24E-10	8.60E-09	0.072535993	0.471087681	0.911458717
H3K4me1_Trynka	0.426568328	0.711592035	0.085242892	1.668178315	0.199834085	0.00155735	-1.14E-10	5.18E-09	-0.021916817	0.508742845	0.911458717
FetalDHS_Trynka.extend.500	0.285010389	0.613186378	0.08580447	2.151452727	0.301057341	0.000319527	-4.07E-10	4.98E-09	-0.081630559	0.53252975	0.911458717
Transcribed_Hoffman.extend.500	0.763061541	0.76346352	0.054696369	1.000526798	0.071680155	0.994114097	-3.63E-10	2.67E-09	-0.135617274	0.553938076	0.911458717
H3K9ac_Trynka.extend.500	0.23058338	0.365306988	0.054341486	1.584272848	0.23566957	0.016670062	-1.12E-09	5.17E-09	-0.216851823	0.585838088	0.911458717
PromoterFlanking_Hoffman	0.008427263	0.007602112	0.031861096	0.902085515	3.780716905	0.979350174	-7.38E-09	3.05E-08	-0.242262966	0.595711796	0.911458717
TSS_Hoffman.extend.500	0.034825544	0.094745474	0.032130824	2.720574121	0.92262232	0.064520307	-3.39E-09	1.33E-08	-0.254808277	0.600564409	0.911458717
Coding_UCSC	0.014658156	0.068110009	0.030920055	4.646560398	2.109409584	0.085678125	-5.76E-09	1.91E-08	-0.301216959	0.618375471	0.911458717
UTR_3_UCSC.extend.500	0.026931133	0.023416692	0.022547067	0.869502653	0.837211956	0.876218174	-3.03E-09	9.05E-09	-0.334392727	0.630958387	0.911458717
UTR_5_UCSC.extend.500	0.027805834	0.035720131	0.02328192	1.284627218	0.837303417	0.733497078	-2.17E-09	6.46E-09	-0.336357035	0.631699177	0.911458717
PromoterFlanking_Hoffman.extend.500	0.033471479	0.033909387	0.037985758	1.013083012	1.134869421	0.990799148	-4.74E-09	1.25E-08	-0.379126818	0.647703154	0.911458717
H3K9ac_Trynka	0.126111135	0.346419317	0.064588476	2.746936794	0.512155219	0.000739478	-3.40E-09	8.62E-09	-0.394783606	0.653498703	0.911458717
Promoter_UCSC	0.031163747	0.040297056	0.029868484	1.293074803	0.95843686	0.759738646	-1.61E-08	2.61E-08	-0.615924999	0.731027989	0.937072682
SuperEnhancer_Hnisz	0.16842023	0.221881769	0.025026285	1.317429435	0.148594293	0.033082734	-2.42E-08	3.71E-08	-0.652488784	0.742957047	0.937072682
H3K27ac_Hnisz.extend.500	0.422590835	0.463745787	0.047402632	1.097387231	0.112171462	0.390905098	-6.26E-09	9.58E-09	-0.653474235	0.743274703	0.937072682
Enhancer_Hoffman.extend.500	0.153929118	0.267329761	0.059005268	1.736706906	0.383327526	0.061735947	-5.63E-09	8.04E-09	-0.699771128	0.757964876	0.937072682
DHS_Trynka	0.167754924	0.202267115	0.12758236	1.205729818	0.760528255	0.786832882	-8.11E-09	1.12E-08	-0.72315503	0.765207678	0.937072682
Intron_UCSC.extend.500	0.397130023	0.504505533	0.028550702	1.270378726	0.07189258	0.000107042	-1.64E-08	2.14E-08	-0.765278479	0.777947132	0.937072682
H3K4me3_peaks_Trynka	0.041789404	0.129988573	0.057793605	3.110562958	1.382972691	0.133400894	-1.09E-08	1.19E-08	-0.920032628	0.821222146	0.967217194
H3K27ac_PGC2.extend.500	0.336034433	0.349324902	0.061074925	1.039550913	0.181751985	0.827959835	-9.98E-09	7.59E-09	-1.314857655	0.905721127	0.999168762
Enhancer_Andersson	0.004335371	-0.018820293	0.019254574	-4.341103469	4.441274921	0.230511478	-5.49E-08	3.41E-08	-1.609963862	0.946297127	0.999168762
CTCF_Hoffman	0.023828848	-0.03823071	0.04276549	-1.6043877	1.794694031	0.148004024	-3.01E-08	1.67E-08	-1.805067388	0.964467931	0.999168762
DHS_peaks_Trynka	0.111765806	0.010095858	0.107787985	0.090330471	0.964409325	0.345891847	-2.74E-08	1.34E-08	-2.050672389	0.979850569	0.999168762
DGF_ENCODE.extend.500	0.541500875	0.561019317	0.082933513	1.036045081	0.153154901	0.814530243	-8.81E-09	3.79E-09	-2.322165293	0.989887981	0.999168762
Coding_UCSC.extend.500	0.064555416	0.027730887	0.031789294	0.429567158	0.492434188	0.251249697	-1.59E-08	5.81E-09	-2.744734614	0.996972008	0.999168762
TFBS_ENCODE.extend.500	0.343443574	0.329069593	0.088771182	0.958147472	0.258473847	0.870840044	-1.26E-08	4.29E-09	-2.932404943	0.998318261	0.999168762
H3K4me3_Trynka.extend.500	0.25548215	0.240839109	0.061976527	0.942684679	0.242586527	0.812706748	-1.52E-08	4.84E-09	-3.144717017	0.999168762	0.999168762

Supplementary Table 5. Results from multiple-tissue analysis of gene expression using LDSC-SEG

Name	Coefficient	Coefficient_std_error	Coefficient_P_value	Coefficient_Pval_FDR
A08.186.211.730.885.287.500.Cerebral.Cortex	2.95E-09	7.58E-10	4.94E-05	0.006903992
A08.186.211.730.885.287.500.270.Frontal.Lobe	2.88E-09	7.55E-10	6.74E-05	0.006903992
A08.186.211.464.Limbic.System	2.86E-09	7.71E-10	1.03E-04	0.007009921
Brain_Cortex	2.52E-09	7.09E-10	0.000185612	0.009512638
A08.186.211.730.885.287.500.571.735.Visual.Cortex	2.48E-09	7.36E-10	0.000385239	0.013032611
Brain_Anterior_cingulate_cortex_(BA24)	2.36E-09	7.06E-10	0.000406643	0.013032611
Brain_Frontal_Cortex_(BA9)	2.17E-09	6.52E-10	0.000445016	0.013032611
A08.186.211.653.Mesencephalon	2.61E-09	7.98E-10	0.000529629	0.013571744
A08.186.211.464.710.225.Entorhinal.Cortex	2.41E-09	7.58E-10	0.000750223	0.015824196
A09.371.729.Retina	2.56E-09	8.08E-10	0.000771912	0.015824196
A08.186.211.730.885.287.249.Basal.Ganglia	2.06E-09	7.26E-10	0.002238084	0.041709753
A08.186.211.730.317.Diencephalon	2.37E-09	8.82E-10	0.003556969	0.060764887
A08.186.211.464.405.Hippocampus	1.94E-09	7.31E-10	0.003992094	0.062952248
A08.186.211.730.317.357.Hypothalamus	2.30E-09	8.76E-10	0.004363785	0.063898281
A08.186.211.730.317.357.352.435.Hypothalamo.Hypophyseal.System	2.15E-09	8.57E-10	0.005967523	0.081556147
Brain_Hypothalamus	1.64E-09	6.77E-10	0.007553561	0.088211877
Brain_Putamen_(basal_ganglia)	1.73E-09	7.11E-10	0.007595006	0.088211877
A08.186.211.Brain	1.86E-09	7.68E-10	0.007745433	0.088211877
A08.186.211.132.Brain.Stem	1.71E-09	7.41E-10	0.010604402	0.11441592
A08.186.211.865.428.Metencephalon	1.72E-09	7.76E-10	0.013377937	0.137123851
Spleen	1.73E-09	7.87E-10	0.014047307	0.137128471
Brain_Nucleus_accumbens_(basal_ganglia)	1.51E-09	6.93E-10	0.014898453	0.138826496
Brain_Caudate_(basal_ganglia)	1.50E-09	7.06E-10	0.016962403	0.151186639
Brain_Cerebellar_Hemisphere	1.41E-09	7.21E-10	0.025209458	0.215330788

Brain_Amygdala	1.31E-09	7.10E-10	0.032539634	0.266825002
A15.145.229.188.Blood.Platelets	1.32E-09	7.43E-10	0.038113828	0.300512875
Brain_Cerebellum	1.27E-09	7.39E-10	0.042227348	0.320615053
A06.407.071.Adrenal.Glands	1.32E-09	7.87E-10	0.046666353	0.332283659
A08.186.211.730.885.287.500.670.Parietal.Lobe	1.29E-09	7.76E-10	0.047602533	0.332283659
Brain_Substantia_nigra	1.19E-09	7.19E-10	0.049258715	0.332283659
A08.186.211.132.810.428.200.Cerebellum	1.28E-09	7.81E-10	0.050247773	0.332283659
A11.872.653.Neural.Stem.Cells	1.40E-09	8.69E-10	0.053899487	0.345293586
Brain_Hippocampus	9.87E-10	6.72E-10	0.071063466	0.441454862
A06.407.071.140.Adrenal.Cortex	1.14E-09	8.06E-10	0.078294651	0.472070687
A08.186.211.730.885.287.249.487.Corpus.Striatum	1.03E-09	7.62E-10	0.087545949	0.511530859
A15.382.490.315.583.Neutrophils	1.13E-09	8.45E-10	0.089829809	0.511530859
A15.145.Blood	1.13E-09	8.54E-10	0.093423633	0.517617426
A07.231.Blood.Vessels	1.06E-09	8.78E-10	0.112542027	0.607134619
A15.145.846.Serum	9.73E-10	8.27E-10	0.119878782	0.630132062
A03.556.875.875.Stomach	1.03E-09	9.21E-10	0.132276329	0.677916189
Pituitary	7.79E-10	7.50E-10	0.149580162	0.747900808
A11.329.830.Stromal.Cells	7.71E-10	8.28E-10	0.175877701	0.854944567
Lung	7.10E-10	7.85E-10	0.182926647	0.854944567
A11.118.637.555.567.562.B.Lymphocytes	7.43E-10	8.24E-10	0.183500297	0.854944567
A07.231.114.Arteries	6.84E-10	7.88E-10	0.192676084	0.877746604
A11.118.637.Leukocytes	6.61E-10	7.92E-10	0.201989378	0.900170054
A15.378.316.Bone.Marrow.Cells	5.57E-10	8.54E-10	0.257327354	0.99999552
Colon_Sigmoid	4.27E-10	7.37E-10	0.281001037	0.99999552
A11.436.275.Endothelial.Cells	4.89E-10	8.91E-10	0.291369941	0.99999552
A15.145.300.Fetal.Blood	4.82E-10	8.79E-10	0.291573769	0.99999552
Thyroid	3.95E-10	7.80E-10	0.306193712	0.99999552
A11.436.397.Keratinocytes	3.46E-10	8.02E-10	0.333160422	0.99999552
A15.382.812.Mononuclear.Phagocyte.System	3.46E-10	8.20E-10	0.336606572	0.99999552

A06.407.900.Thyroid.Gland	2.76E-10	7.10E-10	0.348906009	0.99999552
A11.436.Epithelial.Cells	2.80E-10	7.67E-10	0.357734843	0.99999552
A15.382.680.Phagocytes	2.76E-10	8.13E-10	0.366945916	0.99999552
A15.145.229.Blood.Cells	2.38E-10	8.20E-10	0.385636344	0.99999552
Brain_Spinal_cord_(cervical_c-1)	2.11E-10	7.34E-10	0.386971141	0.99999552
A07.231.908.Veins	1.63E-10	8.10E-10	0.420036793	0.99999552
A15.382.490.555.567.Lymphocytes	1.63E-10	8.22E-10	0.421309032	0.99999552
A11.620.520.Myocytes..Smooth.Muscle	8.18E-11	8.18E-10	0.460158651	0.99999552
A11.872.190.260.Embryoid.Bodies	7.94E-11	8.06E-10	0.460744729	0.99999552
A11.118.637.555.567.569.T.Lymphocytes	8.33E-11	8.75E-10	0.46206523	0.99999552
A15.145.229.637.555.567.562.725.Plasma.Cells	4.67E-11	8.49E-10	0.478061256	0.99999552
A02.633.567.850.Quadriceps.Muscle	1.69E-11	7.78E-10	0.491355478	0.99999552
A03.556.500.760.Salivary.Glands	1.34E-11	7.91E-10	0.493227225	0.99999552
A09.371.Eye	1.03E-12	7.76E-10	0.499472386	0.99999552
A15.145.229.637.555.567.569.200.CD4.Positive.T.Lymphocytes	-7.08E-11	8.01E-10	0.535231982	0.99999552
A10.615.284.473.Chorion	-7.39E-11	7.29E-10	0.540381158	0.99999552
A15.382.Immune.System	-1.01E-10	7.67E-10	0.552462906	0.99999552
A15.145.229.637.555.Leukocytes..Mononuclear	-1.37E-10	8.44E-10	0.564306536	0.99999552
A03.556.875.Upper.Gastrointestinal.Tract	-1.87E-10	9.06E-10	0.58189318	0.99999552
Prostate	-1.53E-10	6.59E-10	0.591960813	0.99999552
Skin_Sun_Exposed_(Lower_leg)	-1.68E-10	7.17E-10	0.592558153	0.99999552
A14.549.167.Dentition	-1.94E-10	7.89E-10	0.59709667	0.99999552
Testis	-1.88E-10	7.33E-10	0.601356897	0.99999552
Fallopian_Tube	-2.10E-10	7.65E-10	0.6081195	0.99999552
Colon_Transverse	-2.25E-10	8.10E-10	0.609209818	0.99999552
Ovary	-2.29E-10	8.24E-10	0.609619199	0.99999552
Heart_Left_Ventricle	-2.17E-10	7.62E-10	0.612255448	0.99999552
Muscle_Skeletal	-2.40E-10	8.14E-10	0.615862246	0.99999552
Esophagus_Muscularis	-2.22E-10	6.86E-10	0.626855278	0.99999552

A07.231.908.670.874.Umbilical.Veins	-2.76E-10	8.37E-10	0.628973224	0.99999552
A11.118.637.555.567.569.200.700.T.Lymphocytes..Regulatory	-2.69E-10	7.57E-10	0.638585802	0.99999552
A03.556.500.760.464.Parotid.Gland	-3.14E-10	7.78E-10	0.65685416	0.99999552
Uterus	-3.40E-10	8.24E-10	0.660268025	0.99999552
A10.690.467.Muscle..Smooth	-3.37E-10	8.02E-10	0.662905157	0.99999552
A11.872.700.500.Induced.Pluripotent.Stem.Cells	-3.48E-10	7.58E-10	0.676988131	0.99999552
Adrenal_Gland	-3.74E-10	7.64E-10	0.687713168	0.99999552
Skin_Not_Sun_Exposed_(Suprapubic)	-3.48E-10	6.94E-10	0.691708531	0.99999552
A15.382.812.260.Dendritic.Cells	-4.16E-10	8.01E-10	0.697962454	0.99999552
A15.378.316.580.Monocytes	-4.12E-10	7.92E-10	0.698466936	0.99999552
A03.556.124.684.Intestine..Small	-4.31E-10	7.81E-10	0.709559165	0.99999552
Small_Intestine_Terminal_Ileum	-3.91E-10	6.93E-10	0.713596153	0.99999552
A14.549.167.646.Periodontium	-4.18E-10	7.16E-10	0.72038236	0.99999552
A11.118.637.555.567.562.440.Precursor.Cells..B.Lymphoid	-5.00E-10	8.55E-10	0.72048869	0.99999552
A11.872.378.Hematopoietic.Stem.Cells	-5.44E-10	8.61E-10	0.736340697	0.99999552
A02.165.Cartilage	-5.55E-10	8.09E-10	0.753779682	0.99999552
A07.541.358.100.Atrial.Appendage	-4.79E-10	6.92E-10	0.75530953	0.99999552
Bladder	-5.04E-10	7.20E-10	0.75825035	0.99999552
A10.272.497.Epidermis	-5.55E-10	7.91E-10	0.758541422	0.99999552
A15.382.812.522.Macrophages	-5.28E-10	7.44E-10	0.760940178	0.99999552
A11.443.Erythroid.Cells	-5.13E-10	7.12E-10	0.76443188	0.99999552
A07.541.358.Heart.Atria	-5.23E-10	6.79E-10	0.779504501	0.99999552
A03.556.124.369.Intestinal.Mucosa	-6.82E-10	8.62E-10	0.785560677	0.99999552
A05.360.490.Germ.Cells	-5.72E-10	7.06E-10	0.791075953	0.99999552
A11.436.348.Hepatocytes	-6.48E-10	7.77E-10	0.7977212	0.99999552
A11.872.378.590.635.Granulocyte.Macrophage.Progenitor.Cells	-7.17E-10	8.51E-10	0.800063142	0.99999552
Nerve_Tibial	-5.80E-10	6.71E-10	0.806365397	0.99999552
A03.556.249.249.356.Colon	-7.58E-10	8.34E-10	0.81819208	0.99999552
A10.615.550.599.Mouth.Mucosa	-7.34E-10	7.92E-10	0.822790207	0.99999552

A11.627.340.360.Granulocyte.Precursor.Cells	-7.51E-10	7.97E-10	0.826930227	0.99999552
Liver	-6.40E-10	6.61E-10	0.833460822	0.99999552
Whole_Blood	-7.55E-10	7.79E-10	0.833559602	0.99999552
Pancreas	-6.37E-10	6.57E-10	0.833892527	0.99999552
A02.835.583.443.800.800.Synovial.Fluid	-8.57E-10	8.59E-10	0.840967899	0.99999552
A14.724.557.Nasopharynx	-7.36E-10	7.26E-10	0.844529107	0.99999552
Adipose_Subcutaneous	-7.08E-10	6.91E-10	0.847201463	0.99999552
A05.360.319.679.690.Myometrium	-8.33E-10	8.06E-10	0.849452978	0.99999552
A10.690.Muscles	-7.84E-10	7.43E-10	0.854122562	0.99999552
A11.436.329.Granulosa.Cells	-8.17E-10	7.68E-10	0.85608069	0.99999552
Artery_Aorta	-6.81E-10	6.27E-10	0.861319366	0.99999552
A03.556.249.124.Ileum	-8.91E-10	8.19E-10	0.861700398	0.99999552
A14.724.Pharynx	-8.03E-10	7.29E-10	0.864619453	0.99999552
Heart_Atrial_Appendage	-7.56E-10	6.70E-10	0.870343296	0.99999552
A03.556.124.526.767.Rectum	-9.26E-10	8.19E-10	0.871035759	0.99999552
Esophagus_Gastroesophageal_Junction	-8.06E-10	7.05E-10	0.873428413	0.99999552
A15.382.520.604.800.Palatine.Tonsil	-9.91E-10	8.67E-10	0.873623922	0.99999552
A10.165.450.300.425.Keloid	-9.34E-10	7.65E-10	0.889000378	0.99999552
A04.411.Lung	-1.08E-09	8.64E-10	0.893932153	0.99999552
A11.872.190.Embryonic.Stem.Cells	-9.47E-10	7.58E-10	0.894215986	0.99999552
Cervix_Ectocervix	-7.05E-10	5.63E-10	0.894872975	0.99999552
Artery_Tibial	-8.87E-10	7.02E-10	0.896848208	0.99999552
A05.810.453.Kidney	-8.88E-10	6.73E-10	0.906470753	0.99999552
A14.549.Mouth	-9.79E-10	7.31E-10	0.909561334	0.99999552
A11.627.624.249.Monocyte.Macrophage.Precursor.Cells	-1.04E-09	7.65E-10	0.912952699	0.99999552
A11.497.497.600.Oocytes	-9.58E-10	7.00E-10	0.914529771	0.99999552
A10.165.114.830.500.750.Subcutaneous.Fat..Abdominal	-1.07E-09	7.69E-10	0.917272287	0.99999552
A03.556.875.500.Esophagus	-1.06E-09	7.54E-10	0.919991983	0.99999552
A07.541.Heart	-9.42E-10	6.61E-10	0.922734847	0.99999552

A03.556.124.Intestines	-1.15E-09	8.05E-10	0.923913408	0.99999552
Stomach	-1.04E-09	7.24E-10	0.92417792	0.99999552
A11.627.635.Myeloid.Progenitor.Cells	-1.16E-09	8.06E-10	0.925132965	0.99999552
A15.382.520.604.700.Spleen	-1.10E-09	7.49E-10	0.929156888	0.99999552
A10.165.450.300.Cicatrix	-1.10E-09	7.37E-10	0.93201845	0.99999552
A05.360.444.492.362.Foreskin	-1.09E-09	7.31E-10	0.932082852	0.99999552
A03.556.249.249.209.Cecum	-1.15E-09	7.57E-10	0.935945757	0.99999552
A07.541.560.Heart.Ventricles	-1.03E-09	6.73E-10	0.936074681	0.99999552
Minor_Salivary_Gland	-1.08E-09	6.97E-10	0.939406991	0.99999552
A11.329.228.Fibroblasts	-1.23E-09	7.62E-10	0.946190724	0.99999552
Cells_Transformed_fibroblasts	-1.18E-09	7.17E-10	0.9497002	0.99999552
A17.815.Skin	-1.25E-09	7.44E-10	0.953427224	0.99999552
A03.556.249.249.356.668.Colon..Sigmoid	-1.28E-09	7.61E-10	0.954237894	0.99999552
Esophagus_Mucosa	-1.13E-09	6.60E-10	0.95647179	0.99999552
Vagina	-9.47E-10	5.51E-10	0.957315101	0.99999552
Kidney_Cortex	-1.24E-09	7.16E-10	0.957957174	0.99999552
A05.360.319.887.Vulva	-1.31E-09	7.15E-10	0.96688432	0.99999552
A10.549.400.Lymph.Nodes	-1.52E-09	8.24E-10	0.967060929	0.99999552
A02.835.583.443.800.Synovial.Membrane	-1.36E-09	7.37E-10	0.967537587	0.99999552
A10.165.114.830.750.Subcutaneous.Fat	-1.36E-09	7.29E-10	0.968656826	0.99999552
A11.872.378.590.817.Megakaryocyte.Erythroid.Progenitor.Cells	-1.34E-09	7.20E-10	0.968737396	0.99999552
A03.734.414.Islets.of.Langerhans	-1.38E-09	7.36E-10	0.969848133	0.99999552
A11.329.Connective.Tissue.Cells	-1.40E-09	7.37E-10	0.971748885	0.99999552
A06.407.312.Gonads	-1.35E-09	7.06E-10	0.971898673	0.99999552
A10.615.789.Serous.Membrane	-1.52E-09	7.97E-10	0.972123166	0.99999552
Breast_Mammary_Tissue	-1.25E-09	6.51E-10	0.972816906	0.99999552
Cervix_Endocervix	-1.35E-09	6.97E-10	0.973341912	0.99999552
A02.835.232.834.151.Cervical.Vertebrae	-1.46E-09	7.46E-10	0.975116115	0.99999552
A06.407.Endocrine.Glands	-1.39E-09	6.91E-10	0.977781092	0.99999552

A05.810.453.324.Kidney.Cortex	-1.52E-09	7.51E-10	0.978258514	0.99999552
A05.360.319.114.373.Fallopian.Tubes	-1.48E-09	7.28E-10	0.979039976	0.99999552
A05.360.319.679.490.Endometrium	-1.42E-09	6.96E-10	0.979429689	0.99999552
A11.382.Endocrine.Cells	-1.54E-09	7.44E-10	0.98097766	0.99999552
A10.272.Epithelium	-1.40E-09	6.65E-10	0.982005883	0.99999552
A10.336.707.Prostate	-1.39E-09	6.45E-10	0.9845563	0.99999552
Artery_Coronary	-1.42E-09	6.57E-10	0.984688818	0.99999552
A05.360.319.114.630.Ovary	-1.62E-09	7.38E-10	0.985809819	0.99999552
A15.382.490.555.567.537.Killer.Cells..Natural	-1.64E-09	7.40E-10	0.986595047	0.99999552
Adipose_Visceral_(Omentum)	-1.52E-09	6.79E-10	0.987430608	0.99999552
A06.407.312.782.Testis	-1.58E-09	6.95E-10	0.988653614	0.99999552
A03.556.Gastrointestinal.Tract	-1.77E-09	7.59E-10	0.990210803	0.99999552
A10.549.Lymphoid.Tissue	-1.91E-09	8.06E-10	0.99108845	0.99999552
A04.531.520.Nasal.Mucosa	-1.59E-09	6.64E-10	0.991753731	0.99999552
A14.549.885.Tongue	-1.80E-09	7.42E-10	0.992238927	0.99999552
A11.329.171.Chondrocytes	-1.89E-09	7.70E-10	0.993014414	0.99999552
A11.329.114.Adipocytes	-1.90E-09	7.70E-10	0.993117298	0.99999552
A11.872.040.Adult.Stem.Cells	-1.84E-09	7.40E-10	0.993479998	0.99999552
A11.872.Stem.Cells	-1.99E-09	7.76E-10	0.994799001	0.99999552
A03.620.Liver	-1.74E-09	6.54E-10	0.996136892	0.99999552
A11.872.580.Mesenchymal.Stem.Cells	-2.09E-09	7.76E-10	0.996455702	0.99999552
A11.329.372.600.Macrophages..Alveolar	-1.84E-09	6.80E-10	0.996562866	0.99999552
Cells_EBV-transformed_lymphocytes	-2.04E-09	7.45E-10	0.996887983	0.99999552
A15.382.490.555.567.622.Lymphocytes..Null	-1.87E-09	6.82E-10	0.996916356	0.99999552
A11.436.294.064.Glucagon.Secreting.Cells	-2.06E-09	7.45E-10	0.997211784	0.99999552
A10.615.Membranes	-2.00E-09	7.19E-10	0.997365916	0.99999552
A05.360.319.679.Uterus	-2.04E-09	7.12E-10	0.997883591	0.99999552
A05.360.319.679.256.Cervix.Uteri	-1.94E-09	6.71E-10	0.99805947	0.99999552
A10.615.550.Mucous.Membrane	-2.15E-09	7.36E-10	0.998240873	0.99999552

A05.810.890.Urinary.Bladder	-2.20E-09	7.40E-10	0.998500963	0.99999552
A05.360.Genitalia	-2.10E-09	6.77E-10	0.999019086	0.99999552
A05.360.319.Genitalia..Female	-2.21E-09	7.03E-10	0.999149499	0.99999552
A11.329.629.Osteoblasts	-2.38E-09	6.84E-10	0.999746463	0.99999552
A05.360.444.Genitalia..Male	-2.48E-09	6.16E-10	0.999970813	0.99999552
A07.541.510.110.Aortic.Valve	-2.82E-09	6.84E-10	0.999981472	0.99999552
A03.734.Pancreas	-2.77E-09	6.23E-10	0.99999552	0.99999552

Abbreviation: FDR, false discovery rate

Supplementary Table 6. Results from the central nervous system (Cahoy) gene expression using LDSC-SEG

**Name**	**Coefficient**	**Coefficient_std_error**	**Coefficient_P_value**	**Coefficient_Pval_FDR**
Neuron	1.78E-09	7.78E-10	0.011112522	0.033337566
Oligodendrocyte	1.10E-10	8.24E-10	0.447080189	0.670620284
Astrocyte	-4.48E-10	7.08E-10	0.736366878	0.736366878

Abbreviation: FDR, false discovery rate

Supplementary Table 7. Results from multiple-tissue analysis of chromatin data (validation) using LDSC-SEG

Name	Coefficient	Coefficient_std_error	Coefficient_P_value	Coefficient_Pval_FDR
Fetal_Brain_Male__DNase	1.12E-07	1.38E-08	2.23E-16	1.09E-13
Fetal_Brain_Female__DNase	1.23E-07	1.59E-08	4.85E-15	1.19E-12
Fetal_Brain_Male__H3K4me1	4.89E-08	6.82E-09	3.74E-13	6.09E-11
Fetal_Brain_Female__H3K4me3	1.77E-07	2.81E-08	1.43E-10	1.75E-08
Brain_Dorsolateral_Prefrontal_Cortex__H3K27ac	4.73E-08	8.17E-09	3.51E-09	3.04E-07
Fetal_Brain_Female__H3K4me1	6.50E-08	1.13E-08	3.73E-09	3.04E-07
Brain_Germinal_Matrix__H3K4me3	1.82E-07	3.29E-08	1.50E-08	1.05E-06
Brain_Dorsolateral_Prefrontal_Cortex__H3K4me1	5.79E-08	1.05E-08	1.78E-08	1.09E-06
Ganglion_Eminence_derived_primary_cultured_neurospheres__H3K4me3	1.31E-07	2.41E-08	2.89E-08	1.57E-06
Brain_Inferior_Temporal_Lobe__H3K27ac	3.08E-08	6.26E-09	4.26E-07	2.08E-05
Brain_Dorsolateral_Prefrontal_Cortex__H3K9ac	9.68E-08	1.99E-08	5.72E-07	2.54E-05
Cortex_derived_primary_cultured_neurospheres__H3K4me3	1.69E-07	3.54E-08	8.59E-07	3.50E-05
Brain_Angular_Gyrus__H3K27ac	3.50E-08	7.56E-09	1.78E-06	6.70E-05
Brain_Angular_Gyrus__H3K9ac	7.24E-08	1.60E-08	3.01E-06	0.000105181
Brain_Dorsolateral_Prefrontal_Cortex__H3K4me3	1.12E-07	2.51E-08	4.02E-06	0.00013092
Brain_Germinal_Matrix__H3K4me1	7.05E-08	1.61E-08	5.88E-06	0.000179741
Brain_Anterior_Caudate__H3K27ac	2.68E-08	6.19E-09	7.13E-06	0.000205221
Brain_Angular_Gyrus__H3K4me1	3.67E-08	8.61E-09	1.00E-05	0.000272699
Brain_Anterior_Caudate__H3K9ac	6.20E-08	1.46E-08	1.15E-05	0.000295453
Brain_Cingulate_Gyrus__H3K9ac	6.78E-08	1.61E-08	1.25E-05	0.000305202
Brain_Inferior_Temporal_Lobe__H3K9ac	5.11E-08	1.35E-08	7.68E-05	0.001787729
Brain_Cingulate_Gyrus__H3K27ac	2.53E-08	6.75E-09	9.15E-05	0.00203269
Brain_Anterior_Caudate__H3K4me1	2.84E-08	7.78E-09	0.000130151	0.002767121
Brain_Cingulate_Gyrus__H3K4me1	2.64E-08	7.58E-09	0.000243224	0.004955696
Brain_Inferior_Temporal_Lobe__H3K4me1	3.19E-08	9.18E-09	0.000258285	0.005052059
Brain_Hippocampus_Middle__H3K27ac	1.99E-08	5.84E-09	0.000336102	0.006281929
Brain_Inferior_Temporal_Lobe__H3K4me3	8.12E-08	2.39E-08	0.000346855	0.006281929

Brain_Angular_Gyrus__H3K4me3	8.91E-08	2.69E-08	0.000456931	0.007979974
Ganglion_Eminence_derived_primary_cultured_neurospheres__H3K4me1	3.63E-08	1.10E-08	0.000482308	0.008132711
Brain_Anterior_Caudate__H3K4me3	6.63E-08	2.03E-08	0.00055047	0.008972655
Spleen__H3K36me3	2.18E-08	6.80E-09	0.000673089	0.010617428
Brain_Hippocampus_Middle__H3K4me1	1.94E-08	6.24E-09	0.000921427	0.014080552
Esoph-GJ_ENTEX__H3K4me3	5.99E-08	1.97E-08	0.001213573	0.017982951
Esoph-Muscularis_ENTEX__H3K27ac	1.93E-08	6.51E-09	0.001485833	0.021369772
Cortex_derived_primary_cultured_neurospheres__H3K4me1	2.09E-08	7.13E-09	0.001687123	0.023571522
Esoph-Mucosa_ENTEX__H3K36me3	2.69E-08	9.25E-09	0.001833483	0.024904804
Brain_Cingulate_Gyrus__H3K4me3	6.99E-08	2.54E-08	0.002974074	0.039306004
Esoph-GJ_ENTEX__H3K27ac	1.76E-08	6.44E-09	0.00312831	0.040256406
Lung__H3K36me3	2.77E-08	1.02E-08	0.003385847	0.042453309
Spleen__H3K4me1	2.21E-08	8.26E-09	0.00371917	0.045466858
Foreskin_Melanocyte_Primary_Cells_skin01__H3K36me3	3.16E-08	1.21E-08	0.00444596	0.053026205
Esophagus__H3K36me3	2.48E-08	9.67E-09	0.005164072	0.060124553
Colon-Sigm_ENTEX__H3K27ac	1.80E-08	7.07E-09	0.005431623	0.061768927
Foreskin_Fibroblast_Primary_Cells_skin02__H3K4me3	2.37E-08	9.40E-09	0.005922148	0.065816596
Thymus__H3K36me3	2.96E-08	1.19E-08	0.006495944	0.070589261
Foreskin_Melanocyte_Primary_Cells_skin01__H3K4me3	6.24E-08	2.60E-08	0.00821275	0.085381507
Esoph-Muscularis_ENTEX__H3K4me3	5.35E-08	2.24E-08	0.008333763	0.085381507
Right_Ventricle__H3K36me3	2.25E-08	9.40E-09	0.008381007	0.085381507
Foreskin_Fibroblast_Primary_Cells_skin01__H3K4me3	2.51E-08	1.11E-08	0.011676446	0.116526163
Brain_Dorsolateral_Prefrontal_Cortex__H3K36me3	2.13E-08	9.48E-09	0.012269227	0.116965405
Esoph-Muscularis_ENTEX__H3K4me1	1.33E-08	5.93E-09	0.012301177	0.116965405
Brain_Substantia_Nigra__H3K27ac	1.24E-08	5.54E-09	0.012438039	0.116965405
Brain_Substantia_Nigra__H3K9ac	3.42E-08	1.53E-08	0.012861089	0.118661743
Brain_Cingulate_Gyrus__H3K36me3	2.07E-08	9.36E-09	0.013426961	0.120421788
Foreskin_Fibroblast_Primary_Cells_skin02__H3K36me3	1.18E-08	5.36E-09	0.013695819	0.120421788
Colon-Sigm_ENTEX__H3K36me3	2.19E-08	9.93E-09	0.013790634	0.120421788

Psoas_Muscle__DNase	2.81E-08	1.28E-08	0.014209047	0.121898669
Fetal_Brain_Female__H3K36me3	1.74E-08	8.03E-09	0.014924025	0.125824973
Gastric__H3K36me3	2.00E-08	9.32E-09	0.016187919	0.134167671
Fetal_Thymus__DNase	2.96E-08	1.39E-08	0.017031471	0.138747726
Brain_Germinal_Matrix__H3K36me3	2.21E-08	1.05E-08	0.017567692	0.138747726
NHLF_Lung_Fibroblast_Primary_Cells__H3K36me3	2.73E-08	1.30E-08	0.017591736	0.138747726
Brain_Substantia_Nigra__H3K4me1	1.45E-08	6.95E-09	0.018530376	0.143831011
Heart-LV_ENTEX__H3K27ac	1.08E-08	5.26E-09	0.01960952	0.149170577
Skeletal_Muscle_Male__H3K36me3	1.89E-08	9.20E-09	0.0198284	0.149170577
skeletal_muscle_ENTEX__H3K4me1	7.07E-09	3.46E-09	0.020611292	0.152710933
Foreskin_Keratinocyte_Primary_Cells_skin02__H3K36me3	1.27E-08	6.29E-09	0.02192876	0.160047215
skeletal_muscle_ENTEX__H3K27ac	5.78E-09	2.95E-09	0.025023715	0.179949951
Stomach_Smooth_Muscle__H3K4me3	2.99E-08	1.53E-08	0.025640391	0.181712337
Spleen__H3K4me3	3.44E-08	1.79E-08	0.027144238	0.189621891
Foreskin_Melanocyte_Primary_Cells_skin01__H3K27ac	3.19E-08	1.68E-08	0.028921043	0.199188593
Brain_Inferior_Temporal_Lobe__H3K36me3	1.52E-08	8.29E-09	0.033008883	0.224185333
Nerve-Tibial_ENTEX__H3K4me3	4.86E-08	2.68E-08	0.035148711	0.233053583
Brain_Hippocampus_Middle__H3K4me3	3.32E-08	1.83E-08	0.035267822	0.233053583
skeletal_muscle_ENTEX__H3K4me3	1.98E-08	1.15E-08	0.043419629	0.283095983
Esoph-GJ_ENTEX__H3K4me1	1.14E-08	6.68E-09	0.044246048	0.284688387
Cortex_derived_primary_cultured_neurospheres__H3K36me3	1.42E-08	8.47E-09	0.046289613	0.293969103
Lung_ENTEX__H3K36me3	8.96E-09	5.44E-09	0.049822333	0.312347701
Foreskin_Keratinocyte_Primary_Cells_skin02__H3K4me3	3.46E-08	2.12E-08	0.051069076	0.316111116
Heart-LV_ENTEX__H3K4me1	1.42E-08	9.11E-09	0.059805411	0.365560576
Foreskin_Melanocyte_Primary_Cells_skin01__H3K4me1	2.14E-08	1.38E-08	0.060644501	0.366113097
Stomach_Smooth_Muscle__H3K27ac	9.33E-09	6.19E-09	0.06579808	0.392381233
Thyroid_gland_ENTEX__H3K36me3	9.88E-09	6.63E-09	0.067890288	0.39998013
Ganglion_Eminence_derived_primary_cultured_neurospheres__H3K36me3	1.18E-08	8.30E-09	0.078355354	0.453358647
Nerve-Tibial_ENTEX__H3K4me1	8.05E-09	5.70E-09	0.078804673	0.453358647

Heart-Atrial_ENTEX__H3K36me3	1.01E-08	7.22E-09	0.080749878	0.459147563
Psoas_Muscle__H3K27ac	1.31E-08	9.48E-09	0.084008193	0.472183983
Pancreatic_Islets__H3K9ac	5.12E-08	3.88E-08	0.09335949	0.517168549
Pancreatic_Islets__H3K27ac	2.29E-08	1.74E-08	0.094126791	0.517168549
Brain_Hippocampus_Middle__H3K36me3	1.03E-08	7.96E-09	0.098476391	0.533851684
Primary_T_cells_effector_memory_enriched_from_peripheral_blood__H3K36me3	9.38E-09	7.30E-09	0.099346632	0.533851684
Brain_Angular_Gyrus__H3K36me3	1.29E-08	1.02E-08	0.103080285	0.547894122
Lung__H3K4me3	3.74E-08	3.04E-08	0.108971975	0.572981673
Skeletal_Muscle_Male__H3K4me1	6.96E-09	5.78E-09	0.114161342	0.587723804
Fetal_Stomach__H3K36me3	5.70E-09	4.74E-09	0.114522211	0.587723804
Pancreas__H3K36me3	1.20E-08	1.00E-08	0.116397786	0.587723804
Brain_Substantia_Nigra__H3K4me3	2.62E-08	2.20E-08	0.11658325	0.587723804
Colonic_Mucosa__H3K4me3	2.68E-08	2.27E-08	0.118385814	0.590721052
Fetal_Muscle_Trunk__H3K4me1	6.74E-09	5.75E-09	0.120561995	0.595503188
Mammary_ENTEX__H3K36me3	6.92E-09	5.94E-09	0.122150244	0.597314691
Fetal_Thymus__H3K4me1	6.67E-09	6.01E-09	0.13362829	0.646972613
Right_Ventricle__H3K4me1	1.05E-08	9.53E-09	0.136440836	0.654113422
Psoas_Muscle__H3K4me3	3.65E-08	3.34E-08	0.137811687	0.654271019
Placenta__H3K36me3	9.70E-09	9.02E-09	0.14119194	0.663873642
Primary_T_helper_naive_cells_from_peripheral_blood_2__H3K36me3	7.30E-09	6.84E-09	0.142986906	0.665910447
Fetal_Brain_Male__H3K4me3	6.09E-08	5.85E-08	0.14887639	0.686105441
Stomach_Smooth_Muscle__H3K4me1	7.30E-09	7.05E-09	0.150129411	0.686105441
Fetal_Thymus__H3K36me3	5.34E-09	5.24E-09	0.1541286	0.697860051
Right_Ventricle__H3K4me3	2.47E-08	2.47E-08	0.158495518	0.71035039
Primary_T_cells_from_peripheral_blood__H3K36me3	5.44E-09	5.47E-09	0.159792521	0.71035039
Skeletal_Muscle_Male__H3K9ac	8.07E-09	8.31E-09	0.165910609	0.721244005
Right_Atrium__H3K4me3	2.72E-08	2.82E-08	0.166757508	0.721244005
Brain_Substantia_Nigra__H3K36me3	1.31E-08	1.36E-08	0.167143214	0.721244005
Nerve-Tibial_ENTEX__H3K27ac	3.96E-09	4.12E-09	0.168142774	0.721244005

Foreskin_Fibroblast_Primary_Cells_skin01__H3K36me3	4.50E-09	4.71E-09	0.16982906	0.722142698
Primary_B_cells_from_peripheral_blood__DNase	1.42E-08	1.51E-08	0.173433932	0.72953453
Colon_Smooth_Muscle__H3K27ac	6.21E-09	6.63E-09	0.174551206	0.72953453
Primary_hematopoietic_stem_cells_G-CSF-mobilized_Female__DNase	1.41E-08	1.53E-08	0.177915406	0.736075191
Stomach_Smooth_Muscle__H3K9ac	1.77E-08	1.92E-08	0.179126683	0.736075191
Foreskin_Melanocyte_Primary_Cells_skin03__H3K4me3	2.56E-08	2.82E-08	0.181914694	0.741302377
Primary_T_cells_from_cord_blood__DNase	1.56E-08	1.73E-08	0.183443185	0.741353037
Placenta_Amnion__H3K36me3	6.93E-09	7.76E-09	0.185899036	0.745119907
Ovary__H3K36me3	8.74E-09	9.86E-09	0.187782768	0.745299149
NHEK-Epidermal_Keratinocyte_Primary_Cells__H3K36me3	5.96E-09	6.76E-09	0.188992013	0.745299149
Heart-Atrial_ENTEX__H3K27ac	4.01E-09	4.66E-09	0.194770865	0.756583757
Fetal_Adrenal_Gland__H3K4me3	3.03E-08	3.56E-08	0.197098302	0.756583757
Artery-Coronary_ENTEX__H3K27ac	4.42E-09	5.20E-09	0.197699755	0.756583757
Rectal_Smooth_Muscle__H3K27ac	5.99E-09	7.05E-09	0.198042374	0.756583757
Foreskin_Keratinocyte_Primary_Cells_skin03__H3K4me3	1.36E-08	1.70E-08	0.212002863	0.800042291
Fetal_Muscle_Trunk__H3K4me3	2.37E-08	2.98E-08	0.21269018	0.800042291
Adrenal_gland_ENTEX__H3K4me3	1.29E-08	1.65E-08	0.216776615	0.809189043
Aorta_ENTEX__H3K36me3	4.10E-09	5.32E-09	0.220373687	0.81638434
Primary_T_cells_from_peripheral_blood__H3K4me1	5.08E-09	6.68E-09	0.223377879	0.81778251
Primary_T_cells_effector_memory_enriched_from_peripheral_blood__H3K4me1	7.20E-09	9.62E-09	0.227001639	0.81778251
Pancreas__H3K4me3	2.63E-08	3.53E-08	0.228023708	0.81778251
Left_Ventricle__H3K4me3	2.26E-08	3.05E-08	0.229001497	0.81778251
Pancreatic_Islets__H3K4me1	5.54E-09	7.46E-09	0.229112891	0.81778251
Fetal_Adrenal_Gland__DNase	1.16E-08	1.60E-08	0.235545324	0.834649734
Gastric__H3K4me3	1.74E-08	2.44E-08	0.237289373	0.834780601
Heart-LV_ENTEX__H3K36me3	5.63E-09	7.97E-09	0.239978154	0.838209408
Pancreatic_Islets__H3K4me3	2.34E-08	3.47E-08	0.249824955	0.866414207
Adrenal_gland_ENTEX__H3K27ac	3.48E-09	5.23E-09	0.253012637	0.866802121
Esoph-Muscularis_ENTEX__H3K36me3	4.48E-09	6.75E-09	0.253482011	0.866802121

Primary_mononuclear_cells_from_peripheral_blood__H3K36me3	3.60E-09	5.60E-09	0.259829005	0.878326912
Foreskin_Fibroblast_Primary_Cells_skin02__DNase	8.15E-09	1.28E-08	0.261414545	0.878326912
Primary_Natural_Killer_cells_from_peripheral_blood__H3K4me1	3.69E-09	5.84E-09	0.263504219	0.878326912
Thymus__H3K4me1	5.91E-09	9.37E-09	0.264036924	0.878326912
Lung_ENTEX__H3K27ac	3.10E-09	5.17E-09	0.274612049	0.907333055
Primary_hematopoietic_stem_cells_G-CSF-mobilized_Male__DNase	1.05E-08	1.91E-08	0.2912916	0.955342291
Skeletal_Muscle_Female__H3K9ac	6.82E-09	1.25E-08	0.293049783	0.955342291
Right_Ventricle__H3K27ac	4.24E-09	9.36E-09	0.325376371	0.999871426
Spleen_ENTEX__H3K27ac	1.72E-09	3.84E-09	0.326650754	0.999871426
Duodenum_Smooth_Muscle__H3K4me1	4.39E-09	9.79E-09	0.326937753	0.999871426
Esophagus__H3K4me3	1.14E-08	2.73E-08	0.338448162	0.999871426
Primary_T_helper_cells_from_peripheral_blood__H3K36me3	1.97E-09	4.80E-09	0.340668393	0.999871426
Osteoblast_Primary_Cells__H3K36me3	2.30E-09	5.64E-09	0.341811993	0.999871426
Osteoblast_Primary_Cells__H3K4me3	6.67E-09	1.64E-08	0.342274214	0.999871426
Primary_T_cells_from_cord_blood__H3K36me3	4.87E-09	1.28E-08	0.351346451	0.999871426
Ovary__DNase	4.38E-09	1.18E-08	0.354547126	0.999871426
Stomach_Smooth_Muscle__H3K36me3	2.85E-09	7.68E-09	0.355208582	0.999871426
Colon-TV_ENTEX__H3K4me1	5.23E-09	1.46E-08	0.360072268	0.999871426
Fetal_Thymus__H3K27ac	2.68E-09	8.03E-09	0.369288794	0.999871426
Foreskin_Fibroblast_Primary_Cells_skin02__H3K4me1	1.81E-09	5.47E-09	0.370249022	0.999871426
Primary_T_helper_naive_cells_from_peripheral_blood_2__H3K4me1	1.83E-09	6.13E-09	0.382454178	0.999871426
Pancreas_ENTEX__H3K4me3	6.95E-09	2.33E-08	0.383050075	0.999871426
Foreskin_Melanocyte_Primary_Cells_skin03__H3K27ac	2.62E-09	8.87E-09	0.383981085	0.999871426
Fetal_Kidney__DNase	4.39E-09	1.52E-08	0.386544974	0.999871426
Skeletal_Muscle_Male__H3K4me3	4.62E-09	1.61E-08	0.387385454	0.999871426
Colonic_Mucosa__H3K36me3	3.43E-09	1.21E-08	0.388068273	0.999871426
Colon-Sigm_ENTEX__H3K4me3	9.19E-09	3.25E-08	0.388589031	0.999871426
Lung__H3K27ac	2.43E-09	9.03E-09	0.393972517	0.999871426
skeletal_muscle_ENTEX__H3K36me3	1.13E-09	4.96E-09	0.409905312	0.999871426

Fetal_Adrenal_Gland__H3K4me1	1.57E-09	7.14E-09	0.412782916	0.999871426
Fetal_Muscle_Trunk__H3K36me3	1.65E-09	7.96E-09	0.418004709	0.999871426
Skeletal_Muscle_Female__H3K4me3	3.09E-09	1.50E-08	0.418348321	0.999871426
Primary_T_helper_naive_cells_from_peripheral_blood_1__H3K36me3	1.56E-09	7.90E-09	0.421512899	0.999871426
Fetal_Muscle_Leg__H3K4me3	5.57E-09	2.97E-08	0.425671635	0.999871426
Primary_T_helper_cells_PMA-I_stimulated__H3K36me3	9.89E-10	5.37E-09	0.426974122	0.999871426
Primary_T_cells_from_peripheral_blood__DNase	2.88E-09	1.57E-08	0.427200296	0.999871426
Primary_T_regulatory_cells_from_peripheral_blood__H3K36me3	9.40E-10	5.18E-09	0.427946315	0.999871426
Heart-Atrial_ENTEX__H3K4me3	4.13E-09	2.37E-08	0.430750901	0.999871426
Heart-LV_ENTEX__H3K4me3	2.91E-09	1.95E-08	0.440863316	0.999871426
Vagina_ENTEX__H3K4me3	2.79E-09	1.98E-08	0.444046389	0.999871426
Artery-Coronary_ENTEX__H3K4me3	1.46E-09	1.22E-08	0.452207598	0.999871426
Primary_B_cells_from_peripheral_blood__H3K27ac	1.11E-09	1.01E-08	0.456235195	0.999871426
Testis_ENTEX__H3K27ac	5.06E-10	4.70E-09	0.457141047	0.999871426
Primary_hematopoietic_stem_cells__H3K4me1	1.22E-09	1.15E-08	0.457588688	0.999871426
Skeletal_Muscle_Female__H3K27ac	6.14E-10	5.89E-09	0.458457515	0.999871426
Fetal_Muscle_Leg__H3K36me3	6.42E-10	7.17E-09	0.464357409	0.999871426
Thymus__H3K4me3	2.34E-09	2.69E-08	0.465353419	0.999871426
Primary_T_killer_memory_cells_from_peripheral_blood__H3K36me3	7.35E-10	9.57E-09	0.469403923	0.999871426
Aorta__H3K4me3	1.42E-09	2.85E-08	0.480061803	0.999871426
Spleen__H3K27ac	2.58E-10	8.46E-09	0.487839958	0.999871426
Fetal_Stomach__H3K4me3	5.22E-10	3.05E-08	0.493163778	0.999871426
Lung_ENTEX__H3K4me1	1.01E-10	6.11E-09	0.493403074	0.999871426
Foreskin_Keratinocyte_Primary_Cells_skin03__H3K36me3	4.10E-11	4.17E-09	0.49607552	0.999871426
Colon_Smooth_Muscle__H3K9ac	1.54E-10	1.74E-08	0.496479797	0.999871426
Aorta_ENTEX__H3K4me3	6.58E-11	1.04E-08	0.497473992	0.999871426
Primary_T_helper_cells_PMA-I_stimulated__H3K4me1	-1.34E-10	5.00E-09	0.510730235	0.999871426
Primary_T_helper_cells_from_peripheral_blood__H3K4me1	-2.05E-10	5.55E-09	0.514699343	0.999871426
Skeletal_Muscle_Female__H3K36me3	-6.09E-10	7.60E-09	0.531928497	0.999871426

Esoph-Mucosa_ENTEX__H3K4me3	-1.35E-09	1.68E-08	0.531947925	0.999871426
Primary_B_cells_from_peripheral_blood__H3K4me1	-5.70E-10	5.84E-09	0.5389006	0.999871426
Right_Atrium__H3K27ac	-9.11E-10	9.27E-09	0.539170623	0.999871426
Foreskin_Fibroblast_Primary_Cells_skin02__H3K27ac	-7.83E-10	7.18E-09	0.543407716	0.999871426
Breast_Myoepithelial_Primary_Cells__H3K9ac	-1.99E-09	1.74E-08	0.545542291	0.999871426
Primary_T_helper_cells_from_peripheral_blood__H3K27ac	-1.41E-09	1.19E-08	0.547221597	0.999871426
Left_Ventricle__H3K27ac	-1.04E-09	7.33E-09	0.556477932	0.999871426
Thymus__H3K27ac	-2.08E-09	1.43E-08	0.557828954	0.999871426
Primary_T_killer_naive_cells_from_peripheral_blood__H3K4me1	-1.04E-09	5.97E-09	0.568830786	0.999871426
Thyroid_gland_ENTEX__H3K4me3	-4.38E-09	2.24E-08	0.577369695	0.999871426
Primary_hematopoietic_stem_cells_G-CSF-mobilized_Male__H3K4me3	-3.06E-09	1.53E-08	0.579534944	0.999871426
Primary_hematopoietic_stem_cells_short_term_culture__H3K36me3	-1.47E-09	7.27E-09	0.579818832	0.999871426
Fetal_Adrenal_Gland__H3K36me3	-1.30E-09	6.32E-09	0.581417756	0.999871426
NHLF_Lung_Fibroblast_Primary_Cells__DNase	-2.81E-09	1.36E-08	0.581726576	0.999871426
Stomach_Mucosa__H3K36me3	-3.77E-09	1.71E-08	0.587095417	0.999871426
Primary_T_helper_memory_cells_from_peripheral_blood_1__H3K4me1	-1.37E-09	5.96E-09	0.591129827	0.999871426
Rectal_Smooth_Muscle__H3K4me1	-2.08E-09	9.00E-09	0.591430556	0.999871426
Testis_ENTEX__H3K4me3	-2.98E-09	1.24E-08	0.595342763	0.999871426
Colon_Smooth_Muscle__H3K36me3	-2.16E-09	8.70E-09	0.597927927	0.999871426
Fetal_Muscle_Trunk__DNase	-3.18E-09	1.24E-08	0.601029335	0.999871426
Spleen_ENTEX__H3K36me3	-1.01E-09	3.87E-09	0.60277331	0.999871426
Foreskin_Melanocyte_Primary_Cells_skin03__H3K36me3	-1.38E-09	5.17E-09	0.605243174	0.999871426
Fetal_Stomach__DNase	-5.02E-09	1.69E-08	0.616568189	0.999871426
Skeletal_Muscle_Female__H3K4me1	-1.51E-09	4.80E-09	0.623478342	0.999871426
Pancreas__DNase	-7.14E-09	2.21E-08	0.626737649	0.999871426
Primary_T_cells_effector_memory_enriched_from_peripheral_blood__H3K27ac	-3.37E-09	1.04E-08	0.626858032	0.999871426
Primary_T_helper_17_cells_PMA-I_stimulated__H3K36me3	-1.94E-09	5.95E-09	0.627612208	0.999871426
Primary_monocytes_from_peripheral_blood__DNase	-4.53E-09	1.38E-08	0.628456862	0.999871426
Right_Atrium__H3K36me3	-5.56E-09	1.67E-08	0.630056325	0.999871426

Colonic_Mucosa__H3K4me1	-3.99E-09	1.20E-08	0.630511088	0.999871426
Vagina_ENTEX__H3K27ac	-1.86E-09	5.50E-09	0.632296816	0.999871426
Primary_T_cells_from_cord_blood__H3K4me1	-4.23E-09	1.22E-08	0.635290163	0.999871426
Fetal_Intestine_Small__H3K36me3	-1.47E-09	4.23E-09	0.635732648	0.999871426
Mammary_ENTEX__H3K4me3	-5.20E-09	1.44E-08	0.641069857	0.999871426
NHLF_Lung_Fibroblast_Primary_Cells__H3K9ac	-9.25E-09	2.56E-08	0.641298866	0.999871426
Primary_hematopoietic_stem_cells_G-CSF-mobilized_Female__H3K4me1	-1.92E-09	5.28E-09	0.641961021	0.999871426
Primary_T_helper_cells_PMA-I_stimulated__H3K27ac	-1.91E-09	5.23E-09	0.642488927	0.999871426
Fetal_Thymus__H3K4me3	-1.05E-08	2.73E-08	0.649470636	0.999871426
Primary_T_helper_naive_cells_from_peripheral_blood_2__H3K4me3	-1.08E-08	2.75E-08	0.65197786	0.999871426
Colonic_Mucosa__H3K9ac	-8.71E-09	2.16E-08	0.656681921	0.999871426
Duodenum_Smooth_Muscle__H3K27ac	-4.63E-09	1.12E-08	0.660032099	0.999871426
Foreskin_Fibroblast_Primary_Cells_skin01__H3K27ac	-3.06E-09	7.33E-09	0.661589043	0.999871426
NHEK-Epidermal_Keratinocyte_Primary_Cells__H3K4me3	-7.08E-09	1.66E-08	0.665286332	0.999871426
Adrenal_gland_ENTEX__H3K4me1	-2.83E-09	6.63E-09	0.665534811	0.999871426
Gastric__H3K4me1	-5.18E-09	1.21E-08	0.665991389	0.999871426
Rectal_Smooth_Muscle__H3K4me3	-1.07E-08	2.47E-08	0.668119186	0.999871426
Primary_T_helper_memory_cells_from_peripheral_blood_2__H3K4me1	-2.56E-09	5.53E-09	0.678482987	0.999871426
Thyroid_gland_ENTEX__H3K4me1	-1.89E-09	4.03E-09	0.680584766	0.999871426
Primary_T_helper_naive_cells_from_peripheral_blood_1__H3K4me1	-3.18E-09	6.70E-09	0.68266559	0.999871426
Placenta__H3K4me1	-3.42E-09	6.97E-09	0.688474083	0.999871426
Fetal_Muscle_Leg__H3K4me1	-2.76E-09	5.51E-09	0.691846997	0.999871426
Adipose_Nuclei__H3K4me1	-3.06E-09	6.11E-09	0.69195208	0.999871426
Primary_T_helper_17_cells_PMA-I_stimulated__H3K27ac	-5.09E-09	1.01E-08	0.692917664	0.999871426
Aorta_ENTEX__H3K4me1	-1.67E-09	3.31E-09	0.692920369	0.999871426
Small_Intestine__H3K36me3	-9.65E-09	1.91E-08	0.693675248	0.999871426
Fetal_Heart__DNase	-5.04E-09	9.76E-09	0.697454399	0.999871426
Primary_T_helper_naive_cells_from_peripheral_blood_2__H3K27ac	-4.40E-09	8.47E-09	0.69815198	0.999871426
liver_ENTEX__H3K27ac	-2.20E-09	4.14E-09	0.702094456	0.999871426

Aorta__H3K36me3	-1.03E-08	1.95E-08	0.702434864	0.999871426
Primary_T_killer_memory_cells_from_peripheral_blood__H3K4me1	-3.90E-09	7.30E-09	0.703412835	0.999871426
Fetal_Stomach__H3K4me1	-3.36E-09	6.22E-09	0.705296906	0.999871426
Foreskin_Melanocyte_Primary_Cells_skin03__H3K4me1	-3.05E-09	5.39E-09	0.714557576	0.999871426
Primary_B_cells_from_cord_blood__H3K4me1	-4.17E-09	7.31E-09	0.715759774	0.999871426
Left_Ventricle__H3K4me1	-5.39E-09	9.45E-09	0.715840249	0.999871426
Primary_T_helper_17_cells_PMA-I_stimulated__H3K4me1	-3.61E-09	6.26E-09	0.718210794	0.999871426
Right_Atrium__H3K4me1	-6.89E-09	1.19E-08	0.718784938	0.999871426
Primary_T_killer_naive_cells_from_peripheral_blood__H3K36me3	-5.83E-09	1.00E-08	0.719630083	0.999871426
Primary_Natural_Killer_cells_from_peripheral_blood__H3K36me3	-3.14E-09	5.38E-09	0.720385685	0.999871426
Primary_T_helper_memory_cells_from_peripheral_blood_1__H3K36me3	-4.77E-09	8.14E-09	0.721191724	0.999871426
Ovary__H3K4me3	-1.39E-08	2.33E-08	0.723896344	0.999871426
Pancreatic_Islets__H3K36me3	-9.39E-09	1.58E-08	0.724356933	0.999871426
Brain_Anterior_Caudate__H3K36me3	-5.26E-09	8.72E-09	0.726820309	0.999871426
Esophagus__H3K4me1	-6.10E-09	1.01E-08	0.727100132	0.999871426
Primary_Natural_Killer_cells_from_peripheral_blood__DNase	-9.19E-09	1.46E-08	0.735923334	0.999871426
Thyroid_gland_ENTEX__H3K27ac	-2.48E-09	3.89E-09	0.738417156	0.999871426
Primary_T_helper_naive_cells_from_peripheral_blood_1__H3K27ac	-1.08E-08	1.68E-08	0.739427919	0.999871426
Stomach_ENTEX__H3K4me3	-1.14E-08	1.77E-08	0.740209524	0.999871426
Artery-Tibial_ENTEX__H3K36me3	-4.10E-09	6.30E-09	0.742139966	0.999871426
Colon_Smooth_Muscle__H3K4me1	-4.20E-09	6.44E-09	0.74296804	0.999871426
Lung_ENTEX__H3K4me3	-1.99E-08	3.01E-08	0.746026631	0.999871426
Fetal_Heart__H3K4me1	-3.33E-09	4.91E-09	0.75139195	0.999871426
liver_ENTEX__H3K4me3	-8.24E-09	1.20E-08	0.754303812	0.999871426
Fetal_Adrenal_Gland__H3K27ac	-4.35E-09	6.32E-09	0.754453261	0.999871426
Foreskin_Fibroblast_Primary_Cells_skin01__DNase	-9.45E-09	1.34E-08	0.760048512	0.999871426
Primary_hematopoietic_stem_cells_short_term_culture__H3K4me3	-1.72E-08	2.43E-08	0.760559636	0.999871426
Primary_hematopoietic_stem_cells_G-CSF-mobilized_Female__H3K27ac	-6.68E-09	9.42E-09	0.760782133	0.999871426
Small_Intestine__H3K27ac	-7.25E-09	1.02E-08	0.76202127	0.999871426

Lung__H3K4me1	-6.54E-09	9.15E-09	0.762774721	0.999871426
Primary_T_helper_memory_cells_from_peripheral_blood_2__H3K36me3	-7.47E-09	1.04E-08	0.763830778	0.999871426
Placenta__H3K27ac	-4.00E-09	5.55E-09	0.764357989	0.999871426
Primary_T_helper_memory_cells_from_peripheral_blood_1__H3K27ac	-8.23E-09	1.13E-08	0.767188717	0.999871426
Aorta_ENTEX__H3K27ac	-2.30E-09	3.14E-09	0.768089727	0.999871426
Liver__H3K27ac	-3.90E-09	5.28E-09	0.770064805	0.999871426
Primary_T_cells_effector_memory_enriched_from_peripheral_blood__H3K4me3	-1.86E-08	2.51E-08	0.771010332	0.999871426
Rectal_Smooth_Muscle__H3K9ac	-2.11E-08	2.82E-08	0.77306902	0.999871426
Ovary__H3K27ac	-7.64E-09	1.01E-08	0.775509491	0.999871426
Primary_B_cells_from_peripheral_blood__H3K36me3	-3.67E-09	4.74E-09	0.781004512	0.999871426
Pancreas__H3K27ac	-1.20E-08	1.54E-08	0.782150785	0.999871426
Fetal_Lung__H3K9ac	-1.56E-08	1.94E-08	0.788496807	0.999871426
Artery-Tibial_ENTEX__H3K4me1	-1.22E-08	1.48E-08	0.795616719	0.999871426
Primary_T_regulatory_cells_from_peripheral_blood__H3K4me1	-5.42E-09	6.55E-09	0.795838962	0.999871426
Skin_tissue_ENTEX__H3K4me3	-2.05E-08	2.44E-08	0.79906263	0.999871426
Foreskin_Fibroblast_Primary_Cells_skin01__H3K4me1	-4.12E-09	4.88E-09	0.800951313	0.999871426
HMEC_Mammary_Epithelial_Primary_Cells__H3K36me3	-8.40E-09	9.79E-09	0.804484572	0.999871426
Placenta_Amnion__H3K27ac	-1.24E-08	1.41E-08	0.811037942	0.999871426
Colon-TV_ENTEX__H3K4me3	-1.83E-08	2.07E-08	0.811993747	0.999871426
Primary_T_killer_naive_cells_from_peripheral_blood__H3K9ac	-2.18E-08	2.46E-08	0.812513886	0.999871426
Primary_hematopoietic_stem_cells_short_term_culture__H3K4me1	-6.06E-09	6.80E-09	0.813824012	0.999871426
Colon_Smooth_Muscle__H3K4me3	-1.66E-08	1.86E-08	0.815002576	0.999871426
NHLF_Lung_Fibroblast_Primary_Cells__H3K4me1	-9.60E-09	1.06E-08	0.816859817	0.999871426
Primary_T_killer_naive_cells_from_peripheral_blood__H3K27ac	-1.62E-08	1.78E-08	0.819175326	0.999871426
Primary_B_cells_from_peripheral_blood__H3K4me3	-2.80E-08	2.94E-08	0.829384803	0.999871426
Colon-TV_ENTEX__H3K36me3	-8.66E-09	8.84E-09	0.836396438	0.999871426
Primary_monocytes_from_peripheral_blood__H3K4me3	-2.65E-08	2.70E-08	0.836731452	0.999871426
Small_Intestine__H3K4me3	-3.02E-08	3.07E-08	0.837226536	0.999871426
Colon-TV_ENTEX__H3K27ac	-5.85E-09	5.80E-09	0.843402683	0.999871426

Left_Ventricle__H3K36me3	-9.60E-09	9.46E-09	0.84492152	0.999871426
Primary_T_helper_naive_cells_from_peripheral_blood__H3K9ac	-2.40E-08	2.32E-08	0.849140791	0.999871426
Primary_T_regulatory_cells_from_peripheral_blood__H3K4me3	-1.77E-08	1.72E-08	0.84949204	0.999871426
Pancreas__H3K4me1	-8.07E-09	7.73E-09	0.851778647	0.999871426
Prostate_ENTEX__H3K4me3	-3.31E-08	3.15E-08	0.852847273	0.999871426
Fetal_Intestine_Small__H3K4me3	-2.44E-08	2.33E-08	0.853148625	0.999871426
Primary_T_regulatory_cells_from_peripheral_blood__H3K27ac	-7.29E-09	6.89E-09	0.854950022	0.999871426
Stomach_ENTEX__H3K4me1	-7.83E-09	7.38E-09	0.855368991	0.999871426
Primary_hematopoietic_stem_cells_G-CSF-mobilized_Male__H3K4me1	-6.11E-09	5.76E-09	0.855745707	0.999871426
Aorta__H3K27ac	-7.89E-09	7.42E-09	0.856282331	0.999871426
Primary_neutrophils_from_peripheral_blood__H3K36me3	-4.13E-09	3.86E-09	0.85792803	0.999871426
Primary_T_helper_cells_from_peripheral_blood__H3K4me3	-2.60E-08	2.41E-08	0.860031852	0.999871426
Primary_mononuclear_cells_from_peripheral_blood__H3K4me3	-3.36E-08	3.09E-08	0.861632195	0.999871426
Spleen_ENTEX__H3K4me1	-1.48E-08	1.34E-08	0.863858137	0.999871426
Primary_T_killer_naive_cells_from_peripheral_blood__H3K4me3	-2.95E-08	2.67E-08	0.865910558	0.999871426
Stomach_ENTEX__H3K36me3	-6.49E-09	5.85E-09	0.866424145	0.999871426
Prostate_ENTEX__H3K36me3	-3.80E-08	3.41E-08	0.867129098	0.999871426
NHLF_Lung_Fibroblast_Primary_Cells__H3K4me3	-3.90E-08	3.47E-08	0.869035627	0.999871426
Fetal_Stomach__H3K27ac	-7.84E-09	6.84E-09	0.874286533	0.999871426
NHLF_Lung_Fibroblast_Primary_Cells__H3K27ac	-1.19E-08	1.03E-08	0.876162664	0.999871426
Sigmoid_Colon__H3K27ac	-1.20E-08	1.04E-08	0.876251197	0.999871426
Sigmoid_Colon__H3K36me3	-1.31E-08	1.12E-08	0.877670525	0.999871426
Primary_neutrophils_from_peripheral_blood__H3K4me3	-1.15E-08	9.87E-09	0.878039829	0.999871426
Primary_hematopoietic_stem_cells_G-CSF-mobilized_Female__H3K4me3	-2.48E-08	2.13E-08	0.878163918	0.999871426
Primary_Natural_Killer_cells_from_peripheral_blood__H3K27ac	-9.31E-09	7.97E-09	0.878644314	0.999871426
Fetal_Intestine_Large__H3K4me3	-3.12E-08	2.63E-08	0.882544629	0.999871426
Adipose_Nuclei__H3K9ac	-1.62E-08	1.36E-08	0.883293083	0.999871426
Mammary_ENTEX__H3K4me1	-1.64E-08	1.37E-08	0.884554445	0.999871426
Primary_T_helper_memory_cells_from_peripheral_blood_2__H3K27ac	-1.34E-08	1.07E-08	0.893439375	0.999871426

Primary_hematopoietic_stem_cells__H3K4me3	-3.70E-08	2.93E-08	0.896968968	0.999871426
Primary_T_cells_from_peripheral_blood__H3K27ac	-1.05E-08	8.27E-09	0.897891295	0.999871426
Pancreas_ENTEX__H3K27ac	-6.13E-09	4.81E-09	0.898818435	0.999871426
Psoas_Muscle__H3K4me1	-1.40E-08	1.09E-08	0.9009511	0.999871426
NHDF-Ad_Adult_Dermal_Fibroblast_Primary_Cells__H3K4me3	-4.37E-08	3.38E-08	0.901447159	0.999871426
Testis_ENTEX__H3K36me3	-3.35E-08	2.59E-08	0.902104085	0.999871426
HMEC_Mammary_Epithelial_Primary_Cells__DNase	-1.56E-08	1.20E-08	0.903711465	0.999871426
Esoph-Mucosa_ENTEX__H3K27ac	-9.67E-09	7.28E-09	0.908072771	0.999871426
Osteoblast_Primary_Cells__H3K27ac	-8.17E-09	6.02E-09	0.912639945	0.999871426
Primary_mononuclear_cells_from_peripheral_blood__H3K9ac	-2.75E-08	2.02E-08	0.914015539	0.999871426
Heart-Atrial_ENTEX__H3K4me1	-3.34E-08	2.45E-08	0.914039599	0.999871426
Small_Intestine__DNase	-1.61E-08	1.18E-08	0.914689352	0.999871426
Placenta__H3K4me3	-3.83E-08	2.77E-08	0.916344665	0.999871426
Breast_variant_Human_Mammary_Epithelial_Cells_(vHMEC)__H3K36me3	-6.47E-09	4.60E-09	0.920182953	0.999871426
Spleen_ENTEX__H3K4me3	-1.14E-08	8.08E-09	0.921456402	0.999871426
Breast_Myoepithelial_Primary_Cells__H3K4me3	-2.45E-08	1.70E-08	0.924890428	0.999871426
Primary_T_helper_17_cells_PMA-I_stimulated__H3K4me3	-2.37E-08	1.64E-08	0.926392321	0.999871426
Primary_mononuclear_cells_from_peripheral_blood__H3K27ac	-3.59E-08	2.46E-08	0.928059962	0.999871426
Sigmoid_Colon__H3K4me3	-4.08E-08	2.74E-08	0.932073896	0.999871426
Fetal_Muscle_Leg__DNase	-1.71E-08	1.14E-08	0.933517148	0.999871426
Fetal_Lung__DNase	-1.66E-08	1.09E-08	0.935936492	0.999871426
Breast_Myoepithelial_Primary_Cells__H3K36me3	-7.90E-09	5.16E-09	0.937090354	0.999871426
Fetal_Kidney__H3K4me1	-2.71E-08	1.76E-08	0.937961944	0.999871426
Foreskin_Melanocyte_Primary_Cells_skin01__DNase	-1.80E-08	1.17E-08	0.93857295	0.999871426
Fetal_Lung__H3K4me1	-9.50E-09	6.13E-09	0.939330356	0.999871426
Rectal_Smooth_Muscle__H3K36me3	-2.32E-08	1.49E-08	0.939845681	0.999871426
Aorta__H3K4me1	-2.15E-08	1.38E-08	0.940132403	0.999871426
Uterus_ENTEX__H3K4me3	-4.35E-08	2.78E-08	0.941033528	0.999871426
Placenta_Amnion__H3K4me3	-5.09E-08	3.24E-08	0.941856163	0.999871426

Mammary_ENTEX__H3K27ac	-8.73E-09	5.49E-09	0.944176419	0.999871426
Uterus_ENTEX__H3K27ac	-7.81E-09	4.90E-09	0.944565821	0.999871426
liver_ENTEX__H3K36me3	-1.00E-08	6.30E-09	0.944638643	0.999871426
SI-Term-Ileum_ENTEX__H3K4me3	-2.62E-08	1.64E-08	0.944703209	0.999871426
Adipose_Nuclei__H3K36me3	-1.12E-08	6.95E-09	0.946298784	0.999871426
Vagina_ENTEX__H3K36me3	-3.14E-08	1.94E-08	0.947148742	0.999871426
Fetal_Heart__H3K36me3	-3.05E-08	1.89E-08	0.947183005	0.999871426
Liver__H3K4me1	-7.55E-09	4.65E-09	0.947692325	0.999871426
Primary_neutrophils_from_peripheral_blood__H3K4me1	-6.64E-09	4.07E-09	0.94843689	0.999871426
Ovary_ENTEX__H3K4me3	-4.97E-08	3.01E-08	0.950533799	0.999871426
Primary_monocytes_from_peripheral_blood__H3K27ac	-1.37E-08	8.31E-09	0.95055621	0.999871426
Fetal_Heart__H3K9ac	-1.29E-08	7.76E-09	0.951182352	0.999871426
Foreskin_Keratinocyte_Primary_Cells_skin03__H3K4me1	-9.29E-09	5.56E-09	0.952608141	0.999871426
Primary_B_cells_from_cord_blood__H3K4me3	-4.88E-08	2.89E-08	0.954232276	0.999871426
Gastric__H3K27ac	-2.14E-08	1.25E-08	0.956221101	0.999871426
HMEC_Mammary_Epithelial_Primary_Cells__H3K4me1	-1.10E-08	6.35E-09	0.95862323	0.999871426
Breast_Myoepithelial_Primary_Cells__H3K4me1	-9.25E-09	5.28E-09	0.960245715	0.999871426
Liver__H3K36me3	-7.78E-09	4.44E-09	0.960334834	0.999871426
Esophagus__H3K27ac	-1.41E-08	8.02E-09	0.960733472	0.999871426
NHDF-Ad_Adult_Dermal_Fibroblast_Primary_Cells__H3K36me3	-2.83E-08	1.61E-08	0.960883776	0.999871426
Placenta_Amnion__H3K4me1	-1.11E-08	6.27E-09	0.961281239	0.999871426
Primary_hematopoietic_stem_cells_G-CSF-mobilized_Female__H3K36me3	-1.07E-08	6.03E-09	0.96132888	0.999871426
Pancreas_ENTEX__H3K4me1	-6.85E-09	3.79E-09	0.964565986	0.999871426
Adrenal_gland_ENTEX__H3K36me3	-1.33E-08	7.28E-09	0.965710229	0.999871426
Breast_variant_Human_Mammary_Epithelial_Cells_(vHMEC)__H3K4me3	-4.73E-08	2.58E-08	0.966363718	0.999871426
Osteoblast_Primary_Cells__H3K4me1	-1.04E-08	5.67E-09	0.966583837	0.999871426
Duodenum_Mucosa__H3K9ac	-2.74E-08	1.47E-08	0.968588572	0.999871426
NHDF-Ad_Adult_Dermal_Fibroblast_Primary_Cells__H3K9ac	-3.45E-08	1.84E-08	0.969487874	0.999871426
Foreskin_Keratinocyte_Primary_Cells_skin02__H3K4me1	-1.47E-08	7.85E-09	0.969589978	0.999871426

Rectal_Mucosa_Donor_31__H3K4me3	-3.45E-08	1.83E-08	0.970130645	0.999871426
Primary_hematopoietic_stem_cells_G-CSF-mobilized_Male__H3K36me3	-9.92E-09	5.25E-09	0.97051843	0.999871426
Primary_monocytes_from_peripheral_blood__H3K36me3	-8.36E-09	4.42E-09	0.970522068	0.999871426
Primary_mononuclear_cells_from_peripheral_blood__H3K4me1	-3.76E-08	1.97E-08	0.971918405	0.999871426
Primary_T_helper_memory_cells_from_peripheral_blood_1__H3K4me3	-3.93E-08	2.05E-08	0.972152695	0.999871426
Fetal_Lung__H3K4me3	-3.67E-08	1.90E-08	0.973096004	0.999871426
Colonic_Mucosa__H3K27ac	-2.07E-08	1.06E-08	0.974116198	0.999871426
Liver__H3K9ac	-1.90E-08	9.79E-09	0.974148164	0.999871426
Primary_T_killer_memory_cells_from_peripheral_blood__H3K4me3	-4.49E-08	2.31E-08	0.974167557	0.999871426
Foreskin_Keratinocyte_Primary_Cells_skin02__DNase	-1.92E-08	9.84E-09	0.974182334	0.999871426
NHEK-Epidermal_Keratinocyte_Primary_Cells__DNase	-1.88E-08	9.61E-09	0.974580367	0.999871426
Pancreas_ENTEX__H3K36me3	-1.14E-08	5.80E-09	0.974908144	0.999871426
Ovary_ENTEX__H3K36me3	-4.67E-08	2.38E-08	0.974937529	0.999871426
Primary_hematopoietic_stem_cells__H3K36me3	-2.55E-08	1.29E-08	0.975624549	0.999871426
NHDF-Ad_Adult_Dermal_Fibroblast_Primary_Cells__DNase	-2.65E-08	1.34E-08	0.975711501	0.999871426
Breast_variant_Human_Mammary_Epithelial_Cells_(vHMEC)__DNase	-2.44E-08	1.23E-08	0.976635925	0.999871426
Primary_T_cells_from_peripheral_blood__H3K4me3	-4.33E-08	2.17E-08	0.977124057	0.999871426
Primary_T_cells_from_cord_blood__H3K4me3	-5.36E-08	2.64E-08	0.978886041	0.999871426
NHEK-Epidermal_Keratinocyte_Primary_Cells__H3K4me1	-1.08E-08	5.31E-09	0.978947879	0.999871426
Fetal_Lung__H3K36me3	-1.22E-08	5.98E-09	0.979050715	0.999871426
Ovary__H3K4me1	-1.77E-08	8.67E-09	0.979280048	0.999871426
Sigmoid_Colon__H3K4me1	-3.19E-08	1.55E-08	0.980137262	0.999871426
Primary_T_helper_naive_cells_from_peripheral_blood_1__H3K4me3	-4.69E-08	2.27E-08	0.980666709	0.999871426
Primary_Natural_Killer_cells_from_peripheral_blood__H3K4me3	-4.70E-08	2.27E-08	0.980792055	0.999871426
Esoph-Mucosa_ENTEX__H3K4me1	-1.32E-08	6.19E-09	0.983374647	0.999871426
Rectal_Mucosa_Donor_29__H3K27ac	-1.79E-08	8.36E-09	0.984036233	0.999871426
Rectal_Mucosa_Donor_31__H3K27ac	-1.80E-08	8.29E-09	0.985040898	0.999871426
Placenta__DNase	-2.39E-08	1.10E-08	0.985129743	0.999871426
Adipose_Nuclei__H3K27ac	-1.00E-08	4.45E-09	0.987803373	0.999871426

Duodenum_Smooth_Muscle__H3K4me3	-2.52E-08	1.12E-08	0.987972592	0.999871426
Adipose_Nuclei__H3K4me3	-3.28E-08	1.45E-08	0.9880036	0.999871426
NHDF-Ad_Adult_Dermal_Fibroblast_Primary_Cells__H3K4me1	-1.74E-08	7.57E-09	0.989214244	0.999871426
Breast_variant_Human_Mammary_Epithelial_Cells_(vHMEC)__H3K4me1	-1.43E-08	6.19E-09	0.989409335	0.999871426
Fetal_Intestine_Small__DNase	-3.78E-08	1.64E-08	0.989412246	0.999871426
Gastric__DNase	-4.25E-08	1.84E-08	0.989449557	0.999871426
Primary_B_cells_from_cord_blood__H3K36me3	-4.34E-08	1.88E-08	0.98960482	0.999871426
Colon-Sigm_ENTEX__H3K4me1	-6.43E-08	2.77E-08	0.989833845	0.999871426
Fetal_Kidney__H3K36me3	-4.11E-08	1.77E-08	0.989883316	0.999871426
Fetal_Muscle_Trunk__H3K27ac	-1.91E-08	8.23E-09	0.989907702	0.999871426
NHDF-Ad_Adult_Dermal_Fibroblast_Primary_Cells__H3K27ac	-2.60E-08	1.11E-08	0.990618926	0.999871426
Fetal_Brain_Male__H3K36me3	-5.74E-08	2.44E-08	0.990737851	0.999871426
Duodenum_Mucosa__H3K36me3	-1.81E-08	7.61E-09	0.991252309	0.999871426
Fetal_Intestine_Small__H3K27ac	-1.42E-08	5.92E-09	0.991837238	0.999871426
Primary_monocytes_from_peripheral_blood__H3K4me1	-1.12E-08	4.64E-09	0.992101199	0.999871426
HMEC_Mammary_Epithelial_Primary_Cells__H3K4me3	-5.80E-08	2.40E-08	0.992205579	0.999871426
liver_ENTEX__H3K4me1	-4.87E-08	2.00E-08	0.992480702	0.999871426
NHEK-Epidermal_Keratinocyte_Primary_Cells__H3K27ac	-1.64E-08	6.74E-09	0.992578252	0.999871426
Nerve-Tibial_ENTEX__H3K36me3	-1.57E-08	6.43E-09	0.992712045	0.999871426
Psoas_Muscle__H3K36me3	-3.73E-08	1.48E-08	0.994210232	0.999871426
Primary_T_killer_memory_cells_from_peripheral_blood__H3K27ac	-3.16E-08	1.25E-08	0.994439364	0.999871426
Fetal_Heart__H3K4me3	-3.05E-08	1.19E-08	0.994687862	0.999871426
Rectal_Mucosa_Donor_31__H3K9ac	-2.91E-08	1.13E-08	0.995052038	0.999871426
Duodenum_Mucosa__H3K4me3	-3.88E-08	1.50E-08	0.995135051	0.999871426
Primary_T_helper_cells_PMA-I_stimulated__H3K4me3	-3.29E-08	1.27E-08	0.995234313	0.999871426
NHEK-Epidermal_Keratinocyte_Primary_Cells__H3K9ac	-2.90E-08	1.11E-08	0.995551997	0.999871426
SI-Term-Ileum_ENTEX__H3K27ac	-1.44E-08	5.45E-09	0.995839812	0.999871426
Stomach_Mucosa__H3K4me3	-6.63E-08	2.49E-08	0.996160352	0.999871426
Primary_T_helper_memory_cells_from_peripheral_blood_2__H3K4me3	-5.98E-08	2.24E-08	0.996221699	0.999871426

Fetal_Intestine_Large__H3K36me3	-1.59E-08	5.93E-09	0.996339426	0.999871426
Fetal_Muscle_Leg__H3K27ac	-1.89E-08	7.03E-09	0.996384173	0.999871426
Fetal_Intestine_Large__H3K27ac	-1.58E-08	5.81E-09	0.996731289	0.999871426
SI-Term-Ileum_ENTEX__H3K4me1	-2.19E-08	7.91E-09	0.997161257	0.999871426
HMEC_Mammary_Epithelial_Primary_Cells__H3K27ac	-2.01E-08	7.16E-09	0.997479015	0.999871426
Duodenum_Mucosa__H3K4me1	-1.98E-08	7.03E-09	0.997605273	0.999871426
Duodenum_Smooth_Muscle__H3K36me3	-2.57E-08	8.78E-09	0.998259808	0.999871426
Prostate_ENTEX__H3K27ac	-1.66E-08	5.62E-09	0.998404439	0.999871426
Stomach_Mucosa__H3K9ac	-3.88E-08	1.29E-08	0.99873552	0.999871426
Stomach_Mucosa__H3K4me1	-1.81E-08	5.89E-09	0.998926555	0.999871426
Liver__H3K4me3	-3.35E-08	1.08E-08	0.999057213	0.999871426
Small_Intestine__H3K4me1	-3.75E-08	1.19E-08	0.999146361	0.999871426
Fetal_Intestine_Large__DNase	-3.91E-08	1.24E-08	0.999160133	0.999871426
Rectal_Mucosa_Donor_29__H3K4me1	-3.13E-08	9.91E-09	0.999207616	0.999871426
Fetal_Kidney__H3K9ac	-9.61E-08	3.02E-08	0.999260357	0.999871426
HMEC_Mammary_Epithelial_Primary_Cells__H3K9ac	-3.97E-08	1.25E-08	0.999281413	0.999871426
Rectal_Mucosa_Donor_31__H3K4me1	-2.29E-08	7.18E-09	0.999282881	0.999871426
Fetal_Intestine_Small__H3K4me1	-1.67E-08	5.24E-09	0.9992857	0.999871426
Stomach_ENTEX__H3K27ac	-1.85E-08	5.59E-09	0.999527261	0.999871426
Foreskin_Keratinocyte_Primary_Cells_skin03__H3K27ac	-2.30E-08	6.91E-09	0.999569285	0.999871426
SI-Term-Ileum_ENTEX__H3K36me3	-5.18E-08	1.55E-08	0.999571994	0.999871426
Fetal_Kidney__H3K4me3	-9.79E-08	2.92E-08	0.999593929	0.999871426
Rectal_Mucosa_Donor_31__H3K36me3	-2.64E-08	7.63E-09	0.999733338	0.999871426
Rectal_Mucosa_Donor_29__H3K4me3	-5.56E-08	1.57E-08	0.999798328	0.999871426
Rectal_Mucosa_Donor_29__H3K9ac	-6.54E-08	1.84E-08	0.99981318	0.999871426
Fetal_Intestine_Large__H3K4me1	-1.94E-08	5.33E-09	0.99986502	0.999871426
Rectal_Mucosa_Donor_29__H3K36me3	-3.23E-08	8.82E-09	0.999871426	0.999871426

Abbreviation: FDR, false discovery rate

Supplementary Table 8. Genetic correlation between irritability and other traits (non-neuroimaging traits)

Trait	Cohort	Category	rg	se	z-score	P	P_FDR
Major depressive disorder	PGC	Mental health	0.56	0.0221	25.3641	6.29E-142	5.72E-140
Ever highly irritable/argumentative for 2 days	UK Biobank (Ben Neale Group)	Mental health	0.7555	0.0408	-18.5154	1.55E-76	7.05E-75
Life satisfaction	23andMe	Mental health	-0.5589	0.034	16.4224	1.32E-60	4.00E-59
Depression	UK Biobank (Ben Neale Group)	Mental health	0.6271	0.0486	-12.9119	3.86E-38	8.78E-37
Ever depressed for a whole week	UK Biobank (Ben Neale Group)	Mental health	0.4704	0.038	-12.3831	3.23E-35	5.88E-34
Family relationship satisfaction	UK Biobank (Ben Neale Group)	Mental health	-0.4724	0.0392	-12.0528	1.88E-33	2.85E-32
Ever unenthusiastic/disinterested for a whole week	UK Biobank (Ben Neale Group)	Mental health	0.5327	0.0458	-11.623	3.15E-31	4.10E-30
Never smoking	UK Biobank (Ben Neale Group)	Life style	-0.3038	0.0276	11.0172	3.16E-28	3.59E-27
Friendships satisfaction	UK Biobank (Ben Neale Group)	Mental health	-0.3737	0.0366	-10.1964	2.06E-24	2.08E-23
Health satisfaction	UK Biobank (Ben Neale Group)	Mental health	-0.3984	0.0409	-9.7385	2.07E-22	1.88E-21
Financial situation satisfaction	UK Biobank (Ben Neale Group)	Mental health	-0.3582	0.042	-8.5329	1.43E-17	1.18E-16
Work/job satisfaction	UK Biobank (Ben Neale Group)	Mental health	-0.4112	0.0519	-7.9251	2.28E-15	1.73E-14
Leisure/social activities: Sports club or gym	UK Biobank (Ben Neale Group)	Life style	-0.2491	0.0334	7.4525	9.16E-14	6.41E-13
Bipolar disorder II	PGC	Mental health	0.38	0.0515	7.3733	1.66E-13	1.08E-12
Attention-deficit/hyperactivity disorder	PGC	Mental health	0.2946	0.0401	7.3397	2.14E-13	1.30E-12
Functional digestive disorders	UK Biobank (SAIGE)	Physical health	0.3693	0.0508	-7.2628	3.79E-13	2.16E-12
Education years	PMID 27225129	Cognitive function	-0.1545	0.0237	6.5063	7.70E-11	4.12E-10
Esophagitis, GERD and related diseases	UK Biobank (SAIGE)	Physical health	0.2452	0.0387	-6.3314	2.43E-10	1.23E-09
Psychiatric cross disorder	PGC	Mental health	0.2434	0.0395	-6.1591	7.31E-10	3.50E-09
Time spent watching television	UK Biobank (Ben Neale Group)	Life style	0.1538	0.0251	-6.1225	9.21E-10	4.19E-09
Constipation	UK Biobank (SAIGE)	Physical health	0.4166	0.0724	-5.7521	8.81E-09	3.82E-08
Ever self-harmed	UK Biobank (Ben Neale Group)	Mental health	0.3687	0.0645	-5.7169	1.08E-08	4.47E-08
Sleep duration	UK Biobank (Ben Neale Group)	Life style	-0.1697	0.0298	5.6898	1.27E-08	5.02E-08
Gastritis and duodenitis	UK Biobank (SAIGE)	Physical health	0.283	0.05	-5.6576	1.54E-08	5.84E-08
Ever manic/hyper for 2 days	UK Biobank (Ben Neale Group)	Mental health	0.4511	0.0831	-5.4292	5.66E-08	2.06E-07

Anxiety disorder	UK Biobank (SAIGE)	Mental health	0.3238	0.06	-5.3939	6.90E-08	2.42E-07
Ever panic attacks	UK Biobank (Ben Neale Group)	Mental health	0.3645	0.0684	-5.3327	9.68E-08	3.26E-07
Hyperlipidemia	UK Biobank (SAIGE)	Physical health	0.1776	0.0338	-5.2571	1.46E-07	4.75E-07
Schizophrenia	PGC	Mental health	0.132	0.0261	5.0626	4.14E-07	1.30E-06
Angina pectoris	UK Biobank (SAIGE)	Physical health	0.1816	0.0368	-4.932	8.14E-07	2.47E-06
Ischemic heart disease	UK Biobank (SAIGE)	Physical health	0.1619	0.0341	-4.7518	2.02E-06	5.93E-06
Autism spectrum disorder	PGC	Mental health	0.222	0.0491	4.519	6.21E-06	1.77E-05
Bipolar disorder I	PGC	Mental health	0.1462	0.033	4.4226	9.75E-06	2.69E-05
Hypothyroidism	UK Biobank (SAIGE)	Physical health	0.1669	0.0405	-4.1236	3.73E-05	9.98E-05
Obsessive-compulsive disorder	PGC	Mental health	0.238	0.0593	4.016	5.92E-05	0.000153436
Asthma	UK Biobank (SAIGE)	Physical health	0.1621	0.0404	-4.01	6.07E-05	0.000153436
Social anxiety disorder	UK Biobank (Ben Neale Group)	Mental health	0.3397	0.0893	-3.8043	1.00E-04	0.000245946
Hypertension	UK Biobank (SAIGE)	Physical health	0.1374	0.0369	-3.7202	2.00E-04	0.000466667
Coronary atherosclerosis	UK Biobank (SAIGE)	Physical health	0.1249	0.0339	-3.6843	2.00E-04	0.000466667
White blood cell (leukocyte) count	UK Biobank (Ben Neale Group)	Laboratory and Physical findings	0.089	0.0243	-3.6593	3.00E-04	0.00065
Body fat percentage	UK Biobank (Ben Neale Group)	Laboratory and Physical findings	0.0791	0.0221	-3.5816	3.00E-04	0.00065
Type 2 diabetes	UK Biobank (SAIGE)	Physical health	0.1392	0.0382	-3.6415	3.00E-04	0.00065
Number of depression episodes	UK Biobank (Ben Neale Group)	Mental health	0.289	0.0838	-3.4478	6.00E-04	0.001269767
Cerebrovascular disease	UK Biobank (SAIGE)	Physical health	0.3171	0.0943	-3.3623	8.00E-04	0.001617778
Myocardial infarction	UK Biobank (SAIGE)	Physical health	0.132	0.0392	-3.3669	8.00E-04	0.001617778
Ever taken cannabis	UK Biobank (Ben Neale Group)	Life style	0.1251	0.0386	-3.2439	0.0012	0.002373913
Anorexia nervosa	PGC	Mental health	0.1337	0.0426	3.1388	0.0017	0.003291489
Cardiac dysrhythmias	UK Biobank (SAIGE)	Physical health	0.1316	0.0432	-3.0449	0.0023	0.004360417
Urinary incontinence	UK Biobank (SAIGE)	Physical health	0.1581	0.0527	-2.9984	0.0027	0.005014286
Body mass index	PMID 30239722	Laboratory and Physical findings	0.0622	0.0208	-2.9906	0.0028	0.005096
Urate	UK Biobank (Ben Neale Group)	Laboratory and Physical findings	0.0651	0.0219	-2.9797	0.0029	0.00517451
Tourette's Syndrome	PGC	Mental health	0.1375	0.0474	2.9016	0.0037	0.006475
Chronotype (eveningness, continuous)	International Sleep Genetic Epidemiology Consortium (ISGEC)	Life style	0.106	0.0372	-2.8484	0.0044	0.007554717
Urinary tract infection	UK Biobank (SAIGE)	Physical health	0.2026	0.076	-2.6645	0.0077	0.012975926

Testosterone	UK Biobank (Ben Neale Group)	Laboratory and Physical findings	-0.0702	0.0272	2.5793	0.0099	0.0160875
Leisure/social activities: religious group	UK Biobank (Ben Neale Group)	Life style	-0.1056	0.0409	2.5801	0.0099	0.0160875
Osteoporosis	UK Biobank (SAIGE)	Physical health	0.1266	0.0512	-2.4735	0.0134	0.021392982
C-reactive protein	UK Biobank (Ben Neale Group)	Laboratory and Physical findings	0.0711	0.0325	-2.1894	0.0286	0.044872414
Vitamin D	UK Biobank (Ben Neale Group)	Laboratory and Physical findings	-0.0534	0.0247	2.1633	0.0305	0.047042373
Atrial fibrillation and flutter	UK Biobank (SAIGE)	Physical health	0.0776	0.0376	-2.0621	0.0392	0.059453333
Chronotype (definite morningness, categorical)	International Sleep Genetic Epidemiology Consortium (ISGEC)	Life style	-0.088	0.043	2.0444	0.0409	0.061014754
Total serum protein	UK Biobank (Ben Neale Group)	Laboratory and Physical findings	-0.0609	0.0304	2.0019	0.0453	0.06648871
HDL cholesterol	UK Biobank (Ben Neale Group)	Laboratory and Physical findings	-0.0451	0.023	1.9638	0.0495	0.0715
Ever attempted suicide	UK Biobank (Ben Neale Group)	Mental health	0.438	0.2261	-1.937	0.0527	0.074932813
Obesity	UK Biobank (SAIGE)	Laboratory and Physical findings	0.0874	0.0461	-1.8959	0.058	0.0812
Hearing loss	UK Biobank (SAIGE)	Physical health	0.2739	0.1588	-1.7244	0.0846	0.116645455
Sleep disorders	UK Biobank (SAIGE)	Physical health	0.1142	0.0677	-1.688	0.0914	0.124140299
Renal failure	UK Biobank (SAIGE)	Physical health	0.1465	0.0906	-1.6175	0.1058	0.141585294
Rheumatoid arthritis and other inflammatory polyarthropathies	UK Biobank (SAIGE)	Physical health	0.1208	0.0753	-1.604	0.1087	0.143357971
Neurological disorders	UK Biobank (SAIGE)	Mental health	0.1985	0.1261	-1.574	0.1155	0.15015
Osteoarthrosis	UK Biobank (SAIGE)	Physical health	0.0889	0.057	-1.5599	0.1188	0.152264789
Leisure/social activities: adult education class	UK Biobank (Ben Neale Group)	Life style	-0.0767	0.0506	1.5168	0.1293	0.163420833
Tryglyceride	PMID 24097068	Laboratory and Physical findings	0.041	0.0282	-1.457	0.1451	0.180878082
Oestradiol	UK Biobank (Ben Neale Group)	Laboratory and Physical findings	-0.1715	0.1257	1.3641	0.1725	0.212128378
Total cholesterol	PMID 24097068	Laboratory and Physical findings	0.0411	0.031	-1.3254	0.1851	0.223009091
Sleep apnea	UK Biobank (SAIGE)	Physical health	0.091	0.069	-1.3192	0.1871	0.223009091
General cognitive function	Centre for Cognitive Ageing and Cognitive Epidemiology (CCACE)	Cognitive function	-0.0316	0.0241	1.3145	0.1887	0.223009091
Iron deficiency anemias	UK Biobank (SAIGE)	Physical health	0.0859	0.0847	-1.0141	0.3105	0.36225
Duration of walks	UK Biobank (Ben Neale Group)	Life style	0.0342	0.0341	-1.0023	0.3162	0.36423038
Miscarriage	UK Biobank (SAIGE)	Physical health	-0.0864	0.1194	0.7237	0.4693	0.53382875
Cognitive performance	Centre for Cognitive Ageing and Cognitive Epidemiology (CCACE)	Cognitive function	-0.0155	0.0234	0.6615	0.5083	0.571053086
Intelligence	PMID 29942086	Cognitive function	-0.0175	0.0278	0.6298	0.5288	0.586839024
Breast cancer	UK Biobank (SAIGE)	Physical health	0.0269	0.0464	-0.5793	0.5624	0.611

Coffee intake	UK Biobank (Ben Neale Group)	Life style	-0.0189	0.0327	0.5769	0.564	0.611
Haemoglobin concentration	UK Biobank (Ben Neale Group)	Laboratory and Physical findings	-0.0105	0.0239	0.4412	0.6591	0.705624706
Verbal numerical reasoning	Centre for Cognitive Ageing and Cognitive Epidemiology (CCACE)	Cognitive function	-0.0103	0.0246	0.4206	0.6741	0.71329186
Never alcohol drinking	UK Biobank (Ben Neale Group)	Life style	-0.0183	0.0518	0.3528	0.7243	0.757601149
Low-density lipoprotein cholesterol	PMID 24097068	Laboratory and Physical findings	0.0094	0.0298	-0.3137	0.7538	0.779497727
Fasting glucose	UK Biobank (Ben Neale Group)	Laboratory and Physical findings	0.0078	0.0314	-0.2475	0.8046	0.822680899
Alzheimer's disease	PMID 30820047	Mental health	-0.0138	0.067	0.2058	0.8369	0.846198889
Leisure/social activities: pub or social club	UK Biobank (Ben Neale Group)	Life style	-5.00E-04	0.0375	0.0126	0.99	0.99

Abbreviations. rg, genetic correlation; GERD, gastroesophageal reflux disease; FDR, false discovery rate; s.e., standard error.

Supplementary Table 9. Genetic correlation between irritability and neuroimaging traits

Trait	cohort	rg	se	z	P	P_FDR
FXST.FA	DTI110	-0.1693	0.0536	-3.1589	0.0016	0.0352
right.precuneus	ROI101	0.1622	0.0541	3.0004	0.0027	0.15655
left.postcentral	ROI101	0.1855	0.0628	2.9545	0.0031	0.15655
EC.MO	DTI110	-0.0985	0.0481	-2.0502	0.0403	0.313866667
FXST.MO	DTI110	-0.1163	0.0556	-2.093	0.0363	0.313866667
SCC.MO	DTI110	0.0908	0.0448	2.0258	0.0428	0.313866667
FX.MO	DTI110	-0.1384	0.0757	-1.8275	0.0676	0.3718
FXST.AD	DTI110	-0.1313	0.0552	-2.3771	0.0174	0.3828
left.caudal.anterior.cingulate	ROI101	0.1281	0.0533	2.4039	0.0162	0.457025
right.caudal.anterior.cingulate	ROI101	0.2139	0.0905	2.363	0.0181	0.457025
UNC.AD	DTI110	-0.1535	0.0758	-2.0244	0.0429	0.4719
GCC.FA	DTI110	-0.0908	0.0468	-1.9417	0.0522	0.5742
right.lateral.ventricle	ROI101	0.1043	0.0476	2.19	0.0285	0.5757
BCC.MO	DTI110	-0.0764	0.0524	-1.4593	0.1445	0.6358
CGC.AD	DTI110	-0.0804	0.0534	-1.5056	0.1322	0.637755556
CGH.AD	DTI110	-0.0693	0.0617	-1.1243	0.2609	0.637755556
CST.AD	DTI110	-0.0826	0.0554	-1.4915	0.1358	0.637755556
EC.AD	DTI110	-0.0668	0.057	-1.1715	0.2414	0.637755556
FX.AD	DTI110	0.0566	0.048	1.1796	0.2381	0.637755556
PTR.AD	DTI110	-0.0764	0.0599	-1.2756	0.2021	0.637755556
SS.AD	DTI110	-0.0728	0.0614	-1.1861	0.2356	0.637755556
CST.RD	DTI110	-0.0948	0.0444	-2.136	0.0327	0.7194
total.brain.volume	ROI101	-0.0877	0.0441	-1.9894	0.0467	0.725095833
right.lingual	ROI101	0.1015	0.0529	1.9172	0.0552	0.725095833
right.fusiform	ROI101	0.0915	0.0499	1.833	0.0668	0.725095833

left.lateral.ventricle	ROI101	0.0901	0.0508	1.7727	0.0763	0.725095833
right.basal.forebrain	ROI101	0.1031	0.0617	1.6713	0.0947	0.725095833
left.basal.forebrain	ROI101	0.2075	0.1246	1.666	0.0957	0.725095833
left.superior.temporal	ROI101	0.0881	0.0549	1.605	0.1085	0.725095833
left.thalamus.proper	ROI101	-0.0747	0.0471	-1.5845	0.1131	0.725095833
right.pars.opercularis	ROI101	0.1252	0.0796	1.5735	0.1156	0.725095833
right.thalamus.proper	ROI101	-0.068	0.0447	-1.5208	0.1283	0.725095833
X3rd.ventricle	ROI101	0.0744	0.0495	1.5009	0.1334	0.725095833
left.rostral.anterior.cingulate	ROI101	0.1007	0.0674	1.4946	0.135	0.725095833
left.cuneus	ROI101	0.1069	0.0719	1.4865	0.1372	0.725095833
left.superior.frontal	ROI101	-0.0869	0.062	-1.4021	0.1609	0.725095833
cerebellar.vermal.lobules.I.V	ROI101	-0.0722	0.0519	-1.3916	0.1641	0.725095833
left.entorhinal	ROI101	0.0764	0.0552	1.3858	0.1658	0.725095833
right.accumbens.area	ROI101	-0.0633	0.0459	-1.3787	0.168	0.725095833
cerebellar.vermal.lobules.VIII.X	ROI101	-0.0638	0.0464	-1.3753	0.169	0.725095833
left.precuneus	ROI101	0.0786	0.0576	1.3649	0.1723	0.725095833
BCC.RD	DTI110	0.0749	0.0496	1.5111	0.1308	0.7276
CGC.RD	DTI110	0.0504	0.051	0.9884	0.3229	0.7276
FX.RD	DTI110	0.0692	0.0532	1.3024	0.1928	0.7276
FXST.RD	DTI110	0.0836	0.0578	1.4448	0.1485	0.7276
GCC.RD	DTI110	0.082	0.0485	1.6889	0.0912	0.7276
IFO.RD	DTI110	0.0716	0.0657	1.09	0.2757	0.7276
PTR.RD	DTI110	-0.0592	0.0512	-1.1566	0.2474	0.7276
RLIC.RD	DTI110	-0.0518	0.0479	-1.0803	0.28	0.7276
SCR.RD	DTI110	-0.0437	0.0482	-0.9081	0.3638	0.7276
SFO.RD	DTI110	0.0558	0.0587	0.9498	0.3422	0.7276
left.vessel	ROI101	0.0977	0.0739	1.3211	0.1865	0.75346
right.lateral.occipital	ROI101	-0.0697	0.0549	-1.2698	0.2042	0.781066667
cerebellar.vermal.lobules.VI.VII	ROI101	-0.0613	0.0488	-1.2569	0.2088	0.781066667

left.hippocampus	ROI101	0.0533	0.0435	1.2247	0.2207	0.785710345
left.middle.temporal	ROI101	-0.0794	0.0655	-1.2118	0.2256	0.785710345
Average.FA	DTI110	-0.0404	0.0459	-0.88	0.3788	0.788186667
BCC.FA	DTI110	-0.073	0.0523	-1.3958	0.1628	0.788186667
CGC.FA	DTI110	-0.0687	0.0482	-1.4254	0.154	0.788186667
CGH.FA	DTI110	-0.0443	0.0485	-0.913	0.3612	0.788186667
EC.FA	DTI110	-0.0378	0.0466	-0.8122	0.4167	0.788186667
FX.FA	DTI110	-0.0535	0.0593	-0.9024	0.3668	0.788186667
IFO.FA	DTI110	-0.0727	0.0573	-1.2695	0.2043	0.788186667
PLIC.FA	DTI110	0.0418	0.0477	0.8765	0.3808	0.788186667
PTR.FA	DTI110	0.0323	0.0507	0.6372	0.524	0.788186667
RLIC.FA	DTI110	0.0416	0.0438	0.9494	0.3424	0.788186667
SCR.FA	DTI110	0.0352	0.0503	0.699	0.4845	0.788186667
SFO.FA	DTI110	-0.0357	0.0578	-0.6167	0.5374	0.788186667
SLF.FA	DTI110	0.03	0.0455	0.6591	0.5098	0.788186667
CGC.MO	DTI110	-0.053	0.047	-1.1275	0.2595	0.79145
PLIC.MO	DTI110	0.054	0.0469	1.1521	0.2493	0.79145
RLIC.MO	DTI110	0.0507	0.0477	1.063	0.2878	0.79145
ACR.MO	DTI110	0.0148	0.0579	0.2564	0.7976	0.7976
ALIC.MO	DTI110	0.0238	0.0447	0.5316	0.595	0.7976
Average.MO	DTI110	-0.0209	0.0534	-0.3909	0.6959	0.7976
CGH.MO	DTI110	-0.0439	0.0589	-0.7459	0.4557	0.7976
CST.MO	DTI110	0.021	0.0653	0.3218	0.7476	0.7976
GCC.MO	DTI110	0.0259	0.0636	0.4078	0.6834	0.7976
IFO.MO	DTI110	-0.0501	0.0613	-0.8173	0.4138	0.7976
PCR.MO	DTI110	-0.0355	0.0518	-0.6859	0.4928	0.7976
PTR.MO	DTI110	0.0163	0.0568	0.2879	0.7735	0.7976
SCR.MO	DTI110	0.0503	0.0525	0.9568	0.3387	0.7976
SFO.MO	DTI110	0.031	0.052	0.5971	0.5504	0.7976

SLF.MO	DTI110	0.0467	0.0514	0.9095	0.3631	0.7976
SS.MO	DTI110	0.0389	0.0612	0.6366	0.5244	0.7976
UNC.MO	DTI110	-0.0348	0.0805	-0.4318	0.6659	0.7976
right.pars.orbitalis	ROI101	-0.092	0.0782	-1.1761	0.2396	0.799854839
right.rostral.anterior.cingulate	ROI101	0.0857	0.0738	1.1614	0.2455	0.799854839
CST.MD	DTI110	-0.0967	0.0465	-2.081	0.0374	0.8228
Average.AD	DTI110	-0.0347	0.0544	-0.6378	0.5236	0.83468
IFO.AD	DTI110	-0.0344	0.0592	-0.5804	0.5616	0.83468
PCR.AD	DTI110	-0.0361	0.0572	-0.6302	0.5286	0.83468
PLIC.AD	DTI110	0.0317	0.0556	0.5694	0.5691	0.83468
RLIC.AD	DTI110	-0.0306	0.0512	-0.5972	0.5504	0.83468
SCC.AD	DTI110	0.0379	0.0447	0.8473	0.3968	0.83468
right.middle.temporal	ROI101	-0.0651	0.0588	-1.1067	0.2684	0.836795745
left.paracentral	ROI101	0.09	0.0838	1.0738	0.2829	0.836795745
left.pars.triangularis	ROI101	-0.0736	0.0706	-1.042	0.2974	0.836795745
right.inferior.lateral.ventricle	ROI101	0.0749	0.0721	1.0399	0.2984	0.836795745
left.inferior.lateral.ventricle	ROI101	0.0589	0.0613	0.9607	0.3367	0.836795745
right.superior.parietal	ROI101	0.066	0.0693	0.9518	0.3412	0.836795745
left.fusiform	ROI101	0.0623	0.0665	0.9363	0.3491	0.836795745
right.postcentral	ROI101	0.0588	0.0628	0.9357	0.3494	0.836795745
Brain.stem	ROI101	-0.0391	0.0418	-0.9358	0.3494	0.836795745
right.cuneus	ROI101	0.0495	0.0543	0.9121	0.3617	0.836795745
right.entorhinal	ROI101	0.0501	0.0565	0.8856	0.3758	0.836795745
right.inferior.temporal	ROI101	-0.056	0.0633	-0.8837	0.3769	0.836795745
left.posterior.cingulate	ROI101	0.0488	0.0558	0.8741	0.3821	0.836795745
right.caudal.middle.frontal	ROI101	0.0541	0.0621	0.8713	0.3836	0.836795745
right.cerebellum.exterior	ROI101	-0.0381	0.044	-0.8669	0.386	0.836795745
optic.chiasm	ROI101	0.302	0.3508	0.8607	0.3894	0.836795745
right.hippocampus	ROI101	0.0417	0.0493	0.8455	0.3978	0.8370375

left.ventral.DC	ROI101	-0.0358	0.0448	-0.7995	0.424	0.84582549
right.paracentral	ROI101	0.0581	0.0728	0.7987	0.4245	0.84582549
left.cerebellum.exterior	ROI101	-0.0337	0.0424	-0.7941	0.4271	0.84582549
left.medial.orbitofrontal	ROI101	-0.042	0.0553	-0.7598	0.4474	0.855010909
right.pars.triangularis	ROI101	-0.0647	0.0859	-0.7531	0.4514	0.855010909
left.amygdala	ROI101	0.0412	0.0556	0.7417	0.4583	0.855010909
left.pallidum	ROI101	0.0365	0.05	0.7296	0.4656	0.855010909
right.precentral	ROI101	-0.0385	0.0554	-0.6947	0.4873	0.861296923
right.inferior.parietal	ROI101	0.0364	0.0537	0.6781	0.4977	0.861296923
left.insula	ROI101	-0.0307	0.0455	-0.6733	0.5008	0.861296923
right.parahippocampal	ROI101	0.0319	0.0486	0.6559	0.5119	0.861296923
right.caudate	ROI101	0.0384	0.0591	0.6502	0.5156	0.861296923
right.superior.frontal	ROI101	-0.0423	0.0672	-0.6301	0.5286	0.861296923
CSF	ROI101	-0.0314	0.0499	-0.6295	0.529	0.861296923
left.pars.orbitalis	ROI101	0.0417	0.0694	0.6016	0.5474	0.861296923
left.isthmus.cingulate	ROI101	-0.0346	0.0577	-0.5999	0.5486	0.861296923
left.cerebellum.white.matter	ROI101	-0.0253	0.0427	-0.5914	0.5543	0.861296923
right.posterior.cingulate	ROI101	0.0376	0.0656	0.5725	0.567	0.867681818
left.rostral.middle.frontal	ROI101	-0.0323	0.0585	-0.5521	0.5809	0.875685075
PLIC.RD	DTI110	-0.0346	0.0545	-0.6346	0.5257	0.889646154
SS.RD	DTI110	-0.0342	0.0505	-0.6769	0.4984	0.889646154
right.medial.orbitofrontal	ROI101	0.0274	0.0537	0.51	0.6101	0.891423377
left.putamen	ROI101	0.0205	0.0426	0.4822	0.6296	0.891423377
left.pars.opercularis	ROI101	-0.0508	0.1088	-0.4667	0.6407	0.891423377
right.insula	ROI101	-0.0201	0.0433	-0.4648	0.6421	0.891423377
left.parahippocampal	ROI101	-0.022	0.0481	-0.4579	0.647	0.891423377
left.caudate	ROI101	0.024	0.0526	0.4561	0.6483	0.891423377
gray.matter	ROI101	-0.0215	0.0488	-0.4396	0.6603	0.891423377
right.superior.temporal	ROI101	0.0234	0.0549	0.4263	0.6699	0.891423377

right.putamen	ROI101	0.0171	0.0405	0.423	0.6723	0.891423377
right.rostral.middle.frontal	ROI101	-0.0256	0.0621	-0.413	0.6796	0.891423377
right.cerebellum.white.matter	ROI101	-0.0164	0.0423	-0.3882	0.6979	0.896726582
right.isthmus.cingulate	ROI101	0.0221	0.0576	0.3834	0.7014	0.896726582
right.supramarginal	ROI101	-0.0205	0.0583	-0.3509	0.7256	0.899762195
right.lateral.orbitofrontal	ROI101	-0.0185	0.053	-0.348	0.7279	0.899762195
left.precentral	ROI101	-0.0208	0.0604	-0.3444	0.7305	0.899762195
left.caudal.middle.frontal	ROI101	-0.0174	0.0574	-0.303	0.7619	0.90287191
X4th.ventricle	ROI101	-0.0139	0.0462	-0.2998	0.7643	0.90287191
left.lateral.orbitofrontal	ROI101	0.0166	0.0564	0.2935	0.7692	0.90287191
left.inferior.parietal	ROI101	0.0152	0.0549	0.2771	0.7817	0.90287191
right.amygdala	ROI101	0.0148	0.0557	0.2649	0.7911	0.90287191
left.supramarginal	ROI101	-0.0151	0.0578	-0.2607	0.7943	0.90287191
right.vessel	ROI101	-0.0172	0.0664	-0.259	0.7956	0.90287191
BCC.MD	DTI110	0.0571	0.0469	1.2174	0.2235	0.904357143
CGH.MD	DTI110	-0.0373	0.0604	-0.6175	0.5369	0.904357143
EC.MD	DTI110	-0.0353	0.0615	-0.573	0.5666	0.904357143
FX.MD	DTI110	0.0684	0.0506	1.3516	0.1765	0.904357143
GCC.MD	DTI110	0.0543	0.0486	1.1183	0.2634	0.904357143
IFO.MD	DTI110	0.0591	0.0797	0.742	0.4581	0.904357143
PCR.MD	DTI110	-0.0283	0.0497	-0.5691	0.5693	0.904357143
PTR.MD	DTI110	-0.0848	0.0531	-1.5952	0.1107	0.904357143
RLIC.MD	DTI110	-0.0518	0.0535	-0.9676	0.3333	0.904357143
SCC.MD	DTI110	0.0267	0.0477	0.5599	0.5755	0.904357143
SFO.MD	DTI110	0.0364	0.0634	0.5737	0.5661	0.904357143
SS.MD	DTI110	-0.054	0.0538	-1.004	0.3154	0.904357143
UNC.MD	DTI110	-0.0926	0.0659	-1.4051	0.16	0.904357143
ACR.AD	DTI110	-0.0152	0.051	-0.2976	0.766	0.91949
BCC.AD	DTI110	0.011	0.0459	0.2399	0.8104	0.91949

GCC.AD	DTI110	-0.0105	0.0505	-0.2072	0.8359	0.91949
SCR.AD	DTI110	0.0167	0.0608	0.2755	0.783	0.91949
SLF.AD	DTI110	0.0136	0.0476	0.2856	0.7752	0.91949
right.ventral.DC	ROI101	0.0094	0.0442	0.2122	0.8319	0.922901075
left.transverse.temporal	ROI101	0.0123	0.0592	0.2075	0.8357	0.922901075
left.lateral.occipital	ROI101	-0.0124	0.0626	-0.1977	0.8433	0.922901075
right.pericalcarine	ROI101	0.0116	0.0613	0.1893	0.8498	0.922901075
left.lingual	ROI101	0.0109	0.0623	0.1743	0.8617	0.925869149
SCC.FA	DTI110	0.019	0.0455	0.4177	0.6762	0.929775
left.accumbens.area	ROI101	-0.0065	0.042	-0.1554	0.8765	0.931857895
PCR.FA	DTI110	0.0138	0.0459	0.3	0.7642	0.934022222
SS.FA	DTI110	0.0159	0.0485	0.3285	0.7425	0.934022222
left.inferior.temporal	ROI101	0.0078	0.058	0.1353	0.8923	0.938773958
ACR.MD	DTI110	-0.0103	0.0477	-0.2168	0.8284	0.9443
ALIC.MD	DTI110	0.0143	0.0702	0.2042	0.8382	0.9443
Average.MD	DTI110	-0.0087	0.0514	-0.169	0.8658	0.9443
CGC.MD	DTI110	-0.0045	0.0612	-0.0729	0.9419	0.9443
FXST.MD	DTI110	-0.0124	0.0628	-0.1974	0.8435	0.9443
PLIC.MD	DTI110	0.0048	0.0689	0.0699	0.9443	0.9443
SCR.MD	DTI110	-0.0119	0.0506	-0.2351	0.8141	0.9443
SLF.MD	DTI110	-0.0113	0.0467	-0.2421	0.8087	0.9443
white.matter	ROI101	-0.0049	0.0458	-0.108	0.914	0.949602
left.pericalcarine	ROI101	0.0045	0.0527	0.0851	0.9322	0.949602
left.superior.parietal	ROI101	0.006	0.072	0.0839	0.9331	0.949602
right.transverse.temporal	ROI101	0.0041	0.0542	0.075	0.9402	0.949602
UNC.FA	DTI110	-0.0128	0.0584	-0.2196	0.8262	0.956652632
ALIC.AD	DTI110	0.003	0.0601	0.0504	0.9598	0.9598
SFO.AD	DTI110	0.0045	0.0556	0.0815	0.935	0.9598
right.pallidum	ROI101	0.0024	0.0473	0.0503	0.9599	0.9599

UNC.RD	DTI110	-0.0308	0.0612	-0.5032	0.6148	0.966114286
PCR.RD	DTI110	-0.0188	0.0474	-0.3962	0.6919	0.979275
SLF.RD	DTI110	-0.0169	0.0459	-0.3689	0.7122	0.979275
ALIC.FA	DTI110	0.0064	0.0511	0.1253	0.9003	0.99033
ACR.FA	DTI110	-3.00E-04	0.044	-0.0069	0.9945	0.9945
CST.FA	DTI110	0.0011	0.0618	0.0182	0.9855	0.9945
ACR.RD	DTI110	-0.0046	0.0457	-0.1016	0.9191	0.9949
ALIC.RD	DTI110	-0.0035	0.0617	-0.0564	0.955	0.9949
Average.RD	DTI110	3.00E-04	0.0499	0.0064	0.9949	0.9949
CGH.RD	DTI110	0.0034	0.0521	0.0661	0.9473	0.9949
EC.RD	DTI110	-0.0033	0.0577	-0.0569	0.9546	0.9949
SCC.RD	DTI110	0.006	0.047	0.1271	0.8989	0.9949

Abbreviations. rg, genetic correlation; FDR, false discovery rate; s.e., standard error.

**ID**	**Full Name**	
ACR	Anterior corona radiata	
ALIC	Anterior limb of internal capsule	
Average	Average across all tracts	
BCC	Body of corpus callosum	
CGC	Cingulum (cingulate gyrus)	
CGH	Cingulum (hippocampus)	
CST	Corticospinal tract	
EC	External capsule	
FX	Fornix (column and body of fornix)	
FXST	Fornix (cres) / Stria terminalis (can not be resolved with current resolution)	
GCC	Genu of corpus callosum	
IFO	Inferior fronto-occipital fasciculus	
PCR	Posterior corona radiata	

PLIC	Posterior limb of internal capsule
PTR	Posterior thalamic radiation (include optic radiation)
RLIC	Retrolenticular part of internal capsule
SCC	Splenium of corpus callosum
SCR	Superior corona radiata
SFO	Superior fronto-occipital fasciculus (could be a part of anterior internal capsule)
SLF	Superior longitudinal fasciculus
SS	Sagittal stratum (include inferior longitidinal fasciculus and inferior fronto-occipital fasciculus)
UNC	Uncinate fasciculus
FA	fractional anisotropy
MD	mean diusivities
AD	axial diusivities
RD	radial diusivities
MO	mode of anisotropy

Supplementary Table 10. Summary of psychiatric disorder datasets

Group	Disorder	#Cases	#Controls	Total Samples	References
Mood and Psychotic Disorders	SCZ	40,675	64,643	105,318	Pardiñas et al., 2018
	BD I	25,060	449,978	475,038	Mullins et al., 2021
	BD II	6,781	364,075	370,856	Mullins et al., 2021
	MDD	170,756	329,443	500,199	Howard et al., 2019
Early-onset Neurodevelopmental Disorders	ASD	18,381	27,969	46,350	Grove et al., 2019
	ADHD	19,099	34,194	53,293	Demontis et al., 2019
	TS	4,819	9,488	14,307	Yu et al., 2019
Disorders with Compulsive behaviors	OCD	2,688	7,037	9,725	IOCDF-GC and OCGAS 2018
	AN	16,992	55,525	72,517	Watson et al., 2019

Abbreviations. SCZ, schizophrenia; BD, bipolar disorder; MDD, major depressive disorder; ASD, autism spectrum disorder; ADHD, attention deficit/hyperactivity disorder; TS, Tourette's Syndrome; OCD, obsessive-compulsive disorder; AN, anorexia nervosa.

Supplementary Table 11. Genetic correlation between irritability and psychiatric disorders (LDSC)

Trait1	Trait2	rg	se	z-score	P	P_FDR
BDI	BDII	0.8518	0.0535	15.9306	3.89E-57	3.06E-56
BDI	SCZ	0.6615	0.0186	35.6152	8.16E-278	1.50E-276
MDD	BDII	0.6496	0.0486	13.3644	9.77E-41	5.37E-40
Irritable	MDD	0.56	0.0221	25.3641	6.29E-142	8.65E-141
SCZ	BDII	0.5417	0.0456	11.881	1.49E-32	6.83E-32
ADHD	MDD	0.4549	0.0323	14.0974	3.94E-45	2.41E-44
AN	OCD	0.4396	0.0819	5.3673	7.99E-08	1.76E-07
OCD	BDII	0.4348	0.1077	4.0378	5.40E-05	8.25E-05
OCD	TS	0.4175	0.0929	4.4944	6.98E-06	1.16E-05
Irritable	BDII	0.38	0.0515	7.3733	1.66E-13	6.09E-13
ADHD	BDII	0.3776	0.0646	5.842	5.16E-09	1.42E-08
ASD	MDD	0.3526	0.035	10.0813	6.68E-24	2.83E-23
ADHD	ASD	0.3453	0.0512	6.7405	1.58E-11	4.83E-11
BDI	MDD	0.3405	0.0263	12.9375	2.77E-38	1.39E-37
OCD	SCZ	0.3318	0.0574	5.7831	7.33E-09	1.75E-08
ASD	BDII	0.3308	0.084	3.9389	8.19E-05	0.000118539
MDD	SCZ	0.3301	0.0222	14.8625	5.78E-50	3.97E-49
Irritable	ADHD	0.2946	0.0401	7.3397	2.14E-13	7.36E-13
BDI	OCD	0.2799	0.0685	4.0865	4.38E-05	6.88E-05
MDD	OCD	0.2591	0.0516	5.0192	5.19E-07	9.84E-07
AN	MDD	0.2568	0.0361	7.1038	1.21E-12	3.91E-12
AN	SCZ	0.2427	0.0303	8.0098	1.15E-15	4.52E-15
Irritable	OCD	0.238	0.0593	4.016	5.92E-05	8.80E-05
ASD	SCZ	0.2379	0.041	5.795	6.83E-09	1.71E-08
AN	BDII	0.2325	0.0639	3.64	3.00E-04	0.000402439

Irritable	ASD	0.222	0.0491	4.519	6.21E-06	1.07E-05
MDD	TS	0.217	0.0418	5.1868	2.14E-07	4.53E-07
ADHD	TS	0.2079	0.0545	3.8157	1.00E-04	0.000141026
ADHD	SCZ	0.1685	0.0331	5.0918	3.55E-07	7.23E-07
Irritable	BDI	0.1462	0.033	4.4226	9.75E-06	1.58E-05
Irritable	TS	0.1375	0.0474	2.9016	0.0037	0.004625
AN	BDI	0.1362	0.0369	3.6917	2.00E-04	0.000275
ASD	BDI	0.1338	0.0445	3.0041	0.0027	0.003453488
Irritable	AN	0.1337	0.0426	3.1388	0.0017	0.00222619
TS	BDII	0.1324	0.0875	1.5143	0.1299	0.137394231
Irritable	SCZ	0.132	0.0261	5.0626	4.14E-07	8.13E-07
ASD	TS	0.129	0.0609	2.1186	0.0341	0.039072917
ASD	OCD	0.1185	0.0827	1.4336	0.1517	0.157424528
AN	TS	0.1111	0.0671	1.656	0.0977	0.105362745
ADHD	BDI	0.1082	0.0384	2.8204	0.0048	0.005866667
AN	ASD	0.1032	0.0571	1.807	0.0708	0.07788
SCZ	TS	0.0852	0.036	2.3673	0.0179	0.021402174
BDI	TS	0.0265	0.0482	0.5491	0.5829	0.593694444
ADHD	AN	-0.0044	0.049	-0.0908	0.9276	0.9276
ADHD	OCD	-0.1691	0.074	-2.2864	0.0222	0.025978723

Abbreviations. rg, genetic correlation; FDR, false discovery rate; s.e., standard error;

SCZ, schizophrenia; BD, bipolar disorder; MDD, major depressive disorder; ASD, autism spectrum disorder;

ADHD, attention deficit/hyperactivity disorder; TS, Tourette's Syndrome; OCD, obsessive-compulsive disorder; AN, anorexia nervosa.

Supplementary Table 12. Univariate analysis (MiXeR)

Disorder	pi		sig2_beta		sig2_zero		h2		nc@p9		AIC	BIC
	mean	std	mean	std	mean	std	mean	std	mean	std		
Irritable	0.002024941	1.06E-04	1.18E-05	5.76E-07	0.971051041	0.003433659	0.049442092	0.000944041	6457.772432	337.2188076	75.87636223	66.37748051
ADHD	0.001823311	0.000142273	5.80606E-05	3.35174E-06	1.086338093	0.004407922	0.21863146	0.005853738	5814.752895	453.7253525	28.69427844	19.35529572
AN	0.002385686	0.000169734	4.33954E-05	2.84941E-06	1.072838978	0.003411482	0.213791365	0.004647011	7608.229578	541.3023511	18.20380961	8.864119256
ASD	0.003845357	0.000456075	2.38889E-05	2.74228E-06	1.043823949	0.00304213	0.188022456	0.005130514	12263.29232	1454.47502	1.763358661	-7.631356267
BD I	0.002311693	6.21E-05	4.94049E-05	1.27E-06	1.124293141	2.98E-03	0.236747106	2.54E-03	7372.258265	198.0783798	128.4989666	119.0342337
BD II	0.004333057	0.003911701	1.90498E-05	9.28604E-06	1.061904568	0.003325115	0.114395643	0.009212311	13818.62436	12474.87085	-1.737004204	-11.20651338
MDD	0.003861685	0.000144143	8.1254E-06	2.78996E-07	1.107111653	0.004406854	0.065006007	0.000787269	12315.36472	459.6899048	105.5140851	96.04884655
OCD	0.000613992	9.8721E-05	0.00025072	3.35869E-05	1.011613436	0.002517721	0.312980569	0.018143818	1958.092746	314.832937	0.112915538	-9.274611498
SCZ	0.003026915	7.44248E-05	6.78402E-05	1.715E-06	1.207951282	0.002851033	0.425683242	0.002964699	9653.184339	237.3493421	308.6044583	299.0738262
TS	0.002749228	0.00084363	5.86289E-05	1.70378E-05	1.04246749	0.003234737	0.306514453	0.018519421	8767.609057	2690.434148	-1.25701962	-10.69084673

Abbreviations. pi, polygenecity; sig2_beta, discoverability; sig2_zero, variance distortion; h2, heritability; nc@p9, number of causal variants that account for 90% of heritability; AIC, akaike information criterion; BIC, bayesian information criterion.

Supplementary Table 13. Bivariate analysis between irritability and psychiatric disorders (MiXeR)

Trait1	Trait2	dice		pi1		pi2		pi12		nc1@p9		nc2@p9		nc12@p9	
		mean	std	mean	std	mean	std	mean	std	mean	std	mean	std	mean	std
Irritable	ADHD	0.449277246	0.208429306	0.001165555	0.000396764	0.000963925	0.000452246	0.000859386	0.000388	3717.089451	1265.328093	3074.069914	1442.264559	2740.682981	1237.37605
Irritable	AN	0.886447743	0.041420266	7.10529E-05	7.55417E-05	0.000431798	0.000194722	0.001953888	0.000100343	226.5961103	240.9112101	1377.053256	620.9910332	6231.176321	320.0051088
Irritable	ASD	0.682285833	0.057008937	3.29173E-05	2.52572E-05	0.001853334	0.000464558	0.001992023	0.000109364	104.9771105	80.54826602	5910.497001	1481.530178	6352.795321	348.7744685
Irritable	BD I	0.881572095	0.054096252	0.000111825	9.96894E-05	0.000398577	0.000154478	0.001913116	0.00015171	356.6229165	317.920987	1271.10875	492.6490699	6101.149515	483.8213607
Irritable	BD II	0.489920272	0.172328534	0.000654398	0.000435947	0.002962514	0.003703896	0.001370543	0.000423569	2086.951915	1390.285618	9447.803844	11812.15691	4370.820516	1350.811225
Irritable	MDD	0.561345402	0.011886003	0.000373242	8.00783E-05	0.002209987	0.000125441	0.001651698	4.94678E-05	1190.313465	255.3790355	7047.905754	400.0470852	5267.458967	157.7585552
Irritable	OCD	0.355043636	0.085716441	0.001556889	0.000170154	0.000145941	0.000105763	0.000468052	0.000112427	4965.101266	542.6425227	465.421581	337.290892	1492.671165	358.5420851
Irritable	SCZ	0.782194974	0.027019699	4.83939E-05	4.36743E-05	0.001050368	0.000112448	0.001976547	0.00010419	154.333808	139.2825579	3349.745716	358.6103536	6303.438624	332.2740025
Irritable	TS	0.728229083	0.185546241	0.000289358	0.00040466	0.001013645	0.000736349	0.001735583	0.000446798	922.7950314	1290.507862	3232.631657	2348.303858	5534.9774	1424.892277

Trait1	Trait2	rho_zero		rho_beta		rg		fraction_concordant_within_shared		best_vs_min_AIC	best_vs_min_BIC	best_vs_max_AIC	best_vs_max_BIC
		mean	std	mean	std	mean	std	mean	std				
Irritable	ADHD	0.024441704	0.002343694	0.760551614	0.256064431	0.290291031	0.0122724	0.80539599	0.125051788	-0.690828272	-9.993938031	0.749300816	-8.553808943
Irritable	AN	0.017434154	0.00228017	0.174657097	0.016810503	0.155148551	0.012634123	0.555889886	0.005433997	8.674832734	-0.623493261	-1.275272866	-10.57359886
Irritable	ASD	0.007102074	0.002352184	0.379806109	0.031767845	0.271463875	0.017644888	0.624085913	0.010924066	0.661605841	-8.687058562	-1.424465453	-10.77312986
Irritable	BD I	0.016997465	0.001932162	0.147310361	0.015105066	0.129668869	0.010010601	0.547067199	0.004863064	18.23126437	8.829257094	-0.588155874	-9.990163154
Irritable	BD II	0.016178288	0.002092853	0.843127648	0.177350696	0.410759703	0.031462343	0.851229092	0.108619055	-1.151090814	-10.55678521	-1.068486644	-10.47418104
Irritable	MDD	0.147652265	0.002317618	0.990991988	0.008118701	0.585755791	0.010337223	0.961204818	0.018037038	-0.88276543	-10.34786029	2.220469827	-7.244625028
Irritable	OCD	0.004676051	0.001864543	0.606547959	0.162109804	0.24159985	0.027197386	0.716766358	0.081132691	-0.861432707	-10.20662307	-0.9776683	-10.32285866
Irritable	SCZ	0.00824393	0.002408242	0.158115917	0.014037578	0.126051018	0.009734763	0.550547138	0.004526131	29.05238419	19.6135074	-0.672004297	-10.1108811
Irritable	TS	0.004027877	0.001638258	0.21400052	0.102715744	0.141981654	0.019964672	0.569295306	0.03557131	-0.219747008	-9.604288989	-0.882225558	-10.26676754

Abbreviations. dice, a proportion of shared variants to the whole variants; pi1, the polygenicity of trait1; pi2, the polygenicity of trait2; pi12, the shared polygenicity of trait1 and trait2;

nc1@p9, the number of causal variants for trait1; nc2@p9, the number of causal variants for trait2; nc12@p9, the number of causal variants for both traits; rho_zero, capturing correlation between z-score;

rho_beta, the correlation of effect sizes among the shared variants; rg, genetic correlation; fraction_concordant_within_shared, the proportion of shared variants which have same effect directions;

best_vs_min_AIC(BIC), the value indicating whether the reported model shows better polygenic overlap than the model with minimum possible overlap;

bes_vs_max_AIC(BIC), the value indicating whether the reported model shows better results than the model with maximum possible overlap.

Supplementary Table 14. Distinct genomic loci associated with schizophrenia at condFDR<0.01 given association with irritability

Locusnum	CHR	Lead SNP	Lead BP	Mininum BP	Maximum BP	condFDR	Status
1	1	rs4648845	2387101	2369498	2402499	1.36E-05	replicated loci of SCZ
2	1	rs11122119	6768856	6644723	6793473	0.00422	replicated loci of SCZ(2022)
3	1	rs301818	8503242	8355669	8888842	0.000111	replicated loci of SCZ
4	1	rs79598313	27284913	26933591	27368126	0.00601	additional loci
5	1	rs6694545	30437268	30412503	30459412	2.99E-05	replicated loci of SCZ
6	1	rs1211184	35888147	35703750	36130279	0.00352	replicated loci of SCZ(2022)
7	1	rs3001723	44037685	44018604	44480093	4.76E-05	replicated loci of SCZ
8	1	rs12138061	50572096	49573780	50591851	0.000435	additional loci
9	1	rs17785382	53707953	53658317	53751610	0.00437	additional loci
10	1	rs12129719	66324512	66304167	66553495	0.00179	replicated loci of SCZ
11	1	rs7531932	72134585	72118484	72226638	0.00112	replicated loci of SCZ(2022)
12	1	rs4571923	73736562	73275828	74108971	4.93E-08	replicated loci of SCZ
13	1	rs6680011	95840866	95838722	95944432	0.00353	replicated loci of SCZ
14	1	rs59274111	97123431	97113731	97284496	0.0076	replicated loci of SCZ
15	1	rs9729897	98391864	97773324	98559093	2.12E-08	replicated loci of SCZ
16	1	rs3008527	112523095	112523095	112536235	0.00894	additional loci
17	1	rs55675132	115619634	115496981	115647658	0.00984	additional loci
18	1	rs140505938	150031490	149998923	151134961	0.000162	replicated loci of SCZ
19	1	rs869506	154859581	154834092	154980351	0.00235	replicated loci of SCZ(2022)
20	1	rs56407270	166902106	166844024	166940899	0.00905	additional loci
21	1	rs61826828	174015261	173580303	174951357	0.00205	replicated loci of SCZ
22	1	rs4650963	177309490	177237533	177428790	0.000312	replicated loci of SCZ
23	1	rs10801008	190995271	190606775	191091588	0.000569	replicated loci of SCZ
24	1	rs6678676	200266503	200253612	200269903	0.00431	replicated loci of SCZ
25	1	rs41314284	226790001	226774042	226790001	0.00581	replicated loci of SCZ(2022)

26	1	rs11807834	230272624	230230169	230272624	0.00106	additional loci
27	1	rs72769124	239210058	239171701	239298749	0.000183	replicated loci of SCZ
28	1	rs10803138	243555219	243234941	244101789	9.22E-05	replicated loci of SCZ
29	2	rs12712510	22749726	22452259	22754466	0.00151	replicated loci of SCZ
30	2	rs12474906	28033538	27961614	28281545	0.00333	replicated loci of SCZ(2022)
31	2	rs2471403	48490508	48178775	48734773	0.000473	additional loci
32	2	rs148661029	54972226	54688458	55055129	0.00864	additional loci
33	2	rs2868985	56191470	56149475	56197276	0.00205	additional loci
34	2	rs7596038	58383820	57931347	58505679	8.37E-08	replicated loci of SCZ
35	2	rs12328348	60785937	60777498	60792671	0.00219	replicated loci of SCZ(2022)
36	2	rs13416544	62721896	62708063	62723220	0.00917	additional loci
37	2	rs2077586	73161551	73129974	73168593	0.00208	replicated loci of SCZ
38	2	rs56145559	73623439	73552542	73900900	0.000437	replicated loci of SCZ
39	2	rs9330316	110284236	110262077	110398109	0.00213	additional loci
40	2	rs6430491	134840967	134839539	134859017	0.00976	replicated loci of SCZ(2022)
41	2	rs12991836	145141541	145139727	145202375	0.000424	replicated loci of SCZ
42	2	rs6430095	146439945	146416874	146441828	4.49E-05	replicated loci of SCZ
43	2	rs12464452	147625594	147567572	147722458	0.00909	additional loci
44	2	rs4131583	175082308	174931752	175091630	0.00662	replicated loci of SCZ(2022)
45	2	rs62176209	185519428	185406883	185926285	3.33E-05	replicated loci of SCZ
46	2	rs35610290	194378903	194336245	194678448	0.00106	replicated loci of SCZ(2022)
47	2	rs6434928	198304577	198144002	198954774	1.11E-07	replicated loci of SCZ
48	2	rs2949006	200715388	199893814	201333001	1.24E-06	replicated loci of SCZ
49	2	rs6435711	213410065	213402472	213415737	0.00664533	additional loci
50	2	rs11685299	225391296	225334070	225467840	0.000919275	replicated loci of SCZ
51	2	rs11688570	229317492	229260731	229337807	0.000235235	replicated loci of SCZ(2022)
52	2	rs1083515	233789463	233550961	233807585	2.07E-06	replicated loci of SCZ
53	3	rs17194490	2547786	2436014	2576606	8.84E-06	replicated loci of SCZ
54	3	rs6800435	10804551	10783461	10871860	0.001095802	replicated loci of SCZ

55	3	rs9881798	16846967	16819132	17888256	9.09E-05	replicated loci of SCZ
56	3	rs9310732	24215557	24086516	24239153	0.003329451	additional loci
57	3	rs6549963	30044778	30022950	30046687	0.005617833	replicated loci of SCZ(2022)
58	3	rs75968099	36858583	36834099	36964583	8.90E-05	replicated loci of SCZ
59	3	rs62253224	44075602	44062007	44083403	0.004582482	additional loci
60	3	rs3617	52833805	52217088	53539241	2.01E-06	replicated loci of SCZ
61	3	rs6780613	60291127	60287845	60293004	0.002540408	replicated loci of SCZ
62	3	rs832190	63842629	63792668	64243673	0.000142068	replicated loci of SCZ
63	3	rs6778026	71571386	71248393	71611630	0.000568931	replicated loci of SCZ
64	3	rs59888335	80660050	80395885	81077325	0.006621931	replicated loci of SCZ(2022)
65	3	rs836927	107201428	107187344	107379837	0.007014383	replicated loci of SCZ(2022)
66	3	rs9845387	116425935	116425935	116432671	0.005280694	additional loci
67	3	rs7432375	136288405	135814009	136712852	8.16E-06	replicated loci of SCZ
68	3	rs6768184	161491740	161394234	161518228	0.001304398	replicated loci of SCZ
69	3	rs34796896	180623255	180524764	181245594	4.27E-06	replicated loci of SCZ
70	4	rs215412	23423586	23366446	23443426	0.003124069	replicated loci of SCZ
71	4	rs13145415	30823157	30596040	30951649	0.003508191	additional loci
72	4	rs148353991	55049167	55039742	55053528	0.007211614	additional loci
73	4	rs6855246	103112470	102702364	103387161	2.91E-06	replicated loci of SCZ
74	4	rs2905627	105450085	105447833	105512391	0.009233618	replicated loci of SCZ(2022)
75	4	rs13121251	143829759	143742567	143895198	0.002544359	replicated loci of SCZ
76	4	rs10520163	170626552	170198392	170646003	0.001995082	replicated loci of SCZ
77	4	rs62334820	176855221	176717618	176970155	2.06E-06	replicated loci of SCZ
78	5	rs1549250	37821221	37809669	37833419	0.001618183	additional loci
79	5	rs10473396	45882608	44637587	50161542	7.67E-05	replicated loci of SCZ
80	5	rs4391122	60598543	60135962	60844213	1.70E-06	replicated loci of SCZ
81	5	rs16867576	88746331	86494363	88871993	5.01E-05	replicated loci of SCZ
82	5	rs35527617	92458257	92363455	92484054	0.006528982	additional loci
83	5	rs7708343	107205182	107145324	107256585	0.005410508	replicated loci of SCZ(2022)

84	5	rs4388249	109036066	109030041	109209342	0.005832878	replicated loci of SCZ(2022)
85	5	rs3849046	137851192	137598340	137948140	0.000401592	replicated loci of SCZ
86	5	rs35123781	139065180	139037130	139086651	0.009161999	replicated loci of SCZ(2022)
87	5	rs270170	139541460	139517048	139685295	0.005675694	replicated loci of SCZ(2022)
88	5	rs145747680	151414297	151414297	151437972	0.009665921	replicated loci of SCZ
89	5	rs111294930	152177121	151874412	152899532	3.58E-06	replicated loci of SCZ
90	5	rs6862346	153507705	153360230	153687444	0.001418532	replicated loci of SCZ(2022)
91	5	rs12652777	155775075	155731511	155915825	0.00246521	replicated loci of SCZ(2022)
92	6	rs5021453	32708124	24988105	33791998	1.74E-07	additional loci
93	6	rs113113059	43160375	43151439	43185733	0.003533782	replicated loci of SCZ(2022)
94	6	rs2789605	73155289	73132745	73171881	0.000171772	replicated loci of SCZ
95	6	rs217287	84407466	83789798	84409255	3.12E-06	replicated loci of SCZ
96	6	rs634940	93077500	93045430	93177270	0.000181645	replicated loci of SCZ
97	6	rs62420371	98360026	98228405	98777764	0.005233129	additional loci
98	6	rs2205735	114705028	114679177	114722063	0.001744004	replicated loci of SCZ
99	6	rs7742212	128328833	128301981	128337810	0.004361989	replicated loci of SCZ
100	6	rs1849527	130606808	130544509	130772152	0.008825041	replicated loci of SCZ(2022)
101	6	rs9403484	143651969	143645035	143709107	0.002443988	replicated loci of SCZ
102	6	rs1961522	158067416	158061062	158085905	0.009683521	additional loci
103	6	rs6919939	164975014	164974975	165025805	0.004803197	additional loci
104	7	rs3800917	2167939	1851205	2323488	6.81E-07	replicated loci of SCZ
105	7	rs12672629	21531831	21470536	21552995	0.001034468	replicated loci of SCZ(2022)
106	7	rs10262624	23915676	23722139	23927052	0.002426317	replicated loci of SCZ
107	7	rs2237326	24797764	24619811	24833601	0.001343713	replicated loci of SCZ
108	7	rs7786896	39093928	39093791	39093928	0.008978027	additional loci
109	7	rs2944815	71811748	71682643	71872935	0.007421837	replicated loci of SCZ(2022)
110	7	rs7791589	78319728	78316766	78414989	0.003782562	additional loci
111	7	rs2888019	82503409	82386473	82575558	0.003026124	additional loci
112	7	rs6958190	83653584	83576015	83740030	0.00796163	additional loci

113	7	rs12704289	86422141	86199080	87221216	6.80E-07	replicated loci of SCZ
114	7	rs492430	100313099	100219167	100330091	0.002763728	additional loci
115	7	rs7796558	103730378	103709196	103845603	0.006414454	replicated loci of SCZ(2022)
116	7	rs7789569	104927586	104454298	105063372	0.000127418	replicated loci of SCZ
117	7	rs211829	110048893	110034378	110106697	0.000988751	replicated loci of SCZ
118	7	rs7803571	110993511	110785679	111236477	6.15E-05	replicated loci of SCZ
119	7	rs35297615	127284269	127272090	127788550	0.001949251	additional loci
120	7	rs7801375	131567263	131533769	131627573	0.000265885	replicated loci of SCZ
121	7	rs3735025	137074844	137039328	137085250	2.73E-06	replicated loci of SCZ
122	7	rs6662	140706157	140665521	140791820	0.006814159	additional loci
123	7	rs1583830	154836010	154800482	154872803	0.008950619	additional loci
124	8	rs10503253	4180844	4177791	4208761	0.001285013	replicated loci of SCZ
125	8	rs11993663	10032894	8088230	10283748	0.000811611	replicated loci of SCZ
126	8	rs7002619	16065557	16007395	16071273	0.000885026	additional loci
127	8	rs1043083	17087014	17059044	17178237	0.00151121	additional loci
128	8	rs2410572	18421474	18396405	18458140	0.000418568	replicated loci of SCZ
129	8	rs1042992	26269191	26190836	26279173	0.001318505	replicated loci of SCZ
130	8	rs11783093	27425349	27327841	27470778	2.07E-05	replicated loci of SCZ
131	8	rs55669358	34312412	33863561	34734293	0.001836608	replicated loci of SCZ
132	8	rs7845911	38135412	38014429	38310910	7.13E-06	replicated loci of SCZ
133	8	rs12674620	52667603	52536828	52842880	0.00120759	additional loci
134	8	rs1473594	60696526	60475926	60956071	3.75E-06	replicated loci of SCZ
135	8	rs11777872	64525347	64496159	64842662	0.001646135	replicated loci of SCZ(2022)
136	8	rs4246111	89342516	89188454	89761163	0.000188801	replicated loci of SCZ
137	8	rs7817028	93216034	93190014	93251556	0.005692716	additional loci
138	8	rs36043959	111472014	111460027	112019813	8.77E-06	replicated loci of SCZ
139	8	rs800886	116468203	116464988	116632819	0.00317468	additional loci
140	8	rs717349	118534875	118486861	118619727	0.00544912	additional loci
141	8	rs4733770	131152665	130853447	131361477	0.000368888	additional loci

142	8	rs62512616	143297663	143267749	143750619	1.20E-06	replicated loci of SCZ
143	8	rs7835528	144243392	144238923	144270473	0.008191044	replicated loci of SCZ
144	8	rs7829793	144848647	144799465	144866918	0.005934154	replicated loci of SCZ(2022)
145	9	rs913588	7174673	7171606	7186083	0.009212013	additional loci
146	9	rs1318729	14515364	14504554	14521627	0.005751565	replicated loci of SCZ(2022)
147	9	rs10967586	26895808	26757269	26941604	0.005168545	additional loci
148	9	rs10124101	36308827	36301075	36319928	0.003486453	replicated loci of SCZ(2022)
149	9	rs12554874	37073902	37073902	37379492	0.009260808	replicated loci of SCZ(2022)
150	9	rs1319017	84736303	84607758	85129970	2.43E-06	replicated loci of SCZ
151	9	rs10985817	101071090	101061951	101079033	0.00022904	replicated loci of SCZ
152	10	rs7893279	18745105	18538669	18971121	3.45E-06	replicated loci of SCZ
153	10	rs12244388	104640052	104229588	105274900	2.12E-08	replicated loci of SCZ
154	11	rs72875860	24406212	24367339	24412992	0.000218832	replicated loci of SCZ
155	11	rs1765142	30378559	30217503	30435051	0.000770439	replicated loci of SCZ
156	11	rs7951870	46373311	46276072	47371598	2.92E-06	replicated loci of SCZ
157	11	rs7129727	57484660	57369008	57681828	1.02E-05	replicated loci of SCZ
158	11	rs74896026	64684990	64590854	64787880	0.008188141	replicated loci of SCZ
159	11	rs1784223	65380248	65378028	65485218	0.003357928	replicated loci of SCZ
160	11	rs7927922	68317515	68192362	68395190	0.009730271	additional loci
161	11	rs12294291	83197722	82987208	83334530	0.006843439	additional loci
162	11	rs2514218	113392994	113317745	113451589	8.51E-08	replicated loci of SCZ
163	11	rs7927176	123395864	123394636	123395987	0.007153292	replicated loci of SCZ
164	11	rs55661361	124613957	124602074	124653926	5.90E-07	replicated loci of SCZ
165	11	rs10791097	130718630	130704653	130894131	1.03E-05	replicated loci of SCZ
166	11	rs2917569	132568255	132387460	132581442	0.000145445	replicated loci of SCZ
167	11	rs73034295	133822133	133701956	134297345	2.77E-08	replicated loci of SCZ
168	12	rs7965923	2341385	2285731	2523772	2.12E-08	replicated loci of SCZ
169	12	rs2466979	23040167	23027722	23068137	0.009349697	replicated loci of SCZ
170	12	rs1120004	23633432	23477601	23638641	0.00160341	replicated loci of SCZ

171	12	rs10783624	39522907	39448519	39533484	0.001011309	replicated loci of SCZ
172	12	rs3214023	53682986	53630558	53698903	0.006360425	replicated loci of SCZ(2022)
173	12	rs61937595	57682956	57331741	57851182	5.74E-06	replicated loci of SCZ
174	12	rs10745572	92252357	92243186	92258265	0.003419152	replicated loci of SCZ
175	12	rs10745694	95181478	95153715	95231062	0.005008208	replicated loci of SCZ(2022)
176	12	rs1492251	99624271	99436519	99691448	0.003751785	replicated loci of SCZ(2022)
177	12	rs35251637	103380562	103354331	103382325	0.002020476	replicated loci of SCZ
178	12	rs35393419	109881809	109791620	109884367	0.008221861	replicated loci of SCZ
179	12	rs4766428	110723245	110336546	111273197	1.30E-06	replicated loci of SCZ
180	12	rs10734901	123743447	123447928	123913433	1.20E-06	replicated loci of SCZ
181	13	rs1330975	38819860	38769016	38872838	0.004433147	replicated loci of SCZ(2022)
182	13	rs4627212	58822051	58719899	58851582	0.00628489	replicated loci of SCZ(2022)
183	13	rs7982263	80061074	79858235	80166707	0.001024226	replicated loci of SCZ
184	13	rs55968332	111529924	111529924	111544842	0.002748066	additional loci
185	13	rs6560936	114945972	114897112	115047700	0.001709942	additional loci
186	14	rs2319380	21147845	21147845	21155393	0.006804406	additional loci
187	14	rs10148671	29469373	29469373	29547008	0.000441454	replicated loci of SCZ
188	14	rs1191551	30000405	30000405	30287870	0.000222419	replicated loci of SCZ
189	14	rs12883788	33303540	33257822	33309495	0.004720673	replicated loci of SCZ
190	14	rs7140760	34253107	34242983	34256251	0.007344157	replicated loci of SCZ
191	14	rs150437760	59981768	59864362	60033892	0.004496816	replicated loci of SCZ
192	14	rs2190864	72416219	72387640	72467631	9.28E-06	replicated loci of SCZ
193	14	rs3742658	91123844	91122317	91123844	0.004756691	replicated loci of SCZ(2022)
194	14	rs35604463	99712032	99662868	99751267	6.14E-05	replicated loci of SCZ
195	14	rs4906337	104040414	103849715	104537680	1.33E-06	replicated loci of SCZ
196	15	rs7403630	34654165	34654165	34656627	0.005185625	replicated loci of SCZ(2022)
197	15	rs55927878	40573201	40566759	40602256	0.002543787	replicated loci of SCZ
198	15	rs11854184	49293194	49293194	49840088	0.00649073	additional loci
199	15	rs12898315	61854003	61831680	61909712	0.000704934	replicated loci of SCZ

200	15	rs12148337	70589272	70573650	70628849	0.001054976	replicated loci of SCZ
201	15	rs8042374	78908032	78711803	78936168	3.17E-06	replicated loci of SCZ
202	15	rs1259180	83244680	82431219	83406228	0.000352562	replicated loci of SCZ
203	15	rs71395431	84806031	84641125	85392298	0.00034815	replicated loci of SCZ
204	15	rs17514846	91416550	91403674	91439417	1.43E-07	replicated loci of SCZ
205	15	rs12914508	93455982	93455982	93561966	0.001679976	additional loci
206	16	rs2270366	4558615	4447771	4596447	0.007592086	replicated loci of SCZ(2022)
207	16	rs8054347	6717757	6713777	6730845	0.008495857	additional loci
208	16	rs12447542	7744180	7736155	7758258	0.001016679	replicated loci of SCZ
209	16	rs7191183	9900057	9875513	9960879	0.001645171	replicated loci of SCZ
210	16	rs7499750	13749265	13616231	13763942	3.60E-05	replicated loci of SCZ
211	16	rs3814883	29994922	29923510	30141913	7.56E-06	replicated loci of SCZ
212	16	rs42945	58545426	58539182	58684999	0.000145791	replicated loci of SCZ
213	16	rs17465671	63712719	63692643	63769815	0.000837401	replicated loci of SCZ
214	16	rs2917704	64305723	64257414	64375453	0.008585268	replicated loci of SCZ
215	16	rs10852439	68288331	67708897	68419298	0.000416389	replicated loci of SCZ
216	16	rs8049057	69210661	69141138	69432250	0.001860063	replicated loci of SCZ
217	16	rs2161711	71359066	71176126	71439049	0.000328661	replicated loci of SCZ
218	17	rs4523957	2208899	2095954	2220815	0.00038323	replicated loci of SCZ
219	17	rs6502460	2580265	2532052	2586229	0.009932076	replicated loci of SCZ
220	17	rs2085045	4040675	3922748	4069735	0.004306496	additional loci
221	17	rs17512210	11230466	11210835	11234195	0.005441802	additional loci
222	17	rs9907138	12866090	12851454	12880010	0.00300784	additional loci
223	17	rs8082590	17958402	17661802	18030240	0.002819538	replicated loci of SCZ
224	17	rs4273100	19153417	18916893	19303635	0.001113384	replicated loci of SCZ
225	17	rs8067545	19912710	19879164	20384834	0.009220894	replicated loci of SCZ
226	17	rs62063686	44132458	43463493	44865603	0.004785566	replicated loci of SCZ(2022)
227	17	rs9916785	78499975	78454756	78706517	0.000786049	replicated loci of SCZ
228	18	rs34777351	23106223	23071420	23197631	0.007395599	additional loci

229	18	rs8086342	31603023	31525209	31603023	0.007440107	additional loci
230	18	rs4632195	50746748	50555931	51055069	0.000390358	replicated loci of SCZ(2022)
231	18	rs28758902	53408187	52716306	53804156	2.51E-08	replicated loci of SCZ
232	18	rs337718	69774278	69747467	69871763	0.006444329	replicated loci of SCZ(2022)
233	18	rs56775891	77575613	77551586	77654017	0.000122654	replicated loci of SCZ
234	19	rs72986630	11849736	11849736	11849736	0.000505759	replicated loci of SCZ
235	19	rs2905432	19484295	19357612	19744079	2.83E-05	replicated loci of SCZ
236	19	rs2053079	30987423	30981639	31051857	3.26E-05	replicated loci of SCZ
237	19	rs34822974	33897713	33812241	34023996	0.000474024	additional loci
238	19	rs7508148	50152142	50067508	50182697	9.04E-05	replicated loci of SCZ
239	20	rs6137163	20820956	20456417	20830612	0.001283426	replicated loci of SCZ
240	20	rs6028163	37477034	37250470	37506313	1.81E-06	replicated loci of SCZ
241	20	rs495146	48130328	48085776	48132580	0.0033254	replicated loci of SCZ(2022)
242	20	rs4610119	53861763	53788474	53875983	0.009532017	additional loci
243	21	rs1482419	22117491	22084693	22169734	0.005157196	replicated loci of SCZ(2022)
244	21	rs4484102	25103088	25093768	25105211	0.008059362	additional loci
245	22	rs8137258	20135961	20057078	20169422	0.001088926	additional loci
246	22	rs116620085	39238455	39173792	39259626	0.00621533	replicated loci of SCZ
247	22	rs5750857	40000221	39869209	40091818	1.34E-06	replicated loci of SCZ
248	22	rs71327107	41418229	41027819	41854446	3.66E-06	replicated loci of SCZ
249	22	rs134873	42657566	42192355	42730970	8.10E-07	replicated loci of SCZ
250	22	rs9615807	48614954	48597454	48638662	0.009628229	additional loci
251	22	rs5769765	50271176	50162136	50340034	0.002167556	replicated loci of SCZ(2022)

Abbreviations: CHR, Chromosome; BP, genomic position in human genome assembly; condFDR, conditional false discovery rate.

* SCZ(2022) indicates Trubetskoy, Vassily, et al., 2022

Supplementary Table 15. Distinct genomic loci associated with bipolar I disorder at condFDR<0.01 given association with irritability

Locusnum	CHR	Lead SNP	Lead BP	Mininum BP	Maximum BP	condFDR	Status
1	1	rs10917509	19992066	19986009	20023833	0.000196	replicated loci of BDI
2	1	rs12134005	42165629	42165629	42165629	0.00302	additional loci
3	1	rs2211577	79241577	79186203	79261383	0.00356	additional loci
4	1	rs7528296	172381383	172349246	172453840	0.00295	additional loci
5	1	rs7536102	174465939	173968516	174996637	0.00482	additional loci
6	2	rs1510606	21531266	21527036	21534298	0.00376	replicated loci of BDI
7	2	rs11889709	81755599	81641348	81880889	0.00523	additional loci
8	2	rs4619651	97416153	97349315	97521748	0.000996	replicated loci of BDI
9	2	rs1976772	198868201	198215056	198952637	0.0092	additional loci
10	2	rs4676412	241553492	241525316	241558146	0.00408	replicated loci of BDI
11	3	rs735931	10525096	10481537	10525468	0.00175	replicated loci of BDI
12	3	rs2697137	11077262	11076907	11092264	0.000934	replicated loci of BDI
13	3	rs4624519	36862980	36834099	36964583	4.49E-06	replicated loci of BDI
14	3	rs2336147	52626443	52217088	53101580	0.000232	replicated loci of BDI
15	3	rs6806239	70488207	70472539	70593081	0.00904	additional loci
16	3	rs9831123	85052150	85002871	85789644	0.000758	replicated loci of BDI
17	3	rs9812434	153184301	153170143	153211841	0.00689	additional loci
18	3	rs10937550	192224548	192212630	192232978	0.00169	additional loci
19	4	rs1501112	101499811	101463177	101593148	0.00826	additional loci
20	4	rs1948223	118545387	118541595	118547300	0.00135	additional loci
21	4	rs112481526	123076007	123026869	123558330	0.000146	replicated loci of BDI
22	5	rs78308718	7533985	7493177	7587233	2.81E-06	replicated loci of BDI
23	5	rs6865469	78849505	78801947	78849697	0.00149	replicated loci of BDI
24	5	rs6897189	80792321	80728732	80969507	0.00327	additional loci
25	5	rs10059194	94140680	94088219	94141447	0.00779	additional loci

26	5	rs10043984	137712121	137598340	137750058	0.000349	replicated loci of BDI
27	5	rs6875495	140107231	139719983	140142174	0.000331	replicated loci of BDI
28	5	rs55737372	152097562	151924995	152339648	0.0062	additional loci
29	5	rs7702334	165701326	165674398	165750226	0.00117	additional loci
30	5	rs261616	169232623	169213476	169233136	0.00632	additional loci
31	6	rs6914824	27139048	25961352	31408329	0.000342	replicated loci of BDI
32	6	rs427037	32212264	32201469	32212867	0.000595	additional loci
33	6	rs59137082	33732365	33715323	33774394	0.00859	additional loci
34	6	rs1180184	83970665	83915335	84154298	0.0078	additional loci
35	6	rs12202969	98576223	98547979	98693636	2.66E-06	replicated loci of BDI
36	6	rs6456095	166984094	166973449	167018915	2.62E-05	replicated loci of BDI
37	7	rs6461049	2017445	1852193	2323488	2.09E-06	replicated loci of BDI
38	7	rs113779084	11871787	11855103	11994774	1.64E-05	replicated loci of BDI
39	7	rs4722033	21499828	21468640	21555536	2.21E-05	replicated loci of BDI
40	7	rs12672003	24647222	24619811	24833601	0.00887	additional loci
41	7	rs11560354	76314260	76286264	76389882	0.00922	additional loci
42	7	rs2966424	110189944	110164447	110237982	0.00633	additional loci
43	7	rs62474680	115016799	114949746	115114049	0.00524	additional loci
44	7	rs10225324	131879665	131859077	131887029	0.00161	additional loci
45	7	rs4726225	140758393	140665521	140805649	0.000419	additional loci
46	8	rs3088186	10226355	9695742	10283602	5.33E-05	replicated loci of BDI
47	8	rs12675289	25255017	25243213	25389631	0.00466	additional loci
48	8	rs6983908	145009593	144973183	145086428	2.75E-05	replicated loci of BDI
49	9	rs1327046	22241938	22241889	22331418	0.00914	additional loci
50	10	rs11596252	18755989	18680963	18782777	0.00138	replicated loci of BDI
51	10	rs1380459	62097331	62060128	62326687	0.000592	replicated loci of BDI
52	10	rs7068482	111729503	111648659	111928784	0.000183	replicated loci of BDI
53	11	rs1481881	13269536	13268386	13350131	0.000296	additional loci
54	11	rs144225206	45844797	45780039	45914046	0.000594	additional loci

55	11	rs174592	61618608	61405178	61624181	2.12E-07	replicated loci of BDI
56	11	rs489337	65854561	65814842	66794108	6.80E-05	replicated loci of BDI
57	11	rs12575685	70517927	70517927	70563919	5.84E-05	replicated loci of BDI
58	11	rs11018471	88831103	88767496	88976157	0.00816	additional loci
59	11	rs9943574	112944821	112914680	113034787	0.00659	additional loci
60	11	rs893949	134296384	134290032	134297345	0.00147	additional loci
61	12	rs769087	2344644	2285731	2420526	2.46E-08	replicated loci of BDI
62	12	rs4760255	57855180	57853153	57870463	0.000607	additional loci
63	12	rs12315174	110586278	110294902	111199274	0.00316	replicated loci of BDI
64	13	rs3803277	31318308	31318020	31337558	0.00207	replicated loci of BDI
65	13	rs1924817	31843598	31842146	31941945	0.00474	replicated loci of BDI
66	13	rs9521162	109729891	109711644	109750926	0.00137	additional loci
67	13	rs9549704	113885187	113818708	113911532	0.00639	additional loci
68	14	rs1957760	25313865	25274272	25324716	0.000877	additional loci
69	14	rs10131905	62425610	62421822	62497042	0.00491	additional loci
70	14	rs4900114	92795735	92760416	92795912	0.00184	additional loci
71	14	rs2693698	99719219	99667179	99719219	0.000587	additional loci
72	15	rs8036248	29858565	29848687	29858805	0.00529	additional loci
73	15	rs6495988	38969545	38935180	38999394	0.000126	replicated loci of BDI
74	15	rs1197546	42902246	42457582	43396604	2.09E-05	replicated loci of BDI
75	15	rs1715887	56816155	56788798	56823787	0.00266	additional loci
76	15	rs3145	59212111	59136688	59272096	0.00928	additional loci
77	15	rs1452386	74148987	74099922	74165781	0.000899	replicated loci of BDI
78	15	rs11856299	83534421	83523163	83977166	0.0061	additional loci
79	15	rs12914433	85184963	84641125	85392298	6.51E-05	replicated loci of BDI
80	15	rs4702	91426560	91416550	91429042	0.006	additional loci
81	16	rs12103041	61727983	61650112	61761418	0.00432	additional loci
82	16	rs1424000	64821351	64722315	64874642	0.00299	additional loci
83	16	rs12925547	69241887	69141138	69432250	0.00801	additional loci

84	17	rs4790841	1835482	1816762	1859542	0.000447	additional loci
85	17	rs12603807	11233901	11210835	11234195	0.00808	additional loci
86	17	rs11650008	34892731	34825861	34961051	0.000232	replicated loci of BDI
87	17	rs7502233	38218804	38128181	38219005	0.00846	additional loci
88	17	rs4473241	42281282	42156471	42330171	0.000555	replicated loci of BDI
89	17	rs9897752	78484082	78463585	78706517	0.0049	additional loci
90	19	rs1975161	10805160	10744807	10853296	0.00234	replicated loci of BDI
91	19	rs7257875	19372150	19350103	19704915	0.000199	replicated loci of BDI
92	20	rs34494381	43691230	43598154	43944323	0.000415	replicated loci of BDI
93	20	rs237475	48049506	48030391	48113300	1.94E-05	replicated loci of BDI
94	20	rs2274938	60857034	60804539	60885142	0.00904	additional loci
95	21	rs2836171	39526409	39451403	39664976	0.00489	additional loci
96	22	rs9606265	20137047	20046042	20169422	0.00356	additional loci

Abbreviations: CHR, Chromosome; BP, genomic position in human genome assembly; condFDR, conditional false discovery rate.

Supplementary Table 16. Distinct genomic loci associated with major depressive disorder at condFDR<0.01 given association with irritability

Locusnum	CHR	Lead SNP	Lead BP	Mininum BP	Maximum BP	condFDR	Status
1	1	rs11577530	8743444	8520487	8895970	0.000332349	replicated loci of MDD(2021)
2	1	rs4141983	18122009	18122009	18131916	0.002335553	replicated loci of MDD
3	1	rs507288	37203702	37196958	37212710	8.91466E-05	replicated loci of MDD(2021)
4	1	rs11211481	47694167	47659445	47708112	0.002307252	additional loci
5	1	rs4926828	50218323	49355640	50589742	4.74161E-06	replicated loci of MDD
6	1	rs1890946	52342427	52266242	52406432	3.29455E-05	replicated loci of MDD
7	1	rs6656912	67083671	66998036	67221552	0.000234729	replicated loci of MDD
8	1	rs7531118	72837239	72748669	72838406	7.34223E-07	replicated loci of MDD
9	1	rs12136984	73725998	73307570	73991792	7.92011E-07	replicated loci of MDD
10	1	rs1730865	107605611	107537916	107627697	0.007089106	additional loci
11	1	rs10913112	175913828	175902660	176406835	0.002819247	replicated loci of MDD
12	1	rs7548487	177025098	177017173	177041419	0.007809303	replicated loci of MDD(2021)
13	1	rs10913341	177402667	177316726	177428790	0.000440645	replicated loci of MDD(2021)
14	1	rs113198479	191347803	191203487	191420181	0.003324906	additional loci
15	1	rs2488401	197702401	197342380	197868281	0.000236344	replicated loci of MDD
16	1	rs12125521	214442481	214413099	214442481	0.008215659	additional loci
17	1	rs6674701	227007182	227000011	227214745	0.000840002	additional loci
18	1	rs116072968	232371512	232340219	232388034	0.003291656	additional loci
19	2	rs13409834	22173664	22045717	22174521	0.001759062	replicated loci of MDD(2021)
20	2	rs12619197	22566548	22479075	22583435	0.001342231	replicated loci of MDD(2021)
21	2	rs151204187	31545596	31538086	31545596	0.007954777	additional loci
22	2	rs62142905	51554749	51554749	51627112	0.00867488	additional loci
23	2	rs1568452	58012833	57813173	58237405	0.00051595	replicated loci of MDD(2021)
24	2	rs56211578	86796962	86570906	86858047	0.001626765	additional loci
25	2	rs2582954	104452335	104452335	104452335	0.000941428	additional loci
26	2	rs4662298	143554417	143331371	143577030	0.00776135	additional loci
27	2	rs13008797	146217925	146122776	146309983	0.00874978	additional loci
28	2	rs4665145	160907869	160899045	160907869	0.00772904	additional loci
29	2	rs4131815	161531306	161440199	161535181	0.004993714	additional loci

30	2	rs6433563	176717873	176717741	176727834	0.003505422	additional loci
31	2	rs2111592	208049581	208017033	208088987	0.005324004	replicated loci of MDD
32	2	rs55897719	212590841	212590505	212636271	0.000866075	replicated loci of MDD
33	2	rs1357139	213050781	213050781	213073599	0.002474621	replicated loci of MDD
34	3	rs7631372	9493315	9403161	9543642	0.001123604	additional loci
35	3	rs56856481	29625970	29625970	29689912	0.003563365	additional loci
36	3	rs112844822	38415478	38415478	38415478	0.008703175	additional loci
37	3	rs9831648	49214303	49109919	49616053	2.19839E-05	replicated loci of MDD
38	3	rs9839427	50301584	50301584	50301584	0.00937939	additional loci
39	3	rs7635184	56226041	56163560	56427303	0.006250217	additional loci
40	3	rs9816087	61144081	61126181	61182765	0.003642239	replicated loci of MDD
41	3	rs6796247	65443260	65440410	65516835	0.007748809	additional loci
42	3	rs4855940	117008483	116954150	117009130	0.003113801	replicated loci of MDD
43	3	rs66511648	117515519	117484931	117822025	0.000112042	replicated loci of MDD
44	3	rs12631196	158171455	157830916	158284681	0.009170513	replicated loci of MDD
45	4	rs3905238	3078937	3074795	3254767	0.00284808	additional loci
46	4	rs9990752	15649079	15538922	15649079	0.000989439	additional loci
47	4	rs12511027	38370580	38324024	38371812	0.00113358	additional loci
48	4	rs10022087	42144966	42106538	42184374	0.007144356	additional loci
49	4	rs11131202	59903212	59864305	59968377	0.001735483	additional loci
50	4	rs35553410	131237381	131134864	131239286	0.001296926	additional loci
51	4	rs1116690	143510148	143486962	143622796	0.004633318	additional loci
52	5	rs448809	88005828	87514778	88102281	0.000290254	replicated loci of MDD
53	5	rs3843487	92392021	92363455	92573277	0.000796385	replicated loci of MDD(2021)
54	5	rs6556833	93104707	92995013	93494731	0.000730563	replicated loci of MDD(2021)
55	5	rs2431108	103947968	103783801	104082179	3.64567E-08	replicated loci of MDD
56	5	rs288160	107364269	107006644	107684324	0.002387485	additional loci
57	5	rs10434704	164488849	164467717	164678946	2.88671E-05	replicated loci of MDD
58	5	rs73352630	166065998	166040547	166065998	0.005689783	additional loci
59	6	rs3846828	24282117	24193869	24316404	0.009989794	additional loci
60	6	rs17720293	28214698	27249686	29211556	2.45155E-06	replicated loci of MDD
61	6	rs6925748	50930041	50930041	50930041	0.005413297	additional loci

62	6	rs68170059	66534041	66524413	66560397	0.004297368	replicated loci of MDD
63	6	rs2214123	67000001	66870010	67000001	0.000903276	replicated loci of MDD
64	6	rs4707630	91321667	91312025	91359756	0.002546434	additional loci
65	6	rs12196783	101028721	100909398	101273149	0.000735051	additional loci
66	6	rs12526217	130757720	130543687	130770858	9.98087E-05	additional loci
67	6	rs1848750	142987461	142868502	143011771	2.03152E-05	replicated loci of MDD
68	6	rs4895718	147973296	147946018	147987135	0.000327518	additional loci
69	6	rs9347903	165114404	165063209	165178503	0.000732876	replicated loci of MDD
70	7	rs10235664	2086814	1911281	2246454	4.38538E-05	replicated loci of MDD
71	7	rs12537408	2662818	2657996	2669863	0.002844142	replicated loci of MDD
72	7	rs3807866	12250378	12233848	12286050	3.64567E-08	replicated loci of MDD
73	7	rs59082935	38724868	38724868	38724868	0.002402039	replicated loci of MDD
74	7	rs1044639	82451558	82388714	82555669	0.001132659	replicated loci of MDD
75	7	rs2396133	109197067	109099919	109229384	0.000144707	replicated loci of MDD
76	7	rs2894699	114059156	114015707	114194615	0.000122721	additional loci
77	7	rs6968380	114940159	114940159	115025708	0.006418965	additional loci
78	7	rs10242758	117522956	117497811	117593308	0.000116916	replicated loci of MDD
79	7	rs3824009	126647487	126647487	126785804	0.007538574	additional loci
80	7	rs9800952	140669703	140665521	140791820	0.003526729	additional loci
81	8	rs11250015	10307328	10288480	10327625	8.05178E-05	additional loci
82	8	rs1016165	31824555	31824555	31947234	0.001185269	additional loci
83	8	rs11777872	64525347	64496159	64624581	0.002248517	additional loci
84	8	rs7837935	65562019	65500967	65745896	0.000531512	additional loci
85	8	rs2607104	92563922	92464180	92768217	0.008271208	additional loci
86	8	rs4735241	94845898	94703482	94872147	0.002548641	additional loci
87	8	rs62519760	114119452	113923381	114388945	0.000401939	additional loci
88	8	rs113103755	114977271	114977271	114977271	0.007481714	additional loci
89	8	rs7842361	118908192	118884379	119020964	0.000708951	additional loci
90	8	rs7009139	129914248	129914248	129992048	0.009166523	additional loci
91	8	rs876575	131210851	131084728	131361477	0.009119358	additional loci
92	9	rs1931388	11203149	11141257	11771159	1.43987E-05	replicated loci of MDD
93	9	rs413550	17025503	17004247	17110944	0.000316285	replicated loci of MDD

94	9	rs3793577	23737627	23736400	23737627	0.007179398	replicated loci of MDD(2021)
95	9	rs59283172	25232978	25049055	25273673	0.007317811	replicated loci of MDD
96	9	rs62535714	37182655	36999369	37406391	1.01791E-05	replicated loci of MDD
97	9	rs11137815	81354004	81331339	81354365	0.00564265	additional loci
98	9	rs111655660	96234468	96174764	96337423	0.004467094	additional loci
99	9	rs10512249	98256309	98191712	98314306	0.004056888	additional loci
100	9	rs2418449	119731359	119699096	119766782	0.000285791	replicated loci of MDD
101	9	rs72616623	120507371	120488996	120534666	0.000873307	replicated loci of MDD
102	9	rs10760393	127892193	127792230	128003979	0.009605511	additional loci
103	10	rs997934	1795194	1772590	1796472	0.008754331	additional loci
104	10	rs7083756	10879075	10840162	10910560	0.002763966	additional loci
105	10	rs4948437	62733317	62676681	62753643	0.007561916	additional loci
106	10	rs2394259	68400027	68395291	68575278	0.000346982	additional loci
107	10	rs10748841	104996309	104965551	105175131	0.009795535	additional loci
108	10	rs1021363	106610839	106469961	106812116	5.0048E-05	replicated loci of MDD
109	11	rs7118807	28664779	28591168	28709434	7.19702E-05	additional loci
110	11	rs1002728	29774810	29680576	29788700	0.007135255	additional loci
111	11	rs76416526	48239731	47925962	49236977	0.007908492	additional loci
112	11	rs2509805	57650796	57404779	57681828	0.000131992	additional loci
113	11	rs198457	61471678	61448384	61525020	3.36497E-05	replicated loci of MDD
114	11	rs2846139	65309983	65217626	65343399	0.000552028	additional loci
115	11	rs4497414	88756779	88469683	88798481	2.82E-07	replicated loci of MDD
116	11	rs578174	89959637	89959637	89997524	0.001267558	additional loci
117	11	rs10790534	99077031	99038365	99087306	0.008976422	additional loci
118	11	rs4936275	113365084	112827048	113413565	1.81761E-06	replicated loci of MDD
119	11	rs612823	133834104	133834104	133834104	0.003546093	additional loci
120	12	rs4337088	22881333	22872641	22890730	0.001095106	additional loci
121	12	rs11612312	52349088	52321150	52395834	0.001568332	replicated loci of MDD
122	12	rs7313797	109896165	109849410	110042348	0.009879327	additional loci
123	12	rs56080343	118876918	118580315	118888131	0.000525503	additional loci
124	13	rs9529218	31790053	31755898	31838516	0.000395932	replicated loci of MDD
125	13	rs1867370	53898419	53823794	54049489	9.99733E-05	replicated loci of MDD

126	13	rs1925060	55616633	55603644	55638510	0.006499998	additional loci
127	13	rs9564291	66501198	66500825	66501198	0.001509108	additional loci
128	13	rs4884833	69721057	69652435	69753336	0.007657261	additional loci
129	13	rs508502	80921519	80917527	80930178	0.006092912	replicated loci of MDD
130	13	rs7318798	97465425	97465425	97505413	0.002194735	additional loci
131	13	rs4772087	99115041	99090837	99241507	0.00143057	additional loci
132	14	rs4981197	34000745	33995060	34013948	0.007771556	additional loci
133	14	rs7149503	41568327	41515811	41569685	0.009549649	replicated loci of MDD
134	14	rs1950829	42097937	41969803	42183025	2.42989E-05	replicated loci of MDD
135	14	rs2933195	47293348	47239743	47416511	0.003811874	additional loci
136	14	rs7152906	75125540	75108290	75373011	5.61354E-06	replicated loci of MDD
137	14	rs8013071	98447604	98396052	98501877	0.005558235	additional loci
138	14	rs911554	103999371	103852607	104014935	9.98474E-07	replicated loci of MDD
139	15	rs779	29856238	29848687	29858805	0.001038693	additional loci
140	15	rs12912299	38897857	38856493	38926835	0.008650911	additional loci
141	15	rs72743363	74104811	74095709	74186934	0.002146305	additional loci
142	15	rs4362360	86940622	86871047	87011615	0.008527805	additional loci
143	15	rs8035777	88943807	88933098	88963298	0.004914298	replicated loci of MDD
144	15	rs17514846	91416550	91412850	91429042	0.009078797	additional loci
145	15	rs12438687	96955776	96955776	96957969	0.002233003	additional loci
146	16	rs10852673	6324967	6266886	6324967	0.001114395	additional loci
147	16	rs9925434	7666407	7657673	7673819	0.003281999	additional loci
148	16	rs7200826	13066833	13021889	13118299	0.002423336	replicated loci of MDD
149	16	rs35951344	13774412	13713926	13805809	2.86367E-05	replicated loci of MDD
150	16	rs12926255	17851620	17851620	17851620	0.008595072	additional loci
151	16	rs2369818	21614009	21595126	21712743	0.006085011	replicated loci of MDD(2021)
152	16	rs1862743	60743834	60583974	60743834	0.00145662	additional loci
153	18	rs58250641	28018056	27827170	28020172	0.005408161	additional loci
154	18	rs10460051	31413679	31303557	31415929	0.002084716	additional loci
155	18	rs11665070	35152563	35127427	35204858	4.87013E-06	replicated loci of MDD
156	18	rs4267411	39305256	39253947	39322811	0.002712945	additional loci
157	18	rs4632195	50746748	50389194	50907127	1.4438E-05	replicated loci of MDD

158	18	rs2924329	53135894	51764473	53725929	3.64567E-08	replicated loci of MDD
159	18	rs3760569	60239081	60223017	60262305	0.003891175	additional loci
160	18	rs7241572	77580712	77551586	77580712	0.000374836	replicated loci of MDD
161	20	rs12624433	44680853	44673546	44747947	1.24259E-05	replicated loci of MDD
162	22	rs12627769	20031102	20025951	20054208	0.000752753	additional loci
163	22	rs71327107	41418229	41408754	41713111	0.000964613	additional loci
164	22	rs12160976	46438246	46411969	46446563	0.000556943	replicated loci of MDD(2021)

Abbreviations: CHR, Chromosome; BP, genomic position in human genome assembly; condFDR, conditional false discovery rate

* MDD(2021) indicates Giannakopoulou, Olga, *et al.*, 2021

Supplementary Table 17. eQTL mapping for additional genomic loci from the condFDR results for psychiatric disorders conditional on irritability

a) Schizophrenia

IndSigSNPs	ensg	symbol	chr	start	end	strand	type	entrezID	HUGO	pLI	ncRVIS	eqtlMapSNPs	eqtlMapminP	eqtlMapminQ	eqtlMapts
rs79598313	ENSG00000090273	*NUDC*	1	27226729	27273353	1	protein_coding	10726	NUDC	0.822775133	0.218790394	5	2.76E-07	1.70E-08	GTEx/v8/Brain_Cortex:GTEx/v8/Brain_Nucleus_accumbens_basal_ganglia
rs79598313	ENSG00000142765	*SYTL1*	1	27668513	27680421	1	protein_coding	84958	SYTL1	1.08E-07	0.055031121	7	2.94E-07	1.48E-08	GTEx/v8/Brain_Cerebellar_Hemisphere:GTEx/v8/Brain_Cerebellum
rs79598313	ENSG00000174950	*CD164L2*	1	27705666	27709870	-1	protein_coding	388611	CD164L2	0.033744866	-0.467583072	3	1.60E-05	0.000165709	GTEx/v8/Brain_Hippocampus
rs79598313	ENSG00000126709	*IFI6*	1	27992572	27998729	-1	protein_coding	2537	IFI6	0.230931225	0.591640949	3	2.83E-05	0.00407134	GTEx/v8/Brain_Cerebellum:GTEx/v8/Brain_Hippocampus
rs1211184	ENSG00000116819	*TFAP2E*	1	36038971	36060929	1	protein_coding	339488	TFAP2E	0.000972259	-0.148652974	16	4.71E-05	0.0216732	GTEx/v8/Brain_Cerebellum
rs17785382	ENSG00000162384	*C1orf123*	1	53679771	53686289	-1	protein_coding	54987	C1orf123	1.26E-07	-0.124832619	63	2.70E-24	3.80E-19	GTEx/v8/Brain_Amygdala:GTEx/v8/Brain_Anterior_cingulate_cortex_BA24:GTEx/v8/Brain_Caudate_basal_ganglia:GTEx/v8/Brain_Cerebellar_Hemisphere:GTEx/v8/Brain_Cerebellum:GTEx/v8/Brain_Cortex:GTEx/v8/Brain_Frontal_Cortex_BA9:GTEx/v8/Brain_Hippocampus:GTEx/v8/Brain_Hypothalamus:GTEx/v8/Brain_Nucleus_accumbens_basal_ganglia:GTEx/v8/Brain_Putamen_basal_ganglia:GTEx/v8/Brain_Spinal_cord_cervical_c-1
rs17785382	ENSG00000157193	*LRP8*	1	53711217	53793742	-1	protein_coding	7804	LRP8	0.99998576	-0.679008322	41	1.93E-10	1.68E-06	GTEx/v8/Brain_Cerebellar_Hemisphere:GTEx/v8/Brain_Cerebellum:GTEx/v8/Brain_Cortex
rs3008527	ENSG00000231437	*RP11-88H9.2*	1	112532392	112541464	1	lincRNA	643355	LINC01750	NA	NA	4	3.43E-06	6.95E-21	GTEx/v8/Brain_Nucleus_accumbens_basal_ganglia
rs3008527	ENSG00000155367	*PPM1J*	1	113252616	113258099	-1	protein_coding	333926	PPM1J	2.31E-07	NA	3	3.22E-05	0.0443881	GTEx/v8/Brain_Caudate_basal_ganglia
rs869506	ENSG00000143603	*KCNN3*	1	154669931	154842756	-1	protein_coding	3782	KCNN3	0.864940881	-0.629504502	84	3.52E-07	0.00153507	GTEx/v8/Brain_Anterior_cingulate_cortex_BA24:GTEx/v8/Brain_Cerebellar_Hemisphere:GTEx/v8/Brain_Cerebellum:GTEx/v8/Brain_Cortex:GTEx/v8/Brain_Frontal_Cortex_BA9:GTEx/v8/Brain_Hippocampus:GTEx/v8/Brain_Hypothalamus:GTEx/v8/Brain_Nucleus_accumbens_basal_ganglia:GTEx/v8/Brain_Spinal_cord_cervical_c-1
rs869506	ENSG00000163344	*PMVK*	1	154897210	154909467	-1	protein_coding	10654	PMVK	0.000305455	-0.527689982	84	1.02E-13	3.03E-09	GTEx/v8/Brain_Caudate_basal_ganglia:GTEx/v8/Brain_Nucleus_accumbens_basal_ganglia
rs869506	ENSG00000163354	*DCST2*	1	154990996	155006257	-1	protein_coding	127579	DCST2	1.46E-09	0.250200946	84	1.74E-12	3.16E-21	GTEx/v8/Brain_Caudate_basal_ganglia:GTEx/v8/Brain_Cerebellar_Hemisphere:GTEx/v8/Brain_Cerebellum:GTEx/v8/Brain_Cortex:GTEx/v8/Brain_Frontal_Cortex_BA9:GTEx/v8/Brain_Nucleus_accumbens_basal_ganglia:GTEx/v8/Brain_Putamen_basal_ganglia
rs869506	ENSG00000163357	*DCST1*	1	155006300	155023406	1	protein_coding	149095	DCST1	3.89E-15	NA	68	3.51E-06	1.73E-06	GTEx/v8/Brain_Cerebellum
rs869506	ENSG00000231064	*RP11-263K19.4*	1	155166659	155175286	1	antisense	NA	NA	NA	NA	2	1.21E-05	0.0299705	GTEx/v8/Brain_Anterior_cingulate_cortex_BA24
rs869506	ENSG00000225855	*RUSC1-AS1*	1	155286654	155293967	-1	antisense	284618	RUSC1-AS1	NA	NA	7	2.10E-05	0.03082	GTEx/v8/Brain_Hippocampus
rs56407270	ENSG00000152382	*TADA1*	1	166825747	166845564	-1	protein_coding	117143	TADA1	0.358858663	1.021892531	3	3.03E-06	0.00363176	GTEx/v8/Brain_Caudate_basal_ganglia:GTEx/v8/Brain_Cerebellum:GTEx/v8/Brain_Frontal_Cortex_BA9
rs56407270	ENSG00000143194	*MAEL*	1	166958346	166991451	1	protein_coding	84944	MAEL	0.021254638	-0.753209225	16	2.58E-11	2.06E-25	GTEx/v8/Brain_Amygdala:GTEx/v8/Brain_Anterior_cingulate_cortex_BA24:GTEx/v8/Brain_Caudate_basal_ganglia:GTEx/v8/Brain_Cortex:GTEx/v8/Brain_Frontal_Cortex_BA9:GTEx/v8/Brain_Hippocampus:GTEx/v8/Brain_Hypothalamus:GTEx/v8/Brain_Nucleus_accumbens_basal_ganglia:GTEx/v8/Brain_Putamen_basal_ganglia:GTEx/v8/Brain_Substantia_nigra
rs11807834	ENSG00000143641	*GALNT2*	1	230193536	230417870	1	protein_coding	2590	GALNT2	0.881985613	4.223663186	1	1.70E-19	1.89E-14	GTEx/v8/Brain_Cerebellar_Hemisphere:GTEx/v8/Brain_Cerebellum
rs11807834	ENSG00000173409	*ARV1*	1	231114727	231136341	1	protein_coding	64801	ARV1	1.55E-06	-0.359624818	1	4.41E-06	2.43E-08	GTEx/v8/Brain_Cortex
rs2471403	ENSG00000170802	*FOXN2*	2	48541776	48606433	1	protein_coding	3344	FOXN2	0.392770621	-1.687830929	50	2.06E-10	5.46E-52	SEPTIN10
rs2471403	ENSG00000272663	*RP11-191L17.1*	2	48667182	48667736	-1	lincRNA	NA	NA	NA	NA	39	7.63E-07	8.38E-08	GTEx/v8/Brain_Cortex:GTEx/v8/Brain_Putamen_basal_ganglia
rs2471403	ENSG00000162869	*PPP1R21*	2	48667737	48742525	1	protein_coding	129285	PPP1R21	0.0004021	0.571659952	27	8.23E-06	0.000147558	GTEx/v8/Brain_Spinal_cord_cervical_c-1
rs2471403	ENSG00000243244	*STON1*	2	48756522	48826025	1	protein_coding	11037	STON1	4.45E-08	1.251076578	51	1.74E-07	1.37E-09	GTEx/v8/Brain_Anterior_cingulate_cortex_BA24:GTEx/v8/Brain_Cortex:GTEx/v8/Brain_Putamen_basal_ganglia
rs2471403	ENSG00000068781	*STON1-GTF2A1L*	2	48757064	49003654	1	protein_coding	286749	STON1-GTF2A1L	1.65E-14	0.572863521	1	0.000107562	4.03E-15	GTEx/v8/Brain_Cerebellum

rs2471403	ENSG00000242441	*GTF2A1L*	2	48844937	48960287	1	protein_coding	11036	GTF2A1L	5.65E-07	0.420928839	5	2.89E-05	4.48E-33	GTEx/v8/Brain_Cerebellum
rs148661029	ENSG00000178021	*TSPYL6*	2	54480315	54483409	-1	protein_coding	388951	TSPYL6	0.581660026	0.521740516	2	1.00E-05	0.00652768	GTEx/v8/Brain_Hypothalamus
rs9330316	ENSG00000186522	*SEPTIN10*	2	110300559	110371783	-1	protein_coding	151011	SEPTIN10	0.00283286	NA	68	4.80E-12	9.13E-08	GTEx/v8/Brain_Caudate_basal_ganglia:GTEx/v8/Brain_Hippocampus:GTEx/v8/Brain_Hypothalamus:GTEx/v8/Brain_Putamen_basal_ganglia
rs6430491	ENSG00000152127	*MGAT5*	2	134877554	135212192	1	protein_coding	4249	MGAT5	0.999925017	2.188752768	10	2.86E-06	0.00371356	GTEx/v8/Brain_Cerebellum
rs4131583	ENSG00000138430	*OLA1*	2	174937175	175113426	-1	protein_coding	29789	OLA1	0.0436207	0.562367562	5	1.82E-05	2.27E-05	GTEx/v8/Brain_Cortex
rs9310732	ENSG00000197885	*NKIRAS1*	3	23933151	23988082	-1	protein_coding	28512	NKIRAS1	0.034887772	-0.466124291	1	1.08E-05	0.00670476	GTEx/v8/Brain_Caudate_basal_ganglia
rs9310732	ENSG00000224822	*THRB-IT1*	3	24496627	24500925	-1	sense_intronic	100874274	THRB-IT1	NA	NA	5	3.96E-06	0.0323564	GTEx/v8/Brain_Amygdala
rs836927	ENSG00000273125	*RP11-115H18.1*	3	107149777	107182759	1	lincRNA	101929607	LINC01990	NA	NA	6	2.14E-11	2.40E-13	GTEx/v8/Brain_Caudate_basal_ganglia:GTEx/v8/Brain_Nucleus_accumbens_basal_ganglia:GTEx/v8/Brain_Putamen_basal_ganglia
rs270170	ENSG00000120306	*CYSTM1*	5	139554227	139661637	1	protein_coding	84418	CYSTM1	0.28430926	-0.813371493	34	1.24E-05	1.16E-06	GTEx/v8/Brain_Caudate_basal_ganglia
rs270170	ENSG00000250069	*CTB-131B5.2*	5	139579748	139582772	1	sense_intronic	NA	NA	NA	NA	53	5.22E-09	4.97E-07	GTEx/v8/Brain_Cerebellar_Hemisphere:GTEx/v8/Brain_Cerebellum
rs270170	ENSG00000213523	*SRA1*	5	139916925	139937895	-1	protein_coding	10011	SRA1	5.00E-06	-0.871642582	35	1.69E-06	5.38E-08	GTEx/v8/Brain_Hypothalamus:GTEx/v8/Brain_Substantia_nigra
rs113113059	ENSG00000112659	*CUL9*	6	43149913	43192325	1	protein_coding	23113	CUL9	0.967475855	-0.195719526	7	3.72E-15	1.67E-14	GTEx/v8/Brain_Caudate_basal_ganglia:GTEx/v8/Brain_Cortex:GTEx/v8/Brain_Frontal_Cortex_BA9:GTEx/v8/Brain_Nucleus_accumbens_basal_ganglia:GTEx/v8/Brain_Putamen_basal_ganglia
rs1849527	ENSG00000164483	*SAMD3*	6	130465460	130686570	-1	protein_coding	154075	SAMD3	1.10E-06	0.088198284	242	8.80E-07	0.00198934	GTEx/v8/Brain_Cortex
rs12672629	ENSG00000105866	*SP4*	7	21467652	21554440	1	protein_coding	6671	SP4	0.951288043	0.671148604	12	5.63E-06	0.0233599	GTEx/v8/Brain_Nucleus_accumbens_basal_ganglia
rs12672629	ENSG00000105877	*DNAH11*	7	21582833	21941457	1	protein_coding	8701	DNAH11	NA	0.456635771	20	3.56E-07	2.36E-08	GTEx/v8/Brain_Caudate_basal_ganglia:GTEx/v8/Brain_Cortex:GTEx/v8/Brain_Frontal_Cortex_BA9:GTEx/v8/Brain_Putamen_basal_ganglia
rs492430	ENSG00000066923	*STAG3*	7	99775186	99819111	1	protein_coding	10734	STAG3	1.10E-06	-0.496377292	1	6.40E-05	7.08E-11	GTEx/v8/Brain_Putamen_basal_ganglia
rs492430	ENSG00000213413	*PVRIG*	7	99815864	99819113	1	protein_coding	79037	PVRIG	0.001001892	0.083525111	12	5.27E-06	1.08E-12	GTEx/v8/Brain_Caudate_basal_ganglia
rs492430	ENSG00000106327	*TFR2*	7	100218039	100240402	-1	protein_coding	7036	TFR2	0.000139031	-0.558362645	18	7.99E-08	0.000452686	GTEx/v8/Brain_Nucleus_accumbens_basal_ganglia
rs492430	ENSG00000146830	*GIGYF1*	7	100277130	100287071	-1	protein_coding	64599	GIGYF1	0.978704812	1.432151846	18	2.42E-14	1.20E-09	GTEx/v8/Brain_Caudate_basal_ganglia:GTEx/v8/Brain_Cerebellum:GTEx/v8/Brain_Cortex:GTEx/v8/Brain_Frontal_Cortex_BA9:GTEx/v8/Brain_Hippocampus:GTEx/v8/Brain_Hypothalamus:GTEx/v8/Brain_Nucleus_accumbens_basal_ganglia:GTEx/v8/Brain_Putamen_basal_ganglia:GTEx/v8/Brain_Spinal_cord_cervical_c-1:GTEx/v8/Brain_Substantia_nigra
rs492430	ENSG00000130427	*EPO*	7	100318423	100321323	1	protein_coding	2056	EPO	0.116862382	NA	1	3.23E-08	2.83E-06	GTEx/v8/Brain_Cerebellar_Hemisphere:GTEx/v8/Brain_Cerebellum
rs6662	ENSG00000090263	*MRPS33*	7	140705854	140715028	-1	protein_coding	51650	MRPS33	0.003395718	0.268392925	42	2.30E-09	1.80E-05	GTEx/v8/Brain_Caudate_basal_ganglia:GTEx/v8/Brain_Cortex:GTEx/v8/Brain_Putamen_basal_ganglia
rs1583830	ENSG00000214106	*PAXIP1-AS2*	7	154720208	154741618	1	protein_coding	100132707	PAXIP1-AS2	0.09168088	NA	80	3.19E-12	1.94E-12	GTEx/v8/Brain_Amygdala:GTEx/v8/Brain_Anterior_cingulate_cortex_BA24:GTEx/v8/Brain_Caudate_basal_ganglia:GTEx/v8/Brain_Cerebellum:GTEx/v8/Brain_Cortex:GTEx/v8/Brain_Nucleus_accumbens_basal_ganglia:GTEx/v8/Brain_Putamen_basal_ganglia
rs1583830	ENSG00000157219	*HTR5A*	7	154862034	154877459	1	protein_coding	3361	HTR5A	0.005788634	2.172405177	1	1.06E-05	0.04112	GTEx/v8/Brain_Hypothalamus
rs7002619	ENSG00000038945	*MSR1*	8	15965387	16424999	-1	protein_coding	4481	MSR1	8.21E-16	1.819933539	46	7.05E-07	0.00568105	GTEx/v8/Brain_Caudate_basal_ganglia:GTEx/v8/Brain_Hypothalamus:GTEx/v8/Brain_Nucleus_accumbens_basal_ganglia
rs1043083	ENSG00000198791	*CNOT7*	8	17086737	17104387	-1	protein_coding	29883	CNOT7	0.986495484	-0.616746759	4	1.51E-05	3.25E-22	GTEx/v8/Brain_Cerebellar_Hemisphere:GTEx/v8/Brain_Cerebellum:GTEx/v8/Brain_Frontal_Cortex_BA9:GTEx/v8/Brain_Hypothalamus
rs12674620	ENSG00000168300	*PCMTD1*	8	52730140	52811735	-1	protein_coding	115294	PCMTD1	2.56E-06	0.758161742	25	8.46E-08	1.24E-22	GTEx/v8/Brain_Cerebellar_Hemisphere:GTEx/v8/Brain_Cerebellum
rs717349	ENSG00000164758	*MED30*	8	118532952	118552501	1	protein_coding	90390	MED30	0.60654151	0.245003676	29	3.61E-09	2.69E-05	GTEx/v8/Brain_Caudate_basal_ganglia:GTEx/v8/Brain_Cortex:GTEx/v8/Brain_Frontal_Cortex_BA9:GTEx/v8/Brain_Putamen_basal_ganglia
rs4733770	ENSG00000153317	*ASAP1*	8	131064353	131455906	-1	protein_coding	50807	ASAP1	0.999983091	0.592920078	25	1.01E-08	1.01E-16	GTEx/v8/Brain_Cerebellar_Hemisphere:GTEx/v8/Brain_Cerebellum:GTEx/v8/Brain_Cortex:GTEx/v8/Brain_Nucleus_accumbens_basal_ganglia:GTEx/v8/Brain_Putamen_basal_ganglia
rs7829793	ENSG00000180921	*FAM83H*	8	144806103	144815971	-1	protein_coding	286077	FAM83H	0.88482471	NA	70	9.04E-15	8.51E-24	GTEx/v8/Brain_Amygdala:GTEx/v8/Brain_Cerebellar_Hemisphere:GTEx/v8/Brain_Cerebellum:GTEx/v8/Brain_Cortex:GTEx/v8/Brain_Frontal_Cortex_BA9
rs7829793	ENSG00000203499	*FAM83H-AS1*	8	144816310	144828507	1	lincRNA	100128338	FAM83H-AS1	NA	NA	70	1.58E-23	2.20E-18	GTEx/v8/Brain_Cerebellar_Hemisphere:GTEx/v8/Brain_Cerebellum:GTEx/v8/Brain_Cortex

rs7829793	ENSG00000254973	*RP11-429J17.7*	8	14484032 3	144853992	-1	lincRNA	105375800	LOC105375800	NA	NA	70	5.10E-17	2.05E-12	GTEx/v8/Brain_Cerebellar_Hemisphere:GTEx/v8/Brain_Cerebellum
rs7829793	ENSG00000180900	*SCRIB*	8	144873090	144897549	-1	protein_coding	23513	SCRIB	0.913273181	NA	31	3.53E-06	0.0097245	GTEx/v8/Brain_Cerebellum
rs10967586	ENSG00000120159	*CAAP1*	9	26840683	26892802	-1	protein_coding	79886	CAAP1	0.708931875	-0.482711693	25	1.20E-06	0.00480448	GTEx/v8/Brain_Cerebellum
rs10967586	ENSG00000096872	*IFT74*	9	26947037	27062928	1	protein_coding	80173	IFT74	9.27E-06	-0.450404011	2	3.41E-05	5.98E-16	GTEx/v8/Brain_Cerebellum:GTEx/v8/Brain_Cortex
rs12554874	ENSG00000147905	*ZCCHC7*	9	37120536	37358146	1	protein_coding	84186	ZCCHC7	3.37E-09	0.160585505	11	8.60E-06	0.0328071	GTEx/v8/Brain_Hippocampus
rs7927922	ENSG00000110697	*PITPNM1*	11	67259239	67273734	-1	protein_coding	9600	PITPNM1	0.722090614	NA	12	2.82E-05	0.0454835	GTEx/v8/Brain_Nucleus_accumbens_basal_ganglia
rs3214023	ENSG00000135476	*ESPL1*	12	53662083	53687427	1	protein_coding	9700	ESPL1	0.999999918	0.276246018	10	5.17E-10	2.21E-18	GTEx/v8/Brain_Caudate_basal_ganglia:GTEx/v8/Brain_Hypothalamus:GTEx/v8/Brain_Nucleus_accumbens_basal_ganglia:GTEx/v8/Brain_Putamen_basal_ganglia
rs3214023	ENSG00000094914	*AAAS*	12	53701240	53718648	-1	protein_coding	8086	AAAS	4.54E-10	-0.354865458	3	3.49E-05	0.00211102	GTEx/v8/Brain_Cortex
rs3214023	ENSG00000205352	*PRR13*	12	53835389	53840429	1	protein_coding	54458	PRR13	0.634332002	-0.290702359	1	2.86E-05	5.66E-07	GTEx/v8/Brain_Spinal_cord_cervical_c-1
rs55968332	ENSG00000088448	*ANKRD10*	13	111530887	111567416	-1	protein_coding	55608	ANKRD10	0.127073152	0.202032918	3	1.51E-07	8.95E-11	GTEx/v8/Brain_Cortex
rs6560936	ENSG00000185989	*RASA3*	13	114747194	114898086	-1	protein_coding	22821	RASA3	0.000433959	0.061217245	48	3.05E-10	2.59E-08	GTEx/v8/Brain_Cerebellar_Hemisphere:GTEx/v8/Brain_Cerebellum:GTEx/v8/Brain_Cortex
rs6560936	ENSG00000130177	*CDC16*	13	115000362	115038198	1	protein_coding	8881	CDC16	0.95225535	NA	9	9.23E-06	2.37E-11	GTEx/v8/Brain_Cerebellum
rs6560936	ENSG00000169062	*UPF3A*	13	115047059	115071283	1	protein_coding	65110	UPF3A	0.000813153	0.189120204	3	3.36E-05	0.00632297	GTEx/v8/Brain_Spinal_cord_cervical_c-1
rs11854184	ENSG00000259531	*RP11-295H24.3*	15	49657321	49658882	-1	pseudogene	NA	NA	NA	NA	3	2.71E-05	0.0307377	GTEx/v8/Brain_Cerebellum
rs2270366	ENSG00000262246	*CORO7*	16	4404543	4475706	-1	protein_coding	79585	CORO7	7.31E-12	-0.351841137	146	1.95E-14	1.47E-14	GTEx/v8/Brain_Amygdala:GTEx/v8/Brain_Anterior_cingulate_cortex_BA24:GTEx/v8/Brain_Caudate_basal_ganglia:GTEx/v8/Brain_Cerebellar_Hemisphere:GTEx/v8/Brain_Cerebellum:GTEx/v8/Brain_Cortex:GTEx/v8/Brain_Frontal_Cortex_BA9:GTEx/v8/Brain_Hippocampus:GTEx/v8/Brain_Hypothalamus:GTEx/v8/Brain_Nucleus_accumbens_basal_ganglia:GTEx/v8/Brain_Putamen_basal_ganglia:GTEx/v8/Brain_Substantia_nigra
rs2270366	ENSG00000168140	*VASN*	16	4421849	4433529	1	protein_coding	114990	VASN	0.053807166	-0.455242703	82	7.48E-07	0.00117221	GTEx/v8/Brain_Cerebellum
rs2270366	ENSG00000153406	*NMRAL1*	16	4511681	4545764	-1	protein_coding	57407	NMRAL1	0.000731171	1.148650738	146	5.51E-56	6.03E-48	GTEx/v8/Brain_Amygdala:GTEx/v8/Brain_Anterior_cingulate_cortex_BA24:GTEx/v8/Brain_Caudate_basal_ganglia:GTEx/v8/Brain_Cerebellar_Hemisphere:GTEx/v8/Brain_Cerebellum:GTEx/v8/Brain_Cortex:GTEx/v8/Brain_Frontal_Cortex_BA9:GTEx/v8/Brain_Hippocampus:GTEx/v8/Brain_Hypothalamus:GTEx/v8/Brain_Nucleus_accumbens_basal_ganglia:GTEx/v8/Brain_Putamen_basal_ganglia:GTEx/v8/Brain_Spinal_cord_cervical_c-1:GTEx/v8/Brain_Substantia_nigra
rs2270366	ENSG00000103415	*HMOX2*	16	4524691	4560348	1	protein_coding	3163	HMOX2	0.001100656	-0.069465183	143	1.61E-08	8.64E-06	GTEx/v8/Brain_Cerebellum:GTEx/v8/Brain_Nucleus_accumbens_basal_ganglia
rs2270366	ENSG00000089486	*CDIP1*	16	4560676	4588829	-1	protein_coding	29965	CDIP1	0.734505012	-0.856573393	146	1.36E-51	3.08E-43	GTEx/v8/Brain_Anterior_cingulate_cortex_BA24:GTEx/v8/Brain_Caudate_basal_ganglia:GTEx/v8/Brain_Cerebellar_Hemisphere:GTEx/v8/Brain_Cerebellum:GTEx/v8/Brain_Cortex:GTEx/v8/Brain_Frontal_Cortex_BA9
rs2270366	ENSG00000102858	*MGRN1*	16	4666494	4740975	1	protein_coding	23295	MGRN1	0.450177891	1.460238482	44	8.53E-06	2.17E-63	GTEx/v8/Brain_Caudate_basal_ganglia:GTEx/v8/Brain_Cerebellum:GTEx/v8/Brain_Cortex:GTEx/v8/Brain_Frontal_Cortex_BA9:GTEx/v8/Brain_Substantia_nigra
rs62063686	ENSG00000136448	*NMT1*	17	43128978	43186384	1	protein_coding	4836	NMT1	0.998500521	0.032447683	1801	9.33E-06	2.39E-07	GTEx/v8/Brain_Cerebellar_Hemisphere:GTEx/v8/Brain_Cerebellum:GTEx/v8/Brain_Spinal_cord_cervical_c-1
rs62063686	ENSG00000267121	*CTD-2020K17.1*	17	43268298	43299589	-1	antisense	339192	LOC339192	NA	NA	2668	6.12E-11	4.50E-07	GTEx/v8/Brain_Cerebellar_Hemisphere:GTEx/v8/Brain_Cerebellum
rs62063686	ENSG00000184922	*FMNL1*	17	43298811	43324687	1	protein_coding	752	FMNL1	0.999687036	NA	2668	1.02E-24	7.89E-19	GTEx/v8/Brain_Cerebellar_Hemisphere:GTEx/v8/Brain_Cerebellum
rs62063686	ENSG00000233175	*CTD-2020K17.3*	17	43315395	43319101	-1	antisense	107985040	LOC107985040	NA	NA	21	9.72E-05	2.69E-16	GTEx/v8/Brain_Cerebellum
rs62063686	ENSG00000006062	*MAP3K14*	17	43340488	43394414	-1	processed_transcript	9020	MAP3K14	NA	NA	2	0.000176915	0.00353334	GTEx/v8/Brain_Cerebellum
rs62063686	ENSG00000159314	*ARHGAP27*	17	43471275	43511787	-1	protein_coding	201176	ARHGAP27	0.001255844	-0.761627793	2806	4.59E-19	7.23E-14	GTEx/v8/Brain_Caudate_basal_ganglia:GTEx/v8/Brain_Cerebellar_Hemisphere:GTEx/v8/Brain_Cerebellum:GTEx/v8/Brain_Cortex:GTEx/v8/Brain_Nucleus_accumbens_basal_ganglia:GTEx/v8/Brain_Putamen_basal_ganglia
rs62063686	ENSG00000225190	*PLEKHM1*	17	43513266	43568115	-1	protein_coding	9842	PLEKHM1	0.84054321	-0.663068032	2806	1.98E-42	8.80E-35	GTEx/v8/Brain_Amygdala:GTEx/v8/Brain_Anterior_cingulate_cortex_BA24:GTEx/v8/Brain_Caudate_basal_ganglia:GTEx/v8/Brain_Cerebellar_Hemisphere:GTEx/v8/Brain_Cerebellum:GTEx/v8/Brain_Cortex:GTEx/v8/Brain_Frontal_Cortex_BA9:GTEx/v8/Brain_Hypothalamus:GTEx/v8/Brain_Nucleus_accumbens_basal_ganglia:GTEx/v8/Brain_Putamen_basal_ganglia
rs62063686	ENSG00000236234	*AC091132.1*	17	43530210	43541431	1	antisense	NA	NA	NA	NA	2806	2.17E-12	2.29E-08	GTEx/v8/Brain_Cerebellar_Hemisphere:GTEx/v8/Brain_Cerebellum
rs62063686	ENSG00000214425	*LRRC37A4P*	17	43578685	43627701	-1	pseudogene	55073	LRRC37A4P	NA	NA	2808	1.81E-58	2.69E-48	GTEx/v8/Brain_Amygdala:GTEx/v8/Brain_Anterior_cingulate_cortex_BA24:GTEx/v8/Brain_Caudate_basal_ganglia:GTEx/v8/Brain_Cerebellar_Hemisphere:GTEx/v8/Brain_Cerebellum:GTEx/v8/Brain_Cortex:GTEx/v8/Brain_Frontal_Cortex_BA9:GTEx/v8/Brain_Hippocampus:GTEx/v8/Brain_Hypothalamus:GTEx/v8/Brain_Nucleus_accumbens_basal_ganglia:GTEx/v8/Brain_Putamen_basal_ganglia:GTEx/v8/Brain_Spinal_cord_cervical_c-1:GTEx/v8/Brain_Substantia_nigra

rs62063686	ENSG00000266918	*RP11-798G7.8*	17	43608943	43611204	1	lincRNA	NA	NA	NA	NA	2806	2.14E-16	6.68E-12	GTEx/v8/Brain_Amygdala:GTEx/v8/Brain_Caudate_basal_ganglia:GTEx/v8/Brain_Cerebellar_Hemisphere:GTEx/v8/Brain_Cerebellum:GTEx/v8/Brain_Cortex
rs62063686	ENSG00000267198	*RP11-798G7.6*	17	43623170	43640596	1	lincRNA	NA	NA	NA	NA	2606	5.49E-06	1.37E-08	GTEx/v8/Brain_Cerebellum
rs62063686	ENSG00000264070	*DND1P1*	17	43663237	43664295	1	pseudogene	644157	DND1P1	NA	NA	2807	7.80E-38	1.42E-31	GTEx/v8/Brain_Amygdala:GTEx/v8/Brain_Anterior_cingulate_cortex_BA24:GTEx/v8/Brain_Caudate_basal_ganglia:GTEx/v8/Brain_Cerebellar_Hemisphere:GTEx/v8/Brain_Cerebellum:GTEx/v8/Brain_Cortex:GTEx/v8/Brain_Frontal_Cortex_BA9:GTEx/v8/Brain_Hippocampus:GTEx/v8/Brain_Hypothalamus:GTEx/v8/Brain_Nucleus_accumbens_basal_ganglia:GTEx/v8/Brain_Putamen_basal_ganglia:GTEx/v8/Brain_Spinal_cord_cervical_c-1:GTEx/v8/Brain_Substantia_nigra
rs62063686	ENSG00000263503	*RP11-707O23.5*	17	43678235	43679706	-1	pseudogene	NA	NA	NA	NA	2807	1.33E-41	1.26E-35	GTEx/v8/Brain_Amygdala:GTEx/v8/Brain_Anterior_cingulate_cortex_BA24:GTEx/v8/Brain_Caudate_basal_ganglia:GTEx/v8/Brain_Cerebellar_Hemisphere:GTEx/v8/Brain_Cerebellum:GTEx/v8/Brain_Cortex:GTEx/v8/Brain_Frontal_Cortex_BA9:GTEx/v8/Brain_Hippocampus:GTEx/v8/Brain_Hypothalamus:GTEx/v8/Brain_Nucleus_accumbens_basal_ganglia:GTEx/v8/Brain_Putamen_basal_ganglia:GTEx/v8/Brain_Spinal_cord_cervical_c-1:GTEx/v8/Brain_Substantia_nigra
rs62063686	ENSG00000204650	*CRHR1-IT1*	17	43697694	43725582	1	pseudogene	147081	CRHR1-IT1	NA	NA	2808	1.06E-38	1.09E-30	GTEx/v8/Brain_Amygdala:GTEx/v8/Brain_Anterior_cingulate_cortex_BA24:GTEx/v8/Brain_Caudate_basal_ganglia:GTEx/v8/Brain_Cerebellar_Hemisphere:GTEx/v8/Brain_Cerebellum:GTEx/v8/Brain_Cortex:GTEx/v8/Brain_Frontal_Cortex_BA9:GTEx/v8/Brain_Hippocampus:GTEx/v8/Brain_Hypothalamus:GTEx/v8/Brain_Nucleus_accumbens_basal_ganglia:GTEx/v8/Brain_Putamen_basal_ganglia:GTEx/v8/Brain_Spinal_cord_cervical_c-1:GTEx/v8/Brain_Substantia_nigra
rs62063686	ENSG00000120088	*CRHR1*	17	43699267	43913194	1	protein_coding	1394	CRHR1	0.792134286	2.038085631	2819	4.29E-06	0.00117547	GTEx/v8/Brain_Caudate_basal_ganglia:GTEx/v8/Brain_Cortex:GTEx/v8/Brain_Hippocampus:GTEx/v8/Brain_Nucleus_accumbens_basal_ganglia:GTEx/v8/Brain_Putamen_basal_ganglia
rs62063686	ENSG00000264589	*MAPT-AS1*	17	43921017	43972966	-1	antisense	100128977	MAPT-AS1	NA	NA	2879	1.07E-21	3.37E-16	GTEx/v8/Brain_Caudate_basal_ganglia:GTEx/v8/Brain_Cerebellar_Hemisphere:GTEx/v8/Brain_Cerebellum:GTEx/v8/Brain_Hippocampus:GTEx/v8/Brain_Hypothalamus:GTEx/v8/Brain_Nucleus_accumbens_basal_ganglia:GTEx/v8/Brain_Putamen_basal_ganglia:GTEx/v8/Brain_Spinal_cord_cervical_c-1:GTEx/v8/Brain_Substantia_nigra
rs62063686	ENSG00000185294	*SPPL2C*	17	43922256	43924438	1	protein_coding	162540	SPPL2C	2.30E-05	0.187394842	2879	6.65E-25	5.44E-19	GTEx/v8/Brain_Cerebellar_Hemisphere:GTEx/v8/Brain_Cerebellum:GTEx/v8/Brain_Cortex:GTEx/v8/Brain_Frontal_Cortex_BA9
rs62063686	ENSG00000186868	*MAPT*	17	43971748	44105700	1	protein_coding	4137	MAPT	4.68E-05	2.01024275	2853	1.10E-07	7.42E-05	GTEx/v8/Brain_Caudate_basal_ganglia:GTEx/v8/Brain_Cerebellar_Hemisphere:GTEx/v8/Brain_Cerebellum
rs62063686	ENSG00000120071	*KANSL1*	17	44107282	44302733	-1	protein_coding	284058	KANSL1	0.999733506	0.058945245	2867	5.76E-07	1.09E-09	GTEx/v8/Brain_Caudate_basal_ganglia:GTEx/v8/Brain_Cerebellar_Hemisphere:GTEx/v8/Brain_Cerebellum:GTEx/v8/Brain_Frontal_Cortex_BA9
rs62063686	ENSG00000214401	*KANSL1-AS1*	17	44270942	44274089	1	antisense	644246	KANSL1-AS1	NA	NA	2879	4.51E-52	1.22E-43	GTEx/v8/Brain_Amygdala:GTEx/v8/Brain_Anterior_cingulate_cortex_BA24:GTEx/v8/Brain_Caudate_basal_ganglia:GTEx/v8/Brain_Cerebellar_Hemisphere:GTEx/v8/Brain_Cerebellum:GTEx/v8/Brain_Cortex:GTEx/v8/Brain_Frontal_Cortex_BA9:GTEx/v8/Brain_Hippocampus:GTEx/v8/Brain_Hypothalamus:GTEx/v8/Brain_Nucleus_accumbens_basal_ganglia:GTEx/v8/Brain_Putamen_basal_ganglia:GTEx/v8/Brain_Spinal_cord_cervical_c-1:GTEx/v8/Brain_Substantia_nigra
rs62063686	ENSG00000262500	*RP11-259G18.2*	17	44320972	44322410	1	pseudogene	NA	NA	NA	NA	2879	2.61E-40	5.94E-34	GTEx/v8/Brain_Amygdala:GTEx/v8/Brain_Anterior_cingulate_cortex_BA24:GTEx/v8/Brain_Caudate_basal_ganglia:GTEx/v8/Brain_Cerebellar_Hemisphere:GTEx/v8/Brain_Cerebellum:GTEx/v8/Brain_Cortex:GTEx/v8/Brain_Frontal_Cortex_BA9:GTEx/v8/Brain_Hippocampus:GTEx/v8/Brain_Hypothalamus:GTEx/v8/Brain_Nucleus_accumbens_basal_ganglia:GTEx/v8/Brain_Putamen_basal_ganglia:GTEx/v8/Brain_Spinal_cord_cervical_c-1:GTEx/v8/Brain_Substantia_nigra
rs62063686	ENSG00000262539	*RP11-259G18.3*	17	44336917	44337972	-1	pseudogene	NA	NA	NA	NA	2880	3.51E-45	1.60E-37	GTEx/v8/Brain_Amygdala:GTEx/v8/Brain_Anterior_cingulate_cortex_BA24:GTEx/v8/Brain_Caudate_basal_ganglia:GTEx/v8/Brain_Cerebellar_Hemisphere:GTEx/v8/Brain_Cerebellum:GTEx/v8/Brain_Cortex:GTEx/v8/Brain_Frontal_Cortex_BA9:GTEx/v8/Brain_Hippocampus:GTEx/v8/Brain_Hypothalamus:GTEx/v8/Brain_Nucleus_accumbens_basal_ganglia:GTEx/v8/Brain_Putamen_basal_ganglia:GTEx/v8/Brain_Spinal_cord_cervical_c-1:GTEx/v8/Brain_Substantia_nigra
rs62063686	ENSG00000261575	*RP11-259G18.1*	17	44344403	44346060	1	pseudogene	NA	NA	NA	NA	2880	7.46E-51	1.13E-41	GTEx/v8/Brain_Amygdala:GTEx/v8/Brain_Anterior_cingulate_cortex_BA24:GTEx/v8/Brain_Caudate_basal_ganglia:GTEx/v8/Brain_Cerebellar_Hemisphere:GTEx/v8/Brain_Cerebellum:GTEx/v8/Brain_Cortex:GTEx/v8/Brain_Frontal_Cortex_BA9:GTEx/v8/Brain_Hippocampus:GTEx/v8/Brain_Hypothalamus:GTEx/v8/Brain_Nucleus_accumbens_basal_ganglia:GTEx/v8/Brain_Putamen_basal_ganglia:GTEx/v8/Brain_Spinal_cord_cervical_c-1:GTEx/v8/Brain_Substantia_nigra
rs62063686	ENSG00000228696	*ARL17B*	17	44352150	44439130	-1	protein_coding	100506084	ARL17B	NA	-0.428243465	32	4.31E-08	2.36E-17	GTEx/v8/Brain_Cerebellar_Hemisphere:GTEx/v8/Brain_Cerebellum:GTEx/v8/Brain_Hypothalamus
rs62063686	ENSG00000176681	*LRRC37A*	17	44370099	44415160	1	protein_coding	9884	LRRC37A	0.890487356	NA	2879	1.21E-30	4.87E-42	GTEx/v8/Brain_Amygdala:GTEx/v8/Brain_Anterior_cingulate_cortex_BA24:GTEx/v8/Brain_Caudate_basal_ganglia:GTEx/v8/Brain_Cerebellar_Hemisphere:GTEx/v8/Brain_Cerebellum:GTEx/v8/Brain_Cortex:GTEx/v8/Brain_Frontal_Cortex_BA9:GTEx/v8/Brain_Hippocampus:GTEx/v8/Brain_Hypothalamus:GTEx/v8/Brain_Nucleus_accumbens_basal_ganglia:GTEx/v8/Brain_Putamen_basal_ganglia:GTEx/v8/Brain_Spinal_cord_cervical_c-1:GTEx/v8/Brain_Substantia_nigra
rs62063686	ENSG00000238083	*LRRC37A2*	17	44588877	44633016	1	protein_coding	474170	LRRC37A2	NA	NA	2733	2.68E-52	7.91E-44	GTEx/v8/Brain_Amygdala:GTEx/v8/Brain_Anterior_cingulate_cortex_BA24:GTEx/v8/Brain_Caudate_basal_ganglia:GTEx/v8/Brain_Cerebellar_Hemisphere:GTEx/v8/Brain_Cerebellum:GTEx/v8/Brain_Cortex:GTEx/v8/Brain_Frontal_Cortex_BA9:GTEx/v8/Brain_Hippocampus:GTEx/v8/Brain_Hypothalamus:GTEx/v8/Brain_Nucleus_accumbens_basal_ganglia:GTEx/v8/Brain_Putamen_basal_ganglia:GTEx/v8/Brain_Spinal_cord_cervical_c-1:GTEx/v8/Brain_Substantia_nigra
rs62063686	ENSG00000185829	*ARL17A*	17	44594068	44657088	-1	protein_coding	51326	ARL17A	NA	-2.38258287	2733	4.39E-38	7.42E-31	GTEx/v8/Brain_Amygdala:GTEx/v8/Brain_Anterior_cingulate_cortex_BA24:GTEx/v8/Brain_Caudate_basal_ganglia:GTEx/v8/Brain_Cerebellar_Hemisphere:GTEx/v8/Brain_Cerebellum:GTEx/v8/Brain_Cortex:GTEx/v8/Brain_Frontal_Cortex_BA9:GTEx/v8/Brain_Hippocampus:GTEx/v8/Brain_Hypothalamus:GTEx/v8/Brain_Nucleus_accumbens_basal_ganglia:GTEx/v8/Brain_Putamen_basal_ganglia
rs62063686	ENSG00000232300	*FAM215B*	17	44636196	44640161	-1	sense_intronic	644297	FAM215B	NA	NA	2733	6.29E-24	2.68E-18	GTEx/v8/Brain_Cerebellar_Hemisphere:GTEx/v8/Brain_Cerebellum
rs62063686	ENSG00000073969	*NSF*	17	44668035	44834830	1	protein_coding	4905	NSF	0.71368213	-0.566246769	2416	1.64E-06	2.14E-10	GTEx/v8/Brain_Cerebellum
rs62063686	ENSG00000108379	*WNT3*	17	44839872	44910520	-1	protein_coding	7473	WNT3	0.946108101	-0.191110949	28	1.53E-05	1.18E-51	GTEx/v8/Brain_Cerebellar_Hemisphere:GTEx/v8/Brain_Cerebellum:GTEx/v8/Brain_Cortex
rs62063686	ENSG00000263142	*LRRC37A17P*	17	45055847	45131935	1	pseudogene	644397	LRRC37A17P	NA	NA	925	2.50E-05	0.0172168	GTEx/v8/Brain_Cerebellum
rs62063686	ENSG00000178852	*EFCAB13*	17	45400656	45518678	1	protein_coding	124989	EFCAB13	8.22E-19	-0.190444586	3	4.77E-05	1.78E-07	GTEx/v8/Brain_Anterior_cingulate_cortex_BA24
rs34822974	ENSG00000168813	*ZNF507*	19	32836500	32878573	1	protein_coding	22847	ZNF507	0.885285924	-0.678666536	1	2.15E-06	0.00968915	GTEx/v8/Brain_Frontal_Cortex_BA9
rs34822974	ENSG00000124299	*PEPD*	19	33877856	34012700	-1	protein_coding	5184	PEPD	0.014507135	0.225176003	26	5.59E-07	1.86E-11	GTEx/v8/Brain_Cerebellar_Hemisphere
rs34822974	ENSG00000124302	*CHST8*	19	34112861	34264414	1	protein_coding	64377	CHST8	0.004222016	-1.382729988	31	5.44E-06	0.0157132	GTEx/v8/Brain_Cortex
rs1482419	ENSG00000224924	*LINC00320*	21	22114908	22175534	-1	lincRNA	387486	LINC00320	NA	NA	66	2.19E-13	1.07E-10	GTEx/v8/Brain_Amygdala:GTEx/v8/Brain_Anterior_cingulate_cortex_BA24:GTEx/v8/Brain_Caudate_basal_ganglia:GTEx/v8/Brain_Cerebellar_Hemisphere:GTEx/v8/Brain_Cerebellum:GTEx/v8/Brain_Cortex:GTEx/v8/Brain_Frontal_Cortex_BA9:GTEx/v8/Brain_Hippocampus:GTEx/v8/Brain_Hypothalamus:GTEx/v8/Brain_Nucleus_accumbens_basal_ganglia:GTEx/v8/Brain_Putamen_basal_ganglia:GTEx/v8/Brain_Spinal_cord_cervical_c-1
rs8137258	ENSG00000236540	*AC006547.13*	22	20050503	20058066	-1	antisense	NA	NA	NA	NA	22	3.87E-06	0.000102016	GTEx/v8/Brain_Caudate_basal_ganglia:GTEx/v8/Brain_Cortex

rs8137258	ENSG00000099899	*TRMT2A*	22	20099389	20104915	-1	protein_coding	27037	TRMT2A	1.31E-05	NA	5	2.59E-05	0.0244377	GTEx/v8/Brain_Cerebellar_Hemisphere
rs8137258	ENSG00000099904	*ZDHHC8*	22	20116979	20135530	1	protein_coding	29801	ZDHHC8	0.988991096	NA	7	9.68E-06	0.000390337	GTEx/v8/Brain_Caudate_basal_ganglia:GTEx/v8/Brain_Cerebellum
rs5769765	ENSG00000260613	*RP3-522J7.6*	22	50226264	50231434	-1	lincRNA	NA	NA	NA	NA	197	1.05E-19	1.15E-18	GTEx/v8/Brain_Cerebellar_Hemisphere:GTEx/v8/Brain_Cerebellum
rs5769765	ENSG00000100426	*ZBED4*	22	50247490	50283726	1	protein_coding	9889	ZBED4	0.989506768	-0.580937239	197	2.29E-14	1.12E-09	GTEx/v8/Brain_Cerebellar_Hemisphere:GTEx/v8/Brain_Cerebellum
rs5769765	ENSG00000182858	*ALG12*	22	50293877	50312106	-1	protein_coding	79087	ALG12	0.007881983	NA	196	1.10E-12	4.38E-08	GTEx/v8/Brain_Anterior_cingulate_cortex_BA24:GTEx/v8/Brain_Caudate_basal_ganglia:GTEx/v8/Brain_Cortex:GTEx/v8/Brain_Frontal_Cortex_BA9:GTEx/v8/Brain_Hippocampus:GTEx/v8/Brain_Nucleus_accumbens_basal_ganglia:GTEx/v8/Brain_Putamen_basal_ganglia
rs5769765	ENSG00000184164	*CRELD2*	22	50311815	50321188	1	protein_coding	79174	CRELD2	4.96E-08	NA	180	2.28E-08	0.00049474	GTEx/v8/Brain_Hippocampus:GTEx/v8/Brain_Hypothalamus:GTEx/v8/Brain_Nucleus_accumbens_basal_ganglia:GTEx/v8/Brain_Putamen_basal_ganglia

b) Bipolar I disorder

IndSigSNPs	ensg	symbol	chr	start	end	strand	type	entrezID	HUGO	pLI	ncRVIS	eqtlMapSNPs	eqtlMapminP	eqtlMapminQ	eqtlMapts
rs7528296	ENSG00000135845	*PIGC*	1	172339329	172413230	-1	protein_coding	5279	PIGC	0.000110183	-0.675062169	6	2.20E-07	2.52E-08	GTEx/v8/Brain_Cerebellar_Hemisphere:GTEx/v8/Brain_Cerebellum
rs7536102	ENSG00000117593	*DARS2*	1	173793641	173827684	1	protein_coding	55157	DARS2	4.41E-09	-1.457801096	14	6.37E-06	7.20E-08	GTEx/v8/Brain_Cortex:GTEx/v8/Brain_Putamen_basal_ganglia
rs7536102	ENSG00000117601	*SERPINC1*	1	173872947	173886516	-1	protein_coding	462	SERPINC1	0.993263587	-0.017134704	14	3.88E-05	2.63E-08	GTEx/v8/Brain_Caudate_basal_ganglia:GTEx/v8/Brain_Nucleus_accumbens_basal_ganglia
rs7536102	ENSG00000227373	*RP11-160H22.5*	1	174084438	174129142	-1	lincRNA	102724601	LOC102724601	NA	NA	673	3.60E-08	0.000112265	GTEx/v8/Brain_Cortex:GTEx/v8/Brain_Frontal_Cortex_BA9:GTEx/v8/Brain_Substantia_nigra
rs7536102	ENSG00000152061	*RABGAP1L*	1	174128548	174964445	1	protein_coding	9910	RABGAP1L	0.089172714	NA	90	1.59E-05	0.000816762	GTEx/v8/Brain_Frontal_Cortex_BA9
rs1976772	ENSG00000065413	*ANKRD44*	2	197831741	198175897	-1	protein_coding	91526	ANKRD44	0.775902812	NA	6	0.000147741	0.0402321	GTEx/v8/Brain_Cerebellar_Hemisphere
rs1976772	ENSG00000115524	*SF3B1*	2	198254508	198299815	-1	protein_coding	23451	SF3B1	0.999999973	-0.619089113	304	9.07E-09	3.02E-05	GTEx/v8/Brain_Caudate_basal_ganglia:GTEx/v8/Brain_Cerebellar_Hemisphere:GTEx/v8/Brain_Cerebellum:GTEx/v8/Brain_Hypothalamus
rs6897189	ENSG00000145687	*SSBP2*	5	80708840	81047616	-1	protein_coding	23635	SSBP2	0.976772519	0.270330685	58	5.43E-06	5.64E-05	GTEx/v8/Brain_Cerebellar_Hemisphere:GTEx/v8/Brain_Cerebellum
rs5573737 2	ENSG00000132911	*NMUR2*	5	151771093	151812929	-1	protein_coding	56923	NMUR2	0.421657261	0.587097871	213	4.38E-10	6.78E-12	GTEx/v8/Brain_Cortex:GTEx/v8/Brain_Frontal_Cortex_BA9
rs55737372	ENSG00000249484	*AC091969.1*	5	151998525	152603103	-1	lincRNA	101927134	LINC01470	NA	NA	319	1.38E-08	8.17E-05	GTEx/v8/Brain_Caudate_basal_ganglia:GTEx/v8/Brain_Putamen_basal_ganglia
rs59137082	ENSG00000161904	*LEMD2*	6	33738979	33756913	-1	protein_coding	221496	LEMD2	0.302373361	0.392920076	40	7.55E-08	5.44E-11	GTEx/v8/Brain_Caudate_basal_ganglia:GTEx/v8/Brain_Cerebellar_Hemisphere:GTEx/v8/Brain_Cortex:GTEx/v8/Brain_Frontal_Cortex_BA9:GTEx/v8/Brain_Hypothalamus:GTEx/v8/Brain_Putamen_basal_ganglia
rs1180184	ENSG00000065833	*ME1*	6	83920108	84140797	-1	protein_coding	4199	ME1	2.22E-10	-0.796952931	69	3.43E-09	2.49E-07	GTEx/v8/Brain_Cerebellar_Hemisphere:GTEx/v8/Brain_Cerebellum:GTEx/v8/Brain_Spinal_cord_cervical_c-1
rs1180184	ENSG00000146250	*PRSS35*	6	84222194	84235423	1	protein_coding	167681	PRSS35	1.34E-06	-0.251367409	36	2.64E-06	0.00204729	GTEx/v8/Brain_Hypothalamus:GTEx/v8/Brain_Spinal_cord_cervical_c-1
rs12672003	ENSG00000105928	*DFNA5*	7	24737972	24809244	-1	protein_coding	1687	DFNA5	1.22E-05	0.144414547	63	2.09E-07	5.84E-19	GTEx/v8/Brain_Hippocampus:GTEx/v8/Brain_Nucleus_accumbens_basal_ganglia
rs11560354	ENSG00000188372	*ZP3*	7	76026835	76071388	1	protein_coding	7784	ZP3	0.001596815	0.259666259	29	4.77E-09	2.52E-22	GTEx/v8/Brain_Anterior_cingulate_cortex_BA24:GTEx/v8/Brain_Caudate_basal_ganglia:GTEx/v8/Brain_Cerebellar_Hemisphere:GTEx/v8/Brain_Cerebellum:GTEx/v8/Brain_Hypothalamus:GTEx/v8/Brain_Nucleus_accumbens_basal_ganglia:GTEx/v8/Brain_Putamen_basal_ganglia:GTEx/v8/Brain_Spinal_cord_cervical_c-1
rs11560354	ENSG00000243566	*UPK3B*	7	76139745	76648340	1	protein_coding	105375355	UPK3B	0.000192328	-0.147576454	28	1.09E-09	4.28E-20	GTEx/v8/Brain_Cerebellum:GTEx/v8/Brain_Cortex:GTEx/v8/Brain_Frontal_Cortex_BA9:GTEx/v8/Brain_Hypothalamus
rs11560354	ENSG00000146707	*POMZP3*	7	76239303	76256578	-1	protein_coding	22932	POMZP3	0.879121225	NA	31	7.70E-20	1.85E-37	GTEx/v8/Brain_Amygdala:GTEx/v8/Brain_Anterior_cingulate_cortex_BA24:GTEx/v8/Brain_Caudate_basal_ganglia:GTEx/v8/Brain_Cerebellar_Hemisphere:GTEx/v8/Brain_Cerebellum:GTEx/v8/Brain_Cortex:GTEx/v8/Brain_Frontal_Cortex_BA9:GTEx/v8/Brain_Hippocampus:GTEx/v8/Brain_Hypothalamus:GTEx/v8/Brain_Nucleus_accumbens_basal_ganglia:GTEx/v8/Brain_Putamen_basal_ganglia:GTEx/v8/Brain_Spinal_cord_cervical_c-1
rs4726225	ENSG00000090263	*MRPS33*	7	140705854	140715028	-1	protein_coding	51650	MRPS33	0.003395718	0.268392925	51	2.30E-09	1.80E-05	GTEx/v8/Brain_Caudate_basal_ganglia:GTEx/v8/Brain_Cortex:GTEx/v8/Brain_Putamen_basal_ganglia
rs144225206	ENSG00000205106	*CTD-2210P24.4*	11	45792983	45793909	1	protein_coding	374387	DKFZp779M0652	NA	NA	14	2.36E-08	3.31E-08	GTEx/v8/Brain_Caudate_basal_ganglia:GTEx/v8/Brain_Cortex:GTEx/v8/Brain_Frontal_Cortex_BA9:GTEx/v8/Brain_Nucleus_accumbens_basal_ganglia:GTEx/v8/Brain_Putamen_basal_ganglia

rs893949	ENSG00000109956	*B3GAT1*	11	134248398	134281812	-1	protein_coding	27087	B3GAT1	0.789152807	2.202818697	1	7.29E-06	0.0265846	GTEx/v8/Brain_Caudate_basal_ganglia
rs4760255	ENSG00000123329	*ARHGAP9*	12	57866038	57882597	-1	protein_coding	64333	ARHGAP9	2.89E-09	-0.374064649	24	1.59E-10	9.83E-07	GTEx/v8/Brain_Cerebellar_Hemisphere:GTEx/v8/Brain_Cerebellum
rs4760255	ENSG00000166986	*MARS*	12	57869228	57911352	1	protein_coding	4141	MARS	2.74E-12	-0.447643065	13	4.40E-07	0.000942149	GTEx/v8/Brain_Cerebellum
rs4760255	ENSG00000166987	*MBD6*	12	57914493	57923931	1	protein_coding	114785	MBD6	0.9636575	NA	7	5.44E-05	0.000957867	GTEx/v8/Brain_Caudate_basal_ganglia
rs4760255	ENSG00000123427	*METTL21B*	12	58165275	58176324	1	protein_coding	25895	METTL21B	0.00509294	-0.795239894	7	4.03E-05	7.14E-32	GTEx/v8/Brain_Cerebellar_Hemisphere
rs9549704	ENSG00000126231	*PROZ*	13	113812968	113826694	1	protein_coding	8858	PROZ	3.84E-09	-0.017048435	136	1.45E-26	5.85E-21	GTEx/v8/Brain_Cerebellar_Hemisphere:GTEx/v8/Brain_Cerebellum:GTEx/v8/Brain_Hippocampus
rs9549704	ENSG00000139842	*CUL4A*	13	113862552	113919399	1	protein_coding	8451	CUL4A	0.999412877	NA	55	8.83E-08	0.000435856	GTEx/v8/Brain_Cerebellum
rs1715887	ENSG00000247982	*LINC00926*	15	57592563	57599959	1	lincRNA	283663	LINC00926	NA	NA	8	3.64E-06	7.21E-24	GTEx/v8/Brain_Caudate_basal_ganglia:GTEx/v8/Brain_Cerebellar_Hemisphere
rs3145	ENSG00000245975	*RP11-30K9.6*	15	59060271	59063173	-1	lincRNA	101928725	LOC101928725	NA	NA	62	7.24E-09	4.62E-08	GTEx/v8/Brain_Amygdala:GTEx/v8/Brain_Anterior_cingulate_cortex_BA24:GTEx/v8/Brain_Hippocampus:GTEx/v8/Brain_Hypothalamus:GTEx/v8/Brain_Nucleus_accumbens_basal_ganglia:GTEx/v8/Brain_Putamen_basal_ganglia:GTEx/v8/Brain_Spinal_cord_cervical_c-1
rs11856299	ENSG00000169609	*C15orf40*	15	83657193	83680393	-1	protein_coding	123207	C15orf40	0.268492643	0.09693066	172	6.97E-23	8.73E-30	GTEx/v8/Brain_Amygdala:GTEx/v8/Brain_Anterior_cingulate_cortex_BA24:GTEx/v8/Brain_Caudate_basal_ganglia:GTEx/v8/Brain_Cerebellar_Hemisphere:GTEx/v8/Brain_Cerebellum:GTEx/v8/Brain_Cortex:GTEx/v8/Brain_Frontal_Cortex_BA9:GTEx/v8/Brain_Hippocampus:GTEx/v8/Brain_Hypothalamus:GTEx/v8/Brain_Nucleus_accumbens_basal_ganglia:GTEx/v8/Brain_Putamen_basal_ganglia:GTEx/v8/Brain_Spinal_cord_cervical_c-1:GTEx/v8/Brain_Substantia_nigra
rs11856299	ENSG00000064726	*BTBD1*	15	83685174	83736106	-1	protein_coding	53339	BTBD1	0.014946424	0.543548994	166	8.75E-07	0.000116971	GTEx/v8/Brain_Frontal_Cortex_BA9:GTEx/v8/Brain_Hippocampus:GTEx/v8/Brain_Nucleus_accumbens_basal_ganglia:GTEx/v8/Brain_Putamen_basal_ganglia
rs4702	ENSG00000140564	*FURIN*	15	91411822	91426688	1	protein_coding	5045	FURIN	0.999933301	-0.156379719	1	2.51E-05	0.0433875	GTEx/v8/Brain_Frontal_Cortex_BA9
rs12925547	ENSG00000103044	*HAS3*	16	69139467	69152622	1	protein_coding	3038	HAS3	0.269387112	-0.838005778	4	4.69E-05	0.0468144	GTEx/v8/Brain_Cerebellar_Hemisphere
rs12925547	ENSG00000141076	*CIRH1A*	16	69165194	69265033	1	protein_coding	84916	CIRH1A	0.094027705	NA	94	6.91E-09	1.84E-21	GTEx/v8/Brain_Caudate_basal_ganglia:GTEx/v8/Brain_Cortex:GTEx/v8/Brain_Frontal_Cortex_BA9:GTEx/v8/Brain_Hippocampus:GTEx/v8/Brain_Nucleus_accumbens_basal_ganglia:GTEx/v8/Brain_Putamen_basal_ganglia
rs12925547	ENSG00000168807	*SNTB2*	16	69221032	69342955	1	protein_coding	6645	SNTB2	0.036218318	-1.417098756	98	1.63E-08	5.96E-05	GTEx/v8/Brain_Cerebellar_Hemisphere:GTEx/v8/Brain_Cerebellum
rs12925547	ENSG00000132612	*VPS4A*	16	69345259	69358949	1	protein_coding	27183	VPS4A	NA	NA	110	3.35E-14	1.09E-09	GTEx/v8/Brain_Anterior_cingulate_cortex_BA24:GTEx/v8/Brain_Caudate_basal_ganglia:GTEx/v8/Brain_Cortex:GTEx/v8/Brain_Frontal_Cortex_BA9:GTEx/v8/Brain_Hippocampus:GTEx/v8/Brain_Hypothalamus:GTEx/v8/Brain_Nucleus_accumbens_basal_ganglia:GTEx/v8/Brain_Putamen_basal_ganglia
rs12925547	ENSG00000213380	*COG8*	16	69354043	69373570	-1	protein_coding	84342	COG8	1.87E-05	-0.173605021	77	4.72E-07	0.00177363	GTEx/v8/Brain_Cortex:GTEx/v8/Brain_Frontal_Cortex_BA9
rs12925547	ENSG00000258429	*PDF*	16	69362524	69364498	-1	protein_coding	64146	PDF	0.050315418	-0.040959267	3	9.46E-05	0.0152748	GTEx/v8/Brain_Cortex
rs12925547	ENSG00000132603	*NIP7*	16	69373333	69377014	1	protein_coding	51388	NIP7	0.603152383	1.129236796	106	3.71E-07	0.00146488	GTEx/v8/Brain_Frontal_Cortex_BA9:GTEx/v8/Brain_Nucleus_accumbens_basal_ganglia:GTEx/v8/Brain_Putamen_basal_ganglia
rs12925547	ENSG00000132604	*TERF2*	16	69389464	69442474	-1	protein_coding	7014	TERF2	0.927472177	-0.576801065	90	1.64E-10	8.32E-07	GTEx/v8/Brain_Cerebellar_Hemisphere:GTEx/v8/Brain_Cerebellum:GTEx/v8/Brain_Cortex
rs4790841	ENSG00000185924	*RTN4RL1*	17	1837971	1928639	-1	protein_coding	146760	RTN4RL1	0.787530525	NA	1	1.20E-05	0.00258335	GTEx/v8/Brain_Cerebellar_Hemisphere
rs7502233	ENSG00000008838	*MED24*	17	38175350	38217468	-1	protein_coding	9862	MED24	8.99E-09	1.28719637	39	1.05E-13	4.47E-09	GTEx/v8/Brain_Anterior_cingulate_cortex_BA24:GTEx/v8/Brain_Caudate_basal_ganglia:GTEx/v8/Brain_Cerebellum:GTEx/v8/Brain_Cortex:GTEx/v8/Brain_Frontal_Cortex_BA9:GTEx/v8/Brain_Hippocampus:GTEx/v8/Brain_Hypothalamus:GTEx/v8/Brain_Nucleus_accumbens_basal_ganglia
rs2274938	ENSG00000228812	*RP11-157P1.5*	20	60928051	60931536	1	antisense	101928158	LAMA5-AS1	NA	NA	4	7.38E-06	0.00551443	GTEx/v8/Brain_Cortex
rs2836171	ENSG00000157542	*KCNJ6*	21	38979678	39288749	-1	protein_coding	3763	KCNJ6	0.812451385	-0.293776228	185	2.48E-11	3.24E-07	GTEx/v8/Brain_Caudate_basal_ganglia
rs9606265	ENSG00000099899	*TRMT2A*	22	20099389	20104915	-1	protein_coding	27037	TRMT2A	1.31E-05	NA	5	2.59E-05	0.0244377	GTEx/v8/Brain_Cerebellar_Hemisphere
rs9606265	ENSG00000099904	*ZDHHC8*	22	20116979	20135530	1	protein_coding	29801	ZDHHC8	0.988991096	NA	5	1.56E-05	0.0313939	GTEx/v8/Brain_Caudate_basal_ganglia

c) Major depressive disorder

IndSigSNPs	ensg	symbol	chr	start	end	strand	type	entrezID	HUGO	pLI	ncRVIS	eqtlMapSNPs	eqtlMapminP	eqtlMapminQ	eqtlMapts
rs11211481	ENSG00000162366	*PDZK1IP1*	1	47649265	47656716	-1	protein_coding	10158	PDZK1IP1	0.000484065	-0.343318344	38	6.02E-06	2.89E-10	GTEx/v8/Brain_Cortex:GTEx/v8/Brain_Putamen_basal_ganglia
rs11211481	ENSG00000162367	*TAL1*	1	47681962	47697892	-1	protein_coding	6886	TAL1	0.824692215	-0.797257236	42	7.92E-06	0.0180879	GTEx/v8/Brain_Cortex
rs11211481	ENSG00000186790	*FOXE3*	1	47881744	47883723	1	protein_coding	2301	FOXE3	NA	-0.345111816	40	3.21E-06	1.28E-08	GTEx/v8/Brain_Cerebellum
rs1730865	ENSG00000198890	*PRMT6*	1	107599267	107601916	1	protein_coding	55170	PRMT6	0.024127621	-0.632333634	40	1.04E-14	4.59E-10	GTEx/v8/Brain_Anterior_cingulate_cortex_BA24:GTEx/v8/Brain_Caudate_basal_ganglia:GTEx/v8/Brain_Cerebellar_Hemisphere:GTEx/v8/Brain_Cerebellum:GTEx/v8/Brain_Cortex:GTEx/v8/Brain_Frontal_Cortex_BA9:GTEx/v8/Brain_Hippocampus:GTEx/v8/Brain_Hypothalamus:GTEx/v8/Brain_Putamen_basal_ganglia:GTEx/v8/Brain_Spinal_cord_cervical_c-1:GTEx/v8/Brain_Substantia_nigra
rs6674701	ENSG00000143801	*PSEN2*	1	227057885	227083806	1	protein_coding	5664	PSEN2	0.027014182	0.479322645	41	3.19E-08	2.60E-04	GTEx/v8/Brain_Caudate_basal_ganglia:GTEx/v8/Brain_Frontal_Cortex_BA9:GTEx/v8/Brain_Hypothalamus:GTEx/v8/Brain_Putamen_basal_ganglia
rs6674701	ENSG00000163050	*ADCK3*	1	227085237	227175246	1	protein_coding	56997	ADCK3	1.235E-05	0.831024079	42	9.30E-08	4.56E-09	GTEx/v8/Brain_Cerebellar_Hemisphere:GTEx/v8/Brain_Cerebellum:GTEx/v8/Brain_Cortex
rs56211578	ENSG00000273080	*RP11-301O19.1*	2	86422713	86423172	1	antisense	NA	NA	NA	NA	9	4.43E-05	2.00E-25	GTEx/v8/Brain_Caudate_basal_ganglia:GTEx/v8/Brain_Nucleus_accumbens_basal_ganglia
rs56211578	ENSG00000115561	*CHMP3*	2	86730554	86948245	-1	protein_coding	51652	CHMP3	0.036245866	-0.19401976	139	2.22E-12	5.21448E-08	GTEx/v8/Brain_Caudate_basal_ganglia:GTEx/v8/Brain_Cortex:GTEx/v8/Brain_Frontal_Cortex_BA9:GTEx/v8/Brain_Nucleus_accumbens_basal_ganglia:GTEx/v8/Brain_Putamen_basal_ganglia
rs56211578	ENSG00000183281	*PLGLB1*	2	87229682	87248975	-1	protein_coding	5343	PLGLB1	NA	-0.640421533	128	5.26E-07	4.221E-22	GTEx/v8/Brain_Caudate_basal_ganglia:GTEx/v8/Brain_Cerebellum:GTEx/v8/Brain_Putamen_basal_ganglia
rs7631372	ENSG00000254485	*RP11-380O24.1*	3	9334272	9404987	-1	antisense	NA	NA	NA	NA	8	5.81E-06	2.25E-02	GTEx/v8/Brain_Cerebellar_Hemisphere:GTEx/v8/Brain_Nucleus_accumbens_basal_ganglia
rs7631372	ENSG00000206573	*SETD5-AS1*	3	9391373	9440263	-1	antisense	440944	SETD5-AS1	NA	NA	32	1.93E-07	7.18E-05	GTEx/v8/Brain_Cerebellar_Hemisphere
rs7631372	ENSG00000134077	*THUMPD3*	3	9404526	9428475	1	protein_coding	25917	THUMPD3	3.99618E-11	0.700963169	43	9.69E-19	1.57E-13	GTEx/v8/Brain_Amygdala:GTEx/v8/Brain_Anterior_cingulate_cortex_BA24:GTEx/v8/Brain_Caudate_basal_ganglia:GTEx/v8/Brain_Cerebellar_Hemisphere:GTEx/v8/Brain_Cerebellum:GTEx/v8/Brain_Cortex:GTEx/v8/Brain_Frontal_Cortex_BA9:GTEx/v8/Brain_Hippocampus:GTEx/v8/Brain_Hypothalamus:GTEx/v8/Brain_Nucleus_accumbens_basal_ganglia:GTEx/v8/Brain_Putamen_basal_ganglia:GTEx/v8/Brain_Spinal_cord_cervical_c-1:GTEx/v8/Brain_Substantia_nigra
rs9839427	ENSG00000173531	*MST1*	3	49721380	49726934	-1	protein_coding	4485	MST1	1.72736E-10	0.158165154	1	1.39E-04	0.000201734	GTEx/v8/Brain_Hippocampus
rs7635184	ENSG00000187672	*ERC2*	3	55542336	56502391	-1	protein_coding	26059	ERC2	0.996856462	-1.915465352	13	3.21E-05	1.08E-15	GTEx/v8/Brain_Cerebellum
rs7635184	ENSG00000163946	*FAM208A*	3	56654161	56717265	-1	protein_coding	23272	FAM208A	1.00E+00	NA	11	5.00E-05	8.41E-05	GTEx/v8/Brain_Cerebellum
rs3905238	ENSG00000249673	*NOP14-AS1*	4	2936626	2963465	1	antisense	317648	NOP14-AS1	NA	NA	5	0.000011576	2.89E-02	GTEx/v8/Brain_Cerebellar_Hemisphere
rs3905238	ENSG00000125388	*GRK4*	4	2965335	3042474	1	protein_coding	2868	GRK4	5.69042E-07	NA	9	3.39E-07	2.32E-27	GTEx/v8/Brain_Cerebellum
rs3905238	ENSG00000188981	*MSANTD1*	4	3246096	3273465	1	protein_coding	345222	MSANTD1	0.322172214	-0.464369361	11	1.49E-07	3.74E-08	GTEx/v8/Brain_Cerebellar_Hemisphere:GTEx/v8/Brain_Cerebellum
rs9990752	ENSG00000237765	*FAM200B*	4	15683285	15707188	1	protein_coding	285550	FAM200B	NA	-0.311233821	4	3.08E-10	1.86E-12	GTEx/v8/Brain_Anterior_cingulate_cortex_BA24:GTEx/v8/Brain_Caudate_basal_ganglia:GTEx/v8/Brain_Cerebellar_Hemisphere:GTEx/v8/Brain_Cerebellum:GTEx/v8/Brain_Cortex:GTEx/v8/Brain_Frontal_Cortex_BA9:GTEx/v8/Brain_Hypothalamus:GTEx/v8/Brain_Nucleus_accumbens_basal_ganglia:GTEx/v8/Brain_Putamen_basal_ganglia
rs12511027	ENSG00000174125	*TLR1*	4	38792298	38858438	-1	protein_coding	7096	TLR1	2.02407E-14	0.436460923	1	4.45E-05	3.25E-02	GTEx/v8/Brain_Cerebellum
rs10022087	ENSG00000182308	*DCAF4L1*	4	41983713	41988476	1	protein_coding	285429	DCAF4L1	0.851426397	0.913124873	1	5.74E-05	0.0158831	GTEx/v8/Brain_Caudate_basal_ganglia
rs10022087	ENSG00000014824	*SLC30A9*	4	41992489	42092474	1	protein_coding	10463	SLC30A9	0.001423485	-0.907696884	50	2.46E-11	2.46E-09	GTEx/v8/Brain_Amygdala:GTEx/v8/Brain_Anterior_cingulate_cortex_BA24:GTEx/v8/Brain_Caudate_basal_ganglia:GTEx/v8/Brain_Cerebellum:GTEx/v8/Brain_Cortex:GTEx/v8/Brain_Hypothalamus:GTEx/v8/Brain_Nucleus_accumbens_basal_ganglia:GTEx/v8/Brain_Putamen_basal_ganglia:GTEx/v8/Brain_Substantia_nigra
rs10022087	ENSG00000188848	*BEND4*	4	42112955	42154895	-1	protein_coding	389206	BEND4	9.87E-01	1.717247406	25	3.67E-06	1.26E-02	GTEx/v8/Brain_Hippocampus
rs12196783	ENSG00000112249	*ASCC3*	6	100956070	101329248	-1	protein_coding	10973	ASCC3	1.43E-10	0.106685768	208	3.78E-09	1.60E-05	GTEx/v8/Brain_Caudate_basal_ganglia:GTEx/v8/Brain_Cerebellum:GTEx/v8/Brain_Cortex
rs12196783	ENSG00000260000	*RP3-467N11.1*	6	101329347	101330863	1	antisense	NA	NA	NA	NA	76	9.57E-06	2.49E-02	GTEx/v8/Brain_Anterior_cingulate_cortex_BA24
rs12526217	ENSG00000164483	*SAMD3*	6	130465460	130686570	-1	protein_coding	154075	SAMD3	1.09708E-06	0.088198284	65	5.84E-07	0.00198934	GTEx/v8/Brain_Cortex
rs4895718	ENSG00000203727	*SAMD5*	6	147830063	148058683	1	protein_coding	389432	SAMD5	0.000898004	1.680995061	56	6.88E-15	2.81E-10	GTEx/v8/Brain_Cerebellar_Hemisphere:GTEx/v8/Brain_Cerebellum:GTEx/v8/Brain_Hypothalamus
rs9800952	ENSG00000090263	*MRPS33*	7	140705854	140715028	-1	protein_coding	51650	MRPS33	0.003395718	0.268392925	51	2.29778E-09	1.80207E-05	GTEx/v8/Brain_Caudate_basal_ganglia:GTEx/v8/Brain_Cortex:GTEx/v8/Brain_Putamen_basal_ganglia
rs11250015	ENSG00000261451	*RP11-981G7.1*	8	10291182	10295822	1	sense_overlapping	NA	NA	NA	NA	32	1.64E-09	6.07003E-19	GTEx/v8/Brain_Cerebellar_Hemisphere:GTEx/v8/Brain_Cerebellum:GTEx/v8/Brain_Cortex

rs11250015	ENSG00000253641	*RP11-981G7.2*	8	10332075	10339484	1	lincRNA	101929191	LINCR-0001	NA	NA	20	3.98E-06	3.40774E-05	GTEx/v8/Brain_Caudate_basal_ganglia
rs11250015	ENSG00000253649	*PRSS51*	8	10340388	10405095	-1	antisense	346702	PRSS51	NA	NA	33	5.26E-09	9.41E-08	GTEx/v8/Brain_Cerebellar_Hemisphere:GTEx/v8/Brain_Cerebellum
rs7842361	ENSG00000182197	*EXT1*	8	118806729	119124092	-1	protein_coding	2131	EXT1	0.999320485	0.987829441	1	3.77E-05	8.57E-03	GTEx/v8/Brain_Caudate_basal_ganglia
rs876575	ENSG00000153317	*ASAP1*	8	131064353	131455906	-1	protein_coding	50807	ASAP1	0.999983091	0.592920078	50	1.01E-08	1.01E-16	GTEx/v8/Brain_Cerebellar_Hemisphere:GTEx/v8/Brain_Cerebellum:GTEx/v8/Brain_Cortex:GTEx/v8/Brain_Nucleus_accumbens_basal_ganglia:GTEx/v8/Brain_Putamen_basal_ganglia
rs111655660	ENSG00000237385	*RP11-165J3.5*	9	96192624	96193581	-1	antisense	NA	NA	NA	NA	54	2.63E-14	2.95E-14	GTEx/v8/Brain_Cerebellum
rs111655660	ENSG00000227603	*RP11-165J3.6*	9	96197614	96199403	-1	antisense	NA	NA	NA	NA	54	2.58701E-22	9.42629E-18	GTEx/v8/Brain_Amygdala:GTEx/v8/Brain_Anterior_cingulate_cortex_BA24:GTEx/v8/Brain_Caudate_basal_ganglia:GTEx/v8/Brain_Cerebellar_Hemisphere:GTEx/v8/Brain_Cerebellum:GTEx/v8/Brain_Cortex:GTEx/v8/Brain_Frontal_Cortex_BA9:GTEx/v8/Brain_Hippocampus:GTEx/v8/Brain_Hypothalamus:GTEx/v8/Brain_Nucleus_accumbens_basal_ganglia:GTEx/v8/Brain_Putamen_basal_ganglia:GTEx/v8/Brain_Spinal_cord_cervical_c-1:GTEx/v8/Brain_Substantia_nigra
rs10760393	ENSG00000185585	*OLFML2A*	9	127539437	127577164	1	protein_coding	169611	OLFML2A	1.74E-05	0.451719558	1	2.96E-05	1.77E-05	GTEx/v8/Brain_Caudate_basal_ganglia:GTEx/v8/Brain_Putamen_basal_ganglia
rs10760393	ENSG00000136950	*ARPC5L*	9	127624409	127640003	1	protein_coding	81873	ARPC5L	0.775599153	-1.27069518	31	4.54E-10	1.03E-17	GTEx/v8/Brain_Cerebellar_Hemisphere:GTEx/v8/Brain_Cerebellum
rs10760393	ENSG00000136935	*GOLGA1*	9	127640646	127710771	-1	protein_coding	2800	GOLGA1	6.11E-03	-0.699247797	124	2.58E-06	7.80E-05	GTEx/v8/Brain_Cerebellar_Hemisphere:GTEx/v8/Brain_Cerebellum
rs10760393	ENSG00000173611	*SCAI*	9	127704887	127905785	-1	protein_coding	286205	SCAI	9.99E-01	NA	1	5.29E-05	1.45E-02	GTEx/v8/Brain_Hippocampus
rs10760393	ENSG00000119414	*PPP6C*	9	127908852	127952218	-1	protein_coding	5537	PPP6C	0.988609354	-0.703511544	123	1.20E-08	8.55E-06	GTEx/v8/Brain_Caudate_basal_ganglia:GTEx/v8/Brain_Frontal_Cortex_BA9:GTEx/v8/Brain_Hippocampus:GTEx/v8/Brain_Putamen_basal_ganglia
rs10760393	ENSG00000232630	*PRPS1P2*	9	127912932	127913868	1	pseudogene	100421295	PRPS1P2	NA	NA	125	3.11E-10	3.26E-07	GTEx/v8/Brain_Caudate_basal_ganglia:GTEx/v8/Brain_Cerebellar_Hemisphere:GTEx/v8/Brain_Cerebellum:GTEx/v8/Brain_Hippocampus:GTEx/v8/Brain_Nucleus_accumbens_basal_ganglia
rs10760393	ENSG00000165219	*GAPVD1*	9	128024073	128129486	1	protein_coding	26130	GAPVD1	1.00E+00	-0.165865959	121	1.14E-07	9.64E-08	GTEx/v8/Brain_Cerebellar_Hemisphere:GTEx/v8/Brain_Cerebellum
rs7083756	ENSG00000225383	*SFTA1P*	10	10826400	10836943	-1	lincRNA	207107	SFTA1P	NA	NA	37	1.00E-12	4.87095E-11	GTEx/v8/Brain_Cortex:GTEx/v8/Brain_Frontal_Cortex_BA9:GTEx/v8/Brain_Hippocampus:GTEx/v8/Brain_Hypothalamus:GTEx/v8/Brain_Nucleus_accumbens_basal_ganglia:GTEx/v8/Brain_Putamen_basal_ganglia:GTEx/v8/Brain_Spinal_cord_cervical_c-1
rs10748841	ENSG00000166275	*C10orf32*	10	104613980	104624718	1	protein_coding	119032	C10orf32	3.16E-02	0.490358211	21	7.77E-06	8.55E-21	GTEx/v8/Brain_Frontal_Cortex_BA9:GTEx/v8/Brain_Putamen_basal_ganglia
rs10748841	ENSG00000214435	*AS3MT*	10	104629273	104661656	1	protein_coding	57412	AS3MT	5.09138E-07	0.726881178	1	2.09E-04	6.43038E-34	GTEx/v8/Brain_Cerebellum
rs10748841	ENSG00000076685	*NT5C2*	10	104845940	104953056	-1	protein_coding	22978	NT5C2	5.22E-02	0.04832858	5	2.62E-05	2.15E-07	GTEx/v8/Brain_Cerebellar_Hemisphere:GTEx/v8/Brain_Hippocampus
rs10748841	ENSG00000148798	*INA*	10	105036920	105050108	1	protein_coding	9118	INA	0.292917015	-0.860661456	14	1.57E-05	3.78E-03	GTEx/v8/Brain_Frontal_Cortex_BA9
rs10748841	ENSG00000148843	*PDCD11*	10	105156405	105206049	1	protein_coding	22984	PDCD11	0.468448698	-0.805378431	31	1.04E-09	5.69E-09	GTEx/v8/Brain_Cerebellar_Hemisphere:GTEx/v8/Brain_Cerebellum:GTEx/v8/Brain_Cortex
rs10748841	ENSG00000138172	*CALHM2*	10	105206543	105212660	-1	protein_coding	51063	CALHM2	5.29E-07	-0.689941205	14	2.90E-05	0.0132227	GTEx/v8/Brain_Putamen_basal_ganglia
rs1002728	ENSG00000261340	*RP11-215H22.1*	11	30001660	30003939	-1	lincRNA	106456574	LINC01616	NA	NA	16	6.59E-06	0.000655507	GTEx/v8/Brain_Caudate_basal_ganglia
rs76416526	ENSG00000086205	*FOLH1*	11	49168187	49230222	-1	protein_coding	2346	FOLH1	0.253396885	-0.094158315	60	1.67E-06	1.48E-06	GTEx/v8/Brain_Amygdala:GTEx/v8/Brain_Caudate_basal_ganglia:GTEx/v8/Brain_Cerebellum:GTEx/v8/Brain_Cortex:GTEx/v8/Brain_Nucleus_accumbens_basal_ganglia:GTEx/v8/Brain_Putamen_basal_ganglia:GTEx/v8/Brain_Spinal_cord_cervical_c-1
rs76416526	ENSG00000254714	*RP11-163O19.1*	11	49872181	49884080	1	pseudogene	NA	NA	NA	NA	20	2.95E-05	0.000158926	GTEx/v8/Brain_Caudate_basal_ganglia
rs76416526	ENSG00000255190	*TRIM51DP*	11	49896497	49898190	1	pseudogene	100419945	TRIM51DP	NA	NA	29	8.21E-05	8.08E-23	GTEx/v8/Brain_Cortex
rs2509805	ENSG00000149150	*SLC43A1*	11	57252007	57283259	-1	protein_coding	8501	SLC43A1	7.85327E-05	-0.136928307	5	4.46E-05	2.48319E-08	GTEx/v8/Brain_Cerebellum
rs2509805	ENSG00000134809	*TIMM10*	11	57295936	57298276	-1	protein_coding	26519	TIMM10	9.49E-03	0.000100078	7	1.68E-05	7.81745E-13	GTEx/v8/Brain_Caudate_basal_ganglia
rs2509805	ENSG00000156603	*MED19*	11	57471186	57479693	-1	protein_coding	219541	MED19	0.683790051	-0.151700149	83	5.44E-06	5.15E-07	GTEx/v8/Brain_Cerebellar_Hemisphere:GTEx/v8/Brain_Cerebellum
rs2509805	ENSG00000242689	*CNTF*	11	58390146	58393198	1	protein_coding	1270	CNTF	0.000349252	-0.109964842	48	1.60E-05	0.0192174	GTEx/v8/Brain_Cortex
rs2846139	ENSG00000162298	*SYVN1*	11	64889252	64902004	-1	protein_coding	84447	SYVN1	0.996650378	-0.305714367	2	1.72E-05	4.37E-02	GTEx/v8/Brain_Amygdala
rs2846139	ENSG00000245532	*NEAT1*	11	65190245	65213011	1	lincRNA	283131	NEAT1	NA	NA	38	2.16E-06	1.10E-19	GTEx/v8/Brain_Cerebellum:GTEx/v8/Brain_Frontal_Cortex_BA9:GTEx/v8/Brain_Nucleus_accumbens_basal_ganglia:GTEx/v8/Brain_Substantia_nigra
rs2846139	ENSG00000260233	*SSSCA1-AS1*	11	65337131	65337744	-1	antisense	254100	SSSCA1-AS1	NA	NA	4	1.92E-05	2.14E-02	GTEx/v8/Brain_Cerebellum

rs2846139	ENSG00000173338	*KCNK7*	11	65360326	65363467	-1	protein_coding	10089	KCNK7	6.42726E-06	0.176005115	2	4.01E-05	4.77E-03	GTEx/v8/Brain_Hypothalamus
rs2846139	ENSG00000214659	*KRT8P26*	11	65494410	65495685	1	pseudogene	390211	KRT8P26	NA	NA	2	8.21E-06	1.28071E-18	GTEx/v8/Brain_Cerebellum
rs612823	ENSG00000080854	*IGSF9B*	11	133778459	133826880	-1	protein_coding	22997	IGSF9B	0.999873394	NA	1	6.52E-09	5.01E-09	GTEx/v8/Brain_Cerebellum
rs7313797	ENSG00000151148	*UBE3B*	12	109915207	109974507	1	protein_coding	89910	UBE3B	0.009387706	0.786225851	38	1.23E-06	3.10E-05	GTEx/v8/Brain_Cerebellum
rs7313797	ENSG00000139428	*MMAB*	12	109991542	110011679	-1	protein_coding	326625	MMAB	3.88E-03	2.918192677	76	6.29E-09	6.67E-12	GTEx/v8/Brain_Caudate_basal_ganglia:GTEx/v8/Brain_Cerebellar_Hemisphere:GTEx/v8/Brain_Cerebellum:GTEx/v8/Brain_Cortex:GTEx/v8/Brain_Frontal_Cortex_BA9:GTEx/v8/Brain_Nucleus_accumbens_basal_ganglia
rs56080343	ENSG00000111707	*SUDS3*	12	118814185	118855840	1	protein_coding	64426	SUDS3	0.007596107	1.08228265	231	1.04E-06	5.33E-03	GTEx/v8/Brain_Cortex:GTEx/v8/Brain_Hippocampus:GTEx/v8/Brain_Nucleus_accumbens_basal_ganglia:GTEx/v8/Brain_Putamen_basal_ganglia
rs4772087	ENSG00000152767	*FARP1*	13	98794816	99102027	1	protein_coding	10160	FARP1	0.045263074	-0.444415571	50	4.83E-08	1.97E-04	GTEx/v8/Brain_Cerebellum
rs4772087	ENSG00000102572	*STK24*	13	99102455	99230194	-1	protein_coding	8428	STK24	0.797998297	0.417068263	50	2.01E-11	3.44636E-07	GTEx/v8/Brain_Cerebellar_Hemisphere:GTEx/v8/Brain_Cerebellum
rs2933195	ENSG00000139915	*MDGA2*	14	47308826	48144157	-1	protein_coding	161357	MDGA2	0.993216871	0.397758699	48	9.51E-06	1.99E-02	GTEx/v8/Brain_Caudate_basal_ganglia:GTEx/v8/Brain_Cerebellar_Hemisphere:GTEx/v8/Brain_Cortex:GTEx/v8/Brain_Nucleus_accumbens_basal_ganglia
rs8013071	ENSG00000246223	*C14orf64*	14	98391947	98444461	-1	protein_coding	388011	C14orf64	NA	NA	31	1.20E-07	9.09E-04	GTEx/v8/Brain_Caudate_basal_ganglia:GTEx/v8/Brain_Cerebellum
rs17514846	ENSG00000140564	*FURIN*	15	91411822	91426688	1	protein_coding	5045	FURIN	0.999933301	-0.156379719	1	2.51E-05	4.34E-02	GTEx/v8/Brain_Frontal_Cortex_BA9
rs2369818	ENSG00000248124	*RRN3P1*	16	21807951	21831731	-1	pseudogene	730092	RRN3P1	NA	NA	1	9.30E-05	1.95E-37	GTEx/v8/Brain_Cerebellum
rs2509805	ENSG00000265566	*RN7SL605P*	17	2657126	2657430	1	misc_RNA	106481087	RN7SL605P	NA	NA	1	1.06E-04	5.83E-09	GTEx/v8/Brain_Frontal_Cortex_BA9
rs3760569	ENSG00000141664	*ZCCHC2*	18	60190240	60254942	1	protein_coding	54877	ZCCHC2	0.305659357	-0.580417645	5	1.57E-05	0.0301536	GTEx/v8/Brain_Cerebellum
rs71327107	ENSG00000128285	*MCHR1*	22	41074754	41078818	1	protein_coding	2847	MCHR1	0.001148393	-0.081006493	6	1.89E-05	8.11E-10	GTEx/v8/Brain_Cerebellum
rs71327107	ENSG00000100372	*SLC25A17*	22	41165634	41215403	-1	protein_coding	10478	SLC25A17	2.42E-02	-0.779478694	41	6.86E-06	2.58E-05	GTEx/v8/Brain_Cerebellum:GTEx/v8/Brain_Nucleus_accumbens_basal_ganglia:GTEx/v8/Brain_Putamen_basal_ganglia
rs71327107	ENSG00000213857	*RP11-12M9.4*	22	41470184	41471243	-1	pseudogene	NA	NA	NA	NA	6	8.01E-05	0.0176512	GTEx/v8/Brain_Cerebellum
rs71327107	ENSG00000100393	*EP300*	22	41487790	41576081	1	protein_coding	2033	EP300	1.00E+00	NA	113	2.44E-06	0.00184947	GTEx/v8/Brain_Cerebellum:GTEx/v8/Brain_Hypothalamus
rs71327107	ENSG00000100395	*L3MBTL2*	22	41601209	41627275	1	protein_coding	83746	L3MBTL2	0.013028043	-0.618259224	120	1.80E-08	5.17512E-05	GTEx/v8/Brain_Anterior_cingulate_cortex_BA24:GTEx/v8/Brain_Frontal_Cortex_BA9
rs71327107	ENSG00000100401	*RANGAP1*	22	41641615	41682255	-1	protein_coding	5905	RANGAP1	0.13271872	-0.376521602	76	7.87E-07	1.39E-06	GTEx/v8/Brain_Cerebellar_Hemisphere:GTEx/v8/Brain_Cerebellum
rs71327107	ENSG00000100403	*ZC3H7B*	22	41697526	41756151	1	protein_coding	23264	ZC3H7B	0.990761551	-0.628157842	124	4.49E-08	1.03E-04	GTEx/v8/Brain_Cerebellum
rs71327107	ENSG00000100412	*ACO2*	22	41865129	41924993	1	protein_coding	50	ACO2	0.454377775	-0.617411984	21	2.91E-05	0.00290367	GTEx/v8/Brain_Cerebellum
rs71327107	ENSG00000100413	*POLR3H*	22	41921808	41940610	-1	protein_coding	171568	POLR3H	0.002007106	-1.305852274	120	2.19E-08	8.24E-14	GTEx/v8/Brain_Cerebellum:GTEx/v8/Brain_Cortex:GTEx/v8/Brain_Frontal_Cortex_BA9:GTEx/v8/Brain_Putamen_basal_ganglia
rs71327107	ENSG00000172346	*CSDC2*	22	41956767	41973745	1	protein_coding	27254	CSDC2	0.079803516	0.31935561	120	2.14E-09	6.71474E-18	GTEx/v8/Brain_Cortex:GTEx/v8/Brain_Frontal_Cortex_BA9
rs71327107	ENSG00000167077	*MEI1*	22	42095503	42195460	1	protein_coding	150365	MEI1	1.21592E-08	0.500155337	115	1.71E-06	1.60234E-05	GTEx/v8/Brain_Anterior_cingulate_cortex_BA24:GTEx/v8/Brain_Frontal_Cortex_BA9
rs71327107	ENSG00000159958	*TNFRSF13C*	22	42321045	42322822	-1	protein_coding	115650	TNFRSF13C	0.641054688	-0.063981155	31	1.87E-05	1.22E-11	GTEx/v8/Brain_Cerebellar_Hemisphere
rs71327107	ENSG00000205704	*LINC00634*	22	42348169	42354937	1	pseudogene	339674	LINC00634	NA	NA	9	6.19E-05	0.000116414	GTEx/v8/Brain_Frontal_Cortex_BA9
rs71327107	ENSG00000183066	*WBP2NL*	22	42394729	42454460	1	protein_coding	164684	WBP2NL	2.07233E-09	0.730692723	1	7.68E-05	1.03831E-06	GTEx/v8/Brain_Frontal_Cortex_BA9
rs71327107	ENSG00000198951	*NAGA*	22	42454358	42466846	-1	protein_coding	4668	NAGA	2.0199E-06	0.183467033	5	1.01E-04	7.04E-29	GTEx/v8/Brain_Cerebellum
rs71327107	ENSG00000100197	*CYP2D6*	22	42522501	42526908	-1	protein_coding	1565	CYP2D6	9.22646E-10	0.153953666	11	2.97107E-05	3.60661E-26	GTEx/v8/Brain_Cerebellum

Abbreviations: pLI, probability of loss of function intolerance; ncRVIS, non-coding residual variation intolerance score; eqtlMapSNPs, the number of SNPs mapped to the gene based on eQTL mapping; eqtlMapminP, mininum eQTL P value of mapped SNPs; eqtlMapminQ, mininum eQTL FDR of mapped SNPs; eQTL Mapts, tissue types of mapped eQTL SNPs (GTEx v8 Brain was selected).

Supplementary Table 18. Distinct genomic loci associated with irritability at condFDR<0.01 given association with each psychiatric disorder

a) Irritability given schizophrenia

Locusnum	CHR	Lead SNP	Lead BP	Mininum BP	Maximum BP	condFDR	Status
1	1	rs6679408	50591565	49463925	50591851	0.00147	additional loci
2	1	rs4295863	87903024	87889822	87962891	0.00266	additional loci
3	1	rs9440302	98492580	98327133	98562260	0.00338	additional loci
4	1	rs12757898	98873281	98861669	98913185	0.00611	additional loci
5	2	rs4953150	45157336	44905806	45170153	0.000338	replicated loci of irritability
6	2	rs12328506	48623184	48576661	48766078	0.00932	additional loci
7	2	rs2717032	58166760	57942987	58746775	0.000127	additional loci
8	2	rs58130738	122681246	122626234	122821817	0.00231	additional loci
9	2	rs12465778	144255201	144178400	144272138	0.00647	additional loci
10	2	rs6430085	146255869	146164102	146316319	0.000947	additional loci
11	2	rs11679484	198921604	198144539	198954774	6.13E-05	replicated loci of irritability
12	3	rs55930629	1836407	1820437	1854707	0.00768	additional loci
13	3	rs9882532	16865845	16843737	16879208	0.006	additional loci
14	3	rs12629637	49286797	48723302	49575913	0.000553	additional loci
15	3	rs683194	107299698	107181434	107379837	0.000227	additional loci
16	4	rs57980547	84600736	84600736	84615059	0.000677	additional loci
17	4	rs4607298	108877283	108853384	108885615	0.00914	additional loci
18	5	rs74994719	16327748	16327748	16327748	0.000922	additional loci
19	5	rs13166120	87948883	87936379	87988934	0.000109	replicated loci of irritability
20	5	rs17454114	107733615	107346393	107879748	4.91E-05	replicated loci of irritability
21	5	rs6579956	152078663	152075050	152153689	0.00154	additional loci
22	5	rs62382095	153534563	153526051	153534563	0.000977	additional loci
23	6	rs386229	57103934	57091166	57146935	0.00998	additional loci
24	6	rs62420383	98395749	98310091	98748005	0.00799	additional loci
25	7	rs62444881	2052318	1899447	2110850	0.00122	additional loci
26	7	rs3113277	69971824	69862423	70001442	0.000176	replicated loci of irritability
27	7	rs776472	114352862	114012911	114391519	0.00123	additional loci
28	7	rs62491417	139835245	139773656	139904406	0.00196	replicated loci of irritability

29	7	rs6464217	140691762	140665521	140791820	0.00428	replicated loci of irritability
30	8	rs1533059	8684953	8524474	8824858	0.00615	additional loci
31	8	rs2952176	10143553	10125535	11333353	1.71E-05	replicated loci of irritability
32	8	rs388391	16049254	16007617	16071273	0.000535	additional loci
33	8	rs9918736	77682553	77588548	77695732	0.00874	additional loci
34	8	rs16884419	89579649	89270547	89761163	2.29E-06	replicated loci of irritability
35	8	rs7835528	144243392	144238923	144270473	0.00315	additional loci
36	9	rs17487823	37252585	37044437	37375140	0.00208	additional loci
37	9	rs999483	135301389	135301389	135301389	9.21E-05	replicated loci of irritability
38	10	rs2225947	9671047	9651373	9963856	0.000812	additional loci
39	10	rs2804459	33676619	33676619	33689922	0.00555	additional loci
40	10	rs11191436	104637602	104585572	104963051	0.00211	additional loci
41	11	rs9633835	13345593	13288698	13350131	0.00716	additional loci
42	11	rs10767733	28642320	28591168	28694440	0.000308	additional loci
43	11	rs477424	88802281	88765779	88943549	0.00502	additional loci
44	12	rs2433634	23060363	23027722	23078449	0.00801	additional loci
45	12	rs2694685	79568343	79552449	79713434	0.00411	additional loci
46	12	rs4762536	99410114	99351017	99432798	0.00826	additional loci
47	12	rs73379978	103432987	103423736	103533984	0.0079	additional loci
48	12	rs12424599	109866615	109790342	110027795	0.00279	additional loci
49	12	rs3741475	117669914	117660827	117699505	0.00866	additional loci
50	13	rs3124426	55937346	55679499	56412644	0.000188	additional loci
51	13	rs73506872	66503729	66502640	66708760	0.0019	additional loci
52	13	rs2893380	111575426	111567531	111575426	0.00883	additional loci
53	13	rs6560933	114937294	114916023	114965548	0.00756	additional loci
54	14	rs187005436	42753172	42637396	43049277	0.00927	additional loci
55	14	rs4903249	75083881	75056894	75378185	0.00498	additional loci
56	14	rs941521	99708876	99700454	99751267	0.00445	additional loci
57	16	rs2054213	30971810	30566747	31109290	6.92E-05	replicated loci of irritability
58	17	rs78454137	43785096	43463493	44874453	1.93E-05	replicated loci of irritability
59	17	rs7207843	78485226	78463585	78590588	0.001198621	additional loci
60	18	rs74365659	26612082	26556001	26678827	0.000574736	replicated loci of irritability

61	18	rs34184697	42229293	42193553	42260348	0.008898465	additional loci
62	18	rs2958162	53083865	52765283	53463661	3.09E-06	replicated loci of irritability
63	18	rs2628207	63546386	63464943	63605950	0.000316243	replicated loci of irritability
64	19	rs7408912	8556363	8484796	8561287	0.009271051	additional loci
65	19	rs1972655	49650168	49646006	49654300	0.002298053	additional loci
66	20	rs13037664	33296988	32924967	33525407	3.13E-05	replicated loci of irritability
67	22	rs11913445	20142513	20049285	20153739	6.91E-05	additional loci
68	22	rs20551	41548008	41085969	41713111	0.00053501	additional loci
69	22	rs738184	48615721	48607014	48622651	0.003437312	additional loci

b) Irritability given bipolar I disorder

Locusnum	CHR	Lead SNP	Lead BP	Mininum BP	Maximum BP	condFDR	Status
1	1	rs4295863	87903024	87889822	87962891	0.000102	additional loci
2	2	rs17428810	32736043	32499150	32849224	0.00857	additional loci
3	2	rs4953150	45157336	44905806	45170153	0.000102	replicated loci of irritability
4	2	rs2678904	58137755	57942987	58484172	0.00561	additional loci
5	2	rs6718682	122663627	122626234	122821817	0.00916	additional loci
6	2	rs7569912	144168639	144144663	144272138	0.00207	additional loci
7	2	rs6430085	146255869	146164102	146316319	0.00566	additional loci
8	2	rs11679484	198921604	198393835	198954774	0.0017	replicated loci of irritability
9	3	rs7638154	49299375	48723302	49575913	0.0069	additional loci
10	3	rs6778895	85517387	85433248	85791383	0.000553	additional loci
11	3	rs600011	107295392	107181434	107379837	0.00033	additional loci
12	4	rs71596176	29381678	29344653	29430786	0.00617	additional loci
13	4	rs35733768	147560199	147474547	147589958	0.00731	additional loci
14	5	rs3814424	87968953	87936379	88010829	4.53E-05	replicated loci of irritability
15	5	rs17454114	107733615	107349092	107879748	0.00109	replicated loci of irritability
16	7	rs62444881	2052318	1899447	2110850	0.00147	additional loci
17	7	rs2158507	69862423	69832563	70001442	5.44E-05	replicated loci of irritability
18	7	rs1916977	113957306	113865735	113963334	0.00721	additional loci
19	7	rs12705984	114337615	114291435	114428727	0.00114	additional loci
20	7	rs62491417	139835245	139773656	139904406	0.000642	replicated loci of irritability

21	7	rs6464217	140691762	140424582	140791820	0.00582	replicated loci of irritability
22	8	rs1533059	8684953	8524474	8824858	0.00195	additional loci
23	8	rs2952176	10143553	10125535	11333353	1.52E-05	replicated loci of irritability
24	8	rs72673200	88963701	88937450	89030307	0.0083	replicated loci of irritability
25	8	rs16884419	89579649	89334910	89622646	0.000139	replicated loci of irritability
26	8	rs12675694	92736335	92464180	92768217	0.00896	additional loci
27	9	rs62533745	37078909	37045825	37406391	0.00501	additional loci
28	10	rs12570128	9957778	9651373	9963856	0.00383	additional loci
29	10	rs2804459	33676619	33676619	33689922	0.00445	additional loci
30	11	rs9633835	13345593	13248539	13350131	0.00119	additional loci
31	11	rs10767733	28642320	28591168	28694440	0.0097	additional loci
32	11	rs174529	61543961	61542006	61624181	0.00177	additional loci
33	11	rs477424	88802281	88188047	89058101	0.000112	additional loci
34	12	rs8181611	103437330	103423736	103533984	0.00412	additional loci
35	13	rs3105081	55954136	55679499	56412644	0.000152	additional loci
36	14	rs8019575	25319866	25274272	25324716	0.00992	additional loci
37	14	rs4903249	75083881	75056894	75113506	0.00537	additional loci
38	14	rs12886000	98501877	98444702	98501877	0.00723	additional loci
39	14	rs941521	99708876	99700454	99751267	0.00604	additional loci
40	16	rs2054213	30971810	30801183	31109290	9.33E-05	replicated loci of irritability
41	16	rs9926539	52528727	52515975	52529948	0.00642	additional loci
42	16	rs16967877	64825472	64746515	64884995	0.00986	additional loci
43	16	rs1424144	71349498	71278016	71376751	0.00725	additional loci
44	17	rs1358071	43803189	43460181	44865603	0.000267	replicated loci of irritability
45	17	rs7207843	78485226	78435577	78590588	0.001775049	additional loci
46	18	rs74365659	26612082	26260155	26678827	0.0001207	replicated loci of irritability
47	18	rs34184697	42229293	42193553	42260348	0.004975137	additional loci
48	18	rs4570961	52789439	52765283	53164693	5.96E-05	replicated loci of irritability
49	18	rs2086874	63545394	63464943	63616633	6.70E-05	replicated loci of irritability
50	20	rs6060009	33303974	32924967	33525407	3.93E-06	replicated loci of irritability
51	21	rs2836750	40289167	40289167	40316902	0.004223161	additional loci
52	22	rs9605069	20131115	20046042	20144796	7.09E-05	additional loci

53	22	rs20551	41548008	41408754	41713111	0.004693515	additional loci
54	22	rs738184	48615721	48607014	48622651	0.000325582	additional loci

c) Irritability given major depressive disorder

Locusnum	CHR	Lead SNP	Lead BP	Mininum BP	Maximum BP	condFDR	Status
1	1	rs114933496	33861277	33829731	33883883	0.005657539	additional loci
2	1	rs568832	37237239	37219429	37261085	0.00857083	additional loci
3	1	rs11211480	47693220	47648377	47708112	0.000425522	additional loci
4	1	rs6679408	50591565	49659375	50591851	0.002107821	additional loci
5	1	rs1993709	72838529	72628347	72959039	0.00408593	additional loci
6	1	rs4295863	87903024	87889822	87962891	0.000245261	additional loci
7	1	rs1198575	98562260	98468538	98562260	0.007379269	additional loci
8	2	rs4019431	32615493	32499150	32854668	0.001932265	additional loci
9	2	rs13402621	43458611	43450843	43578246	0.006656987	additional loci
10	2	rs4953150	45157336	44905806	45170153	1.62E-05	replicated loci of irritability
11	2	rs11682175	57987593	57943567	58065936	0.000241401	additional loci
12	2	rs115962846	58967058	58967058	59000612	0.007548618	additional loci
13	2	rs6718682	122663627	122626234	122821817	0.001997829	additional loci
14	2	rs12465778	144255201	144178400	144272138	9.07E-05	additional loci
15	2	rs6430085	146255869	146164102	146316319	0.007680621	additional loci
16	2	rs9287989	176717741	176717741	176727834	0.005983063	additional loci
17	2	rs11679484	198921604	198393835	198954774	0.003376721	replicated loci of irritability
18	3	rs2442824	9398637	9378654	9437723	0.001079759	additional loci
19	3	rs4308307	49193216	48723302	49575913	0.000118586	additional loci
20	3	rs2100142	71112560	71028756	71120801	0.009688205	additional loci
21	3	rs9851055	107295008	107243852	107295008	0.00666995	additional loci
22	4	rs10029973	15648085	15547333	15649079	0.003273486	additional loci
23	4	rs71596177	29382285	29344653	29430786	0.006450623	additional loci
24	4	rs12650957	41064317	40988780	41067204	0.001771328	additional loci
25	4	rs1880440	58198585	58191992	58242888	0.006827667	additional loci
26	4	rs57980547	84600736	84600736	84615059	0.000859951	additional loci
27	5	rs4048748	23918551	23725743	24006058	0.008862046	additional loci

28	5	rs11745652	62038924	62034795	62041461	0.00153598	additional loci
29	5	rs1301441	65070609	65015276	65080996	0.007926901	additional loci
30	5	rs13166120	87948883	87936379	87988934	1.03E-06	replicated loci of irritability
31	5	rs116509069	93181416	93021116	93503520	0.004291915	additional loci
32	5	rs30266	103972357	103783801	104082179	0.006095864	additional loci
33	5	rs17454114	107733615	107718510	107879748	4.88E-05	replicated loci of irritability
34	5	rs72795740	129346234	129111396	129433560	0.003955689	additional loci
35	5	rs4463213	139545748	139517197	139712550	0.00426164	additional loci
36	5	rs62382095	153534563	153526051	153534563	0.001138136	additional loci
37	5	rs67962168	165992220	165960451	166011041	0.003347548	additional loci
38	6	rs62404522	19307114	19212286	19348175	0.002313199	additional loci
39	6	rs9386123	100824140	100823521	101339400	0.003467795	additional loci
40	6	rs10499151	129431619	129430284	129554685	0.009718752	additional loci
41	6	rs9492590	130755667	130640605	130770858	0.003046796	additional loci
42	6	rs1334585	142976756	142868502	143011771	0.001900679	additional loci
43	6	rs844584	147964120	147946018	147987135	0.003224677	additional loci
44	7	rs62444881	2052318	1899447	2110850	0.000620857	additional loci
45	7	rs7805419	12282451	12233848	12286050	0.001617273	additional loci
46	7	rs2158507	69862423	69862423	69902503	0.000366208	replicated loci of irritability
47	7	rs2014265	114080336	114012911	114352394	0.000416277	additional loci
48	7	rs7778308	126676366	126665052	126784837	0.009755413	additional loci
49	7	rs62491417	139835245	139835245	139853106	0.000530888	replicated loci of irritability
50	7	rs6464217	140691762	140665521	140791820	0.002119477	replicated loci of irritability
51	8	rs2009619	10143943	10126348	10206921	1.60E-05	replicated loci of irritability
52	8	rs17830672	12675621	12662159	12709650	0.006297202	additional loci
53	8	rs16939357	77690563	77588716	77695732	0.001859229	additional loci
54	8	rs16884419	89579649	89334910	89622646	0.000193172	replicated loci of irritability
55	8	rs12675694	92736335	92464180	92768217	0.002552274	additional loci
56	8	rs78819497	131057877	131057877	131349715	0.003648653	additional loci
57	9	rs62533745	37078909	37044437	37406391	0.001854088	additional loci
58	9	rs10867251	81353326	81331339	81372519	0.004417151	additional loci
59	9	rs75259420	98215185	98191712	98314306	0.000590376	additional loci

60	9	rs2149351	120501644	120490567	120623641	0.000543767	additional loci
61	9	rs999483	135301389	135301389	135301389	1.91E-06	replicated loci of irritability
62	10	rs964036	9711702	9651373	9963730	0.001344082	additional loci
63	10	rs2399576	10887345	10840162	10910560	0.005101535	additional loci
64	10	rs7094793	12388925	12388488	12396699	0.007887212	additional loci
65	10	rs1976962	36219717	36208836	36239156	0.003010095	additional loci
66	10	rs7899434	68527738	68482665	68575278	0.008577029	additional loci
67	10	rs1909696	77582203	77537562	77626488	0.00762372	additional loci
68	11	rs1384015	13267489	13248539	13274553	0.004713048	additional loci
69	11	rs10767733	28642320	28591168	28694440	0.000203257	additional loci
70	11	rs2071754	31812582	31807524	31850105	0.000328404	additional loci
71	11	rs499188	57434122	57385856	57681828	0.009352344	additional loci
72	11	rs102275	61557803	61542006	61624181	0.001029881	additional loci
73	11	rs17829309	88271811	88188047	88318926	0.001849884	additional loci
74	11	rs477424	88802281	88765779	88943549	2.39E-05	additional loci
75	11	rs61687445	113438092	113166310	113451229	0.004590538	additional loci
76	11	rs612823	133834104	133834104	133834104	0.005742033	additional loci
77	11	rs7935549	134312341	134312341	134315499	0.005272571	additional loci
78	12	rs11109595	99163271	99162660	99163991	0.001961442	additional loci
79	12	rs8181611	103437330	103423736	103533984	0.003948551	additional loci
80	12	rs3742020	109883254	109790342	110027795	0.005342589	additional loci
81	12	rs3741475	117669914	117660827	117699505	0.002012387	additional loci
82	12	rs11068917	118791120	118586689	118888131	0.001307943	additional loci
83	12	rs4036462	125806709	125806709	125806709	0.008194834	additional loci
84	13	rs3124401	55975510	55679499	56250005	6.62E-05	additional loci
85	13	rs75969085	66584619	66502640	66724659	0.000218085	additional loci
86	13	rs9541796	69734919	69652435	69753336	0.003460212	additional loci
87	13	rs12427932	79275182	79251406	79361121	0.007920582	additional loci
88	13	rs7337667	97489457	97465425	97518070	0.00536093	additional loci
89	14	rs1782179	41693940	41616137	41801953	0.003715713	additional loci
90	14	rs67031627	57292728	57269825	57292728	0.005789785	additional loci
91	14	rs4903249	75083881	75056894	75113506	3.07E-05	additional loci

92	14	rs12886000	98501877	98444702	98501877	0.000715388	additional loci
93	14	rs942866	104014935	103987078	104093521	0.00169613	additional loci
94	15	rs921764	47512281	47488977	47610697	0.003790182	additional loci
95	15	rs8041518	87006916	86874161	87030799	0.001763311	additional loci
96	15	rs2584128	87979604	87910115	88029813	0.003027774	additional loci
97	16	rs11077022	6340010	6266886	6345984	0.001440673	additional loci
98	16	rs739710	18164698	17842728	18171046	0.001752754	additional loci
99	16	rs2054213	30971810	30801183	31109290	1.98E-05	replicated loci of irritability
100	16	rs11076488	49648893	49629325	49665164	0.007803002	additional loci
101	16	rs12325489	52307076	52232367	52317810	0.007905448	additional loci
102	16	rs1424144	71349498	71278016	71376751	0.002137532	additional loci
103	16	rs111504162	88864897	88849754	88864897	0.004168163	additional loci
104	17	rs11204421	19897805	19799698	20002824	0.003353519	additional loci
105	17	rs113173628	43794899	43463493	44793503	0.002708806	replicated loci of irritability
106	18	rs1941879	26604317	26556001	26678827	6.71E-05	replicated loci of irritability
107	18	rs4799463	35273433	35215517	35278126	0.006339552	additional loci
108	18	rs67973517	42238074	42193553	42260348	0.000551924	additional loci
109	18	rs10503002	53109202	52520149	53562185	3.70E-07	replicated loci of irritability
110	18	rs2628209	63546551	63464943	63603002	0.000294942	replicated loci of irritability
111	20	rs6078137	1185894	1171788	1192148	0.007951379	additional loci
112	20	rs13037664	33296988	33140999	33525407	2.57E-05	replicated loci of irritability
113	20	rs910187	45841052	45766642	45848043	0.006250486	additional loci
114	22	rs9605069	20131115	20046042	20144796	3.97E-05	additional loci
115	22	rs20551	41548008	41408754	41713111	0.000351743	additional loci
116	22	rs738184	48615721	48607014	48622651	0.000739185	additional loci

Abbreviations: CHR, Chromosome; BP, genomic position in human genome assembly; condFDR, conditional false discovery rate

Supplementary Table 19. eQTL mapping for distinct genomic loci from the condFDR results for irritability conditional on each psychiatric disorder

a) Irritability given schizophrenia

IndSigSNPs	ensg	symbol	chr	start	end	strand	type	entrezID	HUGO	pLI	ncRVIS	eqtlMapSNPs	eqtlMapminP	eqtlMapminQ	eqtlMapts
rs4953150	ENSG00000236502	*SIX3-AS1*	2	45167293	45169012	-1	antisense	100506108	SIX3-AS1	NA	NA	8	4.68E-07	4.76E-10	GTEx/v8/Brain_Caudate_basal_ganglia:GTEx/v8/Brain_Nucleus_accumbens_basal_ganglia
rs4953150	ENSG00000225156	*AC012354.6*	2	45181803	45195901	1	lincRNA	NA	NA	NA	NA	10	7.40E-08	9.59E-10	GTEx/v8/Brain_Caudate_basal_ganglia:GTEx/v8/Brain_Nucleus_accumbens_basal_ganglia
rs1232850 6	ENSG00000272663	*RP11-191L17.1*	2	48667182	48667736	-1	lincRNA	NA	NA	NA	NA	27	2.58E-08	1.15E-15	GTEx/v8/Brain_Caudate_basal_ganglia:GTEx/v8/Brain_Cortex:GTEx/v8/Brain_Putamen_basal_ganglia
rs12328506	ENSG00000162869	*PPP1R21*	2	48667737	48742525	1	protein_coding	129285	PPP1R21	0.0004021	0.571659952	35	5.65E-09	4.28E-14	GTEx/v8/Brain_Caudate_basal_ganglia:GTEx/v8/Brain_Cortex:GTEx/v8/Brain_Frontal_Cortex_BA9:GTEx/v8/Brain_Hippocampus:GTEx/v8/Brain_Hypothalamus:GTEx/v8/Brain_Nucleus_accumbens_basal_ganglia:GTEx/v8/Brain_Putamen_basal_ganglia:GTEx/v8/Brain_Spinal_cord_cervical_c-1:GTEx/v8/Brain_Substantia_nigra
rs12328506	ENSG00000243244	*STON1*	2	48756522	48826025	1	protein_coding	11037	STON1	4.45E-08	1.251076578	35	2.38E-07	2.82E-11	GTEx/v8/Brain_Anterior_cingulate_cortex_BA24:GTEx/v8/Brain_Cortex:GTEx/v8/Brain_Nucleus_accumbens_basal_ganglia:GTEx/v8/Brain_Putamen_basal_ganglia
rs12328506	ENSG00000068781	*STON1-GTF2A1L*	2	48757064	49003654	1	protein_coding	286749	STON1-GTF2A1L	1.65E-14	0.572863521	28	2.68E-07	4.03E-15	GTEx/v8/Brain_Cerebellum
rs12328506	ENSG00000242441	*GTF2A1L*	2	48844937	48960287	1	protein_coding	11036	GTF2A1L	5.65E-07	0.420928839	32	1.67E-09	4.48E-33	GTEx/v8/Brain_Cerebellum:GTEx/v8/Brain_Frontal_Cortex_BA9:GTEx/v8/Brain_Spinal_cord_cervical_c-1:GTEx/v8/Brain_Substantia_nigra
rs2717032	ENSG00000028116	*VRK2*	2	58134786	58387055	1	protein_coding	7444	VRK2	1.22E-08	-0.188348208	17	1.97E-07	0.00089661	GTEx/v8/Brain_Substantia_nigra
rs2717032	ENSG00000115392	*FANCL*	2	58386378	58468507	-1	protein_coding	55120	FANCL	2.10E-08	NA	19	2.64E-05	0.0327786	GTEx/v8/Brain_Cerebellum
rs9882532	ENSG00000224728	*AC090945.1*	3	15919554	15921078	1	pseudogene	NA	NA	NA	NA	1	9.61E-06	0.000396696	GTEx/v8/Brain_Cerebellar_Hemisphere
rs12629637	ENSG00000164045	*CDC25A*	3	48198636	48229892	-1	protein_coding	993	CDC25A	0.979698045	-0.724000674	31	0.000175411	1.73E-15	GTEx/v8/Brain_Cerebellar_Hemisphere
rs12629637	ENSG00000229759	*MRPS18AP1*	3	48297840	48298428	-1	pseudogene	359761	MRPS18AP1	NA	NA	66	0.000117392	1.29E-23	GTEx/v8/Brain_Cerebellum
rs12629637	ENSG00000232112	*TMA7*	3	48481667	48485616	1	protein_coding	51372	TMA7	0.010007308	0.321268329	6	5.52E-05	2.31E-15	GTEx/v8/Brain_Cerebellum
rs12629637	ENSG00000164053	*ATRIP*	3	48488114	48507115	1	protein_coding	84126	ATRIP	0.00148698	NA	1	6.29E-05	0.00252996	GTEx/v8/Brain_Putamen_basal_ganglia
rs12629637	ENSG00000213689	*TREX1*	3	48506445	48509044	1	protein_coding	11277	TREX1	0.093340719	-0.110327998	6	4.51E-05	2.14E-05	GTEx/v8/Brain_Caudate_basal_ganglia
rs12629637	ENSG00000145040	*UCN2*	3	48599160	48601206	-1	protein_coding	90226	UCN2	0.157235729	0.006326735	3	0.000114759	0.00573708	GTEx/v8/Brain_Cerebellum
rs12629637	ENSG00000213672	*NCKIPSD*	3	48701364	48723797	-1	protein_coding	51517	NCKIPSD	0.007131362	-0.551018211	174	1.59E-34	8.19E-28	GTEx/v8/Brain_Amygdala:GTEx/v8/Brain_Anterior_cingulate_cortex_BA24:GTEx/v8/Brain_Caudate_basal_ganglia:GTEx/v8/Brain_Cerebellar_Hemisphere:GTEx/v8/Brain_Cerebellum:GTEx/v8/Brain_Cortex:GTEx/v8/Brain_Frontal_Cortex_BA9:GTEx/v8/Brain_Hippocampus:GTEx/v8/Brain_Hypothalamus:GTEx/v8/Brain_Nucleus_accumbens_basal_ganglia:GTEx/v8/Brain_Putamen_basal_ganglia:GTEx/v8/Brain_Spinal_cord_cervical_c-1:GTEx/v8/Brain_Substantia_nigra
rs12629637	ENSG00000068745	*IP6K2*	3	48725436	48777786	-1	protein_coding	51447	IP6K2	0.989457703	-0.392798697	1	2.46E-08	1.48E-13	GTEx/v8/Brain_Cerebellar_Hemisphere:GTEx/v8/Brain_Cerebellum:GTEx/v8/Brain_Cortex
rs12629637	ENSG00000178467	*P4HTM*	3	49027319	49044587	1	protein_coding	54681	P4HTM	0.023274936	NA	174	1.50E-11	3.79E-08	GTEx/v8/Brain_Caudate_basal_ganglia:GTEx/v8/Brain_Cerebellar_Hemisphere:GTEx/v8/Brain_Cerebellum:GTEx/v8/Brain_Cortex:GTEx/v8/Brain_Frontal_Cortex_BA9:GTEx/v8/Brain_Hippocampus:GTEx/v8/Brain_Hypothalamus:GTEx/v8/Brain_Nucleus_accumbens_basal_ganglia:GTEx/v8/Brain_Putamen_basal_ganglia:GTEx/v8/Brain_Spinal_cord_cervical_c-1
rs12629637	ENSG00000178252	*WDR6*	3	49044495	49053386	1	protein_coding	11180	WDR6	9.20E-07	-0.058949543	174	2.04E-27	1.30E-21	GTEx/v8/Brain_Amygdala:GTEx/v8/Brain_Caudate_basal_ganglia:GTEx/v8/Brain_Cerebellar_Hemisphere:GTEx/v8/Brain_Cerebellum:GTEx/v8/Brain_Cortex:GTEx/v8/Brain_Frontal_Cortex_BA9:GTEx/v8/Brain_Hippocampus:GTEx/v8/Brain_Nucleus_accumbens_basal_ganglia:GTEx/v8/Brain_Putamen_basal_ganglia:GTEx/v8/Brain_Spinal_cord_cervical_c-1:GTEx/v8/Brain_Substantia_nigra
rs12629637	ENSG00000178149	*DALRD3*	3	49052921	49059726	-1	protein_coding	55152	DALRD3	1.85E-05	0.072306759	174	9.86E-11	1.79E-09	GTEx/v8/Brain_Anterior_cingulate_cortex_BA24:GTEx/v8/Brain_Cerebellar_Hemisphere:GTEx/v8/Brain_Cerebellum:GTEx/v8/Brain_Cortex:GTEx/v8/Brain_Frontal_Cortex_BA9
rs12629637	ENSG00000198218	*QRICH1*	3	49067140	49131796	-1	protein_coding	54870	QRICH1	0.996069748	-0.27929443	170	9.53E-11	3.56E-12	GTEx/v8/Brain_Caudate_basal_ganglia:GTEx/v8/Brain_Cerebellar_Hemisphere:GTEx/v8/Brain_Cerebellum:GTEx/v8/Brain_Cortex:GTEx/v8/Brain_Nucleus_accumbens_basal_ganglia
rs12629637	ENSG00000270441	*RP11-694I15.7*	3	49177519	49198284	-1	pseudogene	NA	NA	NA	NA	155	4.76E-06	0.000344885	GTEx/v8/Brain_Cerebellar_Hemisphere:GTEx/v8/Brain_Cerebellum:GTEx/v8/Brain_Cortex

rs12629637	ENSG00000177352	*CCDC71*	3	49199968	49203754	-1	protein_coding	64925	CCDC71	0.016989639	NA	174	9.42E-20	4.03E-15	GTEx/v8/Brain_Amygdala:GTEx/v8/Brain_Caudate_basal_ganglia:GTEx/v8/Brain_Cerebellar_Hemisphere:GTEx/v8/Brain_Cerebellum:GTEx/v8/Brain_Cortex:GTEx/v8/Brain_Frontal_Cortex_BA9:GTEx/v8/Brain_Hippocampus:GTEx/v8/Brain_Nucleus_accumbens_basal_ganglia:GTEx/v8/Brain_Putamen_basal_ganglia
rs12629637	ENSG00000185909	*KLHDC8B*	3	49209044	49213917	1	protein_coding	200942	KLHDC8B	0.01042142	-0.0773522225	167	1.55E-06	0.00138032	GTEx/v8/Brain_Cerebellum:GTEx/v8/Brain_Nucleus_accumbens_basal_ganglia
rs12629637	ENSG00000225399	*RP11-3B7.1*	3	49297518	49298744	1	protein_coding	NA	NA	NA	NA	32	7.14E-06	0.00866008	GTEx/v8/Brain_Anterior_cingulate_cortex_BA24
rs12629637	ENSG00000114316	*USP4*	3	49315264	49378145	-1	protein_coding	7375	USP4	6.20E-07	NA	6	0.000294146	0.0111109	GTEx/v8/Brain_Cerebellum
rs12629637	ENSG00000233276	*GPX1*	3	49394609	49396033	-1	protein_coding	2876	GPX1	0.006931651	-0.616399091	174	8.94E-15	5.90E-11	GTEx/v8/Brain_Anterior_cingulate_cortex_BA24:GTEx/v8/Brain_Caudate_basal_ganglia:GTEx/v8/Brain_Cerebellar_Hemisphere:GTEx/v8/Brain_Cerebellum:GTEx/v8/Brain_Cortex:GTEx/v8/Brain_Frontal_Cortex_BA9:GTEx/v8/Brain_Nucleus_accumbens_basal_ganglia:GTEx/v8/Brain_Putamen_basal_ganglia
rs12629637	ENSG00000145020	*AMT*	3	49454211	49460186	-1	protein_coding	275	AMT	0.000569475	-0.022637077	174	2.87E-31	7.74E-32	GTEx/v8/Brain_Anterior_cingulate_cortex_BA24:GTEx/v8/Brain_Caudate_basal_ganglia:GTEx/v8/Brain_Cerebellar_Hemisphere:GTEx/v8/Brain_Cerebellum:GTEx/v8/Brain_Cortex:GTEx/v8/Brain_Frontal_Cortex_BA9:GTEx/v8/Brain_Hippocampus:GTEx/v8/Brain_Hypothalamus:GTEx/v8/Brain_Nucleus_accumbens_basal_ganglia:GTEx/v8/Brain_Putamen_basal_ganglia:GTEx/v8/Brain_Spinal_cord_cervical_c-1:GTEx/v8/Brain_Substantia_nigra
rs12629637	ENSG00000145029	*NICN1*	3	49460379	49466759	-1	protein_coding	84276	NICN1	0.257245529	-1.087291051	169	8.31E-07	9.43E-05	GTEx/v8/Brain_Caudate_basal_ganglia:GTEx/v8/Brain_Cerebellum:GTEx/v8/Brain_Cortex:GTEx/v8/Brain_Frontal_Cortex_BA9:GTEx/v8/Brain_Nucleus_accumbens_basal_ganglia
rs12629637	ENSG00000226913	*BSN-AS2*	3	49586739	49591799	-1	lincRNA	100132677	BSN-AS2	NA	NA	174	9.29E-08	4.94E-06	GTEx/v8/Brain_Caudate_basal_ganglia:GTEx/v8/Brain_Cortex:GTEx/v8/Brain_Frontal_Cortex_BA9:GTEx/v8/Brain_Nucleus_accumbens_basal_ganglia:GTEx/v8/Brain_Putamen_basal_ganglia
rs12629637	ENSG00000173540	*GMPPB*	3	49754277	49761384	-1	protein_coding	29925	GMPPB	8.25E-05	-0.65955402	150	6.64E-11	1.80E-28	GTEx/v8/Brain_Amygdala:GTEx/v8/Brain_Anterior_cingulate_cortex_BA24:GTEx/v8/Brain_Cerebellar_Hemisphere:GTEx/v8/Brain_Cerebellum:GTEx/v8/Brain_Cortex:GTEx/v8/Brain_Frontal_Cortex_BA9:GTEx/v8/Brain_Hippocampus:GTEx/v8/Brain_Hypothalamus:GTEx/v8/Brain_Nucleus_accumbens_basal_ganglia:GTEx/v8/Brain_Putamen_basal_ganglia:GTEx/v8/Brain_Spinal_cord_cervical_c-1:GTEx/v8/Brain_Substantia_nigra
rs12629637	ENSG00000164078	*MST1R*	3	49924435	49941299	-1	protein_coding	4486	MST1R	1.69E-20	-0.377644955	8	8.42E-05	0.0236026	GTEx/v8/Brain_Hypothalamus
rs12629637	ENSG00000186792	*HYAL3*	3	50330262	50336899	-1	protein_coding	8372	HYAL3	1.52E-05	-0.353149956	10	1.44E-06	1.68E-09	GTEx/v8/Brain_Spinal_cord_cervical_c-1
rs4607298	ENSG00000155016	*CYP2U1*	4	108852525	108874613	1	protein_coding	113612	CYP2U1	0.081783015	-0.309381857	20	3.65E-10	1.77E-06	GTEx/v8/Brain_Cerebellar_Hemisphere:GTEx/v8/Brain_Cerebellum
rs13166120	ENSG00000250377	*CTC-467M3.3*	5	87988462	87989789	-1	lincRNA	NA	NA	NA	NA	24	1.36E-06	0.000243631	GTEx/v8/Brain_Anterior_cingulate_cortex_BA24:GTEx/v8/Brain_Cerebellar_Hemisphere:GTEx/v8/Brain_Cortex
rs6238209 5	ENSG00000164574	*GALNT10*	5	153570290	153800544	1	protein_coding	55568	GALNT10	0.00044926	1.469543542	2	5.65E-05	9.34E-08	GTEx/v8/Brain_Cerebellum
rs62444881	ENSG00000176349	*AC110781.3*	7	1878222	1889567	1	protein_coding	NA	NA	NA	NA	74	3.26E-07	2.61E-06	GTEx/v8/Brain_Nucleus_accumbens_basal_ganglia
rs62444881	ENSG00000122687	*FTSJ2*	7	2273866	2281840	-1	protein_coding	29960	FTSJ2	1.97E-05	0.096595649	95	1.66E-07	4.01E-12	GTEx/v8/Brain_Caudate_basal_ganglia:GTEx/v8/Brain_Cerebellar_Hemisphere:GTEx/v8/Brain_Cortex
rs62491417	ENSG00000006459	*KDM7A*	7	139784546	139876835	-1	protein_coding	80853	KDM7A	0.99869438	-1.580266986	2	1.06E-17	3.67E-14	GTEx/v8/Brain_Caudate_basal_ganglia:GTEx/v8/Brain_Cerebellar_Hemisphere:GTEx/v8/Brain_Cerebellum:GTEx/v8/Brain_Cortex:GTEx/v8/Brain_Frontal_Cortex_BA9:GTEx/v8/Brain_Hippocampus:GTEx/v8/Brain_Nucleus_accumbens_basal_ganglia:GTEx/v8/Brain_Putamen_basal_ganglia:GTEx/v8/Brain_Spinal_cord_cervical_c-1
rs6464217	ENSG00000157764	*BRAF*	7	140419127	140624564	-1	protein_coding	673	BRAF	0.999978196	NA	1	2.80E-05	0.0377877	GTEx/v8/Brain_Cerebellum
rs6464217	ENSG00000090263	*MRPS33*	7	140705854	140715028	-1	protein_coding	51650	MRPS33	0.00339571 8	0.268392925	51	2.30E-09	1.80E-05	GTEx/v8/Brain_Caudate_basal_ganglia:GTEx/v8/Brain_Cortex:GTEx/v8/Brain_Putamen_basal_ganglia
rs1533059	ENSG00000249188	*ENPP7P1*	8	8011830	8074438	1	pseudogene	100421823	ENPP7P1	NA	NA	111	5.37E-09	1.79E-10	GTEx/v8/Brain_Cerebellar_Hemisphere:GTEx/v8/Brain_Cerebellum
rs1533059	ENSG00000253893	*FAM85B*	8	8025341	8084136	-1	antisense	105379219	FAM85B	NA	NA	114	1.17E-19	1.81E-26	GTEx/v8/Brain_Amygdala:GTEx/v8/Brain_Anterior_cingulate_cortex_BA24:GTEx/v8/Brain_Caudate_basal_ganglia:GTEx/v8/Brain_Cerebellar_Hemisphere:GTEx/v8/Brain_Cerebellum:GTEx/v8/Brain_Cortex:GTEx/v8/Brain_Frontal_Cortex_BA9:GTEx/v8/Brain_Hippocampus:GTEx/v8/Brain_Hypothalamus:GTEx/v8/Brain_Nucleus_accumbens_basal_ganglia:GTEx/v8/Brain_Putamen_basal_ganglia:GTEx/v8/Brain_Spinal_cord_cervical_c-1:GTEx/v8/Brain_Substantia_nigra
rs1533059	ENSG00000173295	*FAM86B3P*	8	8086117	8102387	1	pseudogene	286042	FAM86B3P	NA	NA	114	6.14E-18	9.21E-28	GTEx/v8/Brain_Anterior_cingulate_cortex_BA24:GTEx/v8/Brain_Cerebellar_Hemisphere:GTEx/v8/Brain_Cerebellum:GTEx/v8/Brain_Cortex:GTEx/v8/Brain_Frontal_Cortex_BA9:GTEx/v8/Brain_Hippocampus
rs1533059	ENSG00000253981	*ALG1L13P*	8	8093525	8102189	-1	pseudogene	106479038	ALG1L13P	NA	NA	30	1.99E-06	1.69E-06	GTEx/v8/Brain_Cerebellar_Hemisphere:GTEx/v8/Brain_Cerebellum:GTEx/v8/Brain_Cortex
rs1533059	ENSG00000233609	*RP11-62H7.2*	8	8818710	8836535	1	pseudogene	NA	NA	NA	NA	114	8.42E-20	3.12E-15	GTEx/v8/Brain_Amygdala:GTEx/v8/Brain_Anterior_cingulate_cortex_BA24:GTEx/v8/Brain_Caudate_basal_ganglia:GTEx/v8/Brain_Cerebellar_Hemisphere:GTEx/v8/Brain_Cerebellum:GTEx/v8/Brain_Cortex:GTEx/v8/Brain_Frontal_Cortex_BA9:GTEx/v8/Brain_Hippocampus:GTEx/v8/Brain_Hypothalamus:GTEx/v8/Brain_Nucleus_accumbens_basal_ganglia:GTEx/v8/Brain_Putamen_basal_ganglia:GTEx/v8/Brain_Substantia_nigra
rs1533059	ENSG00000254340	*RP11-10A14.3*	8	8998934	9002945	1	antisense	NA	NA	NA	NA	1	1.64E-05	0.00123905	GTEx/v8/Brain_Cerebellum
rs2952176	ENSG00000261451	*RP11-981G7.1*	8	10291182	10295822	1	sense_overlapping	NA	NA	NA	NA	25	6.94E-07	6.07E-19	GTEx/v8/Brain_Cerebellar_Hemisphere:GTEx/v8/Brain_Cerebellum
rs388391	ENSG00000038945	*MSR1*	8	15965387	16424999	-1	protein_coding	4481	MSR1	8.21E-16	1.819933539	43	7.05E-07	0.00568105	GTEx/v8/Brain_Caudate_basal_ganglia:GTEx/v8/Brain_Hypothalamus:GTEx/v8/Brain_Nucleus_accumbens_basal_ganglia

rs16884419	ENSG00000156103	*MMP16*	8	89044237	89340254	-1	protein_coding	4325	MMP16	0.916974084	-3.43243289 4	95	1.36E-05	0.0179096	GTEx/v8/Brain_Cerebellum
rs7835528	ENSG00000176956	*LY6H*	8	144239331	144242128	-1	protein_coding	4062	LY6H	0.460605569	NA	27	2.41E-13	5.24E-09	GTEx/v8/Brain_Cerebellar_Hemisphere:GTEx/v8/Brain_Cerebellum:GTEx/v8/Brain_Cortex:GTEx/v8/Brain_Frontal_Cortex_BA9
rs7835528	ENSG00000253716	*RP13-582O9.5*	8	144362331	144363860	-1	antisense	100507316	MINCR	NA	NA	4	6.62E-06	5.76E-24	GTEx/v8/Brain_Cortex:GTEx/v8/Brain_Nucleus_accumbens_basal_ganglia:GTEx/v8/Brain_Putamen_basal_ganglia
rs7835528	ENSG00000178209	*PLEC*	8	144989321	145050902	-1	protein_coding	5339	PLEC	0.024936271	-0.44044438	14	3.83E-06	0.0165148	GTEx/v8/Brain_Hippocampus
rs11191436	ENSG00000269609	*RP11-18I14.10*	10	104209574	104220863	1	processed_transcript	1005005761	RPARP-AS1	NA	NA	182	1.74E-07	6.18E-17	GTEx/v8/Brain_Caudate_basal_ganglia:GTEx/v8/Brain_Frontal_Cortex_BA9:GTEx/v8/Brain_Hypothalamus:GTEx/v8/Brain_Nucleus_accumbens_basal_ganglia:GTEx/v8/Brain_Putamen_basal_ganglia
rs11191436	ENSG00000138111	*TMEM180*	10	104221149	104236802	1	protein_coding	79847	TMEM180	0.000178664	2.88274982 1	158	1.58E-05	1.06E-22	GTEx/v8/Brain_Caudate_basal_ganglia:GTEx/v8/Brain_Cerebellar_Hemisphere:GTEx/v8/Brain_Nucleus_accumbens_basal_ganglia:GTEx/v8/Brain_Putamen_basal_ganglia
rs11191436	ENSG00000138175	*ARL3*	10	104433488	104474164	-1	protein_coding	403	ARL3	0.716101007	-0.60002574 8	12	1.67E-05	3.19E-05	GTEx/v8/Brain_Cerebellum
rs11191436	ENSG00000156398	*SFXN2*	10	104474295	104503249	1	protein_coding	118980	SFXN2	0.001084574	-0.83701123 7	12	2.96E-05	3.55E-05	GTEx/v8/Brain_Cerebellum:GTEx/v8/Brain_Cortex
rs11191436	ENSG00000148795	*CYP17A1*	10	104590288	104597290	-1	protein_coding	1586	CYP17A1	0.035030608	0.08668197 7	12	2.44E-05	0.0391891	GTEx/v8/Brain_Frontal_Cortex_BA9
rs11191436	ENSG00000166275	*C10orf32*	10	104613980	104624718	1	protein_coding	119032	C10orf32	0.031637861	0.49035821 1	182	7.87E-33	5.95E-26	GTEx/v8/Brain_Amygdala:GTEx/v8/Brain_Anterior_cingulate_cortex_BA24:GTEx/v8/Brain_Caudate_basal_ganglia:GTEx/v8/Brain_Cerebellar_Hemisphere:GTEx/v8/Brain_Cerebellum:GTEx/v8/Brain_Cortex:GTEx/v8/Brain_Frontal_Cortex_BA9:GTEx/v8/Brain_Hippocampus:GTEx/v8/Brain_Hypothalamus:GTEx/v8/Brain_Nucleus_accumbens_basal_ganglia:GTEx/v8/Brain_Putamen_basal_ganglia:GTEx/v8/Brain_Spinal_cord_cervical_c-1:GTEx/v8/Brain_Substantia_nigra
rs11191436	ENSG00000214435	*AS3MT*	10	104629273	104661656	1	protein_coding	57412	AS3MT	5.09E-07	0.72688117 8	182	3.61E-35	6.43E-34	GTEx/v8/Brain_Amygdala:GTEx/v8/Brain_Anterior_cingulate_cortex_BA24:GTEx/v8/Brain_Caudate_basal_ganglia:GTEx/v8/Brain_Cerebellar_Hemisphere:GTEx/v8/Brain_Cerebellum:GTEx/v8/Brain_Cortex:GTEx/v8/Brain_Frontal_Cortex_BA9:GTEx/v8/Brain_Hippocampus:GTEx/v8/Brain_Hypothalamus:GTEx/v8/Brain_Nucleus_accumbens_basal_ganglia:GTEx/v8/Brain_Putamen_basal_ganglia:GTEx/v8/Brain_Spinal_cord_cervical_c-1:GTEx/v8/Brain_Substantia_nigra
rs11191436	ENSG00000235266	*RP11-753C18.8*	10	104647631	104647945	-1	pseudogene	NA	NA	NA	NA	182	8.05E-14	1.79E-11	GTEx/v8/Brain_Cerebellar_Hemisphere:GTEx/v8/Brain_Cerebellum
rs11191436	ENSG00000272912	*RP11-724N1.1*	10	104674342	104675161	1	lincRNA	NA	NA	NA	NA	182	2.13E-16	3.13E-16	GTEx/v8/Brain_Cerebellar_Hemisphere:GTEx/v8/Brain_Cerebellum:GTEx/v8/Brain_Hypothalamus
rs11191436	ENSG00000076685	*NT5C2*	10	104845940	104953056	-1	protein_coding	22978	NT5C2	0.052197863	0.04832858	171	3.99E-08	2.15E-07	GTEx/v8/Brain_Cerebellar_Hemisphere:GTEx/v8/Brain_Cerebellum:GTEx/v8/Brain_Cortex
rs11191436	ENSG00000148843	*PDCD11*	10	105156405	105206049	1	protein_coding	22984	PDCD11	0.468448698	-0.80537843 1	7	1.06E-05	5.99E-06	GTEx/v8/Brain_Cerebellum
rs12424599	ENSG00000139428	*MMAB*	12	109991542	110011679	-1	protein_coding	326625	MMAB	0.00387716	2.91819267 7	6	7.13E-06	3.85E-05	GTEx/v8/Brain_Caudate_basal_ganglia:GTEx/v8/Brain_Frontal_Cortex_BA9
rs2893380	ENSG00000255874	*LINC00346*	13	111521578	111522162	-1	protein_coding	283487	LINC00346	NA	NA	11	8.98E-07	0.000106238	GTEx/v8/Brain_Cerebellar_Hemisphere:GTEx/v8/Brain_Nucleus_accumbens_basal_ganglia:GTEx/v8/Brain_Substantia_nigra
rs6560933	ENSG00000185989	*RASA3*	13	114747194	114898086	-1	protein_coding	22821	RASA3	0.000433959	0.06121724 5	47	9.46E-10	2.59E-08	GTEx/v8/Brain_Cerebellar_Hemisphere:GTEx/v8/Brain_Cerebellum:GTEx/v8/Brain_Cortex
rs6560933	ENSG00000169062	*UPF3A*	13	115047059	115071283	1	protein_coding	65110	UPF3A	0.000813153	0.18912020 4	3	3.36E-05	0.00632297	GTEx/v8/Brain_Spinal_cord_cervical_c-1
rs4903249	ENSG00000119682	*AREL1*	14	75120140	75179818	-1	protein_coding	9870	AREL1	0.011802084	-0.56009483 9	14	7.29E-07	1.08E-05	GTEx/v8/Brain_Frontal_Cortex_BA9
rs2054213	ENSG00000196118	*C16orf93*	16	30768744	30774031	-1	protein_coding	90835	C16orf93	8.00E-05	-0.75431882 2	102	1.59E-13	2.99E-15	GTEx/v8/Brain_Caudate_basal_ganglia:GTEx/v8/Brain_Cortex:GTEx/v8/Brain_Frontal_Cortex_BA9:GTEx/v8/Brain_Hippocampus:GTEx/v8/Brain_Hypothalamus:GTEx/v8/Brain_Nucleus_accumbens_basal_ganglia
rs2054213	ENSG00000103549	*RNF40*	16	30773066	30787628	1	protein_coding	9810	RNF40	0.999994909	-1.91378361 4	25	4.02E-09	1.74E-05	GTEx/v8/Brain_Caudate_basal_ganglia:GTEx/v8/Brain_Cerebellar_Hemisphere:GTEx/v8/Brain_Cerebellum:GTEx/v8/Brain_Nucleus_accumbens_basal_ganglia:GTEx/v8/Brain_Putamen_basal_ganglia
rs2054213	ENSG00000099381	*SETD1A*	16	30968615	30996437	1	protein_coding	9739	SETD1A	0.999996231	-0.89496931 3	57	3.17E-06	0.00390586	GTEx/v8/Brain_Cerebellum
rs2054213	ENSG00000099377	*HSD3B7*	16	30996519	31000473	1	protein_coding	80270	HSD3B7	4.64E-06	-0.86741698 7	94	1.96E-06	8.50E-05	GTEx/v8/Brain_Anterior_cingulate_cortex_BA24:GTEx/v8/Brain_Cortex:GTEx/v8/Brain_Hypothalamus
rs2054213	ENSG00000099365	*STX1B*	16	31000577	31021949	-1	protein_coding	112755	STX1B	0.944474733	-1.61045008 6	106	5.01E-07	0.000791194	GTEx/v8/Brain_Cerebellar_Hemisphere:GTEx/v8/Brain_Cerebellum
rs2054213	ENSG00000103496	*STX4*	16	31044210	31054296	1	protein_coding	6810	STX4	9.66E-05	-0.42186763 5	137	1.18E-12	1.78E-08	GTEx/v8/Brain_Anterior_cingulate_cortex_BA24:GTEx/v8/Brain_Cerebellum:GTEx/v8/Brain_Cortex:GTEx/v8/Brain_Frontal_Cortex_BA9:GTEx/v8/Brain_Hypothalamus:GTEx/v8/Brain_Nucleus_accumbens_basal_ganglia

rs2054213	ENSG00000260911	*RP11-196G11.2*	16	31054471	31061201	1	lincRNA	NA	NA	NA	NA	137	1.45E-26	3.33E-21	GTEx/v8/Brain_Anterior_cingulate_cortex_BA24:GTEx/v8/Brain_Caudate_basal_ganglia:GTEx/v8/Brain_Cerebellar_Hemisphere:GTEx/v8/Brain_Cerebellum:GTEx/v8/Brain_Cortex:GTEx/v8/Brain_Frontal_Cortex_BA9:GTEx/v8/Brain_Hippocampus:GTEx/v8/Brain_Nucleus_accumbens_basal_ganglia:GTEx/v8/Brain_Putamen_basal_ganglia:GTEx/v8/Brain_Spinal_cord_cervical_c-1:GTEx/v8/Brain_Substantia_nigra
rs2054213	ENSG00000167395	*ZNF646*	16	31085743	31095517	1	protein_coding	9726	ZNF646	0.00556536	0.56456394 2	75	1.54E-06	0.00486533	GTEx/v8/Brain_Caudate_basal_ganglia:GTEx/v8/Brain_Hippocampus
rs2054213	ENSG00000167397	*VKORC1*	16	31102163	31107301	-1	protein_coding	79001	VKORC1	0.081803076	-0.611112737	92	1.23E-06	0.000396364	GTEx/v8/Brain_Caudate_basal_ganglia:GTEx/v8/Brain_Cortex
rs2054213	ENSG00000103510	*KAT8*	16	31127075	31142714	1	protein_coding	84148	KAT8	0.912576388	0.037392946	137	1.20E-12	3.51E-17	GTEx/v8/Brain_Caudate_basal_ganglia:GTEx/v8/Brain_Cerebellar_Hemisphere:GTEx/v8/Brain_Cerebellum:GTEx/v8/Brain_Cortex:GTEx/v8/Brain_Frontal_Cortex_BA9:GTEx/v8/Brain_Hippocampus:GTEx/v8/Brain_Hypothalamus:GTEx/v8/Brain_Nucleus_accumbens_basal_ganglia:GTEx/v8/Brain_Putamen_basal_ganglia
rs2054213	ENSG00000262766	*RP11-196G11.4*	16	31129399	31130068	1	sense_intronic	NA	NA	NA	NA	12	2.78E-05	0.000263052	GTEx/v8/Brain_Cerebellum
rs2054213	ENSG00000178226	*PRSS36*	16	31150246	31161415	-1	protein_coding	146547	PRSS36	4.42E-13	-0.151362733	126	1.72E-11	1.01E-22	GTEx/v8/Brain_Amygdala:GTEx/v8/Brain_Anterior_cingulate_cortex_BA24:GTEx/v8/Brain_Caudate_basal_ganglia:GTEx/v8/Brain_Cerebellar_Hemisphere:GTEx/v8/Brain_Cerebellum:GTEx/v8/Brain_Cortex:GTEx/v8/Brain_Frontal_Cortex_BA9:GTEx/v8/Brain_Hippocampus:GTEx/v8/Brain_Hypothalamus:GTEx/v8/Brain_Nucleus_accumbens_basal_ganglia:GTEx/v8/Brain_Putamen_basal_ganglia
rs7845413 7	ENSG00000136448	*NMT1*	17	43128978	43186384	1	protein_coding	4836	NMT1	0.998500521	0.032447683	1801	9.33E-06	2.39E-07	GTEx/v8/Brain_Cerebellar_Hemisphere:GTEx/v8/Brain_Cerebellum:GTEx/v8/Brain_Spinal_cord_cervical_c-1
rs78454137	ENSG00000267121	*CTD-2020K17.1*	17	43268298	43299589	-1	antisense	339192	LOC339192	NA	NA	2667	6.12E-11	4.50E-07	GTEx/v8/Brain_Cerebellar_Hemisphere:GTEx/v8/Brain_Cerebellum
rs78454137	ENSG00000184922	*FMNL1*	17	43298811	43324687	1	protein_coding	752	FMNL1	0.999687036	NA	2667	1.02E-24	7.89E-19	GTEx/v8/Brain_Cerebellar_Hemisphere:GTEx/v8/Brain_Cerebellum
rs78454137	ENSG00000233175	*CTD-2020K17.3*	17	43315395	43319101	-1	antisense	107985040	LOC107985040	NA	NA	21	9.72E-05	2.69E-16	GTEx/v8/Brain_Cerebellum
rs78454137	ENSG00000006062	*MAP3K14*	17	43340488	43394414	-1	processed_transcript	9020	MAP3K14	NA	NA	2	0.000176915	0.00353334	GTEx/v8/Brain_Cerebellum
rs78454137	ENSG00000159314	*ARHGAP27*	17	43471275	43511787	-1	protein_coding	201176	ARHGAP27	0.001255844	-0.761627793	2804	4.59E-19	7.23E-14	GTEx/v8/Brain_Caudate_basal_ganglia:GTEx/v8/Brain_Cerebellar_Hemisphere:GTEx/v8/Brain_Cerebellum:GTEx/v8/Brain_Cortex:GTEx/v8/Brain_Nucleus_accumbens_basal_ganglia:GTEx/v8/Brain_Putamen_basal_ganglia
rs78454137	ENSG00000225190	*PLEKHM1*	17	43513266	43568115	-1	protein_coding	9842	PLEKHM1	0.84054321	-0.663068032	2804	1.98E-42	8.80E-35	GTEx/v8/Brain_Amygdala:GTEx/v8/Brain_Anterior_cingulate_cortex_BA24:GTEx/v8/Brain_Caudate_basal_ganglia:GTEx/v8/Brain_Cerebellar_Hemisphere:GTEx/v8/Brain_Cerebellum:GTEx/v8/Brain_Cortex:GTEx/v8/Brain_Frontal_Cortex_BA9:GTEx/v8/Brain_Hypothalamus:GTEx/v8/Brain_Nucleus_accumbens_basal_ganglia:GTEx/v8/Brain_Putamen_basal_ganglia
rs78454137	ENSG00000236234	*AC091132.1*	17	43530210	43541431	1	antisense	NA	NA	NA	NA	2804	2.17E-12	2.29E-08	GTEx/v8/Brain_Cerebellar_Hemisphere:GTEx/v8/Brain_Cerebellum
rs78454137	ENSG00000214425	*LRRC37A4P*	17	43578685	43627701	-1	pseudogene	55073	LRRC37A4P	NA	NA	2806	1.81E-58	2.69E-48	GTEx/v8/Brain_Amygdala:GTEx/v8/Brain_Anterior_cingulate_cortex_BA24:GTEx/v8/Brain_Caudate_basal_ganglia:GTEx/v8/Brain_Cerebellar_Hemisphere:GTEx/v8/Brain_Cerebellum:GTEx/v8/Brain_Cortex:GTEx/v8/Brain_Frontal_Cortex_BA9:GTEx/v8/Brain_Hippocampus:GTEx/v8/Brain_Hypothalamus:GTEx/v8/Brain_Nucleus_accumbens_basal_ganglia:GTEx/v8/Brain_Putamen_basal_ganglia:GTEx/v8/Brain_Spinal_cord_cervical_c-1:GTEx/v8/Brain_Substantia_nigra
rs78454137	ENSG00000266918	*RP11-798G7.8*	17	43608943	43611204	1	lincRNA	NA	NA	NA	NA	2804	2.14E-16	6.68E-12	GTEx/v8/Brain_Amygdala:GTEx/v8/Brain_Caudate_basal_ganglia:GTEx/v8/Brain_Cerebellar_Hemisphere:GTEx/v8/Brain_Cerebellum:GTEx/v8/Brain_Cortex
rs78454137	ENSG00000267198	*RP11-798G7.6*	17	43623170	43640596	1	lincRNA	NA	NA	NA	NA	2606	5.49E-06	1.37E-08	GTEx/v8/Brain_Cerebellum
rs78454137	ENSG00000264070	*DND1P1*	17	43663237	43664295	1	pseudogene	644157	DND1P1	NA	NA	2805	7.80E-38	1.42E-31	GTEx/v8/Brain_Amygdala:GTEx/v8/Brain_Anterior_cingulate_cortex_BA24:GTEx/v8/Brain_Caudate_basal_ganglia:GTEx/v8/Brain_Cerebellar_Hemisphere:GTEx/v8/Brain_Cerebellum:GTEx/v8/Brain_Cortex:GTEx/v8/Brain_Frontal_Cortex_BA9:GTEx/v8/Brain_Hippocampus:GTEx/v8/Brain_Hypothalamus:GTEx/v8/Brain_Nucleus_accumbens_basal_ganglia:GTEx/v8/Brain_Putamen_basal_ganglia:GTEx/v8/Brain_Spinal_cord_cervical_c-1:GTEx/v8/Brain_Substantia_nigra
rs78454137	ENSG00000263503	*RP11-707O23.5*	17	43678235	43679706	-1	pseudogene	NA	NA	NA	NA	2805	1.33E-41	1.26E-35	GTEx/v8/Brain_Amygdala:GTEx/v8/Brain_Anterior_cingulate_cortex_BA24:GTEx/v8/Brain_Caudate_basal_ganglia:GTEx/v8/Brain_Cerebellar_Hemisphere:GTEx/v8/Brain_Cerebellum:GTEx/v8/Brain_Cortex:GTEx/v8/Brain_Frontal_Cortex_BA9:GTEx/v8/Brain_Hippocampus:GTEx/v8/Brain_Hypothalamus:GTEx/v8/Brain_Nucleus_accumbens_basal_ganglia:GTEx/v8/Brain_Putamen_basal_ganglia:GTEx/v8/Brain_Spinal_cord_cervical_c-1:GTEx/v8/Brain_Substantia_nigra
rs78454137	ENSG00000204650	*CRHR1-IT1*	17	43697694	43725582	1	pseudogene	147081	CRHR1-IT1	NA	NA	2806	1.06E-38	1.09E-30	GTEx/v8/Brain_Amygdala:GTEx/v8/Brain_Anterior_cingulate_cortex_BA24:GTEx/v8/Brain_Caudate_basal_ganglia:GTEx/v8/Brain_Cerebellar_Hemisphere:GTEx/v8/Brain_Cerebellum:GTEx/v8/Brain_Cortex:GTEx/v8/Brain_Frontal_Cortex_BA9:GTEx/v8/Brain_Hippocampus:GTEx/v8/Brain_Hypothalamus:GTEx/v8/Brain_Nucleus_accumbens_basal_ganglia:GTEx/v8/Brain_Putamen_basal_ganglia:GTEx/v8/Brain_Spinal_cord_cervical_c-1:GTEx/v8/Brain_Substantia_nigra
rs78454137	ENSG00000120088	*CRHR1*	17	43699267	43913194	1	protein_coding	1394	CRHR1	0.792134286	2.038085631	2784	4.29E-06	0.00117547	GTEx/v8/Brain_Caudate_basal_ganglia:GTEx/v8/Brain_Cortex:GTEx/v8/Brain_Hippocampus:GTEx/v8/Brain_Nucleus_accumbens_basal_ganglia:GTEx/v8/Brain_Putamen_basal_ganglia
rs78454137	ENSG00000264589	*MAPT-AS1*	17	43921017	43972966	-1	antisense	100128977	MAPT-AS1	NA	NA	2810	1.07E-21	3.37E-16	GTEx/v8/Brain_Caudate_basal_ganglia:GTEx/v8/Brain_Cerebellar_Hemisphere:GTEx/v8/Brain_Cerebellum:GTEx/v8/Brain_Hippocampus:GTEx/v8/Brain_Hypothalamus:GTEx/v8/Brain_Nucleus_accumbens_basal_ganglia:GTEx/v8/Brain_Putamen_basal_ganglia:GTEx/v8/Brain_Spinal_cord_cervical_c-1:GTEx/v8/Brain_Substantia_nigra
rs78454137	ENSG00000185294	*SPPL2C*	17	43922256	43924438	1	protein_coding	162540	SPPL2C	2.30E-05	0.187394842	2810	6.65E-25	5.44E-19	GTEx/v8/Brain_Cerebellar_Hemisphere:GTEx/v8/Brain_Cerebellum:GTEx/v8/Brain_Cortex:GTEx/v8/Brain_Frontal_Cortex_BA9
rs78454137	ENSG00000186868	*MAPT*	17	43971748	44105700	1	protein_coding	4137	MAPT	4.68E-05	2.01024275	2799	1.10E-07	7.42E-05	GTEx/v8/Brain_Caudate_basal_ganglia:GTEx/v8/Brain_Cerebellar_Hemisphere:GTEx/v8/Brain_Cerebellum
rs78454137	ENSG00000120071	*KANSL1*	17	44107282	44302733	-1	protein_coding	284058	KANSL1	0.999733506	0.058945245	2804	1.32E-06	1.09E-09	GTEx/v8/Brain_Caudate_basal_ganglia:GTEx/v8/Brain_Cerebellar_Hemisphere:GTEx/v8/Brain_Cerebellum:GTEx/v8/Brain_Frontal_Cortex_BA9
rs78454137	ENSG00000214401	*KANSL1-AS1*	17	44270942	44274089	1	antisense	644246	KANSL1-AS1	NA	NA	2810	4.51E-52	1.22E-43	GTEx/v8/Brain_Amygdala:GTEx/v8/Brain_Anterior_cingulate_cortex_BA24:GTEx/v8/Brain_Caudate_basal_ganglia:GTEx/v8/Brain_Cerebellar_Hemisphere:GTEx/v8/Brain_Cerebellum:GTEx/v8/Brain_Cortex:GTEx/v8/Brain_Frontal_Cortex_BA9:GTEx/v8/Brain_Hippocampus:GTEx/v8/Brain_Hypothalamus:GTEx/v8/Brain_Nucleus_accumbens_basal_ganglia:GTEx/v8/Brain_Putamen_basal_ganglia:GTEx/v8/Brain_Spinal_cord_cervical_c-1:GTEx/v8/Brain_Substantia_nigra
rs78454137	ENSG00000262500	*RP11-259G18.2*	17	44320972	44322410	1	pseudogene	NA	NA	NA	NA	2810	2.61E-40	5.94E-34	GTEx/v8/Brain_Amygdala:GTEx/v8/Brain_Anterior_cingulate_cortex_BA24:GTEx/v8/Brain_Caudate_basal_ganglia:GTEx/v8/Brain_Cerebellar_Hemisphere:GTEx/v8/Brain_Cerebellum:GTEx/v8/Brain_Cortex:GTEx/v8/Brain_Frontal_Cortex_BA9:GTEx/v8/Brain_Hippocampus:GTEx/v8/Brain_Hypothalamus:GTEx/v8/Brain_Nucleus_accumbens_basal_ganglia:GTEx/v8/Brain_Putamen_basal_ganglia:GTEx/v8/Brain_Spinal_cord_cervical_c-1:GTEx/v8/Brain_Substantia_nigra
rs78454137	ENSG00000262539	*RP11-259G18.3*	17	44336917	44337972	-1	pseudogene	NA	NA	NA	NA	2811	3.51E-45	1.60E-37	GTEx/v8/Brain_Amygdala:GTEx/v8/Brain_Anterior_cingulate_cortex_BA24:GTEx/v8/Brain_Caudate_basal_ganglia:GTEx/v8/Brain_Cerebellar_Hemisphere:GTEx/v8/Brain_Cerebellum:GTEx/v8/Brain_Cortex:GTEx/v8/Brain_Frontal_Cortex_BA9:GTEx/v8/Brain_Hippocampus:GTEx/v8/Brain_Hypothalamus:GTEx/v8/Brain_Nucleus_accumbens_basal_ganglia:GTEx/v8/Brain_Putamen_basal_ganglia:GTEx/v8/Brain_Spinal_cord_cervical_c-1:GTEx/v8/Brain_Substantia_nigra
rs78454137	ENSG00000261575	*RP11-259G18.1*	17	44344403	44346060	1	pseudogene	NA	NA	NA	NA	2811	7.46E-51	1.13E-41	GTEx/v8/Brain_Amygdala:GTEx/v8/Brain_Anterior_cingulate_cortex_BA24:GTEx/v8/Brain_Caudate_basal_ganglia:GTEx/v8/Brain_Cerebellar_Hemisphere:GTEx/v8/Brain_Cerebellum:GTEx/v8/Brain_Cortex:GTEx/v8/Brain_Frontal_Cortex_BA9:GTEx/v8/Brain_Hippocampus:GTEx/v8/Brain_Hypothalamus:GTEx/v8/Brain_Nucleus_accumbens_basal_ganglia:GTEx/v8/Brain_Putamen_basal_ganglia:GTEx/v8/Brain_Spinal_cord_cervical_c-1:GTEx/v8/Brain_Substantia_nigra

rs78454137	ENSG00000228696	*ARL17B*	17	44352150	44439130	-1	protein_coding	100506084	ARL17B	NA	-0.428243465	4	8.50E-08	2.36E-17	GTEx/v8/Brain_Cerebellar_Hemisphere:GTEx/v8/Brain_Cerebellum:GTEx/v8/Brain_Hypothalamus
rs78454137	ENSG00000176681	*LRRC37A*	17	44370099	44415160	1	protein_coding	9884	LRRC37A	0.890487356	NA	2810	1.21E-30	4.87E-42	GTEx/v8/Brain_Amygdala:GTEx/v8/Brain_Anterior_cingulate_cortex_BA24:GTEx/v8/Brain_Caudate_basal_ganglia:GTEx/v8/Brain_Cerebellar_Hemisphere:GTEx/v8/Brain_Cerebellum:GTEx/v8/Brain_Cortex:GTEx/v8/Brain_Frontal_Cortex_BA9:GTEx/v8/Brain_Hippocampus:GTEx/v8/Brain_Hypothalamus:GTEx/v8/Brain_Nucleus_accumbens_basal_ganglia:GTEx/v8/Brain_Putamen_basal_ganglia:GTEx/v8/Brain_Spinal_cord_cervical_c-1:GTEx/v8/Brain_Substantia_nigra
rs78454137	ENSG00000238083	*LRRC37A2*	17	44588877	44633016	1	protein_coding	474170	LRRC37A2	NA	NA	2664	2.68E-52	7.91E-44	GTEx/v8/Brain_Amygdala:GTEx/v8/Brain_Anterior_cingulate_cortex_BA24:GTEx/v8/Brain_Caudate_basal_ganglia:GTEx/v8/Brain_Cerebellar_Hemisphere:GTEx/v8/Brain_Cerebellum:GTEx/v8/Brain_Cortex:GTEx/v8/Brain_Frontal_Cortex_BA9:GTEx/v8/Brain_Hippocampus:GTEx/v8/Brain_Hypothalamus:GTEx/v8/Brain_Nucleus_accumbens_basal_ganglia:GTEx/v8/Brain_Putamen_basal_ganglia:GTEx/v8/Brain_Spinal_cord_cervical_c-1:GTEx/v8/Brain_Substantia_nigra
rs78454137	ENSG00000185829	*ARL17A*	17	44594068	44657088	-1	protein_coding	51326	ARL17A	NA	-2.38258287	2664	4.39E-38	7.42E-31	GTEx/v8/Brain_Amygdala:GTEx/v8/Brain_Anterior_cingulate_cortex_BA24:GTEx/v8/Brain_Caudate_basal_ganglia:GTEx/v8/Brain_Cerebellar_Hemisphere:GTEx/v8/Brain_Cerebellum:GTEx/v8/Brain_Cortex:GTEx/v8/Brain_Frontal_Cortex_BA9:GTEx/v8/Brain_Hippocampus:GTEx/v8/Brain_Hypothalamus:GTEx/v8/Brain_Nucleus_accumbens_basal_ganglia:GTEx/v8/Brain_Putamen_basal_ganglia
rs78454137	ENSG00000232300	*FAM215B*	17	44636196	44640161	-1	sense_intronic	644297	FAM215B	NA	NA	2664	6.29E-24	2.68E-18	GTEx/v8/Brain_Cerebellar_Hemisphere:GTEx/v8/Brain_Cerebellum
rs78454137	ENSG00000073969	*NSF*	17	44668035	44834830	1	protein_coding	4905	NSF	0.71368213	-0.566246769	2386	1.64E-06	2.14E-10	GTEx/v8/Brain_Cerebellum
rs78454137	ENSG00000108379	*WNT3*	17	44839872	44910520	-1	protein_coding	7473	WNT3	0.946108101	-0.191110949	14	2.52E-05	1.18E-51	GTEx/v8/Brain_Cortex
rs78454137	ENSG00000263142	*LRRC37A17P*	17	45055847	45131935	1	pseudogene	644397	LRRC37A17P	NA	NA	903	2.50E-05	0.0172168	GTEx/v8/Brain_Cerebellum
rs2628207	ENSG00000263720	*RP11-389J22.1*	18	63532498	63533633	1	sense_intronic	NA	NA	NA	NA	187	2.00E-25	4.84E-20	GTEx/v8/Brain_Cerebellar_Hemisphere:GTEx/v8/Brain_Cerebellum
rs7408912	ENSG00000269386	*RAB11B-AS1*	19	8439257	8455569	-1	antisense	100507567	RAB11B-AS1	NA	NA	20	6.22E-07	2.72E-08	GTEx/v8/Brain_Cortex:GTEx/v8/Brain_Frontal_Cortex_BA9
rs7408912	ENSG00000099785	*MARCHF2*	19	8478154	8503901	1	protein_coding	51257	MARCHF2	0.246993249	0.040537347	21	2.18E-09	1.86E-22	GTEx/v8/Brain_Caudate_basal_ganglia:GTEx/v8/Brain_Hippocampus:GTEx/v8/Brain_Nucleus_accumbens_basal_ganglia:GTEx/v8/Brain_Putamen_basal_ganglia
rs13037664	ENSG00000101412	*E2F1*	20	32263489	32274210	-1	protein_coding	1869	E2F1	0.955530545	-0.324323693	1	4.18E-05	4.36E-14	GTEx/v8/Brain_Spinal_cord_cervical_c-1
rs13037664	ENSG00000198646	*NCOA6*	20	33284722	33413452	-1	protein_coding	23054	NCOA6	0.990815417	-0.852306051	90	2.48E-12	3.67E-08	GTEx/v8/Brain_Cerebellar_Hemisphere:GTEx/v8/Brain_Cerebellum:GTEx/v8/Brain_Cortex:GTEx/v8/Brain_Frontal_Cortex_BA9:GTEx/v8/Brain_Hippocampus:GTEx/v8/Brain_Hypothalamus:GTEx/v8/Brain_Nucleus_accumbens_basal_ganglia
rs11913445	ENSG00000099899	*TRMT2A*	22	20099389	20104915	-1	protein_coding	27037	TRMT2A	1.31E-05	NA	5	2.59E-05	0.0244377	GTEx/v8/Brain_Cerebellar_Hemisphere
rs11913445	ENSG00000099904	*ZDHHC8*	22	20116979	20135530	1	protein_coding	29801	ZDHHC8	0.988991096	NA	5	1.56E-05	0.0313939	GTEx/v8/Brain_Caudate_basal_ganglia
rs20551	ENSG00000128285	*MCHR1*	22	41074754	41078818	1	protein_coding	2847	MCHR1	0.001148393	-0.081006493	6	1.89E-05	8.11E-10	GTEx/v8/Brain_Cerebellum
rs20551	ENSG00000100372	*SLC25A17*	22	41165634	41215403	-1	protein_coding	10478	SLC25A17	0.024184844	-0.779478694	41	6.86E-06	2.58E-05	GTEx/v8/Brain_Cerebellum:GTEx/v8/Brain_Nucleus_accumbens_basal_ganglia:GTEx/v8/Brain_Putamen_basal_ganglia
rs20551	ENSG00000213857	*RP11-12M9.4*	22	41470184	41471243	-1	pseudogene	NA	NA	NA	NA	6	8.01E-05	0.0176512	GTEx/v8/Brain_Cerebellum
rs20551	ENSG00000100393	*EP300*	22	41487790	41576081	1	protein_coding	2033	EP300	1	NA	113	2.44E-06	0.00184947	GTEx/v8/Brain_Cerebellum:GTEx/v8/Brain_Hypothalamus
rs20551	ENSG00000100395	*L3MBTL2*	22	41601209	41627275	1	protein_coding	83746	L3MBTL2	0.013028043	-0.618259224	121	1.80E-08	5.18E-05	GTEx/v8/Brain_Anterior_cingulate_cortex_BA24:GTEx/v8/Brain_Frontal_Cortex_BA9
rs20551	ENSG00000100401	*RANGAP1*	22	41641615	41682255	-1	protein_coding	5905	RANGAP1	0.13271872	-0.376521602	76	7.87E-07	1.39E-06	GTEx/v8/Brain_Cerebellar_Hemisphere:GTEx/v8/Brain_Cerebellum
rs20551	ENSG00000100403	*ZC3H7B*	22	41697526	41756151	1	protein_coding	23264	ZC3H7B	0.990761551	-0.628157842	125	4.49E-08	0.00010307	GTEx/v8/Brain_Cerebellum
rs20551	ENSG00000100412	*ACO2*	22	41865129	41924993	1	protein_coding	50	ACO2	0.454377775	-0.617411984	21	2.91E-05	0.00290367	GTEx/v8/Brain_Cerebellum
rs20551	ENSG00000100413	*POLR3H*	22	41921808	41940610	-1	protein_coding	171568	POLR3H	0.002007106	-1.305852274	120	2.19E-08	8.24E-14	GTEx/v8/Brain_Cerebellum:GTEx/v8/Brain_Cortex:GTEx/v8/Brain_Frontal_Cortex_BA9:GTEx/v8/Brain_Putamen_basal_ganglia
rs20551	ENSG00000172346	*CSDC2*	22	41956767	41973745	1	protein_coding	27254	CSDC2	0.079803516	0.31935561	121	2.14E-09	6.71E-18	GTEx/v8/Brain_Cortex:GTEx/v8/Brain_Frontal_Cortex_BA9
rs20551	ENSG00000167077	*MEI1*	22	42095503	42195460	1	protein_coding	150365	MEI1	1.22E-08	0.500155337	115	1.71E-06	1.60E-05	GTEx/v8/Brain_Anterior_cingulate_cortex_BA24:GTEx/v8/Brain_Frontal_Cortex_BA9

rs20551	ENSG00000159958	*TNFRSF13C*	22	42321045	42322822	-1	protein_coding	115650	TNFRSF13C	0.641054688	-0.063981155	31	1.87E-05	1.22E-11	GTEx/v8/Brain_Cerebellar_Hemisphere
rs20551	ENSG00000205704	*LINC00634*	22	42348169	42354937	1	pseudogene	339674	LINC00634	NA	NA	9	6.19E-05	0.000116414	GTEx/v8/Brain_Frontal_Cortex_BA9
rs20551	ENSG00000183066	*WBP2NL*	22	42394729	42454460	1	protein_coding	164684	WBP2NL	2.07E-09	0.730692723	1	7.68E-05	1.04E-06	GTEx/v8/Brain_Frontal_Cortex_BA9
rs20551	ENSG00000198951	*NAGA*	22	42454358	42466846	-1	protein_coding	4668	NAGA	2.02E-06	0.183467033	6	8.98E-07	7.04E-29	GTEx/v8/Brain_Cerebellar_Hemisphere:GTEx/v8/Brain_Cerebellum
rs20551	ENSG00000100197	*CYP2D6*	22	42522501	42526908	-1	protein_coding	1565	CYP2D6	9.23E-10	0.153953666	12	2.97E-05	4.44E-35	GTEx/v8/Brain_Cerebellum:GTEx/v8/Brain_Nucleus_accumbens_basal_ganglia

b) Irritability given bipolar I disorder

IndSigSNPs	ensg	symbol	chr	start	end	strand	type	entrezID	HUGO	pLI	ncRVIS	eqtlMapSNPs	eqtlMapminP	eqtlMapminQ	eqtlMapts
rs4953150	ENSG00000236502	*SIX3-AS1*	2	45167293	45169012	-1	antisense	100506108	SIX3-AS1	NA	NA	8	4.68E-07	4.76E-10	GTEx/v8/Brain_Caudate_basal_ganglia:GTEx/v8/Brain_Nucleus_accumbens_basal_ganglia
rs4953150	ENSG00000225156	*AC012354.6*	2	45181803	45195901	1	lincRNA	NA	NA	NA	NA	10	7.40E-08	9.59E-10	GTEx/v8/Brain_Caudate_basal_ganglia:GTEx/v8/Brain_Nucleus_accumbens_basal_ganglia
rs6244881	ENSG00000176349	*AC110781.3*	7	1878222	1889567	1	protein_coding	NA	NA	NA	NA	74	3.26E-07	2.61E-06	GTEx/v8/Brain_Nucleus_accumbens_basal_ganglia
rs6244881	ENSG00000122687	*FTSJ2*	7	2273866	2281840	-1	protein_coding	29960	FTSJ2	1.97E-05	0.096595649	95	1.66E-07	4.01E-12	GTEx/v8/Brain_Caudate_basal_ganglia:GTEx/v8/Brain_Cerebellar_Hemisphere:GTEx/v8/Brain_Cortex
rs62491417	ENSG00000006459	*KDM7A*	7	139784546	139876835	-1	protein_coding	80853	KDM7A	0.99869438	-1.580266986	2	1.06E-17	3.67E-14	GTEx/v8/Brain_Caudate_basal_ganglia:GTEx/v8/Brain_Cerebellar_Hemisphere:GTEx/v8/Brain_Cerebellum:GTEx/v8/Brain_Cortex:GTEx/v8/Brain_Frontal_Cortex_BA9:GTEx/v8/Brain_Hippocampus:GTEx/v8/Brain_Nucleus_accumbens_basal_ganglia:GTEx/v8/Brain_Putamen_basal_ganglia:GTEx/v8/Brain_Spinal_cord_cervical_c-1
rs6464217	ENSG00000157764	*BRAF*	7	140419127	140624564	-1	protein_coding	673	BRAF	0.999978196	NA	1	2.80E-05	0.0377877	GTEx/v8/Brain_Cerebellum
rs6464217	ENSG00000090263	*MRPS33*	7	140705854	140715028	-1	protein_coding	51650	MRPS33	0.003395718	0.268392925	51	2.30E-09	1.80E-05	GTEx/v8/Brain_Caudate_basal_ganglia:GTEx/v8/Brain_Cortex:GTEx/v8/Brain_Putamen_basal_ganglia
rs1533059	ENSG00000249188	*ENPP7P1*	8	8011830	8074438	1	pseudogene	100421823	ENPP7P1	NA	NA	111	5.37E-09	1.79E-10	GTEx/v8/Brain_Cerebellar_Hemisphere:GTEx/v8/Brain_Cerebellum
rs1533059	ENSG00000253893	*FAM85B*	8	8025341	8084136	-1	antisense	105379219	FAM85B	NA	NA	114	1.17E-19	1.81E-26	GTEx/v8/Brain_Amygdala:GTEx/v8/Brain_Anterior_cingulate_cortex_BA24:GTEx/v8/Brain_Caudate_basal_ganglia:GTEx/v8/Brain_Cerebellar_Hemisphere:GTEx/v8/Brain_Cerebellum:GTEx/v8/Brain_Cortex:GTEx/v8/Brain_Frontal_Cortex_BA9:GTEx/v8/Brain_Hippocampus:GTEx/v8/Brain_Hypothalamus:GTEx/v8/Brain_Nucleus_accumbens_basal_ganglia:GTEx/v8/Brain_Putamen_basal_ganglia:GTEx/v8/Brain_Spinal_cord_cervical_c-1:GTEx/v8/Brain_Substantia_nigra
rs1533059	ENSG00000173295	*FAM86B3P*	8	8086117	8102387	1	pseudogene	286042	FAM86B3P	NA	NA	114	6.14E-18	9.21E-28	GTEx/v8/Brain_Anterior_cingulate_cortex_BA24:GTEx/v8/Brain_Cerebellar_Hemisphere:GTEx/v8/Brain_Cerebellum:GTEx/v8/Brain_Cortex:GTEx/v8/Brain_Frontal_Cortex_BA9:GTEx/v8/Brain_Hippocampus
rs1533059	ENSG00000253981	*ALG1L13P*	8	8093525	8102189	-1	pseudogene	106479038	ALG1L13P	NA	NA	30	1.99E-06	1.69E-06	GTEx/v8/Brain_Cerebellar_Hemisphere:GTEx/v8/Brain_Cerebellum:GTEx/v8/Brain_Cortex
rs1533059	ENSG00000233609	*RP11-62H7.2*	8	8818710	8836535	1	pseudogene	NA	NA	NA	NA	114	8.42E-20	3.12E-15	GTEx/v8/Brain_Amygdala:GTEx/v8/Brain_Anterior_cingulate_cortex_BA24:GTEx/v8/Brain_Caudate_basal_ganglia:GTEx/v8/Brain_Cerebellar_Hemisphere:GTEx/v8/Brain_Cerebellum:GTEx/v8/Brain_Cortex:GTEx/v8/Brain_Frontal_Cortex_BA9:GTEx/v8/Brain_Hippocampus:GTEx/v8/Brain_Hypothalamus:GTEx/v8/Brain_Nucleus_accumbens_basal_ganglia:GTEx/v8/Brain_Putamen_basal_ganglia:GTEx/v8/Brain_Substantia_nigra
rs1533059	ENSG00000254340	*RP11-10A14.3*	8	8998934	9002945	1	antisense	NA	NA	NA	NA	1	1.64E-05	0.00123905	GTEx/v8/Brain_Cerebellum
rs2952176	ENSG00000261451	*RP11-981G7.1*	8	10291182	10295822	1	sense_overlapping	NA	NA	NA	NA	25	6.94E-07	6.07E-19	GTEx/v8/Brain_Cerebellar_Hemisphere:GTEx/v8/Brain_Cerebellum
rs16884419	ENSG00000156103	*MMP16*	8	89044237	89340254	-1	protein_coding	4325	MMP16	0.916974084	-3.432432894	95	1.36E-05	0.0179096	GTEx/v8/Brain_Cerebellum
rs4903249	ENSG00000119682	*AREL1*	14	75120140	75179818	-1	protein_coding	9870	AREL1	0.011802084	-0.560094839	14	7.29E-07	1.08E-05	GTEx/v8/Brain_Frontal_Cortex_BA9
rs2054213	ENSG00000196118	*C16orf93*	16	30768744	30774031	-1	protein_coding	90835	C16orf93	8.00E-05	-0.754318822	102	1.59E-13	2.99E-15	GTEx/v8/Brain_Caudate_basal_ganglia:GTEx/v8/Brain_Cortex:GTEx/v8/Brain_Frontal_Cortex_BA9:GTEx/v8/Brain_Hippocampus:GTEx/v8/Brain_Hypothalamus:GTEx/v8/Brain_Nucleus_accumbens_basal_ganglia
rs2054213	ENSG00000103549	*RNF40*	16	30773066	30787628	1	protein_coding	9810	RNF40	0.999994909	-1.913783614	25	4.02E-09	1.74E-05	GTEx/v8/Brain_Caudate_basal_ganglia:GTEx/v8/Brain_Cerebellar_Hemisphere:GTEx/v8/Brain_Cerebellum:GTEx/v8/Brain_Nucleus_accumbens_basal_ganglia:GTEx/v8/Brain_Putamen_basal_ganglia
rs2054213	ENSG00000099381	*SETD1A*	16	30968615	30996437	1	protein_coding	9739	SETD1A	0.999996231	-0.894969313	57	3.17E-06	0.00390586	GTEx/v8/Brain_Cerebellum

rs2054213	ENSG00000099377	*HSD3B7*	16	30996519	31000473	1	protein_coding	80270	HSD3B7	4.64E-06	-0.867416987	94	1.96E-06	8.50E-05	GTEx/v8/Brain_Anterior_cingulate_cortex_BA24:GTEx/v8/Brain_Cortex:GTEx/v8/Brain_Hypothalamus
rs2054213	ENSG00000099365	*STX1B*	16	31000577	31021949	-1	protein_coding	112755	STX1B	0.944474733	-1.610450086	106	5.01E-07	0.000791194	GTEx/v8/Brain_Cerebellar_Hemisphere:GTEx/v8/Brain_Cerebellum
rs2054213	ENSG00000103496	*STX4*	16	31044210	31054296	1	protein_coding	6810	STX4	9.66E-05	-0.421867635	137	1.18E-12	1.78E-08	GTEx/v8/Brain_Anterior_cingulate_cortex_BA24:GTEx/v8/Brain_Cerebellum:GTEx/v8/Brain_Cortex:GTEx/v8/Brain_Frontal_Cortex_BA9:GTEx/v8/Brain_Hypothalamus:GTEx/v8/Brain_Nucleus_accumbens_basal_ganglia
rs2054213	ENSG00000260911	*RP11-196G11.2*	16	31054471	31061201	1	lincRNA	NA	NA	NA	NA	137	1.45E-26	3.33E-21	GTEx/v8/Brain_Anterior_cingulate_cortex_BA24:GTEx/v8/Brain_Caudate_basal_ganglia:GTEx/v8/Brain_Cerebellar_Hemisphere:GTEx/v8/Brain_Cerebellum:GTEx/v8/Brain_Cortex:GTEx/v8/Brain_Frontal_Cortex_BA9:GTEx/v8/Brain_Hippocampus:GTEx/v8/Brain_Nucleus_accumbens_basal_ganglia:GTEx/v8/Brain_Putamen_basal_ganglia:GTEx/v8/Brain_Spinal_cord_cervical_c-1:GTEx/v8/Brain_Substantia_nigra
rs2054213	ENSG00000167395	*ZNF646*	16	31085743	31095517	1	protein_coding	9726	ZNF646	0.00556536	0.564563942	75	1.54E-06	0.00486533	GTEx/v8/Brain_Caudate_basal_ganglia:GTEx/v8/Brain_Hippocampus
rs2054213	ENSG00000167397	*VKORC1*	16	31102163	31107301	-1	protein_coding	79001	VKORC1	0.081803076	-0.611112737	92	1.23E-06	0.000396364	GTEx/v8/Brain_Caudate_basal_ganglia:GTEx/v8/Brain_Cortex
rs2054213	ENSG00000103510	*KAT8*	16	31127075	31142714	1	protein_coding	84148	KAT8	0.912576388	0.037392946	137	1.20E-12	3.51E-17	GTEx/v8/Brain_Caudate_basal_ganglia:GTEx/v8/Brain_Cerebellar_Hemisphere:GTEx/v8/Brain_Cerebellum:GTEx/v8/Brain_Cortex:GTEx/v8/Brain_Frontal_Cortex_BA9:GTEx/v8/Brain_Hippocampus:GTEx/v8/Brain_Hypothalamus:GTEx/v8/Brain_Nucleus_accumbens_basal_ganglia:GTEx/v8/Brain_Putamen_basal_ganglia
rs2054213	ENSG00000262766	*RP11-196G11.4*	16	31129399	31130068	1	sense_intronic	NA	NA	NA	NA	12	2.78E-05	0.000263052	GTEx/v8/Brain_Cerebellum
rs2054213	ENSG00000178226	*PRSS36*	16	31150246	31161415	-1	protein_coding	146547	PRSS36	4.42E-13	-0.151362733	126	1.72E-11	1.01E-22	GTEx/v8/Brain_Amygdala:GTEx/v8/Brain_Anterior_cingulate_cortex_BA24:GTEx/v8/Brain_Caudate_basal_ganglia:GTEx/v8/Brain_Cerebellar_Hemisphere:GTEx/v8/Brain_Cerebellum:GTEx/v8/Brain_Cortex:GTEx/v8/Brain_Frontal_Cortex_BA9:GTEx/v8/Brain_Hippocampus:GTEx/v8/Brain_Hypothalamus:GTEx/v8/Brain_Nucleus_accumbens_basal_ganglia:GTEx/v8/Brain_Putamen_basal_ganglia
rs6060009	ENSG00000101412	*E2F1*	20	32263489	32274210	-1	protein_coding	1869	E2F1	0.955530545	-0.324323693	1	4.18E-05	4.36E-14	GTEx/v8/Brain_Spinal_cord_cervical_c-1
rs6060009	ENSG00000198646	*NCOA6*	20	33284722	33413452	-1	protein_coding	23054	NCOA6	0.990815417	-0.852306051	104	2.48E-12	3.67E-08	GTEx/v8/Brain_Cerebellar_Hemisphere:GTEx/v8/Brain_Cerebellum:GTEx/v8/Brain_Cortex:GTEx/v8/Brain_Frontal_Cortex_BA9:GTEx/v8/Brain_Hippocampus:GTEx/v8/Brain_Hypothalamus:GTEx/v8/Brain_Nucleus_accumbens_basal_ganglia
rs6060009	ENSG00000131069	*ACSS2*	20	33459949	33515769	1	protein_coding	55902	ACSS2	8.97E-11	-0.504093957	2	2.51E-05	1.45E-11	GTEx/v8/Brain_Cerebellum
rs6060009	ENSG00000078814	*MYH7B*	20	33563206	33590240	1	protein_coding	57644	MYH7B	1.33E-23	0.056785336	4	0.00018444	1.64E-13	GTEx/v8/Brain_Cerebellum
rs20551	ENSG00000128285	*MCHR1*	22	41074754	41078818	1	protein_coding	2847	MCHR1	0.001148393	-0.081006493	6	1.89E-05	8.11E-10	GTEx/v8/Brain_Cerebellum
rs20551	ENSG00000100372	*SLC25A17*	22	41165634	41215403	-1	protein_coding	10478	SLC25A17	0.024184844	-0.779478694	41	6.86E-06	2.58E-05	GTEx/v8/Brain_Cerebellum:GTEx/v8/Brain_Nucleus_accumbens_basal_ganglia:GTEx/v8/Brain_Putamen_basal_ganglia
rs20551	ENSG00000213857	*RP11-12M9.4*	22	41470184	41471243	-1	pseudogene	NA	NA	NA	NA	6	8.01E-05	0.0176512	GTEx/v8/Brain_Cerebellum
rs20551	ENSG00000100393	*EP300*	22	41487790	41576081	1	protein_coding	2033	EP300	1	NA	113	2.44E-06	0.00184947	GTEx/v8/Brain_Cerebellum:GTEx/v8/Brain_Hypothalamus
rs20551	ENSG00000100395	*L3MBTL2*	22	41601209	41627275	1	protein_coding	83746	L3MBTL2	0.013028043	-0.618259224	121	1.80E-08	5.18E-05	GTEx/v8/Brain_Anterior_cingulate_cortex_BA24:GTEx/v8/Brain_Frontal_Cortex_BA9
rs20551	ENSG00000100401	*RANGAP1*	22	41641615	41682255	-1	protein_coding	5905	RANGAP1	0.13271872	-0.376521602	76	7.87E-07	1.39E-06	GTEx/v8/Brain_Cerebellar_Hemisphere:GTEx/v8/Brain_Cerebellum
rs20551	ENSG00000100403	*ZC3H7B*	22	41697526	41756151	1	protein_coding	23264	ZC3H7B	0.990761551	-0.628157842	125	4.49E-08	0.00010307	GTEx/v8/Brain_Cerebellum
rs20551	ENSG00000100412	*ACO2*	22	41865129	41924993	1	protein_coding	50	ACO2	0.454377775	-0.617411984	21	2.91E-05	0.00290367	GTEx/v8/Brain_Cerebellum
rs20551	ENSG00000100413	*POLR3H*	22	41921808	41940610	-1	protein_coding	171568	POLR3H	0.002007106	-1.305852274	120	2.19E-08	8.24E-14	GTEx/v8/Brain_Cerebellum:GTEx/v8/Brain_Cortex:GTEx/v8/Brain_Frontal_Cortex_BA9:GTEx/v8/Brain_Putamen_basal_ganglia
rs20551	ENSG00000172346	*CSDC2*	22	41956767	41973745	1	protein_coding	27254	CSDC2	0.079803516	0.31935561	121	2.14E-09	6.71E-18	GTEx/v8/Brain_Cortex:GTEx/v8/Brain_Frontal_Cortex_BA9
rs20551	ENSG00000167077	*MEI1*	22	42095503	42195460	1	protein_coding	150365	MEI1	1.22E-08	0.500155337	115	1.71E-06	1.60E-05	GTEx/v8/Brain_Anterior_cingulate_cortex_BA24:GTEx/v8/Brain_Frontal_Cortex_BA9
rs20551	ENSG00000159958	*TNFRSF13C*	22	42321045	42322822	-1	protein_coding	115650	TNFRSF13C	0.641054688	-0.063981155	31	1.87E-05	1.22E-11	GTEx/v8/Brain_Cerebellar_Hemisphere

rs20551	ENSG00000205704	*LINC00634*	22	42348169	42354937	1	pseudogene	339674	LINC00634	NA	NA	9	6.19E-05	0.000116414	GTEx/v8/Brain_Frontal_Cortex_BA9
rs20551	ENSG00000183066	*WBP2NL*	22	42394729	42454460	1	protein_coding	164684	WBP2NL	2.07E-09	0.730692723	1	7.68E-05	1.04E-06	GTEx/v8/Brain_Frontal_Cortex_BA9
rs20551	ENSG00000198951	*NAGA*	22	42454358	42466846	-1	protein_coding	4668	NAGA	2.02E-06	0.183467033	6	8.98E-07	7.04E-29	GTEx/v8/Brain_Cerebellar_Hemisphere:GTEx/v8/Brain_Cerebellum
rs20551	ENSG00000100197	*CYP2D6*	22	42522501	42526908	-1	protein_coding	1565	CYP2D6	9.23E-10	0.153953666	12	2.97E-05	4.44E-35	GTEx/v8/Brain_Cerebellum:GTEx/v8/Brain_Nucleus_accumbens_basal_ganglia

c) Irritability given major depressive disorder

IndSigSNPs	ensg	symbol	chr	start	end	strand	type	entrezID	HUGO	pLI	ncRVIS	eqtlMapSNPs	eqtlMapminP	eqtlMapminQ	eqtlMapts
rs1121480	ENSG00000162366	*PDZK1IP1*	1	47649265	47656716	-1	protein_coding	10158	PDZK1IP1	0.000484065	-0.343318344	38	6.02E-06	2.89E-10	GTEx/v8/Brain_Cortex:GTEx/v8/Brain_Putamen_basal_ganglia
rs1121480	ENSG00000162367	*TAL1*	1	47681962	47697892	-1	protein_coding	6886	TAL1	0.824692215	-0.797257236	42	7.92E-06	0.0180879	GTEx/v8/Brain_Cortex
rs1121480	ENSG00000186790	*FOXE3*	1	47881744	47883723	1	protein_coding	2301	FOXE3	NA	-0.345111816	41	3.21E-06	1.28E-08	GTEx/v8/Brain_Cerebellum
rs1993709	ENSG00000172260	*NEGR1*	1	71861623	72748417	-1	protein_coding	257194	NEGR1	0.951349395	-1.713579543	14	4.26E-05	1.84E-05	GTEx/v8/Brain_Caudate_basal_ganglia:GTEx/v8/Brain_Putamen_basal_ganglia
rs1993709	ENSG00000227207	*RPL31P12*	1	72767155	72767512	1	pseudogene	100270887	RPL31P12	NA	NA	99	1.73E-17	1.23E-38	GTEx/v8/Brain_Cerebellar_Hemisphere:GTEx/v8/Brain_Cerebellum
rs1198575	ENSG00000225206	*MIR137HG*	1	98453556	98515419	-1	lincRNA	400765	MIR137HG	NA	NA	1	5.56E-05	5.79E-06	GTEx/v8/Brain_Caudate_basal_ganglia
rs4019431	ENSG00000119820	*YIPF4*	2	32502979	32541663	1	protein_coding	84272	YIPF4	0.019788591	-1.79291052	17	3.05E-06	0.0101889	GTEx/v8/Brain_Putamen_basal_ganglia
rs4953150	ENSG00000236502	*SIX3-AS1*	2	45167293	45169012	-1	antisense	100506108	SIX3-AS1	NA	NA	8	4.68E-07	4.76E-10	GTEx/v8/Brain_Caudate_basal_ganglia:GTEx/v8/Brain_Nucleus_accumbens_basal_ganglia
rs4953150	ENSG00000225156	*AC012354.6*	2	45181803	45195901	1	lincRNA	NA	NA	NA	NA	10	7.40E-08	9.59E-10	GTEx/v8/Brain_Caudate_basal_ganglia:GTEx/v8/Brain_Nucleus_accumbens_basal_ganglia
rs2442824	ENSG00000206573	*SETD5-AS1*	3	9391373	9440263	-1	antisense	440944	SETD5-AS1	NA	NA	25	1.05E-08	7.18E-05	GTEx/v8/Brain_Cerebellar_Hemisphere:GTEx/v8/Brain_Cerebellum
rs2442824	ENSG00000134077	*THUMPD3*	3	9404526	9428475	1	protein_coding	25917	THUMPD3	4.00E-11	0.700963169	24	9.24E-10	1.57E-13	GTEx/v8/Brain_Caudate_basal_ganglia:GTEx/v8/Brain_Cerebellum:GTEx/v8/Brain_Cortex:GTEx/v8/Brain_Frontal_Cortex_BA9:GTEx/v8/Brain_Hypothalamus:GTEx/v8/Brain_Nucleus_accumbens_basal_ganglia
rs4308307	ENSG00000164045	*CDC25A*	3	48198636	48229892	-1	protein_coding	993	CDC25A	0.979698045	-0.724000674	31	0.000175411	1.73E-15	GTEx/v8/Brain_Cerebellar_Hemisphere
rs4308307	ENSG00000229759	*MRPS18AP1*	3	48297840	48298428	-1	pseudogene	359761	MRPS18AP1	NA	NA	66	0.000117392	1.29E-23	GTEx/v8/Brain_Cerebellum
rs4308307	ENSG00000164053	*ATRIP*	3	48488114	48507115	1	protein_coding	84126	ATRIP	0.00148698	NA	1	6.29E-05	0.00252996	GTEx/v8/Brain_Putamen_basal_ganglia
rs4308307	ENSG00000145040	*UCN2*	3	48599160	48601206	-1	protein_coding	90226	UCN2	0.157235729	0.006326735	3	0.000114759	0.00573708	GTEx/v8/Brain_Cerebellum
rs4308307	ENSG00000213672	*NCKIPSD*	3	48701364	48723797	-1	protein_coding	51517	NCKIPSD	0.007131362	-0.551018211	167	1.59E-34	8.19E-28	GTEx/v8/Brain_Amygdala:GTEx/v8/Brain_Anterior_cingulate_cortex_BA24:GTEx/v8/Brain_Caudate_basal_ganglia:GTEx/v8/Brain_Cerebellar_Hemisphere:GTEx/v8/Brain_Cerebellum:GTEx/v8/Brain_Cortex:GTEx/v8/Brain_Frontal_Cortex_BA9:GTEx/v8/Brain_Hippocampus:GTEx/v8/Brain_Hypothalamus:GTEx/v8/Brain_Nucleus_accumbens_basal_ganglia:GTEx/v8/Brain_Putamen_basal_ganglia:GTEx/v8/Brain_Spinal_cord_cervical_c-1:GTEx/v8/Brain_Substantia_nigra
rs4308307	ENSG00000068745	*IP6K2*	3	48725436	48777786	-1	protein_coding	51447	IP6K2	0.989457703	-0.392798697	1	2.46E-08	1.48E-13	GTEx/v8/Brain_Cerebellar_Hemisphere:GTEx/v8/Brain_Cerebellum:GTEx/v8/Brain_Cortex
rs4308307	ENSG00000178467	*P4HTM*	3	49027319	49044587	1	protein_coding	54681	P4HTM	0.023274936	NA	167	1.50E-11	3.79E-08	GTEx/v8/Brain_Caudate_basal_ganglia:GTEx/v8/Brain_Cerebellar_Hemisphere:GTEx/v8/Brain_Cerebellum:GTEx/v8/Brain_Cortex:GTEx/v8/Brain_Frontal_Cortex_BA9:GTEx/v8/Brain_Hippocampus:GTEx/v8/Brain_Hypothalamus:GTEx/v8/Brain_Nucleus_accumbens_basal_ganglia:GTEx/v8/Brain_Putamen_basal_ganglia:GTEx/v8/Brain_Spinal_cord_cervical_c-1
rs4308307	ENSG00000178252	*WDR6*	3	49044495	49053386	1	protein_coding	11180	WDR6	9.20E-07	-0.058949543	167	2.04E-27	1.30E-21	GTEx/v8/Brain_Amygdala:GTEx/v8/Brain_Caudate_basal_ganglia:GTEx/v8/Brain_Cerebellar_Hemisphere:GTEx/v8/Brain_Cerebellum:GTEx/v8/Brain_Cortex:GTEx/v8/Brain_Frontal_Cortex_BA9:GTEx/v8/Brain_Hippocampus:GTEx/v8/Brain_Nucleus_accumbens_basal_ganglia:GTEx/v8/Brain_Putamen_basal_ganglia:GTEx/v8/Brain_Spinal_cord_cervical_c-1:GTEx/v8/Brain_Substantia_nigra

rs4308307	ENSG00000178149	*DALRD3*	3	49052921	49059726	-1	protein_coding	55152	DALRD3	1.85E-05	0.072306759	167	9.86E-11	1.79E-09	GTEx/v8/Brain_Anterior_cingulate_cortex_BA24:GTEx/v8/Brain_Cerebellar_Hemisphere:GTEx/v8/Brain_Cerebellum:GTEx/v8/Brain_Cortex:GTEx/v8/Brain_Frontal_Cortex_BA9
rs4308307	ENSG00000198218	*QRICH1*	3	49067140	49131796	-1	protein_coding	54870	QRICH1	0.996069748	-0.27929443	163	1.05E-07	3.56E-12	GTEx/v8/Brain_Cerebellar_Hemisphere:GTEx/v8/Brain_Cerebellum:GTEx/v8/Brain_Cortex
rs4308307	ENSG00000270441	*RP11-694I15.7*	3	49177519	49198284	-1	pseudogene	NA	NA	NA	NA	148	1.38E-05	0.000344885	GTEx/v8/Brain_Cerebellar_Hemisphere:GTEx/v8/Brain_Cerebellum
rs4308307	ENSG00000177352	*CCDC71*	3	49199968	49203754	-1	protein_coding	64925	CCDC71	0.016989639	NA	167	9.42E-20	4.03E-15	GTEx/v8/Brain_Amygdala:GTEx/v8/Brain_Caudate_basal_ganglia:GTEx/v8/Brain_Cerebellar_Hemisphere:GTEx/v8/Brain_Cerebellum:GTEx/v8/Brain_Cortex:GTEx/v8/Brain_Frontal_Cortex_BA9:GTEx/v8/Brain_Hippocampus:GTEx/v8/Brain_Nucleus_accumbens_basal_ganglia:GTEx/v8/Brain_Putamen_basal_ganglia
rs4308307	ENSG00000185909	*KLHDC8B*	3	49209044	49213917	1	protein_coding	200942	KLHDC8B	0.01042142	-0.077352225	166	1.55E-06	0.00138032	GTEx/v8/Brain_Cerebellum:GTEx/v8/Brain_Nucleus_accumbens_basal_ganglia
rs4308307	ENSG00000225399	*RP11-3B7.1*	3	49297518	49298744	1	protein_coding	NA	NA	NA	NA	32	7.14E-06	0.00866008	GTEx/v8/Brain_Anterior_cingulate_cortex_BA24
rs4308307	ENSG00000233276	*GPX1*	3	49394609	49396033	-1	protein_coding	2876	GPX1	0.006931651	-0.616399091	167	7.53E-11	5.90E-11	GTEx/v8/Brain_Caudate_basal_ganglia:GTEx/v8/Brain_Cerebellar_Hemisphere:GTEx/v8/Brain_Cerebellum:GTEx/v8/Brain_Cortex:GTEx/v8/Brain_Frontal_Cortex_BA9:GTEx/v8/Brain_Nucleus_accumbens_basal_ganglia
rs4308307	ENSG00000145020	*AMT*	3	49454211	49460186	-1	protein_coding	275	AMT	0.000569475	-0.022637077	167	3.90E-23	7.74E-32	GTEx/v8/Brain_Anterior_cingulate_cortex_BA24:GTEx/v8/Brain_Caudate_basal_ganglia:GTEx/v8/Brain_Cerebellar_Hemisphere:GTEx/v8/Brain_Cerebellum:GTEx/v8/Brain_Cortex:GTEx/v8/Brain_Frontal_Cortex_BA9:GTEx/v8/Brain_Hippocampus:GTEx/v8/Brain_Hypothalamus:GTEx/v8/Brain_Nucleus_accumbens_basal_ganglia:GTEx/v8/Brain_Putamen_basal_ganglia:GTEx/v8/Brain_Spinal_cord_cervical_c-1
rs4308307	ENSG00000145029	*NICN1*	3	49460379	49466759	-1	protein_coding	84276	NICN1	0.257245529	-1.087291051	162	8.90E-07	9.43E-05	GTEx/v8/Brain_Caudate_basal_ganglia:GTEx/v8/Brain_Cortex:GTEx/v8/Brain_Frontal_Cortex_BA9:GTEx/v8/Brain_Nucleus_accumbens_basal_ganglia
rs4308307	ENSG00000226913	*BSN-AS2*	3	49586739	49591799	-1	lincRNA	100132677	BSN-AS2	NA	NA	167	1.69E-07	4.94E-06	GTEx/v8/Brain_Caudate_basal_ganglia:GTEx/v8/Brain_Cortex:GTEx/v8/Brain_Frontal_Cortex_BA9:GTEx/v8/Brain_Nucleus_accumbens_basal_ganglia:GTEx/v8/Brain_Putamen_basal_ganglia
rs4308307	ENSG00000173540	*GMPPB*	3	49754277	49761384	-1	protein_coding	29925	GMPPB	8.25E-05	-0.65955402	143	8.85E-08	1.80E-28	GTEx/v8/Brain_Amygdala:GTEx/v8/Brain_Anterior_cingulate_cortex_BA24:GTEx/v8/Brain_Cerebellar_Hemisphere:GTEx/v8/Brain_Cerebellum:GTEx/v8/Brain_Cortex:GTEx/v8/Brain_Frontal_Cortex_BA9:GTEx/v8/Brain_Hippocampus:GTEx/v8/Brain_Nucleus_accumbens_basal_ganglia:GTEx/v8/Brain_Putamen_basal_ganglia:GTEx/v8/Brain_Spinal_cord_cervical_c-1:GTEx/v8/Brain_Substantia_nigra
rs4308307	ENSG00000164078	*MST1R*	3	49924435	49941299	-1	protein_coding	4486	MST1R	1.69E-20	-0.377644955	8	8.42E-05	0.0236026	GTEx/v8/Brain_Hypothalamus
rs4308307	ENSG00000186792	*HYAL3*	3	50330262	50336899	-1	protein_coding	8372	HYAL3	1.52E-05	-0.353149956	10	1.44E-06	1.68E-09	GTEx/v8/Brain_Spinal_cord_cervical_c-1
rs10029973	ENSG00000237765	*FAM200B*	4	15683285	15707188	1	protein_coding	285550	FAM200B	NA	-0.311233821	3	3.08E-10	1.86E-12	GTEx/v8/Brain_Anterior_cingulate_cortex_BA24:GTEx/v8/Brain_Caudate_basal_ganglia:GTEx/v8/Brain_Cerebellar_Hemisphere:GTEx/v8/Brain_Cerebellum:GTEx/v8/Brain_Cortex:GTEx/v8/Brain_Frontal_Cortex_BA9:GTEx/v8/Brain_Hypothalamus:GTEx/v8/Brain_Nucleus_accumbens_basal_ganglia:GTEx/v8/Brain_Putamen_basal_ganglia
rs1301441	ENSG00000123219	*CENPK*	5	64813593	64858998	-1	protein_coding	64105	CENPK	1.66E-08	0.339527267	8	4.34E-06	3.17E-09	GTEx/v8/Brain_Nucleus_accumbens_basal_ganglia
rs1301441	ENSG00000113593	*PPWD1*	5	64859063	64883376	1	protein_coding	23398	PPWD1	0.031785775	0.411549096	11	6.57E-07	2.93E-12	GTEx/v8/Brain_Cortex:GTEx/v8/Brain_Nucleus_accumbens_basal_ganglia
rs13166120	ENSG00000250377	*CTC-467M3.3*	5	87988462	87989789	-1	lincRNA	NA	NA	NA	NA	24	1.36E-06	0.000243631	GTEx/v8/Brain_Anterior_cingulate_cortex_BA24:GTEx/v8/Brain_Cerebellar_Hemisphere:GTEx/v8/Brain_Cortex
rs4463213	ENSG00000113119	*TMCO6*	5	140019012	140024993	1	protein_coding	55374	TMCO6	8.15E-06	-0.195921903	34	8.57E-05	3.32E-13	GTEx/v8/Brain_Cerebellar_Hemisphere
rs4463213	ENSG00000120314	*WDR55*	5	140044261	140053709	1	protein_coding	54853	WDR55	1.48E-06	-1.037518539	3	3.25E-05	8.26E-07	GTEx/v8/Brain_Cerebellum
rs62382095	ENSG00000164574	*GALNT10*	5	153570290	153800544	1	protein_coding	55568	GALNT10	0.00044926	1.469543542	2	5.65E-05	9.34E-08	GTEx/v8/Brain_Cerebellum
rs9492590	ENSG00000164483	*SAMD3*	6	130465460	130686570	-1	protein_coding	154075	SAMD3	1.10E-06	0.088198284	55	5.84E-07	0.00198934	GTEx/v8/Brain_Cortex
rs844584	ENSG00000203727	*SAMD5*	6	147830063	148058683	1	protein_coding	389432	SAMD5	0.000898004	1.680995061	28	2.82E-09	2.81E-10	GTEx/v8/Brain_Cerebellum:GTEx/v8/Brain_Hypothalamus
rs6244881	ENSG00000176349	*AC110781.3*	7	1878222	1889567	1	protein_coding	NA	NA	NA	NA	74	3.26E-07	2.61E-06	GTEx/v8/Brain_Nucleus_accumbens_basal_ganglia
rs6244881	ENSG00000122687	*FTSJ2*	7	2273866	2281840	-1	protein_coding	29960	FTSJ2	1.97E-05	0.096595649	95	1.66E-07	4.01E-12	GTEx/v8/Brain_Caudate_basal_ganglia:GTEx/v8/Brain_Cerebellar_Hemisphere:GTEx/v8/Brain_Cortex
rs7805419	ENSG00000106460	*TMEM106B*	7	12250867	12282993	1	protein_coding	54664	TMEM106B	0.515078464	3.380649935	115	2.12E-07	0.00109844	GTEx/v8/Brain_Cerebellum:GTEx/v8/Brain_Cortex
rs62491417	ENSG00000006459	*KDM7A*	7	139784546	139876835	-1	protein_coding	80853	KDM7A	0.99869438	-1.580266986	2	1.06E-17	3.67E-14	GTEx/v8/Brain_Caudate_basal_ganglia:GTEx/v8/Brain_Cerebellar_Hemisphere:GTEx/v8/Brain_Cerebellum:GTEx/v8/Brain_Cortex:GTEx/v8/Brain_Frontal_Cortex_BA9:GTEx/v8/Brain_Hippocampus:GTEx/v8/Brain_Nucleus_accumbens_basal_ganglia:GTEx/v8/Brain_Putamen_basal_ganglia:GTEx/v8/Brain_Spinal_cord_cervical_c-1

rs6464217	ENSG00000157764	*BRAF*	7	140419127	140624564	-1	protein_coding	673	BRAF	0.999978196	NA	1	2.80E-05	0.0377877	GTEx/v8/Brain_Cerebellum
rs6464217	ENSG00000090263	*MRPS33*	7	140705854	140715028	-1	protein_coding	51650	MRPS33	0.003395718	0.268392925	51	2.30E-09	1.80E-05	GTEx/v8/Brain_Caudate_basal_ganglia:GTEx/v8/Brain_Cortex:GTEx/v8/Brain_Putamen_basal_ganglia
rs2009619	ENSG00000261451	*RP11-981G7.1*	8	10291182	10295822	1	sense_overlapping	NA	NA	NA	NA	23	3.79E-06	3.66E-17	GTEx/v8/Brain_Cerebellar_Hemisphere
rs16884419	ENSG00000156103	*MMP16*	8	89044237	89340254	-1	protein_coding	4325	MMP16	0.916974084	-3.432432894	95	1.36E-05	0.0179096	GTEx/v8/Brain_Cerebellum
rs2149351	ENSG00000233569	*RP11-500B12.1*	9	120410884	120419305	1	lincRNA	101928797	LOC101928797	NA	NA	2	4.01E-06	2.85E-14	GTEx/v8/Brain_Cerebellum
rs2399576	ENSG00000225383	*SFTA1P*	10	10826400	10836943	-1	lincRNA	207107	SFTA1P	NA	NA	37	1.00E-12	4.87E-11	GTEx/v8/Brain_Cortex:GTEx/v8/Brain_Frontal_Cortex_BA9:GTEx/v8/Brain_Hippocampus:GTEx/v8/Brain_Hypothalamus:GTEx/v8/Brain_Nucleus_accumbens_basal_ganglia:GTEx/v8/Brain_Putamen_basal_ganglia:GTEx/v8/Brain_Spinal_cord_cervical_c-1
rs499188	ENSG00000149150	*SLC43A1*	11	57252007	57283259	-1	protein_coding	8501	SLC43A1	7.85E-05	-0.136928307	5	4.46E-05	2.48E-08	GTEx/v8/Brain_Cerebellum
rs499188	ENSG00000134809	*TIMM10*	11	57295936	57298276	-1	protein_coding	26519	TIMM10	0.009494762	0.000100078	15	1.68E-05	7.82E-13	GTEx/v8/Brain_Caudate_basal_ganglia
rs499188	ENSG00000156603	*MED19*	11	57471186	57479693	-1	protein_coding	219541	MED19	0.683790051	-0.151700149	83	5.44E-06	5.15E-07	GTEx/v8/Brain_Cerebellar_Hemisphere:GTEx/v8/Brain_Cerebellum
rs499188	ENSG00000242689	*CNTF*	11	58390146	58393198	1	protein_coding	1270	CNTF	0.000349252	-0.109964842	48	1.60E-05	0.0192174	GTEx/v8/Brain_Cortex
rs102275	ENSG00000134825	*TMEM258*	11	61535973	61560274	-1	protein_coding	746	TMEM258	0.000864646	0.122739186	41	1.36E-06	0.00433323	GTEx/v8/Brain_Frontal_Cortex_BA9:GTEx/v8/Brain_Putamen_basal_ganglia
rs102275	ENSG00000149485	*FADS1*	11	61567099	61596790	-1	protein_coding	3992	FADS1	0.383801127	-0.763752724	55	7.63E-33	3.14E-26	GTEx/v8/Brain_Anterior_cingulate_cortex_BA24:GTEx/v8/Brain_Caudate_basal_ganglia:GTEx/v8/Brain_Cerebellar_Hemisphere:GTEx/v8/Brain_Cerebellum:GTEx/v8/Brain_Cortex:GTEx/v8/Brain_Frontal_Cortex_BA9:GTEx/v8/Brain_Hippocampus:GTEx/v8/Brain_Hypothalamus:GTEx/v8/Brain_Putamen_basal_ganglia:GTEx/v8/Brain_Spinal_cord_cervical_c-1
rs102275	ENSG00000221968	*FADS3*	11	61640991	61659523	-1	protein_coding	3995	FADS3	0.048872245	NA	50	1.79E-11	1.73E-07	GTEx/v8/Brain_Cerebellar_Hemisphere:GTEx/v8/Brain_Cerebellum
rs6168745	ENSG00000149295	*DRD2*	11	113280318	113346413	-1	protein_coding	1813	DRD2	0.733263889	NA	7	1.95E-06	0.00562986	GTEx/v8/Brain_Cerebellar_Hemisphere
rs6168745	ENSG00000166682	*TMPRSS5*	11	113558272	113577095	-1	protein_coding	80975	TMPRSS5	2.58E-08	0.18379421	2	2.13E-05	7.17E-27	GTEx/v8/Brain_Caudate_basal_ganglia:GTEx/v8/Brain_Putamen_basal_ganglia
rs612823	ENSG00000080854	*IGSF9B*	11	133778459	133826880	-1	protein_coding	22997	IGSF9B	0.999873394	NA	1	6.52E-09	5.01E-09	GTEx/v8/Brain_Cerebellum
rs7935549	ENSG00000255545	*RP11-627G23.1*	11	134306367	134375555	1	processed_transcript	NA	NA	NA	NA	1	1.12E-05	2.98E-30	GTEx/v8/Brain_Frontal_Cortex_BA9
rs3742020	ENSG00000139428	*MMAB*	12	109991542	110011679	-1	protein_coding	326625	MMAB	0.00387716	2.918192677	17	3.02E-07	5.53E-08	GTEx/v8/Brain_Caudate_basal_ganglia:GTEx/v8/Brain_Cortex:GTEx/v8/Brain_Frontal_Cortex_BA9:GTEx/v8/Brain_Nucleus_accumbens_basal_ganglia
rs11068917	ENSG00000089220	*PEBP1*	12	118573663	118583389	1	protein_coding	5037	PEBP1	0.148864265	-0.129913998	1	6.41E-07	4.53E-12	GTEx/v8/Brain_Nucleus_accumbens_basal_ganglia:GTEx/v8/Brain_Putamen_basal_ganglia
rs11068917	ENSG00000111707	*SUDS3*	12	118814185	118855840	1	protein_coding	64426	SUDS3	0.007596107	1.08228265	232	1.04E-06	0.00533451	GTEx/v8/Brain_Cortex:GTEx/v8/Brain_Hippocampus:GTEx/v8/Brain_Nucleus_accumbens_basal_ganglia:GTEx/v8/Brain_Putamen_basal_ganglia
rs4903249	ENSG00000119682	*AREL1*	14	75120140	75179818	-1	protein_coding	9870	AREL1	0.011802084	-0.560094839	14	7.29E-07	1.08E-05	GTEx/v8/Brain_Frontal_Cortex_BA9
rs12886000	ENSG00000246223	*C14orf64*	14	98391947	98444461	-1	protein_coding	388011	C14orf64	NA	NA	7	1.20E-07	0.000908772	GTEx/v8/Brain_Caudate_basal_ganglia
rs942866	ENSG00000075413	*MARK3*	14	103851729	103970168	1	protein_coding	4140	MARK3	6.11E-05	-0.099970289	3	3.01E-05	0.000395216	GTEx/v8/Brain_Frontal_Cortex_BA9
rs942866	ENSG00000166166	*TRMT61A*	14	103995521	104003410	1	protein_coding	115708	TRMT61A	0.005702065	0.11345694	53	7.22E-09	8.83E-05	GTEx/v8/Brain_Cortex:GTEx/v8/Brain_Frontal_Cortex_BA9
rs942866	ENSG00000258851	*RP11-894P9.2*	14	104019758	104028214	1	antisense	NA	NA	NA	NA	4	1.52E-05	0.0303807	GTEx/v8/Brain_Cerebellar_Hemisphere
rs942866	ENSG00000166170	*BAG5*	14	104022881	104029168	-1	protein_coding	9529	BAG5	0.043218188	-0.768926857	40	7.54E-07	0.00184945	GTEx/v8/Brain_Anterior_cingulate_cortex_BA24:GTEx/v8/Brain_Cerebellar_Hemisphere:GTEx/v8/Brain_Cerebellum:GTEx/v8/Brain_Hypothalamus
rs942866	ENSG00000126214	*KLC1*	14	104028233	104167888	1	protein_coding	3831	KLC1	0.514955332	NA	43	7.67E-08	3.20E-05	GTEx/v8/Brain_Cerebellar_Hemisphere:GTEx/v8/Brain_Cortex

rs942866	ENSG00000256053	*APOPT1*	14	104029299	104073860	1	protein_coding	84334	APOPT1	0.000361376	-0.703676152	17	1.83E-05	1.30E-07	GTEx/v8/Brain_Frontal_Cortex_BA9:GTEx/v8/Brain_Nucleus_accumbens_basal_ganglia
rs942866	ENSG00000269958	*RP11-73M18.8*	14	104162690	104163500	1	sense_intronic	NA	NA	NA	NA	54	3.12E-10	5.82E-08	GTEx/v8/Brain_Amygdala:GTEx/v8/Brain_Caudate_basal_ganglia:GTEx/v8/Brain_Cortex:GTEx/v8/Brain_Frontal_Cortex_BA9:GTEx/v8/Brain_Hippocampus:GTEx/v8/Brain_Hypothalamus:GTEx/v8/Brain_Nucleus_accumbens_basal_ganglia:GTEx/v8/Brain_Putamen_basal_ganglia:GTEx/v8/Brain_Spinal_cord_cervical_c-1:GTEx/v8/Brain_Substantia_nigra
rs942866	ENSG00000126215	*XRCC3*	14	104163946	104181841	-1	protein_coding	7517	XRCC3	6.32E-08	0.467663173	43	9.53E-09	1.25E-09	GTEx/v8/Brain_Cerebellar_Hemisphere
rs921764	ENSG00000137872	*SEMA6D*	15	47476298	48066420	1	protein_coding	80031	SEMA6D	0.999469976	-0.259466766	75	7.50E-08	2.85E-05	GTEx/v8/Brain_Cerebellum
rs2054213	ENSG00000196118	*C16orf93*	16	30768744	30774031	-1	protein_coding	90835	C16orf93	8.00E-05	-0.754318822	102	1.59E-13	2.99E-15	GTEx/v8/Brain_Caudate_basal_ganglia:GTEx/v8/Brain_Cortex:GTEx/v8/Brain_Frontal_Cortex_BA9:GTEx/v8/Brain_Hippocampus:GTEx/v8/Brain_Hypothalamus:GTEx/v8/Brain_Nucleus_accumbens_basal_ganglia
rs2054213	ENSG00000103549	*RNF40*	16	30773066	30787628	1	protein_coding	9810	RNF40	0.999994909	-1.913783614	25	4.02E-09	1.74E-05	GTEx/v8/Brain_Caudate_basal_ganglia:GTEx/v8/Brain_Cerebellar_Hemisphere:GTEx/v8/Brain_Cerebellum:GTEx/v8/Brain_Nucleus_accumbens_basal_ganglia:GTEx/v8/Brain_Putamen_basal_ganglia
rs2054213	ENSG00000099381	*SETD1A*	16	30968615	30996437	1	protein_coding	9739	SETD1A	0.999996231	-0.894969313	57	3.17E-06	0.00390586	GTEx/v8/Brain_Cerebellum
rs2054213	ENSG00000099377	*HSD3B7*	16	30996519	31000473	1	protein_coding	80270	HSD3B7	4.64E-06	-0.867416987	94	1.96E-06	8.50E-05	GTEx/v8/Brain_Anterior_cingulate_cortex_BA24:GTEx/v8/Brain_Cortex:GTEx/v8/Brain_Hypothalamus
rs2054213	ENSG00000099365	*STX1B*	16	31000577	31021949	-1	protein_coding	112755	STX1B	0.944474733	-1.610450086	106	5.01E-07	0.000791194	GTEx/v8/Brain_Cerebellar_Hemisphere:GTEx/v8/Brain_Cerebellum
rs2054213	ENSG00000103496	*STX4*	16	31044210	31054296	1	protein_coding	6810	STX4	9.66E-05	-0.421867635	137	1.18E-12	1.78E-08	GTEx/v8/Brain_Anterior_cingulate_cortex_BA24:GTEx/v8/Brain_Cerebellum:GTEx/v8/Brain_Cortex:GTEx/v8/Brain_Frontal_Cortex_BA9:GTEx/v8/Brain_Hypothalamus:GTEx/v8/Brain_Nucleus_accumbens_basal_ganglia
rs2054213	ENSG00000260911	*RP11-196G11.2*	16	31054471	31061201	1	lincRNA	NA	NA	NA	NA	137	1.45E-26	3.33E-21	GTEx/v8/Brain_Anterior_cingulate_cortex_BA24:GTEx/v8/Brain_Caudate_basal_ganglia:GTEx/v8/Brain_Cerebellar_Hemisphere:GTEx/v8/Brain_Cerebellum:GTEx/v8/Brain_Cortex:GTEx/v8/Brain_Frontal_Cortex_BA9:GTEx/v8/Brain_Hippocampus:GTEx/v8/Brain_Nucleus_accumbens_basal_ganglia:GTEx/v8/Brain_Putamen_basal_ganglia:GTEx/v8/Brain_Spinal_cord_cervical_c-1:GTEx/v8/Brain_Substantia_nigra
rs2054213	ENSG00000167395	*ZNF646*	16	31085743	31095517	1	protein_coding	9726	ZNF646	0.00556536	0.564563942	75	1.54E-06	0.00486533	GTEx/v8/Brain_Caudate_basal_ganglia:GTEx/v8/Brain_Hippocampus
rs2054213	ENSG00000167397	*VKORC1*	16	31102163	31107301	-1	protein_coding	79001	VKORC1	0.081803076	-0.611112737	92	1.23E-06	0.000396364	GTEx/v8/Brain_Caudate_basal_ganglia:GTEx/v8/Brain_Cortex
rs2054213	ENSG00000103510	*KAT8*	16	31127075	31142714	1	protein_coding	84148	KAT8	0.912576388	0.037392946	137	1.20E-12	3.51E-17	GTEx/v8/Brain_Caudate_basal_ganglia:GTEx/v8/Brain_Cerebellar_Hemisphere:GTEx/v8/Brain_Cerebellum:GTEx/v8/Brain_Cortex:GTEx/v8/Brain_Frontal_Cortex_BA9:GTEx/v8/Brain_Hippocampus:GTEx/v8/Brain_Hypothalamus:GTEx/v8/Brain_Nucleus_accumbens_basal_ganglia:GTEx/v8/Brain_Putamen_basal_ganglia
rs2054213	ENSG00000262766	*RP11-196G11.4*	16	31129399	31130068	1	sense_intronic	NA	NA	NA	NA	12	2.78E-05	0.000263052	GTEx/v8/Brain_Cerebellum
rs2054213	ENSG00000178226	*PRSS36*	16	31150246	31161415	-1	protein_coding	146547	PRSS36	4.42E-13	-0.151362733	126	1.72E-11	1.01E-22	GTEx/v8/Brain_Amygdala:GTEx/v8/Brain_Anterior_cingulate_cortex_BA24:GTEx/v8/Brain_Caudate_basal_ganglia:GTEx/v8/Brain_Cerebellar_Hemisphere:GTEx/v8/Brain_Cerebellum:GTEx/v8/Brain_Cortex:GTEx/v8/Brain_Frontal_Cortex_BA9:GTEx/v8/Brain_Hippocampus:GTEx/v8/Brain_Hypothalamus:GTEx/v8/Brain_Nucleus_accumbens_basal_ganglia:GTEx/v8/Brain_Putamen_basal_ganglia
rs1424144	ENSG00000180917	*CMTR2*	16	71315292	71323618	-1	protein_coding	55783	CMTR2	1.42E-07	-0.541219806	39	4.50E-06	0.0096857	GTEx/v8/Brain_Hypothalamus
rs111504162	ENSG00000224888	*RP5-1142A6.2*	16	88797588	88807826	1	antisense	NA	NA	NA	NA	2	1.68E-06	3.46E-11	GTEx/v8/Brain_Anterior_cingulate_cortex_BA24
rs499188	ENSG00000265566	*RN7SL605P*	17	2657126	2657430	1	misc_RNA	106481087	RN7SL605P	NA	NA	1	0.000105864	5.83E-09	GTEx/v8/Brain_Frontal_Cortex_BA9
rs1120442 1	ENSG00000270091	*RP11-78O7.2*	17	19799903	19800600	-1	lincRNA	NA	NA	NA	NA	67	1.61E-12	1.71E-08	GTEx/v8/Brain_Caudate_basal_ganglia:GTEx/v8/Brain_Cerebellar_Hemisphere:GTEx/v8/Brain_Cerebellum:GTEx/v8/Brain_Cortex:GTEx/v8/Brain_Frontal_Cortex_BA9:GTEx/v8/Brain_Putamen_basal_ganglia
rs11204421	ENSG00000128487	*SPECC1*	17	19912657	20222339	1	protein_coding	92521	SPECC1	0.10945964	0.008122998	24	1.89E-07	3.13E-05	GTEx/v8/Brain_Cortex
rs11204421	ENSG00000154898	*CCDC144CP*	17	20224477	20306870	1	pseudogene	348254	CCDC144CP	NA	NA	67	2.66E-08	2.02E-05	GTEx/v8/Brain_Cerebellum:GTEx/v8/Brain_Cortex
rs11204421	ENSG00000189423	*USP32P3*	17	20318860	20334321	1	pseudogene	347716	USP32P3	NA	NA	62	1.68E-06	8.09E-05	GTEx/v8/Brain_Cortex
rs11204421	ENSG00000237911	*SRP68P3*	17	20319105	20320010	-1	pseudogene	347717	SRP68P3	NA	NA	24	3.79E-08	3.68E-06	GTEx/v8/Brain_Cerebellar_Hemisphere:GTEx/v8/Brain_Cerebellum
rs11204421	ENSG00000230528	*NOS2P3*	17	20339650	20350557	1	pseudogene	339256	NOS2P3	NA	NA	34	4.51E-09	1.23E-17	GTEx/v8/Brain_Anterior_cingulate_cortex_BA24:GTEx/v8/Brain_Cerebellar_Hemisphere:GTEx/v8/Brain_Cerebellum:GTEx/v8/Brain_Cortex:GTEx/v8/Brain_Frontal_Cortex_BA9:GTEx/v8/Brain_Hippocampus:GTEx/v8/Brain_Nucleus_accumbens_basal_ganglia
rs11204421	ENSG00000231645	*AC025627.9*	17	20415873	20420792	-1	pseudogene	NA	NA	NA	NA	14	3.62E-06	0.00671679	GTEx/v8/Brain_Cerebellar_Hemisphere

rs11204421	ENSG00000233098	*RP11-344E13.3*	17	20771746	20905589	1	antisense	NA	NA	NA	NA	1	5.88E-05	0.00138367	GTEx/v8/Brain_Nucleus_accumbens_basal_ganglia
rs11317362	ENSG00000136448	*NMT1*	17	43128978	43186384	1	protein_coding	4836	NMT1	0.998500521	0.032447683	1801	9.33E-06	2.39E-07	GTEx/v8/Brain_Cerebellar_Hemisphere:GTEx/v8/Brain_Cerebellum:GTEx/v8/Brain_Spinal_cord_cervical_c-1
rs11317362	ENSG00000267121	*CTD-2020K17.1*	17	43268298	43299589	-1	antisense	339192	LOC339192	NA	NA	2668	6.12E-11	4.50E-07	GTEx/v8/Brain_Cerebellar_Hemisphere:GTEx/v8/Brain_Cerebellum
rs11317362	ENSG00000184922	*FMNL1*	17	43298811	43324687	1	protein_coding	752	FMNL1	0.999687036	NA	2668	1.02E-24	7.89E-19	GTEx/v8/Brain_Cerebellar_Hemisphere:GTEx/v8/Brain_Cerebellum
rs11317362	ENSG00000233175	*CTD-2020K17.3*	17	43315395	43319101	-1	antisense	107985040	LOC107985040	NA	NA	21	9.72E-05	2.69E-16	GTEx/v8/Brain_Cerebellum
rs11317362	ENSG00000006062	*MAP3K14*	17	43340488	43394414	-1	processed_transcript	9020	MAP3K14	NA	NA	2	0.000176915	0.00353334	GTEx/v8/Brain_Cerebellum
rs11317362	ENSG00000159314	*ARHGAP27*	17	43471275	43511787	-1	protein_coding	201176	ARHGAP27	0.001255844	-0.761627793	2806	4.59E-19	7.23E-14	GTEx/v8/Brain_Caudate_basal_ganglia:GTEx/v8/Brain_Cerebellar_Hemisphere:GTEx/v8/Brain_Cerebellum:GTEx/v8/Brain_Cortex:GTEx/v8/Brain_Nucleus_accumbens_basal_ganglia:GTEx/v8/Brain_Putamen_basal_ganglia
rs11317362	ENSG00000225190	*PLEKHM1*	17	43513266	43568115	-1	protein_coding	9842	PLEKHM1	0.84054321	-0.66306803 2	2806	1.98E-42	8.80E-35	GTEx/v8/Brain_Amygdala:GTEx/v8/Brain_Anterior_cingulate_cortex_BA24:GTEx/v8/Brain_Caudate_basal_ganglia:GTEx/v8/Brain_Cerebellar_Hemisphere:GTEx/v8/Brain_Cerebellum:GTEx/v8/Brain_Cortex:GTEx/v8/Brain_Frontal_Cortex_BA9:GTEx/v8/Brain_Hypothalamus:GTEx/v8/Brain_Nucleus_accumbens_basal_ganglia:GTEx/v8/Brain_Putamen_basal_ganglia
rs11317362	ENSG00000236234	*AC091132.1*	17	43530210	43541431	1	antisense	NA	NA	NA	NA	2806	2.17E-12	2.29E-08	GTEx/v8/Brain_Cerebellar_Hemisphere:GTEx/v8/Brain_Cerebellum
rs11317362	ENSG00000214425	*LRRC37A4P*	17	43578685	43627701	-1	pseudogene	55073	LRRC37A4P	NA	NA	2808	1.81E-58	2.69E-48	GTEx/v8/Brain_Amygdala:GTEx/v8/Brain_Anterior_cingulate_cortex_BA24:GTEx/v8/Brain_Caudate_basal_ganglia:GTEx/v8/Brain_Cerebellar_Hemisphere:GTEx/v8/Brain_Cerebellum:GTEx/v8/Brain_Cortex:GTEx/v8/Brain_Frontal_Cortex_BA9:GTEx/v8/Brain_Hippocampus:GTEx/v8/Brain_Hypothalamus:GTEx/v8/Brain_Nucleus_accumbens_basal_ganglia:GTEx/v8/Brain_Putamen_basal_ganglia:GTEx/v8/Brain_Spinal_cord_cervical_c-1:GTEx/v8/Brain_Substantia_nigra
rs11317362	ENSG00000266918	*RP11-798G7.8*	17	43608943	43611204	1	lincRNA	NA	NA	NA	NA	2806	2.14E-16	6.68E-12	GTEx/v8/Brain_Amygdala:GTEx/v8/Brain_Caudate_basal_ganglia:GTEx/v8/Brain_Cerebellar_Hemisphere:GTEx/v8/Brain_Cerebellum:GTEx/v8/Brain_Cortex
rs11317362	ENSG00000267198	*RP11-798G7.6*	17	43623170	43640596	1	lincRNA	NA	NA	NA	NA	2606	5.49E-06	1.37E-08	GTEx/v8/Brain_Cerebellum
rs11317362	ENSG00000264070	*DND1P1*	17	43663237	43664295	1	pseudogene	644157	DND1P1	NA	NA	2807	7.80E-38	1.42E-31	GTEx/v8/Brain_Amygdala:GTEx/v8/Brain_Anterior_cingulate_cortex_BA24:GTEx/v8/Brain_Caudate_basal_ganglia:GTEx/v8/Brain_Cerebellar_Hemisphere:GTEx/v8/Brain_Cerebellum:GTEx/v8/Brain_Cortex:GTEx/v8/Brain_Frontal_Cortex_BA9:GTEx/v8/Brain_Hippocampus:GTEx/v8/Brain_Hypothalamus:GTEx/v8/Brain_Nucleus_accumbens_basal_ganglia:GTEx/v8/Brain_Putamen_basal_ganglia:GTEx/v8/Brain_Spinal_cord_cervical_c-1:GTEx/v8/Brain_Substantia_nigra
rs11317362	ENSG00000263503	*RP11-707O23.5*	17	43678235	43679706	-1	pseudogene	NA	NA	NA	NA	2807	1.33E-41	1.26E-35	GTEx/v8/Brain_Amygdala:GTEx/v8/Brain_Anterior_cingulate_cortex_BA24:GTEx/v8/Brain_Caudate_basal_ganglia:GTEx/v8/Brain_Cerebellar_Hemisphere:GTEx/v8/Brain_Cerebellum:GTEx/v8/Brain_Cortex:GTEx/v8/Brain_Frontal_Cortex_BA9:GTEx/v8/Brain_Hippocampus:GTEx/v8/Brain_Hypothalamus:GTEx/v8/Brain_Nucleus_accumbens_basal_ganglia:GTEx/v8/Brain_Putamen_basal_ganglia:GTEx/v8/Brain_Spinal_cord_cervical_c-1:GTEx/v8/Brain_Substantia_nigra
rs11317362	ENSG00000204650	*CRHR1-IT1*	17	43697694	43725582	1	pseudogene	147081	CRHR1-IT1	NA	NA	2808	1.06E-38	1.09E-30	GTEx/v8/Brain_Amygdala:GTEx/v8/Brain_Anterior_cingulate_cortex_BA24:GTEx/v8/Brain_Caudate_basal_ganglia:GTEx/v8/Brain_Cerebellar_Hemisphere:GTEx/v8/Brain_Cerebellum:GTEx/v8/Brain_Cortex:GTEx/v8/Brain_Frontal_Cortex_BA9:GTEx/v8/Brain_Hippocampus:GTEx/v8/Brain_Hypothalamus:GTEx/v8/Brain_Nucleus_accumbens_basal_ganglia:GTEx/v8/Brain_Putamen_basal_ganglia:GTEx/v8/Brain_Spinal_cord_cervical_c-1:GTEx/v8/Brain_Substantia_nigra
rs11317362	ENSG00000120088	*CRHR1*	17	43699267	43913194	1	protein_coding	1394	CRHR1	0.792134286	2.038085631	2789	4.29E-06	0.00117547	GTEx/v8/Brain_Caudate_basal_ganglia:GTEx/v8/Brain_Cortex:GTEx/v8/Brain_Hippocampus:GTEx/v8/Brain_Nucleus_accumbens_basal_ganglia:GTEx/v8/Brain_Putamen_basal_ganglia
rs11317362	ENSG00000264589	*MAPT-AS1*	17	43921017	43972966	-1	antisense	100128977	MAPT-AS1	NA	NA	2820	1.07E-21	3.37E-16	GTEx/v8/Brain_Caudate_basal_ganglia:GTEx/v8/Brain_Cerebellar_Hemisphere:GTEx/v8/Brain_Cerebellum:GTEx/v8/Brain_Hippocampus:GTEx/v8/Brain_Hypothalamus:GTEx/v8/Brain_Nucleus_accumbens_basal_ganglia:GTEx/v8/Brain_Putamen_basal_ganglia:GTEx/v8/Brain_Spinal_cord_cervical_c-1:GTEx/v8/Brain_Substantia_nigra
rs11317362	ENSG00000185294	*SPPL2C*	17	43922256	43924438	1	protein_coding	162540	SPPL2C	2.30E-05	0.187394842	2820	6.65E-25	5.44E-19	GTEx/v8/Brain_Cerebellar_Hemisphere:GTEx/v8/Brain_Cerebellum:GTEx/v8/Brain_Cortex:GTEx/v8/Brain_Frontal_Cortex_BA9
rs11317362	ENSG00000186868	*MAPT*	17	43971748	44105700	1	protein_coding	4137	MAPT	4.68E-05	2.01024275	2808	1.10E-07	7.42E-05	GTEx/v8/Brain_Caudate_basal_ganglia:GTEx/v8/Brain_Cerebellar_Hemisphere:GTEx/v8/Brain_Cerebellum
rs11317362	ENSG00000120071	*KANSL1*	17	44107282	44302733	-1	protein_coding	284058	KANSL1	0.999733506	0.058945245	2813	1.32E-06	1.09E-09	GTEx/v8/Brain_Caudate_basal_ganglia:GTEx/v8/Brain_Cerebellar_Hemisphere:GTEx/v8/Brain_Cerebellum:GTEx/v8/Brain_Frontal_Cortex_BA9
rs11317362	ENSG00000214401	*KANSL1-AS1*	17	44270942	44274089	1	antisense	644246	KANSL1-AS1	NA	NA	2820	4.51E-52	1.22E-43	GTEx/v8/Brain_Amygdala:GTEx/v8/Brain_Anterior_cingulate_cortex_BA24:GTEx/v8/Brain_Caudate_basal_ganglia:GTEx/v8/Brain_Cerebellar_Hemisphere:GTEx/v8/Brain_Cerebellum:GTEx/v8/Brain_Cortex:GTEx/v8/Brain_Frontal_Cortex_BA9:GTEx/v8/Brain_Hippocampus:GTEx/v8/Brain_Hypothalamus:GTEx/v8/Brain_Nucleus_accumbens_basal_ganglia:GTEx/v8/Brain_Putamen_basal_ganglia:GTEx/v8/Brain_Spinal_cord_cervical_c-1:GTEx/v8/Brain_Substantia_nigra
rs11317362	ENSG00000262500	*RP11-259G18.2*	17	44320972	44322410	1	pseudogene	NA	NA	NA	NA	2820	2.61E-40	5.94E-34	GTEx/v8/Brain_Amygdala:GTEx/v8/Brain_Anterior_cingulate_cortex_BA24:GTEx/v8/Brain_Caudate_basal_ganglia:GTEx/v8/Brain_Cerebellar_Hemisphere:GTEx/v8/Brain_Cerebellum:GTEx/v8/Brain_Cortex:GTEx/v8/Brain_Frontal_Cortex_BA9:GTEx/v8/Brain_Hippocampus:GTEx/v8/Brain_Hypothalamus:GTEx/v8/Brain_Nucleus_accumbens_basal_ganglia:GTEx/v8/Brain_Putamen_basal_ganglia:GTEx/v8/Brain_Spinal_cord_cervical_c-1:GTEx/v8/Brain_Substantia_nigra
rs11317362	ENSG00000262539	*RP11-259G18.3*	17	44336917	44337972	-1	pseudogene	NA	NA	NA	NA	2821	3.51E-45	1.60E-37	GTEx/v8/Brain_Amygdala:GTEx/v8/Brain_Anterior_cingulate_cortex_BA24:GTEx/v8/Brain_Caudate_basal_ganglia:GTEx/v8/Brain_Cerebellar_Hemisphere:GTEx/v8/Brain_Cerebellum:GTEx/v8/Brain_Cortex:GTEx/v8/Brain_Frontal_Cortex_BA9:GTEx/v8/Brain_Hippocampus:GTEx/v8/Brain_Hypothalamus:GTEx/v8/Brain_Nucleus_accumbens_basal_ganglia:GTEx/v8/Brain_Putamen_basal_ganglia:GTEx/v8/Brain_Spinal_cord_cervical_c-1:GTEx/v8/Brain_Substantia_nigra
rs11317362	ENSG00000261575	*RP11-259G18.1*	17	44344403	44346060	1	pseudogene	NA	NA	NA	NA	2821	7.46E-51	1.13E-41	GTEx/v8/Brain_Amygdala:GTEx/v8/Brain_Anterior_cingulate_cortex_BA24:GTEx/v8/Brain_Caudate_basal_ganglia:GTEx/v8/Brain_Cerebellar_Hemisphere:GTEx/v8/Brain_Cerebellum:GTEx/v8/Brain_Cortex:GTEx/v8/Brain_Frontal_Cortex_BA9:GTEx/v8/Brain_Hippocampus:GTEx/v8/Brain_Hypothalamus:GTEx/v8/Brain_Nucleus_accumbens_basal_ganglia:GTEx/v8/Brain_Putamen_basal_ganglia:GTEx/v8/Brain_Spinal_cord_cervical_c-1:GTEx/v8/Brain_Substantia_nigra
rs11317362	ENSG00000228696	*ARL17B*	17	44352150	44439130	-1	protein_coding	100506084	ARL17B	NA	-0.428243465	8	8.50E-08	2.36E-17	GTEx/v8/Brain_Cerebellar_Hemisphere:GTEx/v8/Brain_Cerebellum:GTEx/v8/Brain_Hypothalamus
rs11317362	ENSG00000176681	*LRRC37A*	17	44370099	44415160	1	protein_coding	9884	LRRC37A	0.890487356	NA	2820	1.21E-30	4.87E-42	GTEx/v8/Brain_Amygdala:GTEx/v8/Brain_Anterior_cingulate_cortex_BA24:GTEx/v8/Brain_Caudate_basal_ganglia:GTEx/v8/Brain_Cerebellar_Hemisphere:GTEx/v8/Brain_Cerebellum:GTEx/v8/Brain_Cortex:GTEx/v8/Brain_Frontal_Cortex_BA9:GTEx/v8/Brain_Hippocampus:GTEx/v8/Brain_Hypothalamus:GTEx/v8/Brain_Nucleus_accumbens_basal_ganglia:GTEx/v8/Brain_Putamen_basal_ganglia:GTEx/v8/Brain_Spinal_cord_cervical_c-1:GTEx/v8/Brain_Substantia_nigra
rs11317362	ENSG00000238083	*LRRC37A2*	17	44588877	44633016	1	protein_coding	474170	LRRC37A2	NA	NA	2674	2.68E-52	7.91E-44	GTEx/v8/Brain_Amygdala:GTEx/v8/Brain_Anterior_cingulate_cortex_BA24:GTEx/v8/Brain_Caudate_basal_ganglia:GTEx/v8/Brain_Cerebellar_Hemisphere:GTEx/v8/Brain_Cerebellum:GTEx/v8/Brain_Cortex:GTEx/v8/Brain_Frontal_Cortex_BA9:GTEx/v8/Brain_Hippocampus:GTEx/v8/Brain_Hypothalamus:GTEx/v8/Brain_Nucleus_accumbens_basal_ganglia:GTEx/v8/Brain_Putamen_basal_ganglia:GTEx/v8/Brain_Spinal_cord_cervical_c-1:GTEx/v8/Brain_Substantia_nigra
rs11317362	ENSG00000185829	*ARL17A*	17	44594068	44657088	-1	protein_coding	51326	ARL17A	NA	-2.38258287	2674	4.39E-38	7.42E-31	GTEx/v8/Brain_Amygdala:GTEx/v8/Brain_Anterior_cingulate_cortex_BA24:GTEx/v8/Brain_Caudate_basal_ganglia:GTEx/v8/Brain_Cerebellar_Hemisphere:GTEx/v8/Brain_Cerebellum:GTEx/v8/Brain_Cortex:GTEx/v8/Brain_Frontal_Cortex_BA9:GTEx/v8/Brain_Hippocampus:GTEx/v8/Brain_Hypothalamus:GTEx/v8/Brain_Nucleus_accumbens_basal_ganglia:GTEx/v8/Brain_Putamen_basal_ganglia
rs11317362	ENSG00000232300	*FAM215B*	17	44636196	44640161	-1	sense_intronic	644297	FAM215B	NA	NA	2674	6.29E-24	2.68E-18	GTEx/v8/Brain_Cerebellar_Hemisphere:GTEx/v8/Brain_Cerebellum

rs113173628	ENSG00000073969	*NSF*	17	44668035	44834830	1	protein_coding	4905	NSF	0.71368213	-0.566246769	2389	1.64E-06	2.14E-10	GTEx/v8/Brain_Cerebellum
rs113173628	ENSG00000108379	*WNT3*	17	44839872	44910520	-1	protein_coding	7473	WNT3	0.946108101	-0.191110949	14	2.52E-05	1.18E-51	GTEx/v8/Brain_Cortex
rs113173628	ENSG00000263142	*LRRC37A17P*	17	45055847	45131935	1	pseudogene	644397	LRRC37A17P	NA	NA	907	2.50E-05	0.0172168	GTEx/v8/Brain_Cerebellum
rs2628209	ENSG00000263720	*RP11-389J22.1*	18	63532498	63533633	1	sense_intronic	NA	NA	NA	NA	187	2.00E-25	4.84E-20	GTEx/v8/Brain_Cerebellar_Hemisphere:GTEx/v8/Brain_Cerebellum
rs13037664	ENSG00000101412	*E2F1*	20	32263489	32274210	-1	protein_coding	1869	E2F1	0.955530545	-0.324323693	1	4.18E-05	4.36E-14	GTEx/v8/Brain_Spinal_cord_cervical_c-1
rs13037664	ENSG00000198646	*NCOA6*	20	33284722	33413452	-1	protein_coding	23054	NCOA6	0.990815417	-0.852306051	90	2.48E-12	3.67E-08	GTEx/v8/Brain_Cerebellar_Hemisphere:GTEx/v8/Brain_Cerebellum:GTEx/v8/Brain_Cortex:GTEx/v8/Brain_Frontal_Cortex_BA9:GTEx/v8/Brain_Hippocampus:GTEx/v8/Brain_Hypothalamus:GTEx/v8/Brain_Nucleus_accumbens_basal_ganglia
rs9605069	ENSG00000099899	*TRMT2A*	22	20099389	20104915	-1	protein_coding	27037	TRMT2A	1.31E-05	NA	5	2.59E-05	0.0244377	GTEx/v8/Brain_Cerebellar_Hemisphere
rs9605069	ENSG00000099904	*ZDHHC8*	22	20116979	20135530	1	protein_coding	29801	ZDHHC8	0.988991096	NA	5	1.56E-05	0.0313939	GTEx/v8/Brain_Caudate_basal_ganglia
rs20551	ENSG00000128285	*MCHR1*	22	41074754	41078818	1	protein_coding	2847	MCHR1	0.001148393	-0.081006493	6	1.89E-05	8.11E-10	GTEx/v8/Brain_Cerebellum
rs20551	ENSG00000100372	*SLC25A17*	22	41165634	41215403	-1	protein_coding	10478	SLC25A17	0.024184844	-0.779478694	41	6.86E-06	2.58E-05	GTEx/v8/Brain_Cerebellum:GTEx/v8/Brain_Nucleus_accumbens_basal_ganglia:GTEx/v8/Brain_Putamen_basal_ganglia
rs20551	ENSG00000213857	*RP11-12M9.4*	22	41470184	41471243	-1	pseudogene	NA	NA	NA	NA	6	8.01E-05	0.0176512	GTEx/v8/Brain_Cerebellum
rs20551	ENSG00000100393	*EP300*	22	41487790	41576081	1	protein_coding	2033	EP300	1	NA	113	2.44E-06	0.00184947	GTEx/v8/Brain_Cerebellum:GTEx/v8/Brain_Hypothalamus
rs20551	ENSG00000100395	*L3MBTL2*	22	41601209	41627275	1	protein_coding	83746	L3MBTL2	0.013028043	-0.618259224	121	1.80E-08	5.18E-05	GTEx/v8/Brain_Anterior_cingulate_cortex_BA24:GTEx/v8/Brain_Frontal_Cortex_BA9
rs20551	ENSG00000100401	*RANGAP1*	22	41641615	41682255	-1	protein_coding	5905	RANGAP1	0.13271872	-0.376521602	76	7.87E-07	1.39E-06	GTEx/v8/Brain_Cerebellar_Hemisphere:GTEx/v8/Brain_Cerebellum
rs20551	ENSG00000100403	*ZC3H7B*	22	41697526	41756151	1	protein_coding	23264	ZC3H7B	0.990761551	-0.628157842	125	4.49E-08	0.00010307	GTEx/v8/Brain_Cerebellum
rs20551	ENSG00000100412	*ACO2*	22	41865129	41924993	1	protein_coding	50	ACO2	0.454377775	-0.617411984	21	2.91E-05	0.00290367	GTEx/v8/Brain_Cerebellum
rs20551	ENSG00000100413	*POLR3H*	22	41921808	41940610	-1	protein_coding	171568	POLR3H	0.002007106	-1.305852274	120	2.19E-08	8.24E-14	GTEx/v8/Brain_Cerebellum:GTEx/v8/Brain_Cortex:GTEx/v8/Brain_Frontal_Cortex_BA9:GTEx/v8/Brain_Putamen_basal_ganglia
rs20551	ENSG00000172346	*CSDC2*	22	41956767	41973745	1	protein_coding	27254	CSDC2	0.079803516	0.31935561	121	2.14E-09	6.71E-18	GTEx/v8/Brain_Cortex:GTEx/v8/Brain_Frontal_Cortex_BA9
rs20551	ENSG00000167077	*MEI1*	22	42095503	42195460	1	protein_coding	150365	MEI1	1.22E-08	0.500155337	115	1.71E-06	1.60E-05	GTEx/v8/Brain_Anterior_cingulate_cortex_BA24:GTEx/v8/Brain_Frontal_Cortex_BA9
rs20551	ENSG00000159958	*TNFRSF13C*	22	42321045	42322822	-1	protein_coding	115650	TNFRSF13C	0.641054688	-0.063981155	31	1.87E-05	1.22E-11	GTEx/v8/Brain_Cerebellar_Hemisphere
rs20551	ENSG00000205704	*LINC00634*	22	42348169	42354937	1	pseudogene	339674	LINC00634	NA	NA	9	6.19E-05	0.000116414	GTEx/v8/Brain_Frontal_Cortex_BA9
rs20551	ENSG00000183066	*WBP2NL*	22	42394729	42454460	1	protein_coding	164684	WBP2NL	2.07E-09	0.730692723	1	7.68E-05	1.04E-06	GTEx/v8/Brain_Frontal_Cortex_BA9
rs20551	ENSG00000198951	*NAGA*	22	42454358	42466846	-1	protein_coding	4668	NAGA	2.02E-06	0.183467033	6	8.98E-07	7.04E-29	GTEx/v8/Brain_Cerebellar_Hemisphere:GTEx/v8/Brain_Cerebellum
rs20551	ENSG00000100197	*CYP2D6*	22	42522501	42526908	-1	protein_coding	1565	CYP2D6	9.23E-10	0.153953666	12	2.97E-05	4.44E-35	GTEx/v8/Brain_Cerebellum:GTEx/v8/Brain_Nucleus_accumbens_basal_ganglia

Abbreviations: pLI, probability of loss of function intolerance; ncRVIS, non-coding residual variation intolerance score; eqtlMapSNPs, the number of SNPs mapped to the gene based on eQTL mapping; eqtlMapminP, mininum eQTL P value of mapped SNPs; eqtlMapminQ, mininum eQTL FDR of mapped SNPs; eQTL Mapts, tissue types of mapped eQTL SNPs (GTEx v8 Brain was selected).

Supplementary Table 20. Distinct genomic loci shared between irritability and each psychiatric disorder at conjFDR<0.05

a) Irritability and schizophrenia

Locusnum	CHR	Lead SNP	Lead BP	Mininum BP	Maximum BP	conjFDR
1	1	rs75460349	27180088	26933591	27368126	0.0151
2	1	rs11581846	35725203	35648492	36107510	0.0224
3	1	rs6679408	50591565	49463925	50591851	0.00147
4	1	rs954299	72121585	72090849	72191607	0.0495
5	1	rs4411173	98507718	98298371	98562260	0.0047
6	1	rs12757898	98873281	98861126	98913185	0.018
7	1	rs66867382	214494378	214469515	214564177	0.0269
8	1	rs2636324	243493484	243458922	243501763	0.0149
9	2	rs11680719	48657843	48178775	48750410	0.0192
10	2	rs2717032	58166760	57942987	58505679	0.000127
11	2	rs2867910	110397232	110283004	110415898	0.0434
12	2	rs12617922	146156679	146111968	146168208	0.0281
13	2	rs62176173	185499540	185406883	185530491	0.0316
14	2	rs12613014	194379926	194336245	194678448	0.0131
15	2	rs67735550	198229453	198144539	198954774	0.002553812
16	3	rs9882532	16865845	16843737	16944316	0.006001108
17	3	rs6769645	24168610	24166164	24185307	0.04937817
18	3	rs4688436	64229821	64178664	64243673	0.04712292
19	3	rs836927	107201428	107152293	107570882	0.007014383
20	3	rs9878792	116432671	116425935	116432671	0.01203297
21	4	rs57980547	84600736	84600736	84615059	0.04686343
22	4	rs59413629	108861655	108853384	108924557	0.04433994
23	5	rs1852599	45757305	45491939	46405067	0.01604732
24	5	rs28752150	107786142	107718510	107814966	0.04467993
25	5	rs6579956	152078663	152075050	152153689	0.005209077
26	5	rs62382095	153534563	153507705	153687444	0.003597288
27	6	rs704480	57091166	57091166	57146935	0.03194306
28	6	rs9374991	98372330	98228405	98777764	0.01045776

29	6	rs11154565	130609869	130544509	130772152	0.02750997
30	7	rs62442944	2015047	1873756	2239569	0.02090473
31	7	rs28541530	21531161	21470536	21552995	0.01814881
32	7	rs12704267	86206830	86199080	86336250	0.03197706
33	7	rs141272885	110290988	110290988	110340974	0.04633682
34	7	rs1229760	114224163	114015707	114290415	0.03382006
35	7	rs1593312	131584453	131535312	131606162	0.02266256
36	7	rs1669022	137061472	137039328	137081525	0.04167944
37	7	rs9800952	140669703	140665521	140791820	0.01294329
38	8	rs1986972	10268805	8218967	10332167	0.00368089
39	8	rs7002619	16065557	16007395	16086683	0.002462988
40	8	rs1043083	17087014	17062213	17087014	0.03457616
41	8	rs13264878	31406138	31397748	31437196	0.04763496
42	8	rs191124455	38239252	38014429	38310910	0.03425939
43	8	rs11786670	60846366	60586256	60883570	0.02639658
44	8	rs6472981	77592275	77588548	77695732	0.04525516
45	8	rs1700143	89480086	89206609	89761163	0.000434758
46	8	rs2721946	116641960	116597635	116645056	0.0370861
47	8	rs72722365	131190914	130853447	131361477	0.01762749
48	8	rs7835528	144243392	144238923	144270473	0.008191044
49	9	rs13296413	37258105	37073902	37379492	0.0230776
50	9	rs7044029	124690789	124681858	124741690	0.02484887
51	10	rs2225947	9671047	9651373	9963730	0.04013508
52	10	rs12416687	104629011	104546183	104963051	0.01911826
53	11	rs10835360	28606699	28591168	28694440	0.01771537
54	11	rs499188	57434122	57385856	57681828	0.02284536
55	11	rs61687445	113438092	113185591	113451229	0.01060903
56	11	rs612823	133834104	133834104	134252303	0.01365261
57	12	rs2159100	2346393	2297353	2408194	0.02250561
58	12	rs2467454	23062674	23013321	23078449	0.01720327
59	12	rs12582877	79670734	79552449	79713434	0.01231817
60	12	rs651548	99560183	99351017	99639930	0.02045895

61	12	rs35393419	109881809	109791620	110027795	0.008221861
62	12	rs10850805	117695594	117659623	117700293	0.03506796
63	13	rs3124429	55927523	55679499	56303709	0.01903029
64	13	rs7332097	111567902	111523068	111597758	0.03013654
65	13	rs6560933	114937294	114916023	114965548	0.007563777
66	14	rs8010744	29588428	29540867	29628625	0.04290949
67	14	rs8019784	47380711	47243109	47416511	0.03469868
68	14	rs941521	99708876	99665331	99751267	0.004450343
69	15	rs703235	56822403	56788798	56823913	0.04032973
70	16	rs12925028	13800509	13763820	13805809	0.04221695
71	16	rs113462046	24081586	24053202	24081586	0.02293472
72	16	rs117829446	30632657	30566747	30632657	0.02063232
73	16	rs8060690	68340085	68160289	68427217	0.04497325
74	16	rs17702834	69354682	69283995	69355229	0.04104698
75	16	rs17609347	72532362	72094348	72913858	0.03811652
76	17	rs17596918	11229208	11210835	11234195	0.03226927
77	17	rs62063686	44132458	43463493	44865603	0.004785566
78	17	rs9897752	78484082	78463585	78706517	0.001406955
79	18	rs7230285	50723080	50620087	50907127	0.03997853
80	18	rs56403421	52765283	52722378	53463661	0.001301724
81	19	rs73022878	30996923	30981639	31051857	0.01584017
82	19	rs34669859	33948890	33884411	34023996	0.01506295
83	20	rs6072230	39629538	39620847	39970434	0.02656422
84	22	rs8137258	20135961	20057078	20161605	0.001088926
85	22	rs20551	41548008	41085969	41854446	0.00053501

b) Irritability and bipolar I disorder

Locusnum	CHR	Lead SNP	Lead BP	Mininum BP	Maximum BP	conjFDR
1	1	rs9425311	172388778	172369347	172434812	0.0329
2	3	rs9874247	11095746	11090603	11096062	0.0384
3	3	rs73147228	85749950	85433248	85791383	0.0054
4	3	rs62261974	107296969	107152293	107570882	0.016

5	4	rs34626331	29379193	29344653	29430786	0.0273
6	4	rs2567375	101509208	101463177	101593148	0.0192
7	5	rs2431108	103947968	103783801	104082179	0.0239
8	7	rs56305291	2048706	1873756	2239569	0.00155
9	7	rs28541530	21531161	21470536	21552995	0.0222
10	7	rs6950909	131867984	131859817	131880252	0.0172
11	7	rs6464217	140691762	140434845	140791820	0.00582
12	8	rs57737477	10180328	8088230	10332167	0.00318
13	8	rs72652619	65085358	64744304	65266656	0.0399
14	9	rs56121711	37360811	37045825	37406391	0.015
15	10	rs12784757	9446213	9436362	9523803	0.0189
16	10	rs10905639	9955322	9842434	9963371	0.0189
17	10	rs1243184	21931937	21768560	22288132	0.0454
18	10	rs1613299	80954785	80939219	80958588	0.0397
19	10	rs12244388	104640052	104572081	104962011	0.0494
20	11	rs34862781	13305263	13268386	13350131	0.00501
21	11	rs174529	61543961	61542006	61624181	0.00177
22	11	rs11018471	88831103	88188047	89058101	0.00816
23	11	rs11223774	134247315	134235392	134252303	0.0331
24	12	rs2159100	2346393	2297353	2408194	0.0272
25	12	rs10783826	57854283	57853153	57870463	0.0444
26	12	rs651548	99560183	99436519	99639930	0.0248
27	13	rs9527083	53991125	53879428	54049489	0.039
28	14	rs8019575	25319866	25274272	25324716	0.00992
29	14	rs941521	99708876	99667179	99751267	0.00604
30	15	rs779	29856238	29848687	29858805	0.0457
31	15	rs12708450	56824086	56787709	56967631	0.0462
32	15	rs4886717	74165128	74109887	74165128	0.0345
33	16	rs12444455	18000389	17910733	18050926	0.0333
34	16	rs117961707	24083865	24030172	24083865	0.0485
35	16	rs1423988	64748469	64725769	64884995	0.0358
36	16	rs17702834	69354682	69283995	69355229	0.022

37	17	rs205024	11227352	11200279	11234195	0.0297
38	17	rs9897752	78484082	78463585	78706517	0.0049
39	18	rs55943003	52754086	52722378	52754086	0.0479
40	22	rs9606265	20137047	20046042	20161605	0.00356
41	22	rs80533	41085969	41085969	41215672	0.0426

c) Irritability and major depressive disorder

Locusnum	CHR	Lead SNP	Lead BP	Mininum BP	Maximum BP	conjFDR
1	1	rs12131717	8718085	8520487	8895970	0.01140091
2	1	rs78787495	21640387	21640387	21642935	0.04372206
3	1	rs114933496	33861277	33829731	33883883	0.02966981
4	1	rs568832	37237239	37219429	37261085	0.00857083
5	1	rs11211481	47694167	47659445	47708112	0.002307252
6	1	rs4926828	50218323	49355640	50591851	0.02443203
7	1	rs884712	66541305	66399895	66553495	0.03457592
8	1	rs1993709	72838529	72628347	73998230	0.00408593
9	1	rs75395158	75515902	75464373	75614734	0.01927512
10	1	rs6698829	76072167	76005994	76072167	0.03900091
11	1	rs11162028	87960702	87893268	87965485	0.01480336
12	1	rs475788	88748147	88692888	88864082	0.03962223
13	1	rs1198575	98562260	98468538	98562260	0.02773675
14	1	rs61786782	98851189	98827493	98858151	0.03818161
15	1	rs1730858	107619244	107537916	107627697	0.03766213
16	1	rs612231	112354653	112155156	112356946	0.02788355
17	1	rs146949757	177354939	177318047	177426976	0.02062249
18	1	rs7548821	181099494	181039628	181112055	0.02754567
19	1	rs7516334	197735331	197346937	197812879	0.01772669
20	1	rs1289395	227042462	227000011	227086937	0.0394977
21	1	rs7549029	230253406	230230169	230261441	0.02927123
22	1	rs116072968	232371512	232340219	232388034	0.0363974
23	1	rs118043249	241976242	241876491	241976242	0.01970448
24	1	rs6429441	244077683	244028907	244087842	0.02265029

25	2	rs13409834	22173664	22045717	22174521	0.02211527
26	2	rs504675	45154689	45130410	45174203	0.02095259
27	2	rs11682175	57987593	57943567	58065936	0.00140534
28	2	rs77593562	58951075	58951075	58951075	0.03798838
29	2	rs13025401	60056624	60055289	60098316	0.04492494
30	2	rs13026790	79398616	79398616	79437214	0.04830625
31	2	rs56875109	86725369	86570906	86858047	0.01806802
32	2	rs60597103	104452277	104421871	104452277	0.02807556
33	2	rs115802337	118817128	118817128	118839678	0.04902707
34	2	rs2126000	143418370	143331371	143559306	0.01254664
35	2	rs4233565	144266089	144178400	144272138	0.01281494
36	2	rs10185841	146261295	146122776	146309983	0.01835245
37	2	rs9287989	176717741	176717741	176727834	0.005983063
38	2	rs138105457	212648667	212648667	212650607	0.03337964
39	2	rs4145961	213074216	213043927	213123391	0.03699948
40	2	rs13023832	215219808	215081228	215219808	0.03510855
41	3	rs2600178	9402061	9378654	9438746	0.002166569
42	3	rs56023784	43439423	43254633	43591405	0.02532865
43	3	rs808719	44074503	43968666	44081022	0.0275928
44	3	rs11130182	49206767	48724811	49334768	0.000413604
45	3	rs4618171	65442578	65440410	65516835	0.01389262
46	3	rs1947150	71113096	71028756	71124867	0.01290989
47	3	rs7617513	114794493	114631548	114950578	0.03757211
48	3	rs3849471	117000128	116973279	117010780	0.01862463
49	4	rs12643329	11863873	11840610	11954403	0.03553374
50	4	rs9990752	15649079	15538922	15649079	0.007994571
51	4	rs62294142	38364637	38324024	38371812	0.01377616
52	4	rs17588767	41063263	40988780	41067204	0.02961262
53	4	rs6828811	42161066	42058329	42189248	0.03616277
54	4	rs1828580	43473352	43470533	43630731	0.04346779
55	4	rs17459996	46902967	46743805	47059807	0.04894061
56	4	rs34808931	58176747	58176747	58227751	0.01788579

57	4	rs7671093	101515453	101463177	101593148	0.04252085
58	4	rs45510091	123186393	123122856	123558330	0.03894747
59	4	rs79217540	183043303	183034755	183043303	0.04381603
60	5	rs2453371	3251092	3222173	3252073	0.04579011
61	5	rs6875412	30841976	30814006	30842041	0.02574048
62	5	rs62372027	45932074	45492398	46405067	0.03891504
63	5	rs62365524	50163883	49559501	50163883	0.03642809
64	5	rs16891019	62039960	62034795	62041461	0.03508926
65	5	rs972501	65010764	64768677	65010764	0.021865
66	5	rs6882046	87968864	87968864	87968864	0.00048933
67	5	rs1566032	92398402	92363455	92557969	0.04210545
68	5	rs1026281	93209839	93048832	93495631	0.02363673
69	5	rs30266	103972357	103783801	104082179	0.006095864
70	5	rs12153436	107063029	107063029	107684324	0.01431658
71	5	rs11952942	113407106	113395126	113464826	0.04661696
72	5	rs184937	129232553	129111396	129433560	0.04359849
73	5	rs4463213	139545748	139517197	139712550	0.01242791
74	5	rs6862346	153507705	153507705	153527056	0.02635323
75	5	rs80302414	166061033	166040547	166065998	0.03268673
76	6	rs6925748	50930041	50930041	50930041	0.01145515
77	6	rs7754169	91313558	91213633	91332937	0.03574147
78	6	rs240154	101065792	100813576	101339400	0.007339193
79	6	rs7768857	130757896	130631174	130771125	0.00851644
80	6	rs62420840	137459375	137316892	137459375	0.01243214
81	6	rs1334585	142976756	142868502	143011771	0.001900679
82	6	rs844584	147964120	147946018	147987135	0.003520535
83	7	rs62444881	2052318	1899447	2110850	0.000620857
84	7	rs9769350	2662211	2657996	2669863	0.01428017
85	7	rs7805419	12282451	12233848	12286050	0.001617273
86	7	rs73182840	70480827	70480827	70519481	0.04757732
87	7	rs1524274	109187479	109181005	109190835	0.02343985
88	7	rs35624276	114040103	114012911	114194615	0.003890194

89	7	rs3824009	126647487	126371011	126785804	0.01173759
90	7	rs55780035	128716440	128675068	128717467	0.04283292
91	7	rs1986692	133743393	133743393	133785003	0.03332791
92	7	rs9800952	140669703	140665521	140791820	0.004369348
93	8	rs55768139	10195477	10132326	10277978	0.000783959
94	8	rs11786924	12686438	12662159	12709650	0.01990126
95	8	rs2346375	31845024	31799091	31945918	0.01587397
96	8	rs11777872	64525347	64496159	64624581	0.02695876
97	8	rs7837935	65562019	65500967	65745896	0.02707532
98	8	rs6472981	77592275	77588548	77695732	0.0112674
99	8	rs1240115	90047848	89998538	90162526	0.03481763
100	8	rs2607106	92578051	92478693	92764816	0.009951287
101	8	rs4735221	94432323	94396331	94501471	0.03432928
102	8	rs59219135	114197277	113923381	114388945	0.01975319
103	8	rs2721938	116635611	116563879	116645056	0.0429535
104	8	rs12544831	118980246	118908192	119020964	0.02618654
105	8	rs876575	131210851	131057877	131361477	0.01137755
106	8	rs56779193	139586079	139586079	139587767	0.03815997
107	9	rs146869170	10347554	10347554	10347554	0.02881899
108	9	rs62533745	37078909	36999369	37406391	0.001854088
109	9	rs11137815	81354004	81331339	81354365	0.009510791
110	9	rs10512249	98256309	98191712	98314306	0.004056888
111	9	rs1927904	120514919	120502674	120730356	0.001763118
112	9	rs7040178	121318847	121240501	121318847	0.04177054
113	9	rs4837010	127923013	127780246	127987802	0.04843022
114	10	rs6602365	9675432	9651373	9963730	0.0227149
115	10	rs2399576	10887345	10840162	10910560	0.005101535
116	10	rs7899434	68527738	68482665	68575278	0.008577029
117	10	rs834920	69849245	69561765	69876042	0.04997423
118	10	rs1909696	77582203	77537562	77626488	0.01106276
119	10	rs10883434	101780614	101774727	101794992	0.03880074
120	11	rs7480631	11617902	11604714	11617902	0.02912446

121	11	rs10767733	28642320	28591168	28694440	0.000203257
122	11	rs60171845	31835468	31808280	32196933	0.01627901
123	11	rs72898691	45791373	45780039	45914046	0.02831814
124	11	rs78519907	48219866	48219866	49165670	0.03740509
125	11	rs499188	57434122	57385856	57681828	0.009352344
126	11	rs99780	61596633	61448384	61624181	0.01661351
127	11	rs17829309	88271811	88188047	88318926	0.00799369
128	11	rs477424	88802281	88765779	88943549	0.000100137
129	11	rs45519937	113194884	112827048	113427677	0.006541437
130	11	rs7937632	126991637	126899760	126998526	0.04894488
131	11	rs612823	133834104	133834104	133834104	0.005742033
132	11	rs11223816	134315499	134312341	134315499	0.03274182
133	12	rs138314529	32084683	32084683	32084683	0.0449975
134	12	rs12229507	99163194	99160676	99163991	0.01686019
135	12	rs7313797	109896165	109849410	110042348	0.009879327
136	12	rs12099598	117675697	117660827	117699505	0.01085684
137	12	rs6490177	118796085	118586689	118888131	0.001734519
138	12	rs4036462	125806709	125806709	125806709	0.02764769
139	13	rs9527083	53991125	53879428	54049489	0.01350838
140	13	rs1925060	55616633	55603644	55638510	0.006499998
141	13	rs9564291	66501198	66500825	66724659	0.001509108
142	13	rs10219949	67248036	66996044	67412175	0.03516542
143	13	rs4884833	69721057	69652435	70014965	0.01078064
144	13	rs7339081	79235501	79144362	79235544	0.03213517
145	13	rs6492656	93909387	93853929	93921542	0.02471614
146	13	rs7337667	97489457	97465425	97518070	0.00536093
147	13	rs9517301	99095088	99090837	99115041	0.01852844
148	14	rs9707362	41806217	41803291	41893906	0.04915825
149	14	rs8019784	47380711	47243109	47416511	0.01429155
150	14	rs2274793	57283968	57269825	57292728	0.01167409
151	14	rs6573413	62518146	62510531	62528686	0.04529657
152	14	rs7147721	75186010	75108290	75378185	0.000657405

153	14	rs11625467	94002924	93700821	94040544	0.03208062
154	14	rs12884892	98501431	98444702	98501877	0.02031267
155	14	rs12586414	101304239	101296662	101321758	0.02039754
156	14	rs942866	104014935	103987078	104093521	0.00169613
157	15	rs779	29856238	29848687	29858805	0.01602586
158	15	rs1604529	38218779	38173509	38263369	0.02570415
159	15	rs78061408	38815419	38722935	38816373	0.03321976
160	15	rs921764	47512281	47488977	47610697	0.02961923
161	15	rs1865216	70567255	70563984	70631100	0.04565444
162	15	rs72743363	74104811	74095709	74186934	0.03150448
163	15	rs4362360	86940622	86871047	87011615	0.01031235
164	15	rs6224	91423543	91412850	91429042	0.03057304
165	16	rs11077022	6340010	6266886	6345984	0.003425603
166	16	rs9929993	7664875	7657432	7673819	0.01507196
167	16	rs12934939	13802298	13763820	13805809	0.01621635
168	16	rs12926255	17851620	17851620	17851620	0.02952803
169	16	rs739710	18164698	18103546	18171046	0.02534304
170	16	rs2080501	49643566	49629325	49672520	0.04443573
171	16	rs34541615	56348261	56343642	56352905	0.04460022
172	16	rs7204052	60585280	60583974	60657996	0.01864341
173	16	rs4312307	71370974	71337760	71390323	0.03860913
174	16	rs13336147	72985338	72981230	72992221	0.04077348
175	17	rs4792133	11225355	11210835	11234195	0.04474547
176	17	rs35979784	19892453	19797869	20341005	0.01137959
177	17	rs56084168	79084574	79045773	79097769	0.03107188
178	18	rs8097544	1839564	1811604	1914051	0.04286005
179	18	rs1893947	25383952	25364193	25449930	0.04994831
180	18	rs2949540	27849827	27824014	27908727	0.03259742
181	18	rs10460051	31413679	31303557	31415929	0.04160687
182	18	rs12606772	35266566	35125161	35278126	0.008405423
183	18	rs12958173	42141977	41999291	42207147	0.01830332
184	18	rs16939951	44254122	44242450	44274374	0.02775647

185	18	rs7230285	50723080	50620087	50907127	0.01663107
186	18	rs12963463	53099093	51973130	53536547	1.70E-06
187	18	rs6567291	60255151	60223017	60657911	0.0249957
188	18	rs8083476	71596834	71596834	71620986	0.01561991
189	18	rs516890	77576767	77576767	77576767	0.04531154
190	19	rs58434384	19786099	19699398	19793545	0.04358916
191	19	rs192435	51804020	51801624	51824541	0.03999267
192	20	rs6040555	1186321	1182173	1186321	0.01680705
193	20	rs34478401	18696782	18484567	18724990	0.02905557
194	20	rs2425753	44705624	44684526	44728661	0.02367278
195	20	rs910187	45841052	45766642	45848043	0.02813169
196	22	rs1974652	20134147	20057078	20135961	0.00158168
197	22	rs926914	41418154	41215672	41713111	0.001471243
198	22	rs7410394	46455794	46448476	46463431	0.0283623

Abbreviations: CHR, Chromosome; BP, genomic position in human genome assembly; conjFDR, conjunctional false discovery rate

Supplementary Table 21. eQTL mapping for distinct genomic loci from the conjFDR results between irritability and each psychiatric disorder

a) Irritability and schizophrenia

IndSigSNPs	ensg	symbol	chr	start	end	strand	type	entrezID	HUGO	pLI	ncRVIS	eqtlMapSNPs	eqtlMapminP	eqtlMapminQ	eqtlMapts
rs7546034 9	ENSG00000090273	*NUDC*	1	27226729	27273353	1	protein_coding	10726	NUDC	0.822775133	0.218790394	5	2.76E-07	1.70E-08	GTEx/v8/Brain_Cortex:GTEx/v8/Brain_Nucleus_accumbens_basal_ganglia
rs7546034 9	ENSG00000142765	*SYTL1*	1	27668513	27680421	1	protein_coding	84958	SYTL1	1.08E-07	0.055031121	7	2.94E-07	1.48E-08	GTEx/v8/Brain_Cerebellar_Hemisphere:GTEx/v8/Brain_Cerebellum
rs7546034 9	ENSG00000174950	*CD164L2*	1	27705666	27709870	-1	protein_coding	388611	CD164L2	0.033744866	-0.467583072	3	1.60E-05	0.000165709	GTEx/v8/Brain_Hippocampus
rs7546034 9	ENSG00000126709	*IFI6*	1	27992572	27998729	-1	protein_coding	2537	IFI6	0.230931225	0.591640949	3	2.83E-05	0.00407134	GTEx/v8/Brain_Cerebellum:GTEx/v8/Brain_Hippocampus
rs11581846	ENSG00000116819	*TFAP2E*	1	36038971	36060929	1	protein_coding	339488	TFAP2E	0.000972259	-0.148652974	16	4.71E-05	0.0216732	GTEx/v8/Brain_Cerebellum
rs4411173	ENSG00000225206	*MIR137HG*	1	98453556	98515419	-1	lincRNA	400765	MIR137HG	NA	NA	1	5.56E-05	5.79E-06	GTEx/v8/Brain_Caudate_basal_ganglia
rs2636324	ENSG00000253326	*RP11-261C10.7*	1	243218163	243219696	-1	pseudogene	NA	NA	NA	NA	2	2.65E-05	4.88E-05	GTEx/v8/Brain_Putamen_basal_ganglia
rs2636324	ENSG00000054282	*SDCCAG8*	1	243419320	243663394	1	protein_coding	10806	SDCCAG8	1.33E-07	-0.422227426	27	2.25E-11	1.50E-08	GTEx/v8/Brain_Cerebellum
rs11680719	ENSG00000272663	*RP11-191L17.1*	2	4867182	4867736	-1	lincRNA	NA	NA	NA	NA	25	2.58E-08	1.15E-15	GTEx/v8/Brain_Caudate_basal_ganglia:GTEx/v8/Brain_Cortex:GTEx/v8/Brain_Putamen_basal_ganglia
rs11680719	ENSG00000162869	*PPP1R21*	2	4867737	4874252 5	1	protein_coding	129285	PPP1R21	0.0004021	0.571659952	34	5.65E-09	4.28E-14	GTEx/v8/Brain_Caudate_basal_ganglia:GTEx/v8/Brain_Frontal_Cortex_BA9:GTEx/v8/Brain_Hippocampus:GTEx/v8/Brain_Hypothalamus:GTEx/v8/Brain_Nucleus_accumbens_basal_ganglia:GTEx/v8/Brain_Putamen_basal_ganglia:GTEx/v8/Brain_Spinal_cord_cervical_c-1:GTEx/v8/Brain_Substantia_nigra
rs11680719	ENSG00000243244	*STON1*	2	4875652 2	4882602 5	1	protein_coding	11037	STON1	4.45E-08	1.251076578	34	2.38E-07	1.37E-09	GTEx/v8/Brain_Anterior_cingulate_cortex_BA24:GTEx/v8/Brain_Cortex:GTEx/v8/Brain_Putamen_basal_ganglia
rs11680719	ENSG00000068781	*STON1-GTF2A1L*	2	4875706 4	4900365 4	1	protein_coding	286749	STON1-GTF2A1L	1.65E-14	0.572863521	27	2.68E-07	4.03E-15	GTEx/v8/Brain_Cerebellum
rs11680719	ENSG00000242441	*GTF2A1L*	2	4884493 7	4896028 7	1	protein_coding	11036	GTF2A1L	5.65E-07	0.420928839	30	1.67E-09	4.48E-33	GTEx/v8/Brain_Cerebellum:GTEx/v8/Brain_Frontal_Cortex_BA9:GTEx/v8/Brain_Spinal_cord_cervical_c-1:GTEx/v8/Brain_Substantia_nigra
rs2717032	ENSG00000028116	*VRK2*	2	58134786	58387055	1	protein_coding	7444	VRK2	1.22E-08	-0.188348208	17	1.97E-07	0.00089661	GTEx/v8/Brain_Substantia_nigra
rs2717032	ENSG00000115392	*FANCL*	2	58386378	58468507	-1	protein_coding	55120	FANCL	2.10E-08	NA	19	2.64E-05	0.0327786	GTEx/v8/Brain_Cerebellum
rs2867910	ENSG00000186522	*SEPTIN10*	2	110300559	110371783	-1	protein_coding	151011	SEPTIN10	0.002883286	NA	40	6.34E-09	9.13E-08	GTEx/v8/Brain_Hippocampus:GTEx/v8/Brain_Putamen_basal_ganglia
rs67735550	ENSG00000115524	*SF3B1*	2	198254508	198299815	-1	protein_coding	23451	SF3B1	0.999999973	-0.619089113	50	1.83E-05	3.02E-05	GTEx/v8/Brain_Cerebellum
rs9882532	ENSG00000224728	*AC090945.1*	3	15919554	15921078	1	pseudogene	NA	NA	NA	NA	1	9.61E-06	0.000396696	GTEx/v8/Brain_Cerebellar_Hemisphere
rs4688436	ENSG00000241158	*ADAMTS9-AS1*	3	64547014	64573878	1	antisense	101929335	ADAMTS9-AS1	NA	NA	4	1.02E-06	0.00796819	GTEx/v8/Brain_Anterior_cingulate_cortex_BA24
rs836927	ENSG00000273125	*RP11-115H18.1*	3	107149777	107182759	1	lincRNA	101929607	LINC01990	NA	NA	6	2.14E-11	2.40E-13	GTEx/v8/Brain_Caudate_basal_ganglia:GTEx/v8/Brain_Nucleus_accumbens_basal_ganglia:GTEx/v8/Brain_Putamen_basal_ganglia
rs59413629	ENSG00000155016	*CYP2U1*	4	108852525	108874613	1	protein_coding	113612	CYP2U1	0.081783015	-0.309381857	20	3.65E-10	1.77E-06	GTEx/v8/Brain_Cerebellar_Hemisphere:GTEx/v8/Brain_Cerebellum
rs1852599	ENSG00000250422	*RP11-484L7.1*	5	45558021	45560088	1	pseudogene	NA	NA	NA	NA	89	5.05E-05	0.0275529	GTEx/v8/Brain_Cerebellar_Hemisphere
rs62382095	ENSG00000164574	*GALNT10*	5	153570290	153800544	1	protein_coding	55568	GALNT10	0.00044926	1.469543542	2	5.65E-05	9.34E-08	GTEx/v8/Brain_Cerebellum

rs11154565	ENSG00000164483	*SAMD3*	6	130465460	130686570	-1	protein_coding	154075	SAMD3	1.10E-06	0.088198284	242	8.80E-07	0.00198934	GTEx/v8/Brain_Cortex
rs6244294	ENSG00000176349	*AC110781.3*	7	1878222	1889567	1	protein_coding	NA	NA	NA	NA	103	2.94E-09	2.61E-06	GTEx/v8/Brain_Caudate_basal_ganglia:GTEx/v8/Brain_Hypothalamus:GTEx/v8/Brain_Nucleus_accumbens_basal_ganglia
rs6244294	ENSG00000122687	*FTSJ2*	7	2273866	2281840	-1	protein_coding	29960	FTSJ2	1.97E-05	0.096595649	114	1.66E-07	4.01E-12	GTEx/v8/Brain_Caudate_basal_ganglia:GTEx/v8/Brain_Cerebellar_Hemisphere:GTEx/v8/Brain_Cortex
rs2854153 0	ENSG00000105866	*SP4*	7	21467652	21554440	1	protein_coding	6671	SP4	0.951288043	0.671148604	12	5.63E-06	0.0233599	GTEx/v8/Brain_Nucleus_accumbens_basal_ganglia
rs28541530	ENSG00000105877	*DNAH11*	7	21582833	21941457	1	protein_coding	8701	DNAH11	NA	0.456635771	21	3.56E-07	2.36E-08	GTEx/v8/Brain_Caudate_basal_ganglia:GTEx/v8/Brain_Cortex:GTEx/v8/Brain_Frontal_Cortex_BA9:GTEx/v8/Brain_Putamen_basal_ganglia
rs1669022	ENSG00000228031	*AC078842.3*	7	137029676	137039229	-1	lincRNA	NA	NA	NA	NA	2	3.19E-06	0.0128012	GTEx/v8/Brain_Caudate_basal_ganglia:GTEx/v8/Brain_Frontal_Cortex_BA9
rs9800952	ENSG00000090263	*MRPS33*	7	140705854	140715028	-1	protein_coding	51650	MRPS33	0.003395718	0.268392925	51	2.30E-09	1.80E-05	GTEx/v8/Brain_Caudate_basal_ganglia:GTEx/v8/Brain_Cortex:GTEx/v8/Brain_Putamen_basal_ganglia
rs1986972	ENSG00000175806	*MSRA*	8	9911778	10286401	1	protein_coding	4482	MSRA	1.70E-06	0.601529643	2	2.12E-05	2.59E-07	GTEx/v8/Brain_Spinal_cord_cervical_c-1
rs1986972	ENSG00000261451	*RP11-981G7.1*	8	10291182	10295822	1	sense_overlapping	NA	NA	NA	NA	2	2.27E-05	1.99E-06	GTEx/v8/Brain_Hippocampus
rs1986972	ENSG00000253641	*RP11-981G7.2*	8	10332075	10339484	1	lincRNA	101929191	LINCR-0001	NA	NA	38	1.54E-09	3.41E-05	GTEx/v8/Brain_Caudate_basal_ganglia:GTEx/v8/Brain_Hippocampus
rs1986972	ENSG00000253678	*RP11-981G7.3*	8	10335001	10336885	-1	lincRNA	NA	NA	NA	NA	7	2.33E-06	1.19E-35	GTEx/v8/Brain_Cerebellum:GTEx/v8/Brain_Cortex:GTEx/v8/Brain_Substantia_nigra
rs1986972	ENSG00000253649	*PRSS51*	8	10340388	10405095	-1	antisense	346702	PRSS51	NA	NA	8	1.15E-05	0.00803096	GTEx/v8/Brain_Cortex
rs1986972	ENSG00000272505	*RP11-981G7.6*	8	10344317	10347176	1	lincRNA	NA	NA	NA	NA	47	4.76E-10	4.62E-06	GTEx/v8/Brain_Anterior_cingulate_cortex_BA24:GTEx/v8/Brain_Cortex:GTEx/v8/Brain_Frontal_Cortex_BA9:GTEx/v8/Brain_Hippocampus:GTEx/v8/Brain_Putamen_basal_ganglia:GTEx/v8/Brain_Spinal_cord_cervical_c-1
rs1986972	ENSG00000184647	*PRSS55*	8	10383056	10411676	1	protein_coding	203074	PRSS55	1.23E-08	0.255257421	44	3.98E-07	2.50E-21	GTEx/v8/Brain_Cerebellar_Hemisphere
rs1986972	ENSG00000269918	*AF131215.9*	8	10962201	10964214	-1	sense_intronic	NA	NA	NA	NA	40	1.57E-07	5.23E-05	GTEx/v8/Brain_Cerebellar_Hemisphere:GTEx/v8/Brain_Cerebellum
rs1986972	ENSG00000255310	*AF131215.2*	8	10965298	10967236	-1	sense_intronic	NA	NA	NA	NA	50	1.90E-10	5.98E-09	GTEx/v8/Brain_Cerebellar_Hemisphere:GTEx/v8/Brain_Cerebellum
rs1986972	ENSG00000154316	*TDH*	8	11197146	11225961	1	pseudogene	157739	TDH	NA	NA	38	3.31E-06	3.83E-09	GTEx/v8/Brain_Cerebellum:GTEx/v8/Brain_Cortex:GTEx/v8/Brain_Nucleus_accumbens_basal_ganglia
rs1986972	ENSG00000255020	*AF131216.5*	8	11203257	11205011	-1	antisense	100129129	LOC100129129	NA	NA	49	2.68E-07	4.05E-17	GTEx/v8/Brain_Cerebellar_Hemisphere:GTEx/v8/Brain_Cerebellum:GTEx/v8/Brain_Cortex:GTEx/v8/Brain_Frontal_Cortex_BA9:GTEx/v8/Brain_Hippocampus:GTEx/v8/Brain_Putamen_basal_ganglia
rs1986972	ENSG00000184608	*C8orf12*	8	11225911	11296167	1	protein_coding	83656	C8orf12	0.31126891	NA	2	3.17E-05	5.17E-05	GTEx/v8/Brain_Cerebellum
rs7002619	ENSG00000038945	*MSR1*	8	15965387	16424999	-1	protein_coding	4481	MSR1	8.21E-16	1.819933539	46	7.05E-07	0.00568105	GTEx/v8/Brain_Caudate_basal_ganglia:GTEx/v8/Brain_Hypothalamus:GTEx/v8/Brain_Nucleus_accumbens_basal_ganglia
rs1043083	ENSG00000198791	*CNOT7*	8	17086737	17104387	-1	protein_coding	29883	CNOT7	0.986495484	-0.616746759	4	1.51E-05	3.25E-22	GTEx/v8/Brain_Cerebellar_Hemisphere:GTEx/v8/Brain_Cerebellum:GTEx/v8/Brain_Frontal_Cortex_BA9:GTEx/v8/Brain_Hypothalamus
rs191124455	ENSG00000085788	*DDHD2*	8	38082736	38133076	1	protein_coding	23259	DDHD2	3.10E-08	-1.458035377	1	5.46E-06	1.12E-08	GTEx/v8/Brain_Cerebellar_Hemisphere:GTEx/v8/Brain_Hippocampus
rs191124455	ENSG00000147535	*PPAPDC1B*	8	38120648	38126761	-1	protein_coding	84513	PPAPDC1B	3.07E-07	-0.702559497	1	3.57E-08	2.19E-10	GTEx/v8/Brain_Cerebellar_Hemisphere:GTEx/v8/Brain_Cerebellum
rs191124455	ENSG00000239218	*RPS20P22*	8	38291865	38293182	-1	pseudogene	100271087	RPS20P22	NA	NA	1	1.11E-08	3.18E-05	GTEx/v8/Brain_Cerebellar_Hemisphere:GTEx/v8/Brain_Cerebellum
rs1700143	ENSG00000156103	*MMP16*	8	89044237	89340254	-1	protein_coding	4325	MMP16	0.916974084	-3.432432894	2	0.000180996	0.0179096	GTEx/v8/Brain_Cerebellum
rs1700143	ENSG00000253553	*RP11-586K2.1*	8	89339065	89749363	1	antisense	NA	NA	NA	NA	6	8.19E-05	0.000376539	GTEx/v8/Brain_Cortex
rs7272365	ENSG00000153317	*ASAP1*	8	131064353	131455906	-1	protein_coding	50807	ASAP1	0.999983091	0.592920078	53	1.01E-08	1.01E-16	GTEx/v8/Brain_Cerebellar_Hemisphere:GTEx/v8/Brain_Cerebellum:GTEx/v8/Brain_Cortex:GTEx/v8/Brain_Nucleus_accumbens_basal_ganglia:GTEx/v8/Brain_Putamen_basal_ganglia
rs7835528	ENSG00000176956	*LY6H*	8	144239331	144242128	-1	protein_coding	4062	LY6H	0.460605569	NA	27	2.41E-13	5.24E-09	GTEx/v8/Brain_Cerebellar_Hemisphere:GTEx/v8/Brain_Cerebellum:GTEx/v8/Brain_Cortex:GTEx/v8/Brain_Frontal_Cortex_BA9
rs7835528	ENSG00000253716	*RP13-582O9.5*	8	144362331	144363860	-1	antisense	100507316	MINCR	NA	NA	4	6.62E-06	5.76E-24	GTEx/v8/Brain_Cortex:GTEx/v8/Brain_Nucleus_accumbens_basal_ganglia:GTEx/v8/Brain_Putamen_basal_ganglia

rs7835528	ENSG00000178209	*PLEC*	8	144989321	145050902	-1	protein_coding	5339	PLEC	0.024936271	-0.44044438	14	3.83E-06	0.0165148	GTEx/v8/Brain_Hippocampus
rs13296413	ENSG00000147905	*ZCCHC7*	9	37120536	37358146	1	protein_coding	84186	ZCCHC7	3.37E-09	0.160585505	11	8.60E-06	0.0328071	GTEx/v8/Brain_Hippocampus
rs12416687	ENSG00000269609	*RP11-18I14.10*	10	104209574	104220863	1	processed_transcript	100505761	RPARP-AS1	NA	NA	182	2.55E-08	6.18E-17	GTEx/v8/Brain_Caudate_basal_ganglia:GTEx/v8/Brain_Cerebellum:GTEx/v8/Brain_Frontal_Cortex_BA9:GTEx/v8/Brain_Hypothalamus:GTEx/v8/Brain_Nucleus_accumbens_basal_ganglia:GTEx/v8/Brain_Putamen_basal_ganglia
rs12416687	ENSG00000138111	*TMEM180*	10	104221149	104236802	1	protein_coding	79847	TMEM180	0.000178664	2.882749821	163	7.24E-08	4.09E-26	GTEx/v8/Brain_Caudate_basal_ganglia:GTEx/v8/Brain_Cerebellar_Hemisphere:GTEx/v8/Brain_Cerebellum:GTEx/v8/Brain_Nucleus_accumbens_basal_ganglia:GTEx/v8/Brain_Putamen_basal_ganglia
rs12416687	ENSG00000138175	*ARL3*	10	104433488	104474164	-1	protein_coding	403	ARL3	0.716101007	-0.600025748	6	2.47E-07	3.19E-05	GTEx/v8/Brain_Cerebellum
rs12416687	ENSG00000156398	*SFXN2*	10	104474295	104503249	1	protein_coding	118980	SFXN2	0.001084574	-0.837011237	18	2.16E-06	3.55E-05	GTEx/v8/Brain_Cerebellum:GTEx/v8/Brain_Cortex
rs12416687	ENSG00000148795	*CYP17A1*	10	104590288	104597290	-1	protein_coding	1586	CYP17A1	0.035030608	0.086681977	18	5.84E-06	0.0022099	GTEx/v8/Brain_Cortex:GTEx/v8/Brain_Frontal_Cortex_BA9
rs12416687	ENSG00000166275	*C10orf32*	10	104613980	104624718	1	protein_coding	119032	C10orf32	0.031637861	0.490358211	182	7.87E-33	5.95E-26	GTEx/v8/Brain_Amygdala:GTEx/v8/Brain_Anterior_cingulate_cortex_BA24:GTEx/v8/Brain_Caudate_basal_ganglia:GTEx/v8/Brain_Cerebellar_Hemisphere:GTEx/v8/Brain_Cerebellum:GTEx/v8/Brain_Cortex:GTEx/v8/Brain_Frontal_Cortex_BA9:GTEx/v8/Brain_Hippocampus:GTEx/v8/Brain_Hypothalamus:GTEx/v8/Brain_Nucleus_accumbens_basal_ganglia:GTEx/v8/Brain_Putamen_basal_ganglia:GTEx/v8/Brain_Spinal_cord_cervical_c-1:GTEx/v8/Brain_Substantia_nigra
rs12416687	ENSG00000214435	*AS3MT*	10	104629273	104661656	1	protein_coding	57412	AS3MT	5.09E-07	0.726881178	182	3.44E-42	6.43E-34	GTEx/v8/Brain_Amygdala:GTEx/v8/Brain_Anterior_cingulate_cortex_BA24:GTEx/v8/Brain_Caudate_basal_ganglia:GTEx/v8/Brain_Cerebellar_Hemisphere:GTEx/v8/Brain_Cerebellum:GTEx/v8/Brain_Cortex:GTEx/v8/Brain_Frontal_Cortex_BA9:GTEx/v8/Brain_Hippocampus:GTEx/v8/Brain_Hypothalamus:GTEx/v8/Brain_Nucleus_accumbens_basal_ganglia:GTEx/v8/Brain_Putamen_basal_ganglia:GTEx/v8/Brain_Spinal_cord_cervical_c-1:GTEx/v8/Brain_Substantia_nigra
rs12416687	ENSG00000235266	*RP11-753C18.8*	10	104647631	104647945	-1	pseudogene	NA	NA	NA	NA	182	1.47E-15	1.79E-11	GTEx/v8/Brain_Cerebellar_Hemisphere:GTEx/v8/Brain_Cerebellum
rs12416687	ENSG00000272912	*RP11-724N1.1*	10	104674342	104675161	1	lincRNA	NA	NA	NA	NA	182	1.95E-21	3.13E-16	GTEx/v8/Brain_Cerebellar_Hemisphere:GTEx/v8/Brain_Cerebellum
rs12416687	ENSG00000076685	*NT5C2*	10	104845940	104953056	-1	protein_coding	22978	NT5C2	0.052197863	0.04832858	173	3.99E-08	2.15E-07	GTEx/v8/Brain_Cerebellar_Hemisphere:GTEx/v8/Brain_Cerebellum:GTEx/v8/Brain_Cortex
rs12416687	ENSG00000148843	*PDCD11*	10	105156405	105206049	1	protein_coding	22984	PDCD11	0.468448698	-0.805378431	7	1.06E-05	5.99E-06	GTEx/v8/Brain_Cerebellum
rs499188	ENSG00000149150	*SLC43A1*	11	57252007	57283259	-1	protein_coding	8501	SLC43A1	7.85E-05	-0.136928307	5	4.46E-05	2.48E-08	GTEx/v8/Brain_Cerebellum
rs499188	ENSG00000134809	*TIMM10*	11	57295936	57298276	-1	protein_coding	26519	TIMM10	0.009494762	0.000100078	15	1.68E-05	7.82E-13	GTEx/v8/Brain_Caudate_basal_ganglia
rs499188	ENSG00000156603	*MED19*	11	57471186	57479693	-1	protein_coding	219541	MED19	0.683790051	-0.151700149	83	5.44E-06	5.15E-07	GTEx/v8/Brain_Cerebellar_Hemisphere:GTEx/v8/Brain_Cerebellum
rs499188	ENSG00000242689	*CNTF*	11	58390146	58393198	1	protein_coding	1270	CNTF	0.000349252	-0.109964842	48	1.60E-05	0.0192174	GTEx/v8/Brain_Cortex
rs61687445	ENSG00000149295	*DRD2*	11	113280318	113346413	-1	protein_coding	1813	DRD2	0.733263889	NA	7	1.95E-06	0.00562986	GTEx/v8/Brain_Cerebellar_Hemisphere
rs61687445	ENSG00000166682	*TMPRSS5*	11	113558272	113577095	-1	protein_coding	80975	TMPRSS5	2.58E-08	0.18379421	2	2.13E-05	7.17E-27	GTEx/v8/Brain_Caudate_basal_ganglia:GTEx/v8/Brain_Putamen_basal_ganglia
rs612823	ENSG00000080854	*IGSF9B*	11	133778459	133826880	-1	protein_coding	22997	IGSF9B	0.999873394	NA	1	6.52E-09	5.01E-09	GTEx/v8/Brain_Cerebellum
rs2159100	ENSG00000151067	*CACNA1C*	12	2079952	2802108	1	protein_coding	775	CACNA1C	0.999998433	-0.35401516	91	1.16E-13	1.53E-12	GTEx/v8/Brain_Cerebellar_Hemisphere:GTEx/v8/Brain_Cerebellum
rs35393419	ENSG00000139428	*MMAB*	12	109991542	110011679	-1	protein_coding	326625	MMAB	0.00387716	2.918192677	6	7.13E-06	3.85E-05	GTEx/v8/Brain_Caudate_basal_ganglia:GTEx/v8/Brain_Frontal_Cortex_BA9
rs7332097	ENSG00000255874	*LINC00346*	13	111521578	111522162	-1	protein_coding	283487	LINC00346	NA	NA	26	3.38E-07	0.000489464	GTEx/v8/Brain_Substantia_nigra
rs6560933	ENSG00000185989	*RASA3*	13	114747194	114898086	-1	protein_coding	22821	RASA3	0.000433959	0.061217245	47	9.46E-10	2.59E-08	GTEx/v8/Brain_Cerebellar_Hemisphere:GTEx/v8/Brain_Cerebellum:GTEx/v8/Brain_Cortex
rs6560933	ENSG00000169062	*UPF3A*	13	115047059	115071283	1	protein_coding	65110	UPF3A	0.000813153	0.189120204	3	3.36E-05	0.00632297	GTEx/v8/Brain_Spinal_cord_cervical_c-1
rs8019784	ENSG00000139915	*MDGA2*	14	47308826	48144157	-1	protein_coding	161357	MDGA2	0.993216871	0.397758699	47	9.51E-06	0.0198975	GTEx/v8/Brain_Caudate_basal_ganglia:GTEx/v8/Brain_Cerebellar_Hemisphere:GTEx/v8/Brain_Cortex:GTEx/v8/Brain_Nucleus_accumbens_basal_ganglia
rs703235	ENSG00000247982	*LINC00926*	15	57592563	57599959	1	lincRNA	283663	LINC00926	NA	NA	8	3.64E-06	7.21E-24	GTEx/v8/Brain_Caudate_basal_ganglia:GTEx/v8/Brain_Cerebellar_Hemisphere

rs8060690	ENSG00000102981	*PARD6A*	16	67694849	67696681	1	protein_coding	50855	PARD6A	0.854734701	NA	1	0.000228794	0.0497999	GTEx/v8/Brain_Cerebellum
rs8060690	ENSG00000124074	*ENKD1*	16	67696848	67701168	-1	protein_coding	84080	ENKD1	2.15E-05	NA	29	6.01E-05	9.56E-08	GTEx/v8/Brain_Cerebellum
rs8060690	ENSG00000141098	*GFOD2*	16	67708434	67753324	-1	protein_coding	81577	GFOD2	0.060537434	-0.955753302	31	2.55E-05	0.0031938	GTEx/v8/Brain_Amygdala
rs8060690	ENSG00000102904	*TSNAXIP1*	16	67840668	67866051	1	protein_coding	55815	TSNAXIP1	9.27E-18	-0.329502309	14	1.15E-06	3.53E-08	GTEx/v8/Brain_Cerebellar_Hemisphere:GTEx/v8/Brain_Frontal_Cortex_BA9
rs8060690	ENSG00000132600	*PRMT7*	16	68344877	68392466	1	protein_coding	54496	PRMT7	8.31E-09	-0.873098608	91	4.95E-10	6.54E-16	GTEx/v8/Brain_Amygdala:GTEx/v8/Brain_Caudate_basal_ganglia:GTEx/v8/Brain_Cortex:GTEx/v8/Brain_Frontal_Cortex_BA9:GTEx/v8/Brain_Nucleus_accumbens_basal_ganglia:GTEx/v8/Brain_Putamen_basal_ganglia:GTEx/v8/Brain_Substantia_nigra
rs17702834	ENSG00000141076	*CIRH1A*	16	69165194	69265033	1	protein_coding	84916	CIRH1A	0.094027705	NA	1	2.19E-11	1.84E-21	GTEx/v8/Brain_Caudate_basal_ganglia:GTEx/v8/Brain_Cerebellar_Hemisphere:GTEx/v8/Brain_Cerebellum:GTEx/v8/Brain_Cortex:GTEx/v8/Brain_Frontal_Cortex_BA9:GTEx/v8/Brain_Hippocampus:GTEx/v8/Brain_Nucleus_accumbens_basal_ganglia:GTEx/v8/Brain_Putamen_basal_ganglia
rs17702834	ENSG00000132612	*VPS4A*	16	69345259	69358949	1	protein_coding	27183	VPS4A	NA	NA	1	6.94E-05	2.83E-08	GTEx/v8/Brain_Frontal_Cortex_BA9
rs499188	ENSG00000265566	*RN7SL605P*	17	2657126	2657430	1	misc_RNA	106481087	RN7SL605P	NA	NA	1	0.000105864	5.83E-09	GTEx/v8/Brain_Frontal_Cortex_BA9
rs62063686	ENSG00000136448	*NMT1*	17	43128978	43186384	1	protein_coding	4836	NMT1	0.998500521	0.032447683	1801	9.33E-06	2.39E-07	GTEx/v8/Brain_Cerebellar_Hemisphere:GTEx/v8/Brain_Cerebellum:GTEx/v8/Brain_Spinal_cord_cervical_c-1
rs62063686	ENSG00000267121	*CTD-2020K17.1*	17	43268298	43299589	-1	antisense	339192	LOC339192	NA	NA	2668	6.12E-11	4.50E-07	GTEx/v8/Brain_Cerebellar_Hemisphere:GTEx/v8/Brain_Cerebellum
rs62063686	ENSG00000184922	*FMNL1*	17	43298811	43324687	1	protein_coding	752	FMNL1	0.999687036	NA	2668	1.02E-24	7.89E-19	GTEx/v8/Brain_Cerebellar_Hemisphere:GTEx/v8/Brain_Cerebellum
rs62063686	ENSG00000233175	*CTD-2020K17.3*	17	43315395	43319101	-1	antisense	107985040	LOC107985040	NA	NA	21	9.72E-05	2.69E-16	GTEx/v8/Brain_Cerebellum
rs62063686	ENSG00000006062	*MAP3K14*	17	43340488	43394414	-1	processed_transcript	9020	MAP3K14	NA	NA	2	0.000176915	0.00353334	GTEx/v8/Brain_Cerebellum
rs62063686	ENSG00000159314	*ARHGAP27*	17	43471275	43511787	-1	protein_coding	201176	ARHGAP27	0.001255844	-0.761627793	2806	4.59E-19	7.23E-14	GTEx/v8/Brain_Caudate_basal_ganglia:GTEx/v8/Brain_Cerebellar_Hemisphere:GTEx/v8/Brain_Cerebellum:GTEx/v8/Brain_Cortex:GTEx/v8/Brain_Nucleus_accumbens_basal_ganglia:GTEx/v8/Brain_Putamen_basal_ganglia
rs62063686	ENSG00000225190	*PLEKHM1*	17	43513266	43568115	-1	protein_coding	9842	PLEKHM1	0.84054321	-0.663068032	2806	1.98E-42	8.80E-35	GTEx/v8/Brain_Amygdala:GTEx/v8/Brain_Anterior_cingulate_cortex_BA24:GTEx/v8/Brain_Caudate_basal_ganglia:GTEx/v8/Brain_Cerebellar_Hemisphere:GTEx/v8/Brain_Cerebellum:GTEx/v8/Brain_Cortex:GTEx/v8/Brain_Frontal_Cortex_BA9:GTEx/v8/Brain_Hypothalamus:GTEx/v8/Brain_Nucleus_accumbens_basal_ganglia:GTEx/v8/Brain_Putamen_basal_ganglia
rs62063686	ENSG00000236234	*AC091132.1*	17	43530210	43541431	1	antisense	NA	NA	NA	NA	2806	2.17E-12	2.29E-08	GTEx/v8/Brain_Cerebellar_Hemisphere:GTEx/v8/Brain_Cerebellum
rs62063686	ENSG00000214425	*LRRC37A4P*	17	43578685	43627701	-1	pseudogene	55073	LRRC37A4P	NA	NA	2808	1.81E-58	2.69E-48	GTEx/v8/Brain_Amygdala:GTEx/v8/Brain_Anterior_cingulate_cortex_BA24:GTEx/v8/Brain_Caudate_basal_ganglia:GTEx/v8/Brain_Cerebellar_Hemisphere:GTEx/v8/Brain_Cerebellum:GTEx/v8/Brain_Cortex:GTEx/v8/Brain_Frontal_Cortex_BA9:GTEx/v8/Brain_Hippocampus:GTEx/v8/Brain_Hypothalamus:GTEx/v8/Brain_Nucleus_accumbens_basal_ganglia:GTEx/v8/Brain_Putamen_basal_ganglia:GTEx/v8/Brain_Spinal_cord_cervical_c-1:GTEx/v8/Brain_Substantia_nigra
rs62063686	ENSG00000266918	*RP11-798G7.8*	17	43608943	43611204	1	lincRNA	NA	NA	NA	NA	2806	2.14E-16	6.68E-12	GTEx/v8/Brain_Amygdala:GTEx/v8/Brain_Caudate_basal_ganglia:GTEx/v8/Brain_Cerebellar_Hemisphere:GTEx/v8/Brain_Cerebellum:GTEx/v8/Brain_Cortex
rs62063686	ENSG00000267198	*RP11-798G7.6*	17	43623170	43640596	1	lincRNA	NA	NA	NA	NA	2606	5.49E-06	1.37E-08	GTEx/v8/Brain_Cerebellum
rs62063686	ENSG00000264070	*DND1P1*	17	43663237	43664295	1	pseudogene	644157	DND1P1	NA	NA	2807	7.80E-38	1.42E-31	GTEx/v8/Brain_Amygdala:GTEx/v8/Brain_Anterior_cingulate_cortex_BA24:GTEx/v8/Brain_Caudate_basal_ganglia:GTEx/v8/Brain_Cerebellar_Hemisphere:GTEx/v8/Brain_Cerebellum:GTEx/v8/Brain_Cortex:GTEx/v8/Brain_Frontal_Cortex_BA9:GTEx/v8/Brain_Hippocampus:GTEx/v8/Brain_Hypothalamus:GTEx/v8/Brain_Nucleus_accumbens_basal_ganglia:GTEx/v8/Brain_Putamen_basal_ganglia:GTEx/v8/Brain_Spinal_cord_cervical_c-1:GTEx/v8/Brain_Substantia_nigra
rs62063686	ENSG00000263503	*RP11-707O23.5*	17	43678235	43679706	-1	pseudogene	NA	NA	NA	NA	2807	1.33E-41	1.26E-35	GTEx/v8/Brain_Amygdala:GTEx/v8/Brain_Anterior_cingulate_cortex_BA24:GTEx/v8/Brain_Caudate_basal_ganglia:GTEx/v8/Brain_Cerebellar_Hemisphere:GTEx/v8/Brain_Cerebellum:GTEx/v8/Brain_Cortex:GTEx/v8/Brain_Frontal_Cortex_BA9:GTEx/v8/Brain_Hippocampus:GTEx/v8/Brain_Hypothalamus:GTEx/v8/Brain_Nucleus_accumbens_basal_ganglia:GTEx/v8/Brain_Putamen_basal_ganglia:GTEx/v8/Brain_Spinal_cord_cervical_c-1:GTEx/v8/Brain_Substantia_nigra
rs62063686	ENSG00000204650	*CRHR1-IT1*	17	43697694	43725582	1	pseudogene	147081	CRHR1-IT1	NA	NA	2808	1.06E-38	1.09E-30	GTEx/v8/Brain_Amygdala:GTEx/v8/Brain_Anterior_cingulate_cortex_BA24:GTEx/v8/Brain_Caudate_basal_ganglia:GTEx/v8/Brain_Cerebellar_Hemisphere:GTEx/v8/Brain_Cerebellum:GTEx/v8/Brain_Cortex:GTEx/v8/Brain_Frontal_Cortex_BA9:GTEx/v8/Brain_Hippocampus:GTEx/v8/Brain_Hypothalamus:GTEx/v8/Brain_Nucleus_accumbens_basal_ganglia:GTEx/v8/Brain_Putamen_basal_ganglia:GTEx/v8/Brain_Spinal_cord_cervical_c-1:GTEx/v8/Brain_Substantia_nigra
rs62063686	ENSG00000120088	*CRHR1*	17	43699267	43913194	1	protein_coding	1394	CRHR1	0.792134286	2.038085631	2819	4.29E-06	0.00117547	GTEx/v8/Brain_Caudate_basal_ganglia:GTEx/v8/Brain_Cortex:GTEx/v8/Brain_Hippocampus:GTEx/v8/Brain_Nucleus_accumbens_basal_ganglia:GTEx/v8/Brain_Putamen_basal_ganglia
rs62063686	ENSG00000264589	*MAPT-AS1*	17	43921017	43972966	-1	antisense	100128977	MAPT-AS1	NA	NA	2879	1.07E-21	3.37E-16	GTEx/v8/Brain_Caudate_basal_ganglia:GTEx/v8/Brain_Cerebellar_Hemisphere:GTEx/v8/Brain_Cerebellum:GTEx/v8/Brain_Hippocampus:GTEx/v8/Brain_Hypothalamus:GTEx/v8/Brain_Nucleus_accumbens_basal_ganglia:GTEx/v8/Brain_Putamen_basal_ganglia:GTEx/v8/Brain_Spinal_cord_cervical_c-1:GTEx/v8/Brain_Substantia_nigra
rs62063686	ENSG00000185294	*SPPL2C*	17	43922256	43924438	1	protein_coding	162540	SPPL2C	2.30E-05	0.187394842	2879	6.65E-25	5.44E-19	GTEx/v8/Brain_Cerebellar_Hemisphere:GTEx/v8/Brain_Cerebellum:GTEx/v8/Brain_Cortex:GTEx/v8/Brain_Frontal_Cortex_BA9
rs62063686	ENSG00000186868	*MAPT*	17	43971748	44105700	1	protein_coding	4137	MAPT	4.68E-05	2.01024275	2853	1.10E-07	7.42E-05	GTEx/v8/Brain_Caudate_basal_ganglia:GTEx/v8/Brain_Cerebellar_Hemisphere:GTEx/v8/Brain_Cerebellum
rs62063686	ENSG00000120071	*KANSL1*	17	44107282	44302733	-1	protein_coding	284058	KANSL1	0.999733506	0.058945245	2867	5.76E-07	1.09E-09	GTEx/v8/Brain_Caudate_basal_ganglia:GTEx/v8/Brain_Cerebellar_Hemisphere:GTEx/v8/Brain_Cerebellum:GTEx/v8/Brain_Frontal_Cortex_BA9
rs62063686	ENSG00000214401	*KANSL1-AS1*	17	44270942	44274089	1	antisense	644246	KANSL1-AS1	NA	NA	2879	4.51E-52	1.22E-43	GTEx/v8/Brain_Amygdala:GTEx/v8/Brain_Anterior_cingulate_cortex_BA24:GTEx/v8/Brain_Caudate_basal_ganglia:GTEx/v8/Brain_Cerebellar_Hemisphere:GTEx/v8/Brain_Cerebellum:GTEx/v8/Brain_Cortex:GTEx/v8/Brain_Frontal_Cortex_BA9:GTEx/v8/Brain_Hippocampus:GTEx/v8/Brain_Hypothalamus:GTEx/v8/Brain_Nucleus_accumbens_basal_ganglia:GTEx/v8/Brain_Putamen_basal_ganglia:GTEx/v8/Brain_Spinal_cord_cervical_c-1:GTEx/v8/Brain_Substantia_nigra

rs62063686	ENSG00000262500	*RP11-259G18.2*	17	44320972	44322410	1	pseudogene	NA	NA	NA	NA	2879	2.61E-40	5.94E-34	GTEx/v8/Brain_Amygdala:GTEx/v8/Brain_Anterior_cingulate_cortex_BA24:GTEx/v8/Brain_Caudate_basal_ganglia:GTEx/v8/Brain_Cerebellar_Hemisphere:GTEx/v8/Brain_Cerebellum:GTEx/v8/Brain_Cortex:GTEx/v8/Brain_Frontal_Cortex_BA9:GTEx/v8/Brain_Hippocampus:GTEx/v8/Brain_Hypothalamus:GTEx/v8/Brain_Nucleus_accumbens_basal_ganglia:GTEx/v8/Brain_Putamen_basal_ganglia:GTEx/v8/Brain_Spinal_cord_cervical_c-1:GTEx/v8/Brain_Substantia_nigra
rs62063686	ENSG00000262539	*RP11-259G18.3*	17	44336917	44337972	-1	pseudogene	NA	NA	NA	NA	2880	3.51E-45	1.60E-37	GTEx/v8/Brain_Amygdala:GTEx/v8/Brain_Anterior_cingulate_cortex_BA24:GTEx/v8/Brain_Caudate_basal_ganglia:GTEx/v8/Brain_Cerebellar_Hemisphere:GTEx/v8/Brain_Cerebellum:GTEx/v8/Brain_Cortex:GTEx/v8/Brain_Frontal_Cortex_BA9:GTEx/v8/Brain_Hippocampus:GTEx/v8/Brain_Hypothalamus:GTEx/v8/Brain_Nucleus_accumbens_basal_ganglia:GTEx/v8/Brain_Putamen_basal_ganglia:GTEx/v8/Brain_Spinal_cord_cervical_c-1:GTEx/v8/Brain_Substantia_nigra
rs62063686	ENSG00000261575	*RP11-259G18.1*	17	44344403	44346060	1	pseudogene	NA	NA	NA	NA	2880	7.46E-51	1.13E-41	GTEx/v8/Brain_Amygdala:GTEx/v8/Brain_Anterior_cingulate_cortex_BA24:GTEx/v8/Brain_Caudate_basal_ganglia:GTEx/v8/Brain_Cerebellar_Hemisphere:GTEx/v8/Brain_Cerebellum:GTEx/v8/Brain_Cortex:GTEx/v8/Brain_Frontal_Cortex_BA9:GTEx/v8/Brain_Hippocampus:GTEx/v8/Brain_Hypothalamus:GTEx/v8/Brain_Nucleus_accumbens_basal_ganglia:GTEx/v8/Brain_Putamen_basal_ganglia:GTEx/v8/Brain_Spinal_cord_cervical_c-1:GTEx/v8/Brain_Substantia_nigra
rs62063686	ENSG00000228696	*ARL17B*	17	44352150	44439130	-1	protein_coding	100506084	ARL17B	NA	-0.428243465	32	4.31E-08	2.36E-17	GTEx/v8/Brain_Cerebellar_Hemisphere:GTEx/v8/Brain_Cerebellum:GTEx/v8/Brain_Hypothalamus
rs62063686	ENSG00000176681	*LRRC37A*	17	44370099	44415160	1	protein_coding	9884	LRRC37A	0.890487356	NA	2879	1.21E-30	4.87E-42	GTEx/v8/Brain_Amygdala:GTEx/v8/Brain_Anterior_cingulate_cortex_BA24:GTEx/v8/Brain_Caudate_basal_ganglia:GTEx/v8/Brain_Cerebellar_Hemisphere:GTEx/v8/Brain_Cerebellum:GTEx/v8/Brain_Cortex:GTEx/v8/Brain_Frontal_Cortex_BA9:GTEx/v8/Brain_Hippocampus:GTEx/v8/Brain_Hypothalamus:GTEx/v8/Brain_Nucleus_accumbens_basal_ganglia:GTEx/v8/Brain_Putamen_basal_ganglia:GTEx/v8/Brain_Spinal_cord_cervical_c-1:GTEx/v8/Brain_Substantia_nigra
rs62063686	ENSG00000238083	*LRRC37A2*	17	44588877	44633016	1	protein_coding	474170	LRRC37A2	NA	NA	2733	2.68E-52	7.91E-44	GTEx/v8/Brain_Amygdala:GTEx/v8/Brain_Anterior_cingulate_cortex_BA24:GTEx/v8/Brain_Caudate_basal_ganglia:GTEx/v8/Brain_Cerebellar_Hemisphere:GTEx/v8/Brain_Cerebellum:GTEx/v8/Brain_Cortex:GTEx/v8/Brain_Frontal_Cortex_BA9:GTEx/v8/Brain_Hippocampus:GTEx/v8/Brain_Hypothalamus:GTEx/v8/Brain_Nucleus_accumbens_basal_ganglia:GTEx/v8/Brain_Putamen_basal_ganglia:GTEx/v8/Brain_Spinal_cord_cervical_c-1:GTEx/v8/Brain_Substantia_nigra
rs62063686	ENSG00000185829	*ARL17A*	17	44594068	44657088	-1	protein_coding	51326	ARL17A	NA	-2.38258287	2733	4.39E-38	7.42E-31	GTEx/v8/Brain_Amygdala:GTEx/v8/Brain_Anterior_cingulate_cortex_BA24:GTEx/v8/Brain_Caudate_basal_ganglia:GTEx/v8/Brain_Cerebellar_Hemisphere:GTEx/v8/Brain_Cerebellum:GTEx/v8/Brain_Cortex:GTEx/v8/Brain_Frontal_Cortex_BA9:GTEx/v8/Brain_Hippocampus:GTEx/v8/Brain_Hypothalamus:GTEx/v8/Brain_Nucleus_accumbens_basal_ganglia:GTEx/v8/Brain_Putamen_basal_ganglia
rs62063686	ENSG00000232300	*FAM215B*	17	44636196	44640161	-1	sense_intronic	644297	FAM215B	NA	NA	2733	6.29E-24	2.68E-18	GTEx/v8/Brain_Cerebellar_Hemisphere:GTEx/v8/Brain_Cerebellum
rs62063686	ENSG00000073969	*NSF*	17	44668035	44834830	1	protein_coding	4905	NSF	0.71368213	-0.566246769	2416	1.64E-06	2.14E-10	GTEx/v8/Brain_Cerebellum
rs62063686	ENSG00000108379	*WNT3*	17	44839872	44910520	-1	protein_coding	7473	WNT3	0.946108101	-0.191110949	28	1.53E-05	1.18E-51	GTEx/v8/Brain_Cerebellar_Hemisphere:GTEx/v8/Brain_Cerebellum:GTEx/v8/Brain_Cortex
rs62063686	ENSG00000263142	*LRRC37A17P*	17	45055847	45131935	1	pseudogene	644397	LRRC37A17P	NA	NA	925	2.50E-05	0.0172168	GTEx/v8/Brain_Cerebellum
rs62063686	ENSG00000178852	*EFCAB13*	17	45400656	45518678	1	protein_coding	124989	EFCAB13	8.22E-19	-0.190444586	3	4.77E-05	1.78E-07	GTEx/v8/Brain_Anterior_cingulate_cortex_BA24
rs3469859	ENSG00000124299	*PEPD*	19	33877856	34012700	-1	protein_coding	5184	PEPD	0.014507135	0.225176003	8	9.74E-06	1.86E-11	GTEx/v8/Brain_Cerebellar_Hemisphere
rs3469859	ENSG00000124302	*CHST8*	19	34112861	34264414	1	protein_coding	64377	CHST8	0.004222016	-1.382729988	31	5.44E-06	0.0157132	GTEx/v8/Brain_Cortex
rs6072230	ENSG00000124181	*PLCG1*	20	39765600	39811629	1	protein_coding	5335	PLCG1	0.763352352	NA	21	2.91E-05	0.027998	GTEx/v8/Brain_Cerebellar_Hemisphere
rs6072230	ENSG00000183798	*EMILIN3*	20	39988606	39995467	-1	protein_coding	90187	EMILIN3	0.001398097	0.56095672	85	3.01E-07	6.10E-10	GTEx/v8/Brain_Anterior_cingulate_cortex_BA24:GTEx/v8/Brain_Hypothalamus:GTEx/v8/Brain_Nucleus_accumbens_basal_ganglia:GTEx/v8/Brain_Putamen_basal_ganglia
rs8137258	ENSG00000236540	*AC006547.13*	22	20050503	20058066	-1	antisense	NA	NA	NA	NA	22	3.87E-06	0.000102016	GTEx/v8/Brain_Caudate_basal_ganglia:GTEx/v8/Brain_Cortex
rs8137258	ENSG00000099899	*TRMT2A*	22	20099389	20104915	-1	protein_coding	27037	TRMT2A	1.31E-05	NA	5	2.59E-05	0.0244377	GTEx/v8/Brain_Cerebellar_Hemisphere
rs8137258	ENSG00000099904	*ZDHHC8*	22	20116979	20135530	1	protein_coding	29801	ZDHHC8	0.988991096	NA	7	9.68E-06	0.000390337	GTEx/v8/Brain_Caudate_basal_ganglia:GTEx/v8/Brain_Cerebellum
rs20551	ENSG00000128285	*MCHR1*	22	41074754	41078818	1	protein_coding	2847	MCHR1	0.001148393	-0.081006493	6	1.89E-05	8.11E-10	GTEx/v8/Brain_Cerebellum
rs20551	ENSG00000100372	*SLC25A17*	22	41165634	41215403	-1	protein_coding	10478	SLC25A17	0.024184844	-0.779478694	41	6.86E-06	2.58E-05	GTEx/v8/Brain_Cerebellum:GTEx/v8/Brain_Nucleus_accumbens_basal_ganglia:GTEx/v8/Brain_Putamen_basal_ganglia
rs20551	ENSG00000213857	*RP11-12M9.4*	22	41470184	41471243	-1	pseudogene	NA	NA	NA	NA	6	8.01E-05	0.0176512	GTEx/v8/Brain_Cerebellum
rs20551	ENSG00000100393	*EP300*	22	41487790	41576081	1	protein_coding	2033	EP300	1	NA	113	2.44E-06	0.00184947	GTEx/v8/Brain_Cerebellum:GTEx/v8/Brain_Hypothalamus
rs20551	ENSG00000100395	*L3MBTL2*	22	41601209	41627275	1	protein_coding	83746	L3MBTL2	0.013028043	-0.618259224	121	1.80E-08	5.18E-05	GTEx/v8/Brain_Anterior_cingulate_cortex_BA24:GTEx/v8/Brain_Frontal_Cortex_BA9
rs20551	ENSG00000100401	*RANGAP1*	22	41641615	41682255	-1	protein_coding	5905	RANGAP1	0.13271872	-0.376521602	76	7.87E-07	1.39E-06	GTEx/v8/Brain_Cerebellar_Hemisphere:GTEx/v8/Brain_Cerebellum
rs20551	ENSG00000100403	*ZC3H7B*	22	41697526	41756151	1	protein_coding	23264	ZC3H7B	0.990761551	-0.628157842	125	4.49E-08	0.00010307	GTEx/v8/Brain_Cerebellum

rs20551	ENSG00000100412	*ACO2*	22	41865129	41924993	1	protein_coding	50	ACO2	0.454377775	-0.617411984	21	2.91E-05	0.00290367	GTEx/v8/Brain_Cerebellum
rs20551	ENSG00000100413	*POLR3H*	22	41921808	41940610	-1	protein_coding	171568	POLR3H	0.002007106	-1.305852274	120	2.19E-08	8.24E-14	GTEx/v8/Brain_Cerebellum:GTEx/v8/Brain_Cortex:GTEx/v8/Brain_Frontal_Cortex_BA9:GTEx/v8/Brain_Putamen_basal_ganglia
rs20551	ENSG00000172346	*CSDC2*	22	41956767	41973745	1	protein_coding	27254	CSDC2	0.079803516	0.31935561	121	2.14E-09	6.71E-18	GTEx/v8/Brain_Cortex:GTEx/v8/Brain_Frontal_Cortex_BA9
rs20551	ENSG00000167077	*MEI1*	22	42095503	42195460	1	protein_coding	150365	MEI1	1.22E-08	0.500155337	115	1.71E-06	1.60E-05	GTEx/v8/Brain_Anterior_cingulate_cortex_BA24:GTEx/v8/Brain_Frontal_Cortex_BA9
rs20551	ENSG00000159958	*TNFRSF13C*	22	42321045	42322822	-1	protein_coding	115650	TNFRSF13C	0.641054688	-0.063981155	31	1.87E-05	1.22E-11	GTEx/v8/Brain_Cerebellar_Hemisphere
rs20551	ENSG00000205704	*LINC00634*	22	42348169	42354937	1	pseudogene	339674	LINC00634	NA	NA	9	6.19E-05	0.000116414	GTEx/v8/Brain_Frontal_Cortex_BA9
rs20551	ENSG00000183066	*WBP2NL*	22	42394729	42454460	1	protein_coding	164684	WBP2NL	2.07E-09	0.730692723	1	7.68E-05	1.04E-06	GTEx/v8/Brain_Frontal_Cortex_BA9
rs20551	ENSG00000198951	*NAGA*	22	42454358	42466846	-1	protein_coding	4668	NAGA	2.02E-06	0.183467033	6	8.98E-07	7.04E-29	GTEx/v8/Brain_Cerebellar_Hemisphere:GTEx/v8/Brain_Cerebellum
rs20551	ENSG00000100197	*CYP2D6*	22	42522501	42526908	-1	protein_coding	1565	CYP2D6	9.23E-10	0.153953666	12	2.97E-05	4.44E-35	GTEx/v8/Brain_Cerebellum:GTEx/v8/Brain_Nucleus_accumbens_basal_ganglia

b) Irritability and bipolar I disorder

IndSigSNPs	ensg	symbol	chr	start	end	strand	type	entrezID	HUGO	pLI	ncRVIS	eqtlMapSNPs	eqtlMapminP	eqtlMapminQ	eqtlMapts
rs9425311	ENSG00000135845	*PIGC*	1	172339329	172413230	-1	protein_coding	5279	PIGC	0.000110183	-0.675062169	27	2.50E-12	2.52E-08	GTEx/v8/Brain_Amygdala:GTEx/v8/Brain_Cerebellar_Hemisphere:GTEx/v8/Brain_Cerebellum:GTEx/v8/Brain_Cortex:GTEx/v8/Brain_Frontal_Cortex_BA9:GTEx/v8/Brain_Hypothalamus:GTEx/v8/Brain_Nucleus_accumbens_basal_ganglia:GTEx/v8/Brain_Putamen_basal_ganglia
rs7314722 8	ENSG00000175161	*CADM2*	3	85008132	86123579	1	protein_coding	253559	CADM2	0.98466891	0.117806454	3	5.28E-05	2.39E-08	GTEx/v8/Brain_Spinal_cord_cervical_c-1
rs73147228	ENSG00000239519	*CADM2-AS1*	3	86041333	86077157	-1	antisense	10087403 8	CADM2-AS1	NA	NA	49	8.34E-07	1.12E-14	GTEx/v8/Brain_Cerebellar_Hemisphere:GTEx/v8/Brain_Cerebellum:GTEx/v8/Brain_Cortex:GTEx/v8/Brain_Nucleus_accumbens_basal_ganglia
rs62261974	ENSG00000241218	*RP11-446H18.1*	3	107046677	107048044	-1	pseudogene	NA	NA	NA	NA	69	3.58E-06	0.0214886	GTEx/v8/Brain_Amygdala
rs56305291	ENSG00000176349	*AC110781.3*	7	1878222	1889567	1	protein_coding	NA	NA	NA	NA	73	3.26E-07	2.61E-06	GTEx/v8/Brain_Nucleus_accumbens_basal_ganglia
rs56305291	ENSG00000122687	*FTSJ2*	7	2273866	2281840	-1	protein_coding	29960	FTSJ2	1.97E-05	0.096595649	93	1.66E-07	4.01E-12	GTEx/v8/Brain_Caudate_basal_ganglia:GTEx/v8/Brain_Cerebellar_Hemisphere:GTEx/v8/Brain_Cortex
rs28541530	ENSG00000105866	*SP4*	7	21467652	21554440	1	protein_coding	6671	SP4	0.951288043	0.671148604	12	5.63E-06	0.0233599	GTEx/v8/Brain_Nucleus_accumbens_basal_ganglia
rs28541530	ENSG00000105877	*DNAH11*	7	21582833	21941457	1	protein_coding	8701	DNAH11	NA	0.456635771	21	3.56E-07	2.36E-08	GTEx/v8/Brain_Caudate_basal_ganglia:GTEx/v8/Brain_Cortex:GTEx/v8/Brain_Frontal_Cortex_BA9:GTEx/v8/Brain_Putamen_basal_ganglia
rs6464217	ENSG00000157764	*BRAF*	7	140419127	140624564	-1	protein_coding	673	BRAF	0.999978196	NA	1	2.80E-05	0.0377877	GTEx/v8/Brain_Cerebellum
rs6464217	ENSG00000090263	*MRPS33*	7	140705854	140715028	-1	protein_coding	51650	MRPS33	0.003395718	0.268392925	51	2.30E-09	1.80E-05	GTEx/v8/Brain_Caudate_basal_ganglia:GTEx/v8/Brain_Cortex:GTEx/v8/Brain_Putamen_basal_ganglia
rs57737477	ENSG00000261451	*RP11-981G7.1*	8	10291182	10295822	1	sense_overlapping	NA	NA	NA	NA	19	3.79E-06	3.66E-17	GTEx/v8/Brain_Cerebellar_Hemisphere
rs1243184	ENSG00000204682	*CASC10*	10	21781587	21786191	-1	protein_coding	399726	CASC10	0.020054862	-1.904040172	104	9.62E-07	0.00239579	GTEx/v8/Brain_Cerebellum
rs1243184	ENSG00000078403	*MLLT10*	10	21823094	22032559	1	protein_coding	8028	MLLT10	0.999583699	-0.518307189	1	4.58E-05	0.0340703	GTEx/v8/Brain_Anterior_cingulate_cortex_BA24
rs12244388	ENSG00000269609	*RP11-18I14.10*	10	104209574	104220863	1	processed_transcript	100505761	RPARP-AS1	NA	NA	163	3.39E-07	6.18E-17	GTEx/v8/Brain_Caudate_basal_ganglia:GTEx/v8/Brain_Cerebellar_Hemisphere:GTEx/v8/Brain_Cerebellum:GTEx/v8/Brain_Frontal_Cortex_BA9:GTEx/v8/Brain_Nucleus_accumbens_basal_ganglia
rs12244388	ENSG00000138111	*TMEM180*	10	104221149	104236802	1	protein_coding	79847	TMEM180	0.000178664	2.882749821	145	9.97E-09	1.06E-22	GTEx/v8/Brain_Caudate_basal_ganglia:GTEx/v8/Brain_Cerebellar_Hemisphere:GTEx/v8/Brain_Nucleus_accumbens_basal_ganglia:GTEx/v8/Brain_Putamen_basal_ganglia:GTEx/v8/Brain_Spinal_cord_cervical_c-1:GTEx/v8/Brain_Substantia_nigra

rs12244388	ENSG00000107882	*SUFU*	10	104263744	104393292	1	protein_coding	51684	SUFU	0.999161485	0.124860143	17	2.99E-05	0.029185	GTEx/v8/Brain_Putamen_basal_ganglia
rs12244388	ENSG00000138175	*ARL3*	10	104433488	104474164	-1	protein_coding	403	ARL3	0.716101007	-0.600025748	155	2.47E-07	3.19E-05	GTEx/v8/Brain_Cerebellum
rs12244388	ENSG00000156398	*SFXN2*	10	104474295	104503249	1	protein_coding	118980	SFXN2	0.001084574	-0.837011237	12	5.51E-05	4.58E-05	GTEx/v8/Brain_Cerebellum
rs12244388	ENSG00000148795	*CYP17A1*	10	104590288	104597290	-1	protein_coding	1586	CYP17A1	0.035030608	0.086681977	3	6.02E-05	0.0022099	GTEx/v8/Brain_Cortex
rs12244388	ENSG00000166275	*C10orf32*	10	104613980	104624718	1	protein_coding	119032	C10orf32	0.031637861	0.490358211	163	2.00E-25	5.95E-26	GTEx/v8/Brain_Anterior_cingulate_cortex_BA24:GTEx/v8/Brain_Caudate_basal_ganglia:GTEx/v8/Brain_Cerebellar_Hemisphere:GTEx/v8/Brain_Cerebellum:GTEx/v8/Brain_Cortex:GTEx/v8/Brain_Frontal_Cortex_BA9:GTEx/v8/Brain_Hippocampus:GTEx/v8/Brain_Hypothalamus:GTEx/v8/Brain_Nucleus_accumbens_basal_ganglia:GTEx/v8/Brain_Putamen_basal_ganglia:GTEx/v8/Brain_Spinal_cord_cervical_c-1:GTEx/v8/Brain_Substantia_nigra
rs12244388	ENSG00000214435	*AS3MT*	10	104629273	104661656	1	protein_coding	57412	AS3MT	5.09E-07	0.726881178	163	3.44E-42	6.43E-34	GTEx/v8/Brain_Amygdala:GTEx/v8/Brain_Anterior_cingulate_cortex_BA24:GTEx/v8/Brain_Caudate_basal_ganglia:GTEx/v8/Brain_Cerebellar_Hemisphere:GTEx/v8/Brain_Cerebellum:GTEx/v8/Brain_Cortex:GTEx/v8/Brain_Frontal_Cortex_BA9:GTEx/v8/Brain_Hippocampus:GTEx/v8/Brain_Hypothalamus:GTEx/v8/Brain_Nucleus_accumbens_basal_ganglia:GTEx/v8/Brain_Putamen_basal_ganglia:GTEx/v8/Brain_Spinal_cord_cervical_c-1:GTEx/v8/Brain_Substantia_nigra
rs12244388	ENSG00000235266	*RP11-753C18.8*	10	104647631	104647945	-1	pseudogene	NA	NA	NA	NA	163	2.27E-16	1.79E-11	GTEx/v8/Brain_Cerebellar_Hemisphere:GTEx/v8/Brain_Cerebellum
rs12244388	ENSG00000272912	*RP11-724N1.1*	10	104674342	104675161	1	lincRNA	NA	NA	NA	NA	163	1.95E-21	3.13E-16	GTEx/v8/Brain_Cerebellar_Hemisphere:GTEx/v8/Brain_Cerebellum:GTEx/v8/Brain_Hypothalamus:GTEx/v8/Brain_Spinal_cord_cervical_c-1
rs12244388	ENSG00000076685	*NT5C2*	10	104845940	104953056	-1	protein_coding	22978	NT5C2	0.052197863	0.04832858	8	1.01E-07	2.15E-07	GTEx/v8/Brain_Cerebellar_Hemisphere:GTEx/v8/Brain_Cerebellum
rs12244388	ENSG00000148843	*PDCD11*	10	105156405	105206049	1	protein_coding	22984	PDCD11	0.468448698	-0.805378431	7	1.06E-05	5.99E-06	GTEx/v8/Brain_Cerebellum
rs174529	ENSG00000134825	*TMEM258*	11	61535973	61560274	-1	protein_coding	746	TMEM258	0.000864646	0.122739186	40	1.36E-06	0.00433323	GTEx/v8/Brain_Frontal_Cortex_BA9:GTEx/v8/Brain_Putamen_basal_ganglia
rs174529	ENSG00000149485	*FADS1*	11	61567099	61596790	-1	protein_coding	3992	FADS1	0.383801127	-0.763752724	53	7.63E-33	3.14E-26	GTEx/v8/Brain_Anterior_cingulate_cortex_BA24:GTEx/v8/Brain_Caudate_basal_ganglia:GTEx/v8/Brain_Cerebellar_Hemisphere:GTEx/v8/Brain_Cerebellum:GTEx/v8/Brain_Cortex:GTEx/v8/Brain_Frontal_Cortex_BA9:GTEx/v8/Brain_Hippocampus:GTEx/v8/Brain_Hypothalamus:GTEx/v8/Brain_Putamen_basal_ganglia:GTEx/v8/Brain_Spinal_cord_cervical_c-1
rs174529	ENSG00000221968	*FADS3*	11	61640991	61659523	-1	protein_coding	3995	FADS3	0.048872245	NA	50	1.79E-11	1.73E-07	GTEx/v8/Brain_Cerebellar_Hemisphere:GTEx/v8/Brain_Cerebellum
rs2159100	ENSG00000151067	*CACNA1C*	12	2079952	2802108	1	protein_coding	775	CACNA1C	0.999998433	-0.35401516	91	1.16E-13	1.53E-12	GTEx/v8/Brain_Cerebellar_Hemisphere:GTEx/v8/Brain_Cerebellum
rs10783826	ENSG00000123329	*ARHGAP9*	12	57866038	57882597	-1	protein_coding	64333	ARHGAP9	2.89E-09	-0.374064649	24	1.59E-10	9.83E-07	GTEx/v8/Brain_Cerebellar_Hemisphere:GTEx/v8/Brain_Cerebellum
rs10783826	ENSG00000166986	*MARS*	12	57869228	57911352	1	protein_coding	4141	MARS	2.74E-12	-0.447643065	13	4.40E-07	0.000942149	GTEx/v8/Brain_Cerebellum
rs10783826	ENSG00000166987	*MBD6*	12	57914493	57923931	1	protein_coding	114785	MBD6	0.9636575	NA	7	5.44E-05	0.000957867	GTEx/v8/Brain_Caudate_basal_ganglia
rs10783826	ENSG00000123427	*METTL21B*	12	58165275	58176324	1	protein_coding	25895	METTL21B	0.00509294	-0.795239894	7	4.03E-05	7.14E-32	GTEx/v8/Brain_Cerebellar_Hemisphere
rs9527083	ENSG00000225510	*PCDH8P1*	13	53774116	53776547	1	pseudogene	100133285	PCDH8P1	NA	NA	12	5.33E-06	0.00408458	GTEx/v8/Brain_Anterior_cingulate_cortex_BA24
rs17702834	ENSG00000141076	*CIRH1A*	16	69165194	69265033	1	protein_coding	84916	CIRH1A	0.094027705	NA	1	2.19E-11	1.84E-21	GTEx/v8/Brain_Caudate_basal_ganglia:GTEx/v8/Brain_Cerebellar_Hemisphere:GTEx/v8/Brain_Cerebellum:GTEx/v8/Brain_Cortex:GTEx/v8/Brain_Frontal_Cortex_BA9:GTEx/v8/Brain_Hippocampus:GTEx/v8/Brain_Nucleus_accumbens_basal_ganglia:GTEx/v8/Brain_Putamen_basal_ganglia
rs17702834	ENSG00000132612	*VPS4A*	16	69345259	69358949	1	protein_coding	27183	VPS4A	NA	NA	1	6.94E-05	2.83E-08	GTEx/v8/Brain_Frontal_Cortex_BA9
rs9606265	ENSG00000099899	*TRMT2A*	22	20099389	20104915	-1	protein_coding	27037	TRMT2A	1.31E-05	NA	5	2.59E-05	0.0244377	GTEx/v8/Brain_Cerebellar_Hemisphere
rs9606265	ENSG00000099904	*ZDHHC8*	22	20116979	20135530	1	protein_coding	29801	ZDHHC8	0.988991096	NA	5	1.56E-05	0.0313939	GTEx/v8/Brain_Caudate_basal_ganglia
rs80533	ENSG00000100372	*SLC25A17*	22	41165634	41215403	-1	protein_coding	10478	SLC25A17	0.024184844	-0.779478694	2	4.95E-09	2.58E-05	GTEx/v8/Brain_Nucleus_accumbens_basal_ganglia:GTEx/v8/Brain_Putamen_basal_ganglia
rs80533	ENSG00000196236	*XPNPEP3*	22	41253081	41363838	1	protein_coding	63929	XPNPEP3	8.88E-06	-0.745193699	1	0.000192103	7.04E-05	GTEx/v8/Brain_Cerebellar_Hemisphere
rs80533	ENSG00000213857	*RP11-12M9.4*	22	41470184	41471243	-1	pseudogene	NA	NA	NA	NA	1	0.000194011	0.0176512	GTEx/v8/Brain_Cerebellum
rs80533	ENSG00000100395	*L3MBTL2*	22	41601209	41627275	1	protein_coding	83746	L3MBTL2	0.013028043	-0.618	2	6.53E-06	5.18E-05	GTEx/v8/Brain_Anterior_cingulate_cortex_BA24:GTEx/v8/Brain_Frontal_Cortex_BA9

											259224				
rs80533	ENSG00000100401	*RANGAP1*	22	41641615	41682255	-1	protein_coding	5905	RANGAP1	0.13271872	-0.376521602	1	0.000137727	1.39E-06	GTEx/v8/Brain_Cerebellum
rs80533	ENSG00000100403	*ZC3H7B*	22	41697526	41756151	1	protein_coding	23264	ZC3H7B	0.990761551	-0.628157842	1	0.000114164	0.00010307	GTEx/v8/Brain_Cerebellum
rs80533	ENSG00000100413	*POLR3H*	22	41921808	41940610	-1	protein_coding	171568	POLR3H	0.002007106	-1.305852274	1	8.98E-06	8.24E-14	GTEx/v8/Brain_Cerebellum

c) Irritability and major depressive disorder

IndSigSNPs	ensg	symbol	chr	start	end	strand	type	entrezID	HUGO	pLI	ncRVIS	eqtlMapSNPs	eqtlMapminP	eqtlMapminQ	eqtlMapts
rs11211481	ENSG00000162366	*PDZK1IP1*	1	47649265	47656716	-1	protein_coding	10158	PDZK1IP1	0.000484065	-0.343318344	38	6.02E-06	2.89E-10	GTEx/v8/Brain_Cortex:GTEx/v8/Brain_Putamen_basal_ganglia
rs11211481	ENSG00000162367	*TAL1*	1	47681962	47697892	-1	protein_coding	6886	TAL1	0.824692215	-0.797257236	42	7.92E-06	0.0180879	GTEx/v8/Brain_Cortex
rs11211481	ENSG00000186790	*FOXE3*	1	47881744	47883723	1	protein_coding	2301	FOXE3	NA	-0.345111816	40	3.21E-06	1.28E-08	GTEx/v8/Brain_Cerebellum
rs1993709	ENSG00000172260	*NEGR1*	1	71861623	72748417	-1	protein_coding	257194	NEGR1	0.951349395	-1.713579543	14	4.26E-05	1.84E-05	GTEx/v8/Brain_Caudate_basal_ganglia:GTEx/v8/Brain_Putamen_basal_ganglia
rs1993709	ENSG00000227207	*RPL31P12*	1	72767155	72767512	1	pseudogene	100270887	RPL31P12	NA	NA	99	1.73E-17	1.23E-38	GTEx/v8/Brain_Cerebellar_Hemisphere:GTEx/v8/Brain_Cerebellum
rs6698829	ENSG00000137968	*SLC44A5*	1	75667816	76076801	-1	protein_coding	204962	SLC44A5	0.03422869	0.661940842	17	2.22E-05	4.82E-09	GTEx/v8/Brain_Cerebellar_Hemisphere:GTEx/v8/Brain_Cerebellum
rs1198575	ENSG00000225206	*MIR137HG*	1	98453556	98515419	-1	lincRNA	400765	MIR137HG	NA	NA	1	5.56E-05	5.79E-06	GTEx/v8/Brain_Caudate_basal_ganglia
rs1730858	ENSG00000198890	*PRMT6*	1	107599267	107601916	1	protein_coding	55170	PRMT6	0.024127621	-0.632333634	40	1.04E-14	4.59E-10	GTEx/v8/Brain_Anterior_cingulate_cortex_BA24:GTEx/v8/Brain_Caudate_basal_ganglia:GTEx/v8/Brain_Cerebellar_Hemisphere:GTEx/v8/Brain_Cerebellum:GTEx/v8/Brain_Cortex:GTEx/v8/Brain_Frontal_Cortex_BA9:GTEx/v8/Brain_Hippocampus:GTEx/v8/Brain_Hypothalamus:GTEx/v8/Brain_Putamen_basal_ganglia:GTEx/v8/Brain_Spinal_cord_cervical_c-1:GTEx/v8/Brain_Substantia_nigra
rs7548821	ENSG00000135823	*STX6*	1	180941861	180992047	-1	protein_coding	10228	STX6	0.019849117	-2.017748203	2	2.78E-05	0.00171914	GTEx/v8/Brain_Frontal_Cortex_BA9
rs7516334	ENSG00000134376	*CRB1*	1	197170592	197447585	1	protein_coding	23418	CRB1	3.36E-09	-0.36786871	23	8.03E-06	0.00600862	GTEx/v8/Brain_Cerebellum:GTEx/v8/Brain_Cortex:GTEx/v8/Brain_Nucleus_accumbens_basal_ganglia
rs7516334	ENSG00000213047	*DENND1B*	1	197473878	197744826	-1	protein_coding	163486	DENND1B	0.636578248	-0.145185446	4	7.19E-06	0.00908843	GTEx/v8/Brain_Cerebellum
rs1289395	ENSG00000143801	*PSEN2*	1	227057885	227083806	1	protein_coding	5664	PSEN2	0.027014182	0.479322645	41	3.19E-08	0.000259971	GTEx/v8/Brain_Caudate_basal_ganglia:GTEx/v8/Brain_Frontal_Cortex_BA9:GTEx/v8/Brain_Hypothalamus:GTEx/v8/Brain_Putamen_basal_ganglia
rs1289395	ENSG00000163050	*ADCK3*	1	227085237	227175246	1	protein_coding	56997	ADCK3	1.24E-05	0.831024079	42	9.30E-08	4.56E-09	GTEx/v8/Brain_Cerebellar_Hemisphere:GTEx/v8/Brain_Cerebellum:GTEx/v8/Brain_Cortex
rs7549029	ENSG00000143641	*GALNT2*	1	230193536	230417870	1	protein_coding	2590	GALNT2	0.881985613	4.223663186	53	4.65E-09	1.89E-14	GTEx/v8/Brain_Cerebellar_Hemisphere:GTEx/v8/Brain_Cerebellum
rs504675	ENSG00000236502	*SIX3-AS1*	2	45167293	45169012	-1	antisense	100506108	SIX3-AS1	NA	NA	8	4.68E-07	4.76E-10	GTEx/v8/Brain_Caudate_basal_ganglia:GTEx/v8/Brain_Nucleus_accumbens_basal_ganglia
rs504675	ENSG00000225156	*AC012354.6*	2	45181803	45195901	1	lincRNA	NA	NA	NA	NA	13	7.40E-08	9.59E-10	GTEx/v8/Brain_Caudate_basal_ganglia:GTEx/v8/Brain_Nucleus_accumbens_basal_ganglia:GTEx/v8/Brain_Putamen_basal_ganglia
rs56875109	ENSG00000273080	*RP11-301O19.1*	2	86422713	86423172	1	antisense	NA	NA	NA	NA	9	4.43E-05	2.00E-25	GTEx/v8/Brain_Caudate_basal_ganglia:GTEx/v8/Brain_Nucleus_accumbens_basal_ganglia
rs56875109	ENSG00000115561	*CHMP3*	2	86730554	86948245	-1	protein_coding	51652	CHMP3	0.036245866	-0.19401976	139	2.22E-12	5.21E-08	GTEx/v8/Brain_Caudate_basal_ganglia:GTEx/v8/Brain_Cortex:GTEx/v8/Brain_Frontal_Cortex_BA9:GTEx/v8/Brain_Nucleus_accumbens_basal_ganglia:GTEx/v8/Brain_Putamen_basal_ganglia
rs56875109	ENSG00000183281	*PLGLB1*	2	87229682	87248975	-1	protein_coding	5343	PLGLB1	NA	-0.640421533	128	5.26E-07	4.22E-22	GTEx/v8/Brain_Caudate_basal_ganglia:GTEx/v8/Brain_Cerebellum:GTEx/v8/Brain_Putamen_basal_ganglia

rs2600178	ENSG00000206573	*SETD5-AS1*	3	9391373	9440263	-1	antisense	440944	SETD5-AS1	NA	NA	15	3.96E-08	7.18E-05	GTEx/v8/Brain_Cerebellar_Hemisphere
rs2600178	ENSG00000134077	*THUMPD3*	3	9404526	9428475	1	protein_coding	25917	THUMPD3	4.00E-11	0.700963169	15	9.24E-10	1.57E-13	GTEx/v8/Brain_Caudate_basal_ganglia:GTEx/v8/Brain_Cerebellum:GTEx/v8/Brain_Cortex:GTEx/v8/Brain_Frontal_Cortex_BA9:GTEx/v8/Brain_Hypothalamus:GTEx/v8/Brain_Nucleus_accumbens_basal_ganglia
rs56023784	ENSG00000163788	*SNRK*	3	43328004	43466256	1	protein_coding	54861	SNRK	0.994142268	-0.871946819	3	1.23E-06	1.61E-06	GTEx/v8/Brain_Cerebellum
rs11130182	ENSG00000164045	*CDC25A*	3	48198636	48229892	-1	protein_coding	993	CDC25A	0.979698045	-0.724000674	31	0.000175411	1.73E-15	GTEx/v8/Brain_Cerebellar_Hemisphere
rs11130182	ENSG00000229759	*MRPS18AP1*	3	48297840	48298428	-1	pseudogene	359761	MRPS18AP1	NA	NA	66	0.000117392	1.29E-23	GTEx/v8/Brain_Cerebellum
rs11130182	ENSG00000145040	*UCN2*	3	48599160	48601206	-1	protein_coding	90226	UCN2	0.157235729	0.006326735	1	0.000301038	0.00573708	GTEx/v8/Brain_Cerebellum
rs11130182	ENSG00000213672	*NCKIPSD*	3	48701364	48723797	-1	protein_coding	51517	NCKIPSD	0.007131362	-0.551018211	149	3.60E-34	8.19E-28	GTEx/v8/Brain_Amygdala:GTEx/v8/Brain_Anterior_cingulate_cortex_BA24:GTEx/v8/Brain_Caudate_basal_ganglia:GTEx/v8/Brain_Cerebellar_Hemisphere:GTEx/v8/Brain_Cerebellum:GTEx/v8/Brain_Cortex:GTEx/v8/Brain_Frontal_Cortex_BA9:GTEx/v8/Brain_Hippocampus:GTEx/v8/Brain_Hypothalamus:GTEx/v8/Brain_Nucleus_accumbens_basal_ganglia:GTEx/v8/Brain_Putamen_basal_ganglia:GTEx/v8/Brain_Spinal_cord_cervical_c-1:GTEx/v8/Brain_Substantia_nigra
rs11130182	ENSG00000178467	*P4HTM*	3	49027319	49044587	1	protein_coding	54681	P4HTM	0.023274936	NA	149	1.50E-11	3.79E-08	GTEx/v8/Brain_Caudate_basal_ganglia:GTEx/v8/Brain_Cerebellar_Hemisphere:GTEx/v8/Brain_Cerebellum:GTEx/v8/Brain_Cortex:GTEx/v8/Brain_Frontal_Cortex_BA9:GTEx/v8/Brain_Hippocampus:GTEx/v8/Brain_Hypothalamus:GTEx/v8/Brain_Nucleus_accumbens_basal_ganglia:GTEx/v8/Brain_Putamen_basal_ganglia:GTEx/v8/Brain_Spinal_cord_cervical_c-1
rs11130182	ENSG00000178252	*WDR6*	3	49044495	49053386	1	protein_coding	11180	WDR6	9.20E-07	-0.058949543	149	2.04E-27	1.30E-21	GTEx/v8/Brain_Amygdala:GTEx/v8/Brain_Caudate_basal_ganglia:GTEx/v8/Brain_Cerebellar_Hemisphere:GTEx/v8/Brain_Cerebellum:GTEx/v8/Brain_Cortex:GTEx/v8/Brain_Frontal_Cortex_BA9:GTEx/v8/Brain_Hippocampus:GTEx/v8/Brain_Nucleus_accumbens_basal_ganglia:GTEx/v8/Brain_Putamen_basal_ganglia:GTEx/v8/Brain_Spinal_cord_cervical_c-1:GTEx/v8/Brain_Substantia_nigra
rs11130182	ENSG00000178149	*DALRD3*	3	49052921	49059726	-1	protein_coding	55152	DALRD3	1.85E-05	0.072306759	149	9.86E-11	1.79E-09	GTEx/v8/Brain_Anterior_cingulate_cortex_BA24:GTEx/v8/Brain_Cerebellar_Hemisphere:GTEx/v8/Brain_Cerebellum:GTEx/v8/Brain_Cortex:GTEx/v8/Brain_Frontal_Cortex_BA9
rs11130182	ENSG00000198218	*QRICH1*	3	49067140	49131796	-1	protein_coding	54870	QRICH1	0.996069748	-0.27929443	145	1.05E-07	3.56E-12	GTEx/v8/Brain_Cerebellar_Hemisphere:GTEx/v8/Brain_Cerebellum
rs11130182	ENSG00000270441	*RP11-694I15.7*	3	49177519	49198284	-1	pseudogene	NA	NA	NA	NA	134	1.38E-05	0.000344885	GTEx/v8/Brain_Cerebellar_Hemisphere:GTEx/v8/Brain_Cerebellum
rs11130182	ENSG00000177352	*CCDC71*	3	49199968	49203754	-1	protein_coding	64925	CCDC71	0.016989639	NA	149	9.42E-20	4.03E-15	GTEx/v8/Brain_Amygdala:GTEx/v8/Brain_Caudate_basal_ganglia:GTEx/v8/Brain_Cerebellar_Hemisphere:GTEx/v8/Brain_Cerebellum:GTEx/v8/Brain_Cortex:GTEx/v8/Brain_Frontal_Cortex_BA9:GTEx/v8/Brain_Hippocampus:GTEx/v8/Brain_Nucleus_accumbens_basal_ganglia:GTEx/v8/Brain_Putamen_basal_ganglia
rs11130182	ENSG00000185909	*KLHDC8B*	3	49209044	49213917	1	protein_coding	200942	KLHDC8B	0.01042142	-0.077352225	149	1.55E-06	0.00138032	GTEx/v8/Brain_Cerebellum
rs11130182	ENSG00000225399	*RP11-3B7.1*	3	49297518	49298744	1	protein_coding	NA	NA	NA	NA	32	7.14E-06	0.00866008	GTEx/v8/Brain_Anterior_cingulate_cortex_BA24
rs11130182	ENSG00000233276	*GPX1*	3	49394609	49396033	-1	protein_coding	2876	GPX1	0.006931651	-0.616399091	149	7.53E-11	5.90E-11	GTEx/v8/Brain_Caudate_basal_ganglia:GTEx/v8/Brain_Cerebellar_Hemisphere:GTEx/v8/Brain_Cerebellum:GTEx/v8/Brain_Cortex:GTEx/v8/Brain_Frontal_Cortex_BA9:GTEx/v8/Brain_Nucleus_accumbens_basal_ganglia
rs11130182	ENSG00000145020	*AMT*	3	49454211	49460186	-1	protein_coding	275	AMT	0.000569475	-0.022637077	149	3.90E-23	7.74E-32	GTEx/v8/Brain_Anterior_cingulate_cortex_BA24:GTEx/v8/Brain_Caudate_basal_ganglia:GTEx/v8/Brain_Cerebellar_Hemisphere:GTEx/v8/Brain_Cerebellum:GTEx/v8/Brain_Cortex:GTEx/v8/Brain_Frontal_Cortex_BA9:GTEx/v8/Brain_Hippocampus:GTEx/v8/Brain_Hypothalamus:GTEx/v8/Brain_Nucleus_accumbens_basal_ganglia:GTEx/v8/Brain_Putamen_basal_ganglia:GTEx/v8/Brain_Spinal_cord_cervical_c-1
rs11130182	ENSG00000145029	*NICN1*	3	49460379	49466759	-1	protein_coding	84276	NICN1	0.257245529	-1.087291051	145	8.90E-07	9.43E-05	GTEx/v8/Brain_Caudate_basal_ganglia:GTEx/v8/Brain_Cortex:GTEx/v8/Brain_Frontal_Cortex_BA9:GTEx/v8/Brain_Nucleus_accumbens_basal_ganglia
rs11130182	ENSG00000226913	*BSN-AS2*	3	49586739	49591799	-1	lincRNA	100132677	BSN-AS2	NA	NA	149	1.69E-07	4.94E-06	GTEx/v8/Brain_Cortex:GTEx/v8/Brain_Frontal_Cortex_BA9:GTEx/v8/Brain_Putamen_basal_ganglia
rs11130182	ENSG00000173540	*GMPPB*	3	49754277	49761384	-1	protein_coding	29925	GMPPB	8.25E-05	-0.65955402	131	8.85E-08	1.80E-28	GTEx/v8/Brain_Amygdala:GTEx/v8/Brain_Anterior_cingulate_cortex_BA24:GTEx/v8/Brain_Cerebellar_Hemisphere:GTEx/v8/Brain_Cerebellum:GTEx/v8/Brain_Cortex:GTEx/v8/Brain_Frontal_Cortex_BA9:GTEx/v8/Brain_Hippocampus:GTEx/v8/Brain_Nucleus_accumbens_basal_ganglia:GTEx/v8/Brain_Putamen_basal_ganglia:GTEx/v8/Brain_Spinal_cord_cervical_c-1:GTEx/v8/Brain_Substantia_nigra
rs9990752	ENSG00000237765	*FAM200B*	4	15683285	15707188	1	protein_coding	285550	FAM200B	NA	-0.311233821	4	3.08E-10	1.86E-12	GTEx/v8/Brain_Anterior_cingulate_cortex_BA24:GTEx/v8/Brain_Caudate_basal_ganglia:GTEx/v8/Brain_Cerebellar_Hemisphere:GTEx/v8/Brain_Cerebellum:GTEx/v8/Brain_Cortex:GTEx/v8/Brain_Frontal_Cortex_BA9:GTEx/v8/Brain_Hypothalamus:GTEx/v8/Brain_Nucleus_accumbens_basal_ganglia:GTEx/v8/Brain_Putamen_basal_ganglia
rs62294142	ENSG00000174125	*TLR1*	4	38792298	38858438	-1	protein_coding	7096	TLR1	2.02E-14	0.436460923	1	4.45E-05	0.0324857	GTEx/v8/Brain_Cerebellum
rs6828811	ENSG00000014824	*SLC30A9*	4	41992489	42092474	1	protein_coding	10463	SLC30A9	0.001423485	-0.907696884	13	1.81E-06	2.46E-09	GTEx/v8/Brain_Caudate_basal_ganglia:GTEx/v8/Brain_Hypothalamus:GTEx/v8/Brain_Nucleus_accumbens_basal_ganglia
rs6828811	ENSG00000188848	*BEND4*	4	42112955	42154895	-1	protein_coding	389206	BEND4	0.986764686	1.717247406	20	1.95E-06	0.012642	GTEx/v8/Brain_Hippocampus
rs79217540	ENSG00000177822	*AC108142.1*	4	182795591	183066402	-1	antisense	NA	NA	NA	NA	1	2.49E-05	7.26E-06	GTEx/v8/Brain_Nucleus_accumbens_basal_ganglia
rs62372027	ENSG00000250422	*RP11-484L7.1*	5	45558021	45560088	1	pseudogene	NA	NA	NA	NA	89	5.05E-05	0.0275529	GTEx/v8/Brain_Cerebellar_Hemisphere

rs972501	ENSG00000123219	*CENPK*	5	64813593	64858998	-1	protein_coding	64105	CENPK	1.66E-08	0.339527267	271	1.08E-07	3.17E-09	GTEx/v8/Brain_Cerebellar_Hemisphere:GTEx/v8/Brain_Cerebellum:GTEx/v8/Brain_Nucleus_accumbens_basal_ganglia
rs972501	ENSG00000113593	*PPWD1*	5	64859063	64883376	1	protein_coding	23398	PPWD1	0.031785775	0.411549096	274	4.10E-17	2.93E-12	GTEx/v8/Brain_Amygdala:GTEx/v8/Brain_Anterior_cingulate_cortex_BA24:GTEx/v8/Brain_Caudate_basal_ganglia:GTEx/v8/Brain_Cerebellar_Hemisphere:GTEx/v8/Brain_Cerebellum:GTEx/v8/Brain_Cortex:GTEx/v8/Brain_Frontal_Cortex_BA9:GTEx/v8/Brain_Hippocampus:GTEx/v8/Brain_Hypothalamus:GTEx/v8/Brain_Nucleus_accumbens_basal_ganglia:GTEx/v8/Brain_Putamen_basal_ganglia:GTEx/v8/Brain_Spinal_cord_cervical_c-1:GTEx/v8/Brain_Substantia_nigra
rs972501	ENSG00000253251	*CTC-534A2.2*	5	64920592	64926718	1	protein_coding	80006	TRAPPC13	NA	NA	272	4.10E-07	0.00103566	GTEx/v8/Brain_Cerebellum:GTEx/v8/Brain_Cortex:GTEx/v8/Brain_Frontal_Cortex_BA9:GTEx/v8/Brain_Hypothalamus:GTEx/v8/Brain_Nucleus_accumbens_basal_ganglia:GTEx/v8/Brain_Spinal_cord_cervical_c-1
rs6882046	ENSG00000250377	*CTC-467M3.3*	5	87988462	87989789	-1	lincRNA	NA	NA	NA	NA	1	7.88E-05	0.0026799	GTEx/v8/Brain_Cortex:GTEx/v8/Brain_Frontal_Cortex_BA9
rs184937	ENSG00000145808	*ADAMTS19*	5	128795958	129074376	1	protein_coding	171019	ADAMTS19	0.951958941	0.787855457	3	7.06E-05	0.015434	GTEx/v8/Brain_Cortex
rs4463213	ENSG00000113119	*TMCO6*	5	140019012	140024993	1	protein_coding	55374	TMCO6	8.15E-06	-0.195921903	34	8.57E-05	3.32E-13	GTEx/v8/Brain_Cerebellar_Hemisphere
rs4463213	ENSG00000120314	*WDR55*	5	140044261	140053709	1	protein_coding	54853	WDR55	1.48E-06	-1.037518539	3	3.25E-05	8.26E-07	GTEx/v8/Brain_Cerebellum
rs7754169	ENSG00000135341	*MAP3K7*	6	91223292	91296764	-1	protein_coding	6885	MAP3K7	0.998336232	0.205430404	31	9.41E-06	0.00610124	GTEx/v8/Brain_Frontal_Cortex_BA9
rs240154	ENSG00000112249	*ASCC3*	6	100956070	101329248	-1	protein_coding	10973	ASCC3	1.43E-10	0.106685768	199	3.78E-09	1.60E-05	GTEx/v8/Brain_Caudate_basal_ganglia:GTEx/v8/Brain_Cerebellum:GTEx/v8/Brain_Cortex
rs240154	ENSG00000260000	*RP3-467N11.1*	6	101329347	101330863	1	antisense	NA	NA	NA	NA	82	5.49E-06	0.0118812	GTEx/v8/Brain_Anterior_cingulate_cortex_BA24:GTEx/v8/Brain_Nucleus_accumbens_basal_ganglia
rs7768857	ENSG00000164483	*SAMD3*	6	130465460	130686570	-1	protein_coding	154075	SAMD3	1.10E-06	0.088198284	58	4.49E-07	0.00198934	GTEx/v8/Brain_Cortex
rs844584	ENSG00000203727	*SAMD5*	6	147830063	148058683	1	protein_coding	389432	SAMD5	0.000898004	1.680995061	28	2.82E-09	2.81E-10	GTEx/v8/Brain_Cerebellum:GTEx/v8/Brain_Hypothalamus
rs6244881	ENSG00000176349	*AC110781.3*	7	1878222	1889567	1	protein_coding	NA	NA	NA	NA	74	3.26E-07	2.61E-06	GTEx/v8/Brain_Nucleus_accumbens_basal_ganglia
rs6244881	ENSG00000122687	*FTSJ2*	7	2273866	2281840	-1	protein_coding	29960	FTSJ2	1.97E-05	0.096595649	95	1.66E-07	4.01E-12	GTEx/v8/Brain_Caudate_basal_ganglia:GTEx/v8/Brain_Cerebellar_Hemisphere:GTEx/v8/Brain_Cortex
rs7805419	ENSG00000106460	*TMEM106B*	7	12250867	12282993	1	protein_coding	54664	TMEM106B	0.515078464	3.380649935	115	2.12E-07	0.00109844	GTEx/v8/Brain_Cerebellum:GTEx/v8/Brain_Cortex
rs1986692	ENSG00000155530	*LRGUK*	7	133812052	133949343	1	protein_coding	136332	LRGUK	1.92E-08	-0.271387377	1	6.66E-07	0.000172206	GTEx/v8/Brain_Cerebellar_Hemisphere:GTEx/v8/Brain_Cerebellum
rs9800952	ENSG00000090263	*MRPS33*	7	140705854	140715028	-1	protein_coding	51650	MRPS33	0.003395718	0.268392925	51	2.30E-09	1.80E-05	GTEx/v8/Brain_Caudate_basal_ganglia:GTEx/v8/Brain_Cortex:GTEx/v8/Brain_Putamen_basal_ganglia
rs55768139	ENSG00000261451	*RP11-981G7.1*	8	10291182	10295822	1	sense_overlapping	NA	NA	NA	NA	16	3.22E-09	6.07E-19	GTEx/v8/Brain_Cerebellar_Hemisphere:GTEx/v8/Brain_Cerebellum:GTEx/v8/Brain_Cortex
rs876575	ENSG00000153317	*ASAP1*	8	131064353	131455906	-1	protein_coding	50807	ASAP1	0.999983091	0.592920078	50	1.01E-08	1.01E-16	GTEx/v8/Brain_Cerebellar_Hemisphere:GTEx/v8/Brain_Cerebellum:GTEx/v8/Brain_Cortex:GTEx/v8/Brain_Nucleus_accumbens_basal_ganglia:GTEx/v8/Brain_Putamen_basal_ganglia
rs1927904	ENSG00000233569	*RP11-500B12.1*	9	120410884	120419305	1	lincRNA	101928797	LOC101928797	NA	NA	2	4.01E-06	2.85E-14	GTEx/v8/Brain_Cerebellum
rs4837010	ENSG00000185585	*OLFML2A*	9	127539437	127577164	1	protein_coding	169611	OLFML2A	1.74E-05	0.451719558	69	4.51E-09	1.77E-05	GTEx/v8/Brain_Caudate_basal_ganglia:GTEx/v8/Brain_Putamen_basal_ganglia
rs4837010	ENSG00000136950	*ARPC5L*	9	127624409	127640003	1	protein_coding	81873	ARPC5L	0.775599153	-1.27069518	70	6.30E-16	1.03E-17	GTEx/v8/Brain_Cerebellar_Hemisphere:GTEx/v8/Brain_Cerebellum:GTEx/v8/Brain_Hypothalamus:GTEx/v8/Brain_Putamen_basal_ganglia
rs4837010	ENSG00000136935	*GOLGA1*	9	127640646	127710771	-1	protein_coding	2800	GOLGA1	0.006107394	-0.699247797	68	1.35E-05	7.80E-05	GTEx/v8/Brain_Cerebellar_Hemisphere:GTEx/v8/Brain_Cerebellum
rs4837010	ENSG00000173611	*SCAI*	9	127704887	127905785	-1	protein_coding	286205	SCAI	0.999445289	NA	42	3.77E-06	0.0144518	GTEx/v8/Brain_Cortex:GTEx/v8/Brain_Hippocampus
rs4837010	ENSG00000119414	*PPP6C*	9	127908852	127952218	-1	protein_coding	5537	PPP6C	0.988609354	-0.703511544	70	9.66E-10	8.55E-06	GTEx/v8/Brain_Anterior_cingulate_cortex_BA24:GTEx/v8/Brain_Caudate_basal_ganglia:GTEx/v8/Brain_Frontal_Cortex_BA9:GTEx/v8/Brain_Hippocampus:GTEx/v8/Brain_Nucleus_accumbens_basal_ganglia:GTEx/v8/Brain_Putamen_basal_ganglia
rs4837010	ENSG00000232630	*PRPS1P2*	9	127912932	127913868	1	pseudogene	100421295	PRPS1P2	NA	NA	70	4.90E-11	3.26E-07	GTEx/v8/Brain_Cerebellar_Hemisphere:GTEx/v8/Brain_Cerebellum:GTEx/v8/Brain_Hippocampus:GTEx/v8/Brain_Nucleus_accumbens_basal_ganglia
rs4837010	ENSG00000165219	*GAPVD1*	9	128024073	128129486	1	protein_coding	26130	GAPVD1	0.999999872	-0.165865959	53	1.95E-05	9.64E-08	GTEx/v8/Brain_Cerebellar_Hemisphere:GTEx/v8/Brain_Cerebellum
rs2399576	ENSG00000225383	*SFTA1P*	10	10826400	10836943	-1	lincRNA	207107	SFTA1P	NA	NA	37	1.00E-12	4.87E-11	GTEx/v8/Brain_Cortex:GTEx/v8/Brain_Frontal_Cortex_BA9:GTEx/v8/Brain_Hippocampus:GTEx/v8/Brain_Hypothalamus:GTEx/v8/Brain_Nucleus_accumbens_basal_ganglia:GTEx/v8/Brain_Putamen_basal_ganglia:GTEx/v8/Brain_Spinal_cord_cervical_c-1

rs10883434	ENSG00000095485	*CWF19L1*	10	101992055	102027437	-1	protein_coding	55280	CWF19L1	5.69E-09	0.127598735	14	4.27E-08	1.17E-31	GTEx/v8/Brain_Caudate_basal_ganglia:GTEx/v8/Brain_Cerebellum:GTEx/v8/Brain_Cortex:GTEx/v8/Brain_Frontal_Cortex_BA9:GTEx/v8/Brain_Hypothalamus:GTEx/v8/Brain_Nucleus_accumbens_basal_ganglia
rs72898691	ENSG00000205106	*CTD-2210P24.4*	11	45792983	45793909	1	protein_coding	374387	DKFZp779M0652	NA	NA	14	2.36E-08	3.31E-08	GTEx/v8/Brain_Caudate_basal_ganglia:GTEx/v8/Brain_Cortex:GTEx/v8/Brain_Frontal_Cortex_BA9:GTEx/v8/Brain_Nucleus_accumbens_basal_ganglia:GTEx/v8/Brain_Putamen_basal_ganglia
rs78519907	ENSG00000086205	*FOLH1*	11	49168187	49230222	-1	protein_coding	2346	FOLH1	0.253396885	-0.094158315	39	1.67E-06	1.48E-06	GTEx/v8/Brain_Amygdala:GTEx/v8/Brain_Caudate_basal_ganglia:GTEx/v8/Brain_Cerebellum:GTEx/v8/Brain_Cortex:GTEx/v8/Brain_Nucleus_accumbens_basal_ganglia:GTEx/v8/Brain_Putamen_basal_ganglia:GTEx/v8/Brain_Spinal_cord_cervical_c-1
rs78519907	ENSG00000254714	*RP11-163O19.1*	11	49872181	49884080	1	pseudogene	NA	NA	NA	NA	4	0.000153193	0.000158926	GTEx/v8/Brain_Caudate_basal_ganglia
rs78519907	ENSG00000255190	*TRIM51DP*	11	49896497	49898190	1	pseudogene	100419945	TRIM51DP	NA	NA	15	9.16E-05	8.08E-23	GTEx/v8/Brain_Cortex
rs499188	ENSG00000149150	*SLC43A1*	11	57252007	57283259	-1	protein_coding	8501	SLC43A1	7.85E-05	-0.136928307	5	4.46E-05	2.48E-08	GTEx/v8/Brain_Cerebellum
rs499188	ENSG00000134809	*TIMM10*	11	57295936	57298276	-1	protein_coding	26519	TIMM10	0.009494762	0.000100078	15	1.68E-05	7.82E-13	GTEx/v8/Brain_Caudate_basal_ganglia
rs499188	ENSG00000156603	*MED19*	11	57471186	57479693	-1	protein_coding	219541	MED19	0.683790051	-0.151700149	83	5.44E-06	5.15E-07	GTEx/v8/Brain_Cerebellar_Hemisphere:GTEx/v8/Brain_Cerebellum
rs499188	ENSG00000242689	*CNTF*	11	58390146	58393198	1	protein_coding	1270	CNTF	0.000349252	-0.109964842	48	1.60E-05	0.0192174	GTEx/v8/Brain_Cortex
rs99780	ENSG00000134825	*TMEM258*	11	61535973	61560274	-1	protein_coding	746	TMEM258	0.000864646	0.122739186	41	1.36E-06	0.00433323	GTEx/v8/Brain_Frontal_Cortex_BA9:GTEx/v8/Brain_Putamen_basal_ganglia
rs99780	ENSG00000149485	*FADS1*	11	61567099	61596790	-1	protein_coding	3992	FADS1	0.383801127	-0.763752724	55	7.63E-33	3.14E-26	GTEx/v8/Brain_Anterior_cingulate_cortex_BA24:GTEx/v8/Brain_Caudate_basal_ganglia:GTEx/v8/Brain_Cerebellar_Hemisphere:GTEx/v8/Brain_Cerebellum:GTEx/v8/Brain_Cortex:GTEx/v8/Brain_Frontal_Cortex_BA9:GTEx/v8/Brain_Hippocampus:GTEx/v8/Brain_Hypothalamus:GTEx/v8/Brain_Putamen_basal_ganglia:GTEx/v8/Brain_Spinal_cord_cervical_c-1
rs99780	ENSG00000221968	*FADS3*	11	61640991	61659523	-1	protein_coding	3995	FADS3	0.048872245	NA	50	1.79E-11	1.73E-07	GTEx/v8/Brain_Cerebellar_Hemisphere:GTEx/v8/Brain_Cerebellum
rs45519937	ENSG00000170209	*ANKK1*	11	113258513	113271140	1	protein_coding	255239	ANKK1	2.51E-08	-0.017548171	6	1.06E-08	2.09E-11	GTEx/v8/Brain_Cerebellar_Hemisphere:GTEx/v8/Brain_Cerebellum:GTEx/v8/Brain_Cortex:GTEx/v8/Brain_Frontal_Cortex_BA9
rs612823	ENSG00000080854	*IGSF9B*	11	133778459	133826880	-1	protein_coding	22997	IGSF9B	0.999873394	NA	1	6.52E-09	5.01E-09	GTEx/v8/Brain_Cerebellum
rs11223816	ENSG00000255545	*RP11-627G23.1*	11	134306367	134375555	1	processed_transcript	NA	NA	NA	NA	1	1.12E-05	2.98E-30	GTEx/v8/Brain_Frontal_Cortex_BA9
rs7313797	ENSG00000151148	*UBE3B*	12	109915207	109974507	1	protein_coding	89910	UBE3B	0.009387706	0.786225851	38	1.23E-06	3.10E-05	GTEx/v8/Brain_Cerebellum
rs7313797	ENSG00000139428	*MMAB*	12	109991542	110011679	-1	protein_coding	326625	MMAB	0.00387716	2.918192677	76	6.29E-09	6.67E-12	GTEx/v8/Brain_Caudate_basal_ganglia:GTEx/v8/Brain_Cerebellar_Hemisphere:GTEx/v8/Brain_Cerebellum:GTEx/v8/Brain_Cortex:GTEx/v8/Brain_Frontal_Cortex_BA9:GTEx/v8/Brain_Nucleus_accumbens_basal_ganglia
rs6490177	ENSG00000111707	*SUDS3*	12	118814185	118855840	1	protein_coding	64426	SUDS3	0.007596107	1.082282657	231	1.04E-06	0.00533451	GTEx/v8/Brain_Cortex:GTEx/v8/Brain_Hippocampus:GTEx/v8/Brain_Nucleus_accumbens_basal_ganglia:GTEx/v8/Brain_Putamen_basal_ganglia
rs9527083	ENSG00000225510	*PCDH8P1*	13	53774116	53776547	1	pseudogene	100133285	PCDH8P1	NA	NA	12	5.33E-06	0.00408458	GTEx/v8/Brain_Anterior_cingulate_cortex_BA24
rs6492656	ENSG00000183098	*GPC6*	13	93879095	95059655	1	protein_coding	10082	GPC6	0.338854613	-0.297958301	2	1.21E-05	7.99E-07	GTEx/v8/Brain_Caudate_basal_ganglia
rs9517301	ENSG00000152767	*FARP1*	13	98794816	99102027	1	protein_coding	10160	FARP1	0.045263074	-0.444415571	9	4.83E-08	0.000197041	GTEx/v8/Brain_Cerebellum
rs9517301	ENSG00000102572	*STK24*	13	99102455	99230194	-1	protein_coding	8428	STK24	0.797998297	0.417068263	9	2.01E-11	3.45E-07	GTEx/v8/Brain_Cerebellar_Hemisphere:GTEx/v8/Brain_Cerebellum
rs8019784	ENSG00000139915	*MDGA2*	14	47308826	48144157	-1	protein_coding	161357	MDGA2	0.993216871	0.397758699	47	9.51E-06	0.0198975	GTEx/v8/Brain_Caudate_basal_ganglia:GTEx/v8/Brain_Cerebellar_Hemisphere:GTEx/v8/Brain_Cortex:GTEx/v8/Brain_Nucleus_accumbens_basal_ganglia
rs7147721	ENSG00000119682	*AREL1*	14	75120140	75179818	-1	protein_coding	9870	AREL1	0.011802084	-0.560094839	92	7.54E-10	1.08E-05	GTEx/v8/Brain_Caudate_basal_ganglia:GTEx/v8/Brain_Cerebellar_Hemisphere:GTEx/v8/Brain_Cortex:GTEx/v8/Brain_Frontal_Cortex_BA9
rs7147721	ENSG00000119689	*DLST*	14	75348594	75370448	1	protein_coding	1743	DLST	0.950393652	0.114612171	7	8.30E-06	0.0103043	GTEx/v8/Brain_Cerebellum
rs7147721	ENSG00000119640	*ACYP1*	14	75519924	75536186	-1	protein_coding	97	ACYP1	0.223001664	-0.092741837	6	8.53E-06	0.0256698	GTEx/v8/Brain_Frontal_Cortex_BA9
rs7147721	ENSG00000119703	*ZC2HC1C*	14	75530873	75545126	1	protein_coding	79696	ZC2HC1C	1.62E-06	-0.426	1	1.29E-05	2.55E-05	GTEx/v8/Brain_Cortex

											601893				
rs12884892	ENSG00000246223	*C14orf64*	14	98391947	98444461	-1	protein_coding	388011	C14orf64	NA	NA	7	1.20E-07	0.000908772	GTEx/v8/Brain_Caudate_basal_ganglia
rs942866	ENSG00000075413	*MARK3*	14	103851729	103970168	1	protein_coding	4140	MARK3	6.11E-05	-0.099970289	3	3.01E-05	0.000395216	GTEx/v8/Brain_Frontal_Cortex_BA9
rs942866	ENSG00000166166	*TRMT61A*	14	103995521	104003410	1	protein_coding	115708	TRMT61A	0.005702065	0.11345694	53	7.22E-09	8.83E-05	GTEx/v8/Brain_Cortex:GTEx/v8/Brain_Frontal_Cortex_BA9
rs942866	ENSG00000258851	*RP11-894P9.2*	14	104019758	104028214	1	antisense	NA	NA	NA	NA	4	1.52E-05	0.0303807	GTEx/v8/Brain_Cerebellar_Hemisphere
rs942866	ENSG00000166170	*BAG5*	14	104022881	104029168	-1	protein_coding	9529	BAG5	0.043218188	-0.768926857	40	7.54E-07	0.00184945	GTEx/v8/Brain_Anterior_cingulate_cortex_BA24:GTEx/v8/Brain_Cerebellar_Hemisphere:GTEx/v8/Brain_Cerebellum:GTEx/v8/Brain_Hypothalamus
rs942866	ENSG00000126214	*KLC1*	14	104028233	104167888	1	protein_coding	3831	KLC1	0.514955332	NA	43	7.67E-08	3.20E-05	GTEx/v8/Brain_Cerebellar_Hemisphere:GTEx/v8/Brain_Cortex
rs942866	ENSG00000256053	*APOPT1*	14	104029299	104073860	1	protein_coding	84334	APOPT1	0.000361376	-0.703676152	17	1.83E-05	1.30E-07	GTEx/v8/Brain_Frontal_Cortex_BA9:GTEx/v8/Brain_Nucleus_accumbens_basal_ganglia
rs942866	ENSG00000269958	*RP11-73M18.8*	14	104162690	104163500	1	sense_intronic	NA	NA	NA	NA	54	3.12E-10	5.82E-08	GTEx/v8/Brain_Amygdala:GTEx/v8/Brain_Caudate_basal_ganglia:GTEx/v8/Brain_Cortex:GTEx/v8/Brain_Frontal_Cortex_BA9:GTEx/v8/Brain_Hippocampus:GTEx/v8/Brain_Hypothalamus:GTEx/v8/Brain_Nucleus_accumbens_basal_ganglia:GTEx/v8/Brain_Putamen_basal_ganglia:GTEx/v8/Brain_Spinal_cord_cervical_c-1:GTEx/v8/Brain_Substantia_nigra
rs942866	ENSG00000126215	*XRCC3*	14	104163946	104181841	-1	protein_coding	7517	XRCC3	6.32E-08	0.467663173	43	9.53E-09	1.25E-09	GTEx/v8/Brain_Cerebellar_Hemisphere
rs921764	ENSG00000137872	*SEMA6D*	15	4747629 8	48066420	1	protein_coding	80031	SEMA6D	0.999469976	-0.259466766	75	7.50E-08	2.85E-05	GTEx/v8/Brain_Cerebellum
rs6224	ENSG00000140564	*FURIN*	15	91411822	91426688	1	protein_coding	5045	FURIN	0.999933301	-0.156379719	1	2.51E-05	0.0433875	GTEx/v8/Brain_Frontal_Cortex_BA9
rs34541615	ENSG00000102931	*ARL2BP*	16	57279010	57287516	1	protein_coding	23568	ARL2BP	0.000774156	-0.164113324	2	1.97E-06	0.00743772	GTEx/v8/Brain_Cerebellar_Hemisphere
rs499188	ENSG00000265566	*RN7SL605P*	17	2657126	2657430	1	misc_RNA	106481087	RN7SL605P	NA	NA	1	0.000105864	5.83E-09	GTEx/v8/Brain_Frontal_Cortex_BA9
rs3597978 4	ENSG00000270091	*RP11-78O7.2*	17	19799903	19800600	-1	lincRNA	NA	NA	NA	NA	33	2.78E-08	1.71E-08	GTEx/v8/Brain_Cerebellum
rs35979784	ENSG00000261033	*RP11-209D14.2*	17	19911364	19912547	-1	antisense	NA	NA	NA	NA	128	5.69E-17	4.10E-12	GTEx/v8/Brain_Amygdala:GTEx/v8/Brain_Anterior_cingulate_cortex_BA24:GTEx/v8/Brain_Caudate_basal_ganglia:GTEx/v8/Brain_Cerebellar_Hemisphere:GTEx/v8/Brain_Cerebellum:GTEx/v8/Brain_Cortex:GTEx/v8/Brain_Frontal_Cortex_BA9:GTEx/v8/Brain_Hippocampus:GTEx/v8/Brain_Hypothalamus:GTEx/v8/Brain_Nucleus_accumbens_basal_ganglia:GTEx/v8/Brain_Putamen_basal_ganglia:GTEx/v8/Brain_Spinal_cord_cervical_c-1
rs35979784	ENSG00000128487	*SPECC1*	17	19912657	20222339	1	protein_coding	92521	SPECC1	0.10945964	0.008122998	105	6.32E-07	3.13E-05	GTEx/v8/Brain_Cortex:GTEx/v8/Brain_Substantia_nigra
rs35979784	ENSG00000154898	*CCDC144CP*	17	20224477	20306870	1	pseudogene	348254	CCDC144CP	NA	NA	1	0.000105426	2.06E-05	GTEx/v8/Brain_Cerebellum
rs35979784	ENSG00000189423	*USP32P3*	17	20318860	20334321	1	pseudogene	347716	USP32P3	NA	NA	33	5.31E-06	0.000487042	GTEx/v8/Brain_Cerebellar_Hemisphere:GTEx/v8/Brain_Putamen_basal_ganglia
rs35979784	ENSG00000237911	*SRP68P3*	17	20319105	20320010	-1	pseudogene	347717	SRP68P3	NA	NA	101	1.71E-07	3.68E-06	GTEx/v8/Brain_Cerebellar_Hemisphere:GTEx/v8/Brain_Cerebellum
rs35979784	ENSG00000230528	*NOS2P3*	17	20339650	20350557	1	pseudogene	339256	NOS2P3	NA	NA	128	1.91E-19	1.23E-17	GTEx/v8/Brain_Amygdala:GTEx/v8/Brain_Anterior_cingulate_cortex_BA24:GTEx/v8/Brain_Caudate_basal_ganglia:GTEx/v8/Brain_Cerebellar_Hemisphere:GTEx/v8/Brain_Cerebellum:GTEx/v8/Brain_Cortex:GTEx/v8/Brain_Frontal_Cortex_BA9:GTEx/v8/Brain_Hippocampus:GTEx/v8/Brain_Hypothalamus:GTEx/v8/Brain_Nucleus_accumbens_basal_ganglia:GTEx/v8/Brain_Putamen_basal_ganglia:GTEx/v8/Brain_Spinal_cord_cervical_c-1:GTEx/v8/Brain_Substantia_nigra
rs35979784	ENSG00000231645	*AC025627.9*	17	20415873	20420792	-1	pseudogene	NA	NA	NA	NA	27	4.94E-06	0.00671679	GTEx/v8/Brain_Cerebellar_Hemisphere
rs1893947	ENSG00000170558	*CDH2*	18	25530930	25757410	-1	protein_coding	1000	CDH2	0.897440931	-0.139325903	53	6.57E-08	5.39E-05	GTEx/v8/Brain_Cerebellar_Hemisphere:GTEx/v8/Brain_Cerebellum
rs6567291	ENSG00000141664	*ZCCHC2*	18	60190240	60254942	1	protein_coding	54877	ZCCHC2	0.305659357	-0.580417645	5	1.57E-05	0.0301536	GTEx/v8/Brain_Cerebellum
rs192435	ENSG00000269392	*CTB-191K22.6*	19	50869137	50872588	-1	antisense	NA	NA	NA	NA	4	8.73E-06	0.014716	GTEx/v8/Brain_Caudate_basal_ganglia
rs192435	ENSG00000142549	*IGLON5*	19	51815102	51833608	1	protein_coding	402665	IGLON5	0.822062298	-0.237406742	28	5.22E-09	6.65E-05	GTEx/v8/Brain_Caudate_basal_ganglia:GTEx/v8/Brain_Nucleus_accumbens_basal_ganglia
rs192435	ENSG00000267905	*CTD-2616J11.16*	19	51843949	51847371	1	antisense	NA	NA	NA	NA	30	5.18E-09	8.22E-12	GTEx/v8/Brain_Cerebellum

rs192435	ENSG00000198093	*ZNF649*	19	52392477	52408293	-1	protein_coding	65251	ZNF649	0.711121458	0.454717805	2	1.59E-05	2.39E-08	GTEx/v8/Brain_Nucleus_accumbens_basal_ganglia
rs34478401	ENSG00000089091	*DZANK1*	20	18364011	18447925	-1	protein_coding	55184	DZANK1	4.60E-15	0.257765494	10	2.45E-05	8.29E-07	GTEx/v8/Brain_Cerebellar_Hemisphere:GTEx/v8/Brain_Frontal_Cortex_BA9
rs34478401	ENSG00000089050	*RBBP9*	20	18467184	18477887	-1	protein_coding	10741	RBBP9	0.103910134	0.941148901	89	6.50E-07	0.00184617	GTEx/v8/Brain_Caudate_basal_ganglia
rs34478401	ENSG00000101310	*SEC23B*	20	18488137	18542059	1	protein_coding	10483	SEC23B	6.86E-14	-0.826131695	66	1.21E-06	1.34E-25	GTEx/v8/Brain_Cerebellar_Hemisphere:GTEx/v8/Brain_Cerebellum:GTEx/v8/Brain_Cortex:GTEx/v8/Brain_Nucleus_accumbens_basal_ganglia
rs34478401	ENSG00000125821	*DTD1*	20	18568537	18744561	1	protein_coding	92675	DTD1	0.290212859	-0.008364778	155	2.57E-09	3.54E-05	GTEx/v8/Brain_Amygdala:GTEx/v8/Brain_Anterior_cingulate_cortex_BA24:GTEx/v8/Brain_Caudate_basal_ganglia:GTEx/v8/Brain_Cortex:GTEx/v8/Brain_Hypothalamus:GTEx/v8/Brain_Nucleus_accumbens_basal_ganglia
rs34478401	ENSG00000179935	*LINC00652*	20	18768295	18774979	-1	lincRNA	29075	LINC00652	NA	NA	87	1.06E-06	0.0101316	GTEx/v8/Brain_Amygdala
rs1974652	ENSG00000236540	*AC006547.13*	22	20050503	20058066	-1	antisense	NA	NA	NA	NA	21	3.87E-06	0.000102016	GTEx/v8/Brain_Caudate_basal_ganglia:GTEx/v8/Brain_Cortex
rs1974652	ENSG00000099899	*TRMT2A*	22	20099389	20104915	-1	protein_coding	27037	TRMT2A	1.31E-05	NA	5	2.59E-05	0.0244377	GTEx/v8/Brain_Cerebellar_Hemisphere
rs1974652	ENSG00000099904	*ZDHHC8*	22	20116979	20135530	1	protein_coding	29801	ZDHHC8	0.988991096	NA	7	9.68E-06	0.000390337	GTEx/v8/Brain_Caudate_basal_ganglia:GTEx/v8/Brain_Cerebellum
rs926914	ENSG00000128285	*MCHR1*	22	41074754	41078818	1	protein_coding	2847	MCHR1	0.001148393	-0.081006493	6	1.89E-05	8.11E-10	GTEx/v8/Brain_Cerebellum
rs926914	ENSG00000100372	*SLC25A17*	22	41165634	41215403	-1	protein_coding	10478	SLC25A17	0.024184844	-0.779478694	42	4.95E-09	2.58E-05	GTEx/v8/Brain_Cerebellum:GTEx/v8/Brain_Nucleus_accumbens_basal_ganglia:GTEx/v8/Brain_Putamen_basal_ganglia
rs926914	ENSG00000196236	*XPNPEP3*	22	41253081	41363838	1	protein_coding	63929	XPNPEP3	8.88E-06	-0.745193699	1	0.000192103	7.04E-05	GTEx/v8/Brain_Cerebellar_Hemisphere
rs926914	ENSG00000213857	*RP11-12M9.4*	22	41470184	41471243	-1	pseudogene	NA	NA	NA	NA	7	8.01E-05	0.0176512	GTEx/v8/Brain_Cerebellum
rs926914	ENSG00000100393	*EP300*	22	41487790	41576081	1	protein_coding	2033	EP300	1	NA	113	2.44E-06	0.00184947	GTEx/v8/Brain_Cerebellum:GTEx/v8/Brain_Hypothalamus
rs926914	ENSG00000100395	*L3MBTL2*	22	41601209	41627275	1	protein_coding	83746	L3MBTL2	0.013028043	-0.618259224	121	1.80E-08	5.18E-05	GTEx/v8/Brain_Anterior_cingulate_cortex_BA24:GTEx/v8/Brain_Frontal_Cortex_BA9
rs926914	ENSG00000100401	*RANGAP1*	22	41641615	41682255	-1	protein_coding	5905	RANGAP1	0.13271872	-0.376521602	77	7.87E-07	1.39E-06	GTEx/v8/Brain_Cerebellar_Hemisphere:GTEx/v8/Brain_Cerebellum
rs926914	ENSG00000100403	*ZC3H7B*	22	41697526	41756151	1	protein_coding	23264	ZC3H7B	0.990761551	-0.628157842	125	4.49E-08	0.00010307	GTEx/v8/Brain_Cerebellum
rs926914	ENSG00000100412	*ACO2*	22	41865129	41924993	1	protein_coding	50	ACO2	0.454377775	-0.617411984	21	2.91E-05	0.00290367	GTEx/v8/Brain_Cerebellum
rs926914	ENSG00000100413	*POLR3H*	22	41921808	41940610	-1	protein_coding	171568	POLR3H	0.002007106	-1.305852274	121	2.19E-08	8.24E-14	GTEx/v8/Brain_Cerebellum:GTEx/v8/Brain_Cortex:GTEx/v8/Brain_Frontal_Cortex_BA9:GTEx/v8/Brain_Putamen_basal_ganglia
rs926914	ENSG00000172346	*CSDC2*	22	41956767	41973745	1	protein_coding	27254	CSDC2	0.079803516	0.31935561	120	2.14E-09	6.71E-18	GTEx/v8/Brain_Cortex:GTEx/v8/Brain_Frontal_Cortex_BA9
rs926914	ENSG00000167077	*MEI1*	22	42095503	42195460	1	protein_coding	150365	MEI1	1.22E-08	0.500155337	115	1.71E-06	1.60E-05	GTEx/v8/Brain_Anterior_cingulate_cortex_BA24:GTEx/v8/Brain_Frontal_Cortex_BA9
rs926914	ENSG00000159958	*TNFRSF13C*	22	42321045	42322822	-1	protein_coding	115650	TNFRSF13C	0.641054688	-0.063981155	31	1.87E-05	1.22E-11	GTEx/v8/Brain_Cerebellar_Hemisphere
rs926914	ENSG00000205704	*LINC00634*	22	42348169	42354937	1	pseudogene	339674	LINC00634	NA	NA	9	6.19E-05	0.000116414	GTEx/v8/Brain_Frontal_Cortex_BA9
rs926914	ENSG00000183066	*WBP2NL*	22	42394729	42454460	1	protein_coding	164684	WBP2NL	2.07E-09	0.730692723	1	7.68E-05	1.04E-06	GTEx/v8/Brain_Frontal_Cortex_BA9
rs926914	ENSG00000198951	*NAGA*	22	42454358	42466846	-1	protein_coding	4668	NAGA	2.02E-06	0.183467033	5	0.000101399	7.04E-29	GTEx/v8/Brain_Cerebellum
rs926914	ENSG00000100197	*CYP2D6*	22	42522501	42526908	-1	protein_coding	1565	CYP2D6	9.23E-10	0.153953666	11	2.97E-05	3.61E-26	GTEx/v8/Brain_Cerebellum

rs7410394	ENSG00000182257	*C22orf26*	22	46445358	46450024	-1	protein_coding	55267	C22orf26	0.035846022	-0.053561593	8	1.68E-06	0.000561202	GTEx/v8/Brain_Caudate_basal_ganglia

Abbreviations: pLI, probability of loss of function intolerance; ncRVIS, non-coding residual variation intolerance score; eqtlMapSNPs, the number of SNPs mapped to the gene based on eQTL mapping; eqtlMapminP, mininum eQTL P value of mapped SNPs; eqtlMapminQ, mininum eQTL FDR of mapped SNPs; eQTL Mapts, tissue types of mapped eQTL SNPs (GTEx v8 Brain was selected).

Supplementary Table 22. Two-stage GWAS results for 10 randomly divided data sets

CHR	SNPID		Set 1			Set 2			Set 3		
		Overall P	Discovery P	Replication P	Meta-analysis P	Discovery P	Replication P	Meta-analysis P	Discovery P	Replication P	Meta-analysis P
2	rs4953150	4.05×10^{-9}	1.7E-09	4.9E-01	3.2E-09	4.0E-08	2.1E-02	2.8E-09	7.7E-08	6.9E-03	2.5E-09
2	rs7573001	4.07×10^{-8}	6.8E-08	1.4E-01	2.4E-08	1.2E-07	5.5E-02	1.8E-08	8.6E-08	9.6E-02	2.1E-08
5	rs13166120	2.39×10^{-9}	5.8E-09	6.8E-02	1.1E-09	4.6E-08	4.5E-03	1.1E-09	4.3E-09	8.3E-02	9.5E-10
5	rs11948261	5.64×10^{-9}	2.9E-08	9.1E-03	1.1E-09	4.3E-09	1.7E-01	2.0E-09	2.2E-09	2.9E-01	2.0E-09
7	rs2158507	3.03×10^{-9}	9.1E-09	7.9E-02	1.9E-09	4.5E-08	7.6E-03	1.5E-09	7.5E-08	2.2E-03	1.2E-09
7	rs62491417	2.75×10^{-8}	6.6E-07	6.3E-03	2.2E-08	2.5E-07	3.1E-02	2.4E-08	7.2E-08	2.2E-01	4.0E-08
8	rs2952176	2.32×10^{-8}	4.2E-08	2.8E-02	3.6E-09	5.4E-07	1.9E-04	2.7E-09	1.7E-08	1.0E-01	4.4E-09
8	rs16884419	2.77×10^{-9}	7.3E-10	5.8E-01	1.9E-09	2.9E-08	1.2E-02	1.3E-09	2.2E-07	2.1E-04	9.9E-10
9	rs999483	8.33×10^{-10}	2.2E-09	1.2E-01	7.3E-10	8.1E-08	3.3E-04	4.4E-10	3.3E-08	1.1E-03	3.2E-10
16	rs2054213	4.44×10^{-10}	4.1E-09	2.5E-02	3.2E-10	2.4E-10	3.2E-01	2.7E-10	2.7E-08	1.0E-03	2.5E-10
17	rs78454137	2.66×10^{-8}	3.2E-07	2.1E-03	5.4E-09	1.3E-07	1.4E-02	7.3E-09	3.1E-08	1.3E-01	1.0E-08
18	rs1941879	4.63×10^{-9}	1.3E-08	1.5E-01	5.2E-09	1.5E-09	7.3E-01	5.3E-09	9.4E-08	7.5E-03	3.3E-09
18	rs10503002	8.12×10^{-10}	5.7E-09	3.0E-02	5.1E-10	2.1E-09	8.4E-02	4.9E-10	3.8E-10	4.4E-01	6.6E-10
18	rs2628207	2.75×10^{-9}	2.4E-08	1.4E-02	1.2E-09	2.1E-08	1.4E-02	1.1E-09	1.6E-08	3.3E-02	1.6E-09
20	rs13037664	1.83×10^{-10}	4.2E-09	3.2E-03	7.2E-11	4.8E-09	1.5E-03	5.1E-11	8.2E-11	3.4E-01	1.1E-10

CHR	SNPID		Set 4			Set 5			Set 6		
		Overall P	Discovery P	Replication P	Meta-analysis P	Discovery P	Replication P	Meta-analysis P	Discovery P	Replication P	Meta-analysis P
2	rs4953150	4.05×10^{-9}	2.1E-08	5.2E-02	3.1E-09	2.8E-08	2.1E-02	1.9E-09	1.5E-07	2.2E-03	2.4E-09
2	rs7573001	4.07×10^{-8}	6.9E-08	1.1E-01	1.9E-08	2.0E-06	3.1E-04	1.4E-08	1.5E-07	3.7E-02	1.7E-08
5	rs13166120	2.39×10^{-9}	9.2E-10	4.3E-01	1.5E-09	7.4E-08	1.8E-03	1.0E-09	1.5E-08	3.0E-02	1.4E-09
5	rs11948261	5.64×10^{-9}	2.1E-07	1.7E-04	8.4E-10	2.4E-09	2.2E-01	1.5E-09	8.8E-10	6.6E-01	2.9E-09
7	rs2158507	3.03×10^{-9}	6.0E-10	8.1E-01	3.0E-09	9.2E-09	7.5E-02	1.8E-09	1.3E-08	4.9E-02	1.8E-09
7	rs62491417	2.75×10^{-8}	4.9E-07	9.6E-03	2.1E-08	1.1E-07	6.5E-02	1.9E-08	1.5E-07	5.4E-02	2.2E-08
8	rs2952176	2.32×10^{-8}	2.7E-08	9.0E-02	6.2E-09	5.2E-09	2.7E-01	4.1E-09	7.5E-07	1.2E-04	3.1E-09

8	rs16884419	2.77×10^{-9}	4.4E-09	1.1E-01	1.3E-09	1.4E-09	2.2E-01	9.0E-10	1.4E-07	6.2E-04	1.1E-09
9	rs999483	8.33×10^{-10}	6.1E-10	2.5E-01	4.8E-10	6.1E-09	3.0E-02	5.5E-10	2.3E-10	6.7E-01	8.4E-10
16	rs2054213	4.44×10^{-10}	5.2E-08	2.6E-04	2.3E-10	5.4E-10	1.9E-01	3.0E-10	2.5E-10	3.9E-01	3.9E-10
17	rs78454137	2.66×10^{-8}	3.1E-08	1.2E-01	9.4E-09	5.4E-08	3.7E-02	6.0E-09	3.0E-08	1.5E-01	1.1E-08
18	rs1941879	4.63×10^{-9}	9.7E-08	4.7E-03	2.4E-09	4.0E-07	2.9E-04	2.4E-09	3.3E-08	3.0E-02	3.0E-09
18	rs10503002	8.12×10^{-10}	8.4E-09	1.4E-02	4.1E-10	2.2E-08	2.1E-03	3.2E-10	7.8E-09	2.4E-02	5.9E-10
18	rs2628207	2.75×10^{-9}	7.5E-09	8.0E-02	1.6E-09	7.9E-09	6.1E-02	1.3E-09	8.3E-08	3.6E-03	1.8E-09
20	rs13037664	1.83×10^{-10}	8.6E-10	3.0E-02	7.7E-11	2.8E-09	4.1E-03	5.7E-11	8.1E-10	2.2E-02	5.6E-11

CHR	SNPID		Set 7			Set 8			Set 9		
		**Overall P**	**Discovery P**	**Replication P**	**Meta-analysis P**	**Discovery P**	**Replication P**	**Meta-analysis P**	**Discovery P**	**Replication P**	**Meta-analysis P**
2	rs4953150	4.05×10^{-9}	5.9E-09	1.3E-01	2.0E-09	3.7E-09	2.9E-01	3.2E-09	3.0E-09	2.6E-01	2.3E-09
2	rs7573001	4.07×10^{-8}	7.3E-08	1.4E-01	2.5E-08	3.5E-07	1.2E-02	1.7E-08	8.5E-08	9.6E-02	2.1E-08
5	rs13166120	2.39×10^{-9}	8.0E-10	5.5E-01	1.9E-09	7.9E-08	1.4E-03	9.3E-10	2.5E-09	1.7E-01	1.1E-09
5	rs11948261	5.64×10^{-9}	3.4E-08	7.1E-03	1.1E-09	2.2E-08	2.2E-02	1.6E-09	1.0E-07	6.0E-04	7.8E-10
7	rs2158507	3.03×10^{-9}	1.2E-08	5.6E-02	1.8E-09	4.6E-09	1.6E-01	2.0E-09	4.2E-08	1.5E-02	2.3E-09
7	rs62491417	2.75×10^{-8}	2.6E-07	1.7E-02	1.6E-08	7.1E-08	1.3E-01	2.3E-08	4.1E-09	9.9E-01	2.8E-08
8	rs2952176	2.32×10^{-8}	6.3E-08	2.5E-02	5.1E-09	4.8E-08	3.3E-02	4.8E-09	7.3E-08	1.0E-02	3.1E-09
8	rs16884419	2.77×10^{-9}	9.3E-09	2.6E-02	7.5E-10	7.3E-09	5.4E-02	1.1E-09	1.8E-09	1.8E-01	9.1E-10
9	rs999483	8.33×10^{-10}	5.2E-08	4.0E-04	3.0E-10	4.5E-08	9.4E-04	4.0E-10	3.1E-10	4.8E-01	6.4E-10
16	rs2054213	4.44×10^{-10}	1.1E-08	7.1E-03	3.5E-10	5.7E-10	2.4E-01	4.3E-10	5.4E-09	1.0E-02	2.1E-10
17	rs78454137	2.66×10^{-8}	6.6E-07	3.1E-04	4.1E-09	1.4E-08	3.1E-01	1.3E-08	6.2E-08	2.6E-02	5.2E-09
18	rs1941879	4.63×10^{-9}	2.1E-10	5.3E-01	6.6E-09	2.6E-08	4.2E-02	3.1E-09	1.1E-08	1.2E-01	3.3E-09
18	rs10503002	8.12×10^{-10}	1.6E-08	5.2E-03	4.1E-10	2.3E-09	8.3E-02	5.2E-10	7.7E-09	2.5E-02	6.0E-10
18	rs2628207	2.75×10^{-9}	2.0E-09	3.6E-01	2.4E-09	1.0E-07	1.0E-03	1.0E-09	1.4E-08	3.2E-02	1.3E-09
20	rs13037664	1.83×10^{-10}	4.7E-09	3.1E-03	8.1E-11	1.6E-10	1.6E-01	7.6E-11	1.2E-10	2.4E-01	9.5E-11

CHR	SNPID		Set 10		
		**Overall P**	**Discovery P**	**Replication P**	**Meta-analysis P**
2	rs4953150	4.05×10^{-9}	1.5E-07	1.2E-03	1.7E-09

2	rs7573001	4.07×10^{-8}	5.0E-08	1.4E-01	1.8E-08
5	rs13166120	2.39×10^{-9}	5.5E-08	2.7E-03	9.7E-10
5	rs11948261	5.64×10^{-9}	8.8E-08	2.3E-03	1.4E-09
7	rs2158507	3.03×10^{-9}	4.5E-08	4.5E-03	1.1E-09
7	rs62491417	2.75×10^{-8}	9.1E-08	8.4E-02	2.0E-08
8	rs2952176	2.32×10^{-8}	3.1E-08	7.6E-02	6.2E-09
8	rs16884419	2.77×10^{-9}	2.9E-08	5.6E-03	7.9E-10
9	rs999483	8.33×10^{-10}	4.2E-10	3.6E-01	5.6E-10
16	rs2054213	4.44×10^{-10}	2.4E-09	5.3E-02	3.5E-10
17	rs78454137	2.66×10^{-8}	6.6E-08	4.1E-02	7.8E-09
18	rs1941879	4.63×10^{-9}	2.1E-07	1.4E-03	2.7E-09
18	rs10503002	8.12×10^{-10}	1.2E-09	1.4E-01	4.5E-10
18	rs2628207	2.75×10^{-9}	6.3E-10	6.2E-01	1.9E-09
20	rs13037664	1.83×10^{-10}	1.0E-09	1.4E-02	4.9E-11
